# Supplementary material for: Synthesis of aryldifluoromethyl aryl ethers via nickel-catalyzed suzuki cross-coupling between aryloxydifluoromethyl bromides and boronic acids
Source: Commun Chem. 2022 Jul 4;5:78. doi: 10.1038/s42004-022-00694-4 (PMC9814959; doi:10.1038/s42004-022-00694-4)
Supplement: Supplementary file 1 — Supplementary Material [file 42004_2022_694_MOESM1_ESM.pdf]

Supporting Information

Synthesis of Aryldifluoromethyl Aryl Ethers via Nickel-Catalyzed Suzuki Cross-Coupling between Aryloxydifluoromethyl Bromides and Boronic Acids

Heng Lu, Ruo-Xuan Xiao, Chang-Yun Shi, Zi-Lan Song, Hou-Wen Lin, and Ao Zhang*

Table of Contents

1. General experimental information--S1

2. General synthetic procedure for starting material--S2

3. Condition optimization--S9

4. General procedures for the synthesis of compounds---S15

4.1. Representative experimental procedure for the synthesis of compounds 3---------------S15

4.2 Representative experimental procedure for the synthesis of compounds 7----------------S33

4.3 Procedures for the synthesis of PD-1/PD-L1interaction inhibitors-------------------------S39

5. Preliminary mechanistic experiments---S48

5.1 Radical inhibition experiments--S48

5.2 Radical clock experiments---S49

5.3 Studies of the role of DABCO in the reaction---S49

6. References--S52

7. X-Ray crystallographic data ---S53

8. Gating strategy ---S55

Supplementary Methods

1. General experimental information

All reactions were performed in flame-dried glassware, including sealed tubes or Schlenck tubes. Liquids and solutions were transferred with syringes. All solvents and chemical reagents were obtained from commercial sources and used without further purifications. ^{1}H and ^{13}C NMR spectra were recorded with tetramethylsilane as an internal reference. High-resolution mass spectra were recorded on EI-TOF (electrospray ionization-time of flight) or APCI. Flash column chromatography on silica gel (200 - 300 mesh) or RP-C18. The column output was monitored by TLC on silica gel (100 - 200 mesh) precoated on glass plates (15 x 50 mm), and spots were visualized by UV light at 254 nm. Commercially available chemicals were obtained from Acros Organics, Strem Chemicals, Alfa Aesar, Adamas-beta, J&K.

2. General synthetic procedure for starting material

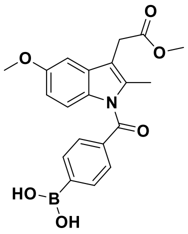
1aa

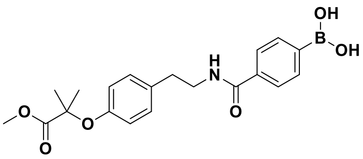
1ab

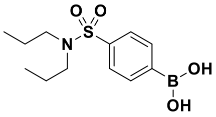
1ac

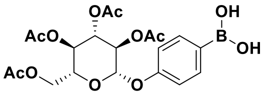
1ad

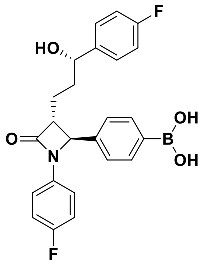
1ae

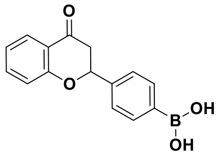
1af

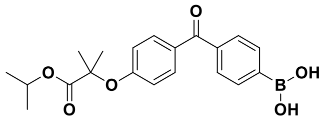
1ag

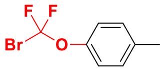
2a

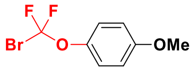
2b

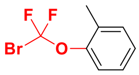
2c

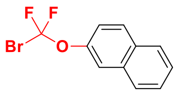
2d

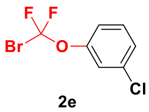
2e

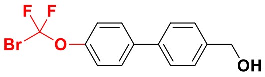
2f

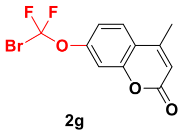
2g

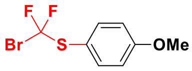
2h

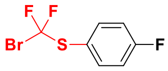
2i

Materials: All reagents were used as received from commercial sources or prepared as described in the literature. All reagents were weighed and handled under air, and refilled with an inert atmosphere of Ar at room temperature. Acetone was dried by 4 Å MS at Ar atmosphere before use. Compounds **1aa**,[1] **1ac**,[2] **1ad**,[3] **1ae**,[4] **1af**,[5] **1ag**,[1] **2a**,[6] **2b**,[7] **2d**,[7] **2h**,[7] and **2i**[8] were prepared according to the literature and all substrates stored at the 4 °C.

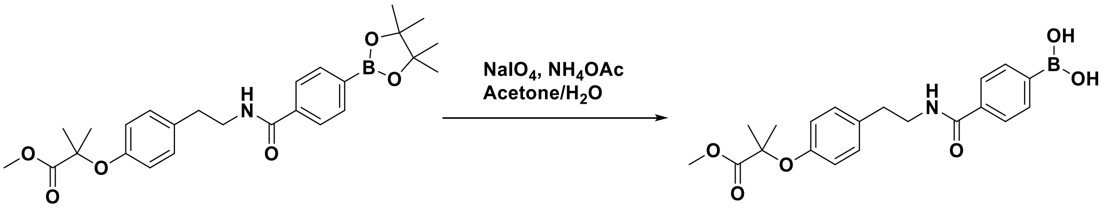

1Abb 1bb

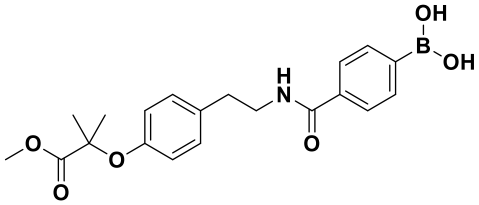

(4-((4-((1-methoxy-2-methyl-1-oxopropan-2-yl)oxy)phenethyl)carbamoyl)phenyl) boronic acid (1ab).

To a 100 mL round-bottom flask were added **1Abb** (385 mg, 1 mmol, 1.0 equiv), $NaIO_4$ (1.3 g, 6 mmol, 6.0 equiv), NH_4OAc (925 mg, 6 mmol, 6.0 equiv), acetone (25 mL), and H_2O (13 mL). After stirring for 48 h at roomtemperature, the reaction mixture was filtered with a pad of cellite. The filtrate was concentrated, theresulting solid was washed with H_2O (6 mL), pentane (6 mL×3), and dried in vacuo to afford the corresponding boronic acid as a white solid, which was used for next step without further purification.

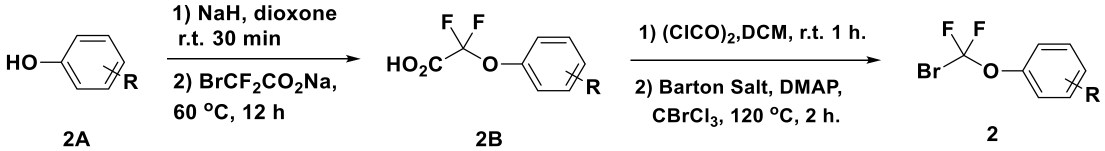

To a 25 mL oven-dried Schlenk tube equipped with a stir bar were added phenol **2A** (4 mmol, 1.0 equiv) under Ar atmosphere, 1,4-dioxane (20 mL) was added to dissolve the phenol. Then NaH (60% purity) (176 mg, 4.4 mmol, 1.1 equiv) and 1,4-dioxane (2 mL) were added with stirring at room temperature under Ar. The solution was stirred at room temperature for 30 min, then $BrCF_2COONa$ (867 mg, 4.4 mmol, 1.1 equiv) and 1,4-dioxane (3 mL) were added. After the mixture was heated at 60 ºC for 12 h. After the mixture was heated for hours (monitor by TLC), then cooled down to room temperature and acidified with 3M HCl (aq) to pH = 1. The mixture was extracted with ethyl acetate for 3 times. The combined organic phase was washed by saturated brines and dried over $NaSO_4$. After the solution was filtered and the solvent was evaporated under vacuum, and dried in vacuo to afford the corresponding carboxylic acid **2B** as a white solid, which was used for next step without further purification.[10]

To a 25 mL of round-bottom flask were added carboxylic acid **2B** (4.0 mmol) in DCM (8 mL), then added DMF (31 µL, 0.4 mmol, 0.1 equiv) and oxalyl chloride (516 µL, 6

mmol, 1.5 equiv) at 0 °C. The reaction mixture was stirred at room temperature for 1 hours, then concentrated in vacuo. The crude acyl chloride was added $BrCCl_3$ (12 mL), 4-Dimethylaminopyridine (98 mg, 0.8 mmol, 0.2 equiv) and sodium-*N*-hydroxy-2-thiopyridone (600 mg, 4.0 mmol). The reaction mixture was refluxed at 120 °C for 2 hours under Ar then concentrated in vacuo. The crude product **2** was purified by silica gel column chromatography.[7]

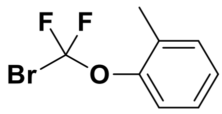

1-(bromodifluoromethoxy)-2-methylbenzene (2c).

The product was purified with silica gel chromatography (Petroleum ether /Ethyl Acetate = 200:1) as colorless oil-like liquid (451 mg, 48% yield). **^{1}H NMR** (400 MHz, $CDCl_3$) δ 7.28-7.18 (m, 4H), 2.32 (s, 3H). **^{19}F NMR** (376 MHz, $CDCl_3$) δ -14.15 (s, 2F). **^{13}C NMR** (101 MHz, $CDCl_3$) δ 149.67(t, J = 1.5 Hz), 131.65, 130.83, 127.05, 126.80, 120.96(t, J = 2.0 Hz), 114.59(t, J = 309.6 Hz), 16.43. **HRMS** (EI): m/z $[(M)^{\cdot+}]$ calcd for $C_8H_7{}^{79}BrF_2O$, 235.9648. found, 235.9654.

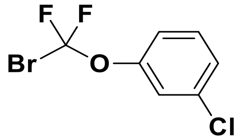

1-(bromodifluoromethoxy)-3-chlorobenzene (2e).

The product was purified with silica gel chromatography (Petroleum ether /Ethyl Acetate = 200:1) as colorless oil-like liquid (540 mg, 53% yield). **^{1}H NMR** (400 MHz, $CDCl_3$) δ 7.39-7.31 (m, 2H), 7.27 (s, 1H), 7.19-7.14 (m, 1H). **^{19}F NMR** (376 MHz, $CDCl_3$) δ -16.06 (s, 2F). **^{13}C NMR** (101 MHz, $CDCl_3$) δ 151.06(t, J = 2.0 Hz), 135.12, 130.52, 127.35, 122.03, 119.64, 114.27(t, J = 311.1 Hz). **HRMS** (EI): m/z $[(M)^{\cdot+}]$ calcd for $C_7H_4{}^{79}Br^{35}ClF_2O$, 255.9102. found, 255.9108.

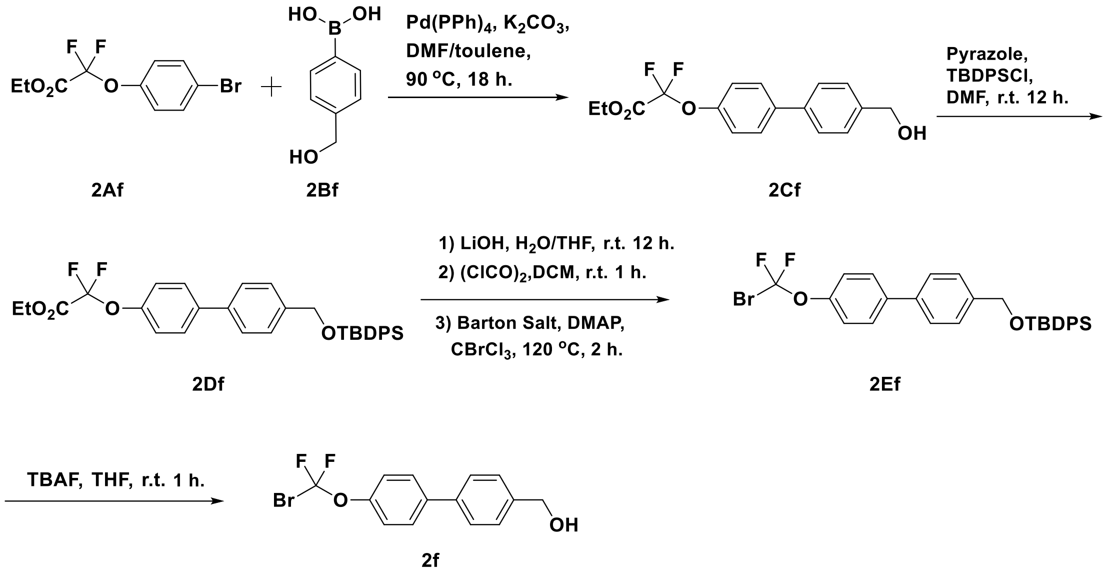

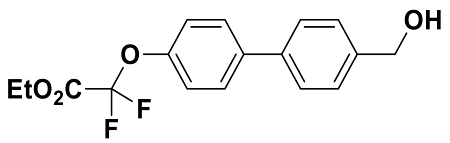

ethyl 2,2-difluoro-2-((4'-(hydroxymethyl)-[1,1'-biphenyl]-4-yl)oxy)acetate (2Cf). To a 100 mL of Schlenck tube were added ethyl 2-(4-bromophenoxy)-2,2-difluoroacetate **2Af**[7] (2.9 g, 10 mmol, 1.0 equiv), phenylboronic acid **2Bf** (2.3 g, 15 mmol, 1.5 equiv), $P(PPh_3)_4$ (1.1 g, 1 mmol, 5 mol %), and K_2CO_3 (2.6 g, 20 mmol, 2.0 equiv). The reaction mixture was then evacuated and backfilled with Ar (3 times). DMF (10 mL) and toluene (20 mL) were added subsequently. The tube was screw capped and put into a preheated oil bath (100 °C). After stirring for 18 h, the reaction mixture was cooled to room temperature. Water (100 mL) was added and the mixture was extracted with EA (30 mL×3). The combined organic layers were washed with brine, dried over Na_2SO_4, and concentrated under reduced pressure. The residue was purified by flash column chromatography (Petroleum ether /Ethyl Acetate = 5:1) as a white solid (1.9 g, 61% yield). **^{1}H NMR** (400 MHz, $CDCl_3$) δ 7.59-7.54 (m, 4H), 7.44 (d, J = 8.1, 2H), 7.29 (d, J = 8.6, 2H), 4.74 (s, 2H), 4.40 (q, J = 7.1, 2H), 1.80 (s, 1H), 1.38 (t, J = 7.2, 3H). **^{19}F NMR** (376 MHz, $CDCl_3$) δ -76.26 (s, 2F). **^{13}C NMR** (101 MHz, $CDCl_3$) δ 159.84(t, J = 41.4 Hz), 148.92, 140.21, 139.40, 139.07, 128.21, 127.52, 127.25, 121.97, 114.01(t, J = 273.2 Hz), 65.01, 63.75, 13.87. **HRMS** (APCI): m/z [$(M-OH)^+$] calcd for $C_{17}H_{15}F_2O_3$, 305.0984. found, 305.0987.

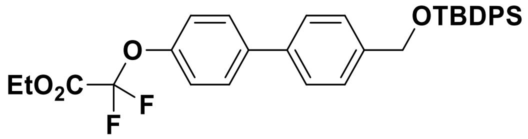

ethyl 2-((4'-(((tert-butyldiphenylsilyl)oxy)methyl)-[1,1'-biphenyl]-4-yl)oxy)-2,2-difluoroacetate (2Df).

To a 100 mL of round-bottom flask were added **2Cf** (1.8 g, 6 mmol, 1.0 equiv), pyrazole (0.6 g, 9 mmol, 1.5 equiv), TBDPSCl (2.1 g, 7.8 mmol, 1.3 equiv), DMF (10 mL). After stirring for 12 h at room temperature, Water (50 mL) was added and the mixture was extracted with EA (15 mL×3). The combined organic layers were washed with brine, dried over Na_2SO_4, and concentrated under reduced pressure. The residue was purified by flash column chromatography (Petroleum ether /Ethyl Acetate = 20:1) as a yellow oil-like liquid (3.2 g, 96% yield). **^{1}H NMR** (400 MHz, $CDCl_3$) δ 7.76-7.71 (m, 4H), 7.62-7.58 (m, 2H), 7.55 (d, J = 8.2, 2H), 7.47-7.38 (m, 8H), 7.30 (d, J = 8.6, 2H), 4.84 (s, 2H), 4.42 (q, J = 7.1, 2H), 1.39 (t, J = 7.2, 3H), 1.13 (s, 9H). **^{19}F NMR** (376 MHz, $CDCl_3$) δ -76.20 (s, 2F). **^{13}C NMR** (101 MHz, $CDCl_3$) δ 159.87(t, J = 41.4 Hz), 148.80(t, J = 2.0 Hz), 140.52, 139.36, 138.62, 135.61, 133.49, 129.76, 128.18, 127.77, 126.94, 126.52, 121.94, 114.05(t, J = 274.2 Hz), 65.28, 63.72, 26.89, 19.37, 13.89. **HRMS** (EI): m/z [$(M)^{\cdot+}$] calcd for $C_{33}H_{34}F_2O_4Si$, 560.2194. found, 560.2187.

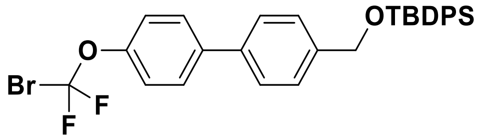

((4'-(bromodifluoromethoxy)-[1,1'-biphenyl]-4-yl)methoxy)(tert-butyl)diphenylsilane (2Ef).

To a 25 mL of round-bottom flask equipped with a stir bar were added **2Df** (2.2 g, 4 mmol, 1.0 equiv), $LiOH\cdot H_2O$ (336 mg, 8 mmol, 2.0 equiv), THF (5 mL) and H_2O (5 mL). After the mixture was stired at room temperature for 12 h. The mixture was acidified with 3M HCl (aq) to pH = 1 and was extracted with ethyl acetate for 3 times.

The combined organic phase was washed by saturated brines and dried over $NaSO_4$. After the solution was filtered and the solvent was evaporated under vacuum, and dried in vacuo to afford the corresponding carboxylic acid as a white solid, which was used for next step without further purification

To a 25 mL of round-bottom flask were added carboxylic acid in DCM (8 mL), then added DMF (31 μL, 0.4 mmol, 0.1 equiv) and oxalyl chloride (516 μL, 6 mmol, 1.5 equiv) at 0 ºC. The reaction mixture was stirred at room temperature for 1 hours, then concentrated in vacuo. The crude acyl chloride was added $BrCCl_3$ (12 mL), 4-Dimethylaminopyridine (98 mg, 0.8 mmol, 0.2 equiv) and sodium-*N*-hydroxy-2-thiopyridone (600 mg, 4.0 mmol). The reaction mixture was refluxed at 120 ºC for 2 hours under Ar then concentrated in vacuo. The crude product **2** was purified by silica gel column chromatography (Petroleum ether /Ethyl Acetate = 30:1) as a colorless oil-like liquid (1.5 g, 65% yield). **^{1}H NMR** (400 MHz, $CDCl_3$) δ 7.73-7.69 (m, 4H), 7.64-7.59 (m, 2H), 7.54 (d, J = 8.2, 2H), 7.44-7.37 (m, 8H), 7.30 (d, J = 8.7, 2H), 4.82 (s, 2H), 1.12 (s, 9H). **^{19}F NMR** (376 MHz, $CDCl_3$) δ -15.41 (s, 2F). **^{13}C NMR** (101 MHz, $CDCl_3$) δ 150.11(t, J = 2.0 Hz), 140.72, 140.04, 138.41, 135.61, 133.47, 129.77, 128.30, 127.78, 126.97, 126.55, 121.65, 114.67(t, J = 310.1 Hz), 65.26, 26.89, 26.80, 19.37. **HRMS** (EI): m/z [$(M)^{\cdot+}$] calcd for $C_{30}H_{29}{}^{79}BrF_2O_2Si$, 566.1088. found, 566.1080.

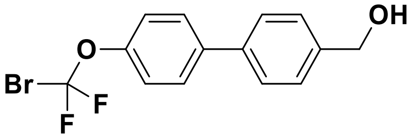

(4'-(bromodifluoromethoxy)-[1,1'-biphenyl]-4-yl)methanol (2f).

To a 25 mL of round-bottom flask were added **2Ef** (1.4 g, 2.5 mmol, 1.0 equiv), TBATF (4 mL, 4 mmol, 1.6 equiv, 1M in THF), THF (10 mL). After stirring for 12 h at room temperature, solvent was concentrated under reduced pressure. The residue was purified by flash column chromatography (Petroleum ether /Ethyl Acetate = 5:1) as a white solid (714 mg, 92% yield). **^{1}H NMR** (400 MHz, $CDCl_3$) δ 7.61 (d, J = 8.7, 2H), 7.57 (d, J = 8.2, 2H), 7.45 (d, J = 8.2, 2H), 7.32 (d, J = 8.7, 2H), 4.74 (s, 2H), 2.11 (s, 1H). **^{19}F NMR** (376 MHz, $CDCl_3$) δ -15.44 (s, 2F). **^{13}C NMR** (101 MHz, $CDCl_3$) δ

150.23(t, J = 2.0 Hz), 140.41, 139.75, 139.14, 128.34, 127.56, 127.26, 121.69, 114.64(t, J = 310.1 Hz), 64.91. **HRMS** (APCI): m/z [$(M-OH)^+$] calcd for $C_{14}H_{10}{}^{79}BrF_2O_1$, 310.9877. found, 310.9877.

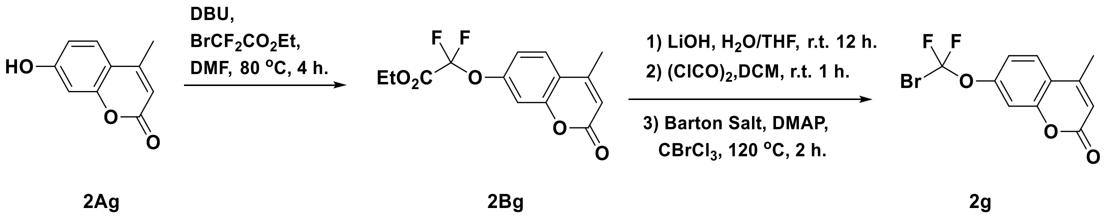

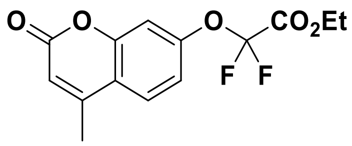

ethyl 2,2-difluoro-2-((4-methyl-2-oxo-2H-chromen-7-yl)oxy)acetate (2Bg).

To a 25 mL of round-bottom flask were added **2Af** (530 mg, 3 mmol, 1.0 equiv), DBU (684 mg, 4.5 mmol, 1.5 equiv), $BrCF_2CO_2Et$ (1.2 g, 6 mmol, 2.0 equiv), DMF (12 mL). After stirring for 4 h at 80 °C, and solvent was concentrated under reduced pressure. The residue was purified by flash column chromatography (Petroleum ether /Ethyl Acetate = 8:1) as a colorless oil-like liquid (730 mg, 81% yield). **^{1}H NMR** (400 MHz, $CDCl_3$) δ 7.60 (d, J = 8.7, 1H), 7.23-7.13 (m, 2H), 6.28 (d, J = 1.1, 1H), 4.41 (q, J = 7.1, 2H), 2.43 (d, J = 1.2, 3H), 1.39 (t, J = 7.2, 3H). **^{19}F NMR** (376 MHz, $CDCl_3$) δ -76.77 (s, 2F). **^{13}C NMR** (101 MHz, $CDCl_3$) δ 160.21, 159.18(t, J = 40.9 Hz), 154.14, 151.77(t, J = 2.0 Hz), 151.67, 125.69, 118.06, 117.25, 114.75, 113.77(t, J = 275.7 Hz), 109.82, 64.01, 18.66, 13.83. **HRMS** (APCI): m/z [$(M+H)^+$] calcd for $C_{14}H_{13}F_2O_5$, 299.0726. found, 299.0732.

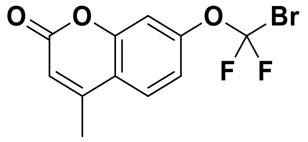

7-(bromodifluoromethoxy)-4-methyl-2H-chromen-2-one (2g).

To a 25 mL of round-bottom flask equipped with a stir bar were added **2Bg** (600 mg, 2 mmol, 1.0 equiv), $LiOH{\cdot}H_2O$ (170 mg, 4 mmol, 2.0 equiv), THF (3 mL) and H_2O (3

mL). After the mixture was stired at room temperature for 12 h. The mixture was acidified with 3M HCl (aq) to pH = 1 and was extracted with ethyl acetate for 3 times. The combined organic phase was washed by saturated brines and dried over $NaSO_4$. After the solution was filtered and the solvent was evaporated under vacuum, and dried in vacuo to afford the corresponding carboxylic acid as a white solid, which was used for next step without further purification

To a 25 mL of round-bottom flask were added carboxylic acid in DCM (4 mL), then added DMF (15 µL, 0.2 mmol, 0.1 equiv) and oxalyl chloride (260 µL, 3 mmol, 1.5 equiv) at 0 °C. The reaction mixture was stirred at room temperature for 1 hours, then concentrated in vacuo. The crude acyl chloride was added $BrCCl_3$ (6 mL), 4-Dimethylaminopyridine (49 mg, 0.4 mmol, 0.2 equiv) and sodium-*N*-hydroxy-2-thiopyridone (300 mg, 2.0 mmol). The reaction mixture was refluxed at 120 °C for 2 hours under Ar then concentrated in vacuo. The crude product **2** was purified by silica gel column chromatography (Petroleum ether /Ethyl Acetate = 20:1) as a white solid (354 mg, 58% yield). **^{1}H NMR** (400 MHz, $CDCl_3$) δ 7.65 (d, J = 8.7, 1H), 7.25-7.16 (m, 2H), 6.30 (d, J = 1.1, 1H), 2.45 (d, J = 1.2, 3H). **^{19}F NMR** (376 MHz, $CDCl_3$) δ -16.31 (s, 2F). **^{13}C NMR** (101 MHz, $CDCl_3$) δ 160.03, 154.16, 152.55(t, J = 2.0 Hz), 151.61, 125.93, 118.69, 117.11, 115.16, 113.89(t, J = 312.1 Hz), 109.87, 18.72. **HRMS** (APCI): m/z $[(M+H)^+]$ calcd for $C_{11}H_7{}^{79}BrF_2O_3$, 304.9620. found, 304.9629.

3. Condition optimization

Table S1. Screening of Reported Methods about Difluoroalkylation Reaction of Arylboronic Acids[a]

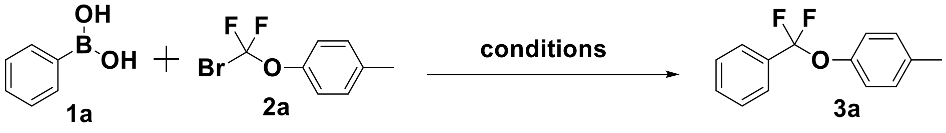

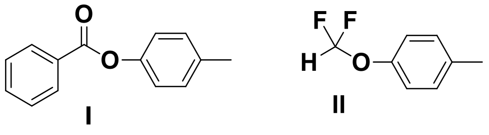

Entry	methods	Yield [%][b]		
		3a	I	II

1[11]	$Pd(OAc)_2$, $PAd_2{}^nBu$, K_2CO_3, dioxone, 70 °C, 7 h.	-	-	-
2[12]	CuI, tpy, Cs_2CO_3, toluene, 100 °C, 18 h..	-	-	26
3[13]	$CoBr_2$. DME, L_1 in article, $ZnBr_2$, DME, 80 °C, 12 h.	-	-	-
4[14]	$Ni(PPh_3)Br_2$, bpy, DMAP, K_2CO_3, THF, 80 °C, 17 h.	-	-	11
5[15]	$NiCl_2$· DME, dtbbpy, DMAP, K_2CO_3, triglyme, 80 °C, 12 h.	-	-	-
6[16]	$Ni(acac)_2$, PPh_3, K_2CO_3, DCM, 100 °C, 24 h	-	-	5
7	$Ni(diMebpy)_2Br_2$, DABCO, K_2CO_3, acetone, 80 °C, 24 h	90	6	-

[a] Reaction condition A: **1a** (0.4 mmol), **2a** (0.2 mmol). [b] The yields were determined by GC-MS with n-dodecane as an internal standard.

Table S2. Screening of the Solvent[a]

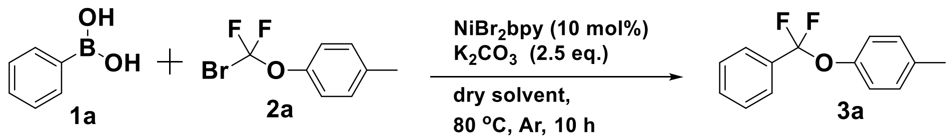

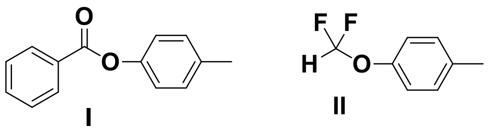

Entry	Solvent	Yield [%][b]		
		3a	I	II
1	NMP	-	-	-
2	DMF	24	8	41
3	DMSO	-	-	-
4	Dioxone	-	-	37
5	Acetone	45	10	-
6	EA	30	33	26
7	MeCN	42	19	11
8	DCE	-	-	-
9	Toulene	-	-	25
10	MeOH	-	-	-
11[c]	Acetone	25	16	-
12[d]	Acetone	40	12	-

[a] Reaction condition A: **1a** (0.4 mmol), **2a** (0.2 mmol), $NiBr_2bpy$ (10 mol%), K_2CO_3 (0.5 mmol),

solvent (3 mL), 80 ºC, 10 h. [b] The yields were determined by GC-MS with n-dodecane as an internal standard. [c] Using 2.0 ml solvent. [d] Using 4.0 ml solvent.

Table S3. Screening of the Ni Catalyst and Ligand [a]

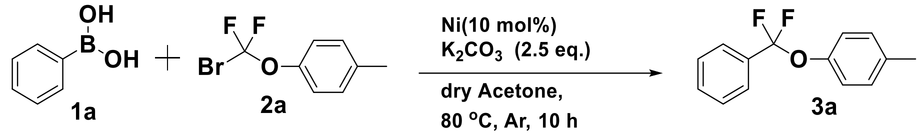

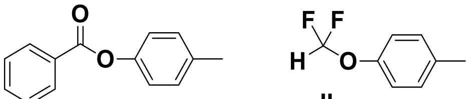

I II

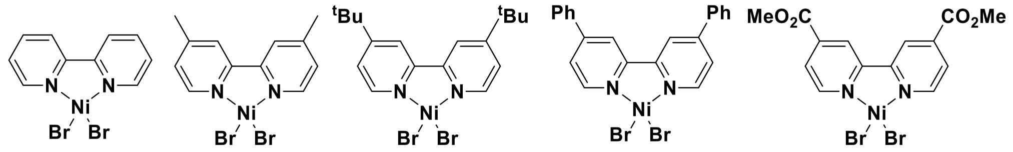

Ni-1 Ni-2 Ni-3 Ni-4 Ni-5

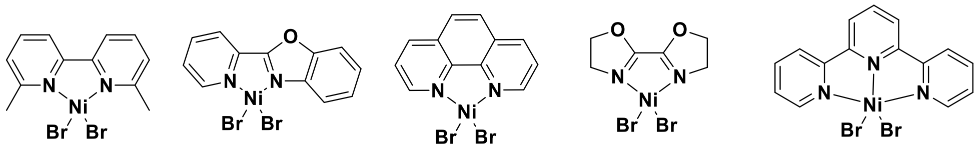

Ni-6 Ni-7 Ni-8 Ni-9 Ni-10

Entry	Catalyst	Yield [%][b]		
		3a	I	II
1	Ni-1	45	10	-
2	Ni-2	55	15	-
3	Ni-3	51	19	5
4	Ni-4	36	10	-
5	Ni-5	38	21	-
6	Ni-6	-	-	-
7	Ni-7	-	-	-
8	Ni-8	32	17	18
9	Ni-9	-	-	-
10	Ni-10	-	-	15
11	$NiCl_2$dppf	-	-	-
12	$NiCl_2$dppe	-	-	-
13[c]	Ni-2	40	6	-

[a] Reaction condition A: **1a** (0.4 mmol), **2a** (0.2 mmol), $NiBr_2L$ (10 mol%), K_2CO_3 (0.5 mmol), solvent (3 mL), 80 ºC, 10 h. [b] The yields were determined by GC-MS with n-dodecane as an

internal standard. [c] Using 5 mol%l Ni-2 as catalyst. .

Preparation of $NiBr_2L$:

To a stirring solution of corresponding L (1.0 mmol, 1.0 equiv) in EtOH (5 mL) was added a solution of $NiBr_2$·DME (308 mg, 1.0 mmol, 1.0 equiv) in EtOH (5 mL) dropwise, the reaction mixture was stirred at 80 °C for another 8 h. After cooled down to room temperature, the solvent was evaporated in vacuo to give corresponding solid, the solid was recrystallized from EtOH.

Table S4. Screening of the Ni Additive [a]

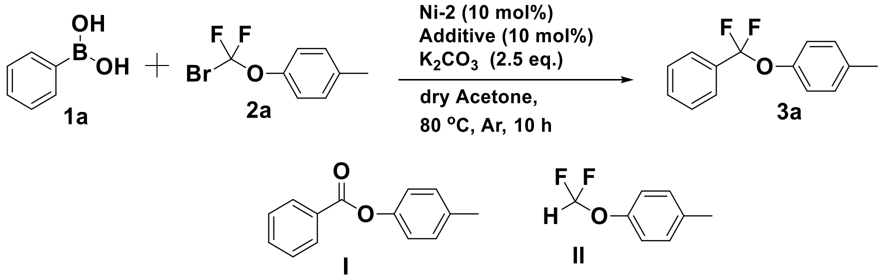

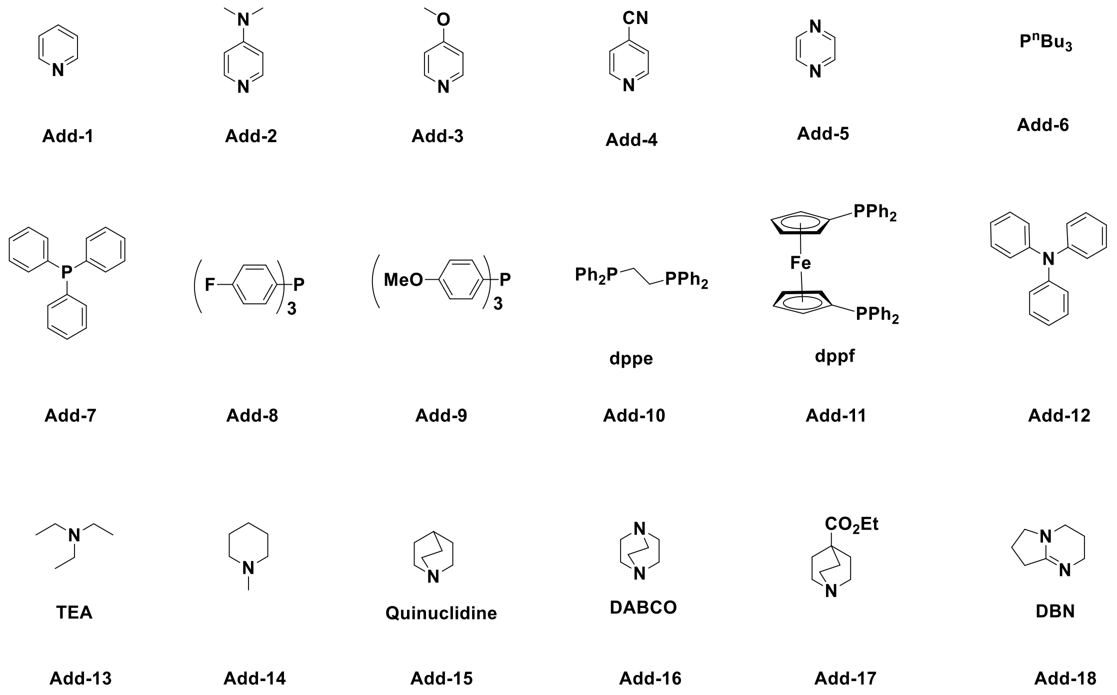

Entry	Additive	Yield [%][b]		
		3a	I	II
1	Add-1	67	12	5
2	Add-2	83	14	-
3	Add-3	74	10	6
4	Add-4	50	18	16
5	Add-5	56	15	12
6	Add-6	36	12	19

7	Add-7	59	10	21
8	Add-8	33	5	28
9	Add-9	68	15	16
10	Add-10	14	-	15
11	Add-11	5	-	11
12	Add-12	60	13	11
13	Add-13	71	8	-
14	Add-14	75	15	-
15	Add-15	82	6	-
16	Add-16	90	6	-
17	Add-17	88	5	-
18	Add-18	18	12	9

[a] Reaction condition A: **1a** (0.4 mmol), **2a** (0.2 mmol), Ni-2 (10 mol%), Additive(10 mol%), K_2CO_3 (0.5 mmol), Acetone (3 mL), 80 °C, 10 h. [b] The yields were determined by GC-MS with n-dodecane as an internal standard.

Table S5. Screening of the Base [a]

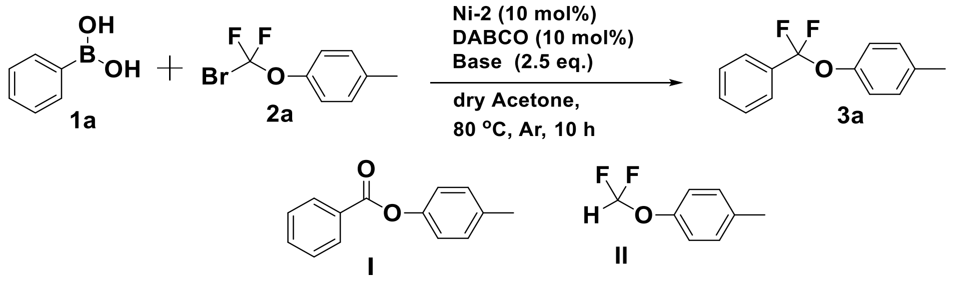

Entry	Base	Yield [%][b]		
		3a	I	II
1	K_2CO_3	90	6	-
2	K_3PO_4	84	8	-
3	KOAc	-	-	34
4	KOtBu	45	12	-
5	Cs_2CO_3	61	13	24
6	Na_2CO_3	80	6	-
7	$PhCO_2Na$	-	10	21
8	DBU	-	-	19
9	DIPEA	-	-	-

[a] Reaction condition A: **1a** (0.4 mmol), **2a** (0.2 mmol), Ni-2 (10 mol%), DABCO (10 mol%), base (0.5 mmol), Acetone (3 mL), 80 °C, 10 h. [b] The yields were determined by GC-MS with n-

dodecane as an internal standard.

Table S6. Screening of the Temperature [a]

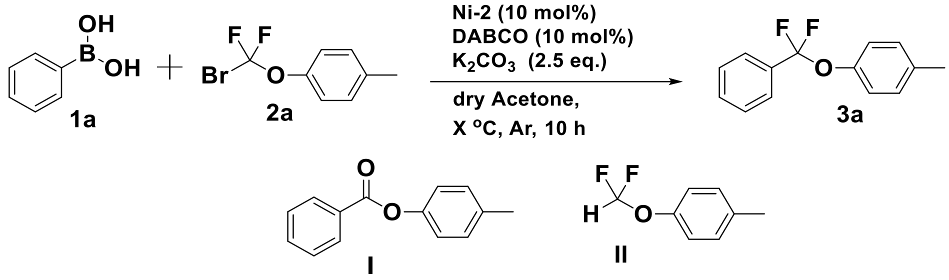

Entry	Temperature	Yield [%][b]		
		3a	I	II
1	60 °C	33	9	10
2	70 °C	86	15	-
3	80 °C	90	6	-
4	90 °C	82	16	-
5	100 °C	51	10	-

[a] Reaction condition A: **1a** (0.4 mmol), **2a** (0.2 mmol), Ni-2 (10 mol%), DABCO (10 mol%), K_2CO_3 (0.5 mmol), Acetone (3 mL), X °C, 10 h. [b] The yields were determined by GC-MS with n-dodecane as an internal standard.

Table S7. Screening of the Boric Acid[a]

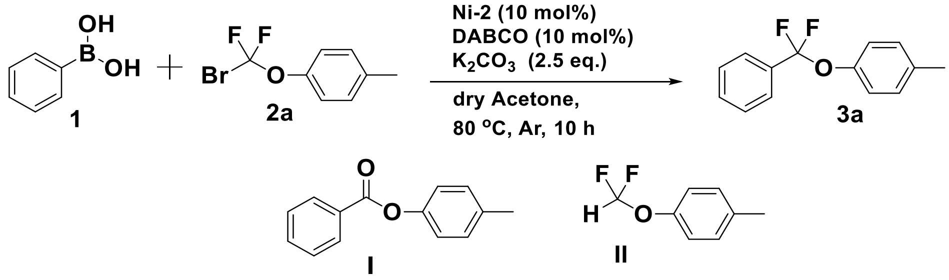

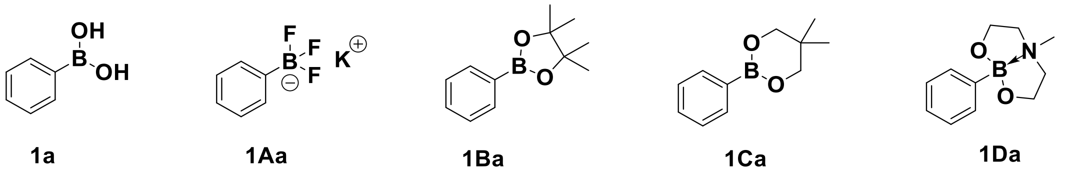

Entry	Boric Acid	Yield [%][b]		
		3a	I	II
1	1a	90	6	-
2	1Aa	-	-	18
3	1Ba	-	-	15
4	1Ca	21	16	11

5	1Da	-	-	-

[a] Reaction condition A: **boric acid** (0.4 mmol), **2a** (0.2 mmol), Ni-2 (10 mol%), DABCO (10 mol%), K_2CO_3 (0.5 mmol), Acetone (3 mL), 80 °C, 10 h. [b] The yields were determined by GC-MS with n-dodecane as an internal standard.

4. General procedures for the synthesis of compounds

4.1 Representative experimental procedure for the synthesis of compounds 3

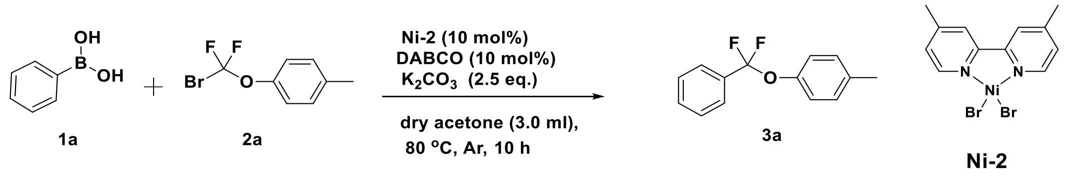

To a dried 10 ml Schlenk-type tube equipped with a magnetic stir bar was charged with arylboronic acid **1a** (49.0 mg, 0.4 mmol, 2.0 equiv), Ni-2 (8.0 mg, 0.02 mmol, 10 mol %), DABCO (2.3 mg, 0.02 mmol, 10 mol %) and K_2CO_3 (69.0 mg, 0.5 mmol, 2.5 equiv) under air. The reaction mixture was then evacuated and backfilled with Ar (3 times). 1-(bromodifluoromethoxy)-4-methylbenzene **2a** (47.0 mg, 0.2mmol, 1.0 equiv), and acetone (3 mL) were added. The mixture was stirred at 80 °C for 10 h. After cooled to room temperature, the reaction mixture was filtered and the filtrate was concentrated. The residue was purified on a preparative TLC with petroleum ether/ethyl acetate as the eluent to afford the products **3a.**

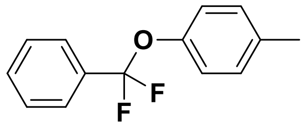

1-(difluoro(phenyl)methoxy)-4-methylbenzene (3a).

Following the general procedure, preparative TLC (petroleum ether/ethyl acetate = 200/1) to afford **3a** as a colorless oil (40.2 mg, 86%). Known compound.[17] **^{1}H NMR** (400 MHz, $CDCl_3$) δ 7.78-7.72 (m, 2H), 7.53-7.44 (m, 3H), 7.17 (m, 4H), 2.35 (s, 3H).

^{19}F NMR (376 MHz, $CDCl_3$) δ -65.41 (s, 2F). **^{13}C NMR** (101 MHz, $CDCl_3$) δ 148.21, 135.26, 133.91(t, J = 47.7 Hz), 130.75(t, J = 1.7 Hz), 129.85, 128.41, 125.58(t, J = 4.0 Hz), 122.18(t, J = 262.2 Hz), 121.86, 20.80. **HRMS** (EI): m/z [$(M)^{\cdot+}$] calcd for $C_{14}H_{12}F_2O$, 234.0856. found, 234.0862.

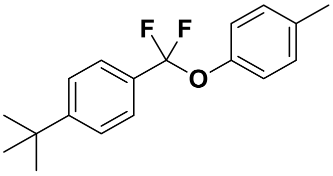

1-(tert-butyl)-4-(difluoro(p-tolyloxy)methyl)benzene (3b).

Following the general procedure, preparative TLC (petroleum ether/ethyl acetate = 200/1) to afford **3b** as a colorless oil (48.1 mg, 83%). **^{1}H NMR** (400 MHz, $CDCl_3$) δ 7.69 (d, J = 8.3, 2H), 7.50 (d, J = 8.4, 2H), 7.22-7.13 (m, 4H), 2.36 (s, 3H), 1.36 (s, 9H). **^{19}F NMR** (376 MHz, $CDCl_3$) δ -65.01 (s, 2F). **^{13}C NMR** (101 MHz, $CDCl_3$) δ 153.99(t, J = 1.4 Hz), 148.32, 135.11, 131.11(t, J = 32.2 Hz), 129.81, 125.35, 125.31(t, J = 3.8 Hz), 122.32(t, J = 261.0 Hz), 121.82, 34.82, 31.20, 20.77. **HRMS** (EI): m/z [$(M)^{\cdot+}$] calcd for $C_{18}H_{20}F_2O$, 290.1482. found, 290.1471.

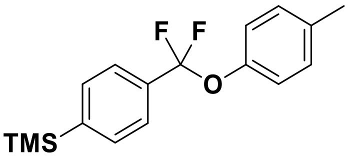

(4-(difluoro(p-tolyloxy)methyl)phenyl)trimethylsilane (3c).

Following the general procedure, dry acetone (2.5 ml) and DMF (0.5 ml) were used for solvent. Preparative TLC (petroleum ether/ethyl acetate = 200/1) to afford **3c** as a colorless oil-like liquid (49.5 mg, 81% yield). **^{1}H NMR** (400 MHz, $CDCl_3$) δ 7.72 (d, J = 8.1, 2H), 7.63 (d, J = 8.1, 2H), 7.21-7.14 (m, 4H), 2.35 (s, 3H), 0.31 (s, 9H). **^{19}F NMR** (376 MHz, $CDCl_3$) δ -65.63 (s, 2F). **^{13}C NMR** (101 MHz, $CDCl_3$) δ 148.27, 144.01, 135.17, 134.19(t, J =31.3 Hz), 133.35, 129.83, 124.65(t, J = 3.9 Hz), 122.23(t,

J = 263.0 Hz), 121.80, 20.80, -1.28. **HRMS** (EI): m/z [(M)$^{\cdot+}$] calcd for $C_{17}H_{20}F_2OSi$, 306.1251. found, 206.1245.

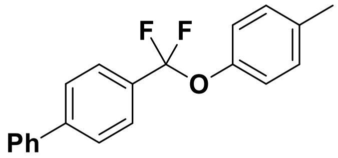

4-(difluoro(p-tolyloxy)methyl)-1,1'-biphenyl (3d).

Following the general procedure, dry acetone (2.5 ml) and DMF (0.5 ml) were used for solvent. Preparative TLC (petroleum ether/ethyl acetate = 200/1) to afford **3d** as a white solid (49.0 mg, 79% yield). ^{1}**H NMR** (400 MHz, $CDCl_3$) δ 7.82 (d, J = 8.6, 2H), 7.69 (d, J = 8.3, 2H), 7.62 (m, 2H), 7.50-7.45 (m, 2H), 7.43-7.38 (m, 1H), 7.23-7.14 (m, 4H), 2.36 (s, 3H). ^{19}**F NMR** (376 MHz, $CDCl_3$) δ -65.05 (s, 2F). ^{13}**C NMR** (101 MHz, $CDCl_3$) δ 148.24, 143.67(t, J = 1.2 Hz), 140.13, 135.24, 132.75(t, J = 32.0 Hz), 129.86, 128.87, 127.88, 127.24, 127.14, 126.07(t, J = 3.7 Hz), 122.25(t, J = 262.1 Hz), 121.83, 20.79. **HRMS** (EI): m/z [(M)$^{\cdot+}$] calcd for $C_{20}H_{16}F_2O$, 310.1169. found, 310.1161.

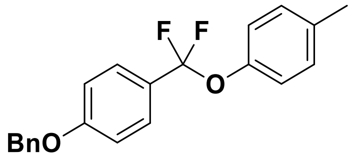

1-(benzyloxy)-4-(difluoro(p-tolyloxy)methyl)benzene (3e).

Following the general procedure, dry acetone (2.5 ml) and DMF (0.5 ml) were used for solvent and 100 mg 4 Å MS was added. Preparative TLC (petroleum ether/ethyl acetate = 100/1) to afford **3e** as a white solid (55.2 mg, 81% yield). ^{1}**H NMR** (400 MHz, DMSO-d_6) δ 7.66 (d, J = 8.8, 2H), 7.46 (d, J = 7.1, 2H), 7.40 (t, J = 7.3, 2H), 7.34 (t, J = 7.1, 1H), 7.20 (d, J = 8.5, 2H), 7.15 (m, 4H), 5.17 (s, 2H), 2.28 (s, 3H). ^{19}**F NMR** (376 MHz, DMSO-d_6) δ -62.27 (s, 2F). ^{13}**C NMR** (101 MHz, DMSO-d_6) δ 160.30, 147.73, 136.57, 135.13, 130.03, 128.50, 127.96, 127.75, 127.18(t, J = 3.5 Hz), 125.17(t, J = 32.4 Hz), 122.37(t, J = 260.0 Hz), 121.62, 114.86, 69.42, 20.29. **HRMS** (EI): m/z [(M)$^{\cdot+}$] calcd for $C_{21}H_{18}F_2O_2$, 340.1275. found, 340.1269.

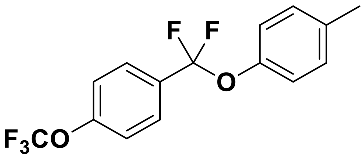

1-(difluoro(4-(trifluoromethoxy)phenyl)methoxy)-4-methylbenzene (3f).

Following the general procedure, dry acetone (2.5 ml) and DMF (0.5 ml) were used for solvent and 100 mg 4 Å MS was added. Preparative TLC (petroleum ether/ethyl acetate = 100/1) to afford **3f** as a colorless oil-like liquid (45.3 mg, 71% yield). **^{1}H NMR** (400 MHz, $CDCl_3$) δ 7.78 (d, J = 8.9, 2H), 7.30 (d, J = 8.3, 2H), 7.19-7.11 (m, 4H), 2.34 (s, 3H). **^{19}F NMR** (376 MHz, $CDCl_3$) δ -57.85 (s, 3F), -65.04 (s, 2F). **^{13}C NMR** (101 MHz, $CDCl_3$) δ 150.88, 147.98, 135.53, 132.56(t, =32.7 Hz), 129.93, 127.57(t, J = 3.7 Hz), 121.81, 121.55(t, J = 262.4 Hz),120.69, 120.37(q, J =259.5 Hz), 20.77. **HRMS** (EI): m/z [$(M)^{\cdot+}$] calcd for $C_{15}H_{11}F_5O_2$, 318.0679. found, 318.0681.

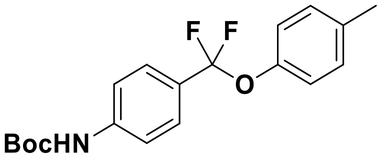

tert-butyl (4-(difluoro(p-tolyloxy)methyl)phenyl)carbamate (3g).

Following the general procedure, dry acetone (2.5 ml) and DMF (0.5 ml) were used for solvent and 100 mg 4 Å MS was added. Preparative TLC (petroleum ether/ethyl acetate = 20/1) to afford **3g** as a white solid (53.6 mg, 77% yield). **^{1}H NMR** (400 MHz, DMSO-d_6) δ 9.68 (s, 1H), 7.61 (m, 4H), 7.20 (d, J = 8.5, 2H), 7.14 (d, J = 8.6, 2H), 2.28 (s, 3H), 1.48 (s, 9H). **^{19}F NMR** (376 MHz, DMSO-d_6) δ -62.61 (s, 2F). **^{13}C NMR** (101 MHz, DMSO-d_6) δ 152.60, 148.73, 147.70, 142.14, 135.08, 130.09, 126.19(t, J = 3.7 Hz), 126.01(t, J = 32.3 Hz), 122.34(t, J = 260.6 Hz), 121.56, 117.45, 79.46, 28.03, 20.28. **HRMS** (EI): m/z [$(M)^{\cdot+}$] calcd for $C_{19}H_{21}F_2NO_3$, 349.1489. found, 349.1492.

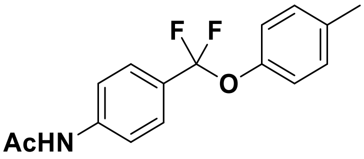

N-(4-(difluoro(p-tolyloxy)methyl)phenyl)acetamide (3h).

Following the general procedure, dry acetone (2.5 ml) and DMF (0.5 ml) were used for solvent and 100 mg 4 Å MS was added. Preparative TLC (petroleum ether/ethyl acetate = 20/1) to afford **3h** as a white solid (46.4 mg, 80% yield). **^{1}H NMR** (400 MHz, CD_3CN) δ 8.59 (s, 1H), 7.69 (d, J = 8.9, 2H), 7.65 (d, J = 8.9, 2H), 7.19 (d, J = 8.6, 2H), 7.14 (d, J = 8.6, 2H), 2.32 (s, 3H), 2.08 (s, 3H). **^{19}F NMR** (376 MHz, CD_3CN) δ -64.48 (s, 2F). **^{13}C NMR** (101 MHz, CD_3CN) δ 169.95, 149.13, 142.59, 136.68, 130.92, 128.94(t, J = 32.8 Hz), 127.29(t, J = 3.9 Hz), 123.47(t, J = 260.6 Hz), 122.73, 119.67, 24.39, 20.73. HRMS (APCI): m/z [$(M+H)^+$] calcd for $C_{16}H_{16}F_2NO_2$, 292.1144. found, 292.1148.

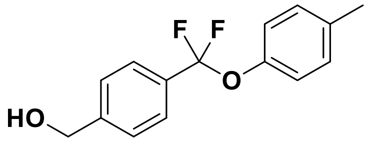

(4-(difluoro(p-tolyloxy)methyl)phenyl)methanol (3i).

Following the general procedure, dry acetone (2.5 ml) and DMF (0.5 ml) were used for solvent. Preparative TLC (petroleum ether/ethyl acetate = 10/1) to afford **3i** as a colorless oil-like liquid (39.7 mg, 75% yield). **^{1}H NMR** (400 MHz, $CDCl_3$) δ 7.71 (d, J = 8.2, 2H), 7.43 (d, J = 8.0, 2H), 7.17-7.12 (m, 4H), 4.71 (s, 2H), 2.34 (s, 3H), 2.08 (s, 1H). **^{19}F NMR** (376 MHz, $CDCl_3$) δ -65.07 (s, 2F). **^{13}C NMR** (101 MHz, $CDCl_3$) δ 148.17, 143.62, 135.22, 133.45, 133.12(t, J = 32.3 Hz), 132.81, 129.78, 126.63, 125.82(t, J = 3.8 Hz), 122.12(t, J = 262.1 Hz), 121.77, 64.57, 20.73. **HRMS** (EI): m/z [$(M)^{\cdot+}$] calcd for $C_{15}H_{14}F_2O_2$, 264.0962. found, 264.9067.

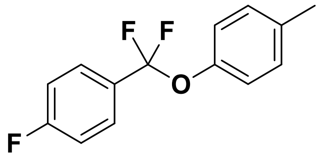

1-(difluoro(4-fluorophenyl)methoxy)-4-methylbenzene (3j).

Following the general procedure, dry acetone (2.5 ml) and DMF (0.5 ml) were used for solvent. Preparative TLC (petroleum ether/ethyl acetate = 200/1) to afford **3j** as a colorless oil-like liquid (41.7 mg, 83% yield). **^{1}H NMR** (400 MHz, $CDCl_3$) δ 7.77-7.71 (m, 2H), 7.19-7.13 (m, 6H), 2.36 (s, 3H). **^{19}F NMR** (376 MHz, $CDCl_3$) δ -69.71 (s, 2F),

-114.81 (s, 1F). **^{13}C NMR** (101 MHz, $CDCl_3$) δ 164.03(d, J = 251.5 Hz), 148.08, 135.39, 129.88, 129.73(td, J = 31.4, 1.3 Hz), 127.90(dt, J = 9.3, 4.1 Hz), 121.81(t, J = 261.6 Hz), 121.80, 115.47(d, J = 22.2 Hz), 20.77. **HRMS** (EI): m/z [(M)$^{\cdot+}$] calcd for $C_{14}H_{11}F_3O$, 252.0762. found, 252.0767.

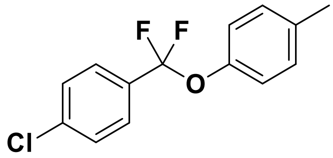

1-chloro-4-(difluoro(p-tolyloxy)methyl)benzene (3k).

Following the general procedure, dry acetone (2.5 ml) and DMF (0.5 ml) were used for solvent. Preparative TLC (petroleum ether/ethyl acetate = 100/1) to afford **3k** as a white solid (37.4 mg, 70% yield). **^{1}H NMR** (400 MHz, $CDCl_3$) δ 7.68 (d, J = 8.8, 2H), 7.45 (d, J = 8.8, 2H), 7.20-7.12 (m, 4H), 2.36 (s, 3H).**^{19}F NMR** (376 MHz, $CDCl_3$) δ -65.12 (s, 2F). **^{13}C NMR** (101 MHz, $CDCl_3$) δ 148.02, 136.95, 135.43, 132.45(t, J = 32.8 Hz), 129.89, 128.70, 127.14(t, J = 3.9 Hz), 121.79, 121.75(t, J = 262.6 Hz), 20.77. **HRMS** (EI): m/z [(M)$^{\cdot+}$] calcd for $C_{14}H_{11}{}^{35}ClF_2O$, 268.0466. found, 268.0467.

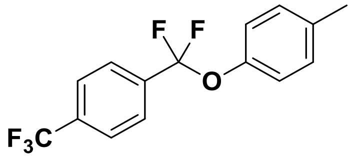

1-(difluoro(4-(trifluoromethyl)phenyl)methoxy)-4-methylbenzene (3l).

Following the general procedure, dry acetone (2.5 ml) and DMF (0.5 ml) were used for solvent. Preparative TLC (petroleum ether/ethyl acetate = 200/1) to afford **3l** as a white solid (32.0 mg, 53% yield). **^{1}H NMR** (400 MHz, $CDCl_3$) δ 7.88 (d, J = 8.2, 2H), 7.75 (d, J = 8.2, 2H), 7.21-7.13 (m, 4H), 2.36 (s, 3H).**^{19}F NMR** (376 MHz, $CDCl_3$) δ -63.01 (s, 3F), -65.75 (s, 2F). **^{13}C NMR** (101 MHz, $CDCl_3$) δ 147.89, 137.40(t, J = 33.3 Hz), 135.65, 132.89(q, J = 33.0 Hz), 129.97, 126.25(t, J = 4.0 Hz), 125.55(q, J = 4.0 Hz), 123.65(q, J = 273.7 Hz), 121.80, 121.38(t, J = 262.6 Hz), 20.78. HRMS (EI): m/z [(M)$^{\cdot+}$] calcd for $C_{15}H_{11}F_5O$, 302.0730. found, 302.0730.

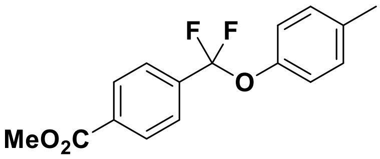

methyl 4-(difluoro(p-tolyloxy)methyl)benzoate (3m).

Following the general procedure, dry acetone (1.5 ml) and DMF (1.5 ml) were used for solvent and 100 mg 4 Å MS was added. Preparative TLC (petroleum ether/ethyl acetate = 20/1) to afford **3m** as a white solid (42.0 mg, 72% yield). **^{1}H NMR** (400 MHz, $CDCl_3$) δ 8.14 (d, J = 8.7, 2H), 7.81 (d, J = 8.6, 2H), 7.21-7.12 (m, 4H), 3.95 (s, 3H), 2.35 (s, 3H). **^{19}F NMR** (376 MHz, $CDCl_3$) δ -65.73 (s, 2F). **^{13}C NMR** (101 MHz, $CDCl_3$) δ 166.22, 147.90, 138.19, 137.61(t, J = 30.4 Hz), 132.29, 129.90, 129.69, 125.77(t, J = 4.0 Hz), 121.81, 121.61(t, J = 262.5 Hz), 52.36, 20.77. **HRMS** (EI): m/z [$(M)^{\cdot+}$] calcd for $C_{16}H_{14}F_2O_3$, 292.0911. found, 292.0920.

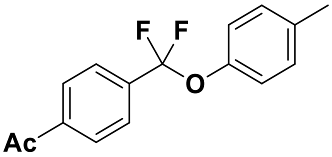

1-(4-(difluoro(p-tolyloxy)methyl)phenyl)ethan-1-one (3n).

Following the general procedure, dry acetone (1.5 ml) and DMF (1.5 ml) were used for solvent and 100 mg 4 Å MS was added. Preparative TLC (petroleum ether/ethyl acetate = 30/1) to afford **3n** as a white solid (39.2 mg, 71% yield). **^{1}H NMR** (400 MHz, $CDCl_3$) δ 8.04 (d, J = 8.2, 2H), 7.83 (d, J = 8.3, 2H), 7.23-7.08 (m, 4H), 2.64 (s, 3H), 2.35 (s, 3H). **^{19}F NMR** (376 MHz, $CDCl_3$) δ -65.68 (s, 2F). **^{13}C NMR** (101 MHz, $CDCl_3$) δ 197.24, 147.85, 138.81, 137.91(t, J = 32.8 Hz), 135.50, 129.90, 128.34, 125.99(t, J = 3.7 Hz), 121.75, 121.57(t, J = 263.1 Hz), 26.70, 20.74. **HRMS** (EI): m/z [$(M)^{\cdot+}$] calcd for $C_{16}H_{14}F_2O_2$, 276.0962. found, 276.0955.

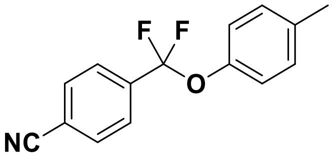

4-(difluoro(p-tolyloxy)methyl)benzonitrile (3o).

Following the general procedure, dry acetone (1.5 ml) and DMF (1.5 ml) were used for solvent. Preparative TLC (petroleum ether/ethyl acetate = 20/1) to afford **3o** as a white solid (32.4mg, 55% yield). **^{1}H NMR** (400 MHz, $CDCl_3$) δ 7.85 (d, J = 8.3, 2H), 7.77 (d, J = 8.3, 2H), 7.18 (d, J = 8.7, 2H), 7.14 (d, J = 8.8, 2H), 2.36 (s, 3H). **^{19}F NMR** (376 MHz, $CDCl_3$) δ -65.96 (s, 2F). **^{13}C NMR** (101 MHz, $CDCl_3$) δ 147.69, 138.09(t, J =33.3 Hz), 135.78, 132.34, 129.99, 126.52(t, J = 3.5 Hz), 121.74, 121.04(t, J = 263.1 Hz), 117.87, 114.86, 20.63. **HRMS** (EI): m/z [$(M)^{\cdot+}$] calcd for $C_{15}H_{11}F_2NO$, 259.0809. found, 259.0810.

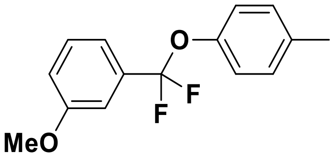

1-(difluoro(p-tolyloxy)methyl)-3-methoxybenzene (3p).

Following the general procedure, dry acetone (2.5 ml) and DMF (0.5 ml) were used for solvent and 100 mg 4 Å MS was added. Preparative TLC (petroleum ether/ethyl acetate = 100/1) to afford **3p** as a colorless oil-like liquid (43.3mg, 82% yield). **^{1}H NMR** (400 MHz, $CDCl_3$) δ 7.40 (t, J = 7.9, 1H), 7.35 (d, J = 7.8, 1H), 7.29 (s, 1H), 7.21-7.15 (m, 4H), 7.05 (d, J = 8.0, 1H), 3.87 (s, 3H), 2.37 (s, 3H). **^{19}F NMR** (376 MHz, $CDCl_3$) δ -65.30 (s, 2F). **^{13}C NMR** (101 MHz, $CDCl_3$) δ 159.54, 148.20, 135.23(t, J = 31.8 Hz), 129.83, 129.57, 121.98(t, J = 262.3 Hz), 121.81, 117.83(t, J = 3.5 Hz), 116.59, 111.05(t, J = 3.5 Hz), 55.36, 20.75. **HRMS** (EI): m/z [$(M)^{\cdot+}$] calcd for $C_{15}H_{14}F_2O_2$, 264.0962. found, 264.0971.

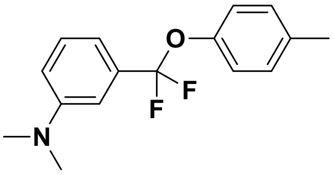

3-(difluoro(p-tolyloxy)methyl)-N,N-dimethylaniline (3q).

Following the general procedure, dry acetone (2.5 ml) and DMF (0.5 ml) were used for solvent and 100 mg 4 Å MS was added. Preparative TLC (petroleum ether/ethyl acetate = 30/1) to afford **3q** as a white solid (49.9 mg, 90% yield). **^{1}H NMR** (400 MHz, DMSO-

d_6) δ 7.32 (t, J = 7.9, 1H), 7.21 (d, J = 8.6, 2H), 7.17 (d, J = 8.7, 2H), 6.97 (t, J = 5.4, 2H), 6.89 (dd, J = 8.3, 2.4, 1H), 2.94 (s, 6H), 2.29 (s, 3H). **^{19}F NMR** (376 MHz, DMSO-d_6) δ -63.68 (s, 2F). **^{13}C NMR** (101 MHz, DMSO-d_6) δ 150.28, 147.78, 135.06, 133.52(t, J = 30.8Hz), 129.99, 129.32, 122.47(t, J = 261.1 Hz), 121.61, 114.66, 112.61(t, J = 3.5 Hz), 108.41(t, J = 3.5 Hz), 39.93, 20.27. **HRMS** (APCI): m/z [(M+H)$^+$] calcd for $C_{16}H_{18}F_2NO$, 278.1351. found, 278.1359.

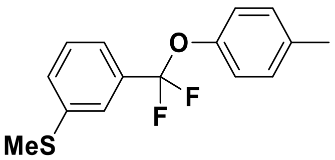

(3-(difluoro(p-tolyloxy)methyl)phenyl)(methyl)sulfane (3r).

Following the general procedure, dry acetone (2.5 ml) and DMF (0.5 ml) were used for solvent and 100 mg 4 Å MS was added. Preparative TLC (petroleum ether/ethyl acetate = 100/1) to afford **3r** as a white solid (49.9 mg, 88% yield). **^{1}H NMR** (400 MHz, DMSO-d_6) δ 7.56 (s, 1H), 7.51-7.46 (m, 3H), 7.24-7.17 (m, 4H), 2.53 (s, 3H), 2.29 (s, 3H). **^{19}F NMR** (376 MHz, DMSO-d_6) δ -63.96 (s, 2F). **^{13}C NMR** (101 MHz, DMSO-d_6) δ 147.95, 139.96, 135.77, 133.95(t, J = 32.3Hz), 130.49, 129.83, 128.89, 122.74(t, J = 3.5 Hz), 122.22(t, J = 3.5 Hz), 122.19(t, J = 261.6 Hz), 122.13, 20.73, 14.92. **HRMS** (EI): m/z [(M)$^{\cdot+}$] calcd for $C_{15}H_{14}F_2O^{32}S$, 280.0733. found, 280.0722.

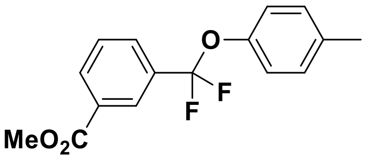

methyl 3-(difluoro(p-tolyloxy)methyl)benzoate (3s).

Following the general procedure, dry acetone (2.5 ml) and DMF (0.5 ml) were used for solvent and 100 mg 4 Å MS was added. Preparative TLC (petroleum ether/ethyl acetate = 20/1) to afford **3s** as a white solid (53.7mg, 92% yield). **^{1}H NMR** (400 MHz, $CDCl_3$) δ 8.44 (s, 1H), 8.19 (d, J = 7.8, 1H), 7.93 (d, J = 7.8, 1H), 7.56 (t, J = 7.8, 1H), 7.21-7.14 (m, 4H), 3.96 (s, 3H), 2.35 (s, 3H). **^{19}F NMR** (376 MHz, $CDCl_3$) δ -65.38 (s, 2F). **^{13}C NMR** (101 MHz, $CDCl_3$) δ 166.17, 147.97, 135.46, 134.41(t, J = 32.8 Hz), 131.81,

130.58, 129.94(t, J = 3.1 Hz), 129.88, 128.66, 126.95(t, J = 4.0 Hz), 121.85, 121.63(t, J = 262.1 Hz), 52.33, 20.75. **HRMS** (EI): m/z [(M)$^{\cdot+}$] calcd for $C_{16}H_{14}F_2O_3$, 292.0911. found, 292.0913.

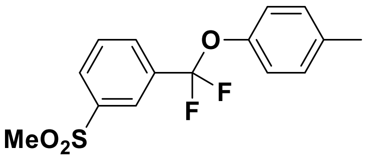

1-(difluoro(p-tolyloxy)methyl)-3-(methylsulfonyl)benzene (3t).

Following the general procedure, dry acetone (2.5 ml) and DMF (0.5 ml) were used for solvent and 100 mg 4 Å MS was added. Preparative TLC (petroleum ether/ethyl acetate = 30/1) to afford **3t** as a colorless oil-like liquid (48.6mg, 78% yield). **^{1}H NMR** (400 MHz, $CDCl_3$) δ 8.32 (s, 1H), 8.08 (d, J = 7.8, 1H), 8.01 (d, J = 7.8, 1H), 7.69 (t, J = 7.8, 1H), 7.18-7.13 (m, 4H), 3.08 (s, 3H), 2.34 (s, 3H). **^{19}F NMR** (376 MHz, $CDCl_3$) δ -65.29 (s, 2F). **^{13}C NMR** (101 MHz, $CDCl_3$) δ 147.72(t, J = 1.5 Hz), 141.25, 135.86, 135.65(t, J = 33.8 Hz), 130.95(t, J = 3.5 Hz), 130.13, 130.04, 129.88, 129.72, 125.01(t, J = 4.0 Hz), 121.91, 121.07(t, J = 263.1 Hz), 44.49, 20.83. **HRMS** (EI): m/z [(M)$^{\cdot+}$] calcd for $C_{15}H_{14}F_2O_3{}^{32}S$, 312.0632. found, 312.0624.

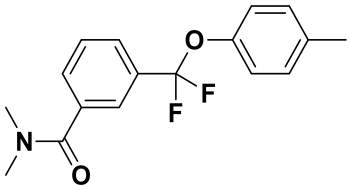

3-(difluoro(p-tolyloxy)methyl)-N,N-dimethylbenzamide (3u).

Following the general procedure, dry acetone (2.5 ml) and DMF (0.5 ml) were used for solvent and 100 mg 4 Å MS was added. Preparative TLC (petroleum ether/ethyl acetate = 100/1) to afford **3u** as a white solid (52.5mg, 86% yield). **^{1}H NMR** (400 MHz, $CDCl_3$) δ 7.81-7.73 (m, 2H), 7.52 (m, 3H), 7.19 – 7.07 (m, 4H), 3.11 (s, 3H), 2.95 (s, 3H), 2.32 (s, 3H). **^{19}F NMR** (376 MHz, $CDCl_3$) δ -65.30 (s, 2F). **^{13}C NMR** (101 MHz, $CDCl_3$) δ 170.42, 147.94, 136.57, 135.32, 134.11(t, J = 32.8 Hz), 129.79, 129.30, 128.55, 126.63(t, J = 3.5 Hz), 124.39(t, J = 3.5 Hz), 121.73, 121.64(t, J = 262.6 Hz), 39.39,

35.27, 20.67. **HRMS** (APCI): m/z [(M+H)$^+$] calcd for $C_{17}H_{18}F_2NO_2$, 306.1300. found, 306.1307.

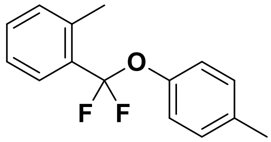

1-(difluoro(p-tolyloxy)methyl)-2-methylbenzene (3v).

Following the general procedure, preparative TLC (petroleum ether/ethyl acetate = 200/1) to afford **3v** as a colorless oil-like liquid (37.8mg, 76% yield). **^{1}H NMR** (500 MHz, $CDCl_3$) δ 7.74 (d, J = 7.8, 1H), 7.39 (t, J = 7.2, 1H), 7.28 (d, J = 10.1, 2H), 7.22-7.14 (m, 4H), 2.61 (s, 3H), 2.36 (s, 3H). **^{19}F NMR** (471 MHz, $CDCl_3$) δ -66.02 (s, 2F). **^{13}C NMR** (126 MHz, $CDCl_3$) δ 148.25, 136.65, 135.17, 131.97(t, J = 30.2 Hz), 131.75, 130.66, 129.83, 125.99(t, J = 5.7 Hz), 125.51, 122.55(t, J = 263.3 Hz), 121.73, 20.78, 19.81(t, J = 1.9 Hz). **HRMS** (EI): m/z [(M)$^{\cdot+}$] calcd for $C_{15}H_{14}F_2O$, 248.1013. found, 248.1006.

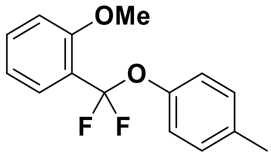

1-(difluoro(p-tolyloxy)methyl)-2-methoxybenzene (3w).

Following the general procedure, dry acetone (2.5 ml) and DMF (0.5 ml) were used for solvent and 100 mg 4 Å MS was added. Preparative TLC (petroleum ether/ethyl acetate = 100/1) to afford **3w** as a colorless oil-like liquid (34.8mg, 66% yield). **^{1}H NMR** (400 MHz, $CDCl_3$) δ 7.67 (d, J = 7.7, 1H), 7.43 (t, J = 7.9, 1H), 7.13 (m, 4H), 6.98 (m, 2H), 3.91 (s, 3H), 2.32 (s, 3H). **^{19}F NMR** (376 MHz, $CDCl_3$) δ -65.81 (s, 2F). **^{13}C NMR** (101 MHz, $CDCl_3$) δ 157.43, 148.41, 134.95, 132.27, 129.84(t, J = 32.0 Hz), 129.67, 127.56(t, J = 5.5 Hz), 121.91, 121.75(t, J = 260.6 Hz), 119.89, 112.06, 55.92, 20.76. **HRMS** (EI): m/z [(M)$^{\cdot+}$] calcd for $C_{15}H_{14}F_2O_2$, 264.0962. found, 264.0967.

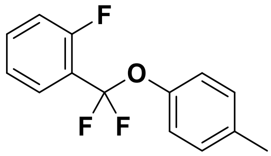

1-(difluoro(p-tolyloxy)methyl)-2-fluorobenzene (3x).

Following the general procedure, dry acetone (2.5 ml) and DMF (0.5 ml) were used for solvent and 100 mg 4 Å MS was added. Preparative TLC (petroleum ether/ethyl acetate = 200/1) to afford **3x** as a colorless oil-like liquid (36.8mg, 73% yield). **^{1}H NMR** (400 MHz, $CDCl_3$) δ 7.72 (td, J = 7.6, 1.5, 1H), 7.53-7.45 (m, 1H), 7.24-7.14 (m, 6H), 2.34 (s, 3H). **^{19}F NMR** (376 MHz, $CDCl_3$) δ -64.97(d, J = 12.3Hz, 2F), -114.08(t, J = 13.2Hz, 3F). **^{13}C NMR** (101 MHz, $CDCl_3$) δ 159.84(dt, J = 255.5, 2.1 Hz), 148.02(t, J = 2.0 Hz), 135.45, 132.79(d, J = 8.1 Hz), 129.86, 127.65(td, J = 4.5, 2.0 Hz), 123.72(d, J = 4.0 Hz), 121.94, 120.73(td, J = 263.1, 2.0 Hz), 116.70(d, J = 21.2 Hz), 20.79. **HRMS** (EI): m/z [$(M)^{\cdot+}$] calcd for $C_{14}H_{11}F_3O$, 252.0762. found, 252.0766.

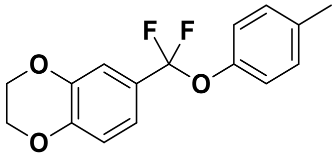

6-(difluoro(p-tolyloxy)methyl)-2,3-dihydrobenzo[b][1,4]dioxine (3y).

Following the general procedure, dry acetone (2.5 ml) and DMF (0.5 ml) were used for solvent and 100 mg 4 Å MS was added. Preparative TLC (petroleum ether/ethyl acetate = 50/1) to afford **3y** as a white solid (46.1mg, 79% yield). **^{1}H NMR** (400 MHz, $CDCl_3$) δ 7.28 (d, J = 2.1, 1H), 7.23 (dd, J = 8.4, 2.1, 1H), 7.20-7.11 (m, 4H), 6.94 (d, J = 8.4, 1H), 4.30-4.27 (m, 4H), 2.35 (s, 3H). **^{19}F NMR** (376 MHz, $CDCl_3$) δ -64.27 (s, 2F). **^{13}C NMR** (101 MHz, $CDCl_3$) δ 148.28(t, J = 1.5 Hz), 145.51(t, J = 2.0 Hz), 143.25, 135.10, 129.80, 127.14(t, J = 32.3 Hz), 122.00(t, J = 262.1 Hz), 121.73, 118.88(t, J = 4.1 Hz), 117.22, 115.19(t, J = 4.1 Hz), 64.41, 64.22, 20.75. **HRMS** (EI): m/z [$(M)^{\cdot+}$] calcd for $C_{16}H_{14}F_2O_3$, 292.0911. found, 292.0920.

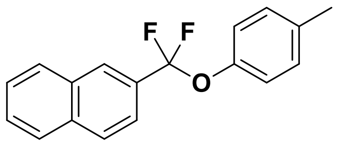

2-(difluoro(p-tolyloxy)methyl)naphthalene (3z).

Following the general procedure, 100 mg 4 Å MS was added. Preparative TLC (petroleum ether/ethyl acetate = 200/1) to afford **3z** as a white solid (39.2mg, 69% yield). **^{1}H NMR** (400 MHz, $CDCl_3$) δ 8.27 (s, 1H), 7.95 (d, J = 8.8, 2H), 7.90 (d, J = 6.9, 1H), 7.80 (dd, J = 8.6, 1.6, 1H), 7.62-7.54 (m, 2H), 7.23 (d, J = 8.6, 2H), 7.18 (d, J = 8.5, 2H), 2.36 (s, 3H). **^{19}F NMR** (376 MHz, $CDCl_3$) δ -64.88 (s, 2F). **^{13}C NMR** (101 MHz, $CDCl_3$) δ 148.29, 135.25, 134.19(t, J = 3.5 Hz), 132.37, 131.11(t, J = 32.8 Hz), 129.86, 128.77, 128.49, 127.74, 127.45, 126.71, 125.68(t, J = 4.5 Hz), 122.41(t, J = 3.5 Hz), 122.37(t, J = 262.1 Hz), 121.85, 20.77. **HRMS** (EI): m/z [$(M)^{\cdot+}$] calcd for $C_{18}H_{14}F_2O$, 284.1013. found, 274.1007.

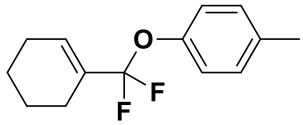

1-(cyclohex-1-en-1-yldifluoromethoxy)-4-methylbenzene (3aa).

Following the general procedure, 100 mg 4 Å MS was added. Preparative TLC (petroleum ether/ethyl acetate = 200/1) to afford **3aa** as a colorless oil-like liquid (37.1mg, 78% yield). **^{1}H NMR** (400 MHz, $CDCl_3$) δ 7.15 (d, J = 8.5, 2H), 7.10 (d, J = 8.5, 2H), 6.43-6.35 (m, 1H), 2.35 (s, 3H), 2.25 (m, 2H), 2.17-2.12 (m, 2H), 1.73 (m, 2H), 1.66 -1.60 (m, 2H). **^{19}F NMR** (376 MHz, $CDCl_3$) δ -72.69 (s, 2F). **^{13}C NMR** (101 MHz, $CDCl_3$) δ 148.32, 134.84, 130.84(t, J = 30.3 Hz), 129.69, 128.75(t, J = 5.5 Hz), 122.15(t, J = 262.1 Hz), 121.68, 24.44, 22.59(t, J = 1.5 Hz), 21.81, 21.54, 20.73. **HRMS** (EI): m/z [$(M)^{\cdot+}$] calcd for $C_{14}H_{16}F_2O$, 238.1169. found, 238.1166.

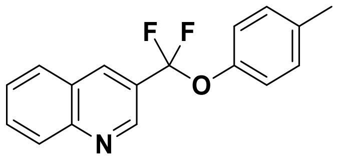

3-(difluoro(p-tolyloxy)methyl)quinoline (3ab).

Following the general procedure, dry acetone (2.5 ml) and DMF (0.5 ml) were used for solvent and 100 mg 4 Å MS was added. Preparative TLC (petroleum ether/ethyl acetate = 10/1) to afford **3ab** as a white solid (32.5mg, 57% yield). **^{1}H NMR** (400 MHz, $CDCl_3$) δ 9.21 (d, J = 2.1, 1H), 8.51 (s, 1H), 8.17 (d, J = 8.5, 1H), 7.90 (d, J = 8.2, 1H), 7.83-7.78 (m, 1H), 7.62 (t, J = 7.5, 1H), 7.19 (d, J = 8.9, 2H), 7.16 (d, J = 8.8, 2H), 2.34 (s, 3H). **^{19}F NMR** (376 MHz, $CDCl_3$) δ -64.38 (s, 2F). **^{13}C NMR** (101 MHz, $CDCl_3$) δ 148.84, 147.88, 147.14(t, J = 3.0 Hz), 135.67, 133.87(t, J = 3.0 Hz), 131.08, 129.98, 129.42, 128.54, 127.52, 126.90(t, J = 31.3 Hz), 126.57, 121.84(t, J = 262.1 Hz), 121.55, 20.77. **HRMS** (APCI): m/z [$(M+H)^+$] calcd for $C_{17}H_{14}F_2NO$, 286.1038. found, 286.1043.

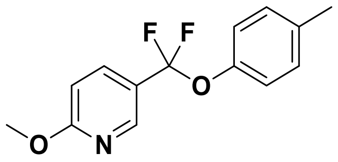

5-(difluoro(p-tolyloxy)methyl)-2-methoxypyridine (3ac).

Following the general procedure, dry acetone (2.5 ml) and DMF (0.5 ml) were used for solvent and 100 mg 4 Å MS was added. Preparative TLC (petroleum ether/ethyl acetate = 5/1) to afford **3ac** as a white solid (39.8 mg, 75% yield). **^{1}H NMR** (400 MHz, DMSO-d_6) δ 8.56 (d, J = 1.8, 1H), 8.03 (dd, J = 8.7, 2.6, 1H), 7.22 (d, J = 8.5, 2H), 7.18 (d, J = 8.7, 2H), 6.96 (d, J = 8.7, 1H), 3.91 (s, 3H), 2.29 (s, 3H). **^{19}F NMR** (376 MHz, DMSO-d_6) δ -62.49 (s, 2F). **^{13}C NMR** (101 MHz, DMSO-d_6) δ 165.20, 147.46(t, J = 2.0 Hz), 144.92(t, J = 4.5 Hz), 136.69(t, J = 3.0 Hz), 135.43, 130.10, 122.39(t, J = 32.8 Hz), 121.93(t, J = 259.6 Hz), 121.70, 110.84, 53.71, 20.28. **HRMS** (APCI): m/z [$(M+H)^+$] calcd for $C_{14}H_{14}F_2NO_2$, 266.0987. found, 266.0993.

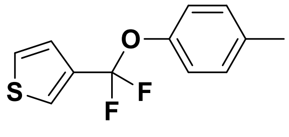

3-(difluoro(p-tolyloxy)methyl)thiophene (3ad).

Following the general procedure, dry acetone (2.5 ml) and DMF (0.5 ml) were used for solvent and 100 mg 4 Å MS was added. Preparative TLC (petroleum ether/ethyl acetate = 60/1) to afford **3ad** as a white solid (30.7 mg, 64% yield). **^{1}H NMR** (400 MHz, DMSO-d_6) δ 8.15-8.04 (m, 1H), 7.74-7.70 (m, 1H), 7.37 (d, J = 5.1, 1H), 7.21 (d, J = 8.5, 2H), 7.15 (d, J = 8.4, 2H), 2.29 (s, 3H). **^{19}F NMR** (376 MHz, DMSO-d_6) δ -59.59 (s, 2F). **^{13}C NMR** (101 MHz, DMSO-d_6) δ 147.54, 135.25, 134.02(t, J = 34.8 Hz), 130.03, 128.32, 127.17(t, J = 4.5 Hz), 124.89(t, J = 2.1 Hz), 121.60, 120.35(t, J = 260.6 Hz), 20.28. **HRMS** (EI): m/z [(M)$^{\cdot+}$] calcd for $C_{12}H_{10}F_2O^{32}S$, 240.0420. found, 240.0426.

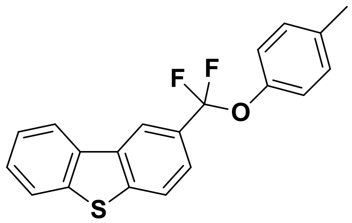

2-(difluoro(p-tolyloxy)methyl)dibenzo[b,d]thiophene (3ae).

Following the general procedure, dry acetone (2.5 ml) and DMF (0.5 ml) were used for solvent and 100 mg 4 Å MS was added. Preparative TLC (petroleum ether/ethyl acetate = 40/1) to afford **3ae** as a white solid (30.7 mg, 64% yield). **^{1}H NMR** (400 MHz, DMSO-d_6) δ 8.78 (s, 1H), 8.60-8.53 (m, 1H), 8.21 (d, J = 8.4, 1H), 8.14-8.00 (m, 1H), 7.85 (dd, J = 8.4, 1.6, 1H), 7.62-7.51 (m, 2H), 7.26 (d, J = 8.7, 2H), 7.22 (d, J = 8.7, 2H), 2.28 (s, 3H). **^{19}F NMR** (376 MHz, DMSO-d_6) δ -62.29 (s, 2F). **^{13}C NMR** (101 MHz, DMSO-d_6) δ 147.72, 141.48, 139.12, 135.29, 135.05, 134.48, 130.08, 129.58(t, J = 32.3 Hz), 127.72, 125.04, 123.76(t, J = 3.0 Hz), 123.57, 123.13, 122.64, 122.50(t, J = 260.1 Hz), 121.75, 119.34(t, J = 4.0 Hz), 20.28. **HRMS** (EI): m/z [(M)$^{\cdot+}$] calcd for $C_{20}H_{14}F_2O^{32}S$, 340.0733. found, 340.0729.

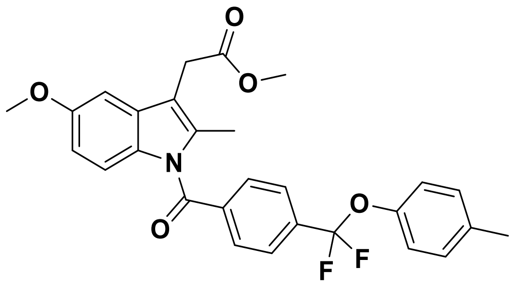

methyl 2-(1-(4-(difluoro(p-tolyloxy)methyl)benzoyl)-5-methoxy-2-methyl-1H-indol-3-yl)acetate (3af).

Following the general procedure, dry acetone (2.5 ml) and DMF (0.5 ml) were used for solvent and 100 mg 4 Å MS was added. Preparative TLC (petroleum ether/ethyl acetate = 4/1) to afford **3af** as a white solid (61.1mg, 62% yield). **^{1}H NMR** (400 MHz, DMSO-d_6) δ 7.93 (d, J = 8.2, 2H), 7.81 (d, J = 8.1, 2H), 7.23 (m, 4H), 7.05 (d, J = 2.1, 1H), 6.93 (d, J = 9.0, 1H), 6.71 (dd, J = 9.0, 2.2, 1H), 3.79 (s, 2H), 3.77 (s, 3H), 3.63 (s, 3H), 2.31 (s, 3H), 2.18 (s, 3H). **^{19}F NMR** (376 MHz, DMSO-d_6) δ -64.11 (s, 2F). **^{13}C NMR** (101 MHz, DMSO-d_6) δ 170.99, 167.99, 155.71, 147.40, 138.18, 136.07(t, J = 32.3 Hz), 135.59, 135.36, 130.68, 130.15, 129.58, 126.18(t, J = 3.0 Hz), 121.83, 121.65(t, J = 261.1 Hz), 114.73, 113.03, 111.41, 101.72, 55.41, 51.80, 29.11, 20.32, 13.28. **HRMS** (APCI): m/z [(M+H)$^+$] calcd for $C_{28}H_{26}F_2NO_5$, 494.1774. found, 494.1782.

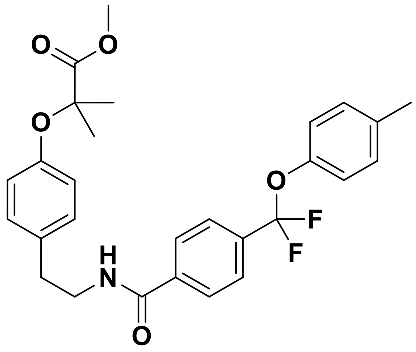

methyl 2-(4-(2-(4-(difluoro(p-tolyloxy)methyl)benzamido)ethyl)phenoxy)-2-methylpropanoate (3ag).

Following the general procedure, dry acetone (2.5 ml) and DMF (0.5 ml) were used for solvent and 100 mg 4 Å MS was added. Preparative TLC (petroleum ether/ethyl acetate = 3/1) to afford **3ag** as a white solid (56.6mg, 57% yield). **^{1}H NMR** (400 MHz, DMSO-d_6) δ 8.77 (t, J = 5.5, 1H), 7.99 (d, J = 8.3, 2H), 7.84 (d, J = 8.4, 2H), 7.26 – 7.13 (m,

6H), 6.73 (d, J = 8.5, 2H), 3.69 (s, 3H), 3.48 (m, 2H), 2.80 (t, J = 7.4, 2H), 2.30 (s, 3H), 1.50 (s, 6H). **^{19}F NMR** (376 MHz, DMSO-d$_6$) δ -63.85 (s, 2F). **^{13}C NMR** (101 MHz, DMSO-d$_6$) δ 173.81, 165.21, 153.32, 147.47, 137.26, 135.43, 134.91(t, J = 32.3 Hz), 133.07, 130.11, 129.49, 127.67, 125.55(t, J = 3.5 Hz), 121.82(t, J = 261.1 Hz), 121.71, 118.90, 78.57, 52.24, 40.99, 34.14, 24.97, 20.32. **HRMS** (APCI): m/z [(M+H)$^+$] calcd for $C_{28}H_{30}F_2NO_5$, 498.2087. found, 498.2104.

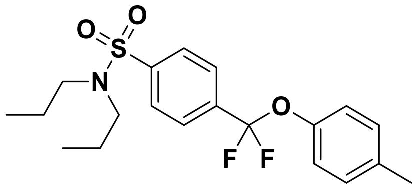

4-(difluoro(p-tolyloxy)methyl)-N,N-dipropylbenzenesulfonamide (3ah).

Following the general procedure, dry acetone (2.5 ml) and DMF (0.5 ml) were used for solvent and 100 mg 4 Å MS was added. Preparative TLC (petroleum ether/ethyl acetate = 4/1) to afford **3ah** as a white solid (39.6mg, 50% yield). **^{1}H NMR** (400 MHz, DMSO-d$_6$) δ 8.04 -7.94 (m, 4H), 7.25 (d, J = 8.6, 2H), 7.20 (d, J = 8.6, 2H), 3.09-3.01 (m, 4H), 2.31 (s, 3H), 1.49 (m, 4H), 0.81 (t, J = 7.4, 6H). **^{19}F NMR** (376 MHz, DMSO-d$_6$) δ -64.29 (s, 2F). **^{13}C NMR** (101 MHz, DMSO-d$_6$) δ 147.27, 142.29, 136.25(t, J = 32.8 Hz), 135.65, 130.15, 127.41, 126.72(t, J = 3.5 Hz), 121.78, 121.34(t, J = 261.6 Hz), 49.59, 21.56, 20.30, 10.91. **HRMS** (APCI): m/z [(M+H)$^+$] calcd for $C_{20}H_{26}F_2NO_3{}^{32}S$, 398.1596. found, 398.1603.

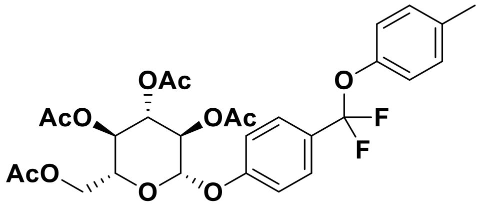

(2R,3R,4S,5R,6S)-2-(acetoxymethyl)-6-(4-(difluoro(p-tolyloxy)methyl)phenoxy)tetrahydro-2H-pyran-3,4,5-triyl triacetate (3ai).

Following the general procedure, dry acetone (2.5 ml) and DMF (0.5 ml) were used for solvent and 100 mg 4 Å MS was added. Preparative TLC (petroleum ether/ethyl acetate

= 1/1) to afford **3ai** as a white solid (49.9mg, 43% yield). **^{1}H NMR** (400 MHz, DMSO-d_6) δ 7.71 (d, J = 8.8, 2H), 7.21 (d, J = 8.5, 2H), 7.19-7.08 (m, 4H), 5.69 (d, J = 8.0, 1H), 5.43 (t, J = 9.6, 1H), 5.10 (dd, J = 9.7, 8.0, 1H), 5.03 (t, J = 9.7, 1H), 4.28 (m, 1H), 4.21 (dd, J = 12.2, 5.4, 1H), 4.08 (dd, J = 12.2, 2.0, 1H), 2.29 (s, 3H), 2.08-1.99 (m, 9H), 1.97 (s, 3H). **^{19}F NMR** (376 MHz, DMSO-d_6) δ -62.60 (s, 2F). **^{13}C NMR** (101 MHz, DMSO-d_6) δ 169.98, 169.60, 169.31, 169.10, 158.18, 147.65, 135.24, 130.08, 127.38(t, J = 3.5 Hz), 127.25(t, J = 32.3 Hz), 122.10(t, J = 260.1 Hz), 121.61, 116.38, 96.71, 71.87, 70.95, 70.63, 67.95, 61.56, 20.45, 20.37, 20.30, 20.29, 20.27. **HRMS** (APCI): m/z [(M+Na)$^+$] calcd for $C_{28}H_{30}F_2O_{11}Na$, 603.1649. found, 603.1646.

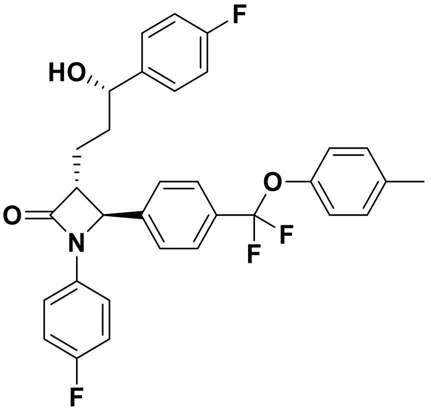

(3R,4S)-4-(4-(difluoro(p-tolyloxy)methyl)phenyl)-1-(4-fluorophenyl)-3-((S)-3-(4-fluorophenyl)-3-hydroxypropyl)azetidin-2-one (3aj).

Following the general procedure, dry acetone (2.5 ml) and DMF (0.5 ml) were used for solvent and 100 mg 4 Å MS was added. Preparative TLC (petroleum ether/ethyl acetate = 1/1) to afford **3ae** as a white solid (71.4mg, 65% yield). **^{1}H NMR** (400 MHz, DMSO-d_6) δ 7.78 (d, J = 8.3, 2H), 7.59 (d, J = 8.2, 2H), 7.33 (dd, J = 8.5, 5.7, 2H), 7.26 – 7.09 (m, 10H), 5.34 (d, J = 4.5, 1H), 5.08 (d, J = 2.1, 1H), 4.58 – 4.49 (m, 1H), 3.17 (dd, J = 8.5, 2.3, 1H), 2.28 (s, 3H), 1.98 – 1.74 (m, 4H). **^{19}F NMR** (376 MHz, DMSO-d_6) δ -63.85 (s, 2F), -116.33 (s, 1F), -118.34 (s, 1F). **^{13}C NMR** (101 MHz, DMSO-d_6) δ 166.90, 161.08(d, J = 242.4 Hz), 158.20(d, J = 241.4 Hz), 147.49, 142.14(d, J = 3.0 Hz), 141.56, 135.37, 133.75(d, J = 2.0 Hz), 132.73(t, J = 31.8 Hz), 127.55(d, J = 8.1 Hz), 126.67, 126.23(t, J = 3.0 Hz), 121.88(t, J = 261.1 Hz), 121.71, 118.26(d, J = 8.1 Hz), 115.98(d,

J = 23.2 Hz), 114.67(d, J = 21.2 Hz), 71.15, 59.71, 59.08, 36.34, 24.59, 20.28. **HRMS** (APCI): m/z [(M-OH)$^+$] calcd for $C_{32}H_{26}F_4NO_2$, 532.1894. found, 532.1913.

4.2 Representative experimental procedure for the synthesis of compounds 7

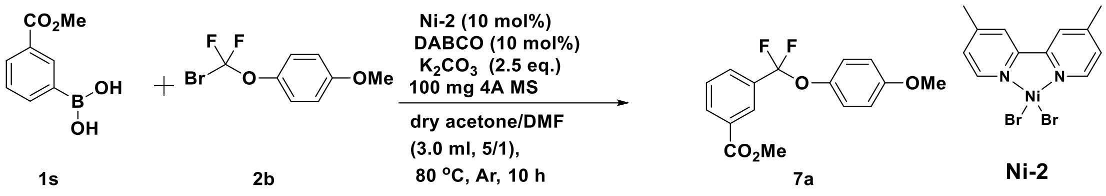

To a dried 10 ml Schlenk-type tube equipped with a magnetic stir bar was charged with arylboronic acid **1s** (72.0 mg, 0.4 mmol, 2.0 equiv), Ni-2 (8.0 mg, 0.02 mmol, 10 mol %), DABCO (2.3 mg, 0.02 mmol, 10 mol %), 100 mg 4 Å MS and K_2CO_3 (69.0 mg, 0.5 mmol, 2.5 equiv) under air. The reaction mixture was then evacuated and backfilled with Ar (3 times). 1-(bromodifluoromethoxy)-4-methoxybenzene **2b** (50.4 mg, 0.2mmol, 1.0 equiv), and acetone (2.5 mL) and DMF (0.5 mL) were added. The mixture was stirred at 80 ^{o}C for 10 h. After cooled to room temperature, the reaction mixture was filtered and the filtrate was concentrated. The residue was purified on a preparative TLC with petroleum ether/ethyl acetate as the eluent to afford the products **7a.**

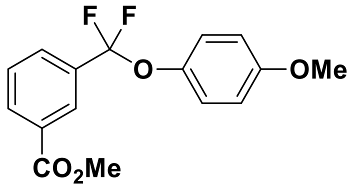

methyl 3-(difluoro(4-methoxyphenoxy)methyl)benzoate (7a).

Following the general procedure, preparative TLC (petroleum ether/ethyl acetate = 25/1) to afford **7a** as a white solid (50.0mg, 81% yield). **^{1}H NMR** (400 MHz, DMSO-d$_6$) δ 8.24 (s, 1H), 8.17 (d, J = 7.8, 1H), 8.03 (d, J = 7.9, 1H), 7.73 (t, J = 7.8, 1H), 7.25 (d, J = 9.0, 2H), 7.01 – 6.95 (m, 2H), 3.89 (s, 3H), 3.75 (s, 3H). **^{19}F NMR** (376 MHz, DMSO-

d_6) δ -64.38 (s, 2F). **^{13}C NMR** (101 MHz, DMSO-d_6) δ 165.77, 157.77, 143.23(t, J = 2.0 Hz), 133.87(t, J = 32.8Hz), 132.40, 130.76, 130.63(t, J = 3.5 Hz), 130.21, 126.24(t, J = 3.5 Hz), 123.82, 121.97(t, J = 260.6 Hz), 115.11, 55.87, 52.94. **HRMS** (EI): m/z [(M)$^{·+}$] calcd for $C_{16}H_{14}F_2O_4$, 308.0860. found, 308.0854.

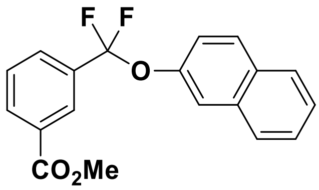

methyl 3-(difluoro(naphthalen-2-yloxy)methyl)benzoate (7b).

Following the general procedure, preparative TLC (petroleum ether/ethyl acetate = 25/1) to afford **7b** as a white solid (46.5mg, 71% yield). **^{1}H NMR** (400 MHz, DMSO-d_6) δ 8.34 (s, 1H), 8.21 (d, J = 7.8, 1H), 8.14 (d, J = 7.8, 1H), 8.03 (d, J = 8.9, 1H), 7.99 (d, J = 7.9, 2H), 7.92 (s, 1H), 7.78 (t, J = 7.8, 1H), 7.56 (m, 3H), 3.93 (s, 3H). **^{19}F NMR** (376 MHz, DMSO-d_6) δ -63.87 (s, 2F). **^{13}C NMR** (101 MHz, DMSO-d_6) δ 165.30, 147.18(t, J = 2.1 Hz), 133.29, 133.25(t, J = 32.8 Hz), 132.08, 131.06, 130.38, 130.23(t, J = 3.5 Hz), 129.86, 129.83, 127.67, 127.65, 126.95, 126.11, 125.82(t, J = 4.1 Hz), 121.77(t, J = 262.1 Hz), 121.42, 118.83, 52.52. **HRMS** (EI): m/z [(M)$^{·+}$] calcd for $C_{19}H_{14}F_2O_3$, 328.0911. found, 328.0903.

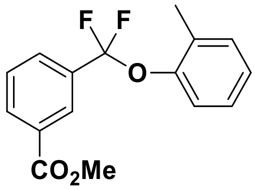

methyl 3-(difluoro(o-tolyloxy)methyl)benzoate (7c).

Following the general procedure, preparative TLC (petroleum ether/ethyl acetate = 25/1) to afford **7c** as a white solid (43.2mg, 74% yield). **^{1}H NMR** (400 MHz, DMSO-d_6) δ 8.28 (s, 1H), 8.18 (d, J = 7.8, 1H), 8.06 (d, J = 7.8, 1H), 7.74 (t, J = 7.8, 1H), 7.32 (d, J = 7.7, 2H), 7.27 (t, J = 7.5, 1H), 7.21 (t, J = 7.3, 1H), 3.89 (s, 3H), 2.27 (s, 3H). **^{19}F NMR** (376 MHz, DMSO-d_6) δ -63.66 (s, 2F). **^{13}C NMR** (101 MHz, DMSO-d_6) δ 165.28, 148.04, 133.49(t, J = 32.8 Hz), 131.99, 131.40, 130.83, 130.36, 130.02(t, J =

3.5 Hz), 129.86, 127.11, 126.25, 125.64(t, J = 4.0 Hz), 122.13, 121.68(t, J = 262.1 Hz), 52.49, 16.11. **HRMS** (EI): m/z [$(M)^{\cdot+}$] calcd for $C_{16}H_{14}F_2O_3$, 292.0911. found, 292.0901

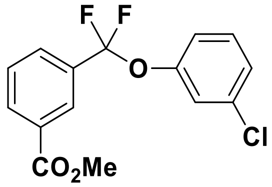

methyl 3-((3-chlorophenoxy)difluoromethyl)benzoate (7d).

Following the general procedure, preparative TLC (petroleum ether/ethyl acetate = 25/1) to afford **7d** as a white solid (43.7mg, 70% yield). **^{1}H NMR** (400 MHz, DMSO-d_6) δ 8.28 (s, 1H), 8.21 (d, J = 7.8, 1H), 8.10 (d, J = 8.0, 1H), 7.77 (t, J = 7.8, 1H), 7.51 (m, 2H), 7.46-7.40 (m, 1H), 7.37 (m, 1H), 3.92 (s, 3H). **^{19}F NMR** (376 MHz, DMSO-d_6) δ -64.40 (s, 2F). **^{13}C NMR** (101 MHz, DMSO-d_6) δ 165.25, 150.14(t, J = 2.0 Hz), 133.68, 132.82(t, J = 32.3 Hz), 132.19, 131.29, 130.37, 130.23(t, J = 3.5 Hz), 129.87, 126.53, 125.79(t, J = 3.5 Hz), 122.29, 121.54(t, J = 262.6Hz), 120.82, 52.52. **HRMS** (EI): m/z [$(M)^{\cdot+}$] calcd for $C_{15}H_{11}{}^{35}ClF_2O_3$, 312.0365. found, 312.0361.

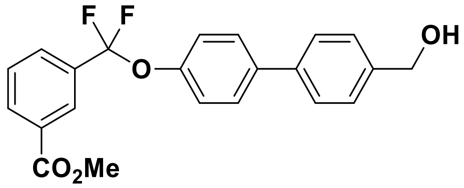

methyl 3-(difluoro((4'-(hydroxymethyl)-[1,1'-biphenyl]-4-yl)oxy)methyl)benzoate (7e).

Following the general procedure, preparative TLC (petroleum ether/ethyl acetate = 5/1) to afford **7e** as a white solid (55.3mg, 72% yield). **^{1}H NMR** (400 MHz, DMSO-d_6) δ 8.28 (s, 1H), 8.19 (d, J = 7.8, 1H), 8.08 (d, J = 7.9, 1H), 7.74 (m, 3H), 7.62 (d, J = 8.1, 2H), 7.45-7.37 (m, 4H), 5.31 (t, J = 5.7, 1H), 4.55 (d, J = 5.7, 2H), 3.90 (s, 3H). **^{19}F NMR** (376 MHz, DMSO-d_6) δ -63.98 (s, 2F). **^{13}C NMR** (101 MHz, DMSO-d_6) δ 165.30, 148.91, 142.09, 138.17, 137.37, 133.24(t, J = 32.8 Hz), 132.08, 130.37, 130.23(t, J = 4.0 Hz), 129.88, 127.91, 127.06, 126.38, 125.75(t, J = 3.5 Hz), 122.35,

121.63(t, J = 261.2 Hz), 62.54, 52.53. **HRMS** (APCI): m/z [(M-OH)$^+$] calcd for $C_{22}H_{17}F_2O_3$, 367.1140. found, 367.1145.

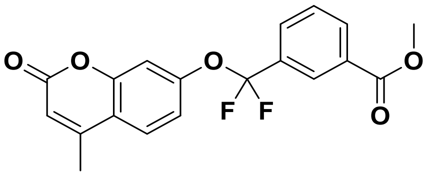

methyl 3-(difluoro((4-methyl-2-oxo-2H-chromen-7-yl)oxy)methyl)benzoate (7f).

Following the general procedure, preparative TLC (petroleum ether/ethyl acetate = 5/1) to afford **7f** as a white solid (41.0 mg, 57% yield). **^{1}H NMR** (400 MHz, DMSO-d_6) δ 8.27 (s, 1H), 8.18 (d, J = 7.8, 1H), 8.09 (d, J = 8.0, 1H), 7.83 (d, J = 8.7, 1H), 7.75 (t, J = 7.8, 1H), 7.40 (d, J = 2.1, 1H), 7.37-7.31 (m, 1H), 6.39 (d, J = 1.1, 1H), 3.90 (s, 3H), 2.42 (d, J = 1.1, 3H). **^{19}F NMR** (376 MHz, DMSO-d_6) δ -64.28 (s, 2F). **^{13}C NMR** (101 MHz, DMSO-d_6) δ 165.70, 159.94, 154.08, 153.20, 152.26, 133.12(t, J = 32.8 Hz), 132.73, 130.88, 130.73(t, J = 3.0 Hz), 130.39, 127.36, 126.28, 122.16(t, J = 263.6 Hz), 118.25, 118.18, 114.48, 110.12, 53.05, 18.54. **HRMS** (APCI): m/z [(M+H)$^+$] calcd for $C_{19}H_{15}F_2O_5$, 361.0882. found, 361.0894.

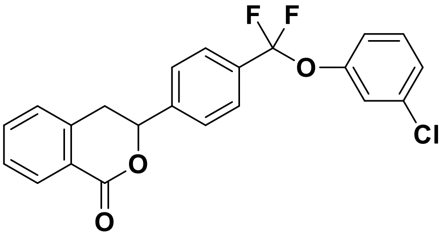

3-(4-((3-chlorophenoxy)difluoromethyl)phenyl)isochroman-1-one (7g).

Following the general procedure, preparative TLC (petroleum ether/ethyl acetate = 8/1) to afford **7g** as a white solid (55.2 mg, 69% yield). **^{1}H NMR** (400 MHz, DMSO-d_6) δ 7.85 (d, J = 8.4, 2H), 7.82 (dd, J = 7.8, 1.6, 1H), 7.75 (d, J = 8.3, 2H), 7.62 (m, 1H), 7.48 (dd, J = 14.3, 6.2, 2H), 7.40 (m, 1H), 7.37-7.31 (m, 1H), 7.17-7.09 (m, 2H), 5.81 (dd, J = 12.8, 2.8, 1H), 3.26 (dd, J = 16.8, 12.9, 1H), 2.92 (dd, J = 16.8, 3.0, 1H). **^{19}F NMR** (376 MHz, DMSO-d_6) δ -64.28 (s, 2F). **^{13}C NMR** (101 MHz, DMSO-d_6) δ 191.17, 160.85, 150.40, 142.50, 136.35, 133.65, 132.19(t, J = 31.8 Hz), 131.28, 126.96,

126.35, 126.29, 125.86(t, J = 3.5 Hz), 122.03, 122.01(t, J = 262.6 Hz), 121.66, 120.68, 120.57, 118.05, 78.12, 43.46. **HRMS** (APCI): m/z [(M+H)$^+$] calcd for $C_{22}H_{16}{}^{35}ClF_2O_3$, 401.0751. found, 401.0746.

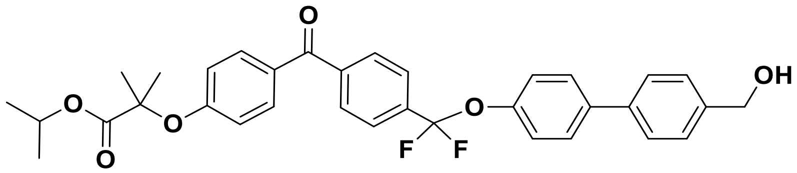

isopropyl 2-(4-(4-(difluoro((4'-(hydroxymethyl)-[1,1'-biphenyl]-4-yl)oxy)methyl)benzoyl)phenoxy)-2-methylpropanoate (7h).

Following the general procedure, preparative TLC (petroleum ether/ethyl acetate = 2/1) to afford **7h** as a white solid (64.2 mg, 56% yield). **^{1}H NMR** (400 MHz, DMSO-d$_6$) δ 7.98 (d, J = 8.3, 2H), 7.87 (d, J = 8.2, 2H), 7.76 (t, J = 9.1, 4H), 7.65 (d, J = 8.1, 2H), 7.43 (d, J = 8.3, 4H), 6.93 (d, J = 8.8, 2H), 5.27 (t, J = 5.7, 1H), 5.00 (m, 1H), 4.57 (d, J = 5.7, 2H), 1.62 (s, 6H), 1.16 (d, J = 6.2, 6H). **^{19}F NMR** (376 MHz, DMSO-d$_6$) δ -64.07 (s, 2F). **^{13}C NMR** (101 MHz, DMSO-d$_6$) δ 193.63, 172.07, 159.46, 148.99, 142.04, 140.34, 138.12, 137.41, 135.45(t, J = 31.8 Hz), 132.03, 129.63, 129.31, 127.92, 127.05, 126.38, 125.73(t, J = 3.5 Hz), 122.24, 121.77(t, J = 261.6 Hz), 117.17, 79.10, 68.95, 62.57, 25.00, 21.20. **HRMS** (APCI): m/z [(M-OH)$^+$] calcd for $C_{34}H_{31}F_2O_5$, 557.2134. found, 557.2151.

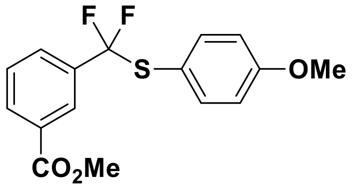

methyl 3-(difluoro((4-methoxyphenyl)thio)methyl)benzoate (7i).

Following the general procedure, crude product was dissolved in 2 ml DCM, mCPBA (75% purity) (69 mg, 1.5 mmol) was added at 0 ^{o}C, after stirring 30 min at same temperature, solvent was removed under vacuum, which obliterated by-product diphenyl sulfide. The residue was purified on a preparative TLC (petroleum ether/ethyl acetate = 25/1) to afford **7i** as a white solid (34.3mg, 53% yield). **^{1}H NMR** (400 MHz,

DMSO-d_6) δ 8.14 (d, J = 7.8, 1H), 8.08 (s, 1H), 7.85 (d, J = 8.0, 1H), 7.69 (t, J = 7.8, 1H), 7.54 (d, J = 8.8, 2H), 7.08-7.01 (m, 2H), 3.91 (s, 3H), 3.82 (s, 3H). **^{19}F NMR** (376 MHz, DMSO-d_6) δ -71.91 (s, 2F). **^{13}C NMR** (101 MHz, DMSO-d_6) δ 165.18, 161.23, 138.10, 135.52(t, J = 26.2 Hz), 131.59, 130.13, 129.57(t, J = 4.0 Hz), 129.53, 127.09(t, J = 278.3 Hz), 125.48(t, J = 4.5 Hz), 116.19, 114.97, 55.31, 52.38. **HRMS** (EI): m/z [(M)$^{\cdot+}$] calcd for $C_{16}H_{14}F_2O_3{}^{32}S$, 324.0632. found, 324.0645.

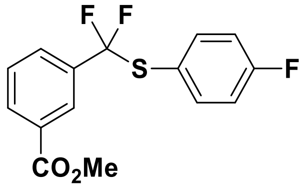

methyl 3-(difluoro((4-fluorophenyl)thio)methyl)benzoate (7j).

Following the general procedure, crude product was dissolved in 2 ml DCM, mCPBA (75% purity) (69 mg, 1.5 mmol) was added at 0 ^{o}C, after stirring 30 min at same temperature, solvent was removed under vacuum, which obliterated by-product diphenyl sulfide. The residue was purified on a preparative TLC (petroleum ether/ethyl acetate = 25/1) to afford **7j** as a white solid (25.0mg, 40% yield). **^{1}H NMR** (400 MHz, DMSO-d_6) δ 8.15 (d, J = 7.8, 1H), 8.08 (s, 1H), 7.87 (d, J = 8.0, 1H), 7.75-7.67 (m, 3H), 7.38-7.29 (m, 2H), 3.92 (s, 3H). **^{19}F NMR** (376 MHz, DMSO-d_6) δ -71.37 (s, 2F), -109.82 (s, 1F). **^{13}C NMR** (101 MHz, DMSO-d_6) δ 165.15, 163.65(d, J = 250.5 Hz), 138.78(d, J = 9.1 Hz), 135.21(t, J = 25.3 Hz), 131.79, 130.22, 129.68, 129.62(t, J = 4.0 Hz), 127.03(t, J = 278.8 Hz), 125.46(t, J = 5.0 Hz), 121.62(d, J = 3.0 Hz), 116.65(d, J = 23.2 Hz), 52.44. **HRMS** (EI): m/z [(M)$^{\cdot+}$] calcd for $C_{15}H_{11}F_3O_2{}^{32}S$, 312.0432. found, 312.0438.

4.3 Procedures for the synthesis of PD-1/PD-L1 interaction inhibitors

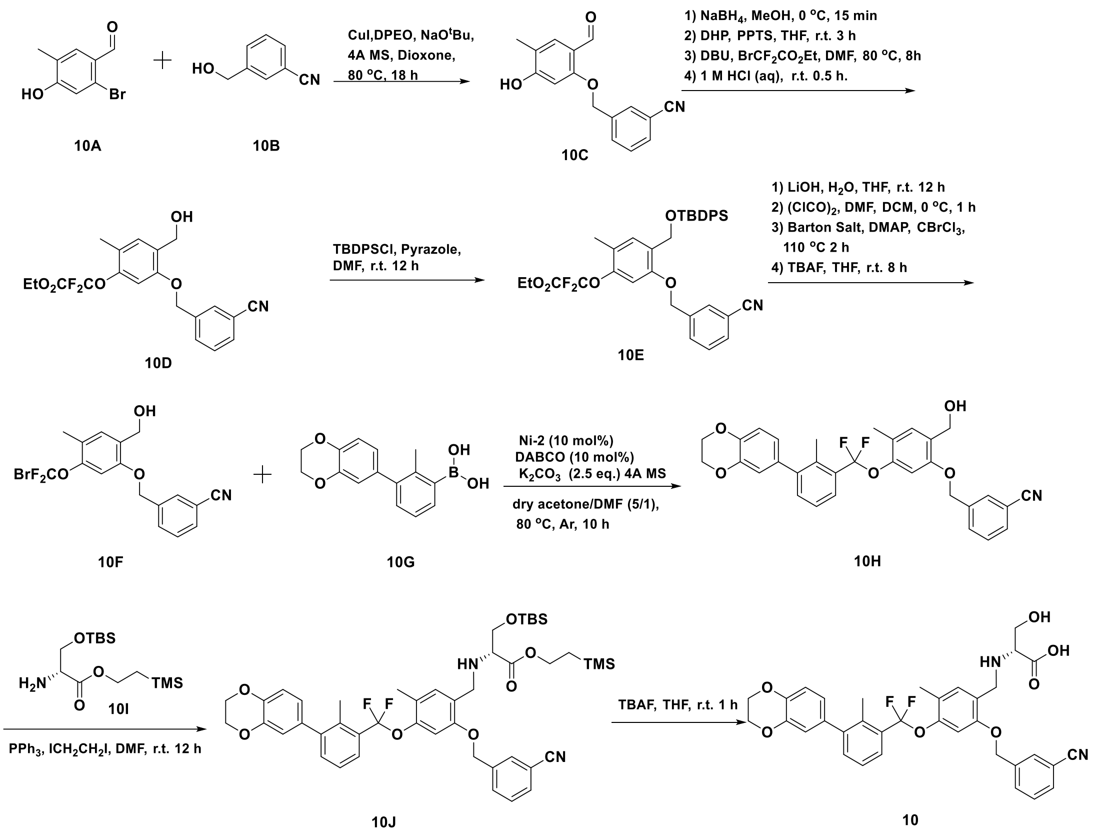

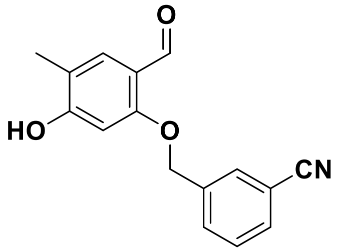

3-((2-formyl-5-hydroxy-4-methylphenoxy)methyl)benzonitrile (10C).

The 2-bromo-4-hydroxy-5-methylbenzaldehyde **10A**[17] (1.1 g, 5.0 mmol, 1.0 equiv), *t*-BuONa (1.1 g, 11 mmol, 2.2 equiv), CuI (95.2 mg, 0.5 mmol, 10 mol%), ligand DPEO (148.2 mg, 0.5 mmol, 10 mol%), 3-(hydroxymethyl)benzonitrile **10B** (1.3g, 10.0 mmol) and freshly activated 4 Å MS (400 mg) were placed into a Schlenk tube (100 mL) with a magnetic stir bar. The reaction vessel was evacuated and backfilled with argon three times, then 1,4-dioxane (15 mL) were added under positive argon pressure The reaction mixture was heated at 80 °C for 18 h under vigorous stirring. The cooled solution was acidified with 2 N HCl, then diluted with ethyl acetate and washed with brine. The organic phase was dried over Na_2SO_4 and concentrated in vacuo. The residue was

purified by silica gel flash chromatography (petroleum ether/ethyl acetate =10/1 to 1/1) to afford the corresponding ether **10C** as a light red solid (570 mg, 43% yield).[19] **^{1}H NMR** (400 MHz, DMSO-d$_6$) δ 10.65 (s, 1H), 10.20 (s, 1H), 7.98 (s, 1H), 7.84 (t, J = 8.6, 2H), 7.63 (d, J = 7.8, 1H), 7.48 (s, 1H), 6.58 (s, 1H), 5.23 (s, 2H), 2.07 (s, 3H). **^{13}C NMR** (101 MHz, DMSO-d$_6$) δ 186.96, 162.92, 160.51, 138.31, 132.17, 131.73, 130.89, 130.21, 129.79, 118.69, 117.66, 116.87, 111.49, 99.60, 68.49, 14.96. **HRMS** (APCI): m/z [(M+H)$^+$] calcd for $C_{16}H_{14}NO_3$, 268.0968. found, 268.0972.

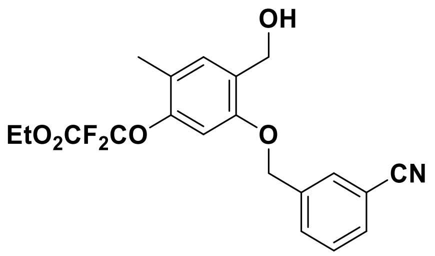

ethyl 2-(5-((3-cyanobenzyl)oxy)-4-(hydroxymethyl)-2-methylphenoxy)-2,2-difluoroacetate (10D).

To a 50 mL of round-bottom flask were added **10C** (1.1 g, 4 mmol, 1.0 equiv), MeOH (20 mL) at 0 ^{o}C. Then, $NaBH_4$ (76 mg, 2 mmol, 0.5 equiv) was added carefully. After stirring for 15 min at same temperature, solvent was removed under vacuum. The residue was used for next step without further purification.

The crude product was dissolved in 10 ml dry THF, 3,4-dihydro-2H-pyran (403 mg, 4.8 mmol, 1.2 equiv) and p-toluenesulfonic acid (34.4 mg, 0.2 mmol, 5 mol%) were added successively at Ar atmosphere. after stirring 3 h at room temperature, solvent was removed under vacuum, the residue was used for next step without further purification.

To a 50 mL of round-bottom flask were added crude product, DBU (912 mg, 6.0 mmol, 1.5 equiv), $BrCF_2CO_2Et$ (1.6 g, 8 mmol, 2.0 equiv), DMF (15 mL). After stirring for 5 h at 80 ^{o}C and cooled down to room temperature. The cooled solution was acidified with 1 N HCl (aq) and stirring for 30 min, then diluted with EA and washed with brine. The organic phase was dried over Na_2SO_4 and concentrated in vacuo. The residue was purified by silica gel flash chromatography (petroleum ether/ethyl acetate =20/1 to 4/1)

to afford compound **10D** as a white solid (910 mg, 58% yield). **^{1}H NMR** (400 MHz, $CDCl_3$) δ 7.71 (s, 1H), 7.68-7.61 (m, 2H), 7.51 (t, J = 7.7, 1H), 7.22 (s, 1H), 6.78 (s, 1H), 5.08 (s, 2H), 4.70 (s, 2H), 4.39 (q, J = 7.1, 2H), 2.23 (s, 3H), 2.03 (s, 1H), 1.37 (t, J = 7.1, 3H). **^{19}F NMR** (376 MHz, $CDCl_3$) δ -75.94 (s, 2F). **^{13}C NMR** (101 MHz, $CDCl_3$) δ 159.73(t, J = 41.4 Hz), 153.98, 147.32, 137.93, 131.80, 131.45, 131.11, 130.61, 129.52, 127.59, 123.94, 118.47, 114.08(t, J = 273.7 Hz), 112.85, 106.34, 69.08, 63.69, 60.75, 15.34, 13.80. **HRMS** (APCI): m/z [$(M\text{-}OH)^+$] calcd for $C_{20}H_{18}F_2NO_4$, 374.1198. found, 374.1198.

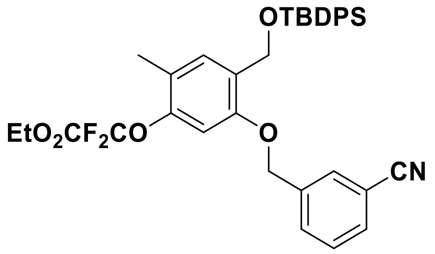

ethyl 2-(4-(((tert-butyldiphenylsilyl)oxy)methyl)-5-((3-cyanobenzyl)oxy)-2-methylphenoxy)-2,2-difluoroacetate (10E).

To a 25 mL of round-bottom flask were added **10D** (1.2 g, 3 mmol, 1.0 equiv), pyrazole (0.3 g, 4.5 mmol, 1.5 equiv), TBDPSCl (1.1 g, 3.9 mmol, 1.3 equiv), DMF (5 mL). After stirring for 12 h at room temperature, Water (50 mL) was added and the mixture was extracted with EA (15 mL×3). The combined organic layers were washed with brine, dried over Na_2SO_4, and concentrated under reduced pressure. The residue was purified by flash column chromatography (Petroleum ether /Ethyl Acetate = 30:1 to 15:1) to afford compound **10E** as a colorless oil-like liquid (1.7 g, 90% yield). **^{1}H NMR** (400 MHz, $CDCl_3$) δ 7.69 (dd, J = 7.9, 1.3, 4H), 7.58 (d, J = 7.6, 1H), 7.54 (s, 1H), 7.50-7.31 (m, 9H), 6.70 (s, 1H), 4.95 (s, 2H), 4.79 (s, 2H), 4.40 (q, J = 7.1, 2H), 2.28 (s, 3H), 1.38 (t, J = 7.1, 3H), 1.11 (s, 9H). **^{19}F NMR** (376 MHz, $CDCl_3$) δ -75.85 (s, 2F). **^{13}C NMR** (101 MHz, $CDCl_3$) δ 159.91(t, J = 41.4 Hz), 153.00, 146.74, 138.23, 135.58, 133.42, 131.61, 131.31, 130.39, 130.00, 129.77, 129.35, 128.04, 127.73, 123.71, 118.53, 114.22(t, J = 273.2 Hz), 112.75, 106.03, 68.94, 63.68, 60.65, 26.88, 19.35,

15.68, 13.88. **HRMS** (APCI): m/z [(M+H)$^+$] calcd for $C_{33}H_{33}{}^{79}BrF_2NO_3Si$, 630.2482. found, 630.2493.

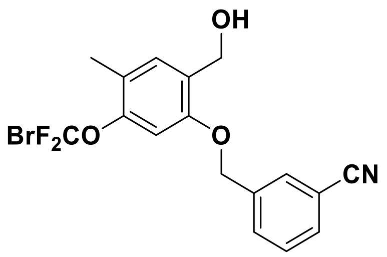

3-((5-(bromodifluoromethoxy)-2-(hydroxymethyl)-4-methylphenoxy)methyl)benzonitrile (10F).

To a 25 mL of round-bottom flask equipped with a stir bar were added **10E** (1.3 g, 2 mmol, 1.0 equiv), LiOH.H_2O (170 mg, 4 mmol, 2.0 equiv), THF (5 mL) and H_2O (5 mL). After the mixture was stired at room temperature for 12 h. The mixture was acidified with 3M HCl (aq) to pH = 1 and was extracted with ethyl acetate for 3 times. The combined organic phase was washed by saturated brines and dried over $NaSO_4$. After the solution was filtered and the solvent was evaporated under vacuum, and dried in vacuo to afford the corresponding carboxylic acid as a white solid, which was used for next step without further purification

To a 25 mL of round-bottom flask were added carboxylic acid in DCM (6 mL), then added DMF (15 µL, 0.2 mmol, 0.1 equiv) and oxalyl chloride (258 µL, 3 mmol, 1.5 equiv) at 0 ^{o}C. The reaction mixture was stirred at room temperature for 1 hours, then concentrated in vacuo. The crude acyl chloride was added $BrCCl_3$ (8 mL), 4-Dimethylaminopyridine (49 mg, 0.4 mmol, 0.2 equiv) and sodium-N-hydroxy-2-thiopyridone (300 mg, 2.0 mmol, 1.0 equiv). The reaction mixture was refluxed at 120 ^{o}C for 2 hours under Ar then concentrated in vacuo. The residue was dissolved in THF (10 mL), and TBATF (3 mL, 3 mmol, 1.5 equiv, 1M in THF) was added. After stirring for 8 h at room temperature, solvent was concentrated under reduced pressure. The crude product was purified by silica gel column chromatography (Petroleum ether /Ethyl Acetate = 30:1 to 5:1) to afford compound **10F** as a white solid (294 mg, 37% yield). **^{1}H NMR** (400 MHz, $CDCl_3$) δ 7.72 (s, 1H), 7.65 (t, J = 7.9, 2H), 7.52 (t, J = 7.7,

1H), 7.27 (s, 1H), 6.79 (s, 1H), 5.12 (s, 2H), 4.73 (s, 2H), 2.25 (s, 3H), 1.89 (s, 1H). **^{19}F NMR** (376 MHz, $CDCl_3$) δ -14.48 (s, 2F). **^{13}C NMR** (101 MHz, $CDCl_3$) δ 154.06, 148.91, 137.82, 131.89, 131.45, 131.35, 130.64, 129.60, 128.20, 123.41, 118.43, 114.62(t, J = 310.1 Hz), 112.97, 105.57, 69.18, 60.75, 29.64, 15.57. **HRMS** (APCI): m/z [$(M-OH)^+$] calcd for $C_{17}H_{13}{}^{79}BrF_2NO_2$, 380.092. found, 380.0094.

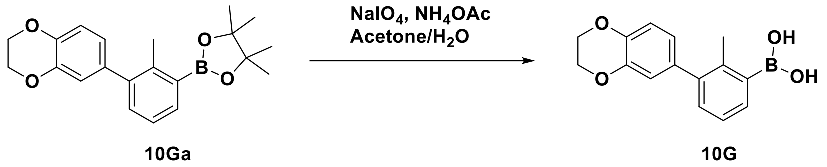

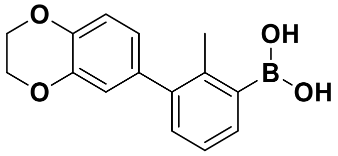

(3-(2,3-dihydrobenzo[b][1,4]dioxin-6-yl)-2-methylphenyl)boronic acid (10G).

To a 100 mL round-bottom flask were added **10Ga** (352 mg, 1 mmol, 1.0 equiv), $NaIO_4$ (1.3 g, 6 mmol, 6.0 equiv), NH_4OAc (925 mg, 6 mmol, 6.0 equiv), acetone (25 mL), and H_2O (13 mL). After stirring for 24 h at roomtemperature, the reaction mixture was filtered with a pad of cellite. The filtrate was concentrated, theresulting solid was washed with H_2O (6 mL), pentane (6 mL×3), and dried in vacuo to afford the corresponding boronic acid as a white solid (110 mg, 41% yield). **^{1}H NMR** (400 MHz, DMSO-d_6) δ 7.61 (d, J = 8.7, 2H), 7.57 (d, J = 8.2, 2H), 7.45 (d, J = 8.2, 2H), 7.32 (d, J = 8.7, 2H), 4.74 (s, 2H), 2.11 (s, 1H). **^{13}C NMR** (101 MHz, DMSO-d_6) δ 143.37, 142.77, 140.99, 138.92, 137.63, 135.75, 131.69, 130.19, 125.13, 122.42, 117.99, 117.21, 64.57, 64.54, 20.76. **HRMS** (APCI): m/z [$(M+Cl)^{\cdot+}$] calcd for $C_{15}H_{15}BO_4$, 305.0752. found, 305.0761.

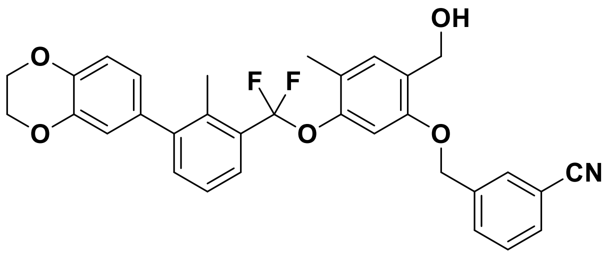

3-((5-((3-(2,3-dihydrobenzo[b][1,4]dioxin-6-yl)-2-methylphenyl)difluoromethoxy)-2-(hydroxymethyl)-4-methylphenoxy)methyl)benzonitrile (10H).

To a dried 10 ml Schlenk-type tube equipped with a magnetic stir bar was charged with arylboronic acid **10G** (108 mg, 0.4 mmol, 2.0 equiv), Ni-2 (8.0 mg, 0.02 mmol, 10 mol %), DABCO (2.3 mg, 0.02 mmol, 10 mol %), 100 mg 4 Å MS and K_2CO_3 (69.0 mg, 0.5 mmol, 2.5 equiv) under air. The reaction mixture was then evacuated and backfilled with Ar (3 times). Compound **10F** (80 mg, 0.2mmol, 1.0 equiv), and acetone (2.5 mL) and DMF (0.5 mL) were added. The mixture was stirred at 80 °C for 10 h. After cooled to room temperature, the reaction mixture was filtered and the filtrate was concentrated. The residue was purified on a preparative TLC with petroleum ether/ethyl acetate (20:1 to 4:1) as the eluent to afford the products **10H** as a white solid (57 mg, 53% yield). **^{1}H NMR** (400 MHz, CD_2Cl_2) δ 7.86-7.72 (m, 2H), 7.70 (d, J = 7.7, 1H), 7.64 (d, J = 7.5, 1H), 7.52 (t, J = 7.7, 1H), 7.38 (d, J = 7.4, 1H), 7.32 (t, J = 7.6, 1H), 7.27 (s, 1H), 7.03-6.89 (m, 2H), 6.84 (s, 1H), 6.79 (d, J = 8.2, 1H), 5.11 (s, 2H), 4.72 (s, 2H), 4.29 (s, 4H), 2.49 (s, 3H), 2.46 (s, 1H), 2.26 (s, 3H). **^{19}F NMR** (376 MHz, CD_2Cl_2) δ -64.22 (s, 2F). **^{13}C NMR** (101 MHz, CD_2Cl_2) δ 154.54, 148.93, 144.27, 143.80, 143.48, 138.87, 135.12, 134.96, 133.23, 132.89(t, J = 29.2 Hz), 132.24, 132.07, 131.28, 131.22, 129.99, 127.86, 125.74, 125.51(t, J = 6.6 Hz), 124.22, 123.64(t, J = 265.6 Hz), 122.94, 119.12, 118.68, 117.40, 113.24, 107.35, 69.67, 65.04, 60.95, 17.52, 16.25. **HRMS** (APCI): m/z [$(M-OH)^+$] calcd for $C_{32}H_{26}F_2NO_4$, 526.1824. found, 526.1832.

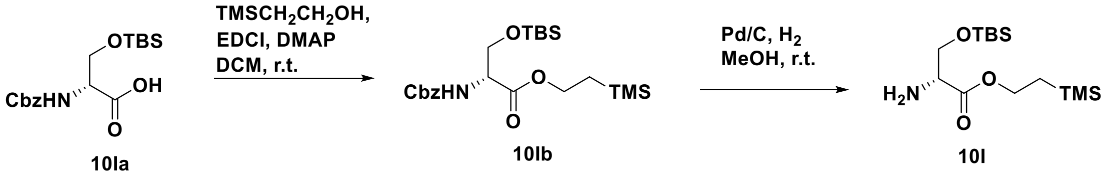

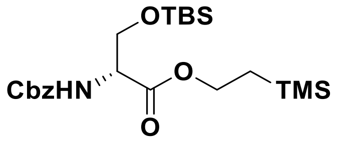

2-(trimethylsilyl)ethyl N-((benzyloxy)carbonyl)-O-(tert-butyldimethylsilyl)-D-serinate (10Ib).

To a 100 mL of round-bottom flask were added carboxylic acid **10Ia**[20](1.8 g, 5.0 mmol, 1.0 equiv) in DCM (40 mL), then added 2-(trimethylsilyl)ethanol (885 mg, 7.5 mmol, 1.5 equiv), EDCI (1.1 g, 6.0 mmol, 1.2 equiv) and DMAP (915 mg, 7.5 mmol, 1.5 equiv) at 0 ^{o}C. The reaction mixture was stirred at room temperature for 12 hours, then concentrated in vacuo. The crude product was purified by silica gel column chromatography (Petroleum ether /Ethyl Acetate = 30:1 to 5:1) to afford the products **10Ib** as a white solid (2.1 g, 92% yield). **^{1}H NMR** (400 MHz, $CDCl_3$) δ 7.42-7.27 (m, 5H), 5.60 (d, J = 8.5, 1H), 5.13 (s, 2H), 4.47-4.29 (m, 1H), 4.30-4.16 (m, 2H), 4.07 (dd, J = 10.0, 2.5, 1H), 3.79 (dd, J = 33.0, 4.7, 1H), 1.00 (t, J = 8.6, 2H), 0.85 (s, 9H), 0.04 (s, 9H), 0.02 (d, J = 2.8, 6H). **^{13}C NMR** (101 MHz, $CDCl_3$) δ 170.46, 155.91, 136.31, 128.46, 128.09, 128.07, 66.88, 63.82, 63.62, 56.03, 25.66, 18.13, 17.34, -1.61, -5.59, -5.71. **HRMS** (APCI): m/z [(M+H)$^+$] calcd for $C_{22}H_{40}NO_5Si_2$, 454.2440. found, 454.2439.

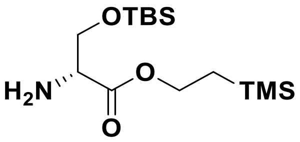

2-(trimethylsilyl)ethyl O-(tert-butyldimethylsilyl)-D-serinate (10I).

To a 100 ml three-necked, round-bottomed flask equipped with a magnetic stir bar was charged with amino acid **10Ib** (2.0 g, 4.5 mmol, 1.0 equiv), Pd/C (Palladium 10% on Carbon (wetted with ca. 55% Water)) (200 mg, 10 wt. %) and MeOH (30 mL) under air. The reaction mixture was then evacuated and backfilled with H_2 (3 times) and was stirred at H_2 atmosphere for 18 hours. The reaction mixture was filtered and the filtrate was concentrated in vacuo. The residue was purified by silica gel column chromatography (Petroleum ether /Ethyl Acetate = 10:1 to 2:1) to afford the products **10I** as a colorless oil-like liquid (1.4 g, 99% yield). **^{1}H NMR** (400 MHz, $CDCl_3$) δ 4.26-4.15 (m, 2H), 3.90 (dd, J = 9.7, 4.5, 1H), 3.81 (dd, J = 9.7, 3.7, 1H), 3.48 (t, J = 4.1, 1H), 1.78 (s, 2H), 1.00 (t, J = 8.6, 2H), 0.87 (s, 9H), 0.05 (s, 3H), 0.04 (m, 12H). **^{13}C**

NMR (101 MHz, $CDCl_3$) δ 174.11, 65.34, 63.24, 56.58, 25.74, 18.20, 17.41, -1.55, -5.49, -5.59. **HRMS** (APCI): m/z [$(M+H)^+$] calcd for $C_{14}H_{34}NO_3Si_2$, 320.2072 found, 320.2069.

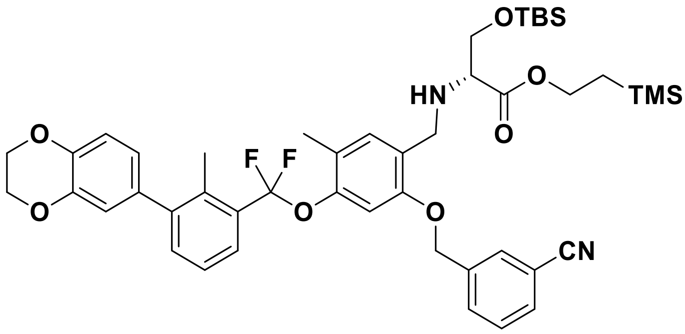

2-(trimethylsilyl)ethyl O-(tert-butyldimethylsilyl)-N-(2-((3-cyanobenzyl)oxy)-4-((3-(2,3-dihydrobenzo[b][1,4]dioxin-6-yl)-2-methylphenyl)difluoromethoxy)-5-methylbenzyl)-D-serinate (10J).

Alcohol **10H** (54 mg, 0.1 mmol, 1.0 equiv), triphenylphosphine (32 mg, 0.12 mmol, 1.2 equiv) and anhydrous DMF (1.0 mL) were added into a 10 mL sealed tube under a Ar atmosphere. 1,2-Diiodoethane (34 mg, 0.12 mmol, 1.2 equiv) was then added and the resulting mixture was stirred for around 10 min until the 1,2-diiodoethane was completely dissolved. Amine **10I** (96 mg, 0.3 mmol, 3.0 equiv) was added subsequently and the mixture was stirred at room temperature for 12 h. Dichloromethane (20 mL) was added and the resulting solution was washed with water (20 mL x 3). The organic layer was dried over Na_2SO_4. After filtration, the solvent was removed by concentration under reduced pressure. The residue was purified by silica gel column chromatography (Petroleum ether /Ethyl Acetate = 10:1 to 1:1) to afford the products **10J** as a white solid (54 mg, 64% yield).[21] **^{1}H NMR** (400 MHz, CD_2Cl_2) δ 7.82 (s, 1H), 7.77 (dd, J = 10.9, 8.1, 2H), 7.65 (d, J = 7.7, 1H), 7.53 (t, J = 7.7, 1H), 7.37 (d, J = 7.1, 1H), 7.32 (t, J = 7.6, 1H), 7.26 (s, 1H), 6.93 (d, J = 8.1, 2H), 6.83 (d, J = 2.0, 1H), 6.78 (dd, J = 8.2, 2.0, 1H), 5.11 (s, 2H), 4.30 (s, 4H), 4.20-4.15 (m, 2H), 3.98-3.78 (m, 4H), 3.41 (m, 1H), 2.64 (s, 1H), 2.48 (s, 3H), 2.26 (s, 3H), 1.03-0.97 (m, 2H), 0.88 (s, 9H), 0.07 (s, 9H), 0.06 (s, 6H). **^{19}F NMR** (376 MHz, CD_2Cl_2) δ -64.21 (s, 2F). **^{13}C NMR** (101 MHz,

CD_2Cl_2) δ 173.69, 155.11, 148.69, 144.35, 143.88, 143.56, 139.18, 135.22, 135.03, 133.25, 133.05(t, J = 29.3 Hz), 132.62, 132.17, 132.08, 131.27, 129.97, 126.75, 125.76, 125.56(t, J = 6.1 Hz), 124.08, 123.68(t, J = 265.1 Hz), 122.99, 119.16, 118.73, 117.46, 113.35, 107.55, 69.83, 65.38, 65.10, 63.42, 63.29, 47.13, 26.15, 18.69, 17.98, 17.57, 16.32, -1.26, -5.18, -5.22. **HRMS** (APCI): m/z [$(M+H)^+$] calcd for $C_{46}H_{59}F_2N_2O_7Si_2$, 845.3824. found, 845.3838.

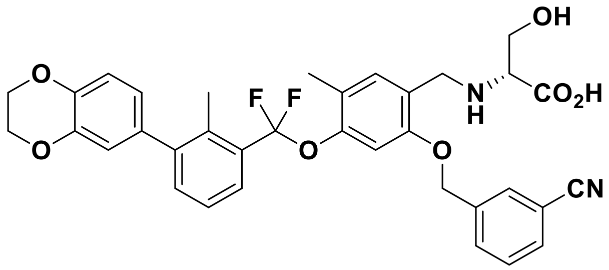

(2-((3-cyanobenzyl)oxy)-4-((3-(2,3-dihydrobenzo[b][1,4]dioxin-6-yl)-2-methylphenyl)difluoromethoxy)-5-methylbenzyl)-D-serine (10).

To a 25 mL of round-bottom flask were added **10J** (42 mg, 0.05 mmol, 1.0 equiv), TBATF (120 µL, 0.12 mmol, 2.4 equiv, 1M in THF) and THF (1 mL). After stirring for 1 h at room temperature, solvent was concentrated under reduced pressure. The residue was purified by recrystallized from MeOH as a white solid (14 mg, 45% yield). **^{1}H NMR** (400 MHz, DMSO-d_6) δ 8.00 (s, 1H), 7.90 (d, J = 7.7, 1H), 7.81 (d, J = 7.7, 1H), 7.78-7.72 (m, 1H), 7.61 (t, J = 7.8, 1H), 7.48-7.26 (m, 3H), 7.00 (s, 1H), 6.94 (d, J = 8.2, 1H), 6.82 (s, 1H), 6.78 (d, J = 8.2, 1H), 5.22 (s, 2H), 4.29 (s, 4H), 4.01 (s, 2H), 3.79-3.68 (m, 1H), 3.63 (m, 1H), 3.32 (s, 1H), 3.23-3.16 (m, 1H), 2.39 (s, 3H), 2.18 (s, 3H). **^{19}F NMR** (376 MHz, CD_2Cl_2) δ -62.91 (s, 2F). **^{13}C NMR** (176 MHz, DMSO-d_6) δ 170.27, 154.94, 148.69, 143.78, 143.54, 143.25, 138.81, 134.13, 134.01, 133.34, 132.94, 132.21, 132.06(t, J = 28.2 Hz), 131.90 131.60, 130.21, 126.20, 125.31(t, J = 5.3 Hz), 123.16(t, J = 264.0 Hz), 123.15, 122.64, 122.28, 119.16, 118.28, 117.38, 111.90, 107.77, 69.22, 64.57, 63.00, 61.36, 45.55, 31.17, 17.35, 16.03. **HRMS** (APCI): m/z [$(M+H)^+$] calcd for $C_{35}H_{33}F_2N_2O_7$, 631.2251. found, 631.2263.

5. Preliminary mechanistic experiments

5.1 Radical inhibition experiments

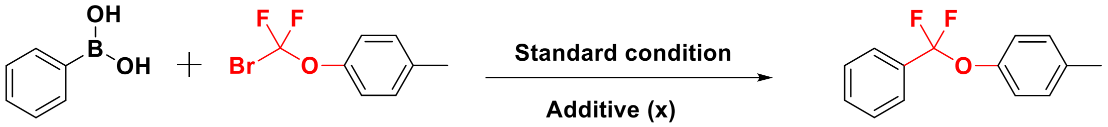

1a 2a 3a

Entry	Additive (x)	Yield/%[a]
1	None	90
2	TEMPO (1.0 equiv.)	0
3	PBN (1.0 equiv.)	32
4	1.4-dinitrobenzene (1.0 equiv.)	0

Reaction conditions: unless otherwise noted, a solution of 1a (0.2 mmol), 2a (0.4 mmol), Ni-2 (10 mol%), DABCO (10 mol%), Additive (x) and K_2CO_3 (0.5 mmol) in dry acetone (3.0 mL) was performed at 80 °C under argon for 10 h. The yields were determined by GC-MS with n-dodecane as an internal standard.

General Procedure: To a dried 10 ml Schlenk-type tube equipped with a magnetic stir bar was charged with arylboronic acid **1a** (49.0 mg, 0.4 mmol, 2.0 equiv), Ni-2 (8.0 mg, 0.02 mmol, 10 mol %), DABCO (2.3 mg, 0.02 mmol, 10 mol %) and K_2CO_3 (69.0 mg, 0.5 mmol, 2.5 equiv) under air. The reaction mixture was then evacuated and backfilled with Ar (3 times). 1-(bromodifluoromethoxy)-4-methylbenzene **2a** (47.0 mg, 0.2mmol, 1.0 equiv), and acetone (3 mL) were added. The mixture was stirred at 80 °C for 10 h. The reaction mixture was cooled to room temperature. The yields were determined by GC-MS with n-dodecane as an internal standard.

5.2 Radical clock experiments

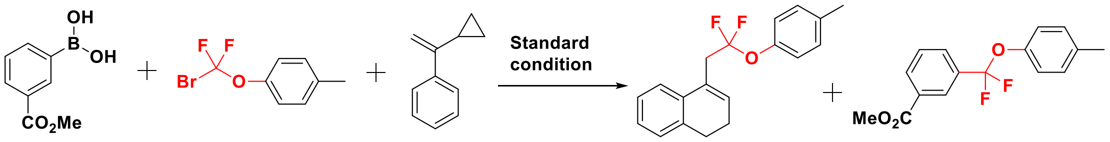

1s (2.0 equiv) **2a** (1.0 equiv) **8** (4.0 equiv) **9**, 16% **3s**, 32%

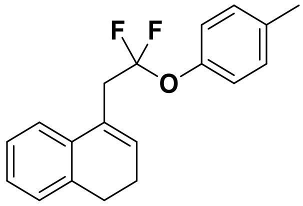

4-(2,2-difluoro-2-(p-tolyloxy)ethyl)-1,2-dihydronaphthalene (9).

To a dried 10 ml Schlenk-type tube equipped with a magnetic stir bar was charged with arylboronic acid **1s** (72.0 mg, 0.4 mmol, 2.0 equiv), Ni-2 (8.0 mg, 0.02 mmol, 10 mol %), DABCO (2.3 mg, 0.02 mmol, 10 mol %) and K_2CO_3 (69.0 mg, 0.5 mmol, 2.5 equiv) under air. The reaction mixture was then evacuated and backfilled with Ar (3 times). 1-(bromodifluoromethoxy)-4-methylbenzene **2a** (47.0 mg, 0.2mmol, 1.0 equiv), (1-cyclopropylvinyl)benzene **8** (115.2 mg, 0.8mmol, 4.0 equiv) and acetone (3 mL) were added. The mixture was stirred at 80 °C for 10 h. After cooled to room temperature, the reaction mixture was filtered and the filtrate was concentrated. The crude product was purified by silica gel column chromatography (Petroleum ether /Ethyl Acetate = 50:1) to afford compound **9** as a colorless oil-like liquid (9.6mg, 16% yield). **^{1}H NMR** (400 MHz, DMSO-d_6) δ 7.39 (d, J = 7.5, 1H), 7.24-7.11 (m, 5H), 7.00 (d, J = 8.3, 2H), 6.18 (t, J = 4.5, 1H), 3.40 (t, J = 11.7, 2H), 2.71 (t, J = 8.1, 2H), 2.30-2.22 (m, 5H). **^{19}F NMR** (376 MHz, DMSO-d_6) δ -67.17 (t, J = 11.3, 2F). **^{13}C NMR** (101 MHz, DMSO-d_6) δ 147.51, 135.78, 134.90, 133.93, 131.39, 129.96, 127.99, 127.43, 126.88, 126.23, 124.02, 123.35, 121.39, 37.70(t, J = 29.8 Hz), 27.50, 22.71, 20.28. **HRMS** (EI): m/z [(M)$^{\cdot+}$] calcd for $C_{19}H_{18}F_2O$, 300.1326. found, 300.1332.

5.3 Studies of the role of DABCO in the reaction

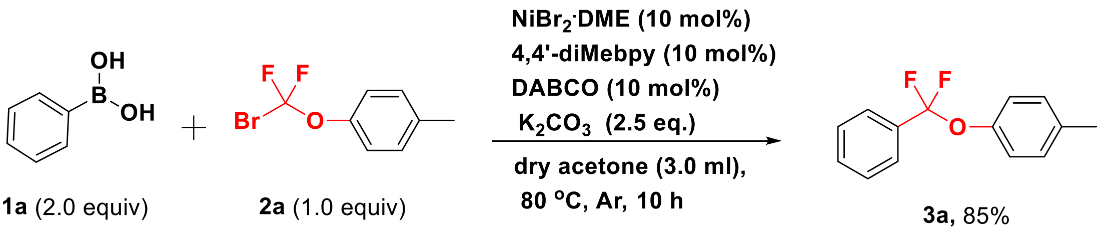

To a dried 10 ml Schlenk-type tube equipped with a magnetic stir bar was charged with arylboronic acid **1a** (49.0 mg, 0.4 mmol, 2.0 equiv), $NiBr_2 \cdot DME$ (6.2 mg, 0.02 mmol,

10 mol %), 4,4'-diMebpy (3.7 mg, 0.02 mmol, 10 mol %), DABCO (2.3 mg, 0.02 mmol, 10 mol %) and K_2CO_3 (69.0 mg, 0.5 mmol, 2.5 equiv) under air. The reaction mixture was then evacuated and backfilled with Ar (3 times). 1-(bromodifluoromethoxy)-4-methylbenzene **2a** (47.0 mg, 0.2mmol, 1.0 equiv) and acetone (3 mL) were added. The mixture was stirred at 80 °C for 10 h. The reaction mixture was cooled to room temperature. The yields were determined by GC-MS with n-dodecane as an internal standard.

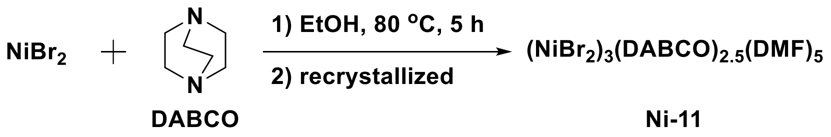

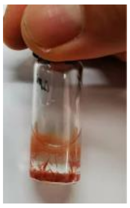

Preparation of Ni-11: To a stirring solution of DABCO (336 mg, 3.0 mmol, 1.0 equiv) in EtOH (30 mL) was added $NiBr_2$ (648 mg, 3.0 mmol, 1.0 equiv), the reaction mixture was stirred at 80 °C for another 5 h, then the solution was filtrated and the filtrate waswhich was recrystallized from DMF and ^{t}BuOMe to give an orange solid.

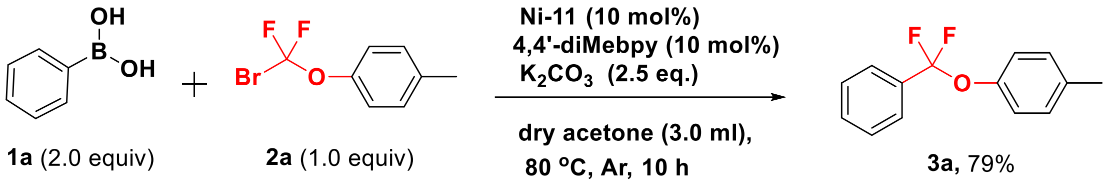

To a dried 10 ml Schlenk-type tube equipped with a magnetic stir bar was charged with arylboronic acid **1a** (49.0 mg, 0.4 mmol, 2.0 equiv), **Ni-11** (9.5 mg, 0.02 mmol, 10 mol %), 4,4'-diMebpy (3.7 mg, 0.02 mmol, 10 mol %) and K_2CO_3 (69.0 mg, 0.5 mmol, 2.5 equiv) under air. The reaction mixture was then evacuated and backfilled with Ar (3 times). 1-(bromodifluoromethoxy)-4-methylbenzene **2a** (47.0 mg, 0.2mmol, 1.0 equiv), and acetone (3 mL) were added. The mixture was stirred at 80 °C for 10 h. The reaction mixture was cooled to room temperature. The yields were determined by GC-MS with n-dodecane as an internal standard.

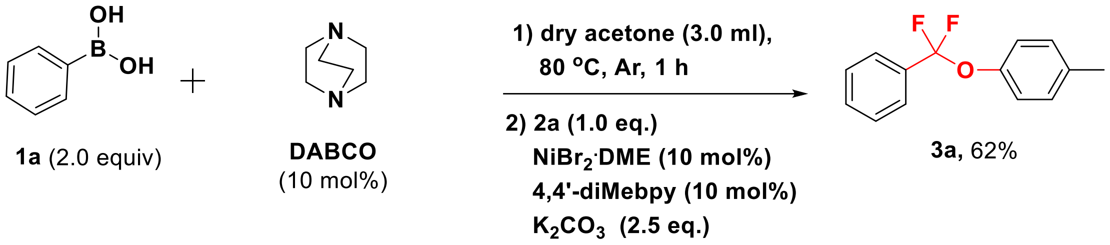

To a dried 10 ml Schlenk-type tube equipped with a magnetic stir bar was charged with arylboronic acid **1a** (49.0 mg, 0.4 mmol, 2.0 equiv) and DABCO (2.3 mg, 0.02 mmol, 10 mol %) under air. The reaction mixture was then evacuated and backfilled with Ar (3 times) and acetone (3 mL) were added. After stirring at 80 °C for 1 h. $NiBr_2$·DME (6.2 mg, 0.02 mmol, 10 mol %), 4,4'-diMebpy (3.7 mg, 0.02 mmol, 10 mol %), DABCO (2.3 mg, 0.02 mmol, 10 mol %), K_2CO_3 (69.0 mg, 0.5 mmol, 2.5 equiv) and 1-(bromodifluoromethoxy)-4-methylbenzene **2a** (47.0 mg, 0.2mmol, 1.0 equiv) were added. The mixture was stirred at 80 °C for 10 h. The reaction mixture was cooled to room temperature. The yields were determined by GC-MS with n-dodecane as an internal standard.

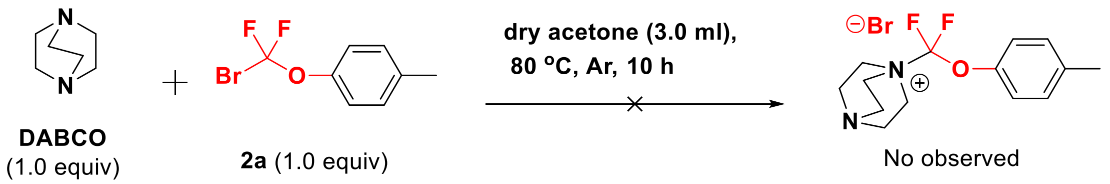

To a dried 10 ml Schlenk-type tube equipped with a magnetic stir bar was charged with DABCO (23 mg, 0.2 mmol, 1.0 equiv) under air. The reaction mixture was then evacuated and backfilled with Ar (3 times). 1-(bromodifluoromethoxy)-4-methylbenzene **2a** (47.0 mg, 0.2mmol, 1.0 equiv), and acetone (3 mL) were added. The mixture was stirred at 80 °C for 10 h. The reaction mixture was cooled to room temperature. No precipitation was observed. The reaction was analysed by ^{19}F NMR, which ^{19}F NMR chemical-shift were same basically between the reaction solution (δ -16.15) and **2a** (δ -16.10)

6. References

[1] A. Graham, G. Doyle. *Org. Lett.* **2012**, *14*, 1616.
[2] M. Kayumov, J.-N. Zhao, S. Mirzaakhmedov, D.-Yu. Wang, A. Zhang. *Advanced Synthesis & Catalysis*.**2020**, *362*, 776.
[3] X.-W. Deng, G. Zhou, X. Han, K. Ullah, R. Srinivasan. *Org. Lett.* **2021**, DOI: 10.1021/acs.orglett.1c00305.
[4] Z. Feng, Q.-Q. Min, X.-P. Fu, L. An, X.-G. Zhang. *Nature Chemistry*. **2017**, *9*, 918.
[5] Y.-L. Xiao, W.-H. Guo, G.-Z. He, Q. Pan, X.-G. Zhang. *Angew. Chem. Int. Ed.* **2014**, *53*, 9909.
[6] D. Mandal, R. Gupta, A. K. Jaiswal, R. D. Young. *J. Am. Chem. Soc.* **2020**, *142*, 2572.
[7] T. Khotavivattana, S. Verhoog, M. Tredwell, L. Pfeifer, S. Calderwood, K. Wheelhouse, T. L. Collier, V. Gouverneur. *Angew. Chem. Int. Ed.* **2015**, *54*, 9991.
[8] F. Toulgoat, B. R. Langlois, M. Médebielle, J. Sanchez. *J. Org. Chem*. **2007**, *72*, 9046.
[9] L.-H Liao, R An, H.-M Li, Y Xu, J.-J Wu, X.-D, Zhao. *Angew. Chem. Int. Ed.* **2020**, *59*, 11010.
[10] M. Zhou, C. Ni, Z.-B. He, J.-B. Hu. *Org. Lett.* **2016**, *18*, 3754.
[11] Y.-L. Xiao, B. Zhang, Z. Feng, X.-G. Zhang. *Org. Lett.* **2014**, *16*, 4822.
[12] J. B. Sap, T. C. Wilson, C. W. Kee, L. Zhang, C. Genicotc, V. Gouverneur. *Chem. Sci.,* **2019***, 10,* 3237.
[13] W. Huang, X.-L. Wan, Q.-L. Shen. *Org. Lett.* **2020***, 22,* 4327.
[14] X. He, X. Gao, X.-G. Zhang. *Chin. J. Chem*. **2018**, *36*, 143.
[15] Y.-Y. Xiao, Q.-Q. Min, C. Xu, R.-W. Wang, X.-G. Zhang. *Angew. Chem. Int. Ed.* **2016**, *55*, 5837.
[16] Y.-M. Su, G.-S. Feng, Z.-Y. Wang, Q. Lan, X.-S. Wang. *Angew. Chem. Int. Ed.* **2015**, *54*, 6003.
[17] J. Guidottia, V. Schanenb, M. Tordeuxa, C. Wakselman. *Journal of Fluorine Chemistry*. **2005**, *126*, 445.
[18] T. J. Doyon, J. C. Perkins, S.A. Dockrey, E. O. Romero, K. C. Skinner, P. M. Zimmerman, A. R. H. Narayan. *J. Am. Chem. Soc.* **2019**, *141*, 20269.
[19] Z.-X. Chen, Y.-W. Jiang, L. Zhang, Y.-L. Guo, D.-W. Ma. *J. Am. Chem. Soc.* **2019**, *141*, 3541.
[20] S. Sato, A. Hirayama, H. Ueda, H. Tokuyama. *Asian J. Org. Chem.* **2017**, *6*, 54.
[21] J. Chen, J.-H. Lin, and J.-C. Xiao. *Chem. Commun.,* **2018**, *54*, 7034.
[22] W.-T. Gu, Z.-L, Song, A. Zhang. *Faming Zhuanli Shenqing*, CN110240587, 17 Sep 2019

7. X-Ray crystallographic data

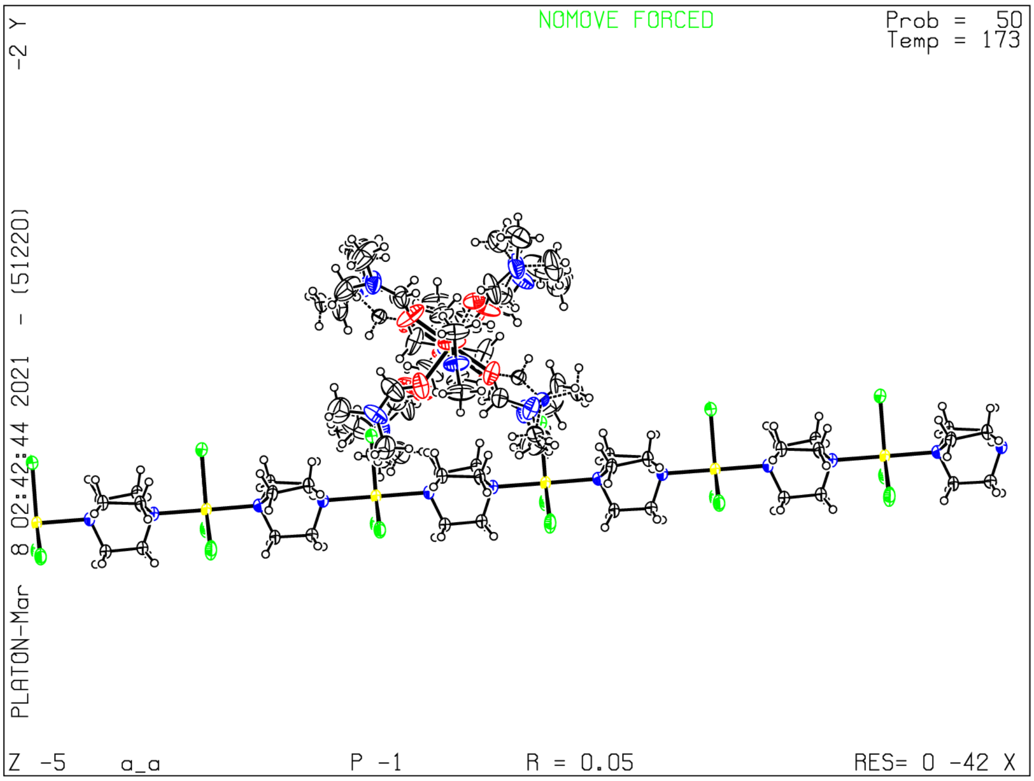

Figure S1. Ortep representation of **Ni-11**

Table S8. Crystal data and structure refinement details for **Ni-11**

**CCDC 2095880**	
Identification code	a_a
Empirical formula	C30H51Br6N10Ni3O5
Formula weight	1294.45
Temperature/K	173.0
Crystal system	triclinic
Space group	P-1
a/Å	13.9596(4)
b/Å	14.9091(5)
c/Å	15.0915(5)
α/°	99.812(2)
β/°	103.0370(10)
γ/°	103.820(2)
Volume/Å3	2886.65(16)
Z	2
ρcalcg/cm3	1.489
μ/mm-1	6.309
F(000)	1284.0

Crystal size/mm3	? × ? × ?
Radiation	CuKα (λ = 1.54178)
2Θ range for data collection/°	6.188 to 137.014
Index ranges	-16 ≤ h ≤ 16, -17 ≤ k ≤ 17, -16 ≤ l ≤ 18
Reflections collected	62461
Independent reflections	10599 [Rint = 0.0499, Rsigma = 0.0330]
Data/restraints/parameters	10599/144/598
Goodness-of-fit on F2	1.070
Final R indexes [I>=2σ (I)]	R1 = 0.0505, wR2 = 0.1341
Final R indexes [all data]	R1 = 0.0581, wR2 = 0.1392
Largest diff. peak/hole / e Å-3	1.59/-1.10

8. Gating strategy

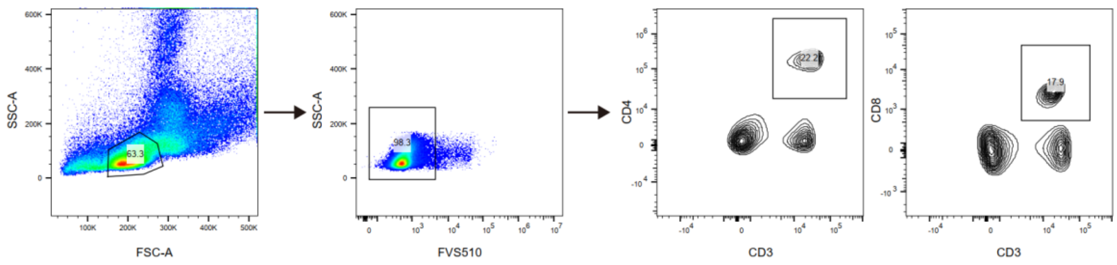

Figure S2. The gating strategy of $CD4^+$ and $CD8^+$ T cells from tumors and spleens.
