# Supplementary material for: Synthesis of aryldifluoromethyl aryl ethers via nickel-catalyzed suzuki cross-coupling between aryloxydifluoromethyl bromides and boronic acids
Source: Commun Chem. 2022 Jul 4;5:78. doi: 10.1038/s42004-022-00694-4 (PMC9814959; doi:10.1038/s42004-022-00694-4)
Supplement: Supplementary file 2 — Reporting Summary [file 42004_2022_694_MOESM2_ESM.pdf]

nature portfolio

Corresponding author(s): Ruo-xuan Xiao

Last updated by author(s): Jun 17, 2022

Reporting Summary

Nature Portfolio wishes to improve the reproducibility of the work that we publish. This form provides structure for consistency and transparency in reporting. For further information on Nature Portfolio policies, see our Editorial Policies and the Editorial Policy Checklist.

Statistics

For all statistical analyses, confirm that the following items are present in the figure legend, table legend, main text, or Methods section.

n/a	Confirmed	
☐	☒	The exact sample size (n) for each experimental group/condition, given as a discrete number and unit of measurement
☒	☐	A statement on whether measurements were taken from distinct samples or whether the same sample was measured repeatedly
☒	☐	The statistical test(s) used AND whether they are one- or two-sided<br>*Only common tests should be described solely by name; describe more complex techniques in the Methods section.*
☒	☐	A description of all covariates tested
☒	☐	A description of any assumptions or corrections, such as tests of normality and adjustment for multiple comparisons
☒	☐	A full description of the statistical parameters including central tendency (e.g. means) or other basic estimates (e.g. regression coefficient) AND variation (e.g. standard deviation) or associated estimates of uncertainty (e.g. confidence intervals)
☒	☐	For null hypothesis testing, the test statistic (e.g. F, t, r) with confidence intervals, effect sizes, degrees of freedom and P value noted<br>*Give P values as exact values whenever suitable.*
☒	☐	For Bayesian analysis, information on the choice of priors and Markov chain Monte Carlo settings
☒	☐	For hierarchical and complex designs, identification of the appropriate level for tests and full reporting of outcomes
☒	☐	Estimates of effect sizes (e.g. Cohen's d, Pearson's r), indicating how they were calculated

Our web collection on statistics for biologists contains articles on many of the points above.

Software and code

Policy information about availability of computer code

Data collection	The FLOW data were collected from CytoFLEX flow cytometry (BECKMAN COULTER).
Data analysis	GraphPad Prism 9 was used for data analysis.

For manuscripts utilizing custom algorithms or software that are central to the research but not yet described in published literature, software must be made available to editors and reviewers. We strongly encourage code deposition in a community repository (e.g. GitHub). See the Nature Portfolio guidelines for submitting code & software for further information.

Data

Policy information about availability of data

All manuscripts must include a data availability statement. This statement should provide the following information, where applicable:

- Accession codes, unique identifiers, or web links for publicly available datasets
- A description of any restrictions on data availability
- For clinical datasets or third party data, please ensure that the statement adheres to our policy

N/A

nature portfolio | reporting summary

March 2021

Human research participants

Policy information about studies involving human research participants and Sex and Gender in Research.

Reporting on sex and gender	N/A
Population characteristics	N/A
Recruitment	N/A
Ethics oversight	N/A

Note that full information on the approval of the study protocol must also be provided in the manuscript.

Field-specific reporting

Please select the one below that is the best fit for your research. If you are not sure, read the appropriate sections before making your selection.

☒ Life sciences ☐ Behavioural & social sciences ☐ Ecological, evolutionary & environmental sciences

For a reference copy of the document with all sections, see nature.com/documents/nr-reporting-summary-flat.pdf

Life sciences study design

All studies must disclose on these points even when the disclosure is negative.

Sample size	No methods were used to predetermine samples size.
Data exclusions	No data were excluded from the analysis.
Replication	The number of replications are indicated in legends and methods.
Randomization	Mice were randomly allocated into experimental groups.
Blinding	In the animal studies, blinding was not possible for the administrator owing to practical reasons, but the appropriate controls were present.

Reporting for specific materials, systems and methods

We require information from authors about some types of materials, experimental systems and methods used in many studies. Here, indicate whether each material, system or method listed is relevant to your study. If you are not sure if a list item applies to your research, read the appropriate section before selecting a response.

Materials & experimental systems

n/a	Involved in the study	
☐	☒	Antibodies
☐	☒	Eukaryotic cell lines
☒	☐	Palaeontology and archaeology
☐	☒	Animals and other organisms
☒	☐	Clinical data
☒	☐	Dual use research of concern

Methods

n/a	Involved in the study	
☒	☐	ChIP-seq
☐	☒	Flow cytometry
☒	☐	MRI-based neuroimaging

Antibodies

Antibodies used	Purified Rat Anti-Mouse CD16/CD32 (BD Pharmingen, 553141)<br>anti-mouse CD3e PerCP-Cy5.5 (BD, 561108)<br>anti-mouse CD8a APC (BD,561093)<br>anti-mouse CD4 PE (BD, 561829)
Validation	All antibodies are commercially available. Each antibody was validated for species and application according to the manufacturer's website.

Eukaryotic cell lines

Policy information about cell lines and Sex and Gender in Research

Cell line source(s)	The MC38 cells were kindly provided by Deng L.F.(Sci Transl Med. 2020 Jun 24;12(549):eaay9013.)
Authentication	N/A
Mycoplasma contamination	The cell lines used in this study were free of mycoplasma contamination, according to results from the MycAway™ -Color One-Step Mycoplasma Detection Kit (Yeasen)
Commonly misidentified lines (See ICLAC register)	N/A

Animals and other research organisms

Policy information about studies involving animals; ARRIVE guidelines recommended for reporting animal research, and Sex and Gender in Research

Laboratory animals	6-week-old female mice were purchased from Shanghai SLAC Laboratory Animal Co.,Ltd.
Wild animals	N/A
Reporting on sex	*Indicate if findings apply to only one sex; describe whether sex was considered in study design, methods used for assigning sex. Provide data disaggregated for sex where this information has been collected in the source data as appropriate; provide overall numbers in this Reporting Summary. Please state if this information has not been collected. Report sex-based analyses where performed, justify reasons for lack of sex-based analysis.*
Field-collected samples	N/A
Ethics oversight	The animal experimental protocols were approved by the Instructional Animal Care and Use Committee of Shanghai Jiao Tong University.

Note that full information on the approval of the study protocol must also be provided in the manuscript.

Flow Cytometry

Plots

Confirm that:

☒ The axis labels state the marker and fluorochrome used (e.g. CD4-FITC).

☒ The axis scales are clearly visible. Include numbers along axes only for bottom left plot of group (a 'group' is an analysis of identical markers).

☒ All plots are contour plots with outliers or pseudocolor plots.

☒ A numerical value for number of cells or percentage (with statistics) is provided.

Methodology

Sample preparation	Tumor tissues were minced and digested in 1mg/mL hyaluronidase, 1mg/mL collagenase IV and 0.15mg/mL DNase I for 2 hours and filtered with 70 μm strainer. Mononuclear cells were isolated with Ficoll-Paque PREMIUM 1.073 (Cytiva) as the manufacturer's instruction, and blocked with Purified Rat Anti-Mouse CD16/CD32 (BD Pharmingen) for 1 hours at 4 ℃. After staining with Fixable Viability Stain 520, anti-mouse CD3e PerCP-Cy5.5, anti-mouse CD8a APC and anti-mouse CD4 PE (BD Pharmingen) for 30 min at 4 ℃, the cells were washed and analyzed.
Instrument	CytoFLEX flow cytometry (BECKMAN COULTER)
Software	FlowJo 10.6.2
Cell population abundance	We collect 500,000 cells / sample, of which the percentages of live cells are 80% and 98% in tumor and spleen tissues respectively.
Gating strategy	The gating strategy has shown in supplementary information.

☒ Tick this box to confirm that a figure exemplifying the gating strategy is provided in the Supplementary Information.
