# Supplementary material for: Synthesis of aryldifluoromethyl aryl ethers via nickel-catalyzed suzuki cross-coupling between aryloxydifluoromethyl bromides and boronic acids
Source: Commun Chem. 2022 Jul 4;5:78. doi: 10.1038/s42004-022-00694-4 (PMC9814959; doi:10.1038/s42004-022-00694-4)
Supplement: Supplementary file 4 — Supplementary Data 2 [file 42004_2022_694_MOESM4_ESM.pdf]

1. Copies of NMR spectra data

^{1}H, ^{13}C and ^{19}F NMR spectra of compound 2c

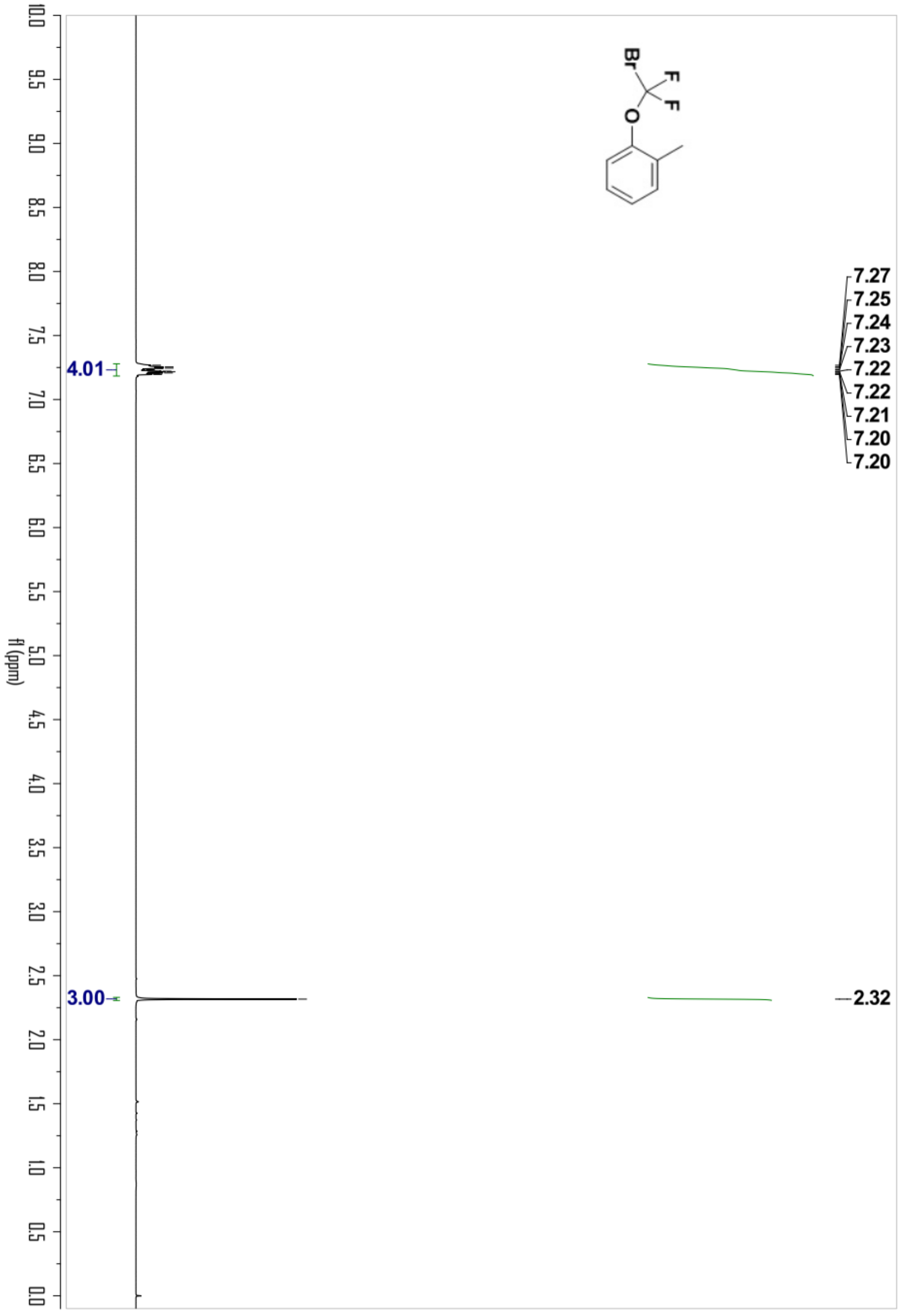

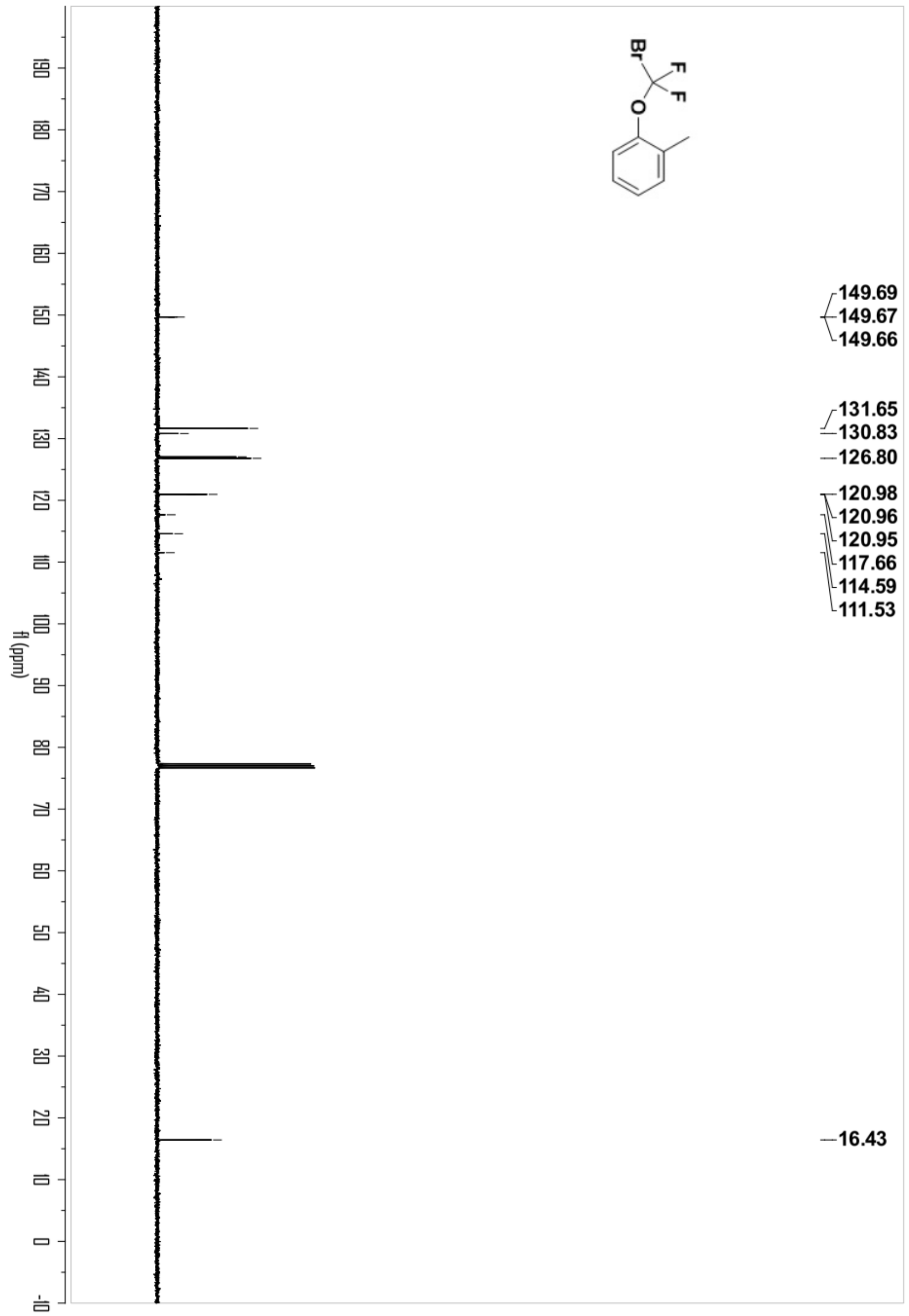
Br
F
F
O
149.69
149.67
149.66
131.65
130.83
126.80
120.98
120.96
120.95
117.66
114.59
111.53
16.43
f1 (ppm)
190
180
170
160
150
140
130
120
110
100
90
80
70
60
50
40
30
20
10
0
-10

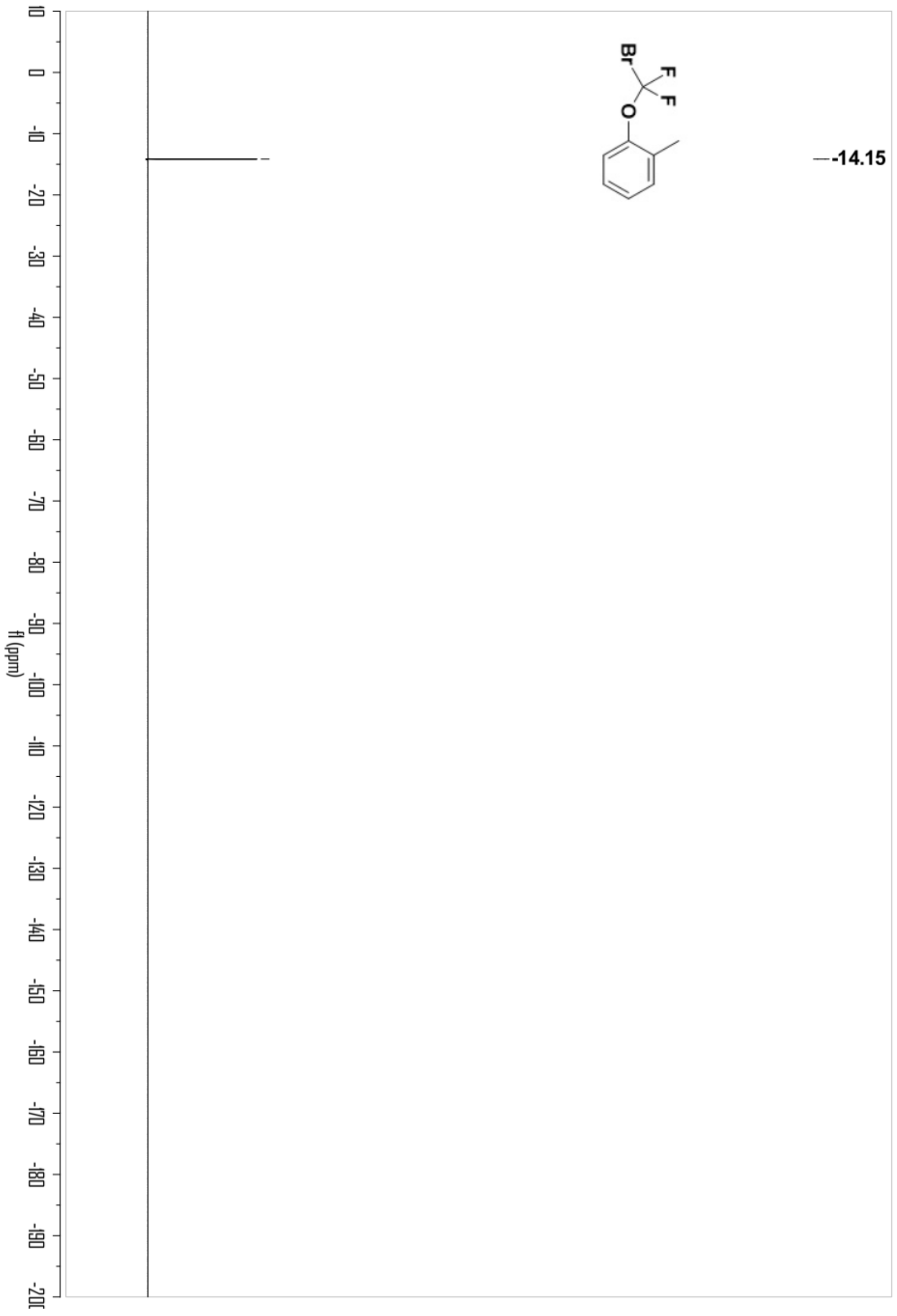
Br
F
F
O
-14.15
f1 (ppm)

^{1}H, ^{13}C and ^{19}F NMR spectra of compound 2e

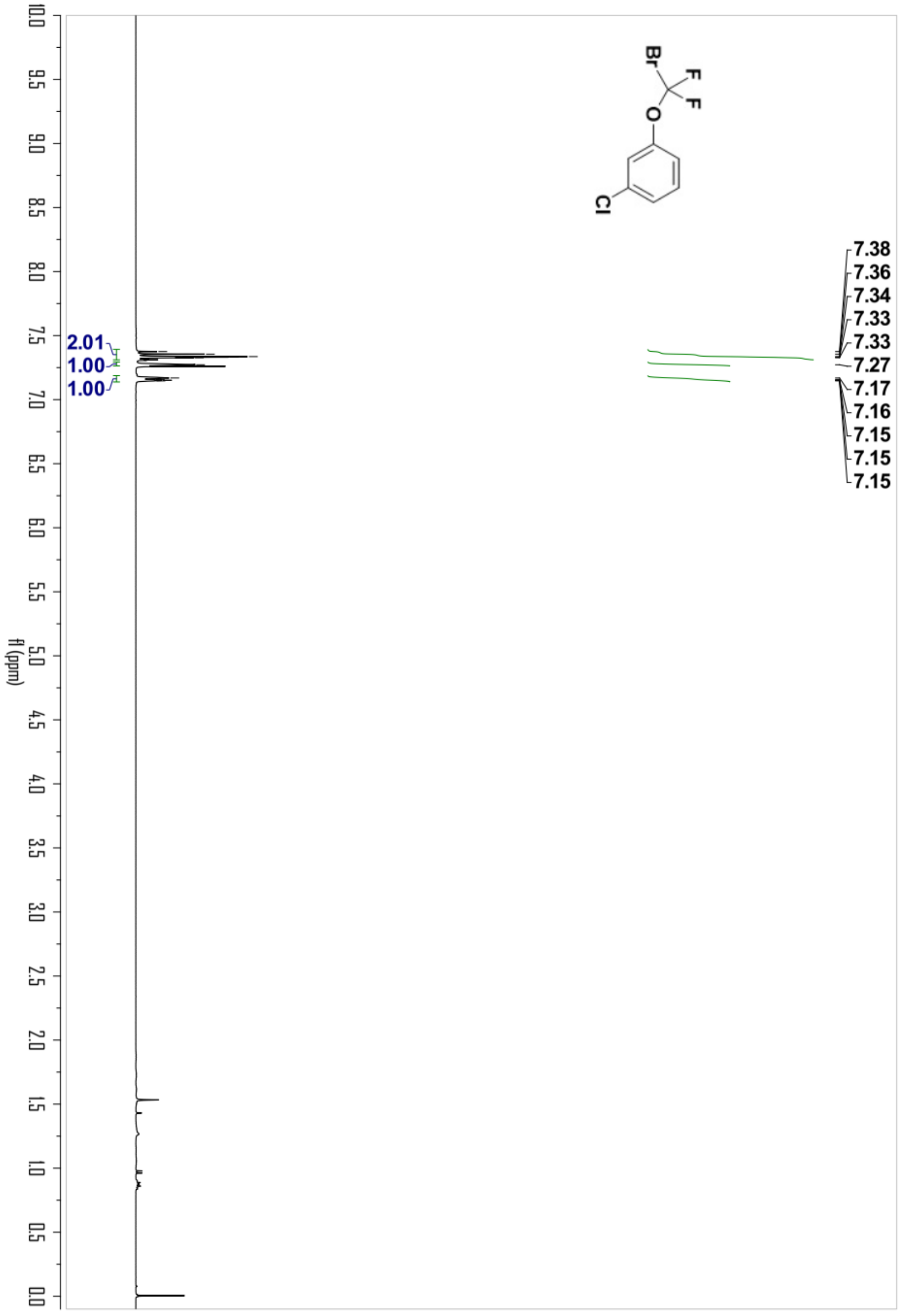

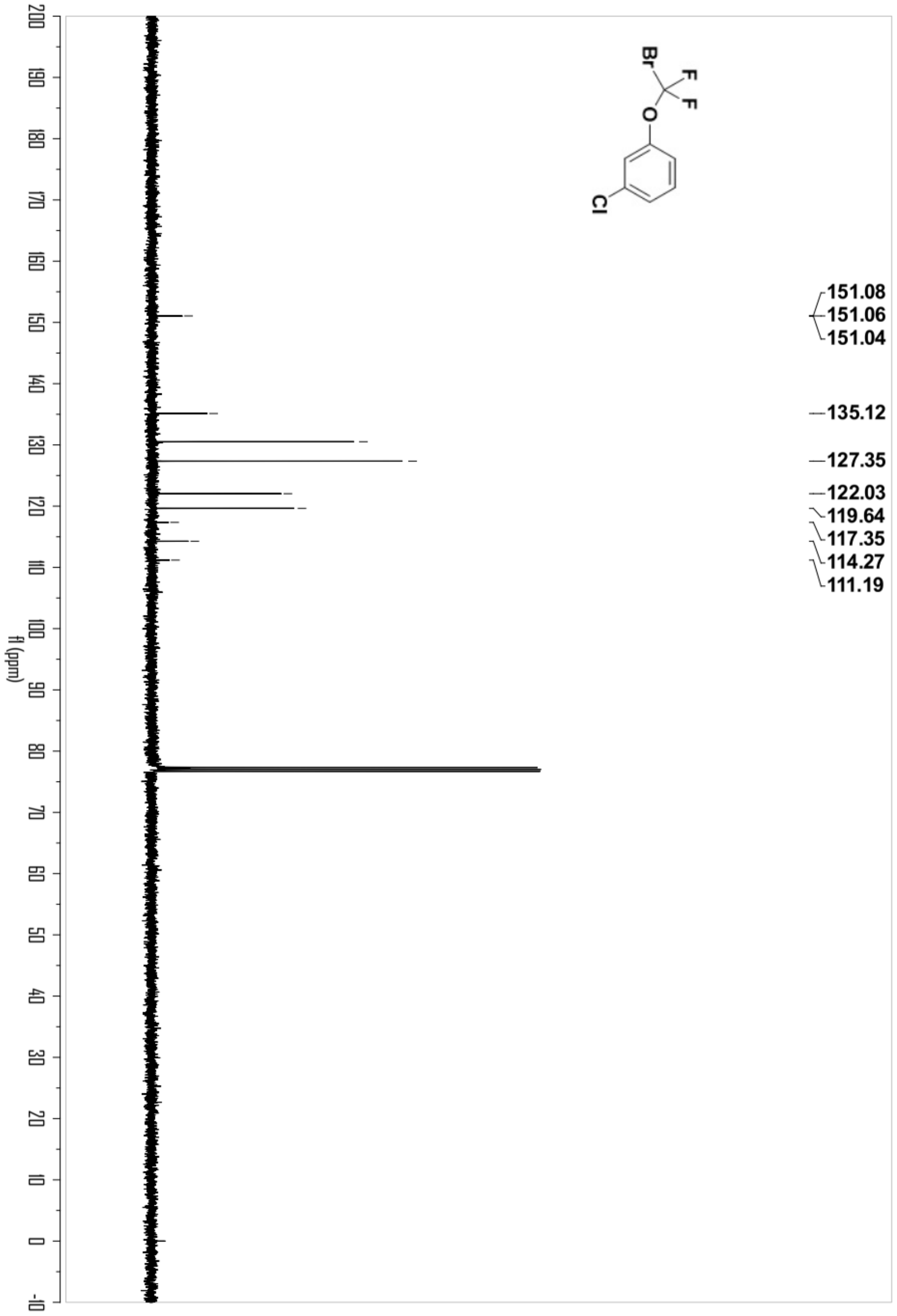
Br
F
F
O
Cl
151.08
151.06
151.04
135.12
127.35
122.03
119.64
117.35
114.27
111.19
f1 (ppm)

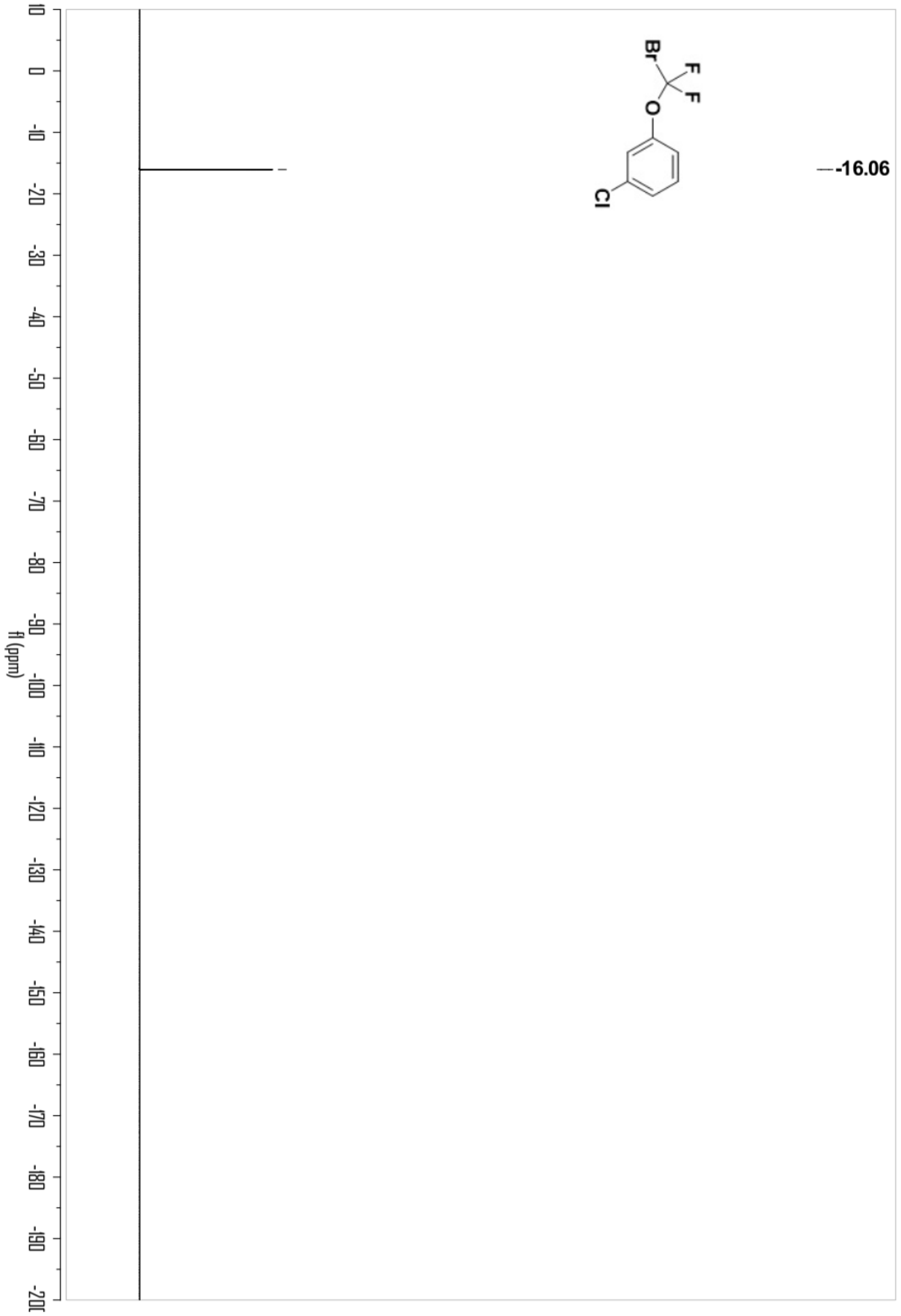
-16.06
f1 (ppm)
Br
F
F
O
Cl

^{1}H, ^{13}C and ^{19}F NMR spectra of compound 2Cf

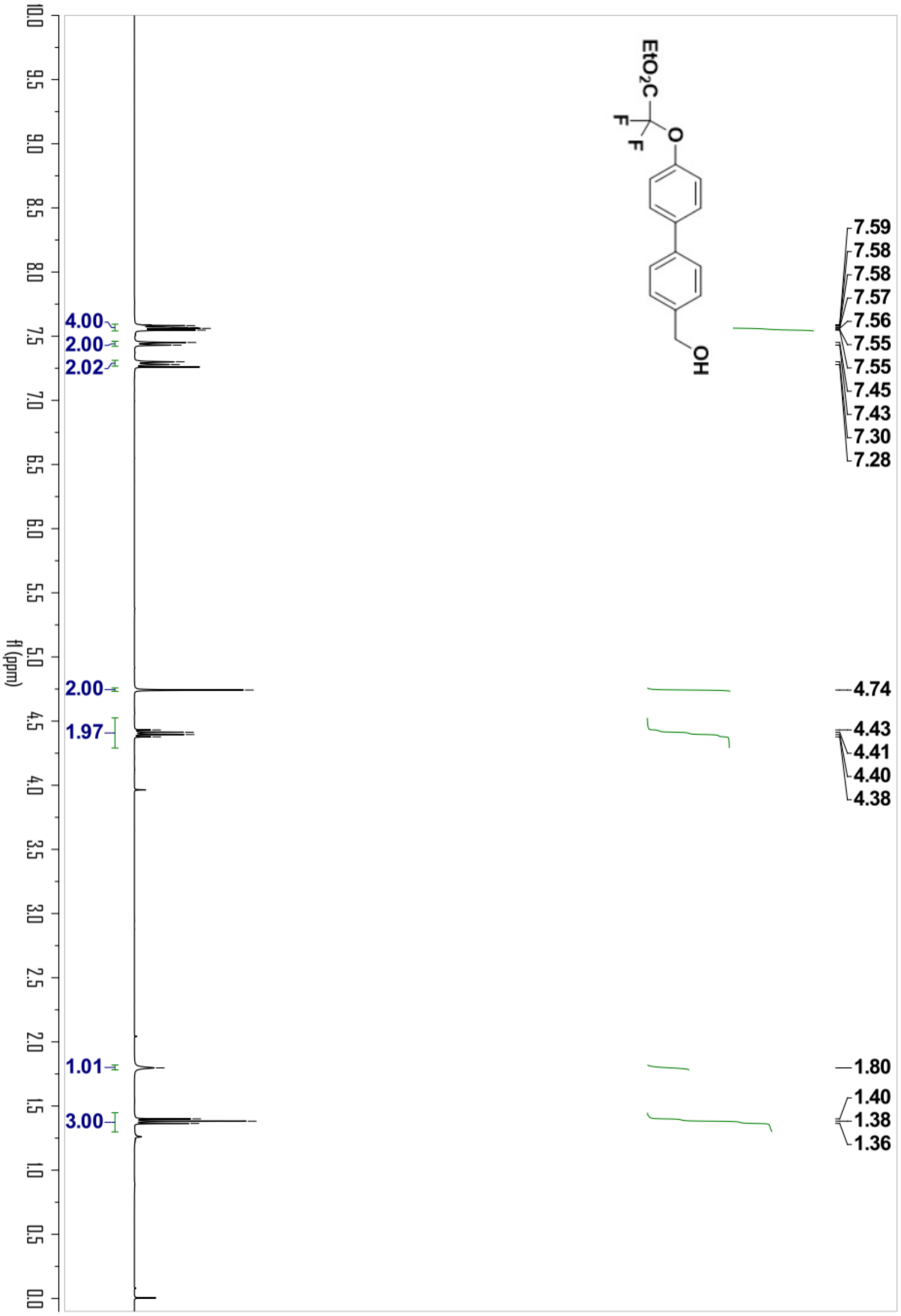

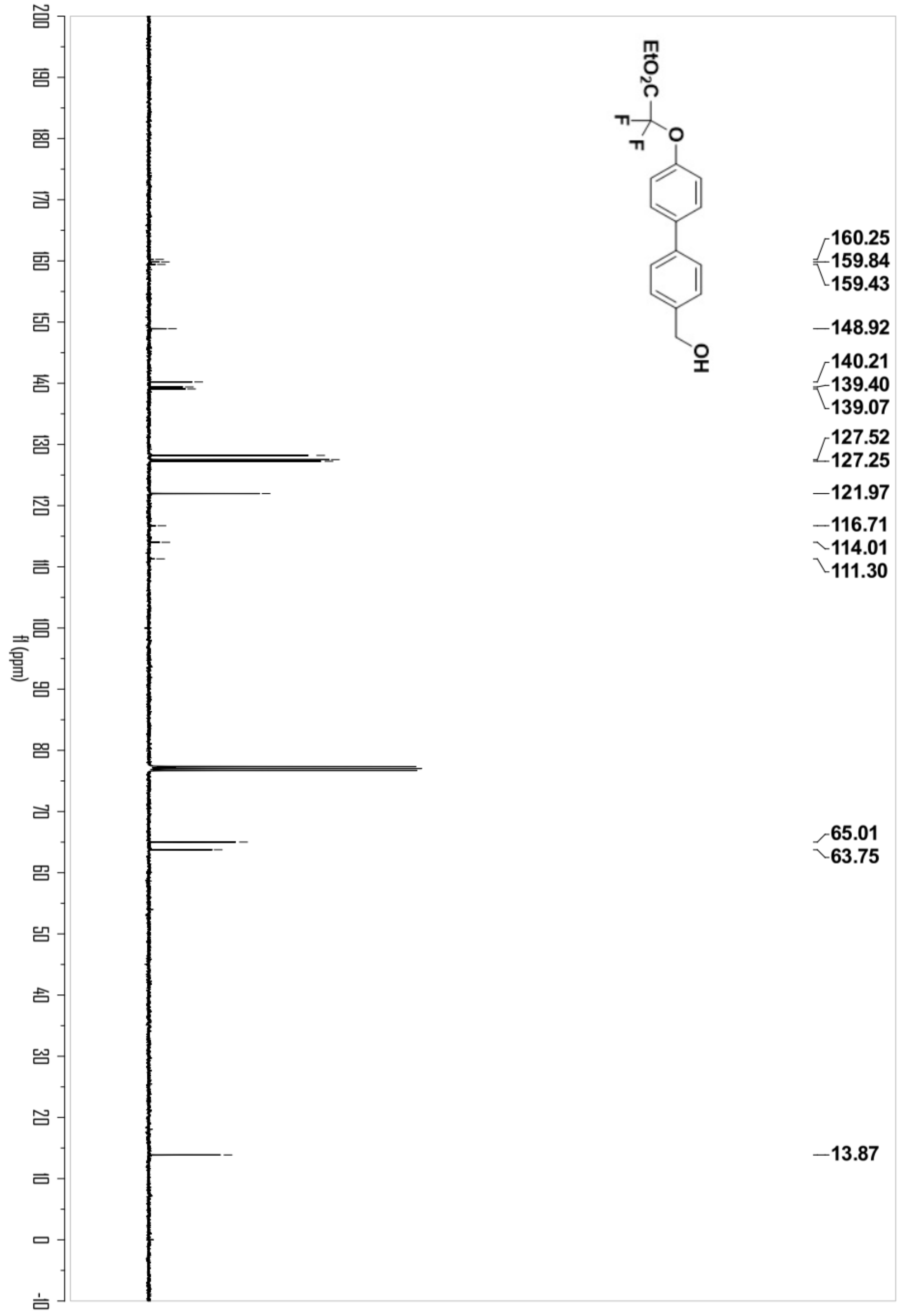

EtO_2C
F
F
O
OH
160.25
159.84
159.43
148.92
140.21
139.40
139.07
127.52
127.25
121.97
116.71
114.01
111.30
65.01
63.75
13.87
f1 (ppm)

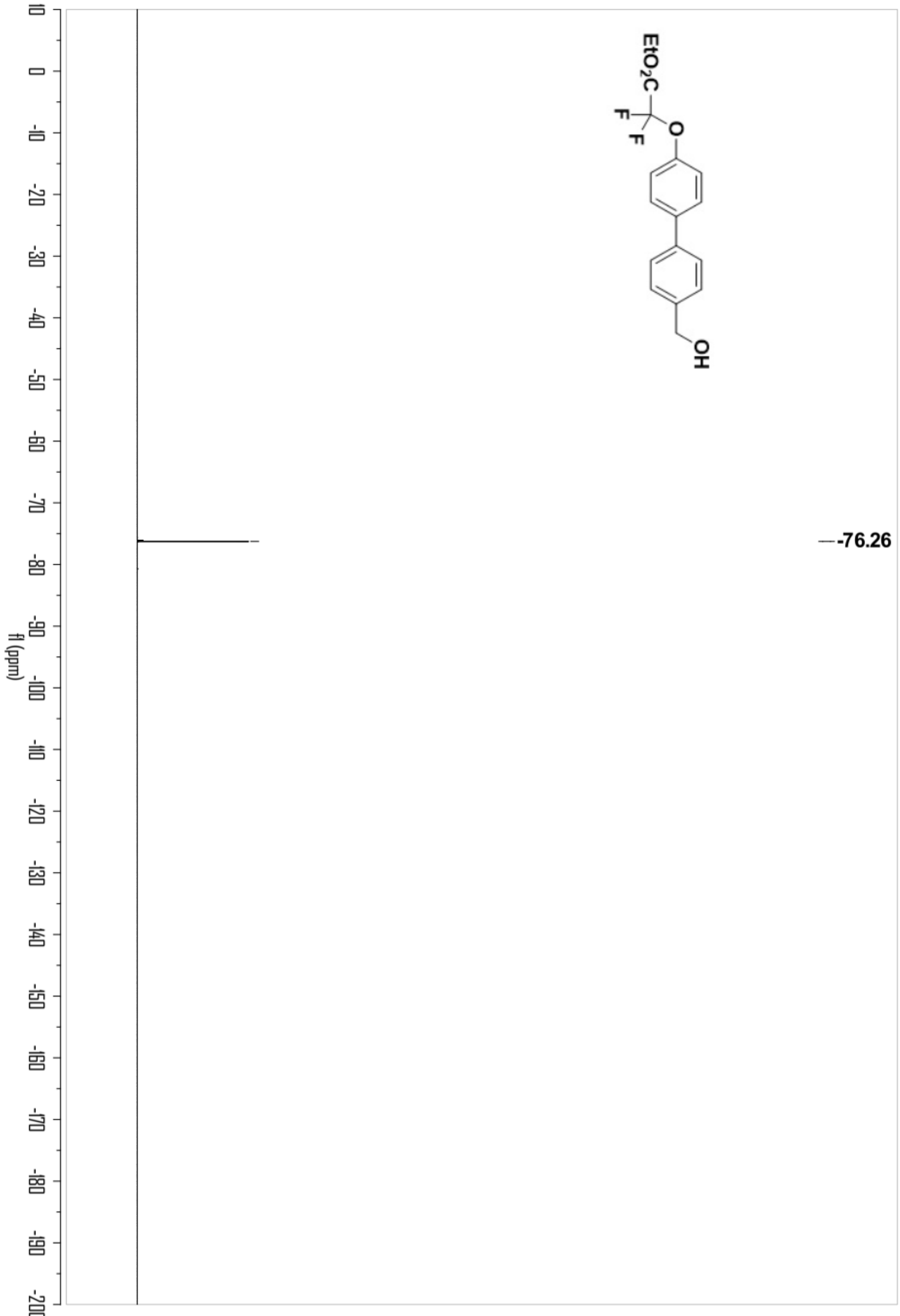
-76.26
f1 (ppm)
EtO2C
F
F
O
OH

^{1}H, ^{13}C and ^{19}F NMR spectra of compound 2Df

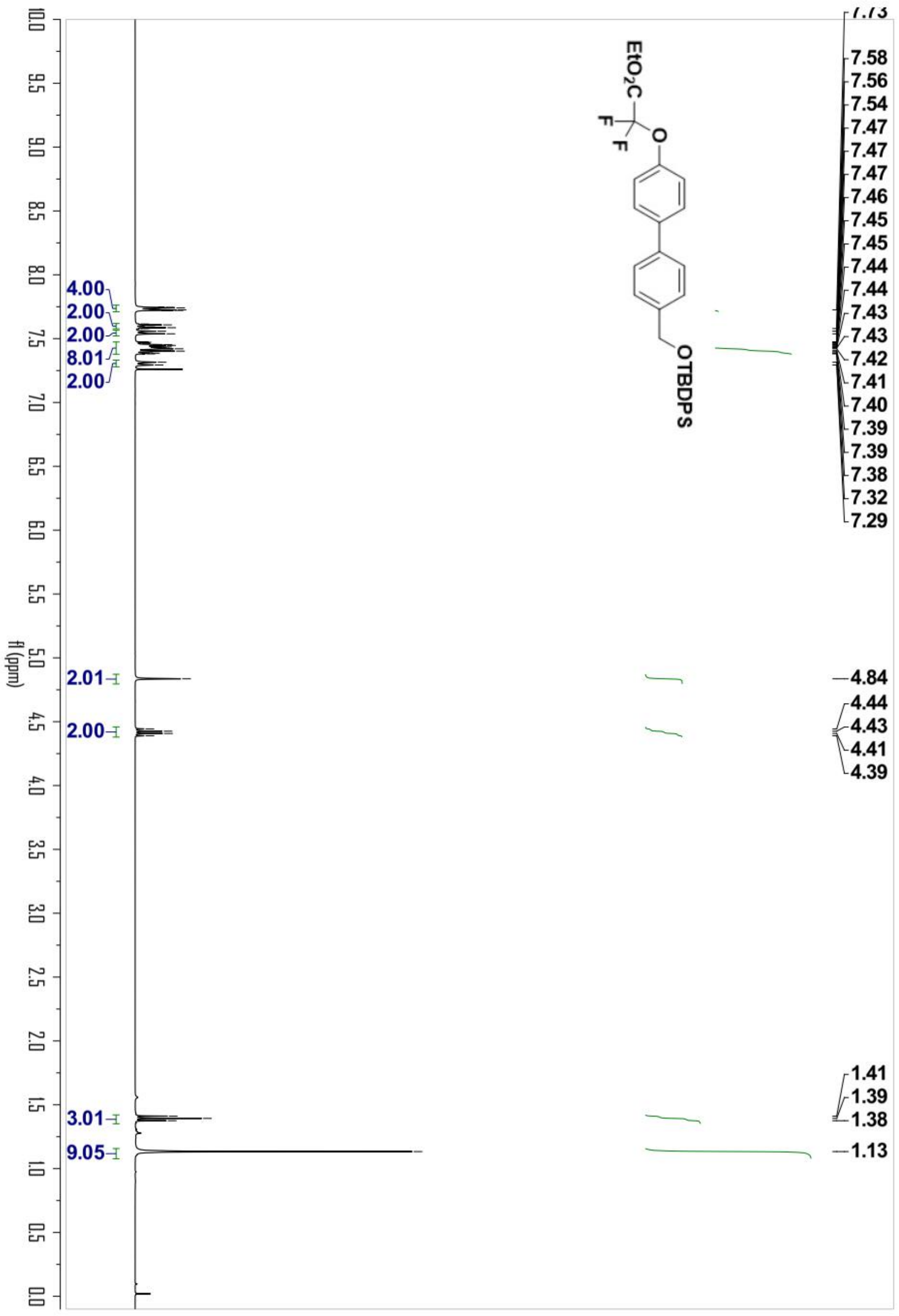

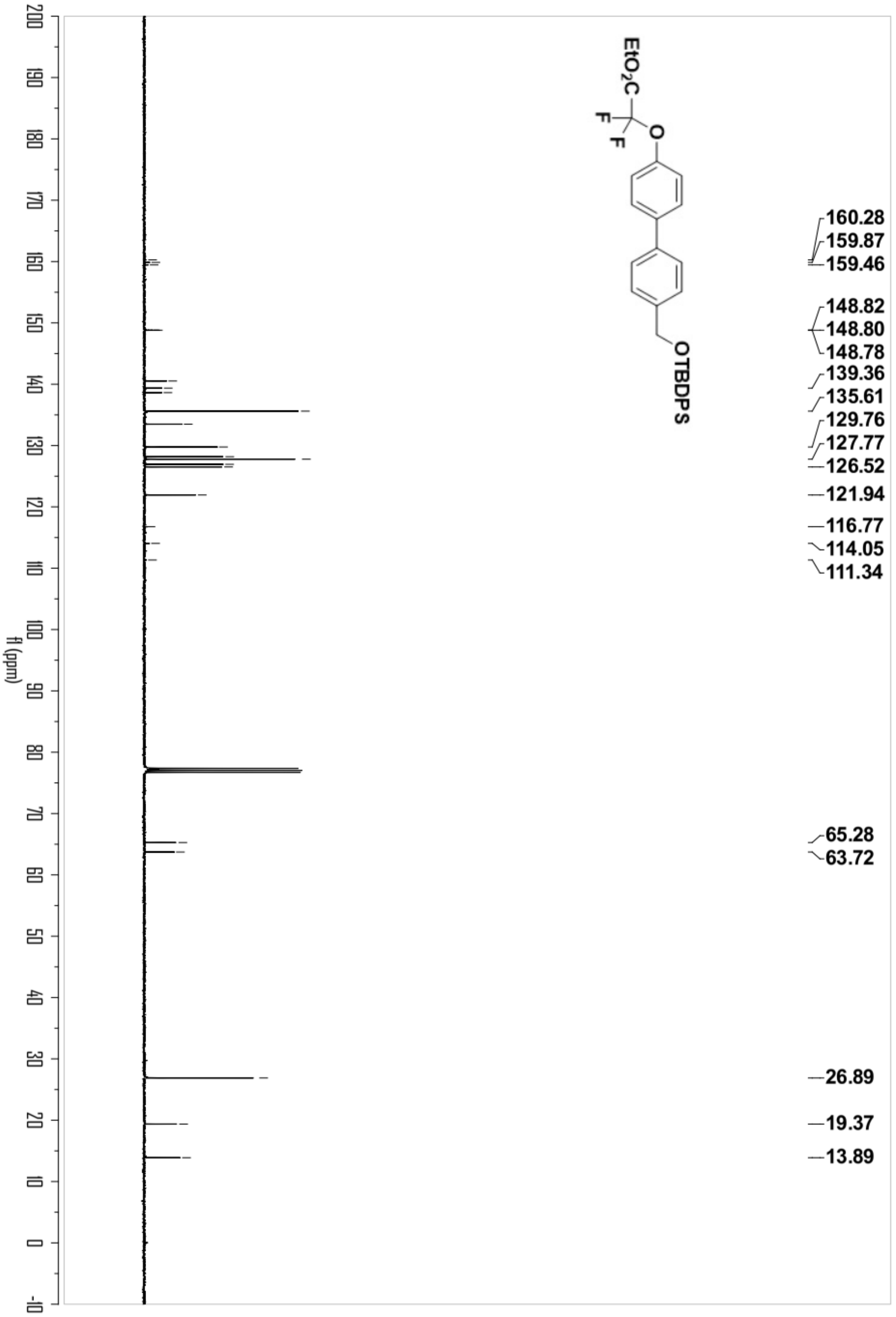

EtO2C
F
F
O
OTBDPS
160.28
159.87
159.46
148.82
148.80
148.78
139.36
135.61
129.76
127.77
126.52
121.94
116.77
114.05
111.34
65.28
63.72
26.89
19.37
13.89
f1 (ppm)

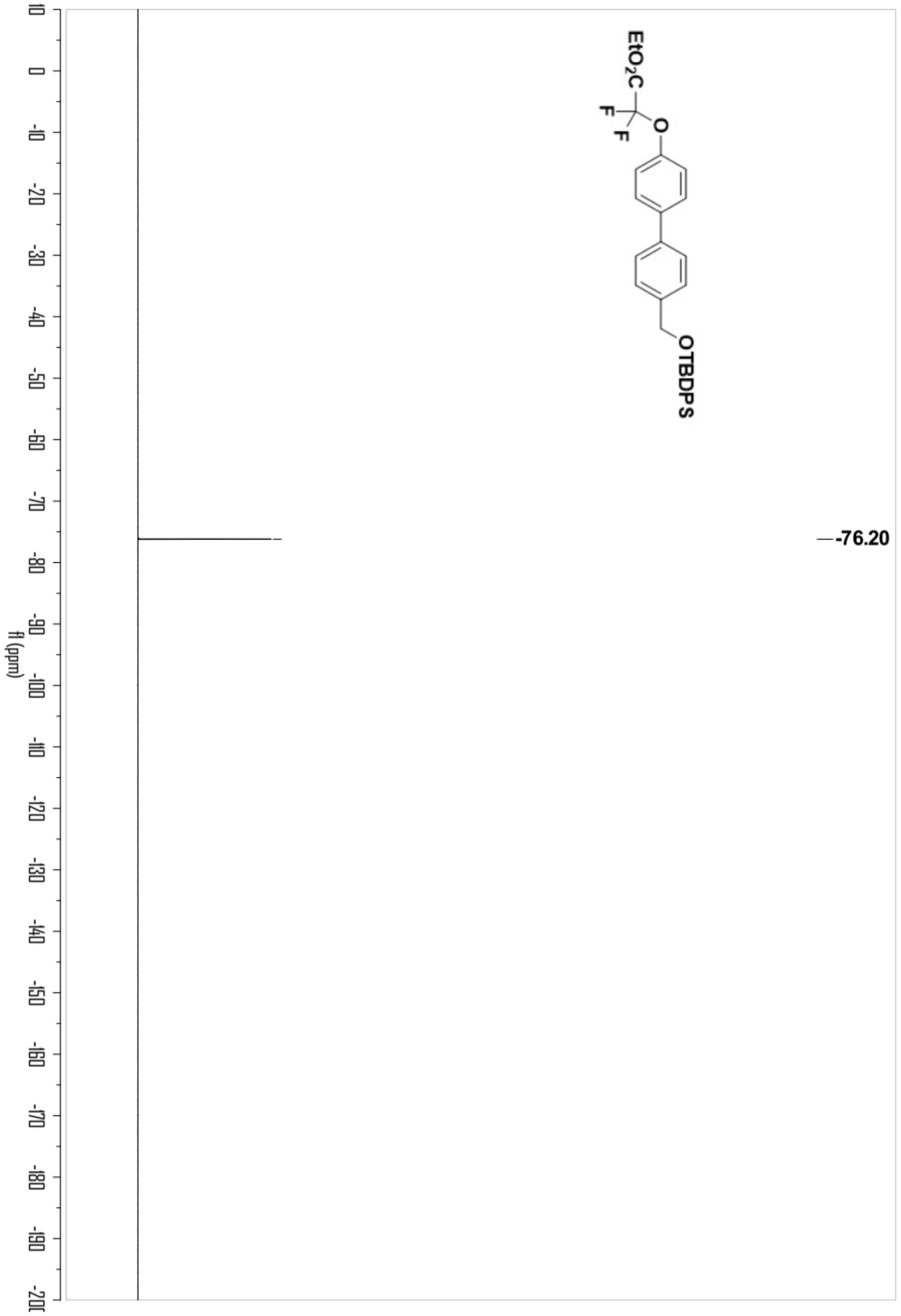
EtO2C
F F
O
OTBDPS
-76.20
f1 (ppm)

^{1}H, ^{13}C and ^{19}F NMR spectra of compound 2Ef

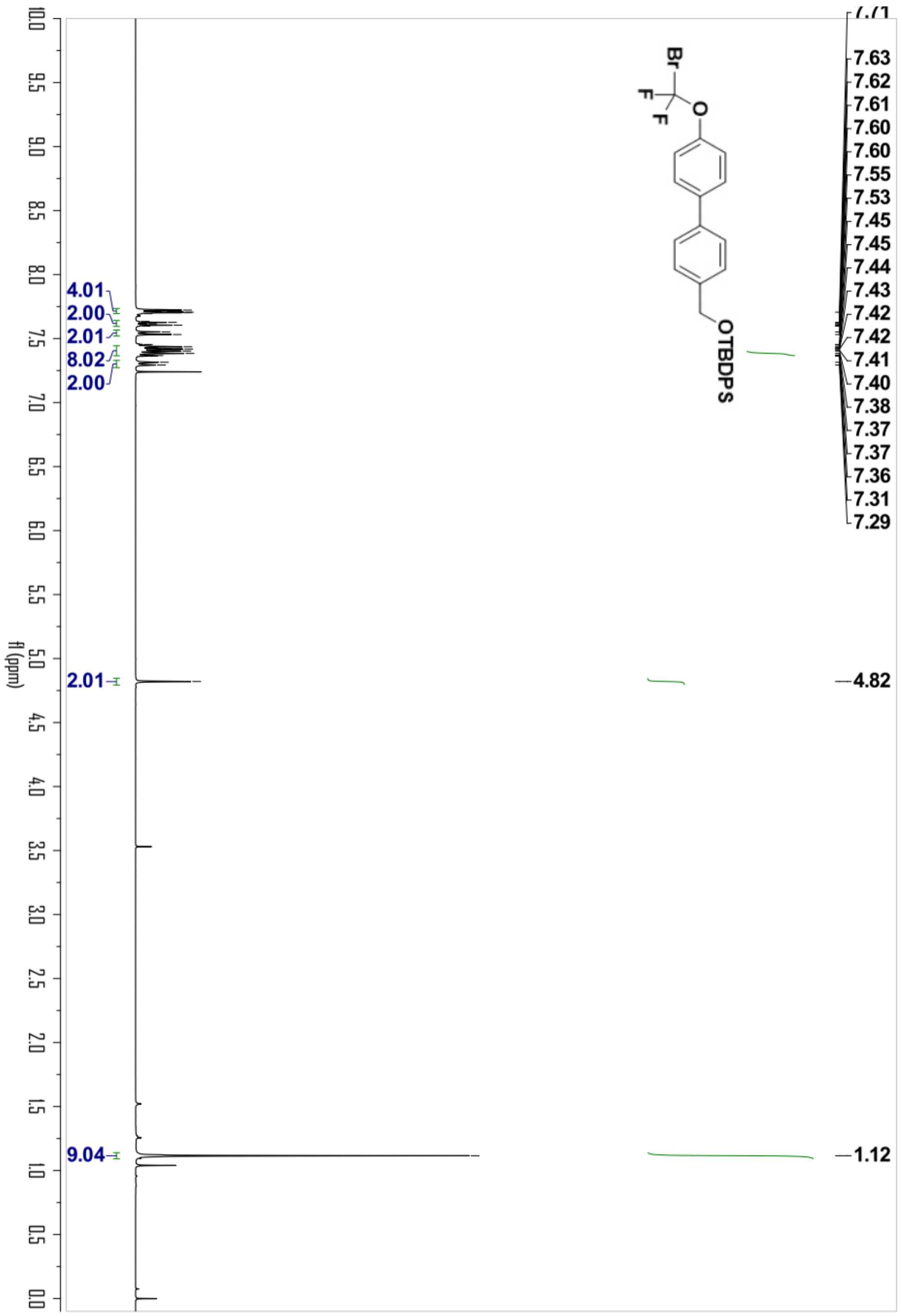

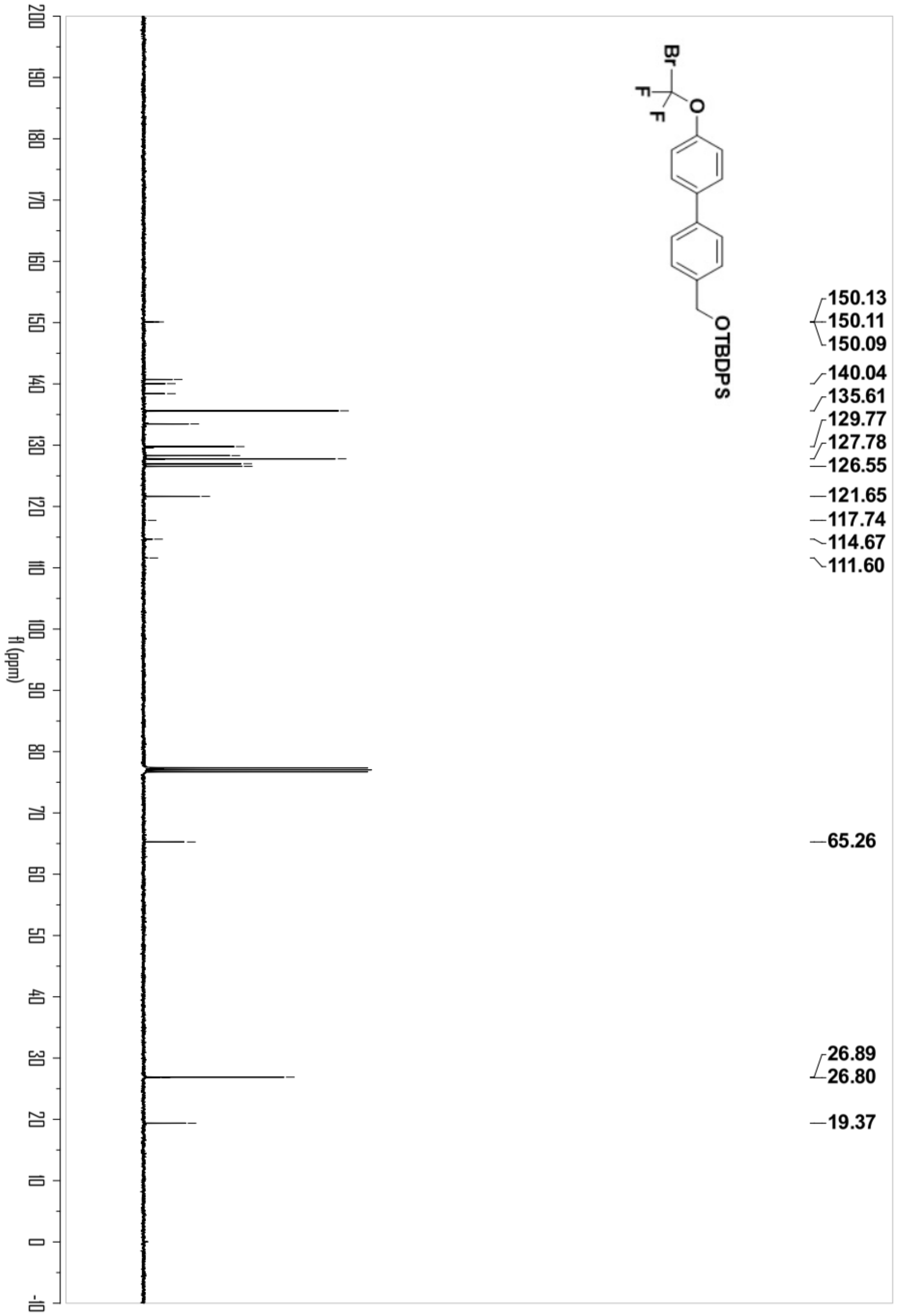

Br
F
F
O
OTBDPS
150.13
150.11
150.09
140.04
135.61
129.77
127.78
126.55
121.65
117.74
114.67
111.60
65.26
26.89
26.80
19.37
f1 (ppm)

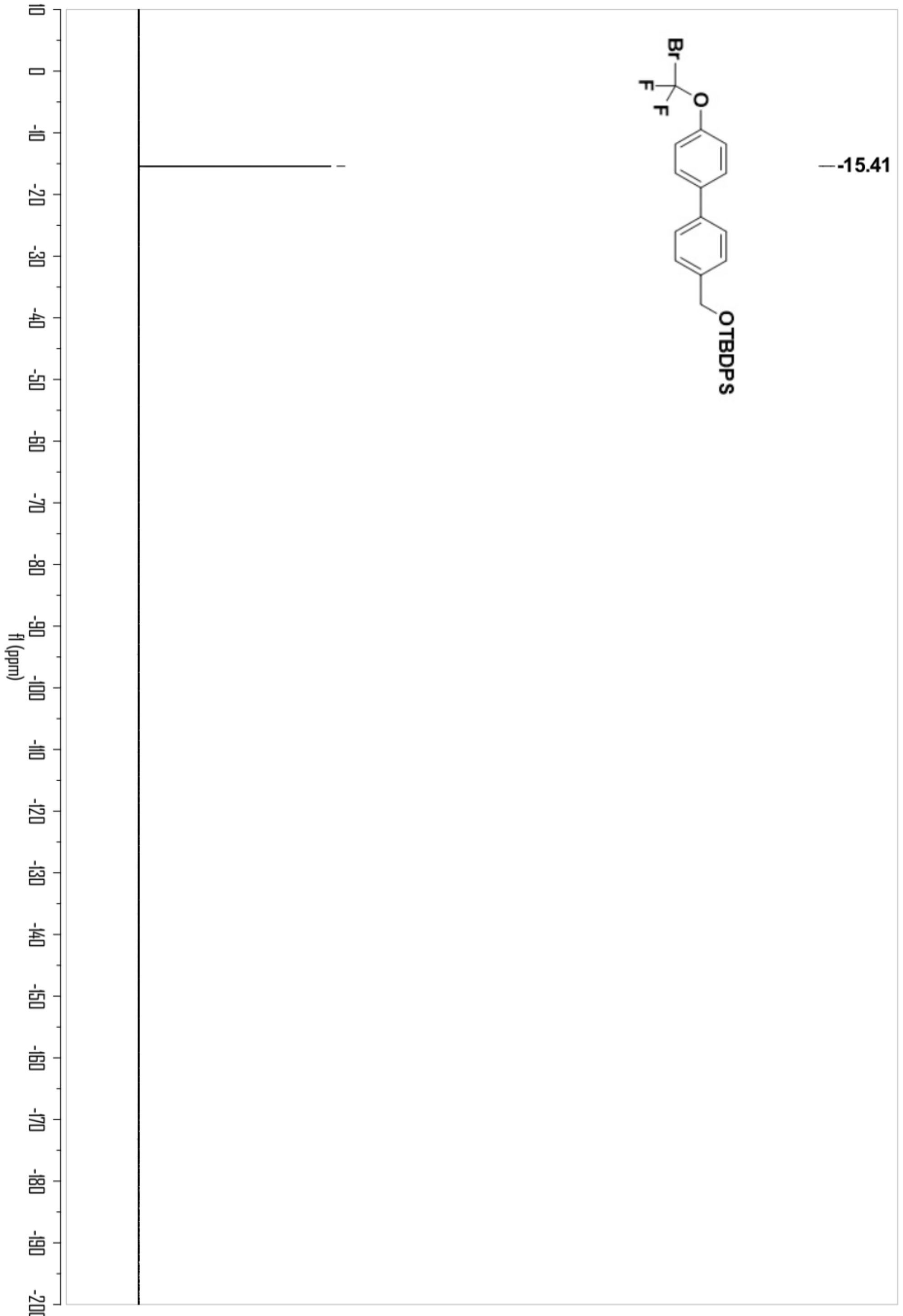
-15.41
Br
F
F
O
OTBDPS
f1 (ppm)

^{1}H, ^{13}C and ^{19}F NMR spectra of compound 2f

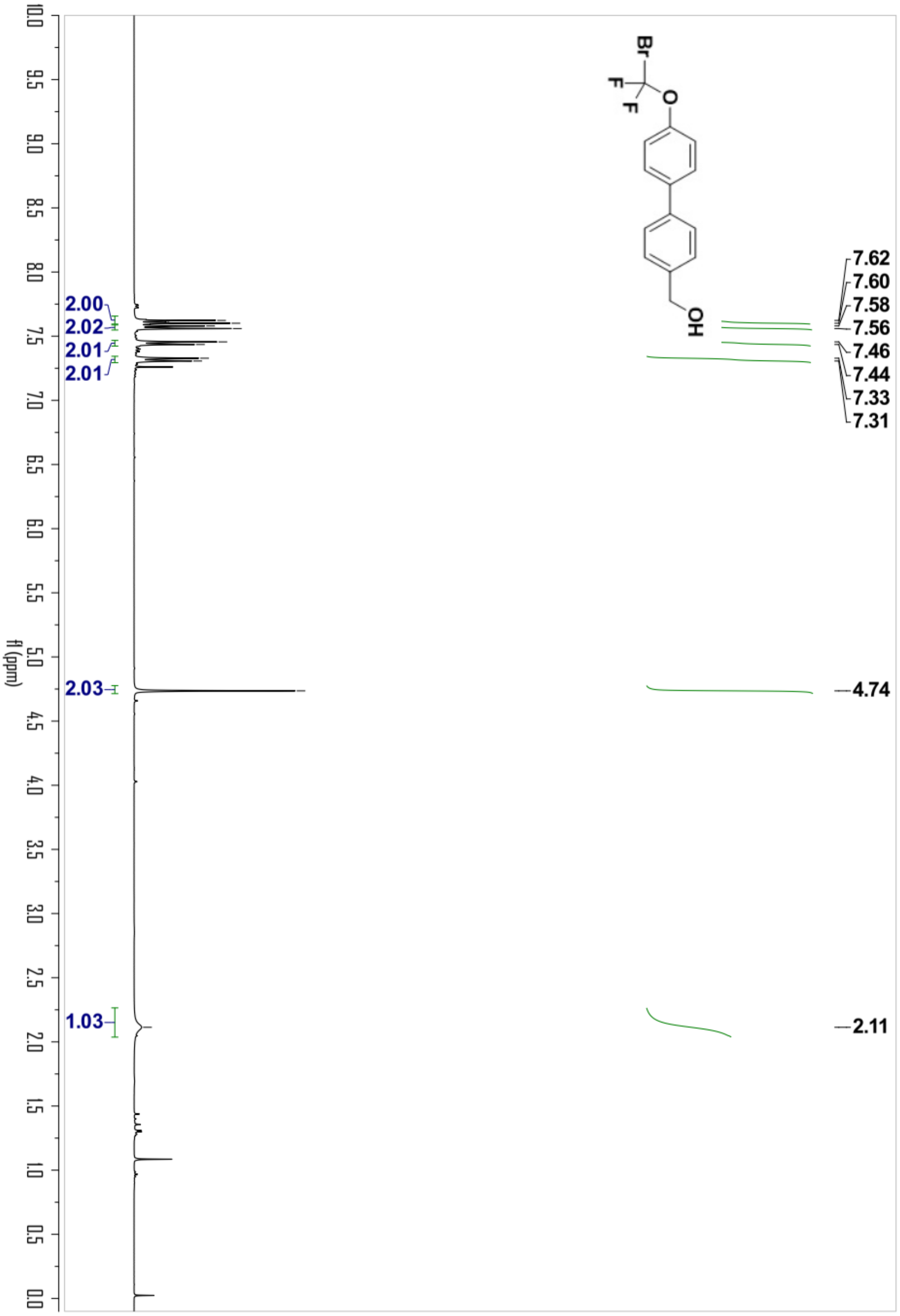

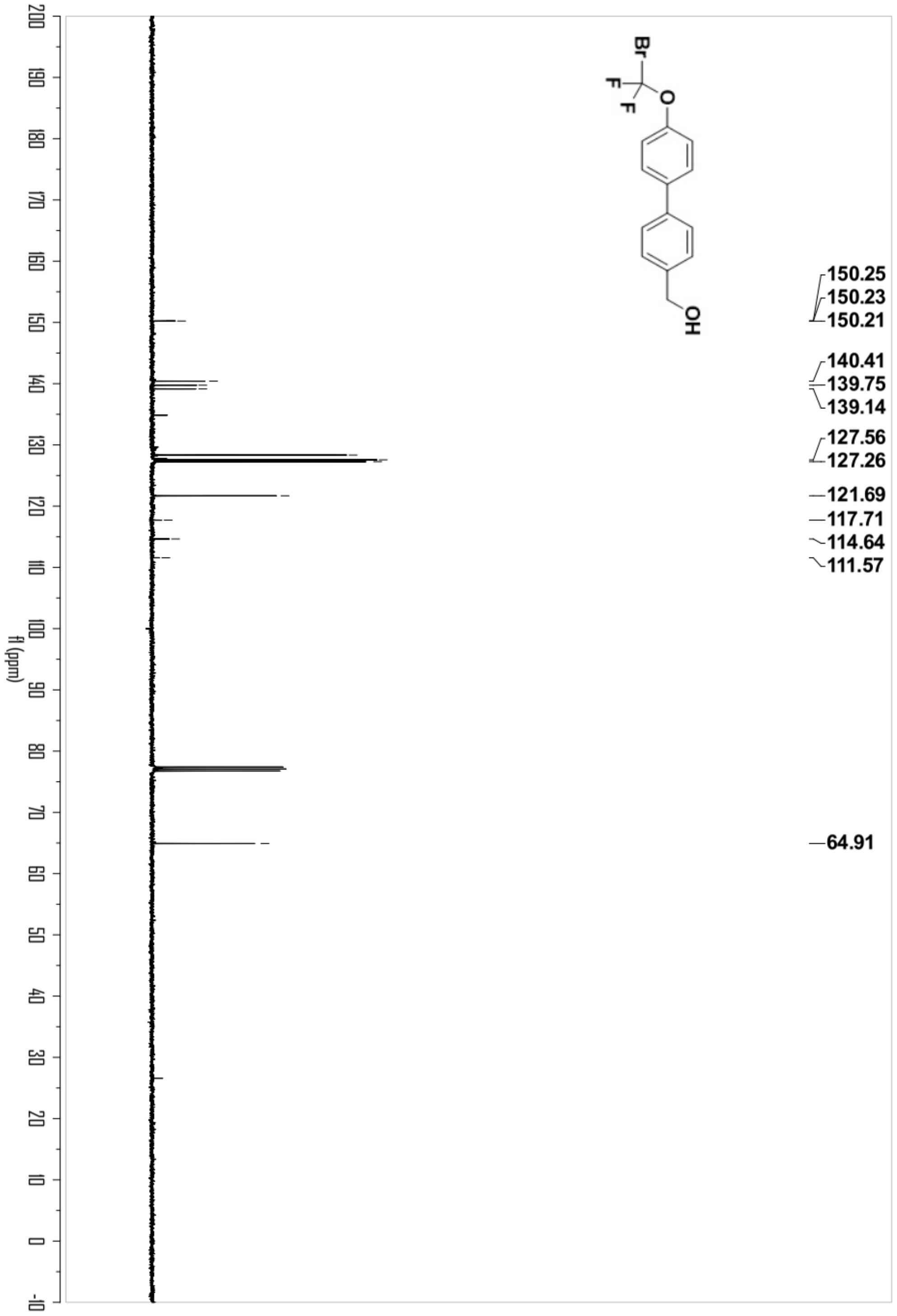
150.25
150.23
150.21
140.41
139.75
139.14
127.56
127.26
121.69
117.71
114.64
111.57
64.91
f1 (ppm)
OH
Br
F
F
O

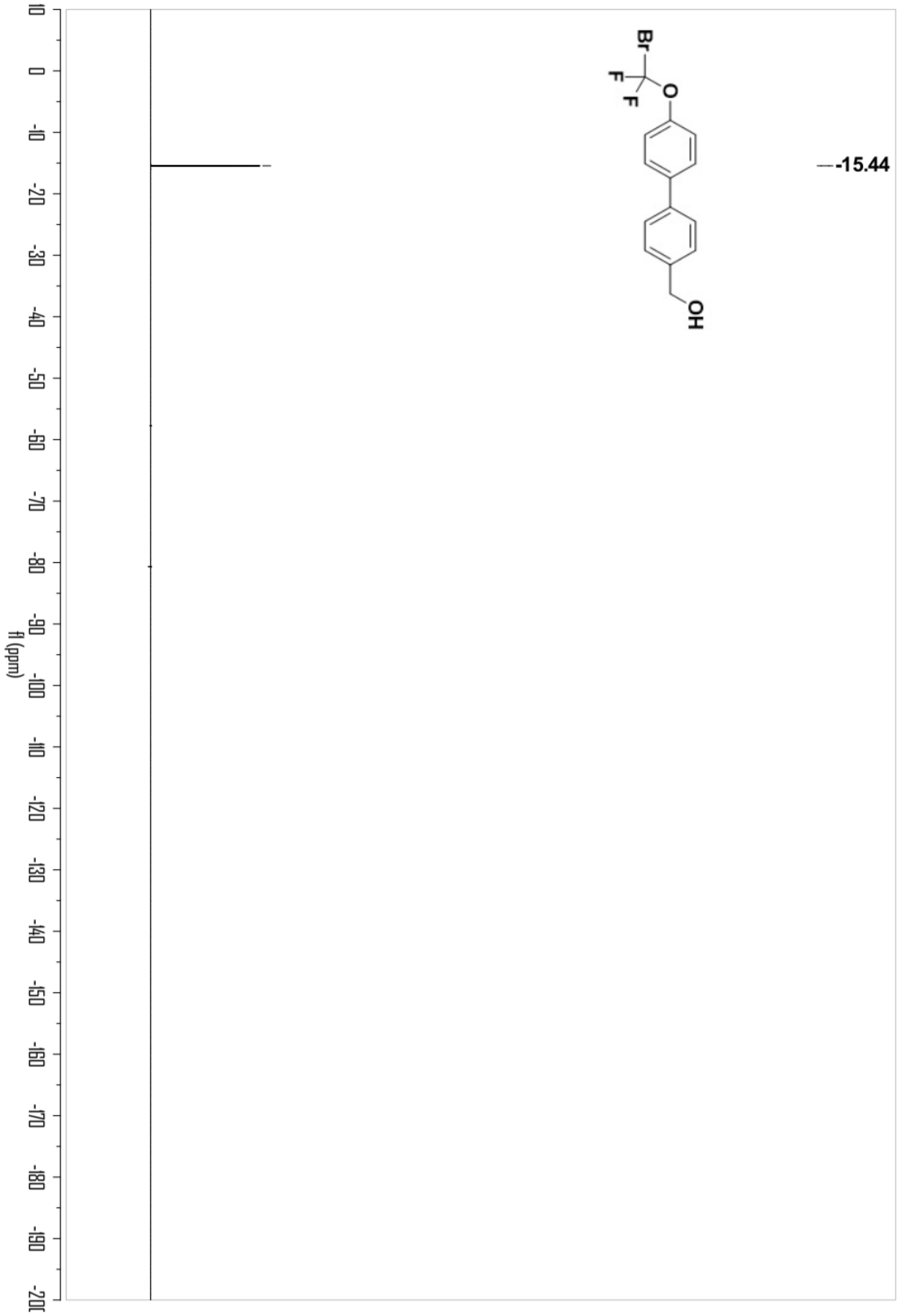

-15.44
f1 (ppm)
Br
F
F
O
OH

^{1}H, ^{13}C and ^{19}F NMR spectra of compound 2Bg

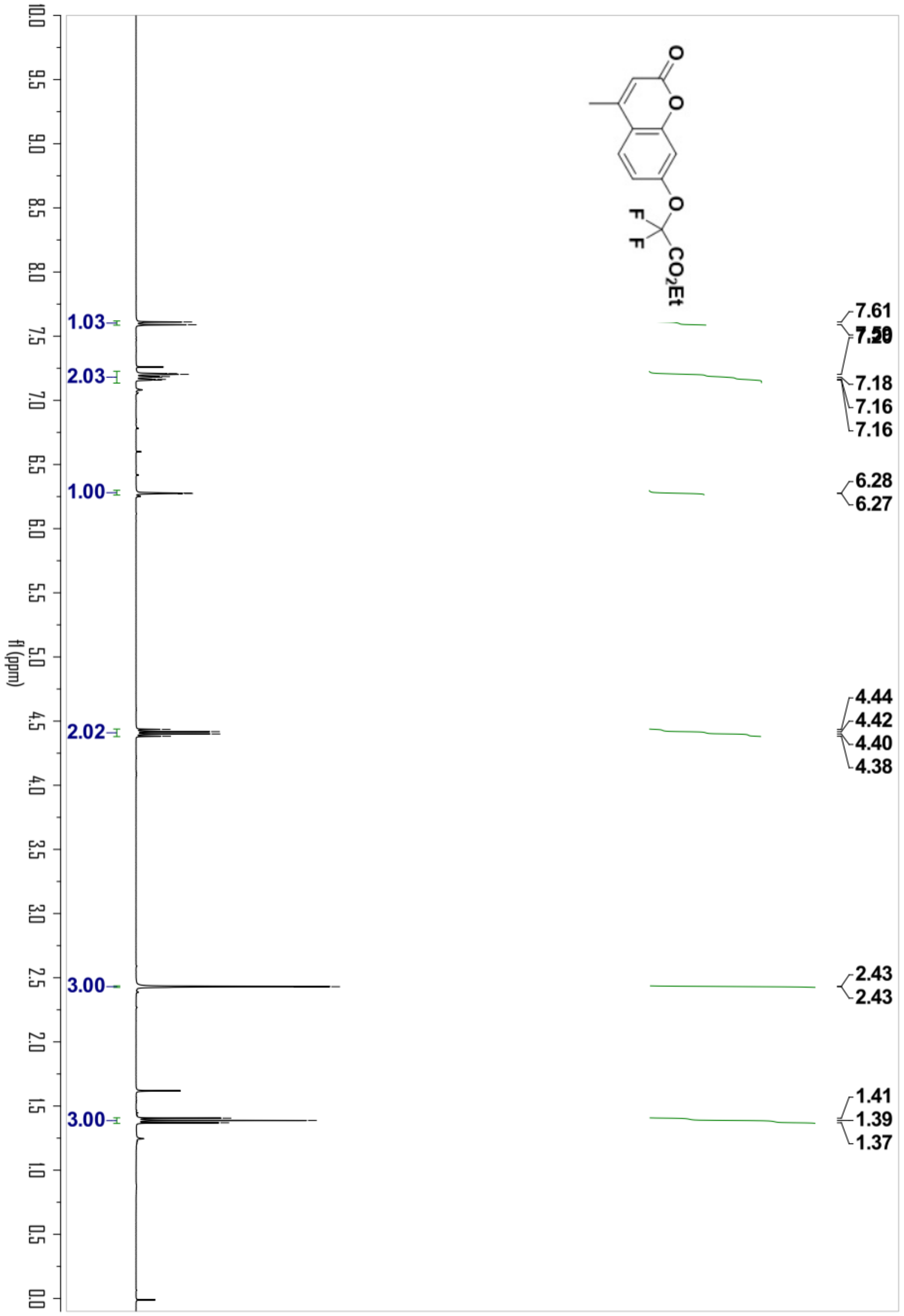

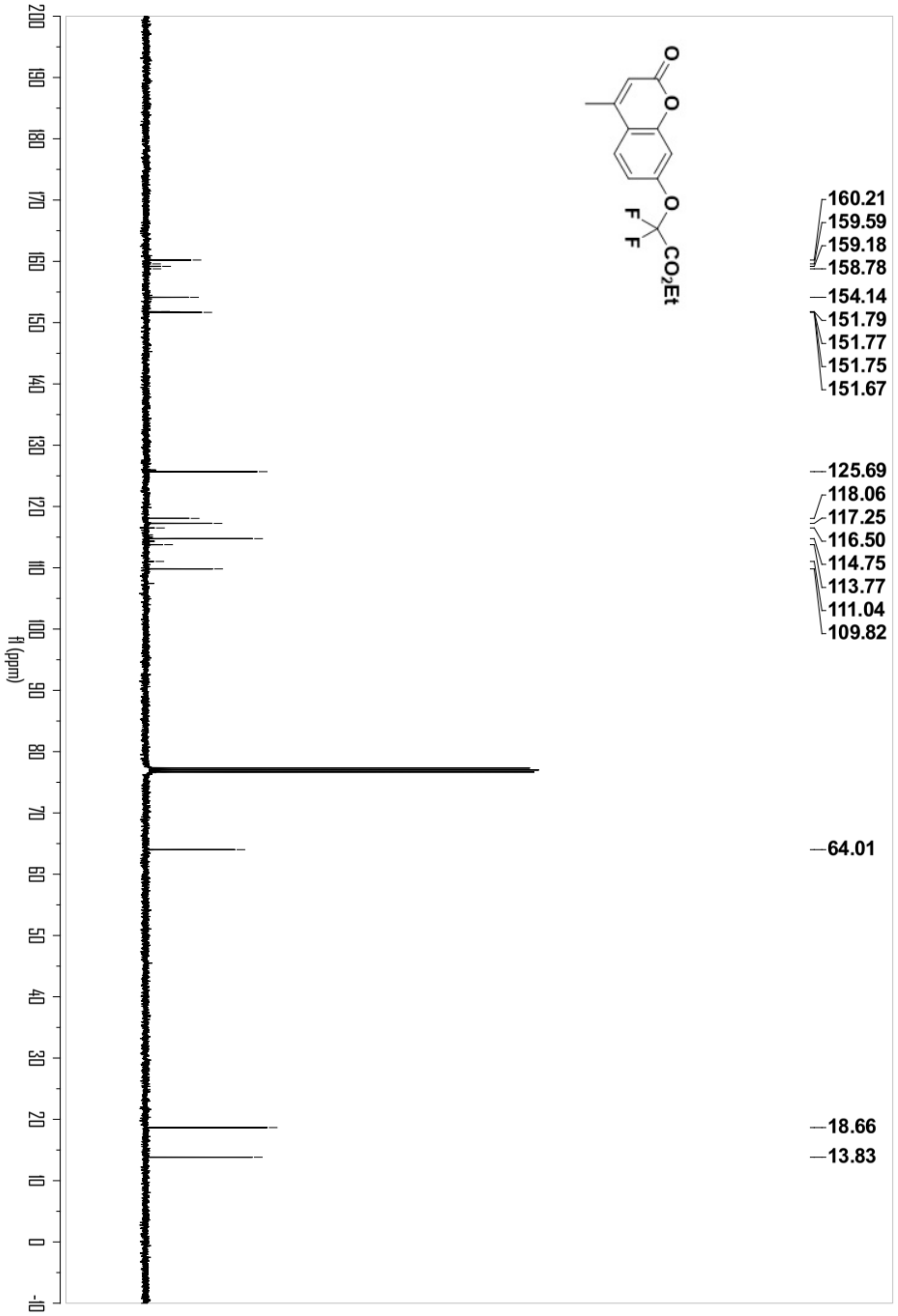
CO2Et
F F
160.21
159.59
159.18
158.78
154.14
151.79
151.77
151.75
151.67
125.69
118.06
117.25
116.50
114.75
113.77
111.04
109.82
64.01
18.66
13.83
f1 (ppm)

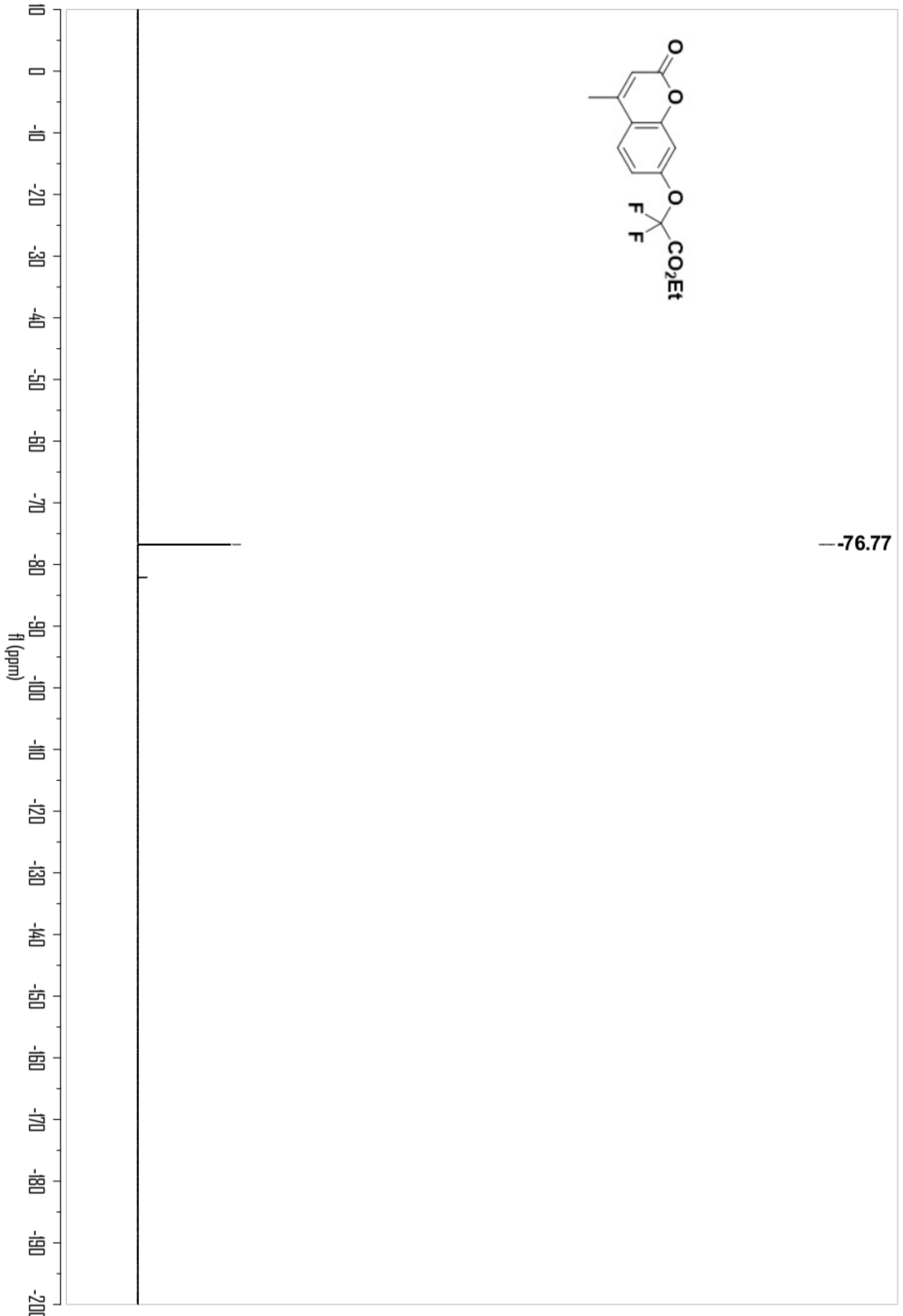

-76.77
f1 (ppm)
CO2Et
F F
O

^{1}H, ^{13}C and ^{19}F NMR spectra of compound 2g

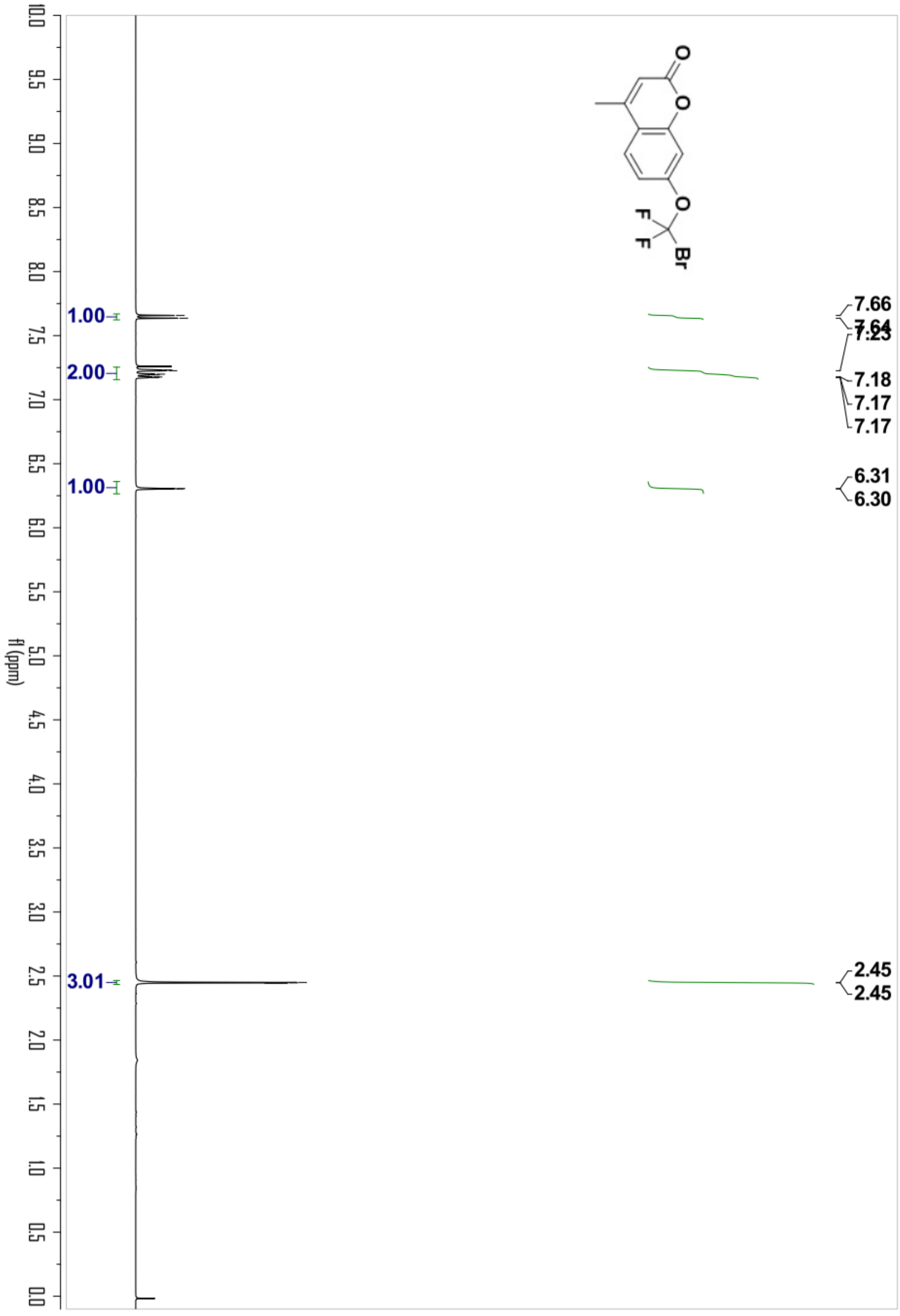

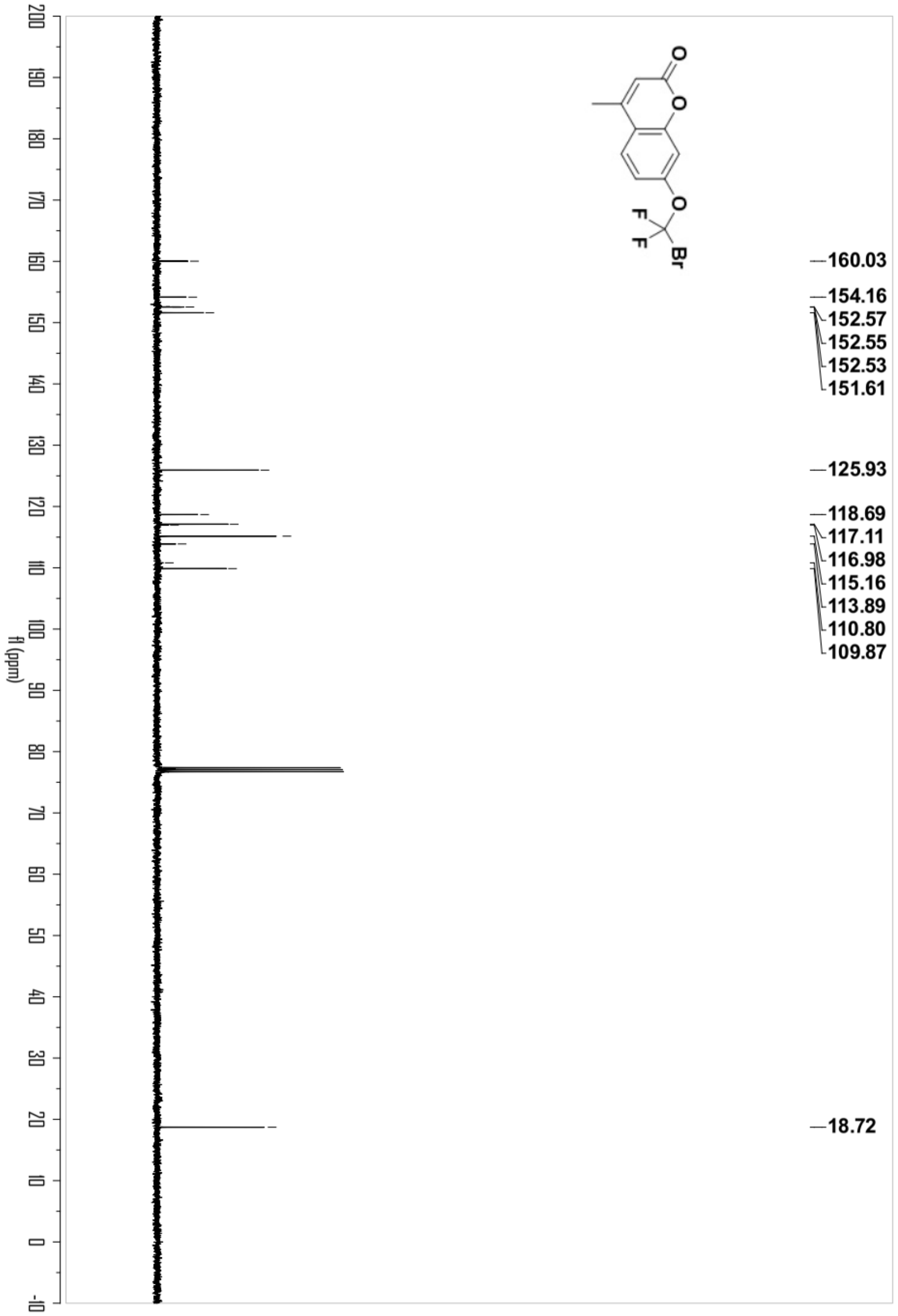
160.03
154.16
152.57
152.55
152.53
151.61
125.93
118.69
117.11
116.98
115.16
113.89
110.80
109.87
18.72
f1 (ppm)
Br
F
F
O

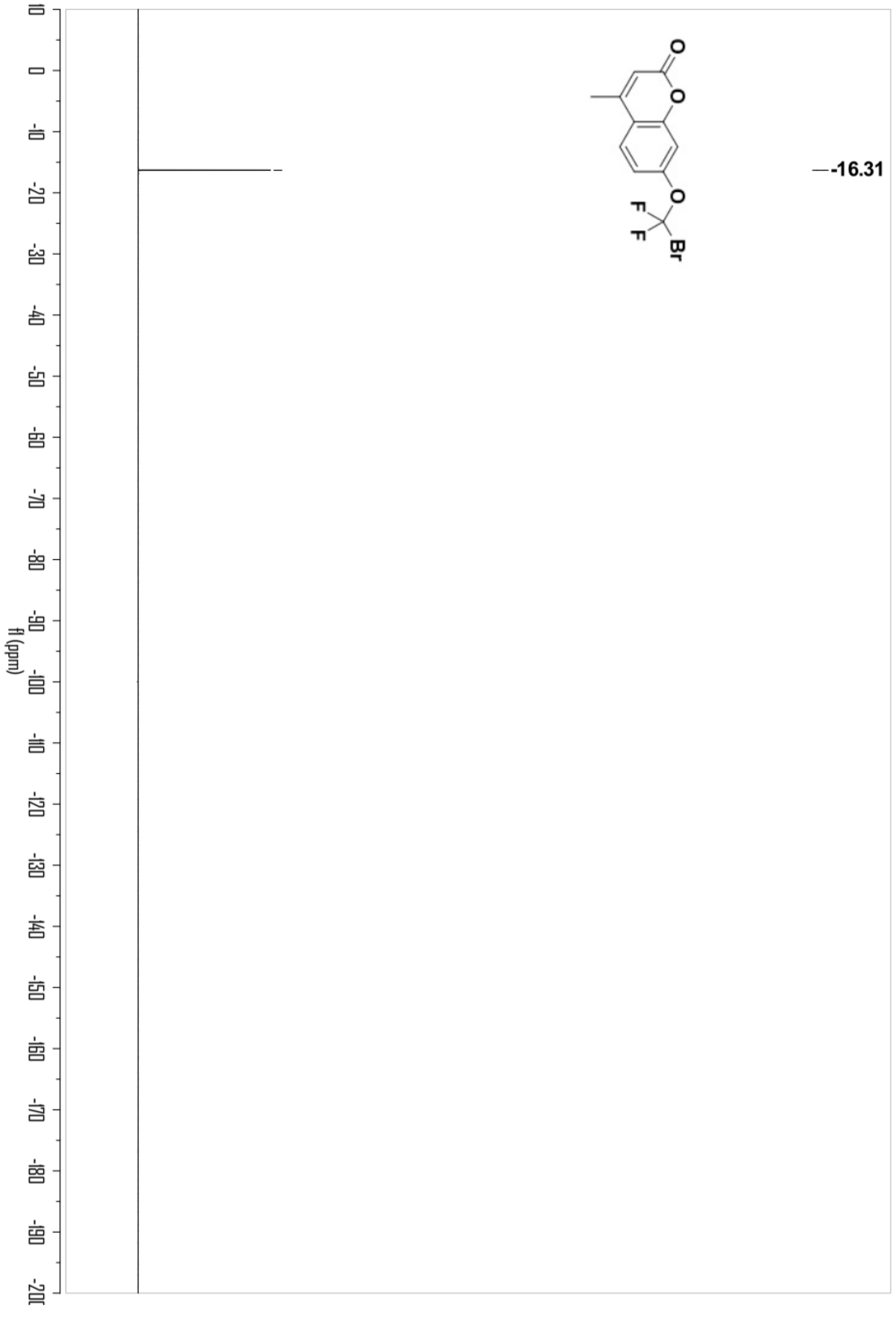
-16.31
f1 (ppm)
10
0
-10
-20
-30
-40
-50
-60
-70
-80
-90
-100
-110
-120
-130
-140
-150
-160
-170
-180
-190
-200

^{1}H, ^{13}C and ^{19}F NMR spectra of compound 3a

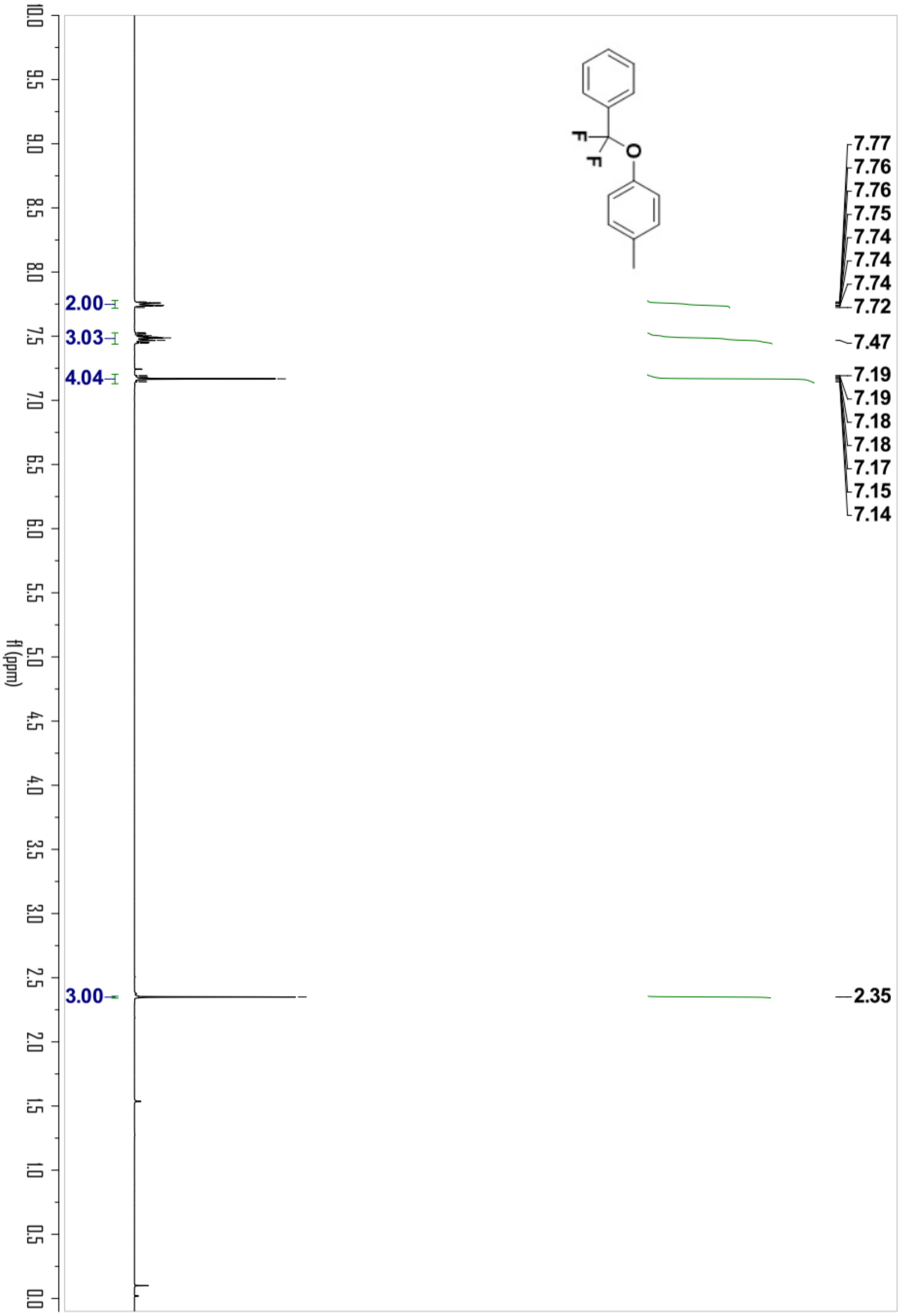

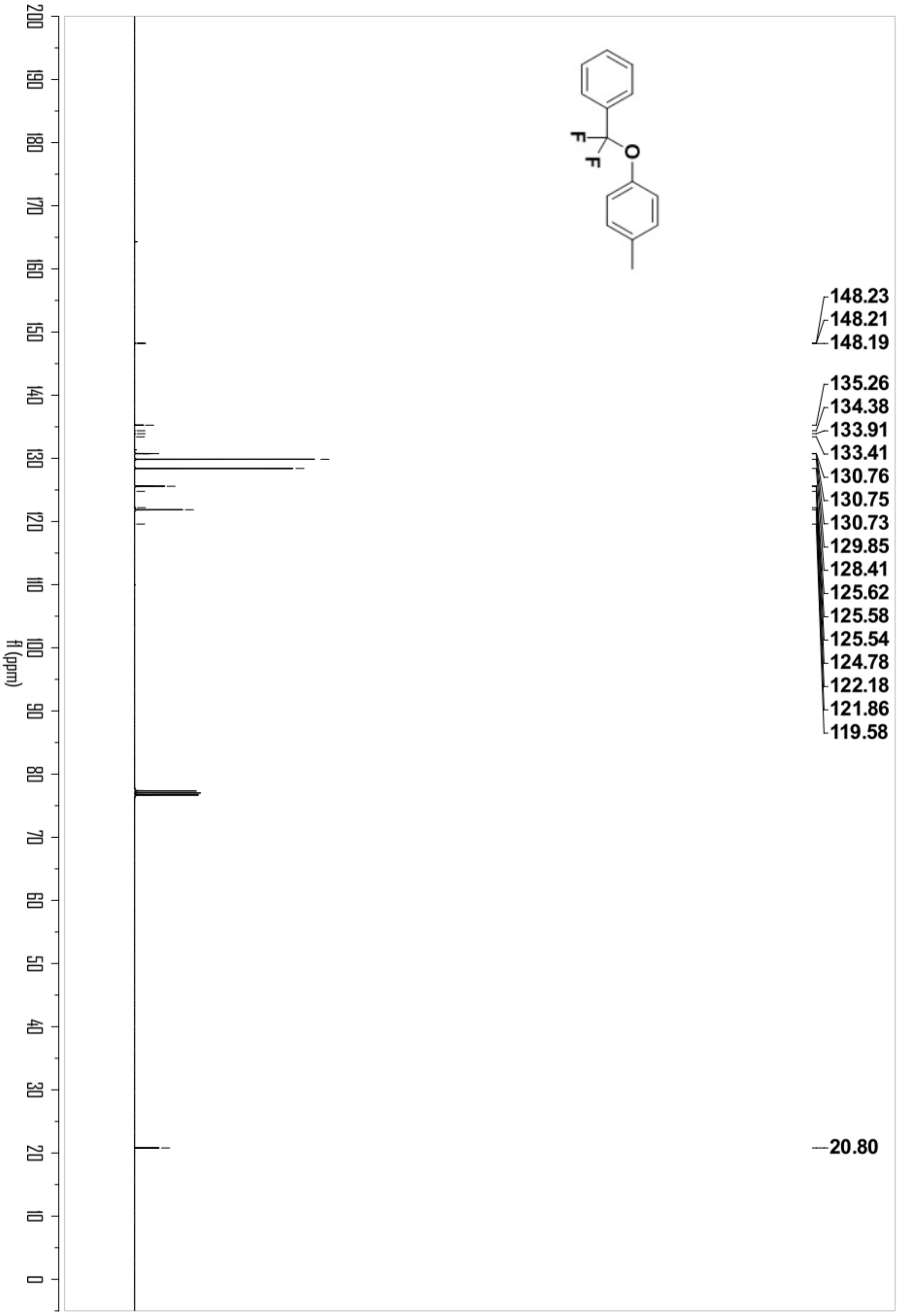
148.23
148.21
148.19
135.26
134.38
133.91
133.41
130.76
130.75
130.73
129.85
128.41
125.62
125.58
125.54
124.78
122.18
121.86
119.58
20.80
f1 (ppm)

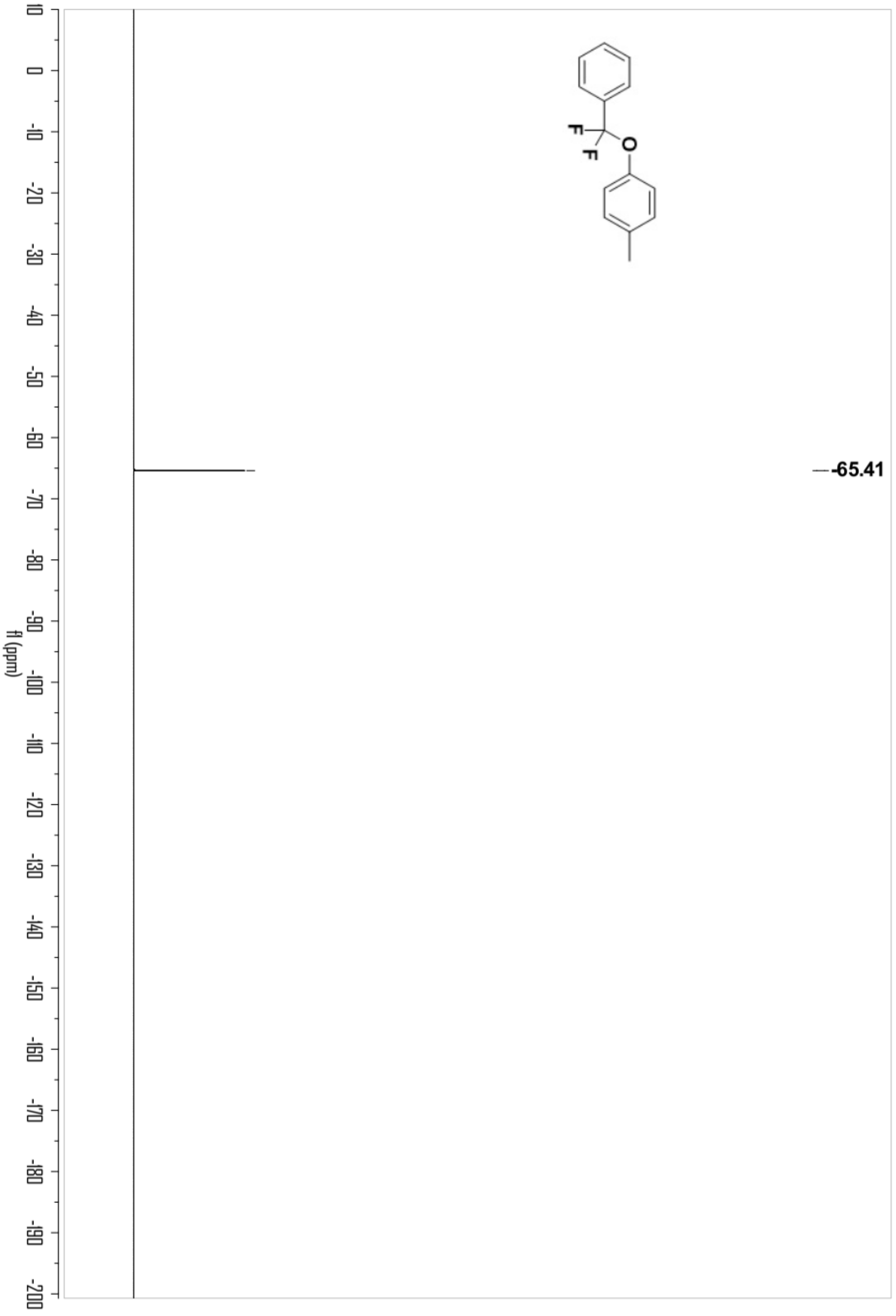
-65.41
f1 (ppm)

^{1}H, ^{13}C and ^{19}F NMR spectra of compound 3b

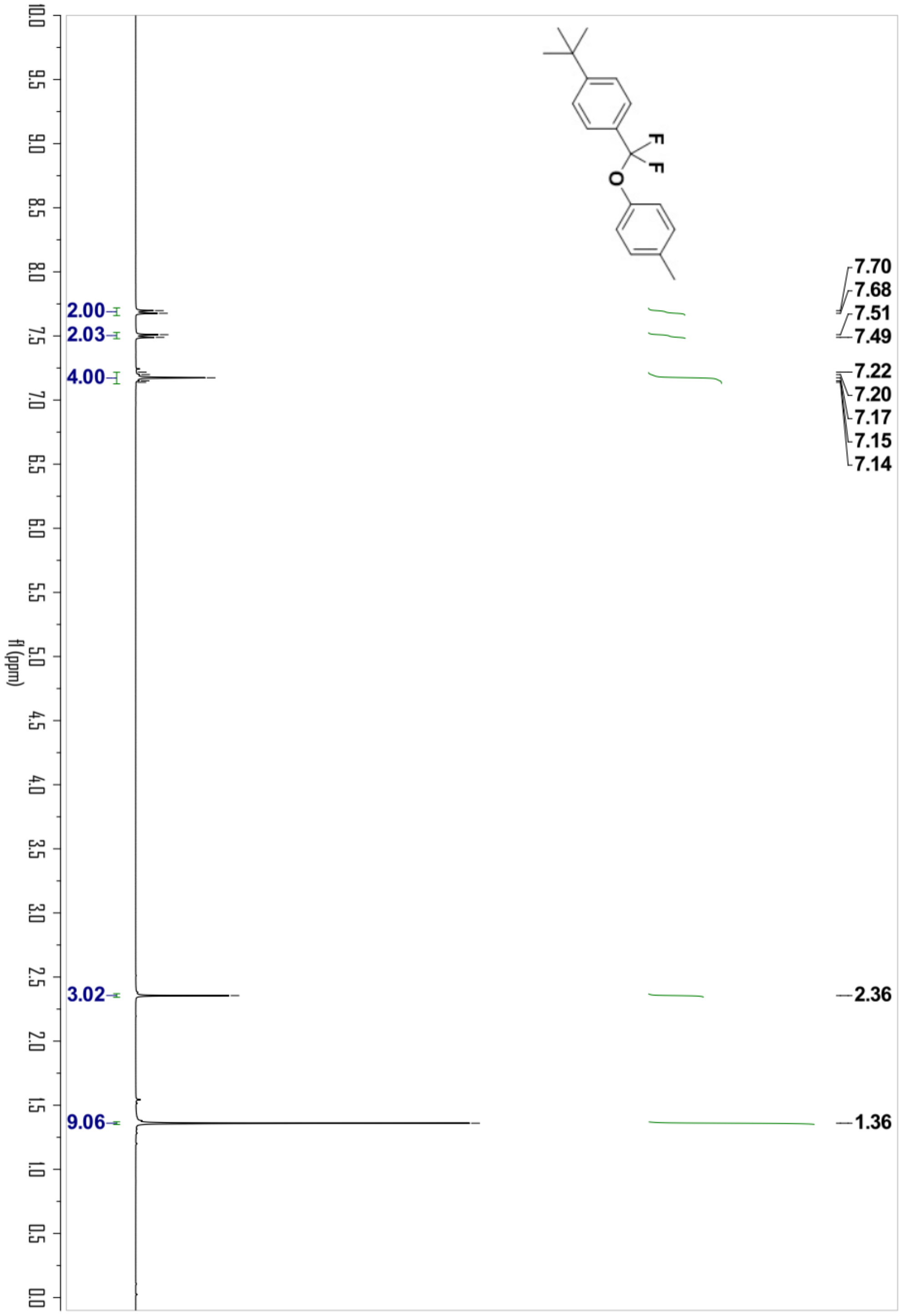

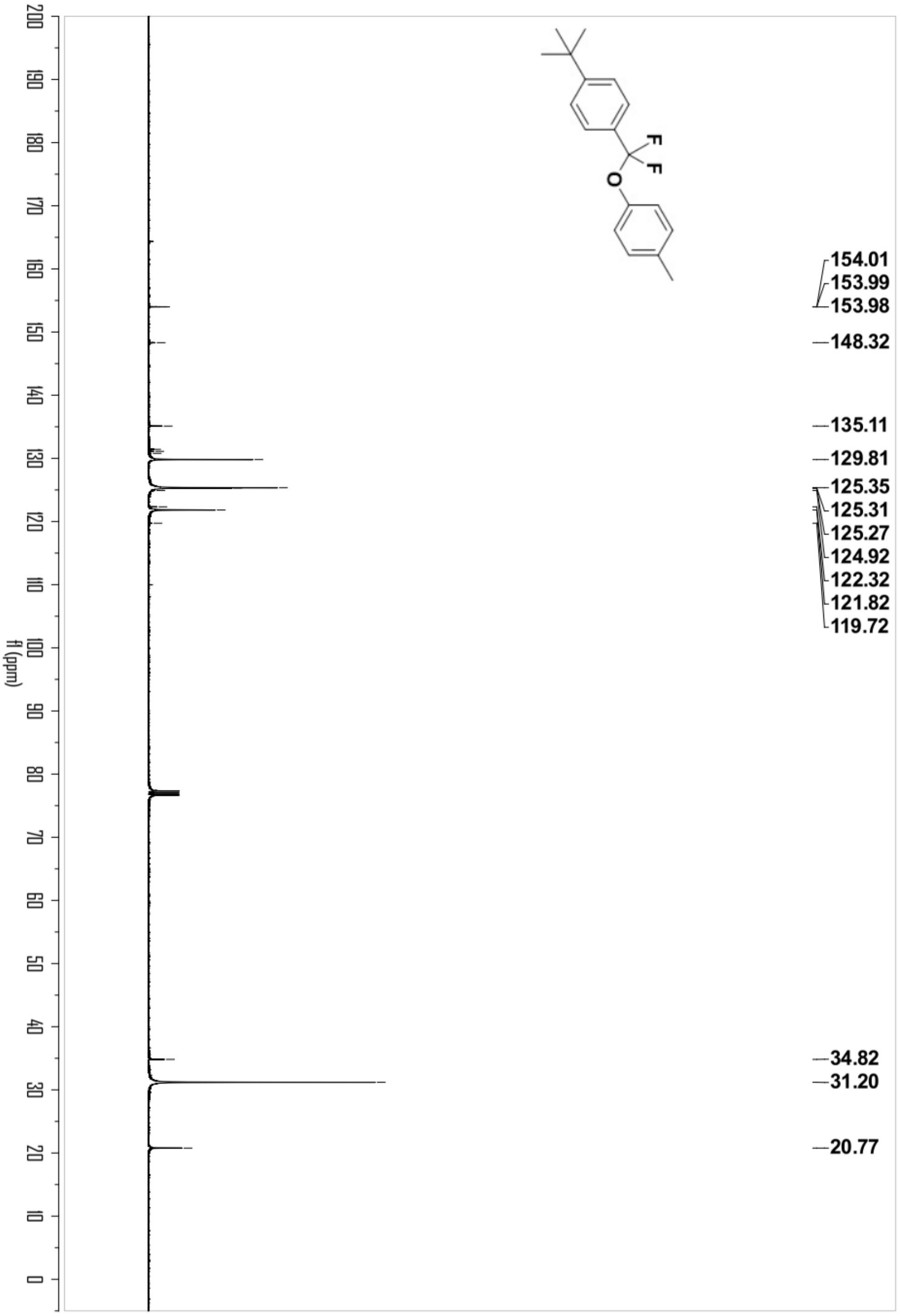
F
F
O
154.01
153.99
153.98
148.32
135.11
129.81
125.35
125.31
125.27
124.92
122.32
121.82
119.72
34.82
31.20
20.77
f1 (ppm)

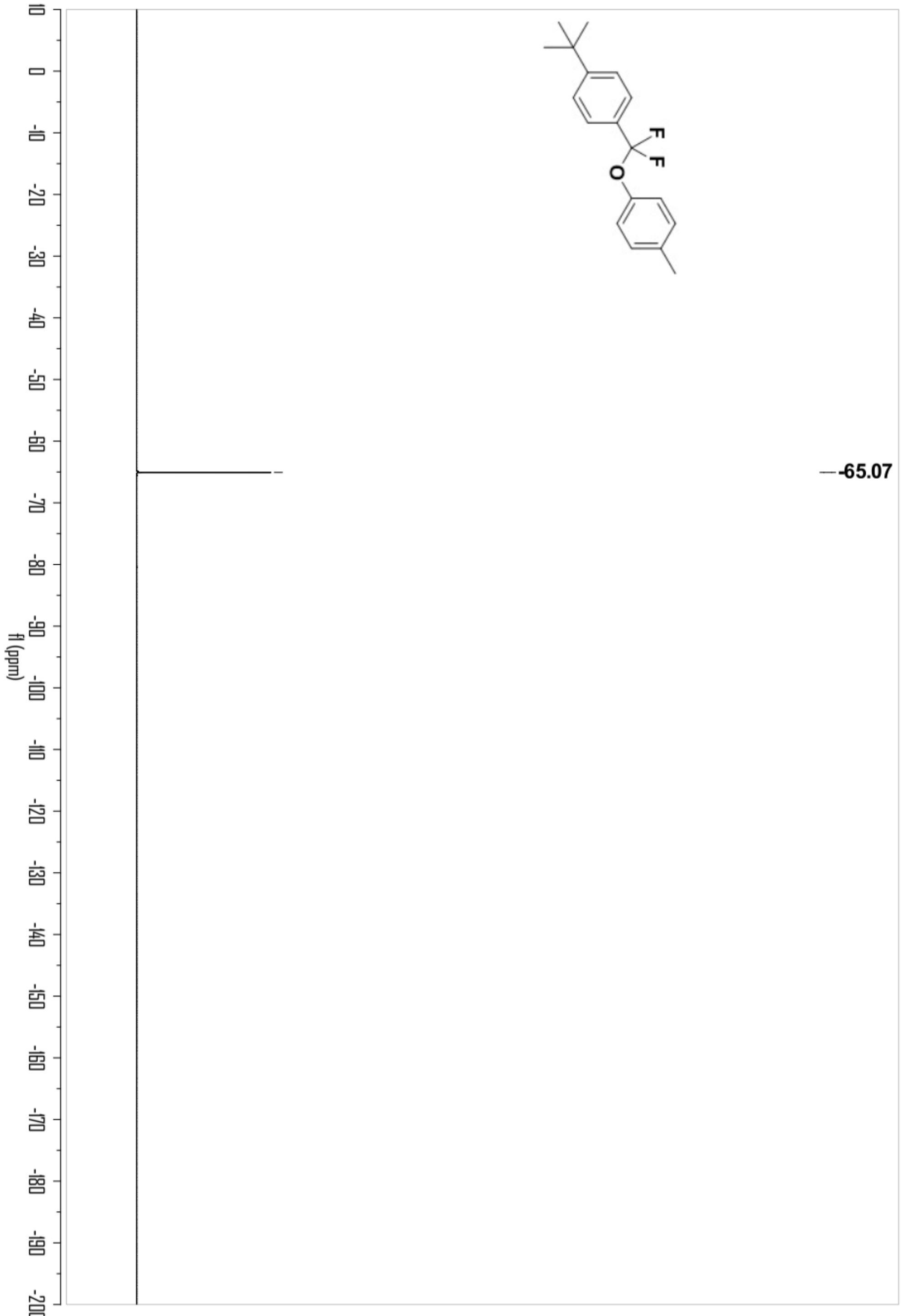
-65.07
F
F
O
f1 (ppm)

^{1}H, ^{13}C and ^{19}F NMR spectra of compound 3c

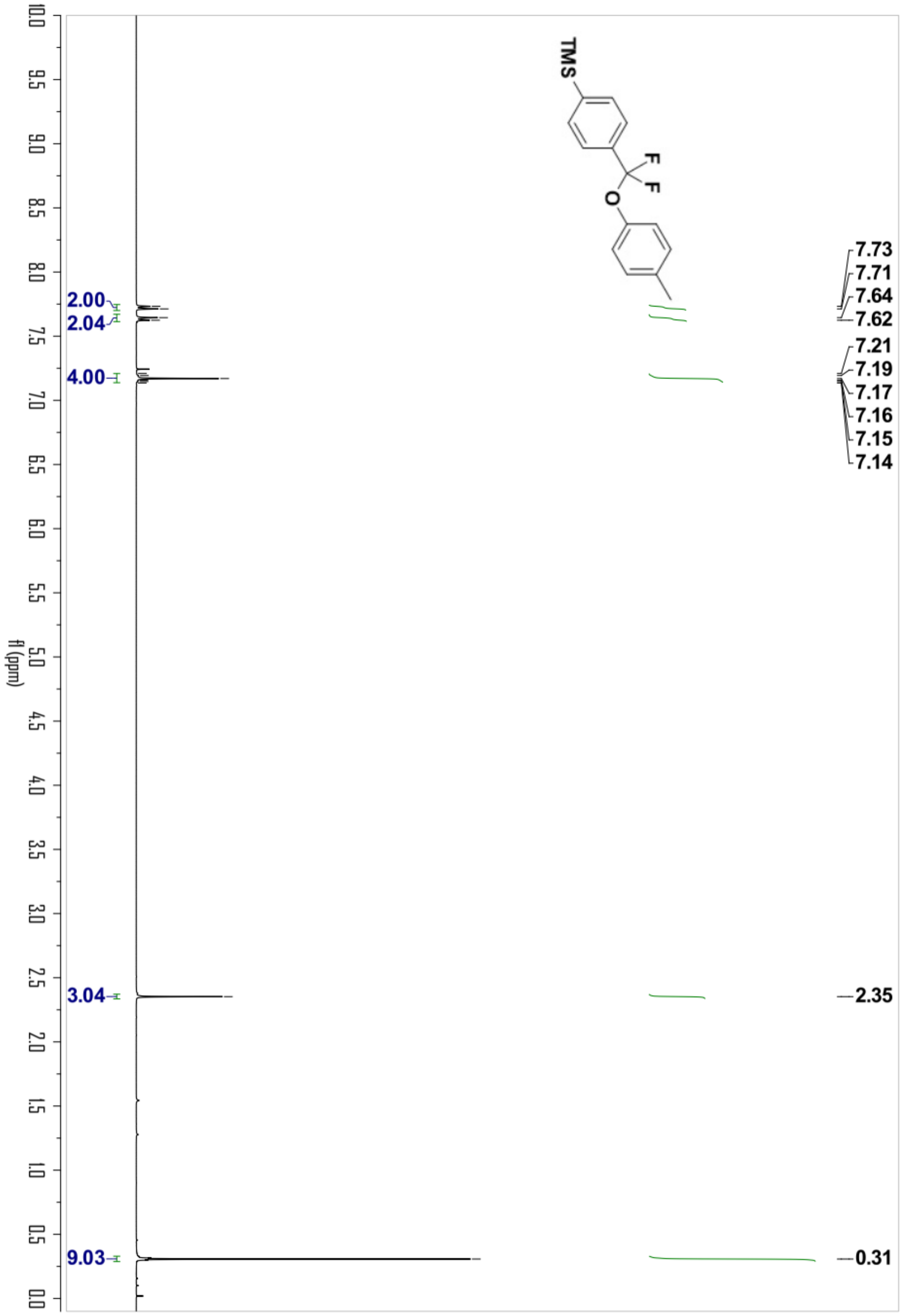

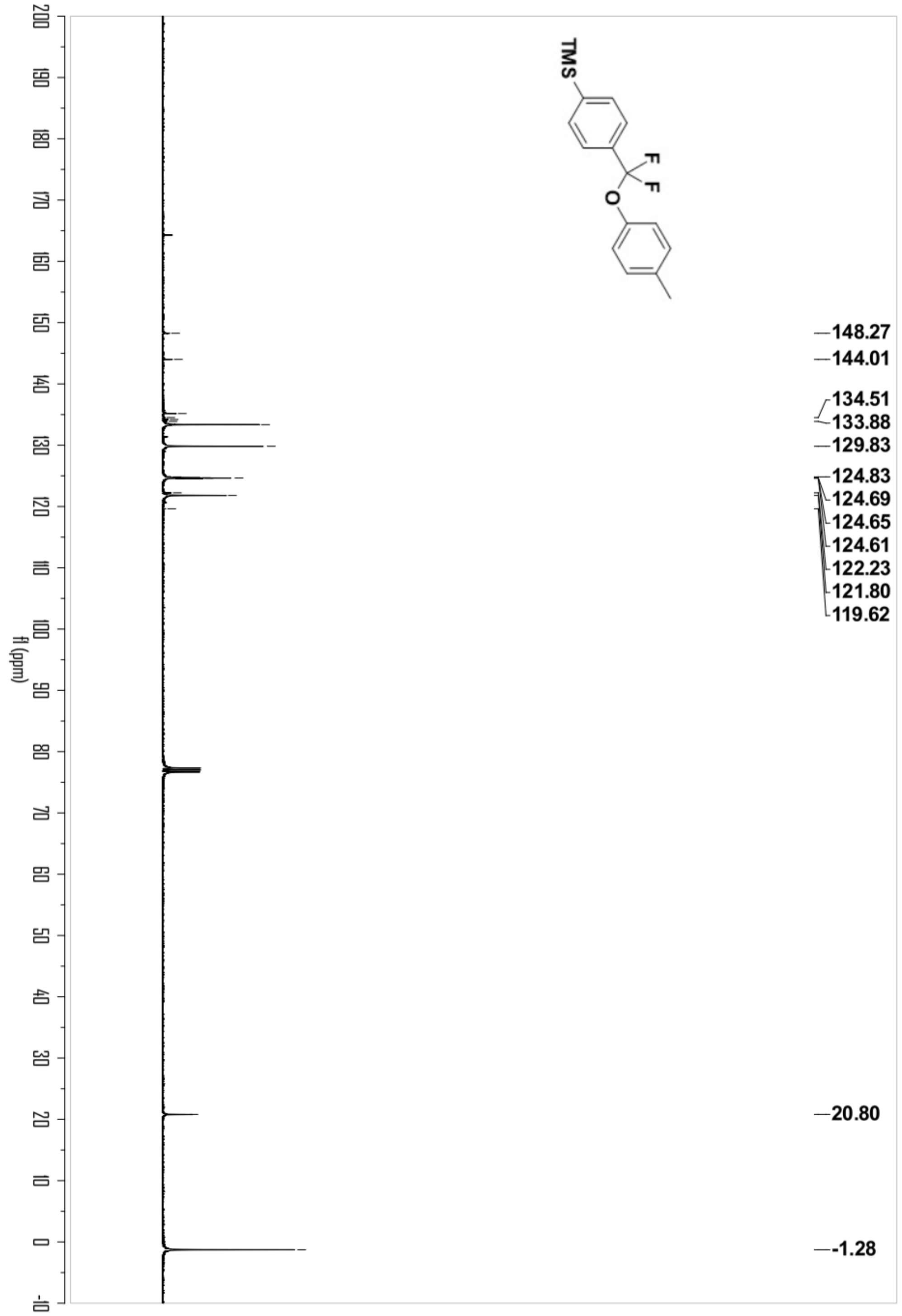
TMS
F
F
O
148.27
144.01
134.51
133.88
129.83
124.83
124.69
124.65
124.61
122.23
121.80
119.62
20.80
-1.28
f1 (ppm)

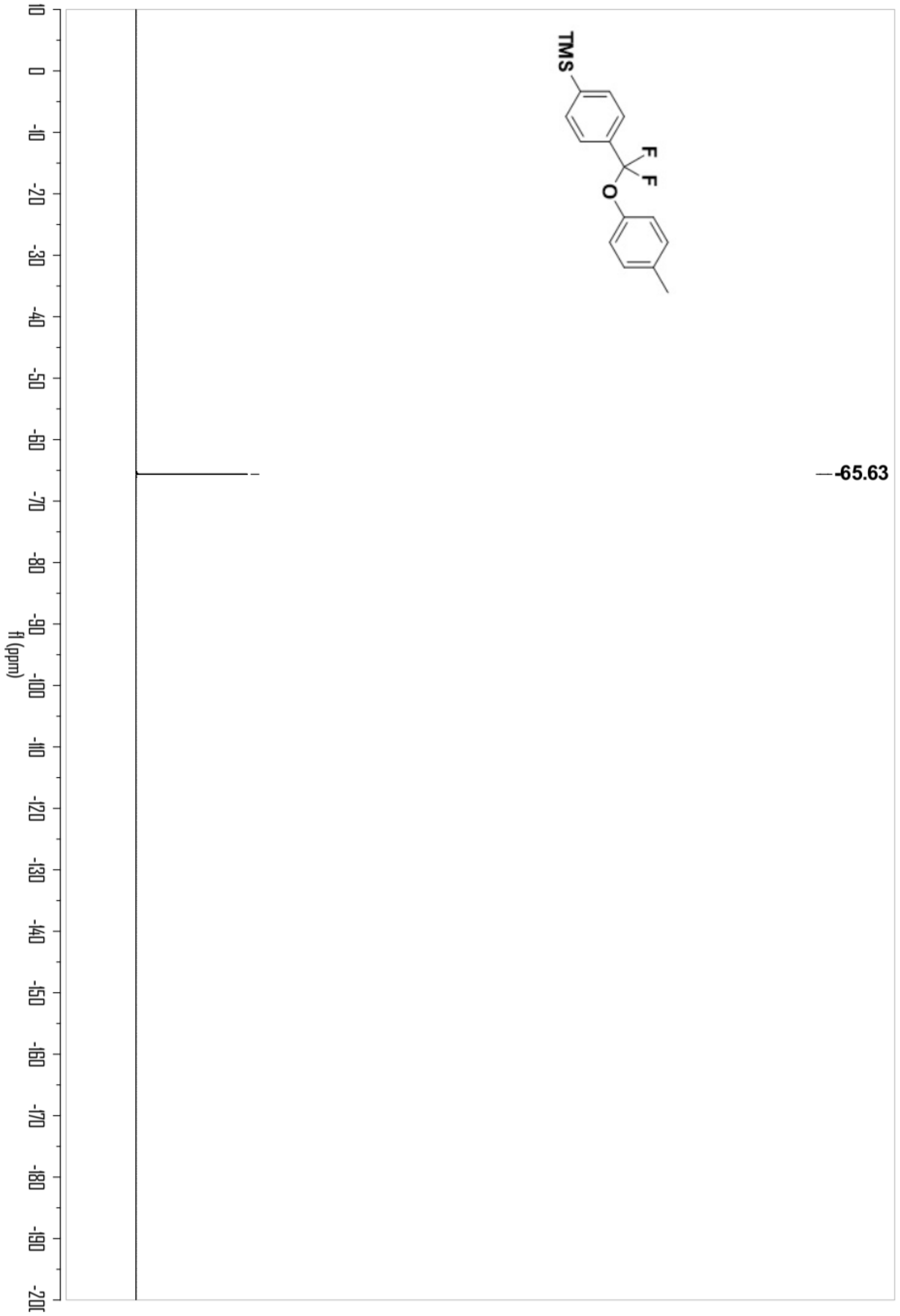
TMS
F
F
O
-65.63
f1 (ppm)

^{1}H, ^{13}C and ^{19}F NMR spectra of compound 3d

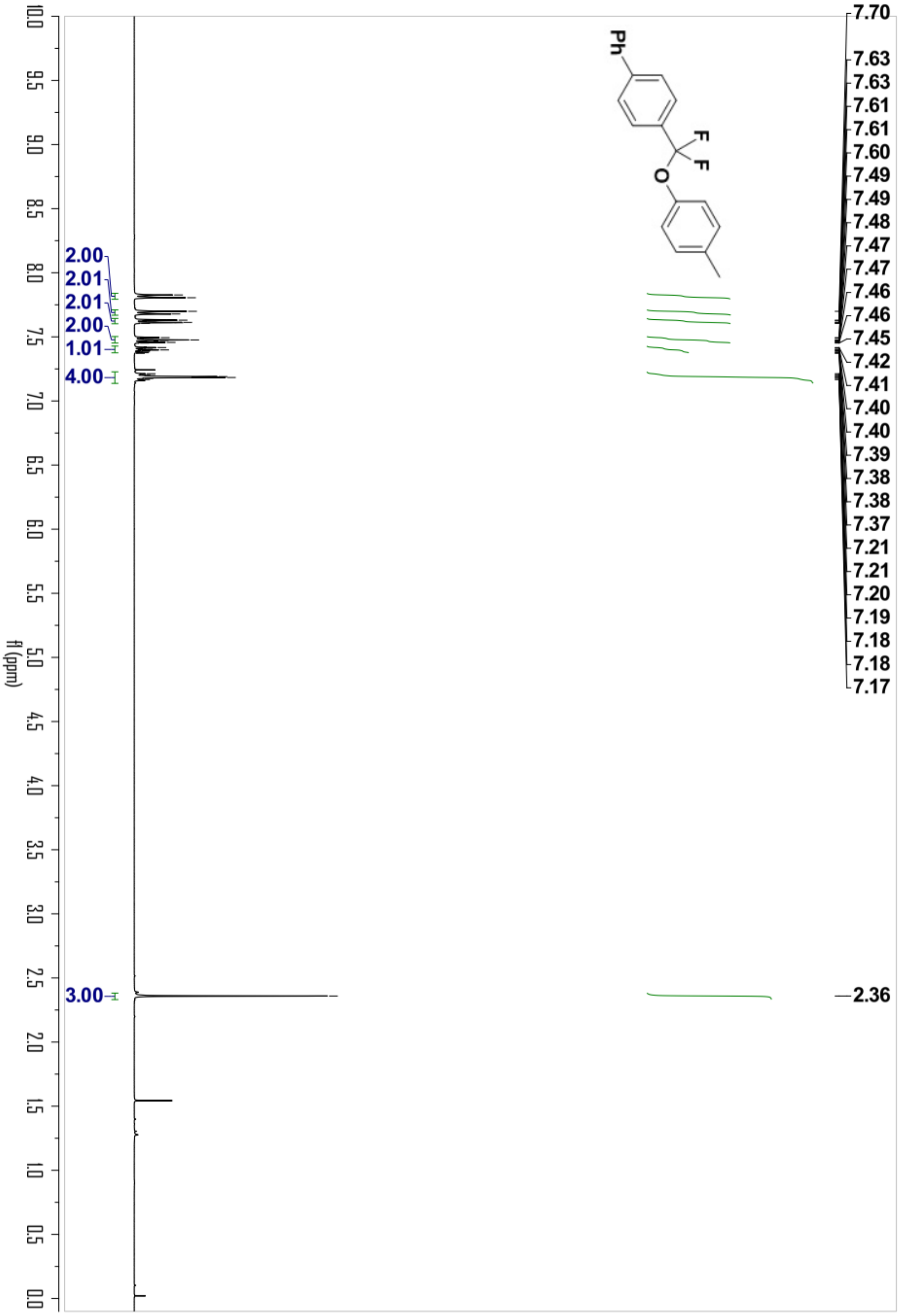

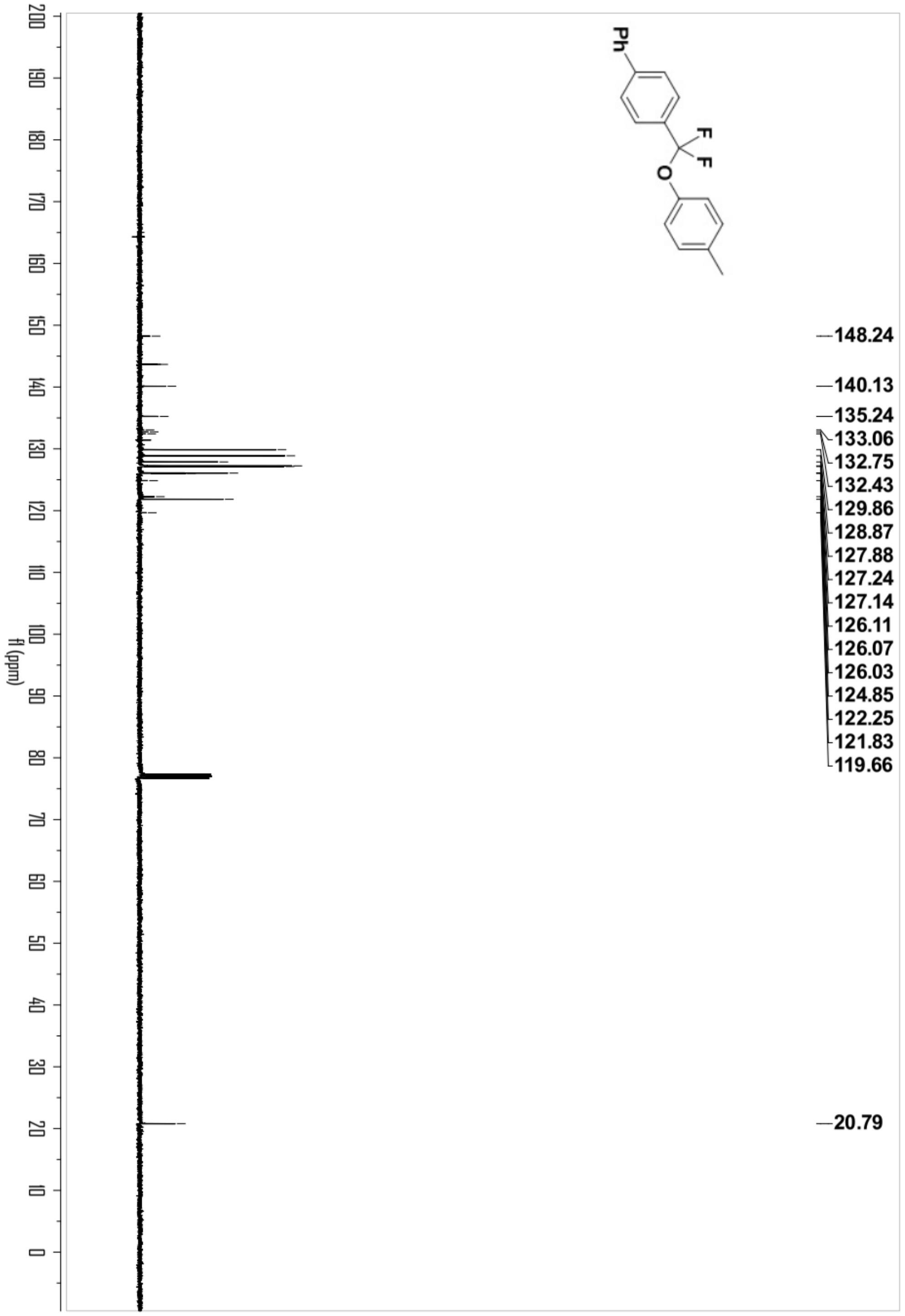
Ph
F
F
O
148.24
140.13
135.24
133.06
132.75
132.43
129.86
128.87
127.88
127.24
127.14
126.11
126.07
126.03
124.85
122.25
121.83
119.66
20.79
f1 (ppm)

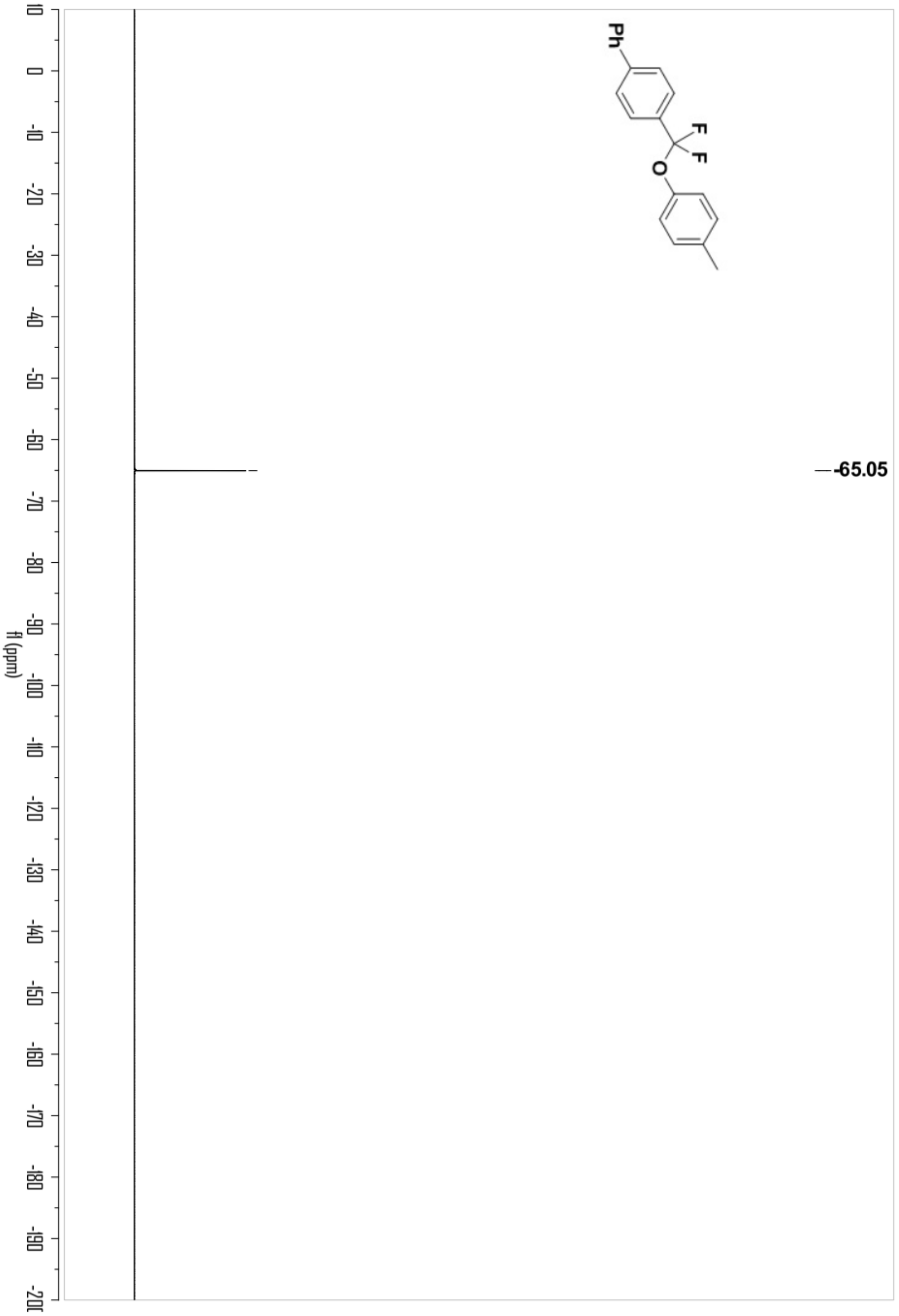

Ph
F
F
O
-65.05
f1 (ppm)
10
0
-10
-20
-30
-40
-50
-60
-70
-80
-90
-100
-110
-120
-130
-140
-150
-160
-170
-180
-190

^{1}H, ^{13}C and ^{19}F NMR spectra of compound 3e

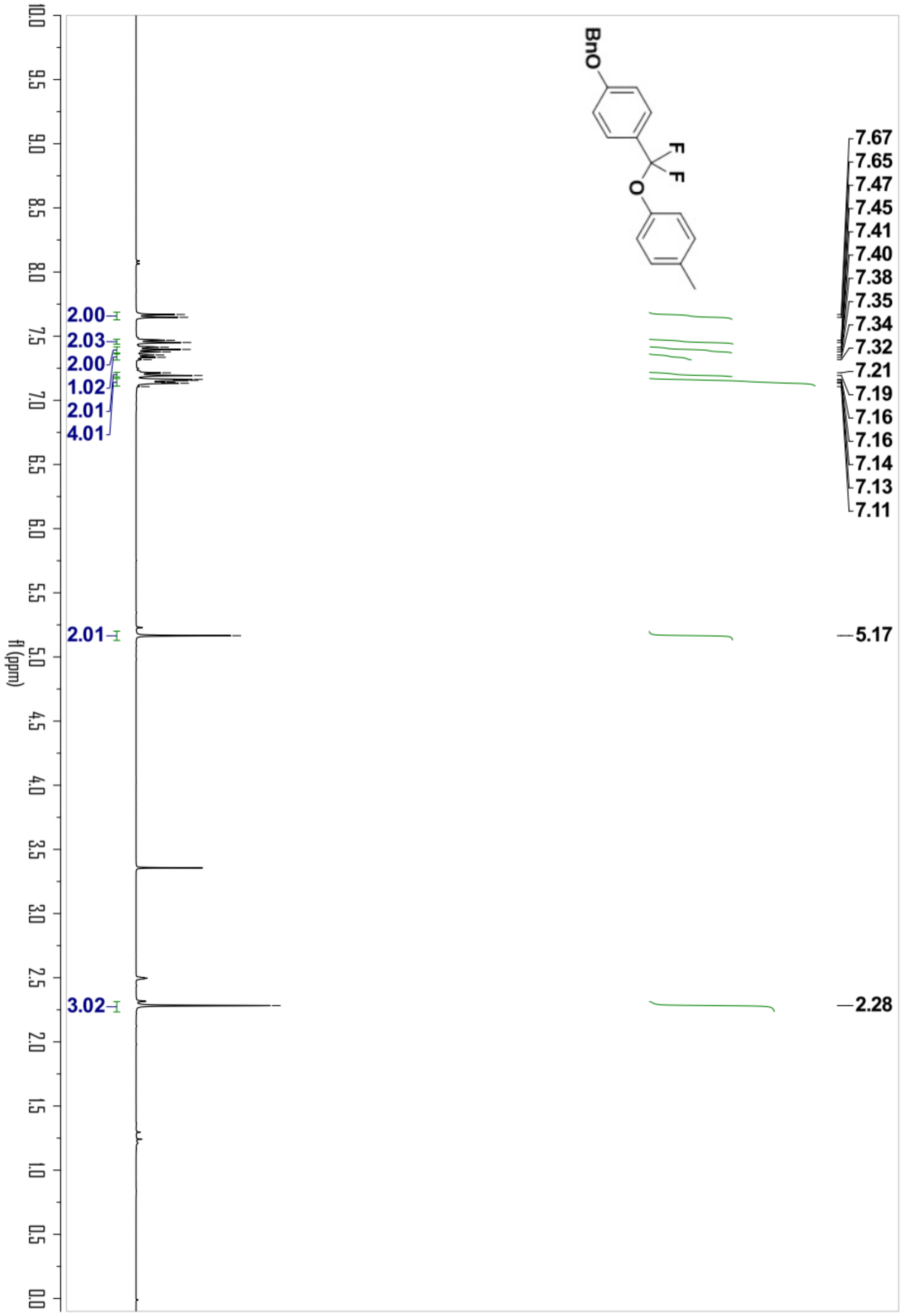

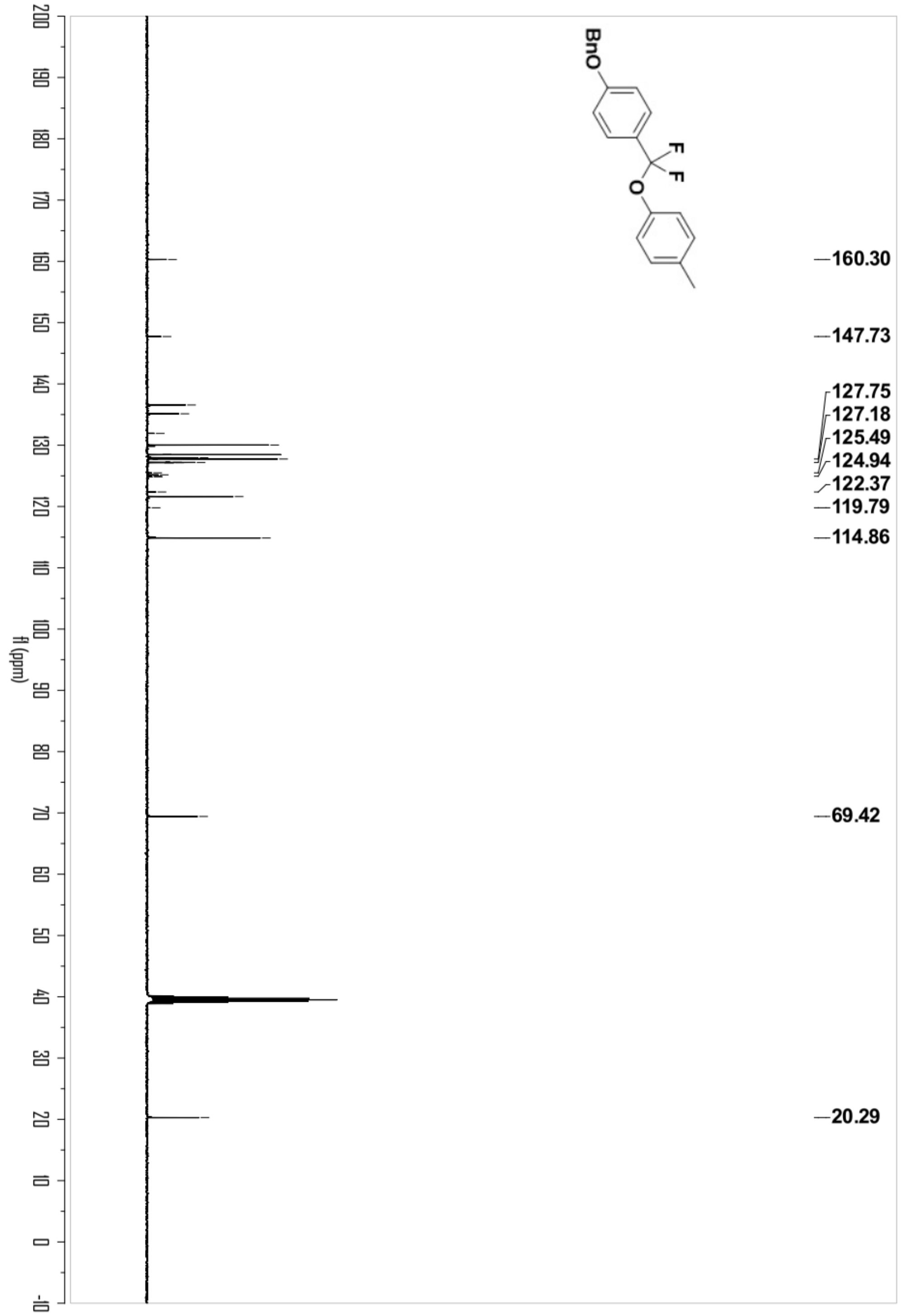
BnO
F
F
O
160.30
147.73
127.75
127.18
125.49
124.94
122.37
119.79
114.86
69.42
20.29
f1 (ppm)

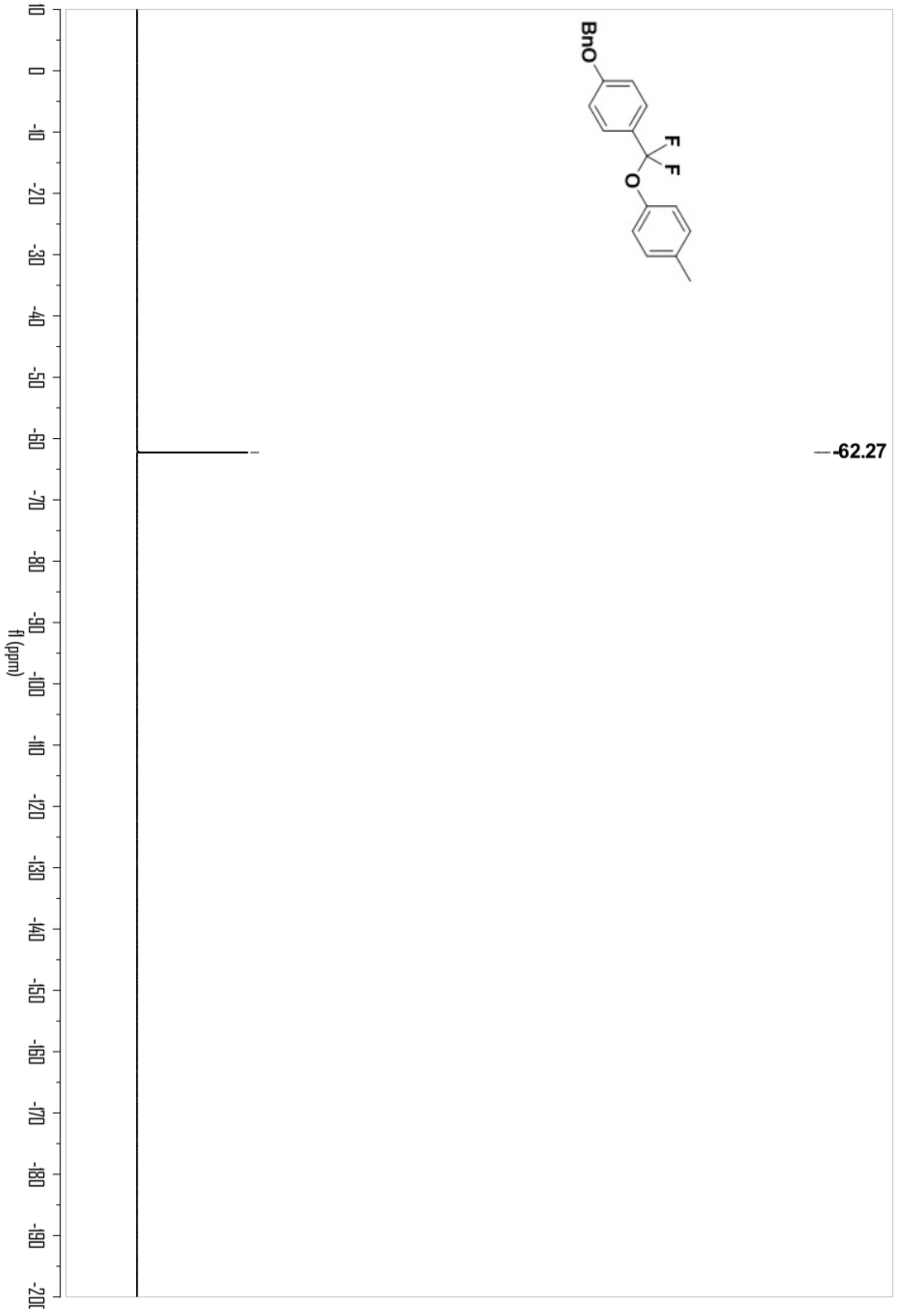
BnO
F
F
O
-62.27
f1 (ppm)

^{1}H, ^{13}C and ^{19}F NMR spectra of compound 3f

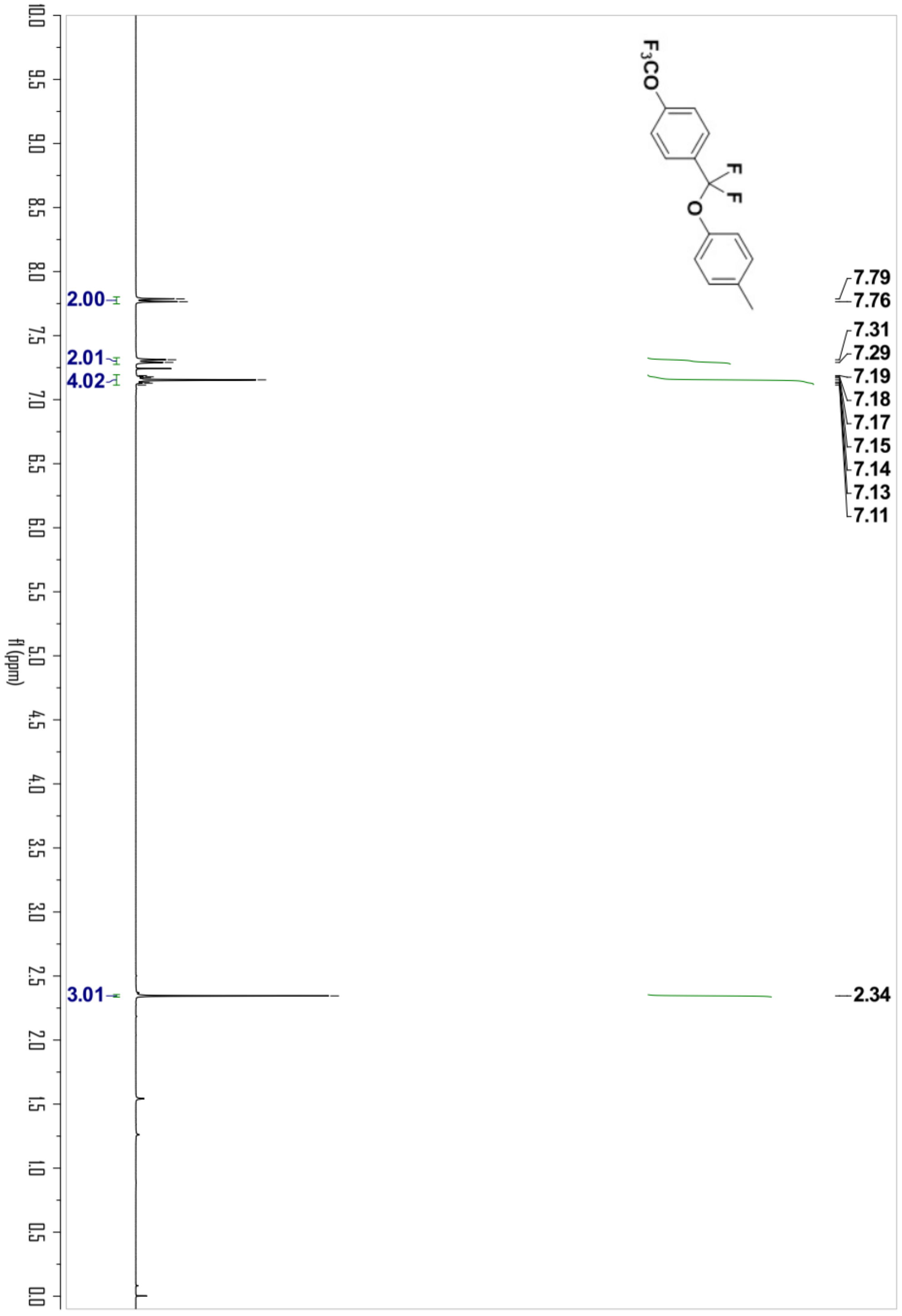

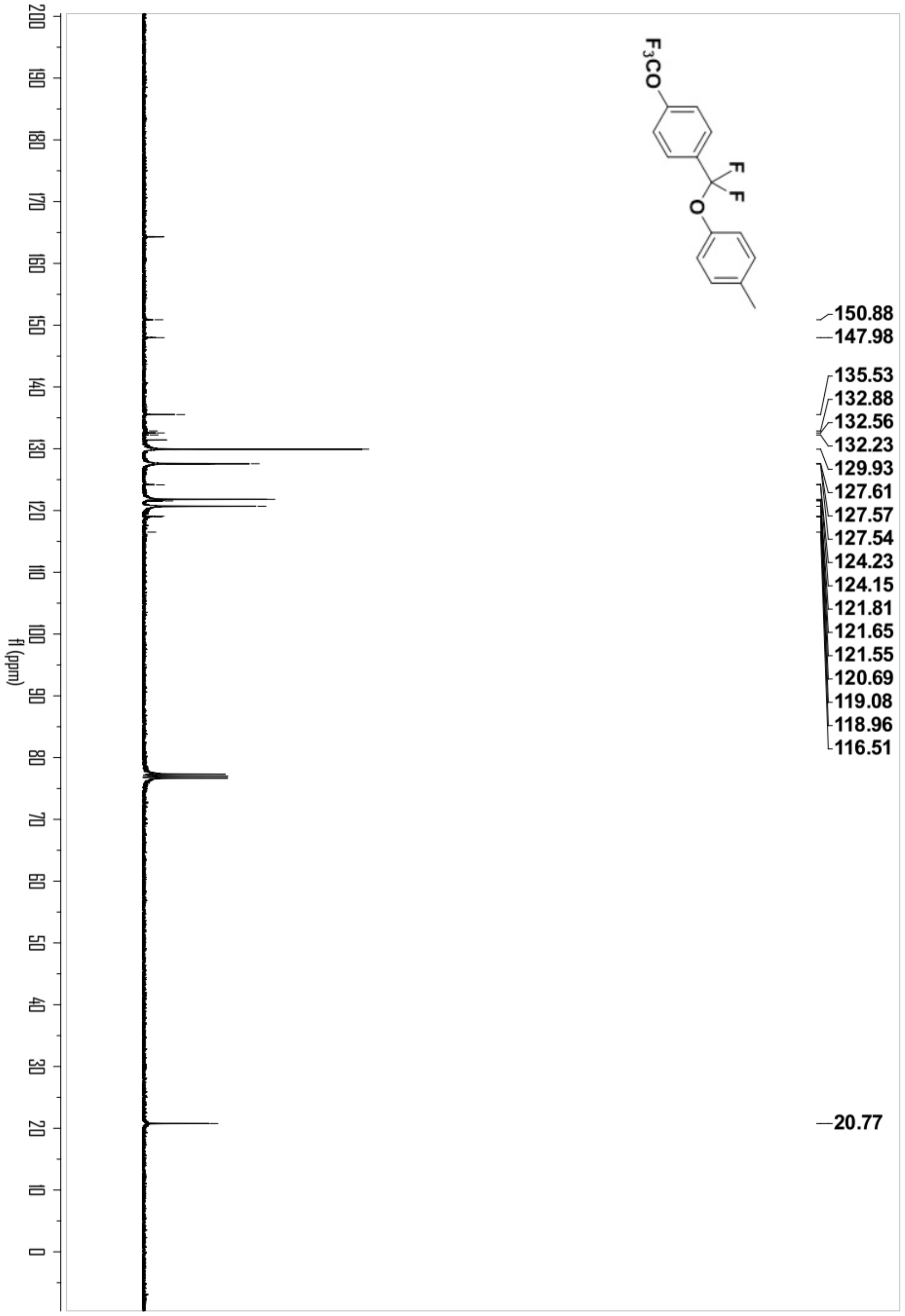
F3CO
F
F
O
150.88
147.98
135.53
132.88
132.56
132.23
129.93
127.61
127.57
127.54
124.23
124.15
121.81
121.65
121.55
120.69
119.08
118.96
116.51
20.77
f1 (ppm)

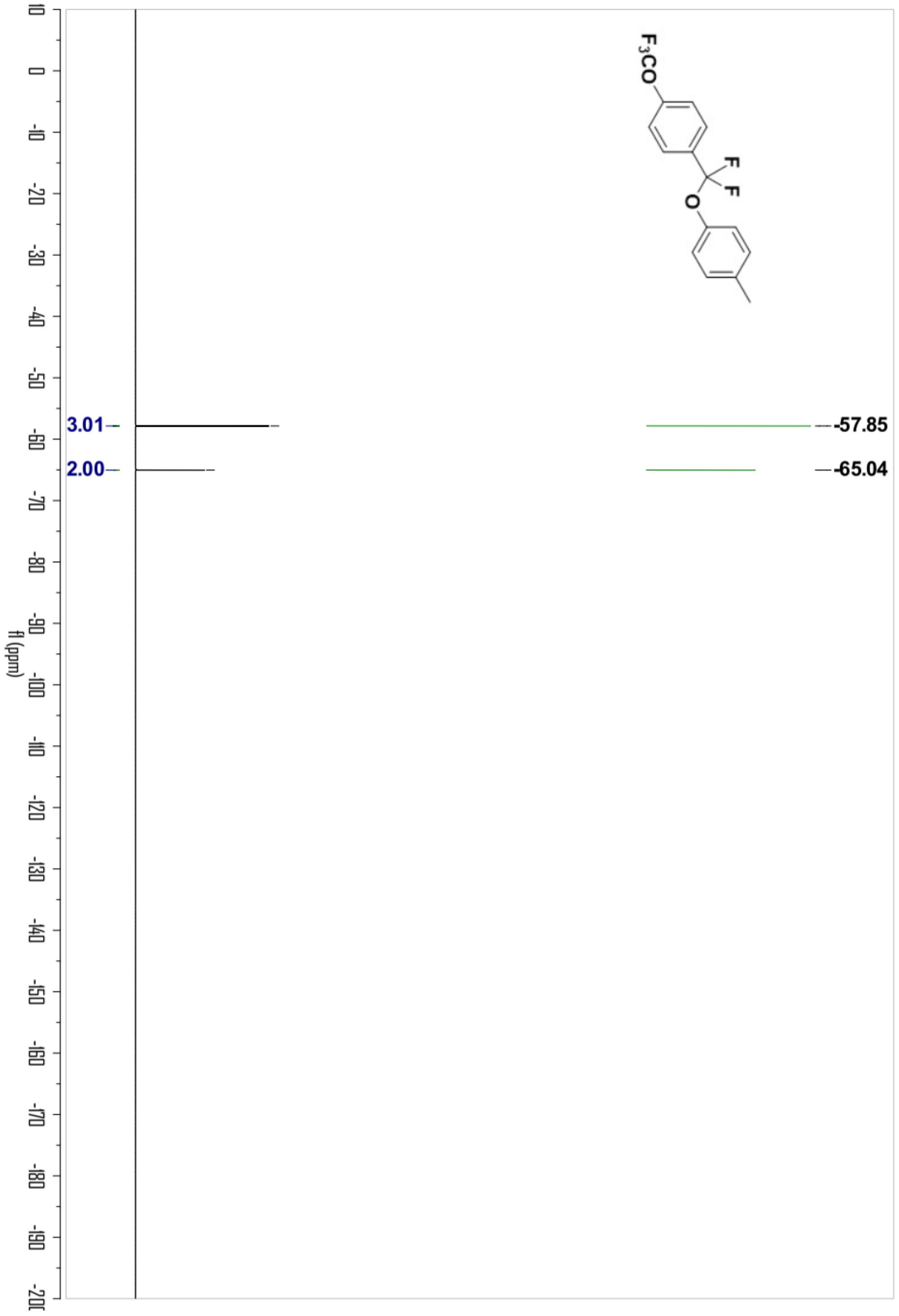

-57.85
-65.04
3.01
2.00
f1 (ppm)

^{1}H, ^{13}C and ^{19}F NMR spectra of compound 3g

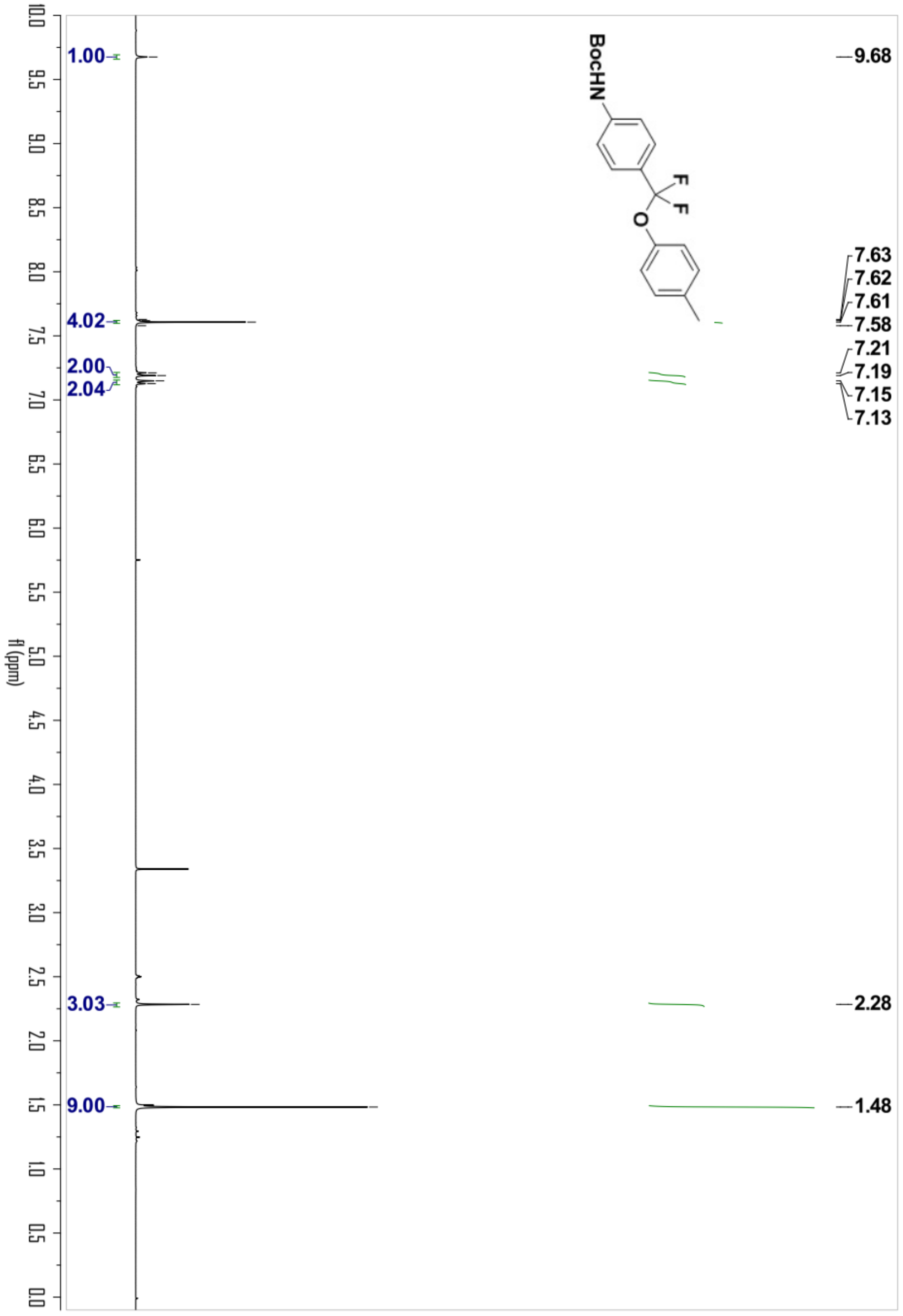

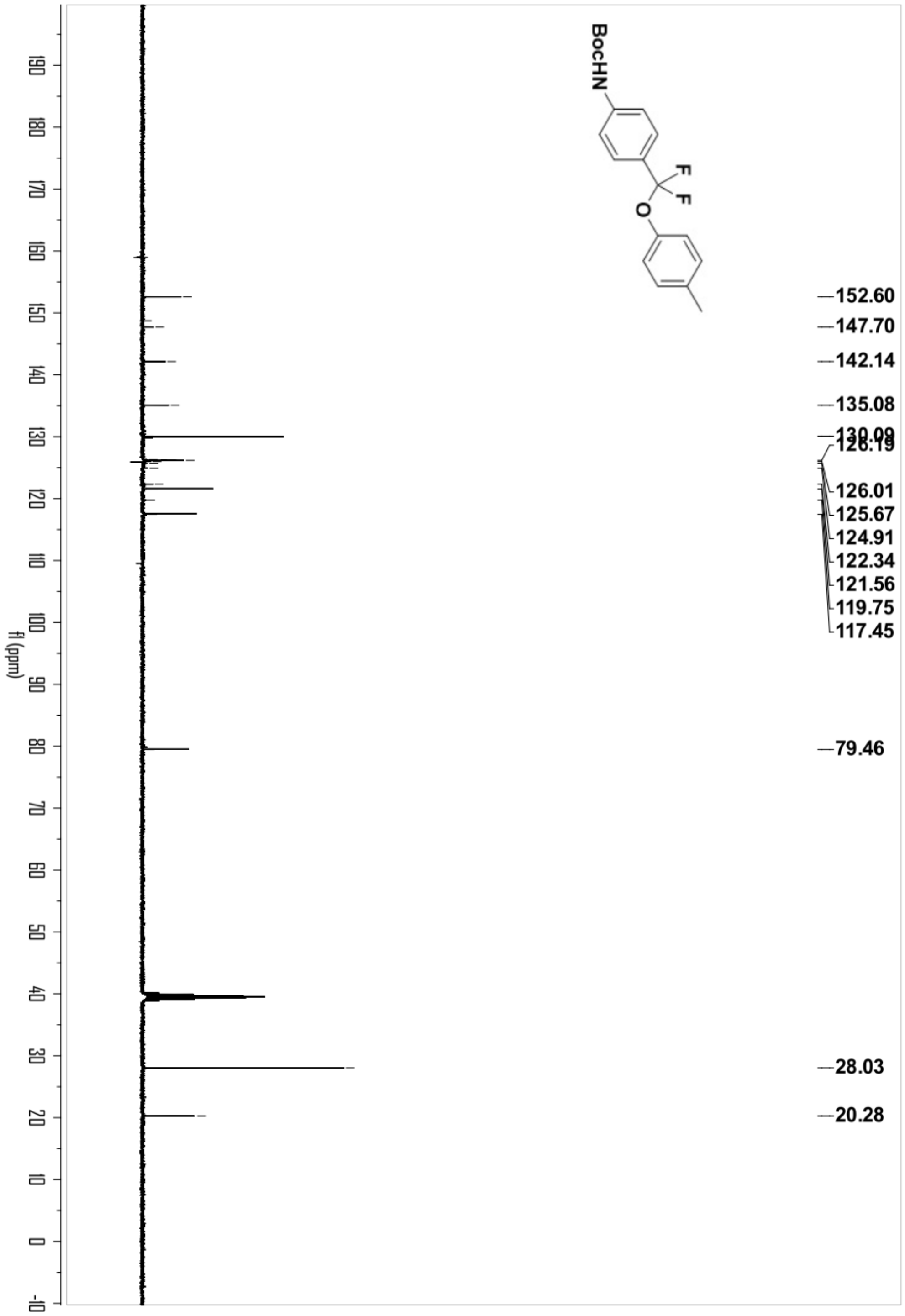
BocHN
F
F
O
152.60
147.70
142.14
135.08
130.09
126.19
126.01
125.67
124.91
122.34
121.56
119.75
117.45
79.46
28.03
20.28
f1 (ppm)

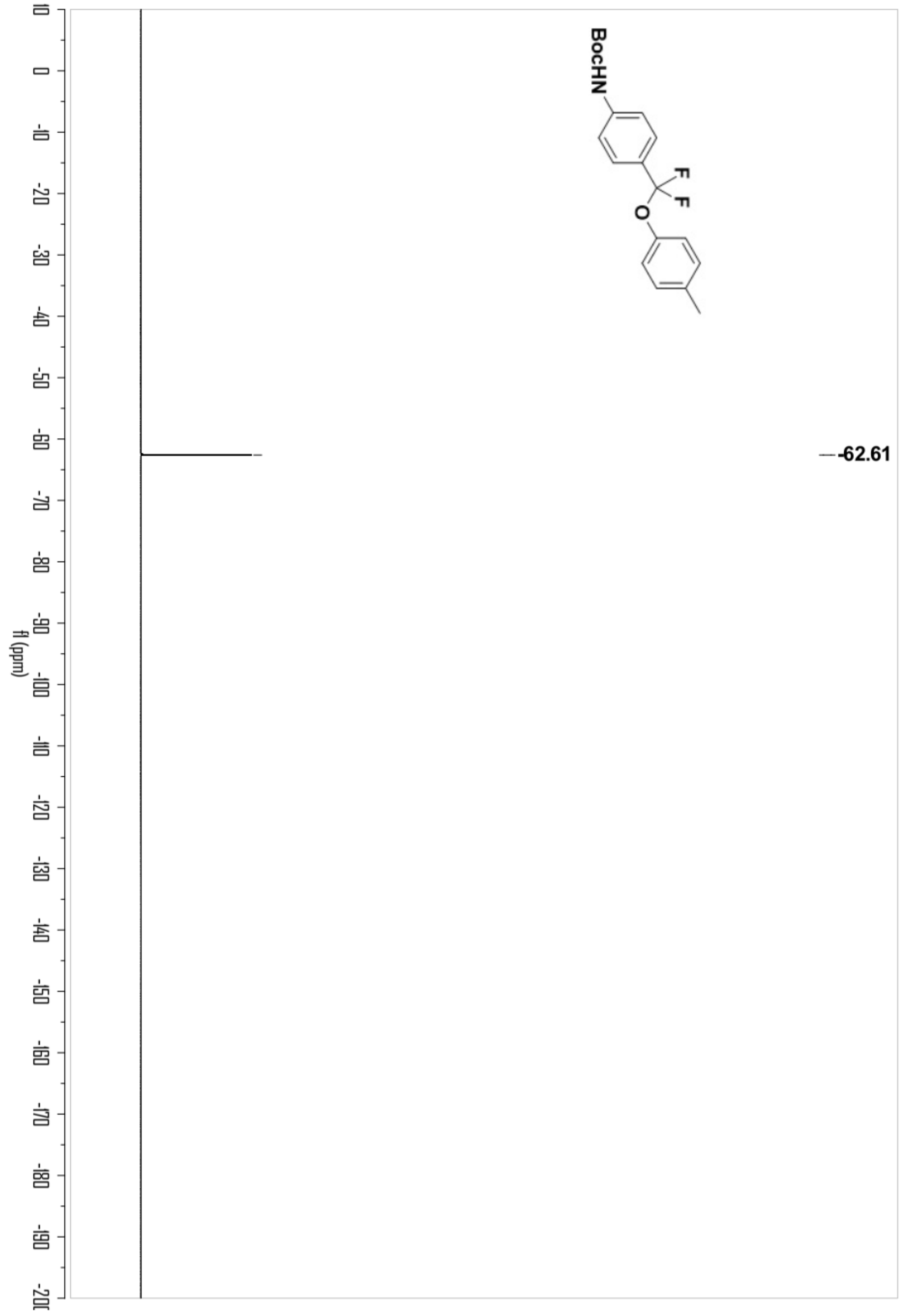
BocHN
F
F
O
-62.61
f1 (ppm)

^{1}H, ^{13}C and ^{19}F NMR spectra of compound 3h

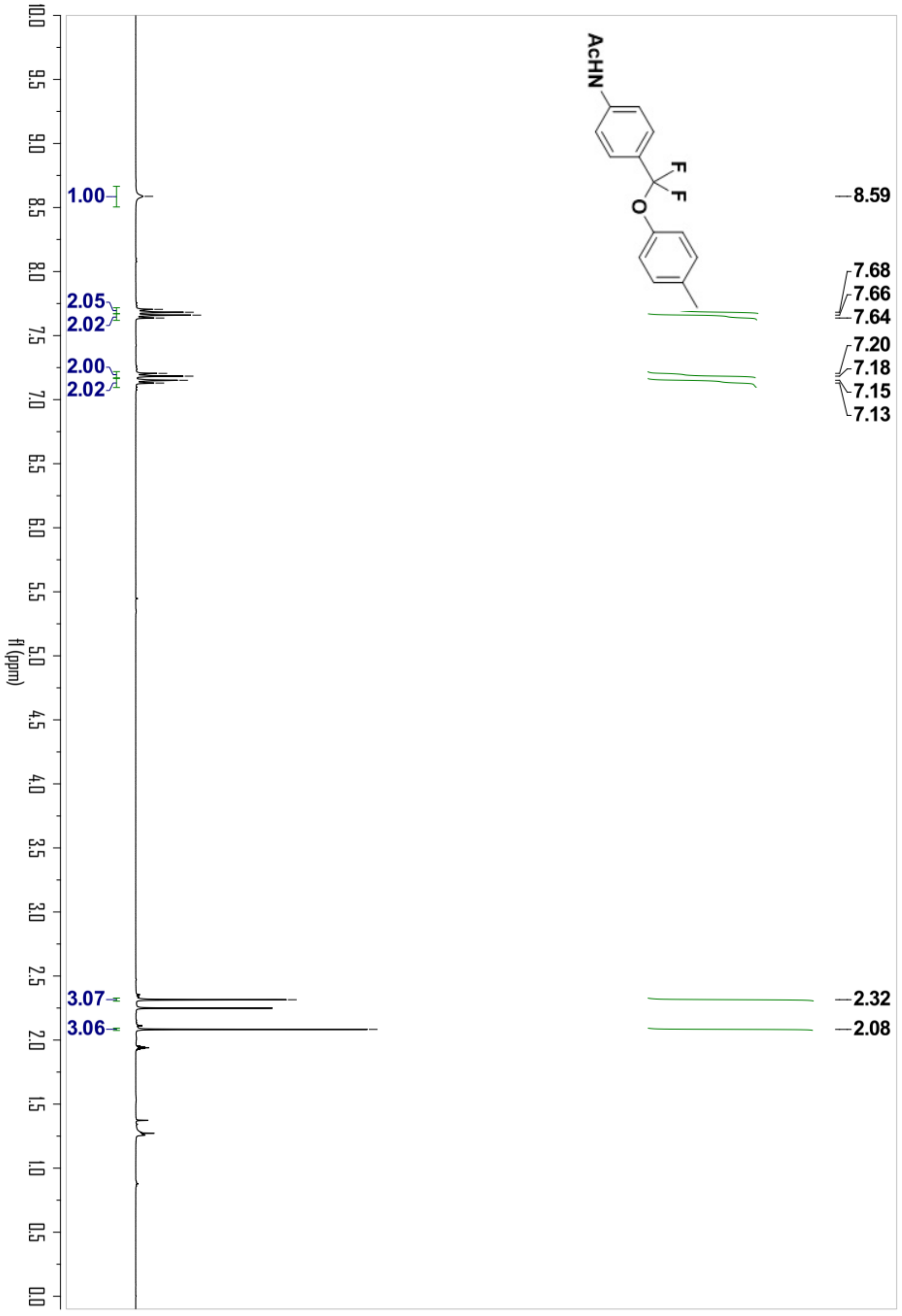

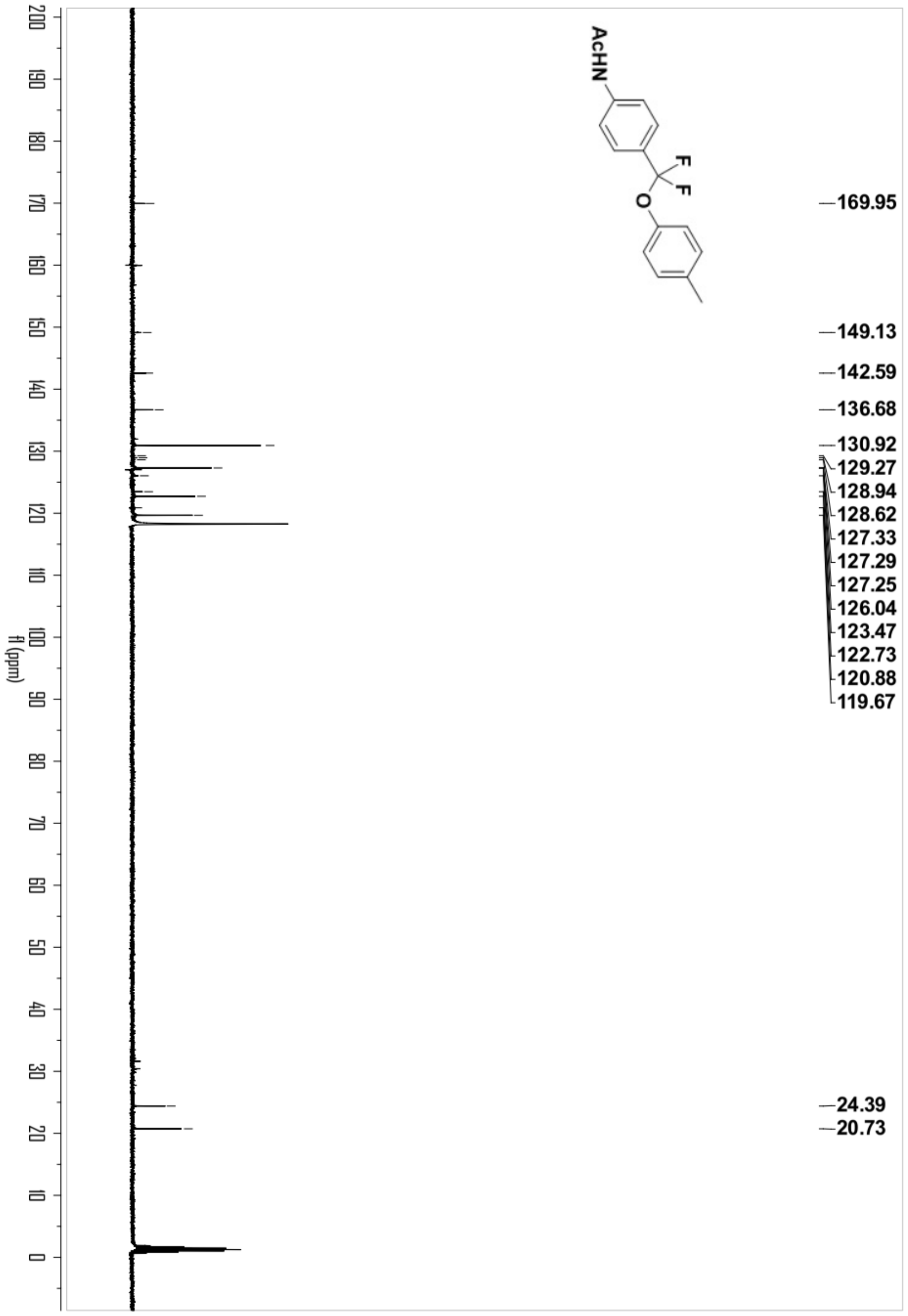
AcHN
F
F
O
169.95
149.13
142.59
136.68
130.92
129.27
128.94
128.62
127.33
127.29
127.25
126.04
123.47
122.73
120.88
119.67
24.39
20.73
f1 (ppm)

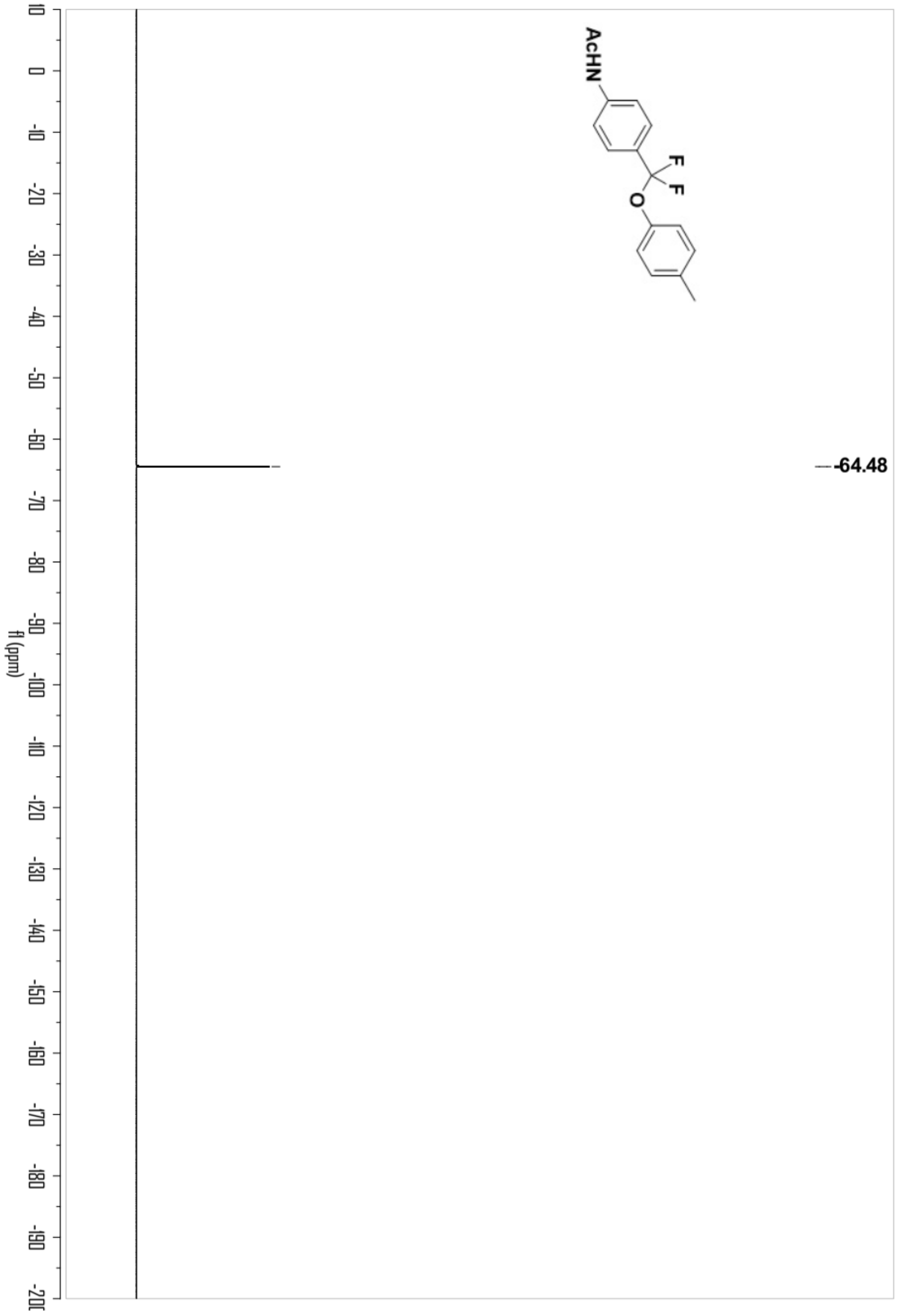
-64.48
AcHN
F
F
O
f1 (ppm)

^{1}H, ^{13}C and ^{19}F NMR spectra of compound 3i

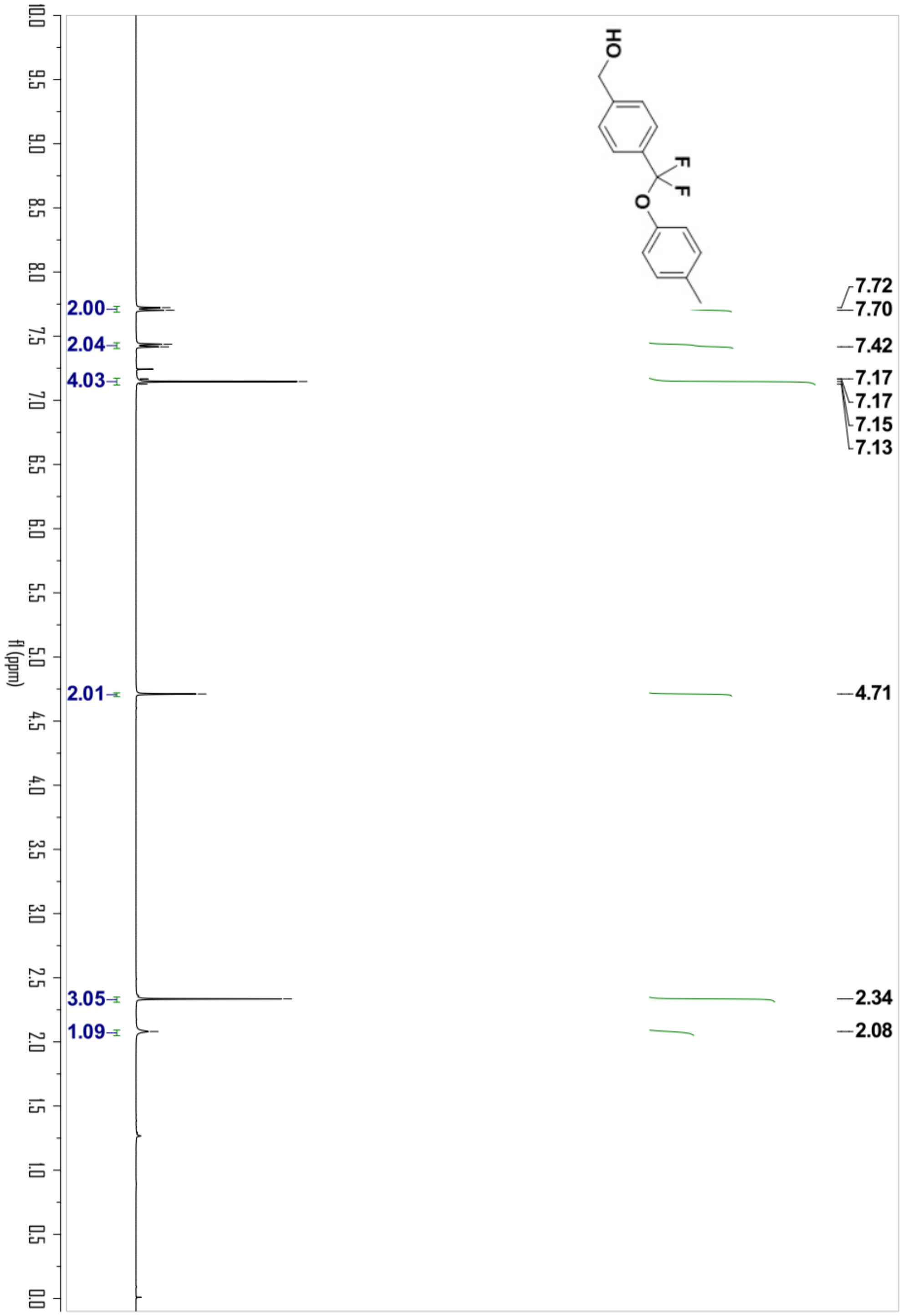

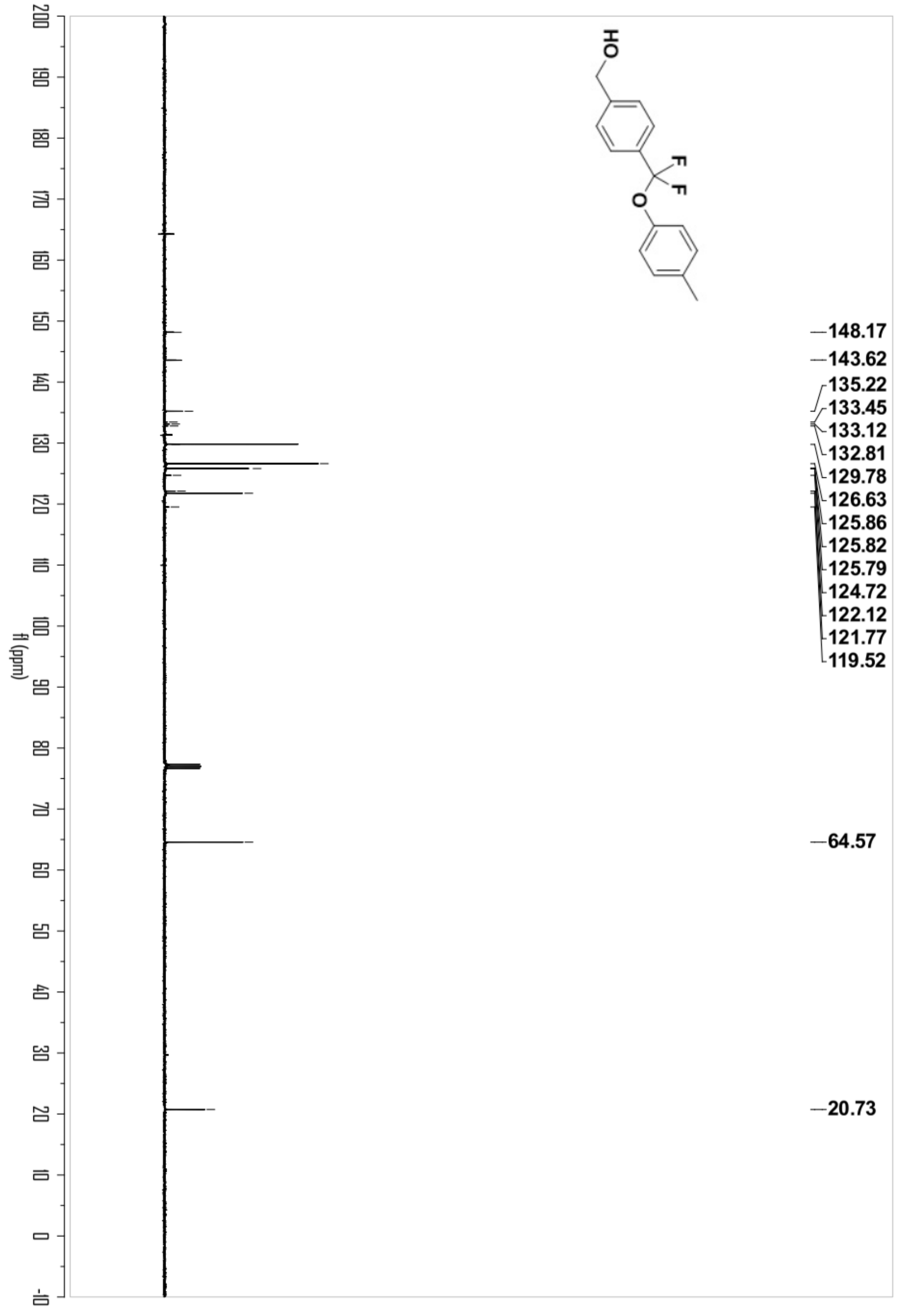
148.17
143.62
135.22
133.45
133.12
132.81
129.78
126.63
125.86
125.82
125.79
124.72
122.12
121.77
119.52
64.57
20.73
f1 (ppm)
HO
F
F
O

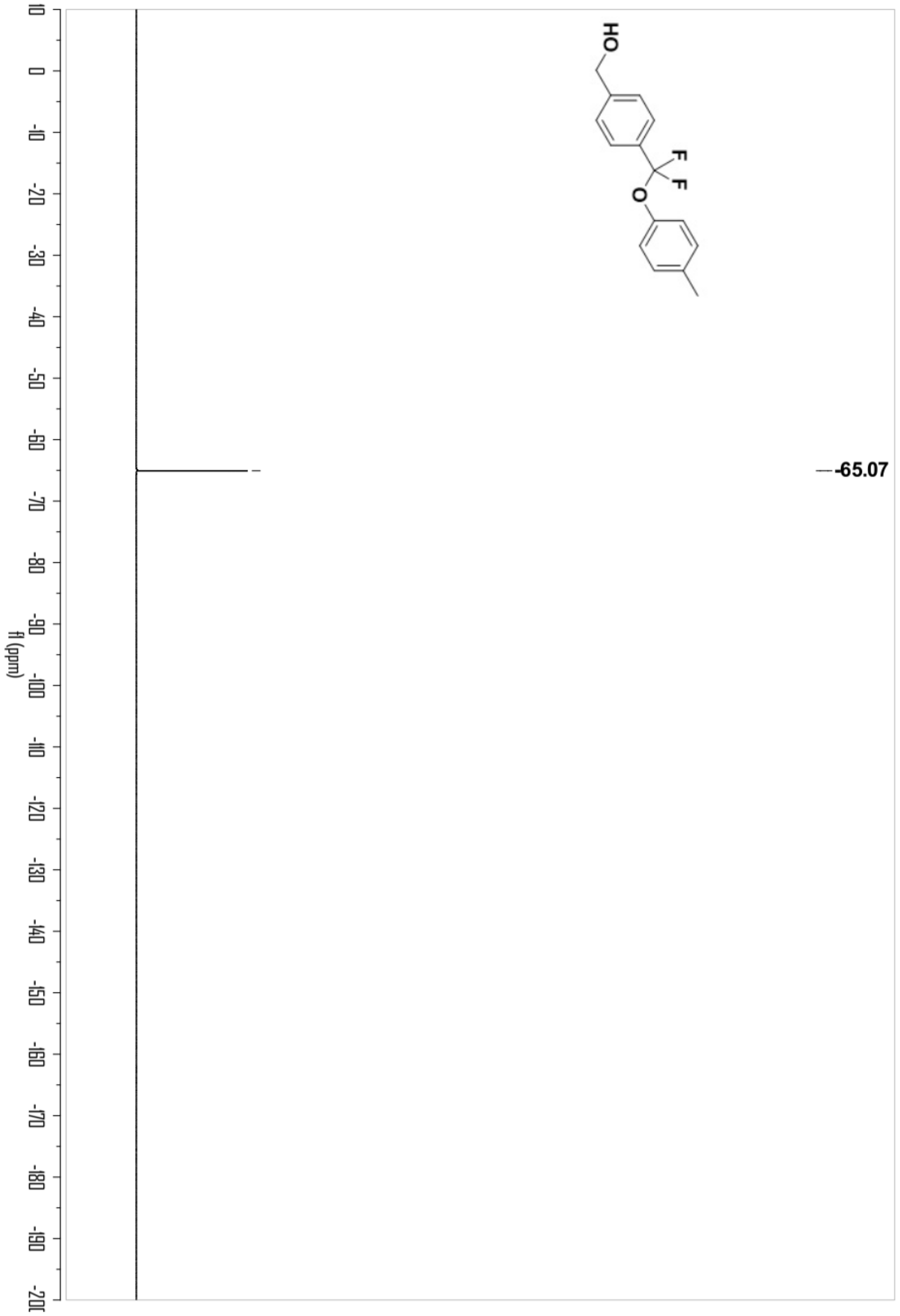
-65.07
f1 (ppm)
HO
F
F
O

^{1}H, ^{13}C and ^{19}F NMR spectra of compound 3j

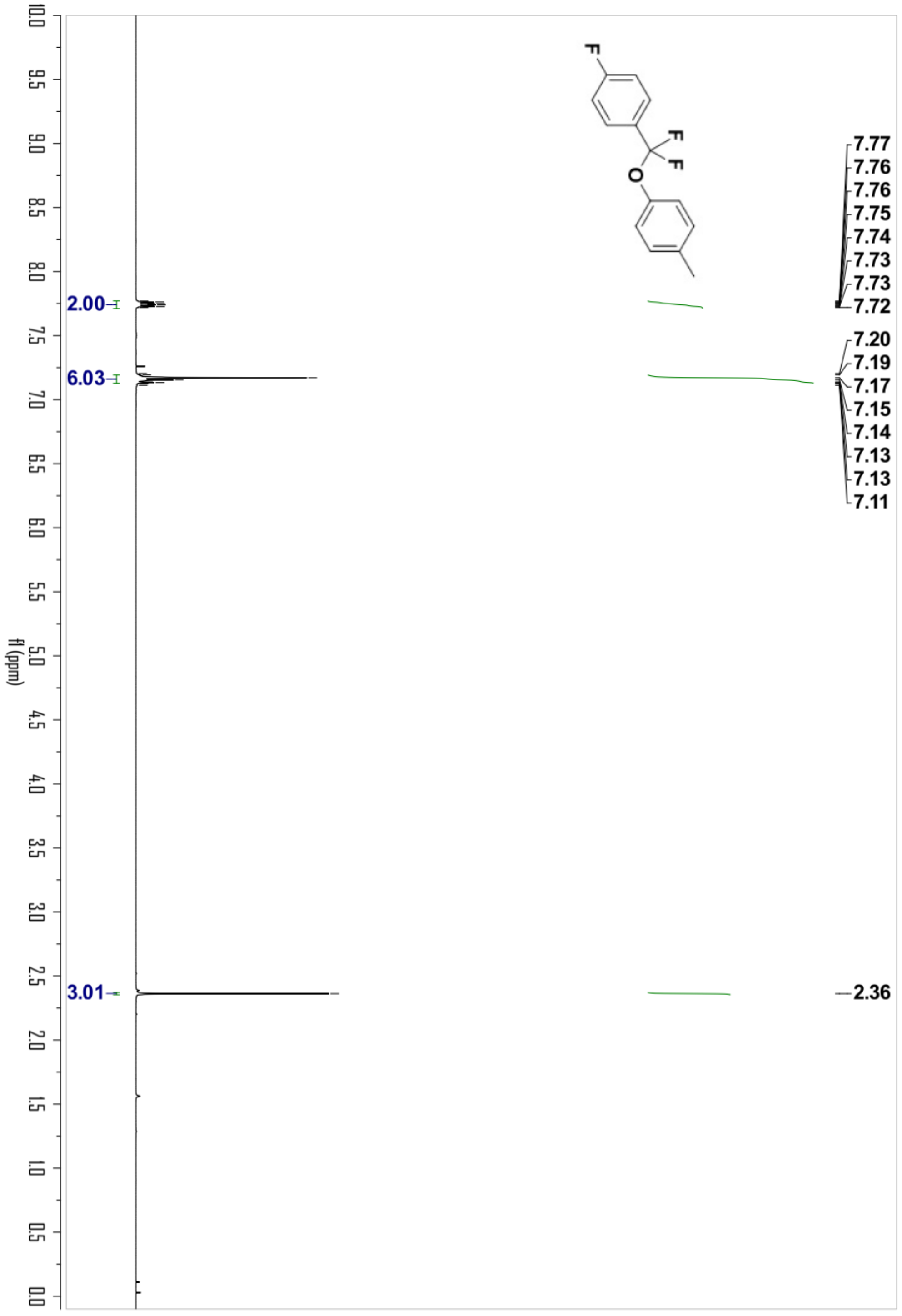

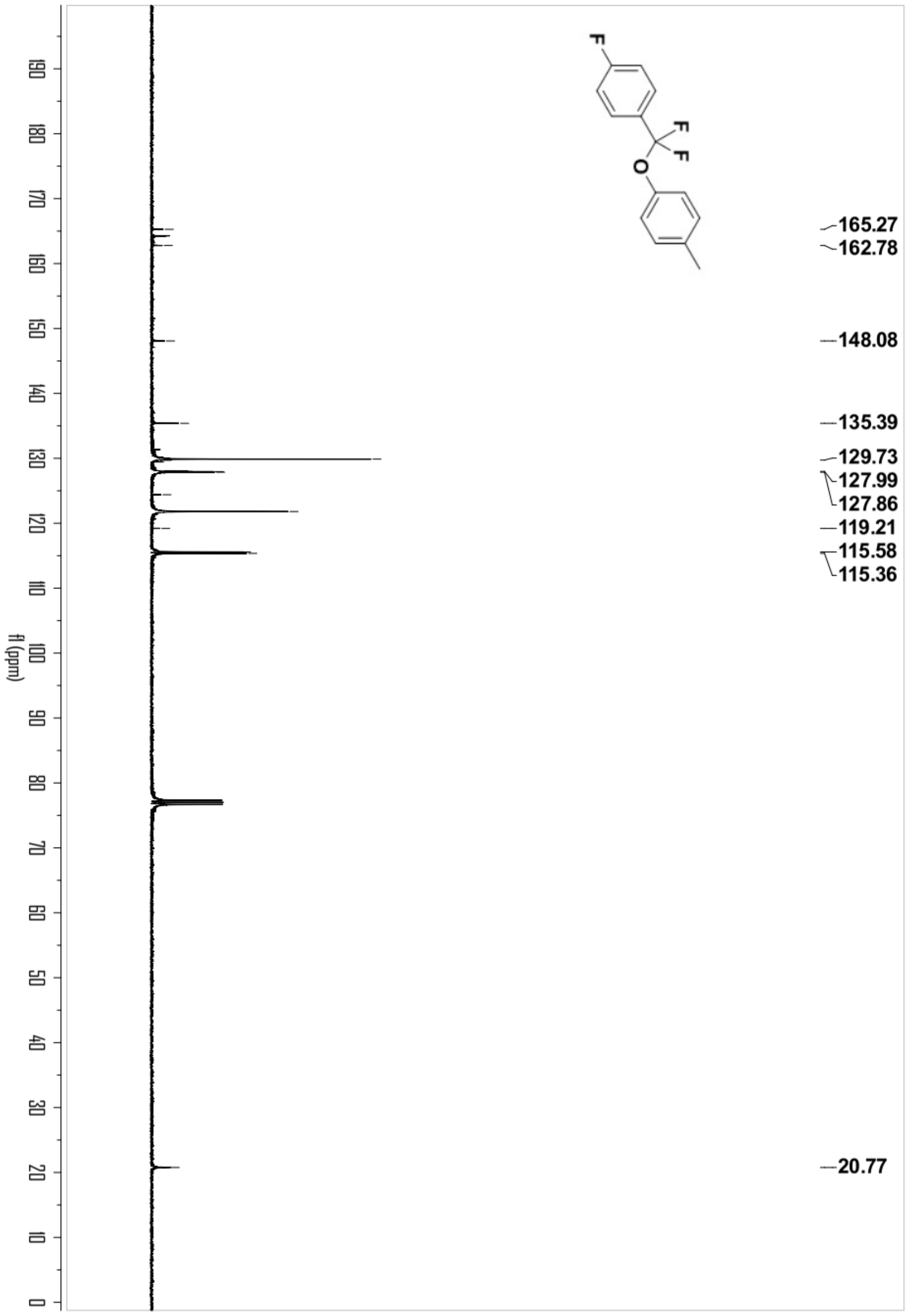
165.27
162.78
148.08
135.39
129.73
127.99
127.86
119.21
115.58
115.36
20.77
f1 (ppm)

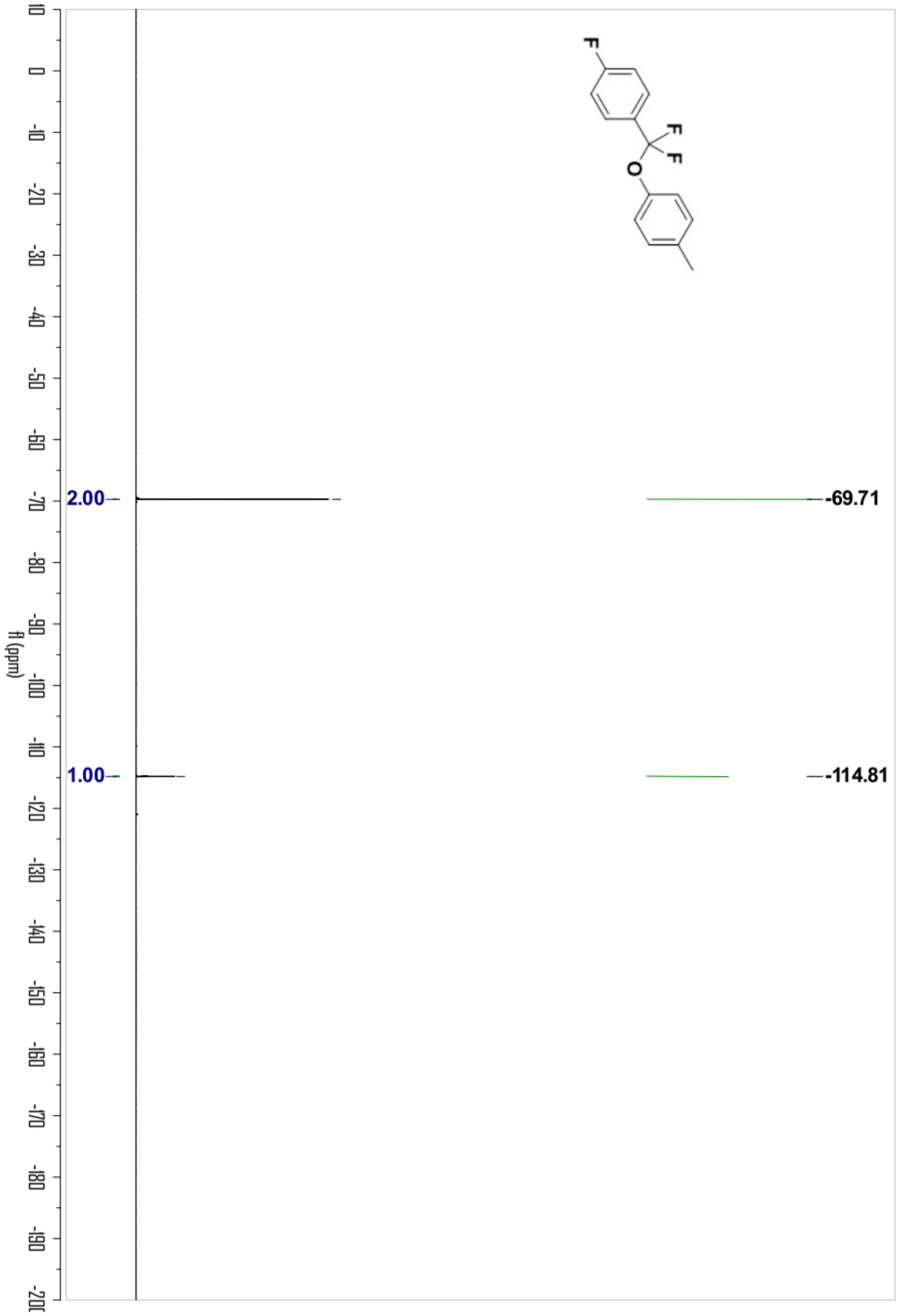

-69.71
-114.81
2.00
1.00
f1 (ppm)

^{1}H, ^{13}C and ^{19}F NMR spectra of compound 3k

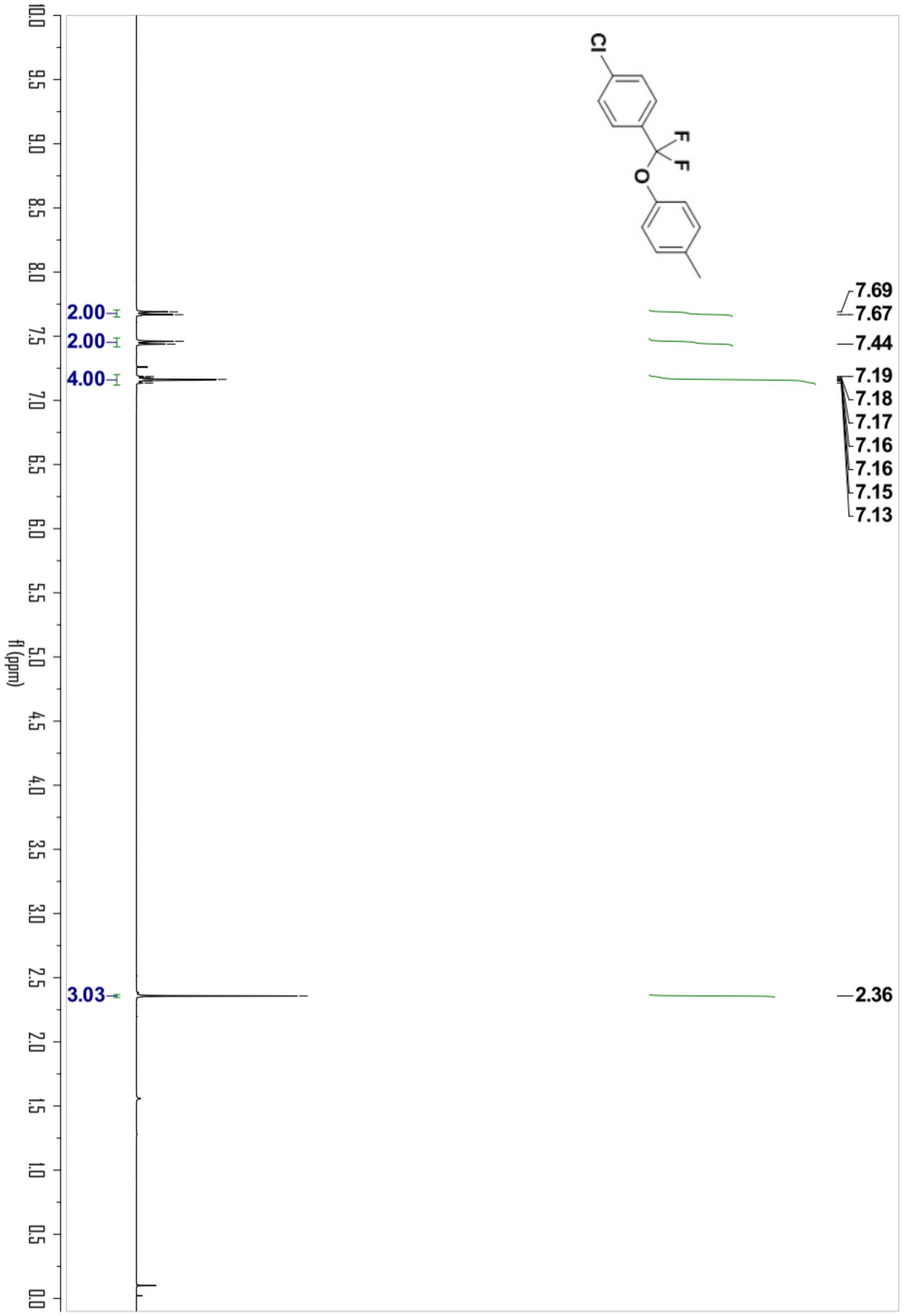

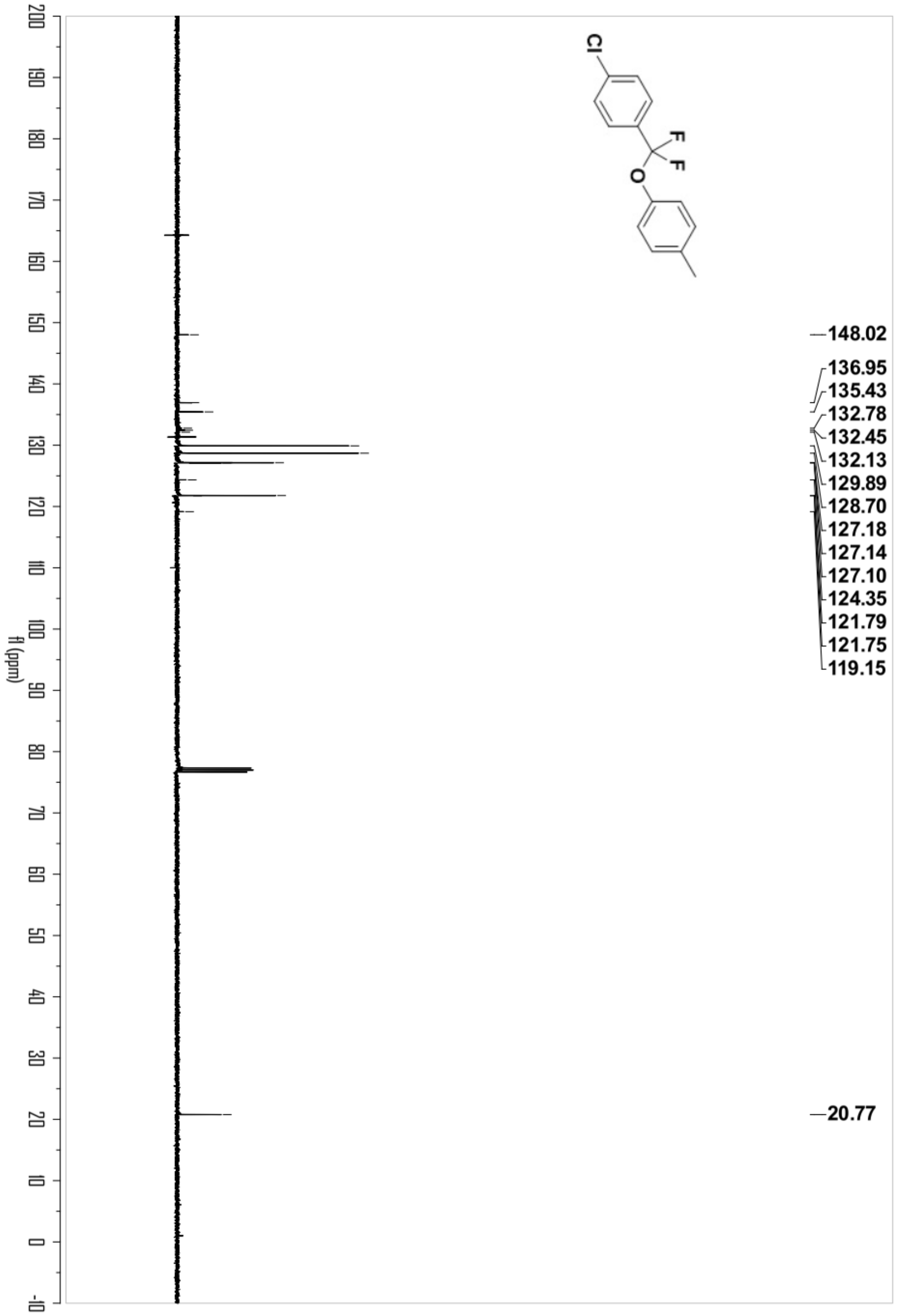
Cl
F
F
O
148.02
136.95
135.43
132.78
132.45
132.13
129.89
128.70
127.18
127.14
127.10
124.35
121.79
121.75
119.15
20.77
f1 (ppm)

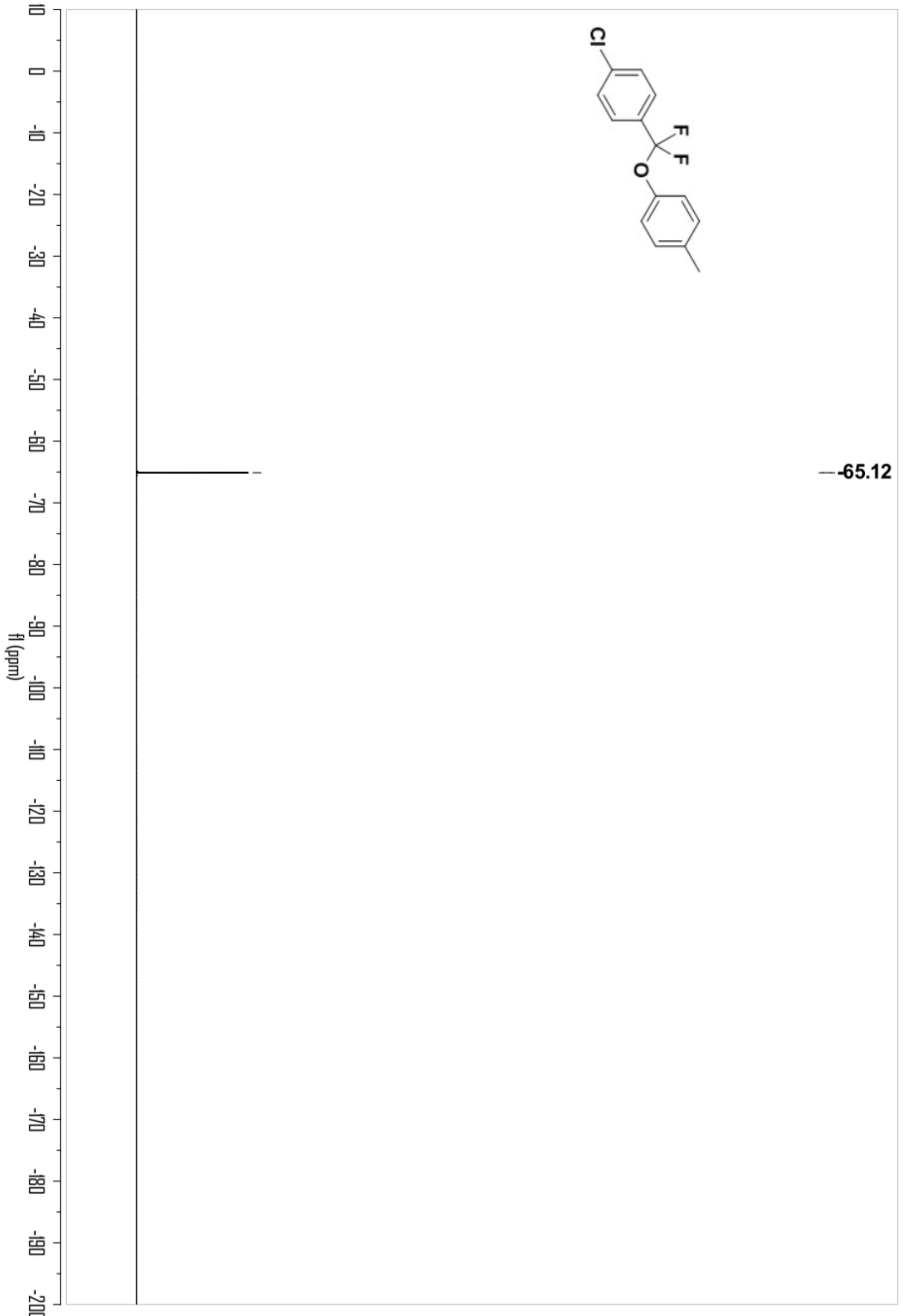
-65.12
f1 (ppm)
Cl
F
F
O

^{1}H, ^{13}C and ^{19}F NMR spectra of compound 3l

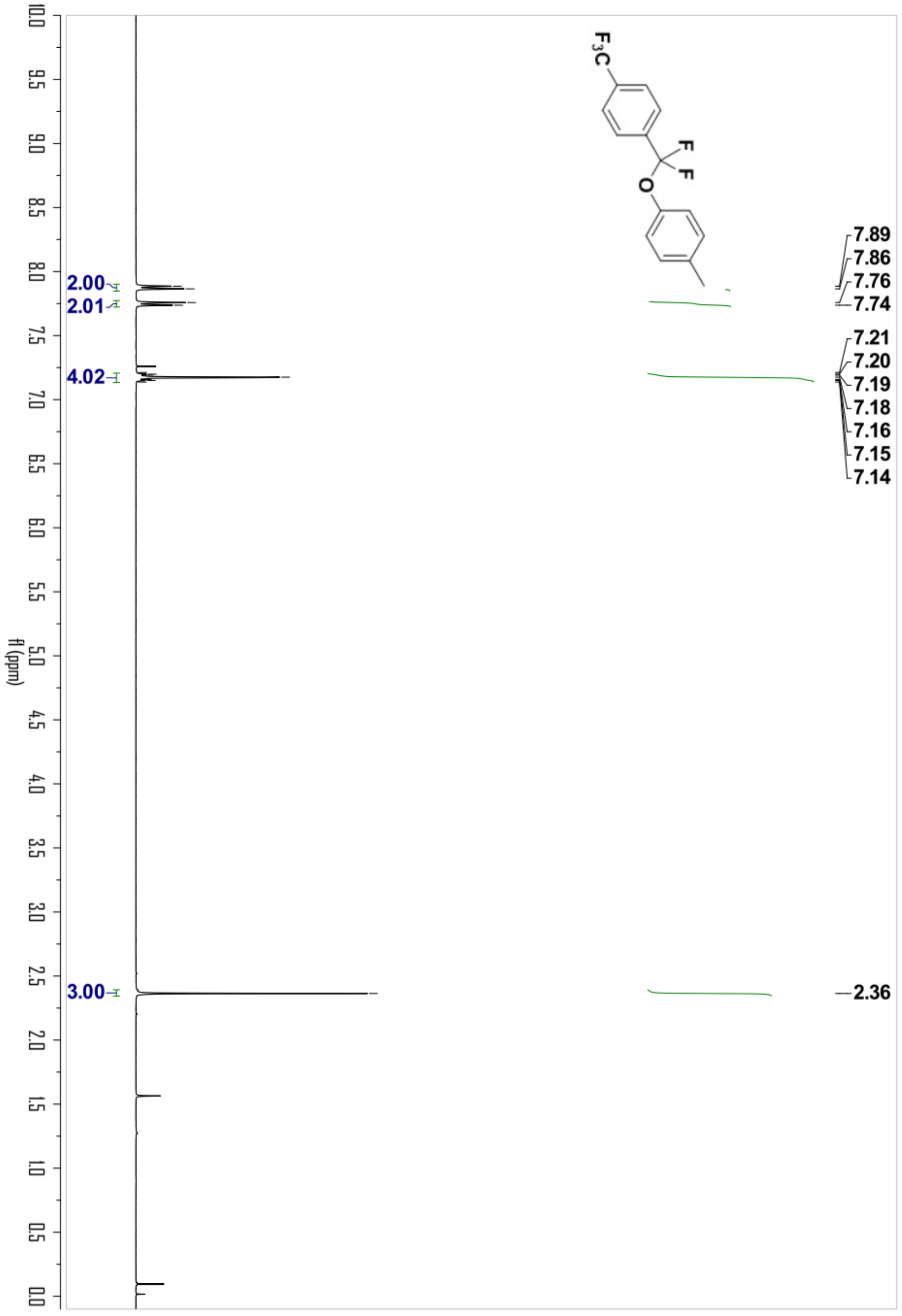

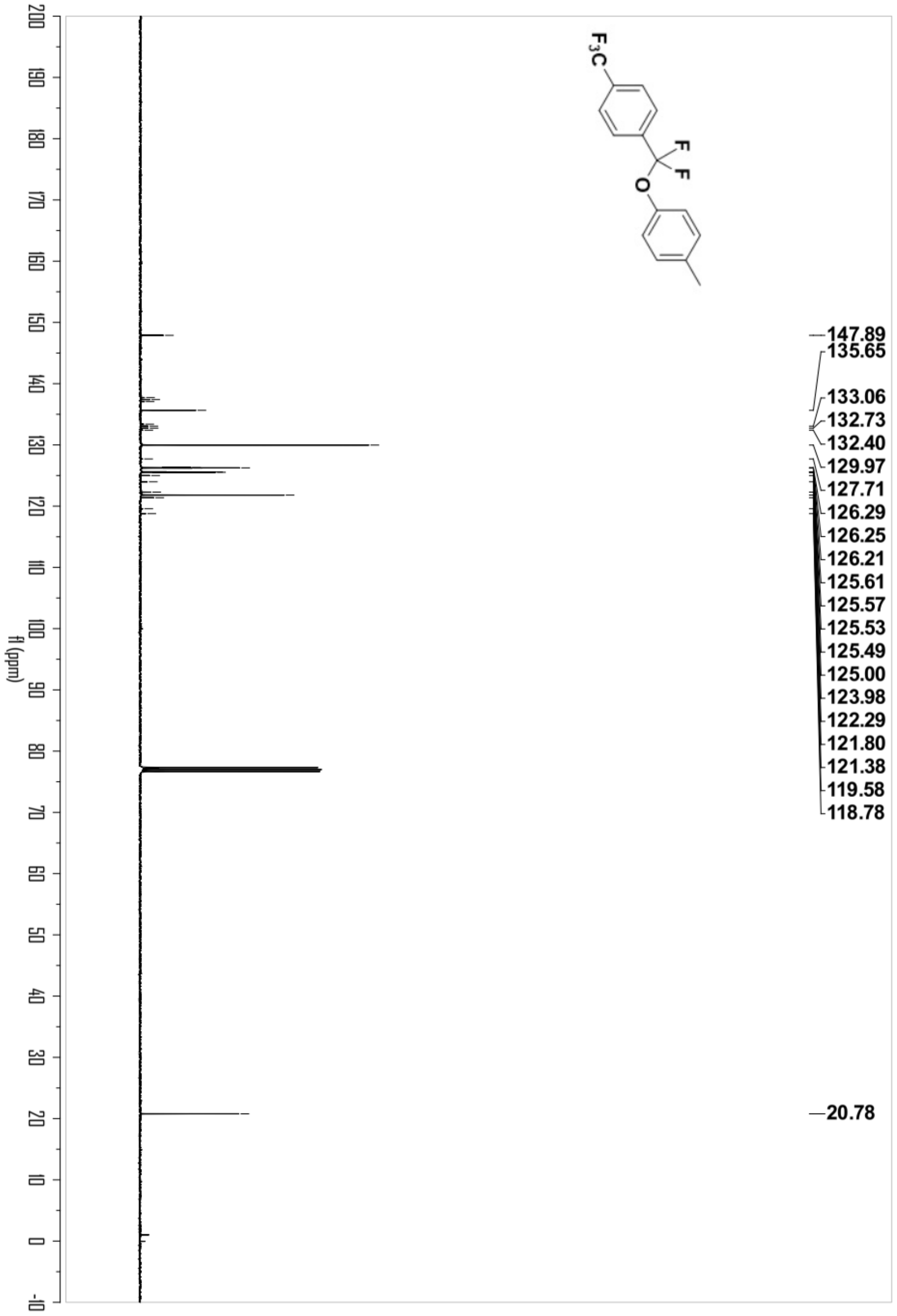

F_3C
F
F
O
147.89
135.65
133.06
132.73
132.40
129.97
127.71
126.29
126.25
126.21
125.61
125.57
125.53
125.49
125.00
123.98
122.29
121.80
121.38
119.58
118.78
20.78
f1 (ppm)

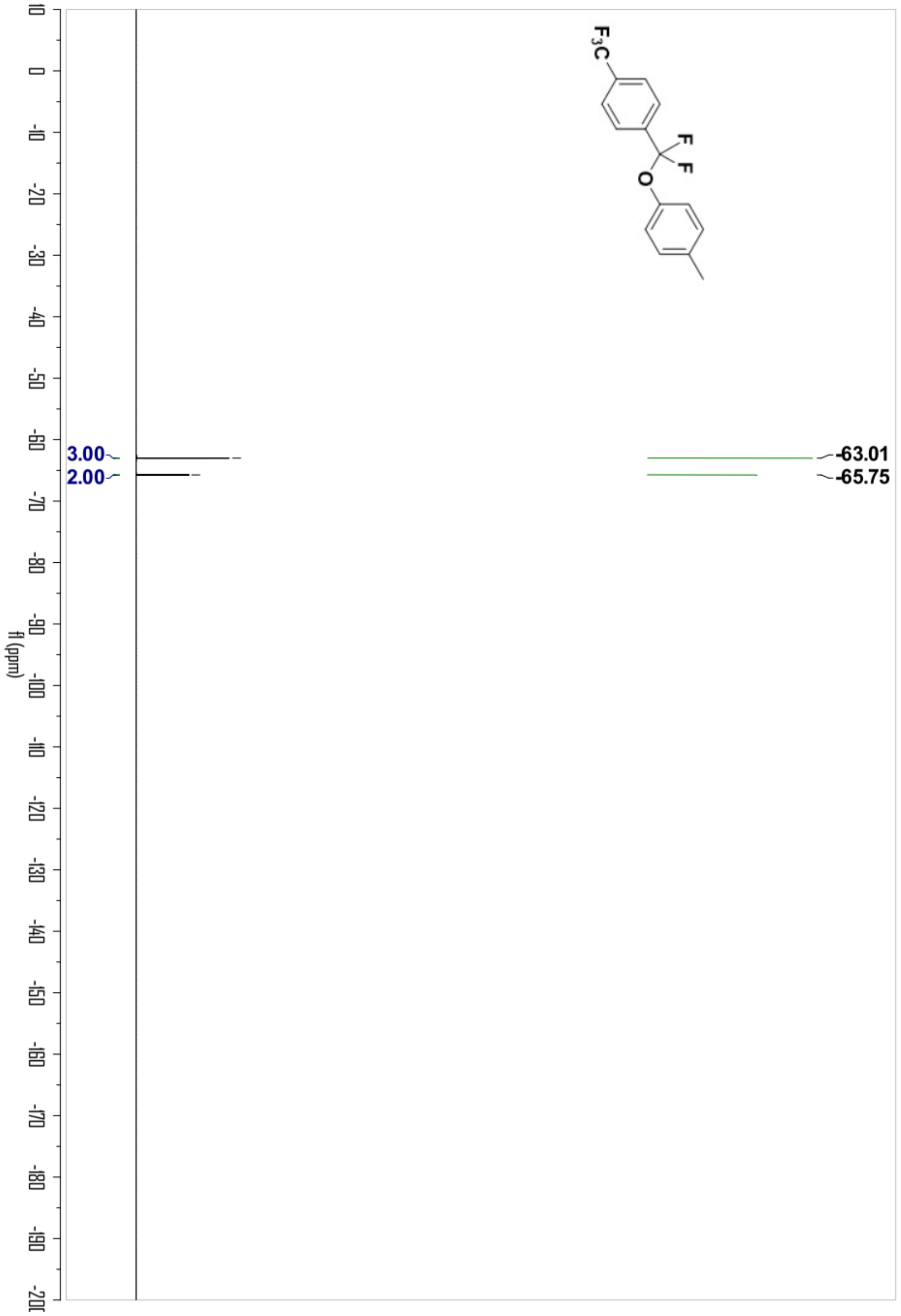
3.00
2.00
-63.01
-65.75
f1 (ppm)

^{1}H, ^{13}C and ^{19}F NMR spectra of compound 3m

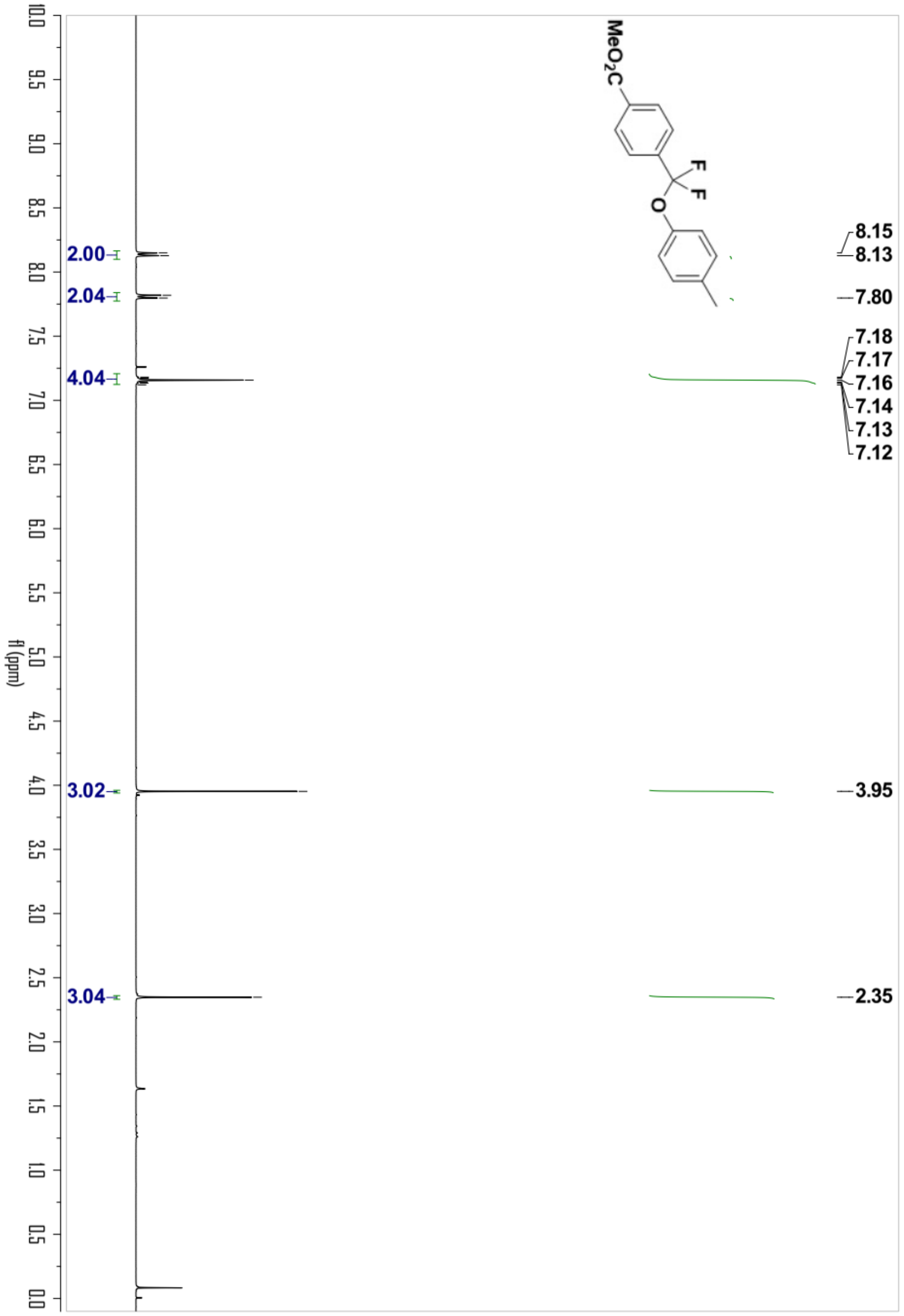

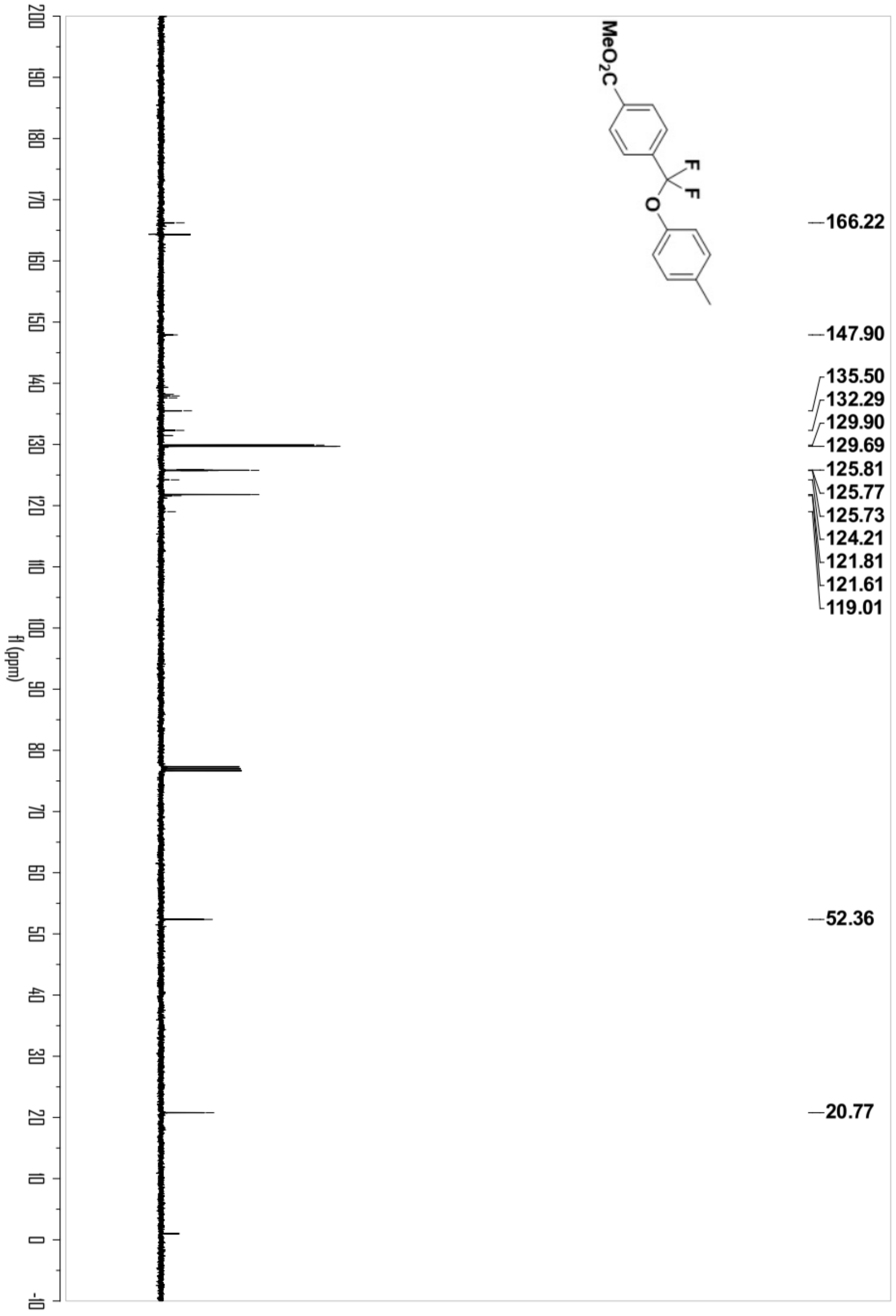
MeO2C
F
F
O
166.22
147.90
135.50
132.29
129.90
129.69
125.81
125.77
125.73
124.21
121.81
121.61
119.01
52.36
20.77
f1 (ppm)
200
190
180
170
160
150
140
130
120
110
100
90
80
70
60
50
40
30
20
10
0
-10

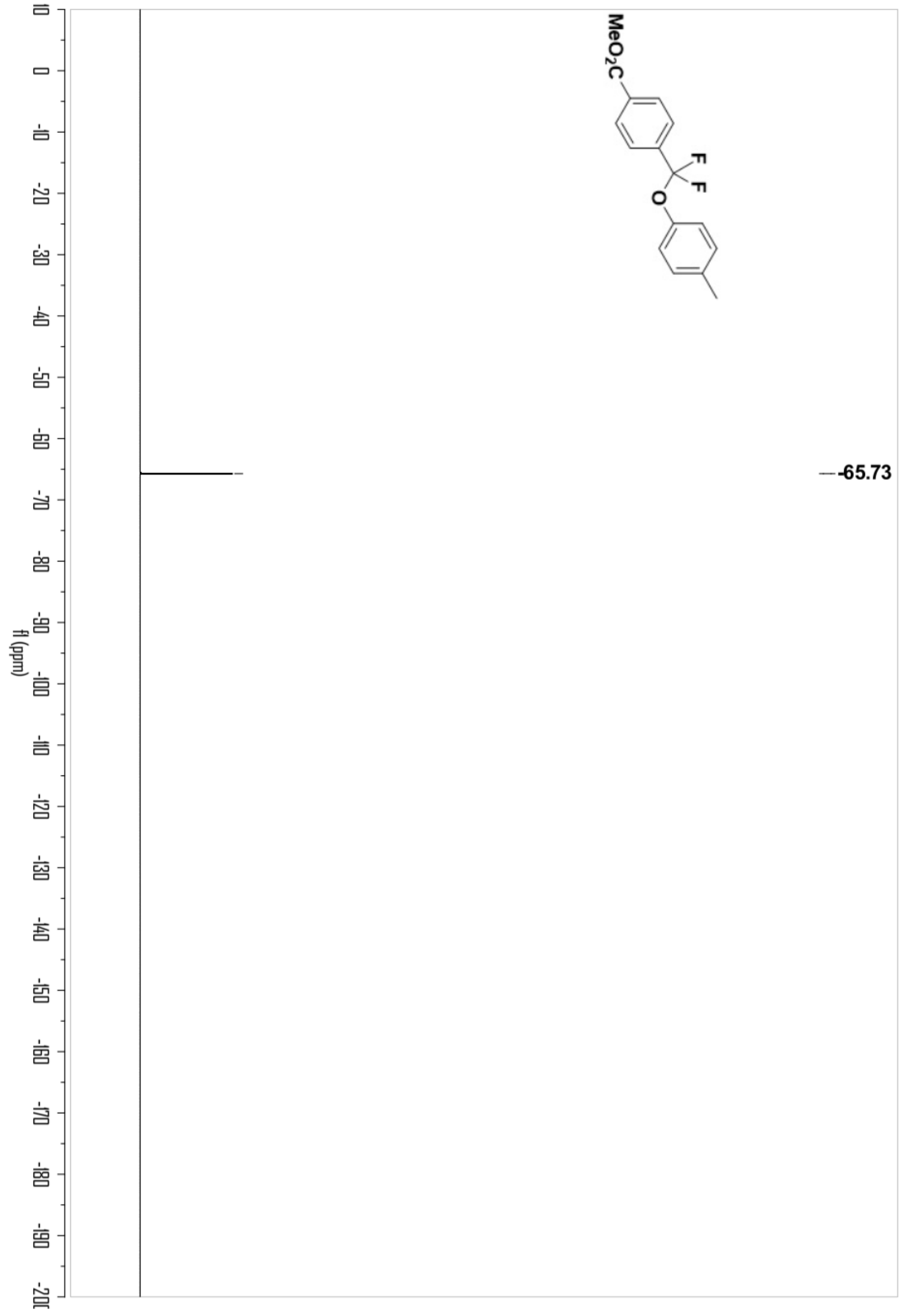
MeO2C
F
F
O
-65.73
f1 (ppm)

^{1}H, ^{13}C and ^{19}F NMR spectra of compound 3n

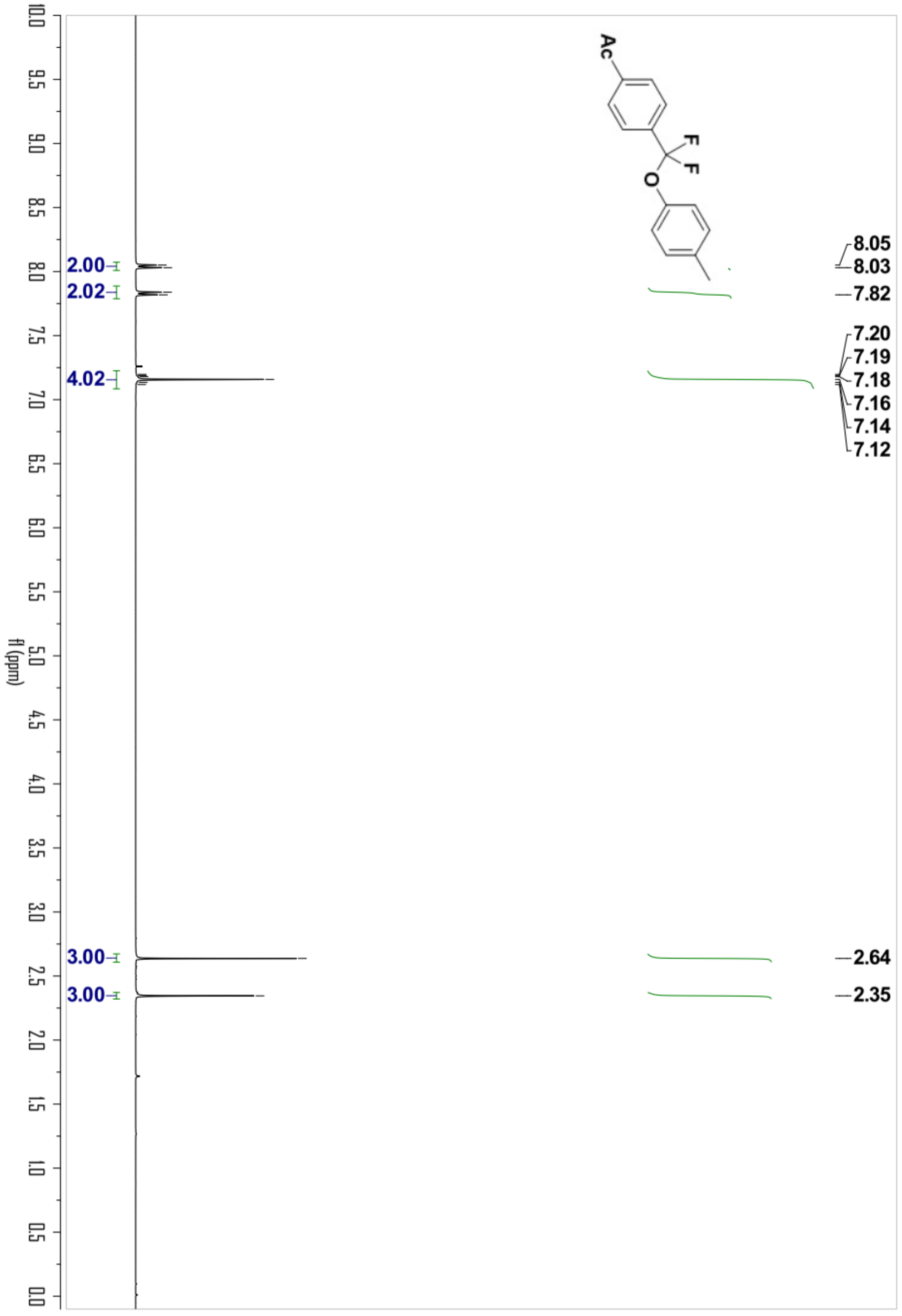

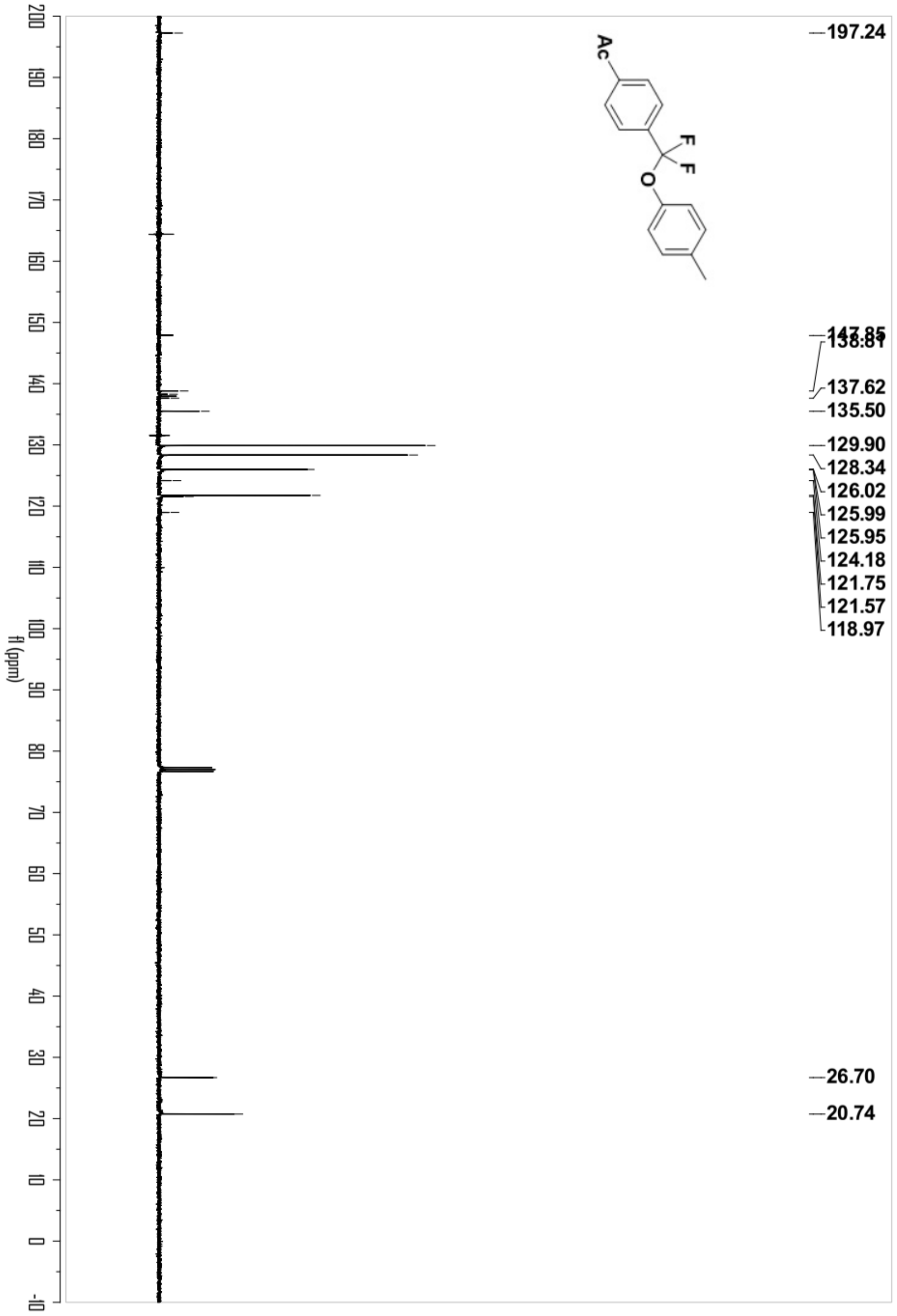
197.24
147.85
138.81
137.62
135.50
129.90
128.34
126.02
125.99
125.95
124.18
121.75
121.57
118.97
26.70
20.74
Ac
F
F
O
f1 (ppm)

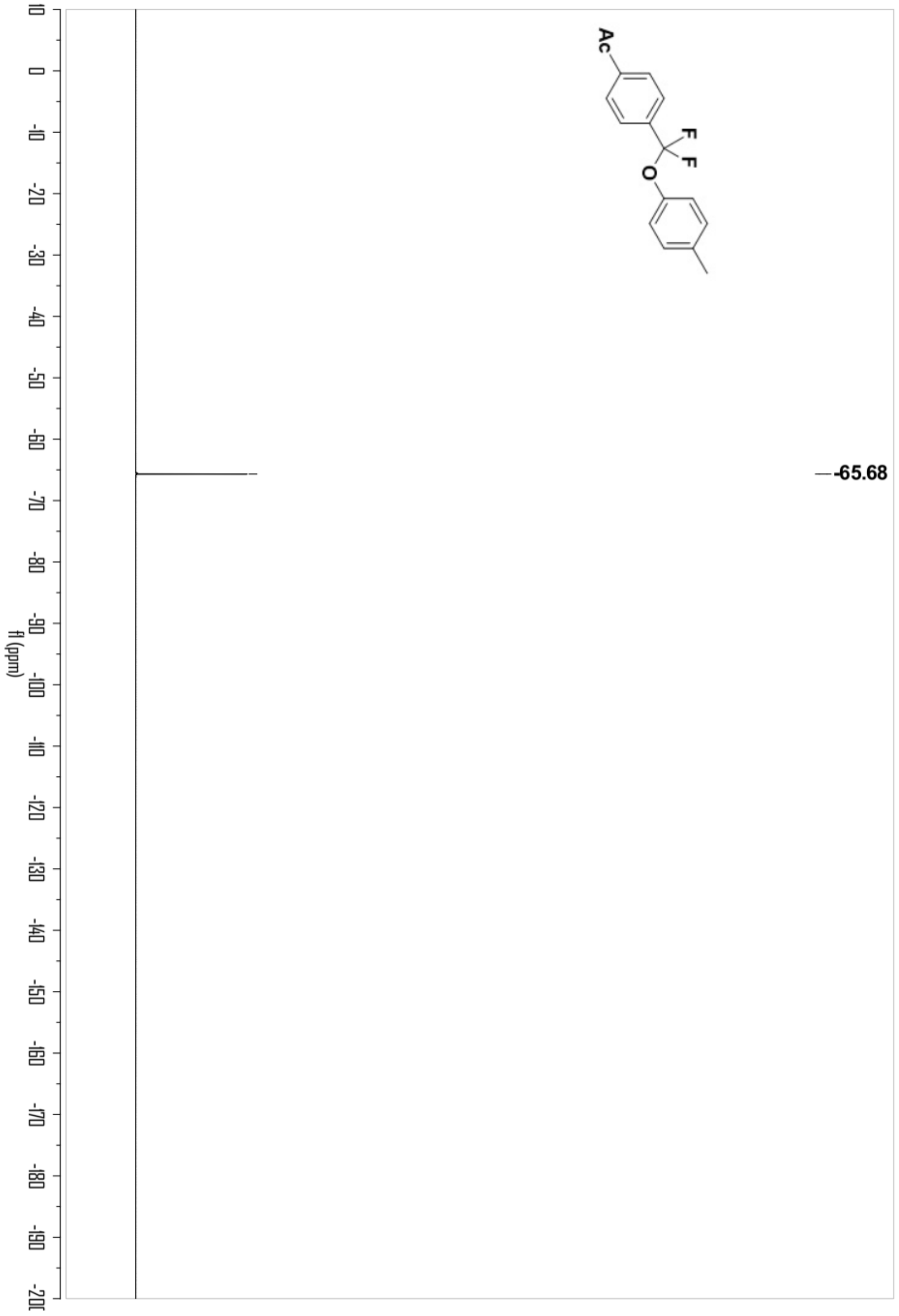
Ac
F
F
O
-65.68
f1 (ppm)

^{1}H, ^{13}C and ^{19}F NMR spectra of compound 30

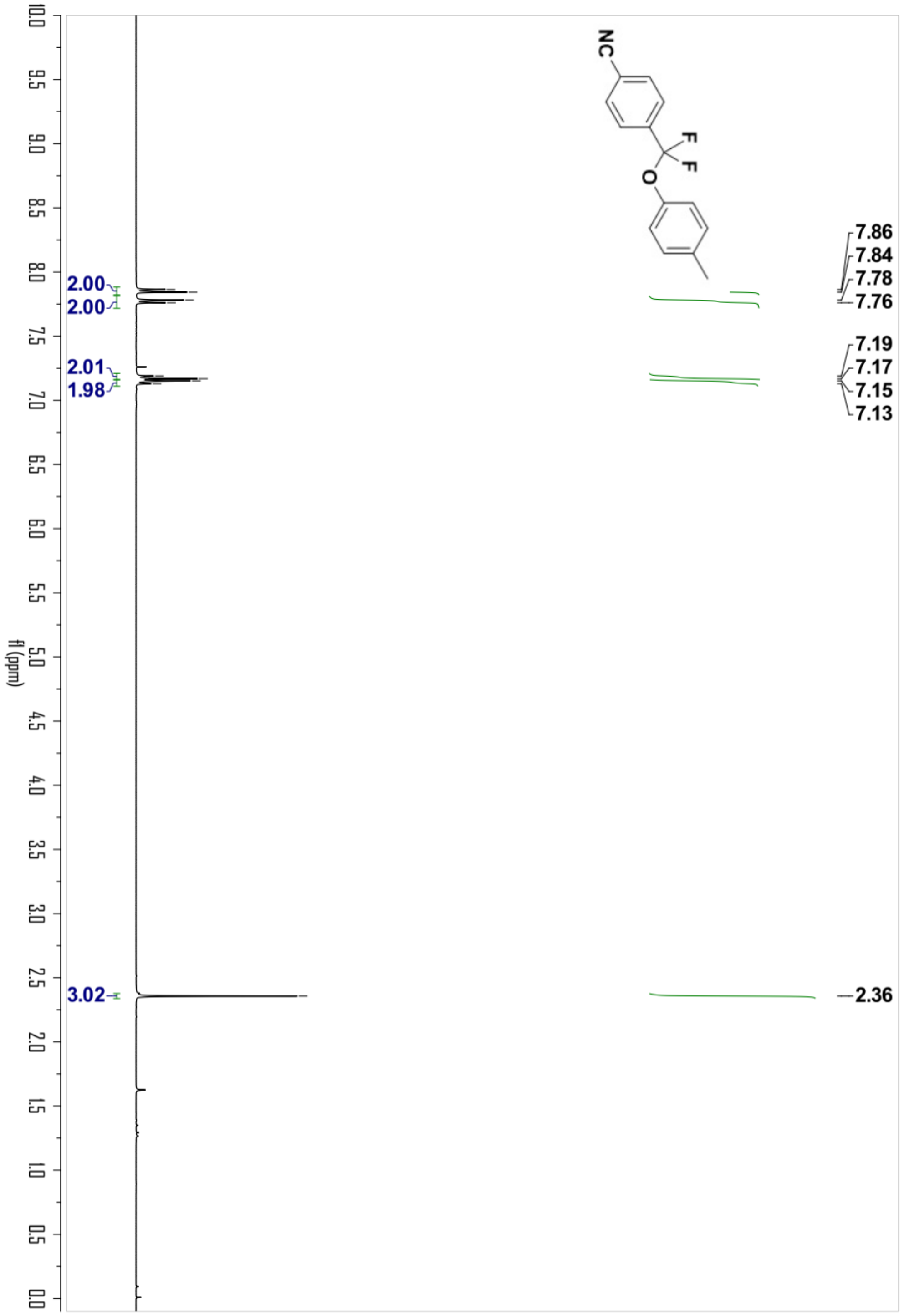

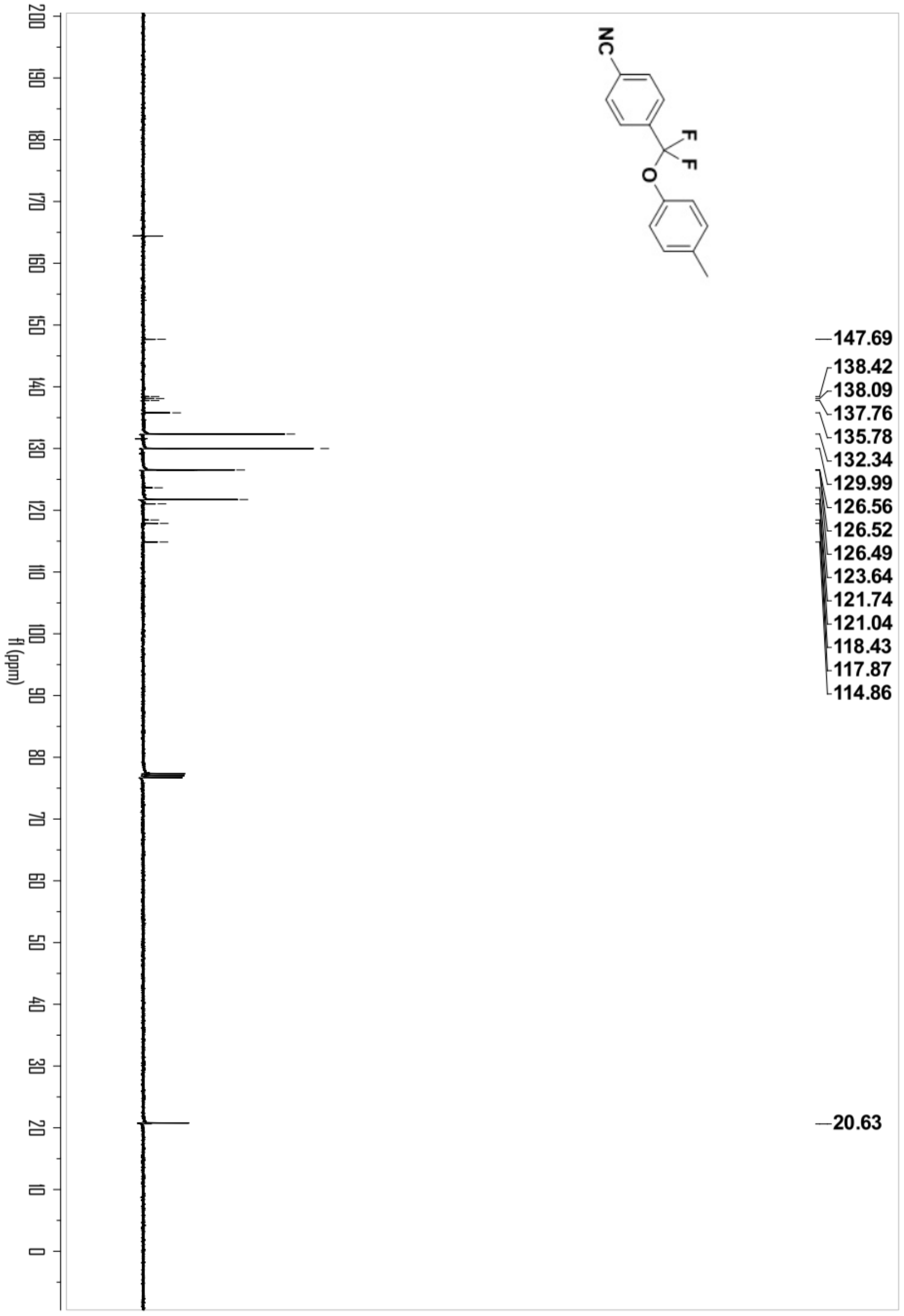

NC
F
F
O
147.69
138.42
138.09
137.76
135.78
132.34
129.99
126.56
126.52
126.49
123.64
121.74
121.04
118.43
117.87
114.86
20.63
f1 (ppm)

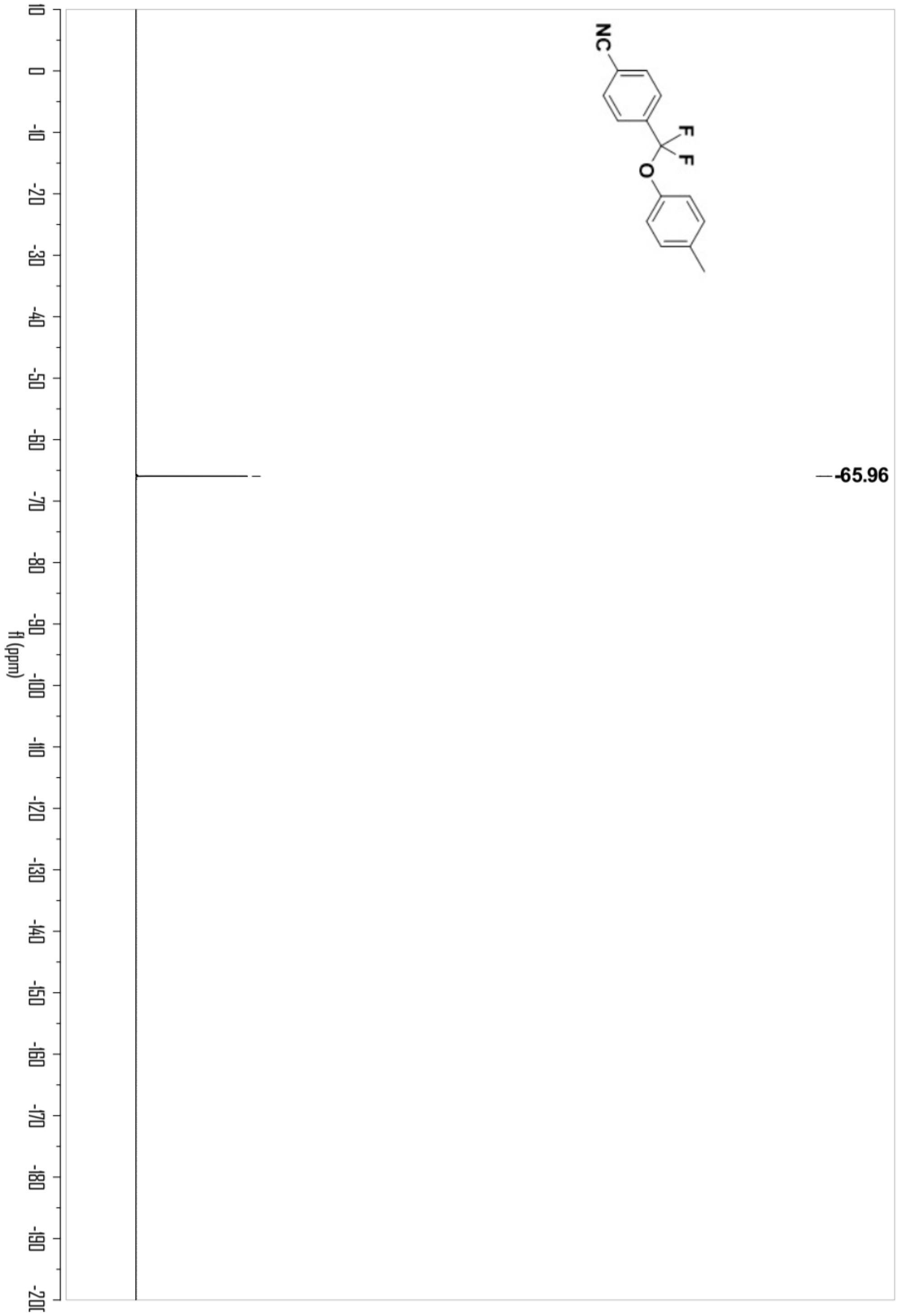
NC
F
F
O
-65.96
f1 (ppm)

^{1}H, ^{13}C and ^{19}F NMR spectra of compound 3p

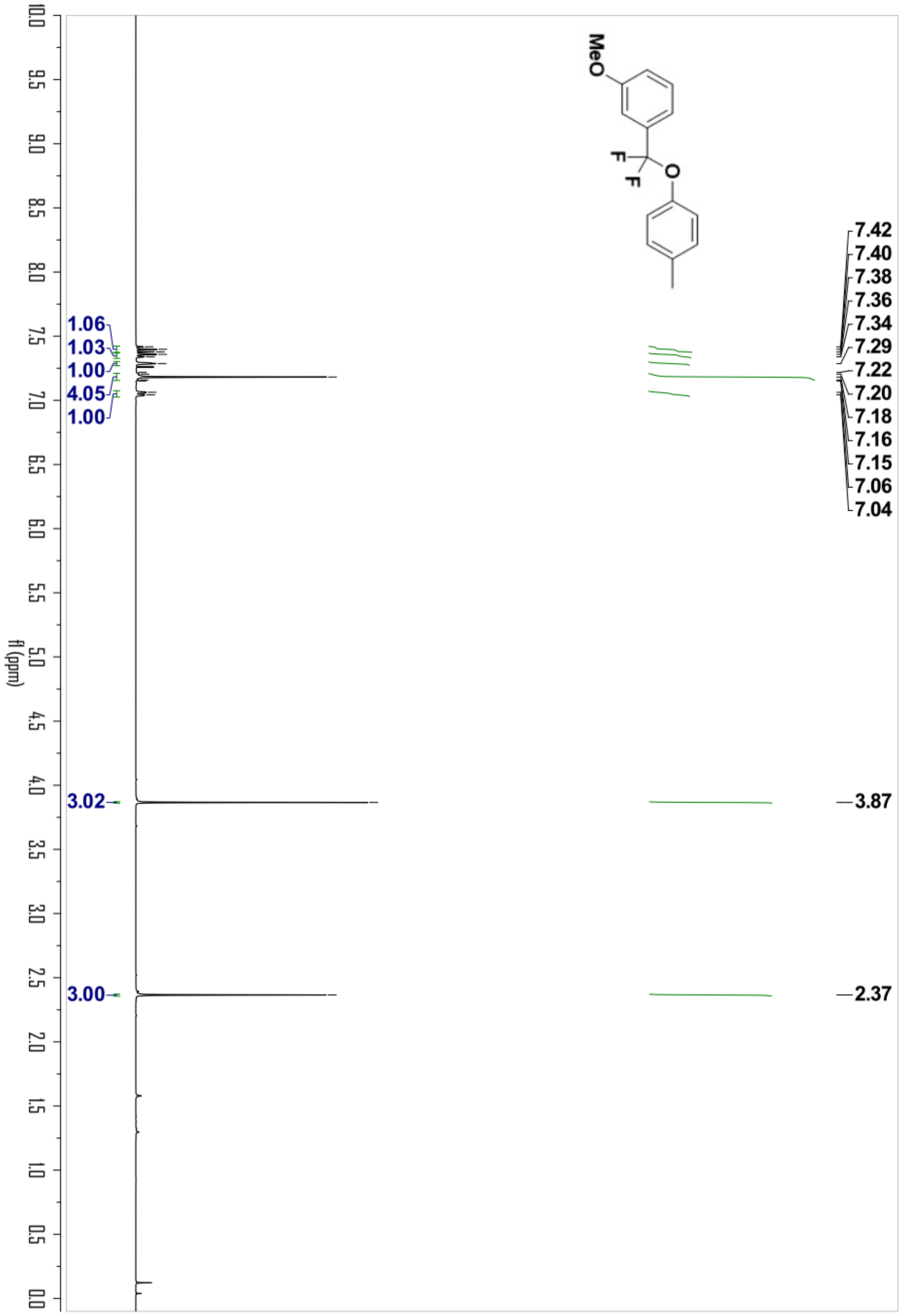

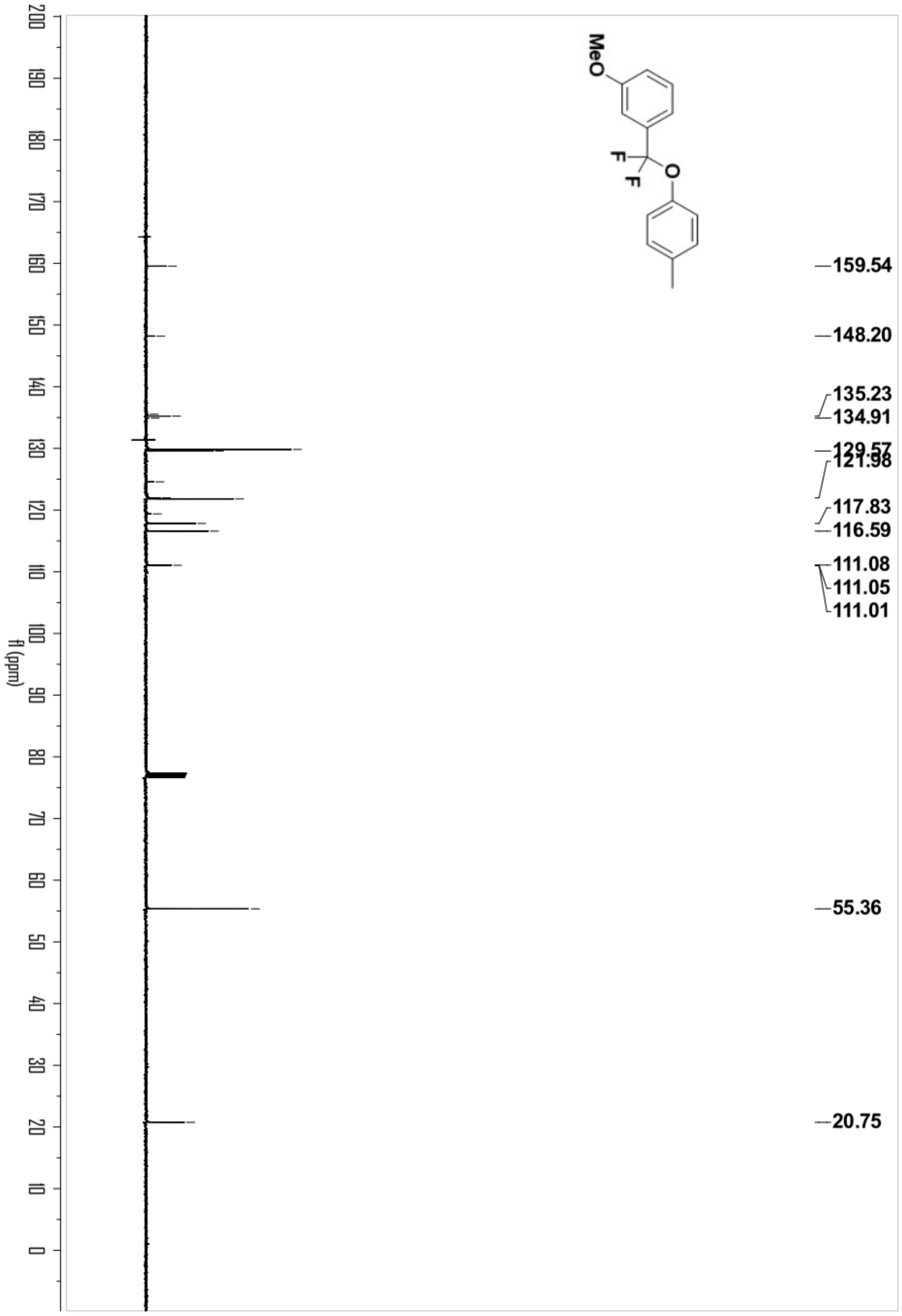
MeO
F
F
O
159.54
148.20
135.23
134.91
129.57
121.98
117.83
116.59
111.08
111.05
111.01
55.36
20.75
f1 (ppm)

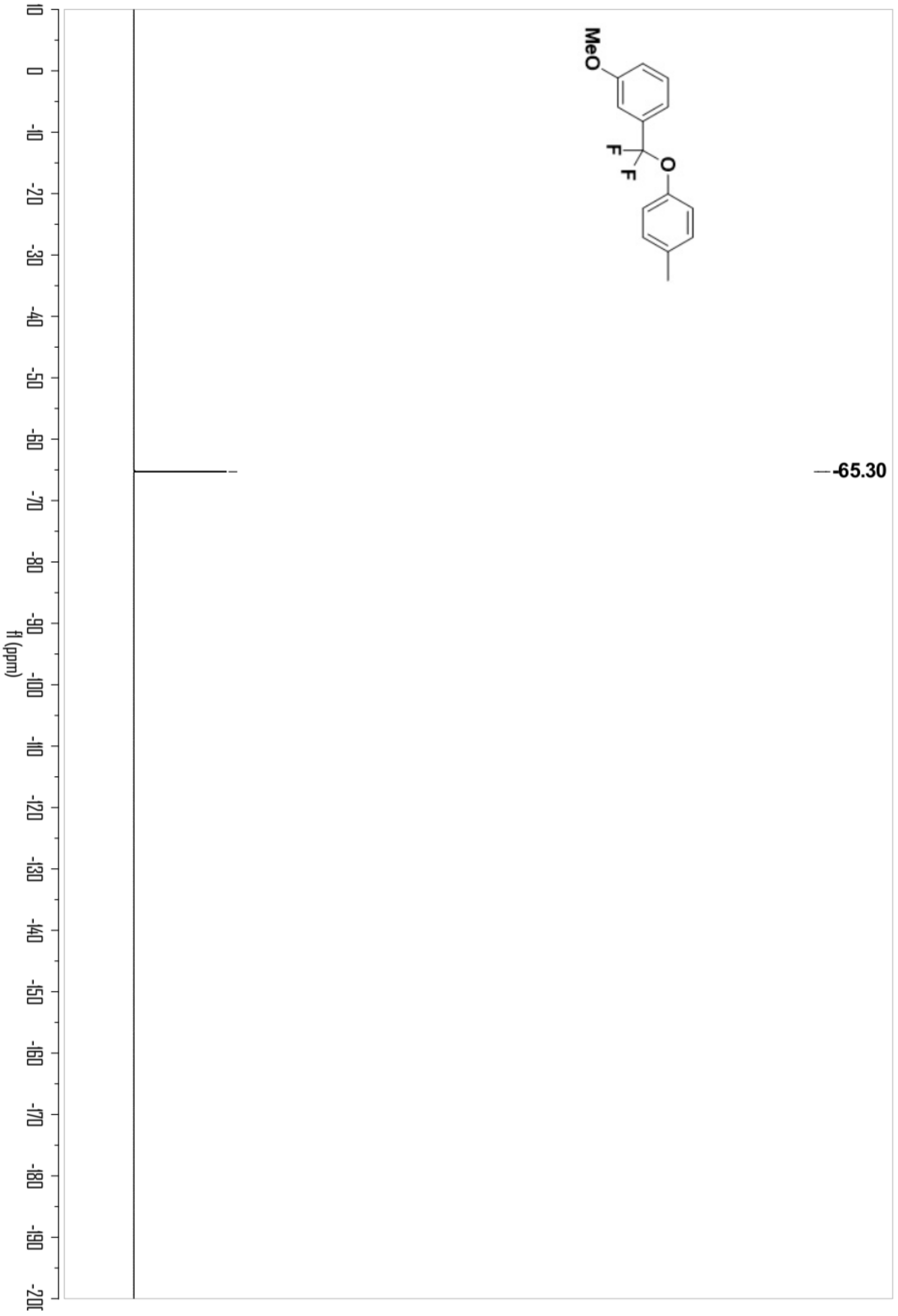
MeO
F
F
O
-65.30
f1 (ppm)

^{1}H, ^{13}C and ^{19}F NMR spectra of compound 3q

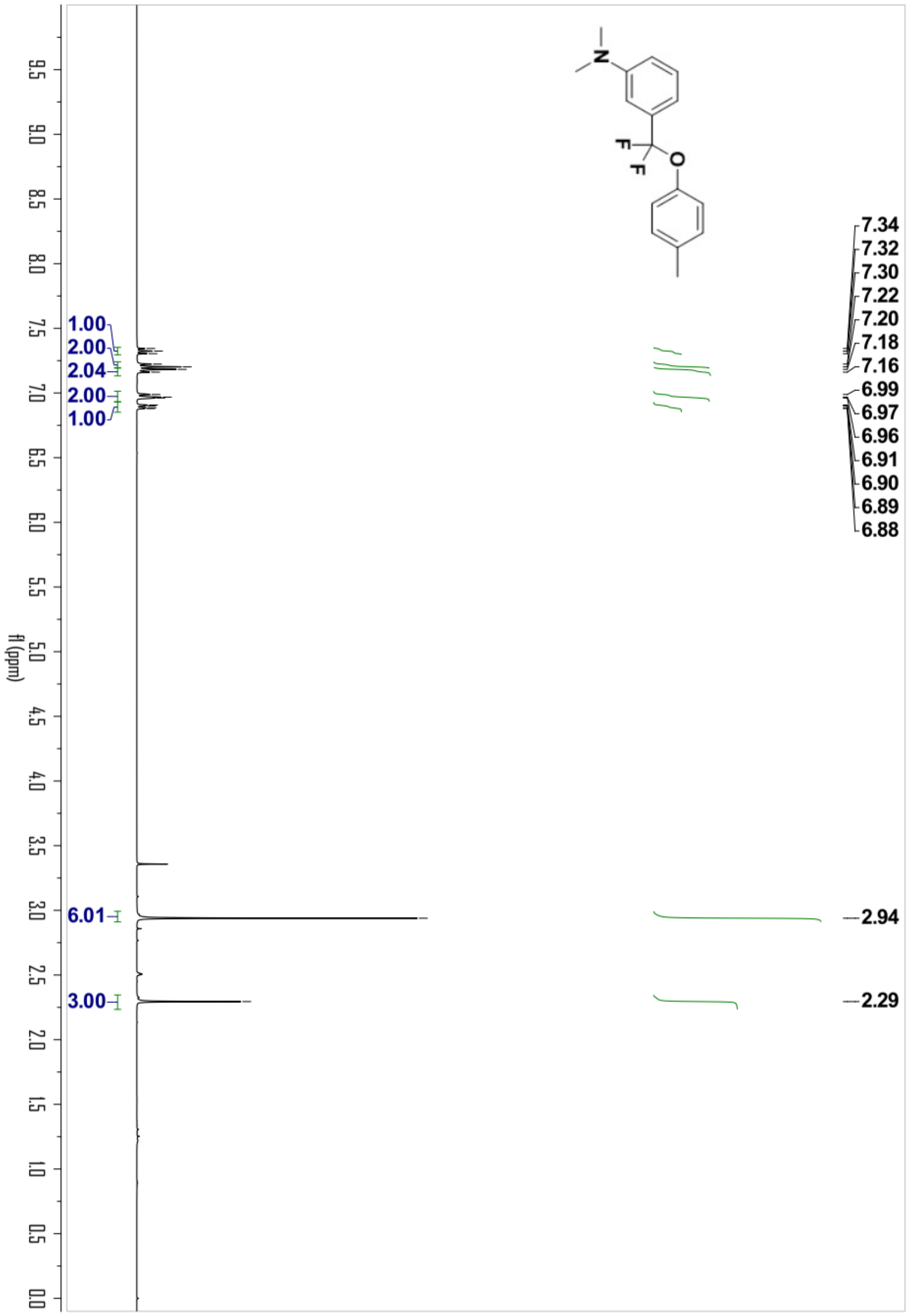

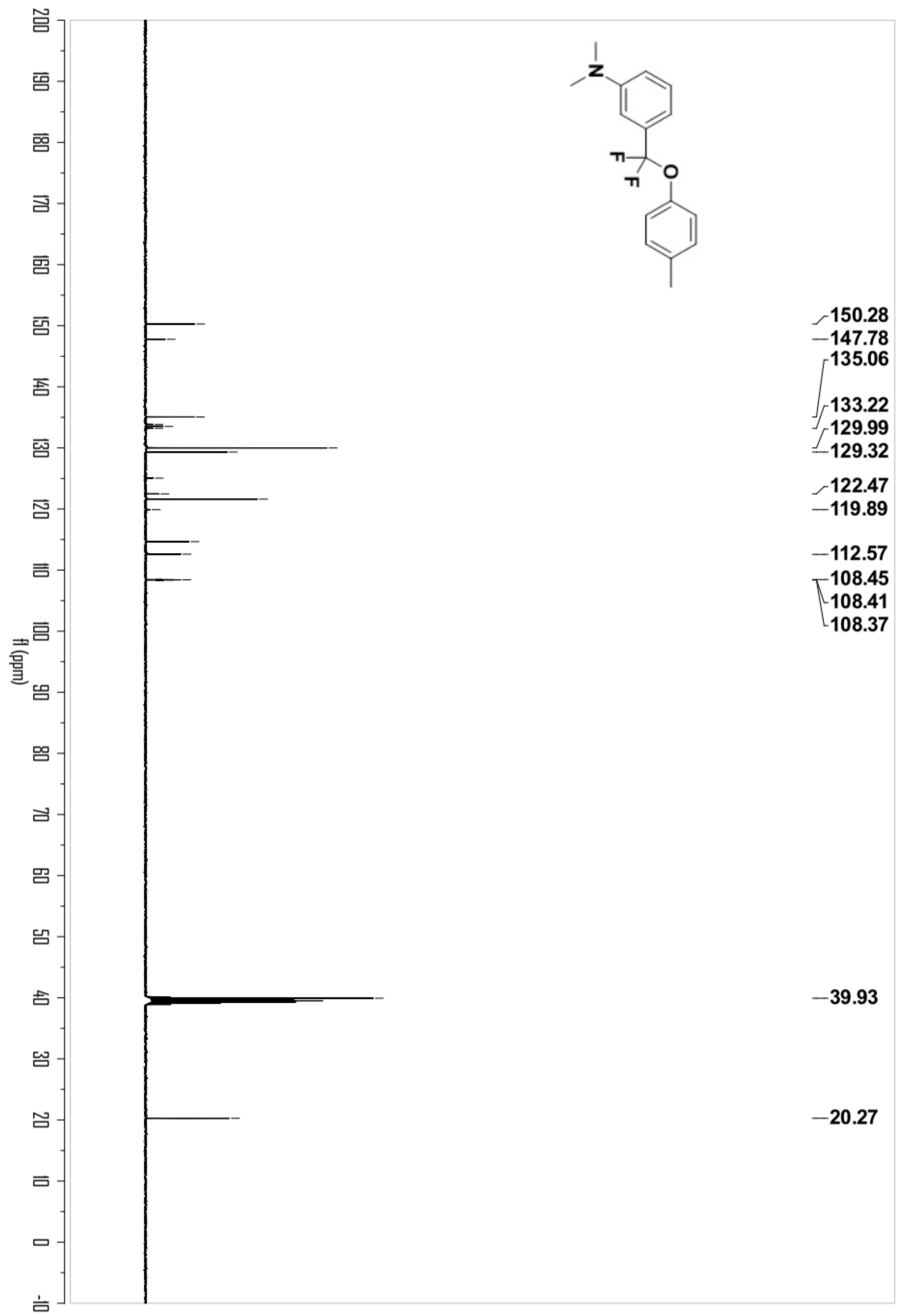
150.28
147.78
135.06
133.22
129.99
129.32
122.47
119.89
112.57
108.45
108.41
108.37
39.93
20.27
f1 (ppm)
N
F
F
O

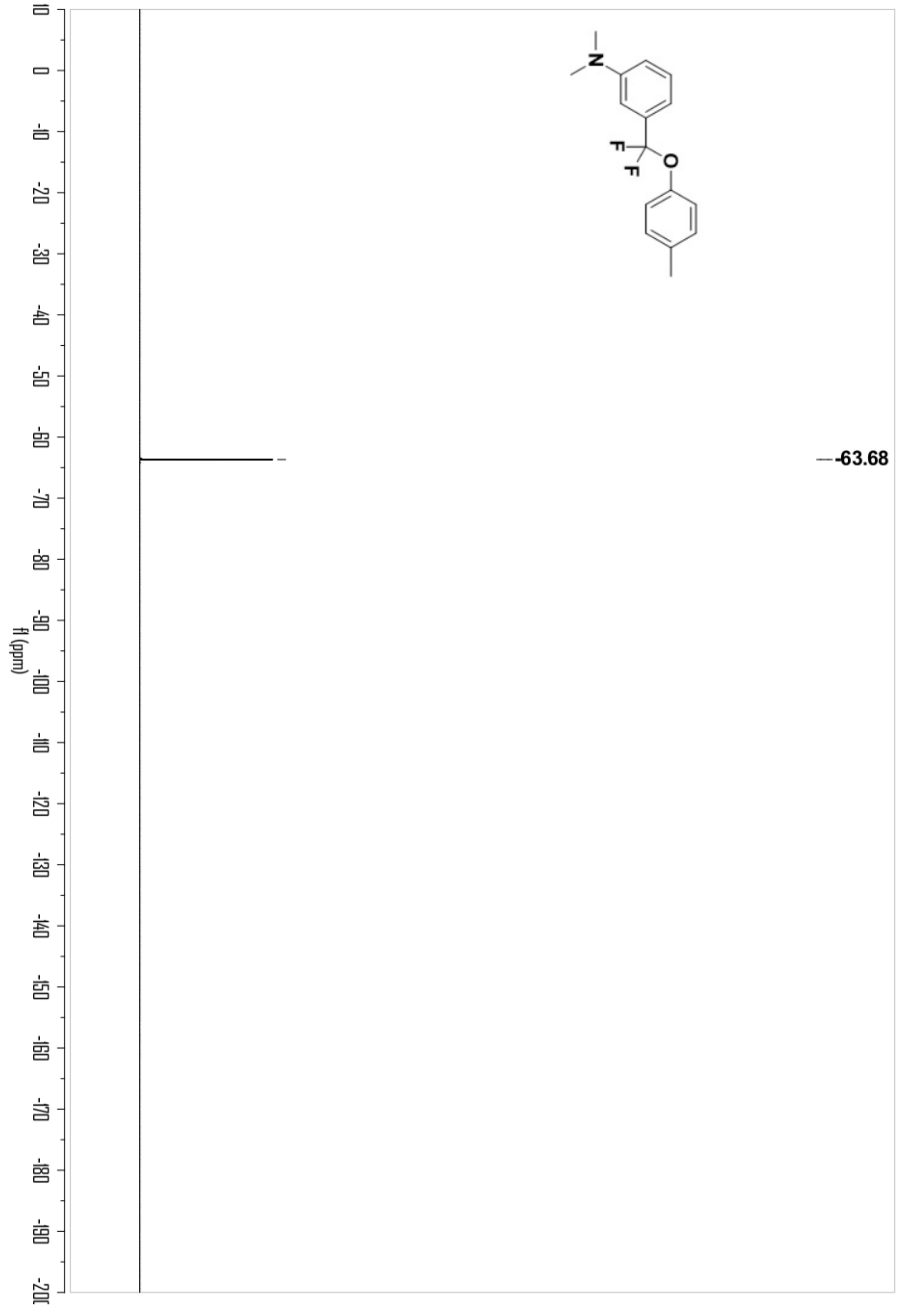
-63.68
f1 (ppm)

^{1}H, ^{13}C and ^{19}F NMR spectra of compound 3r

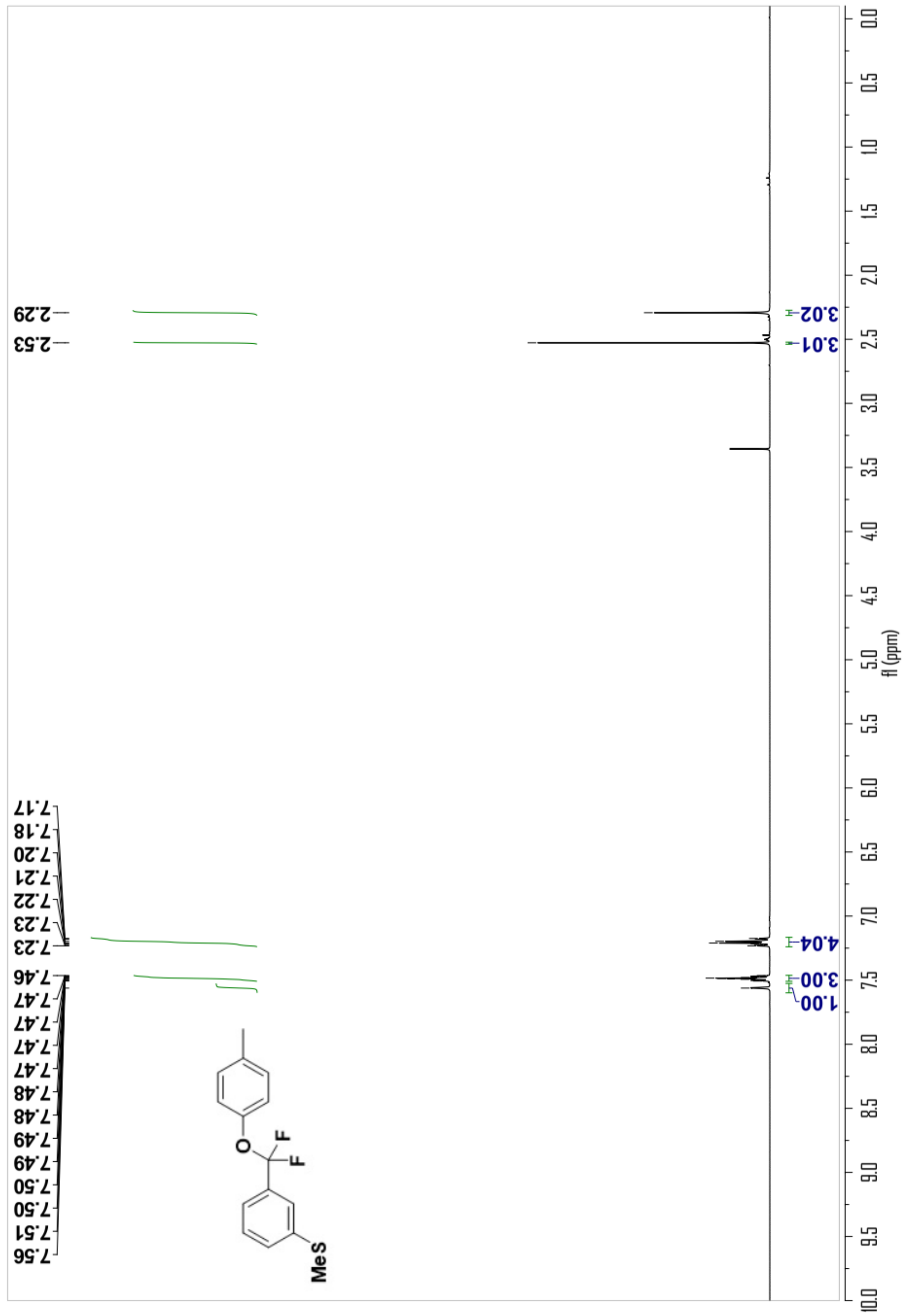

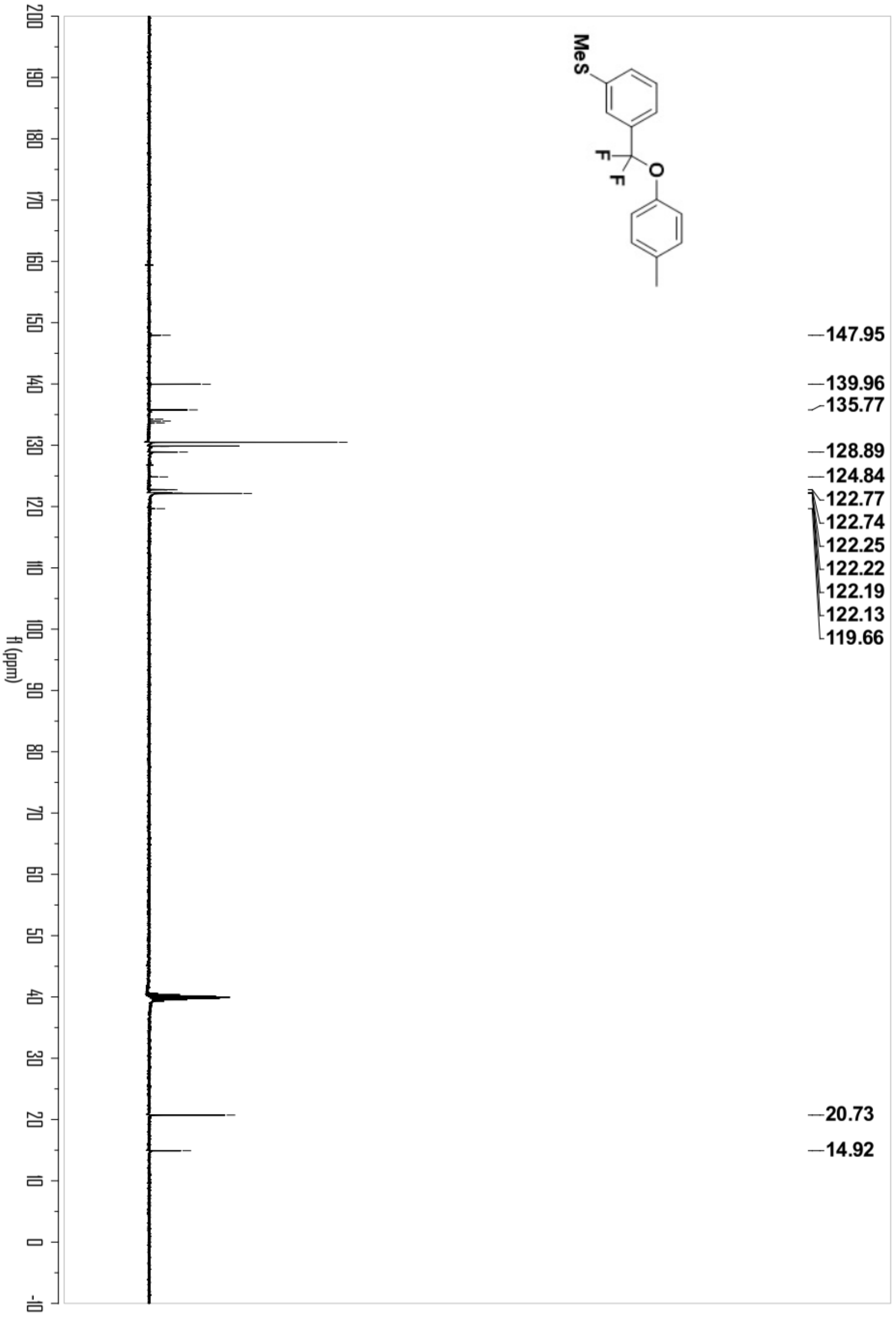
MeS
F
F
O
147.95
139.96
135.77
128.89
124.84
122.77
122.74
122.25
122.22
122.19
122.13
119.66
20.73
14.92
f1 (ppm)

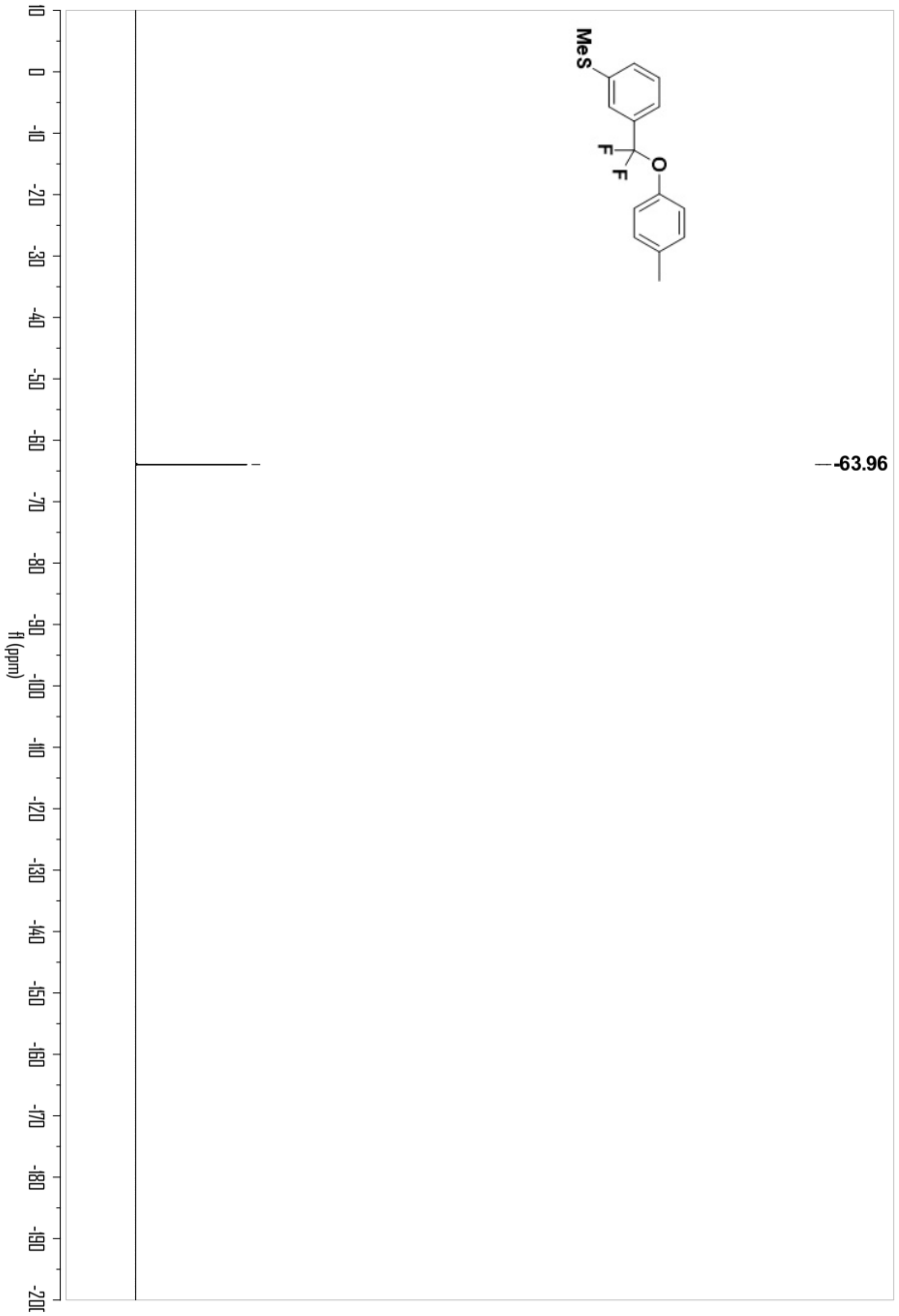
MeS
F
F
O
-63.96
f1 (ppm)

^{1}H, ^{13}C and ^{19}F NMR spectra of compound 3s

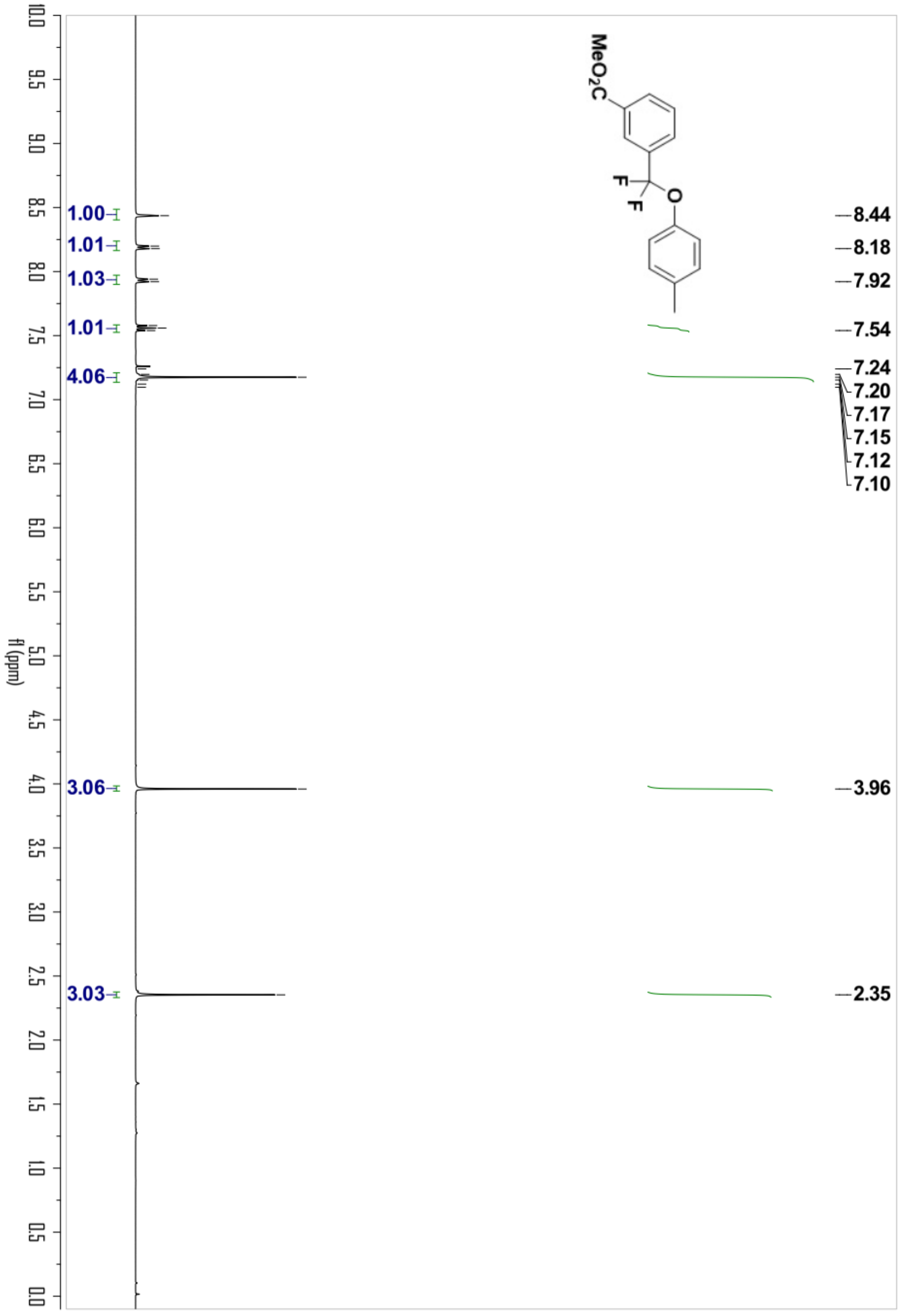

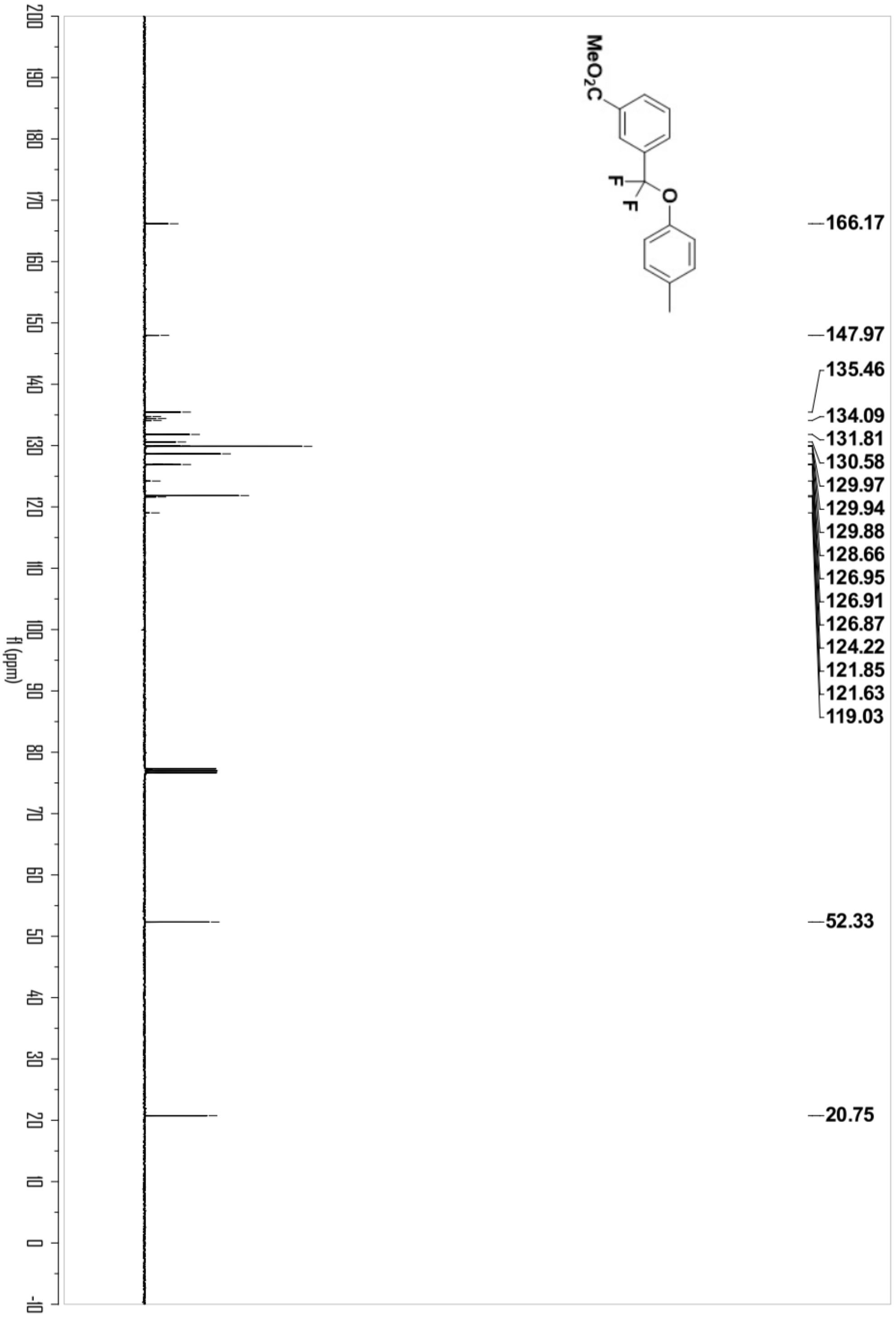
MeO2C
F
F
O
166.17
147.97
135.46
134.09
131.81
130.58
129.97
129.94
129.88
128.66
126.95
126.91
126.87
124.22
121.85
121.63
119.03
52.33
20.75
f1 (ppm)

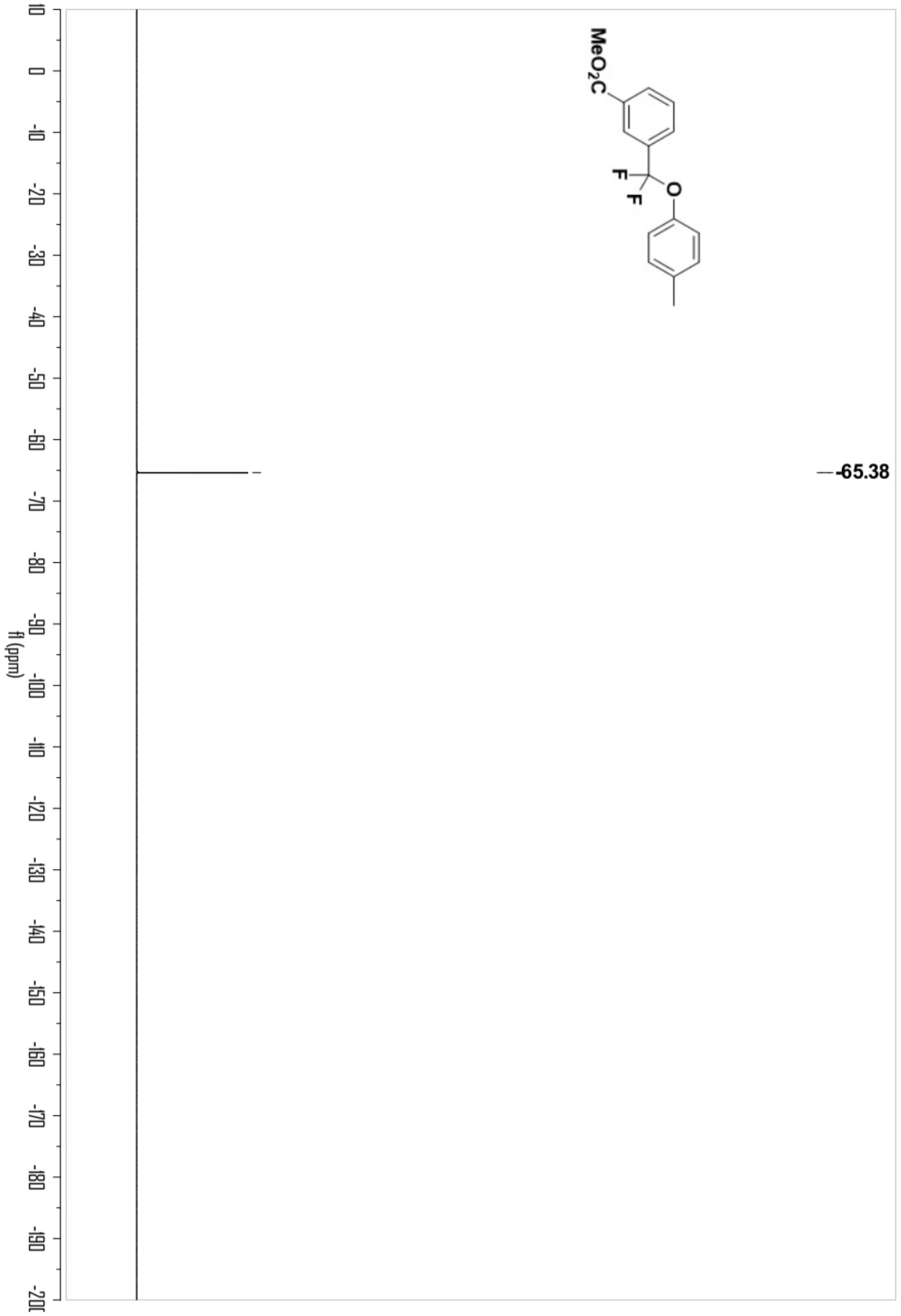
-65.38

^{1}H, ^{13}C and ^{19}F NMR spectra of compound 3t

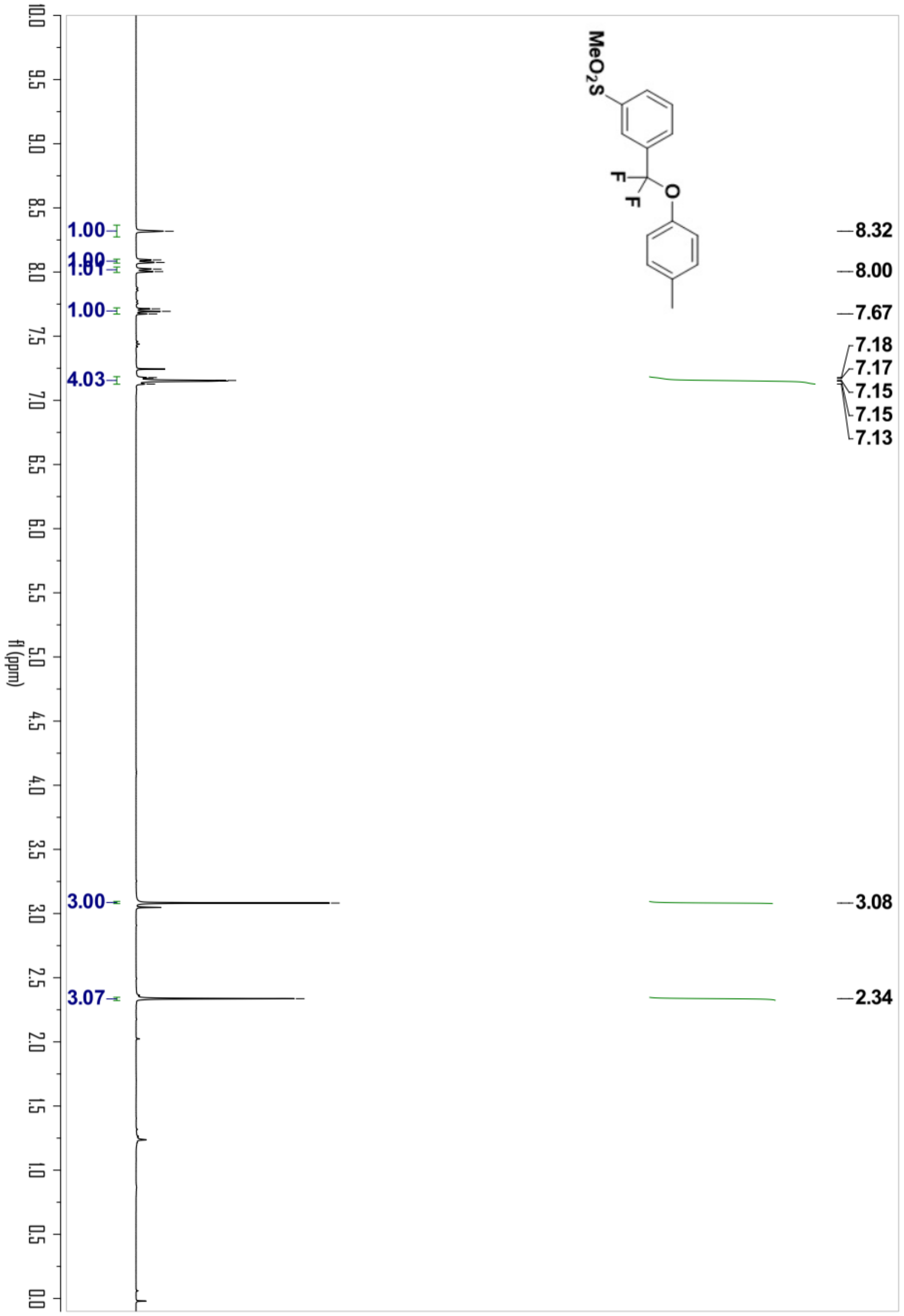

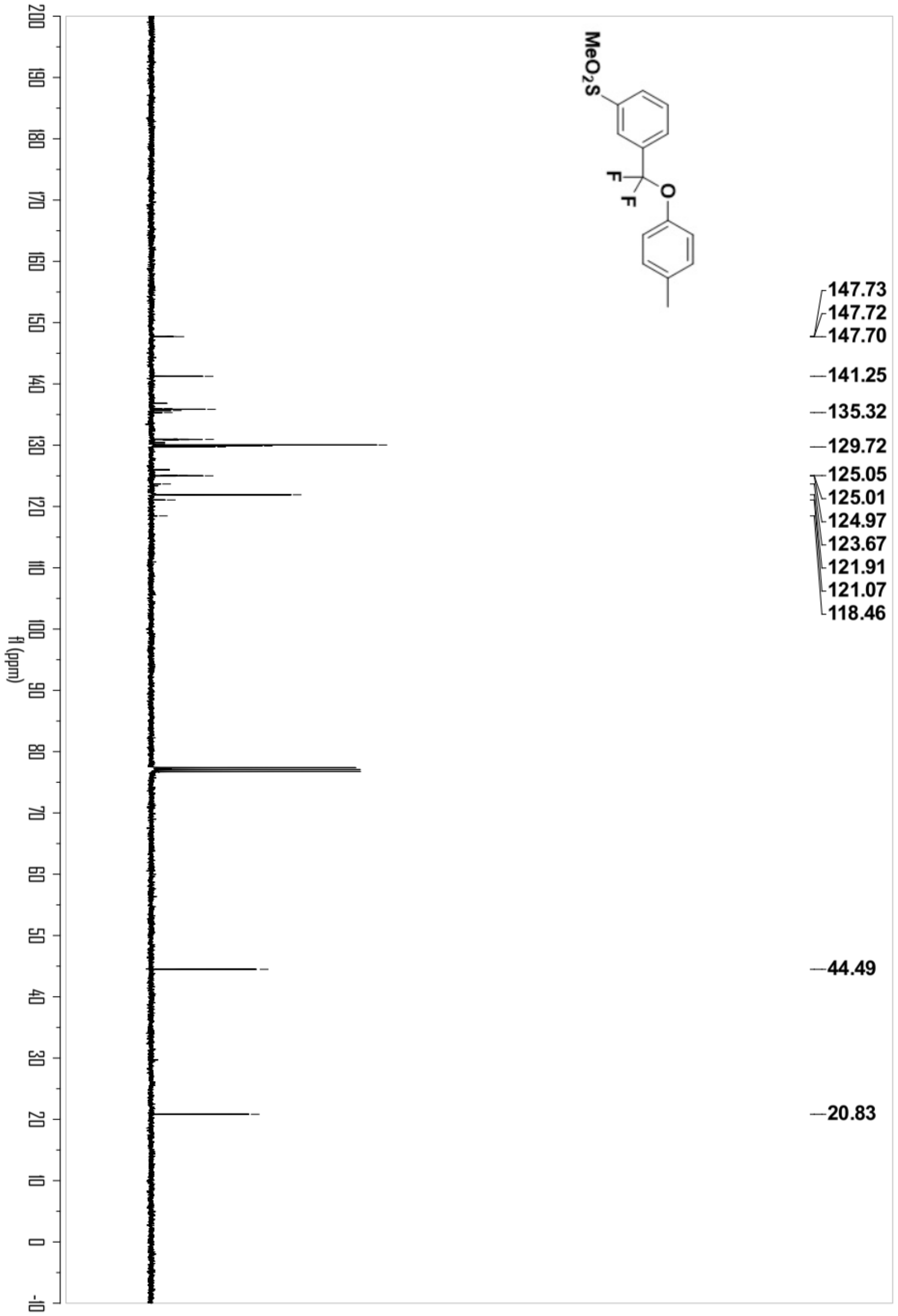
MeO2S
F
F
O
147.73
147.72
147.70
141.25
135.32
129.72
125.05
125.01
124.97
123.67
121.91
121.07
118.46
44.49
20.83
f1 (ppm)

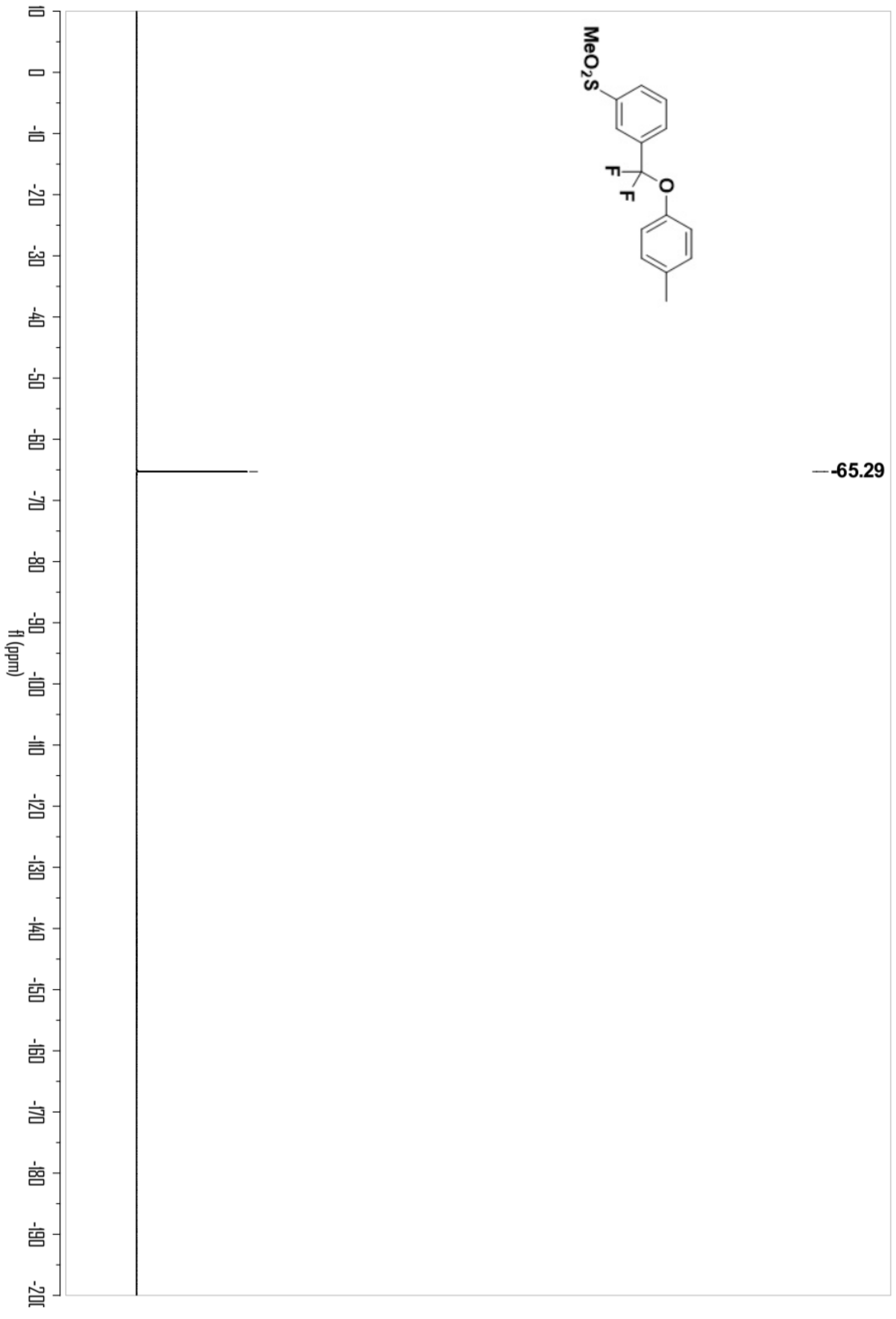
MeO2S
F
F
O
-65.29
f1 (ppm)
10
0
-10
-20
-30
-40
-50
-60
-70
-80
-90
-100
-110
-120
-130
-140
-150
-160
-170
-180
-190
-200

^{1}H, ^{13}C and ^{19}F NMR spectra of compound 3u

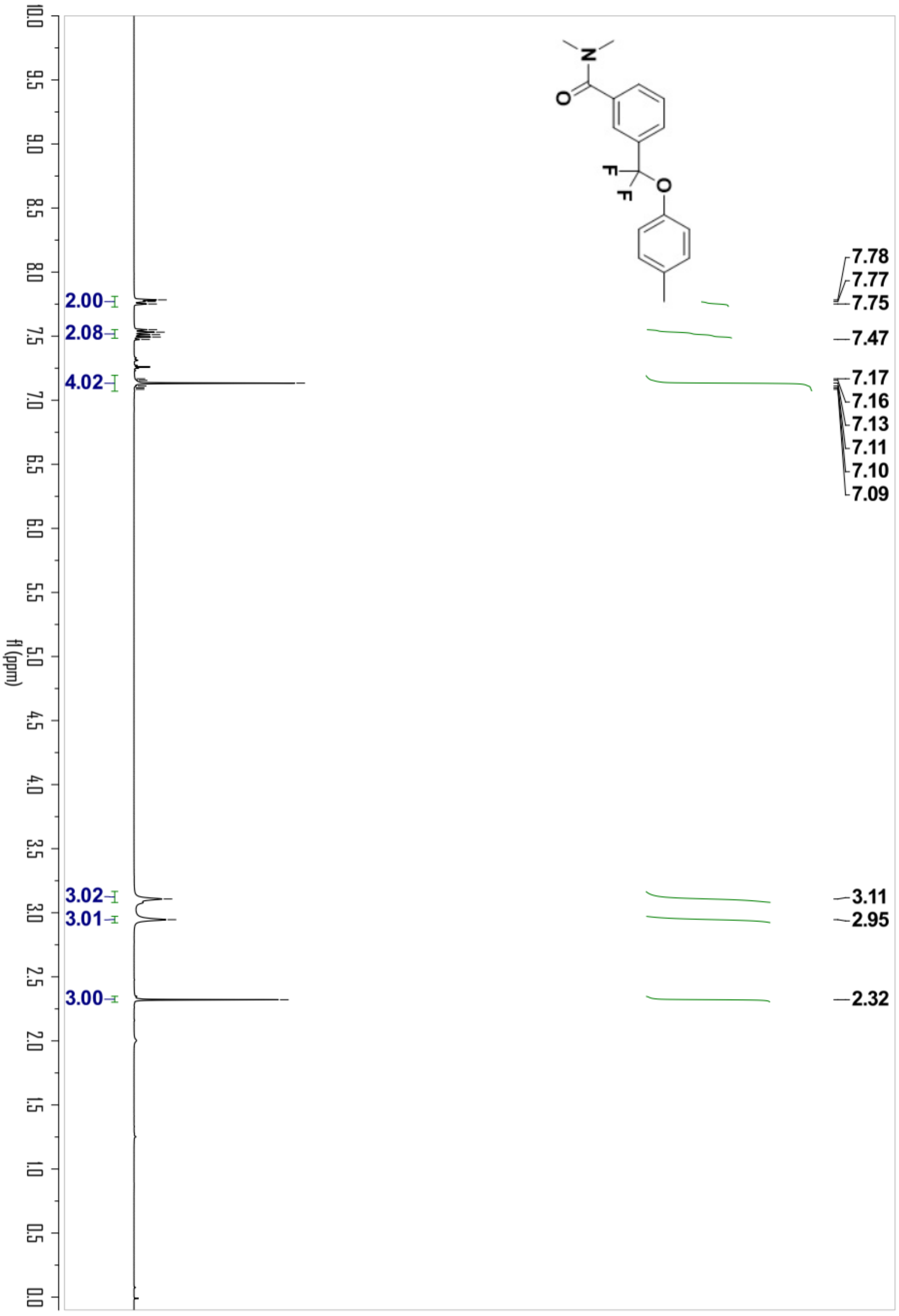

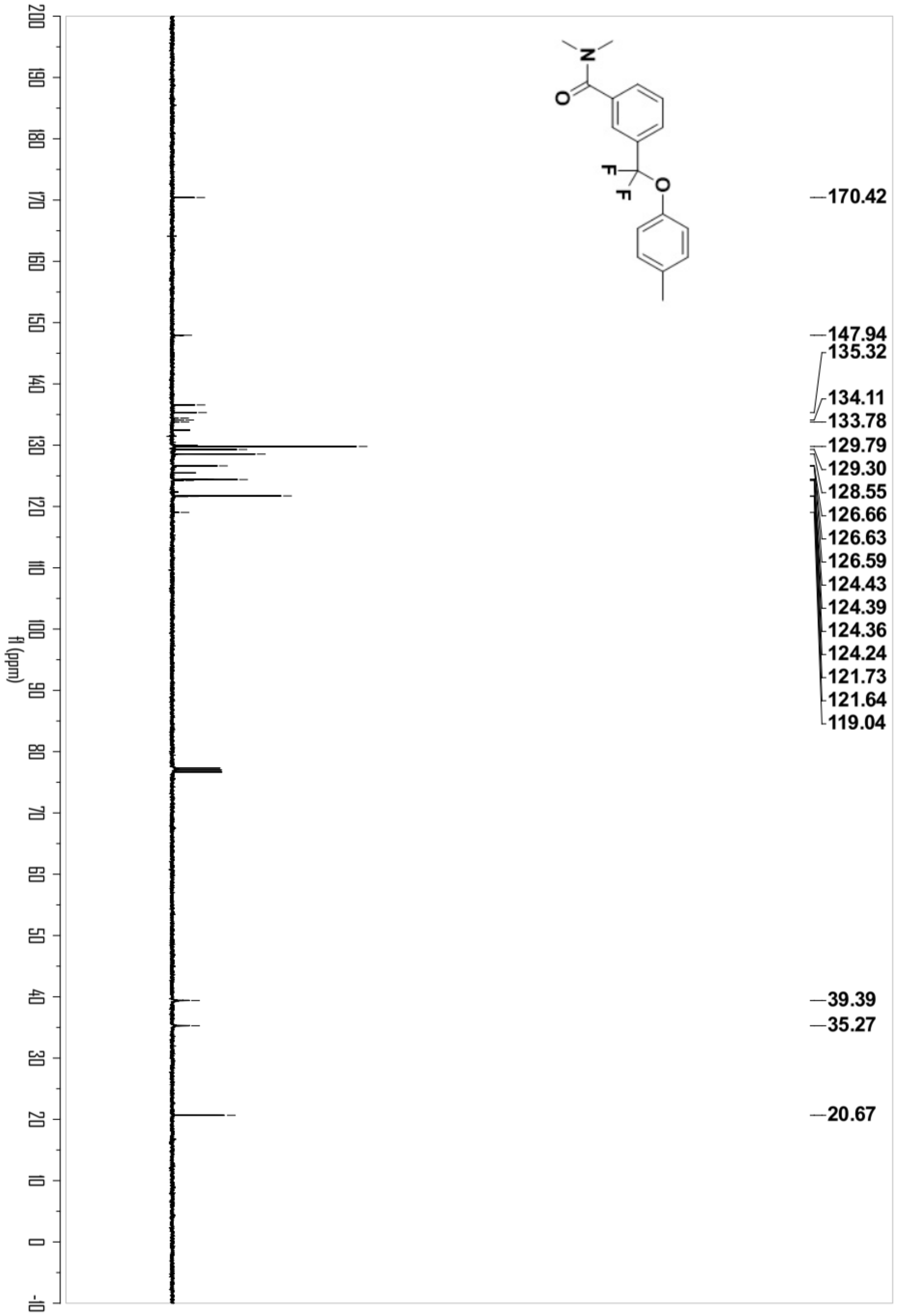
170.42
147.94
135.32
134.11
133.78
129.79
129.30
128.55
126.66
126.63
126.59
124.43
124.39
124.36
124.24
121.73
121.64
119.04
39.39
35.27
20.67
f1 (ppm)

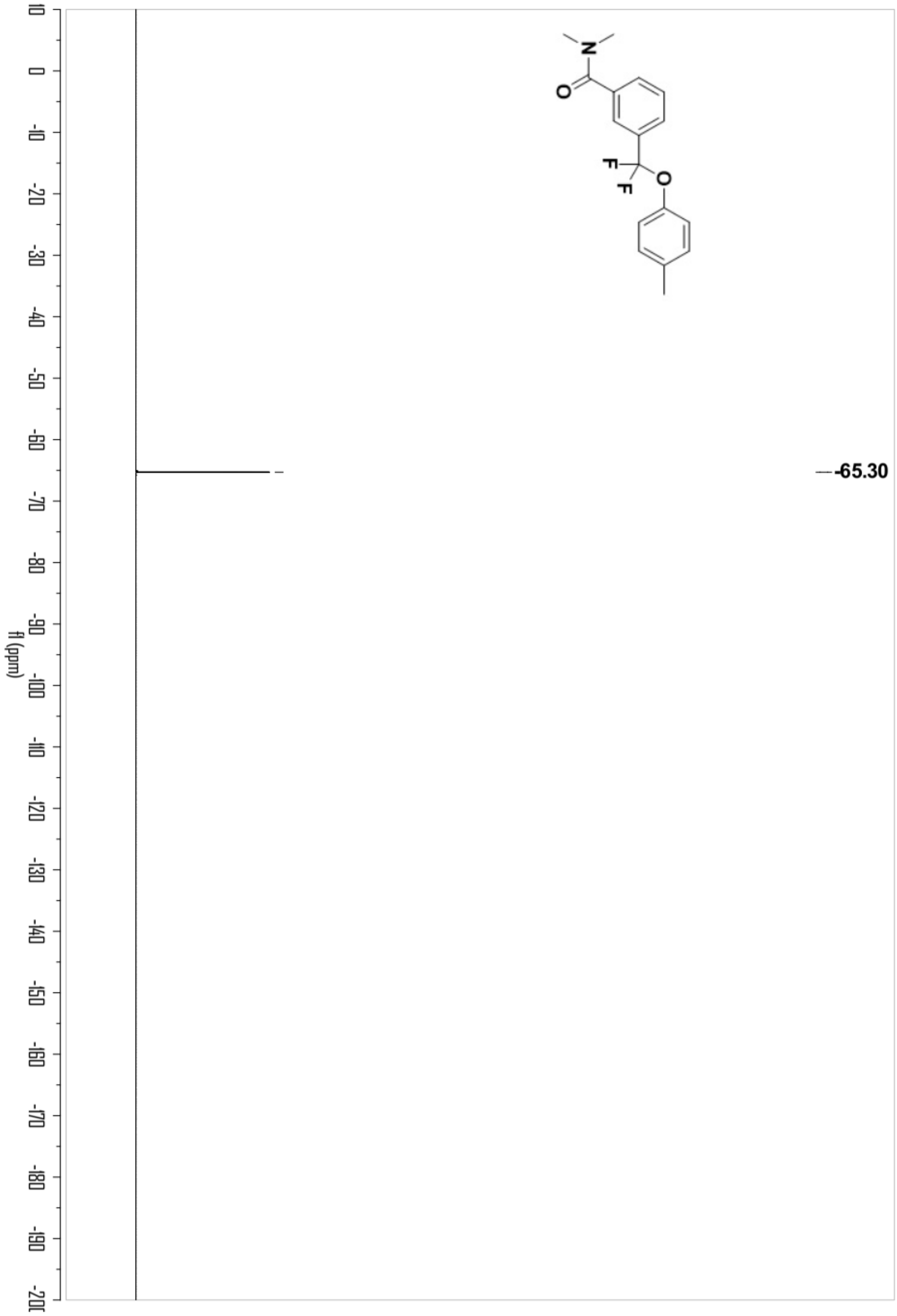
-65.30
f1 (ppm)

^{1}H, ^{13}C and ^{19}F NMR spectra of compound 3v

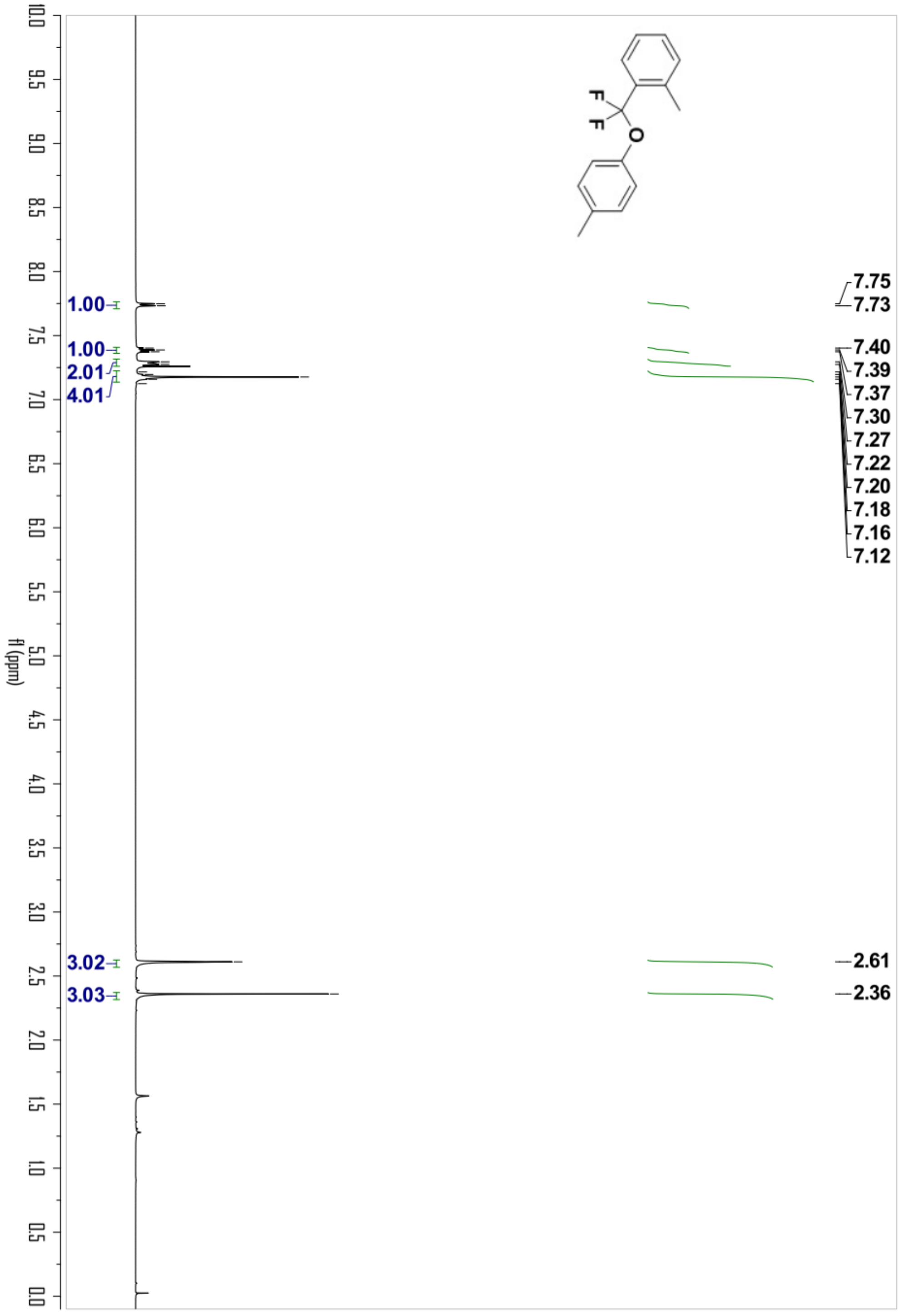

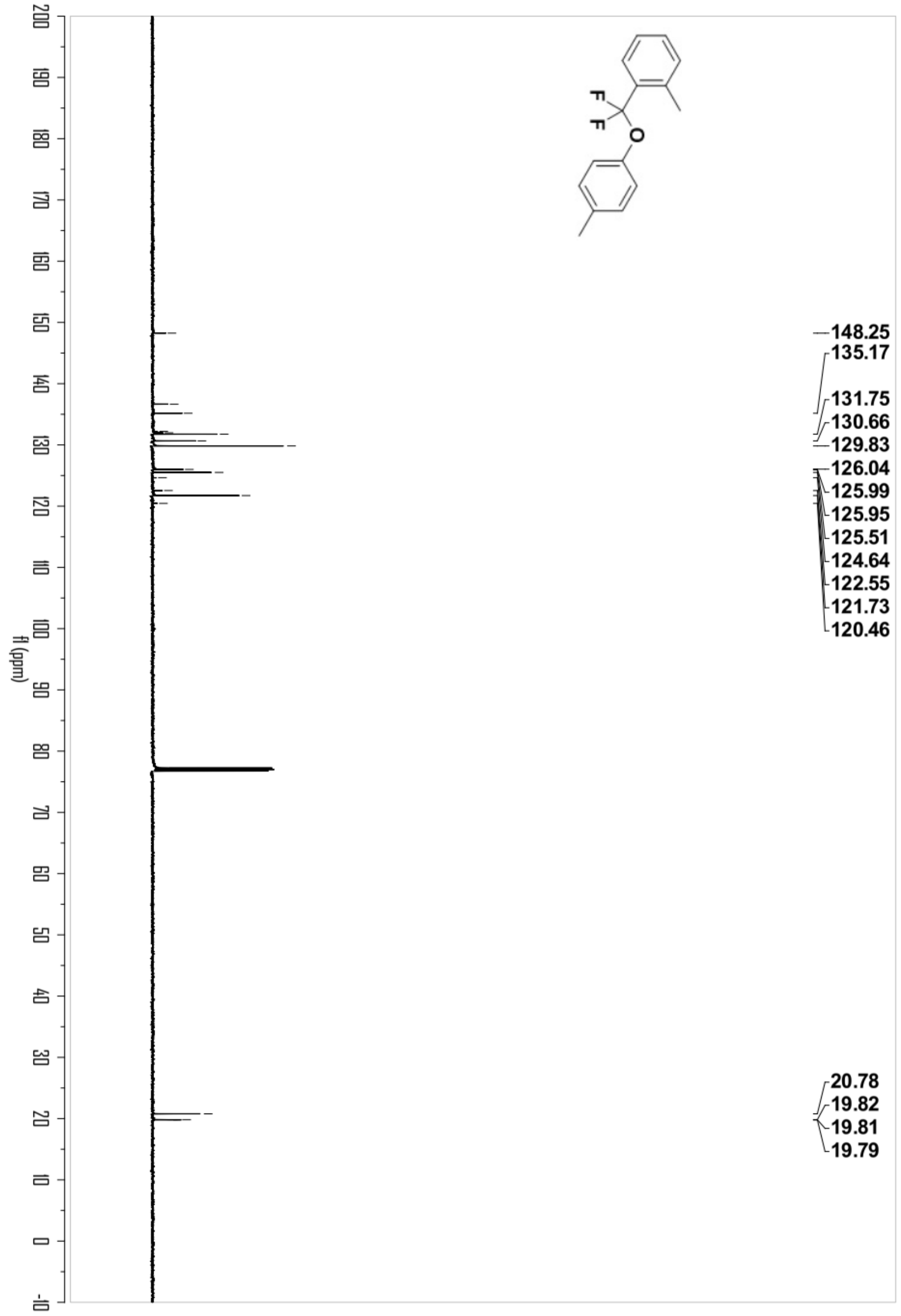
148.25
135.17
131.75
130.66
129.83
126.04
125.99
125.95
125.51
124.64
122.55
121.73
120.46
20.78
19.82
19.81
19.79
f1 (ppm)

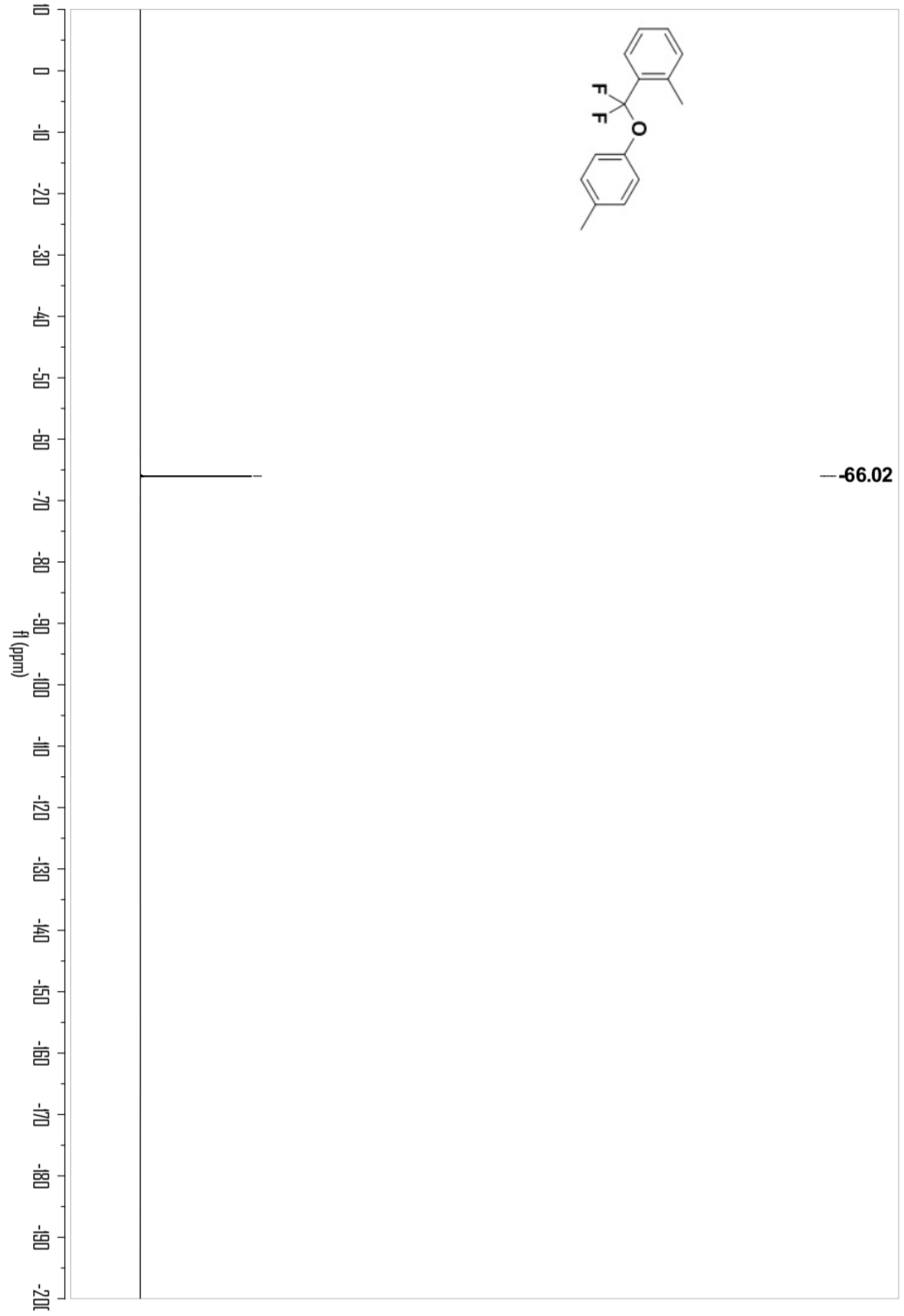
-66.02
f1 (ppm)

^{1}H, ^{13}C and ^{19}F NMR spectra of compound 3w

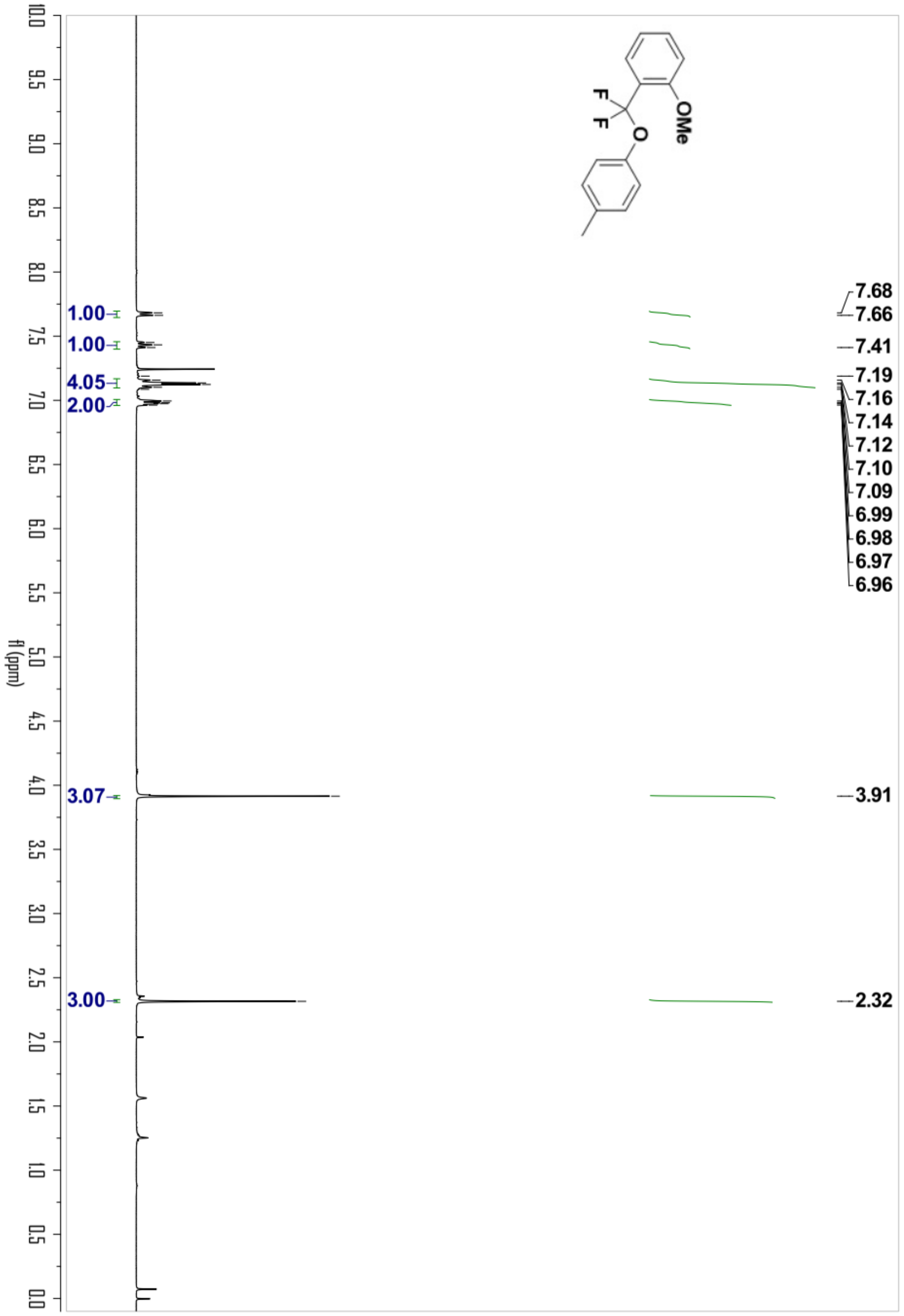

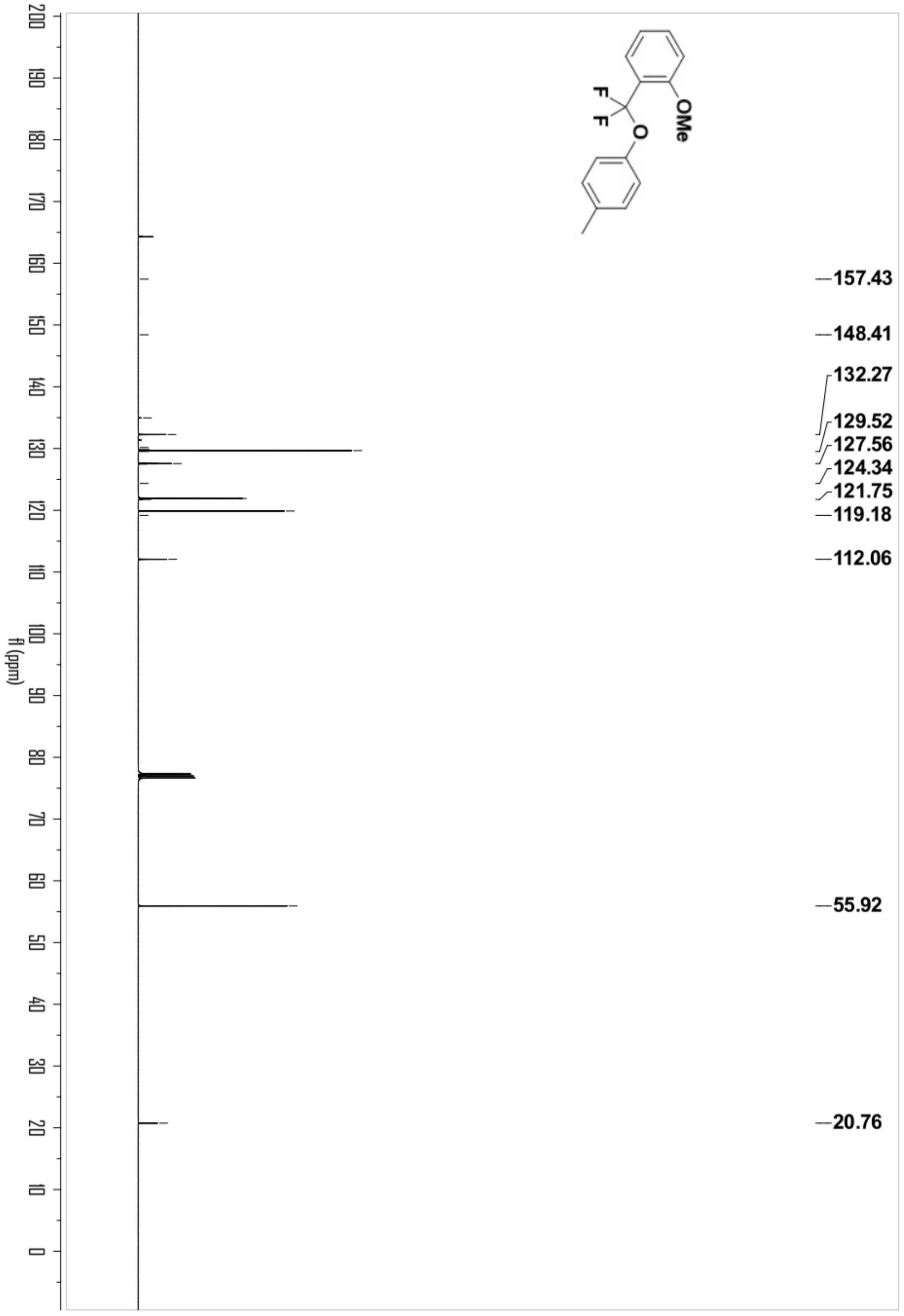
OMe
F
F
O
157.43
148.41
132.27
129.52
127.56
124.34
121.75
119.18
112.06
55.92
20.76
f1 (ppm)

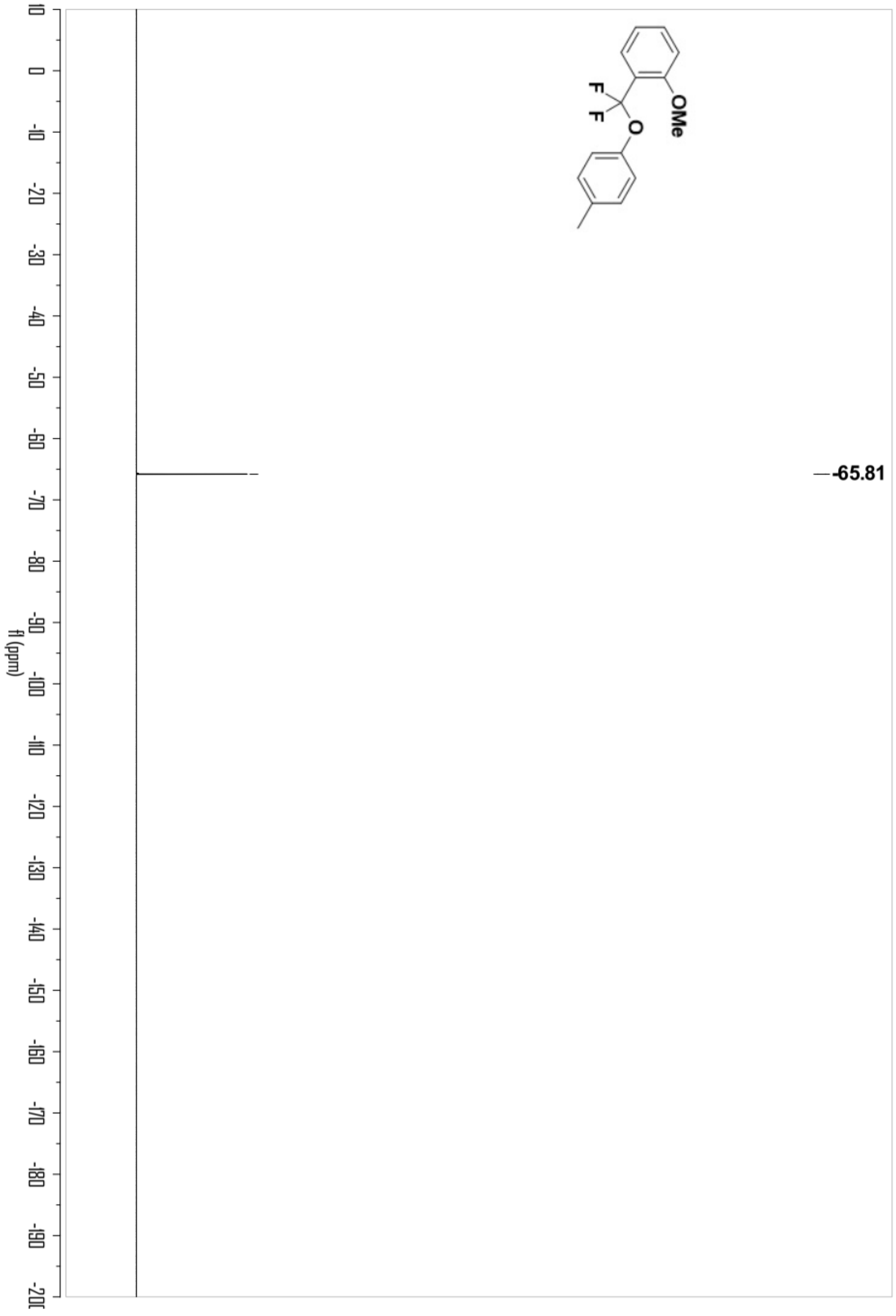
OMe
F
F
O
-65.81
f1 (ppm)

^1H, ^{13}C and ^{19}F NMR spectra of compound 3x

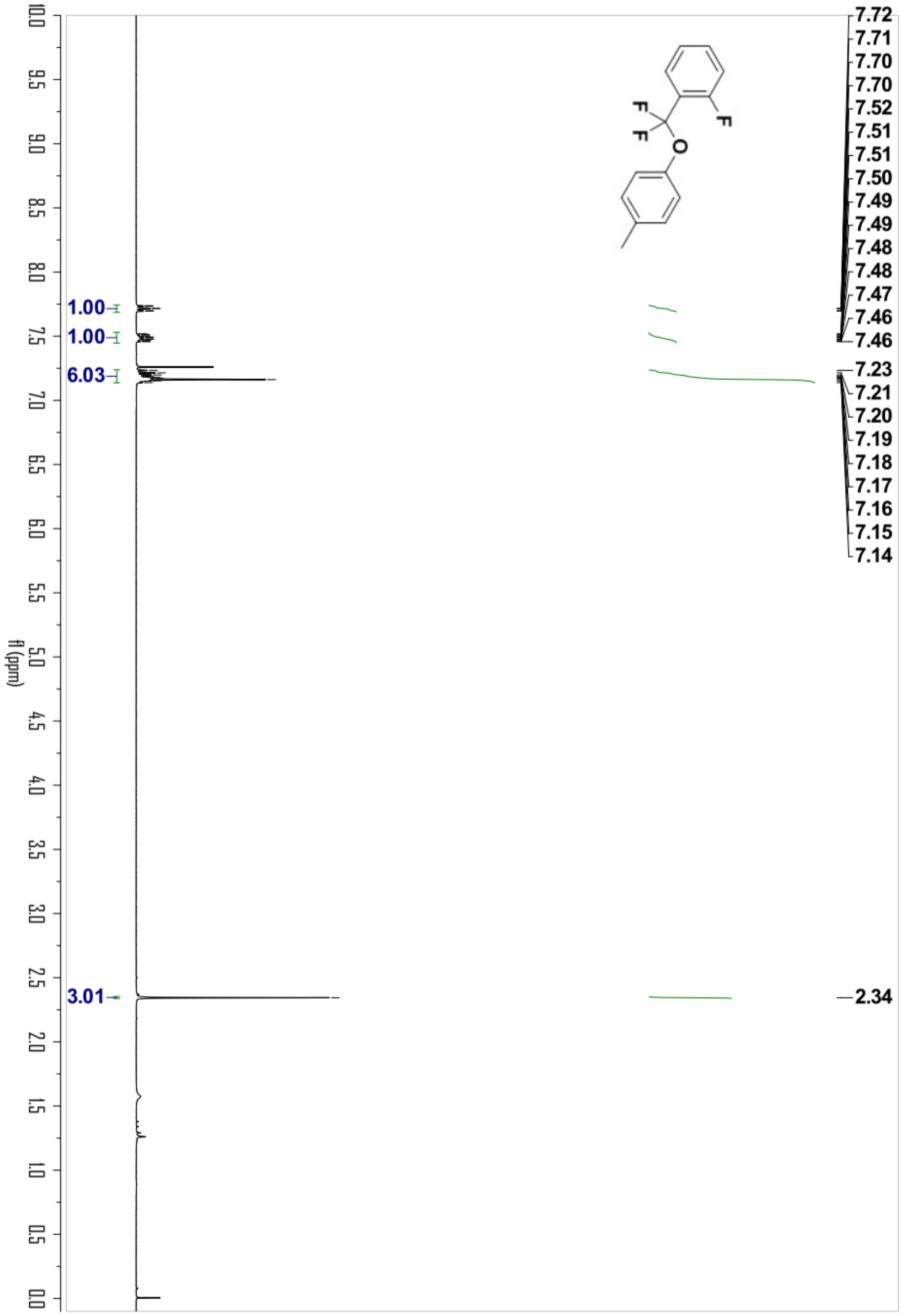

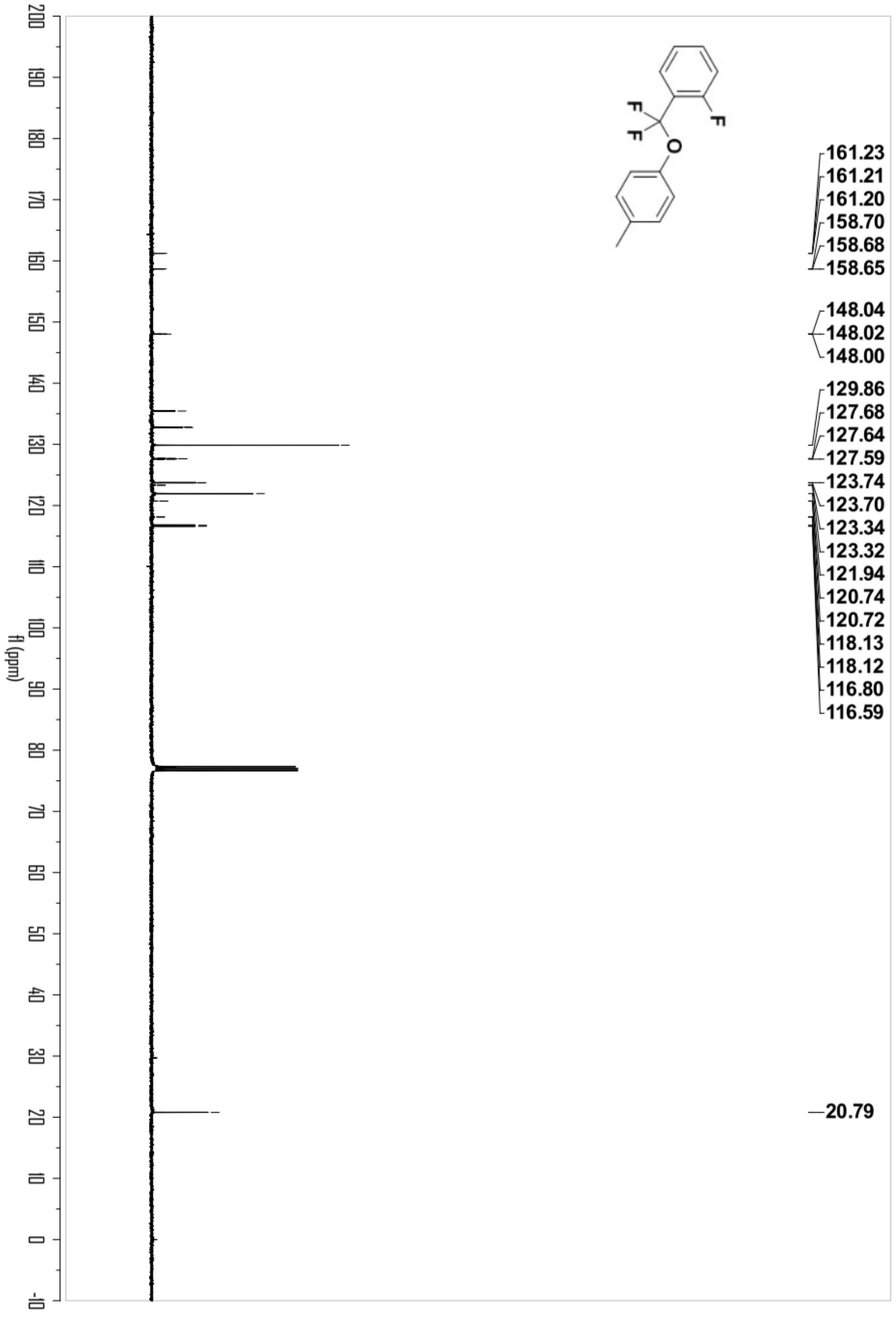
F
F
F
O
161.23
161.21
161.20
158.70
158.68
158.65
148.04
148.02
148.00
129.86
127.68
127.64
127.59
123.74
123.70
123.34
123.32
121.94
120.74
120.72
118.13
118.12
116.80
116.59
20.79
f1 (ppm)

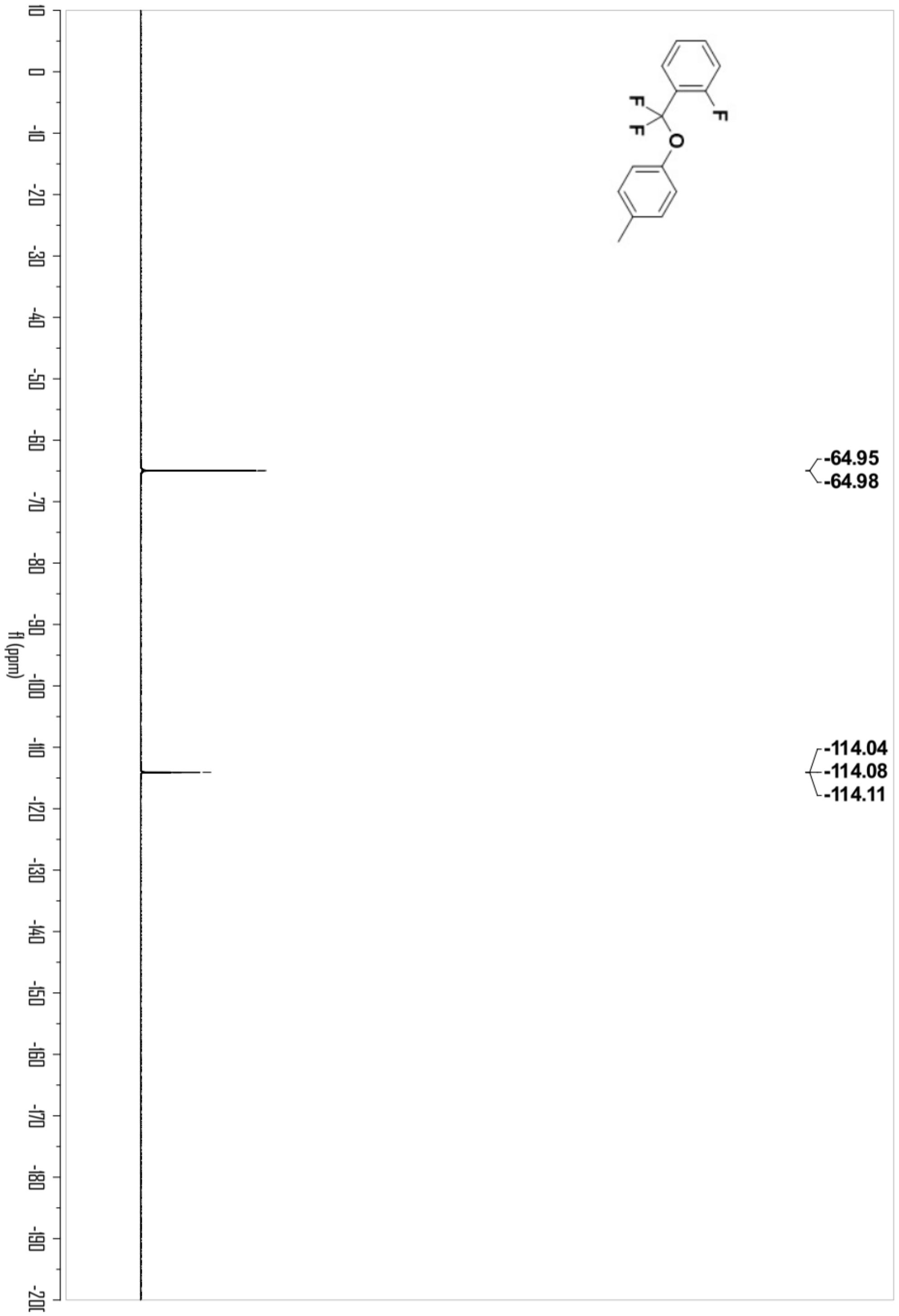
-64.95
-64.98
-114.04
-114.08
-114.11
f1 (ppm)

^{1}H, ^{13}C and ^{19}F NMR spectra of compound 3y

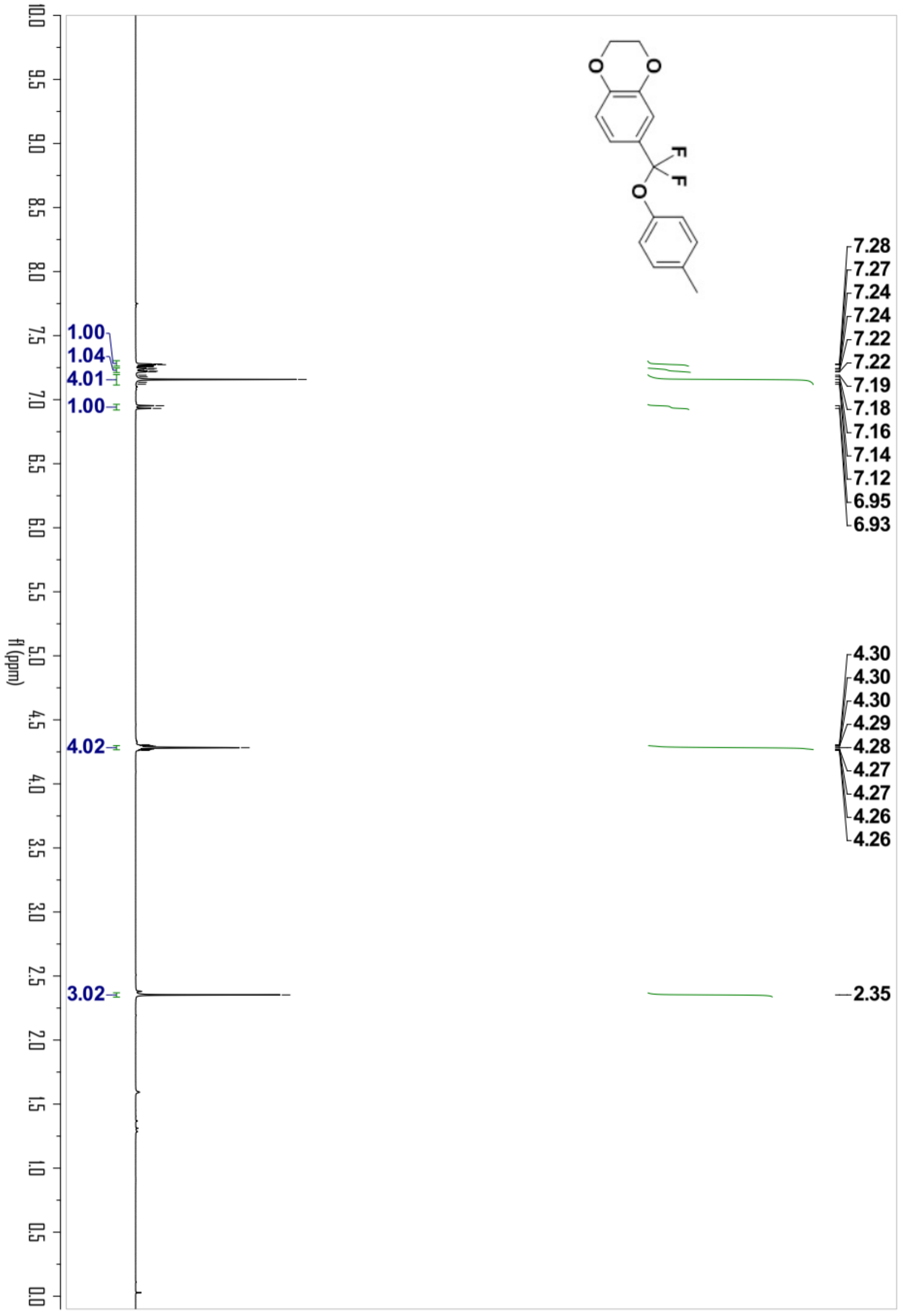

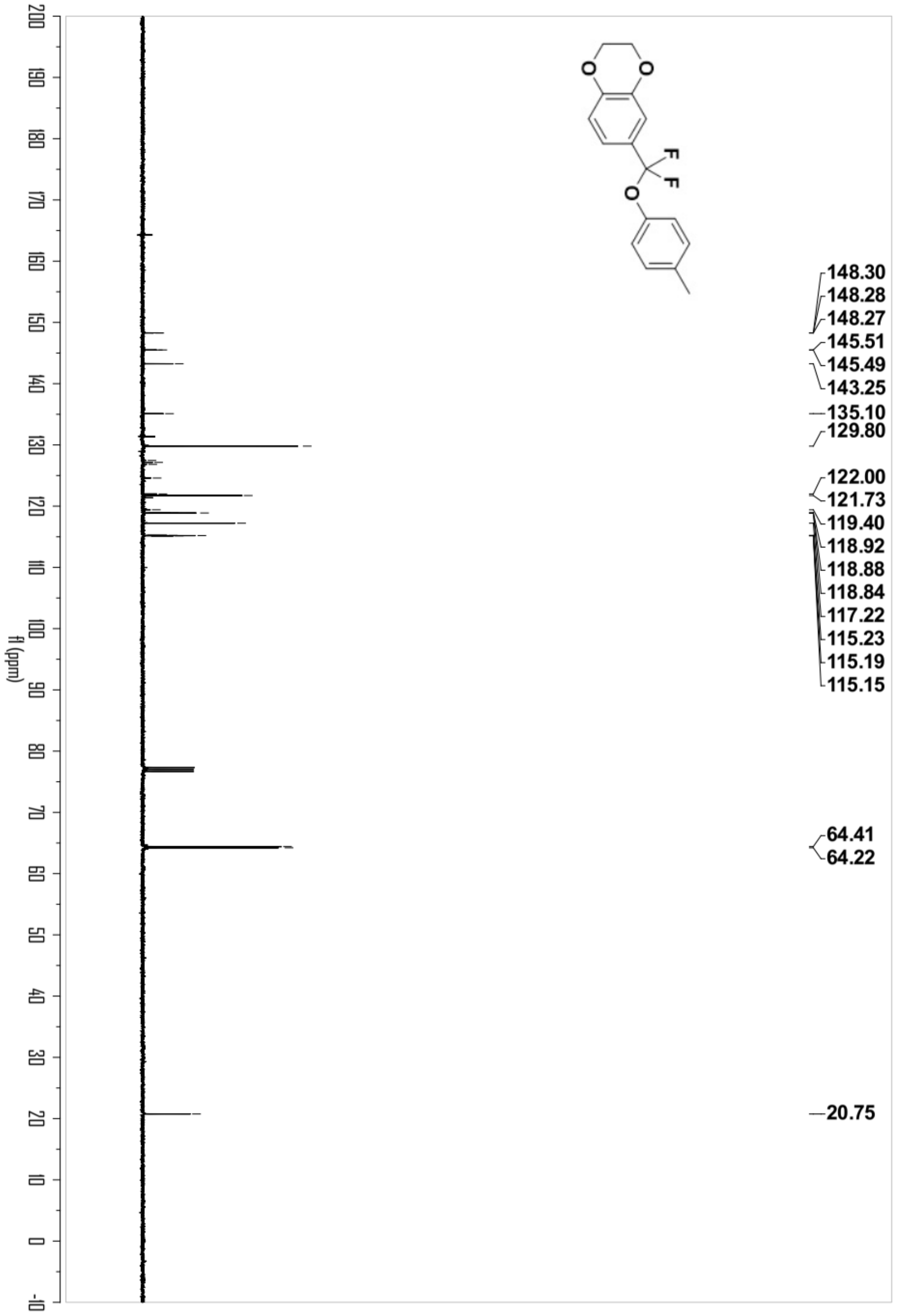
148.30
148.28
148.27
145.51
145.49
143.25
135.10
129.80
122.00
121.73
119.40
118.92
118.88
118.84
117.22
115.23
115.19
115.15
64.41
64.22
20.75
F
F
O
O
O
f1 (ppm)

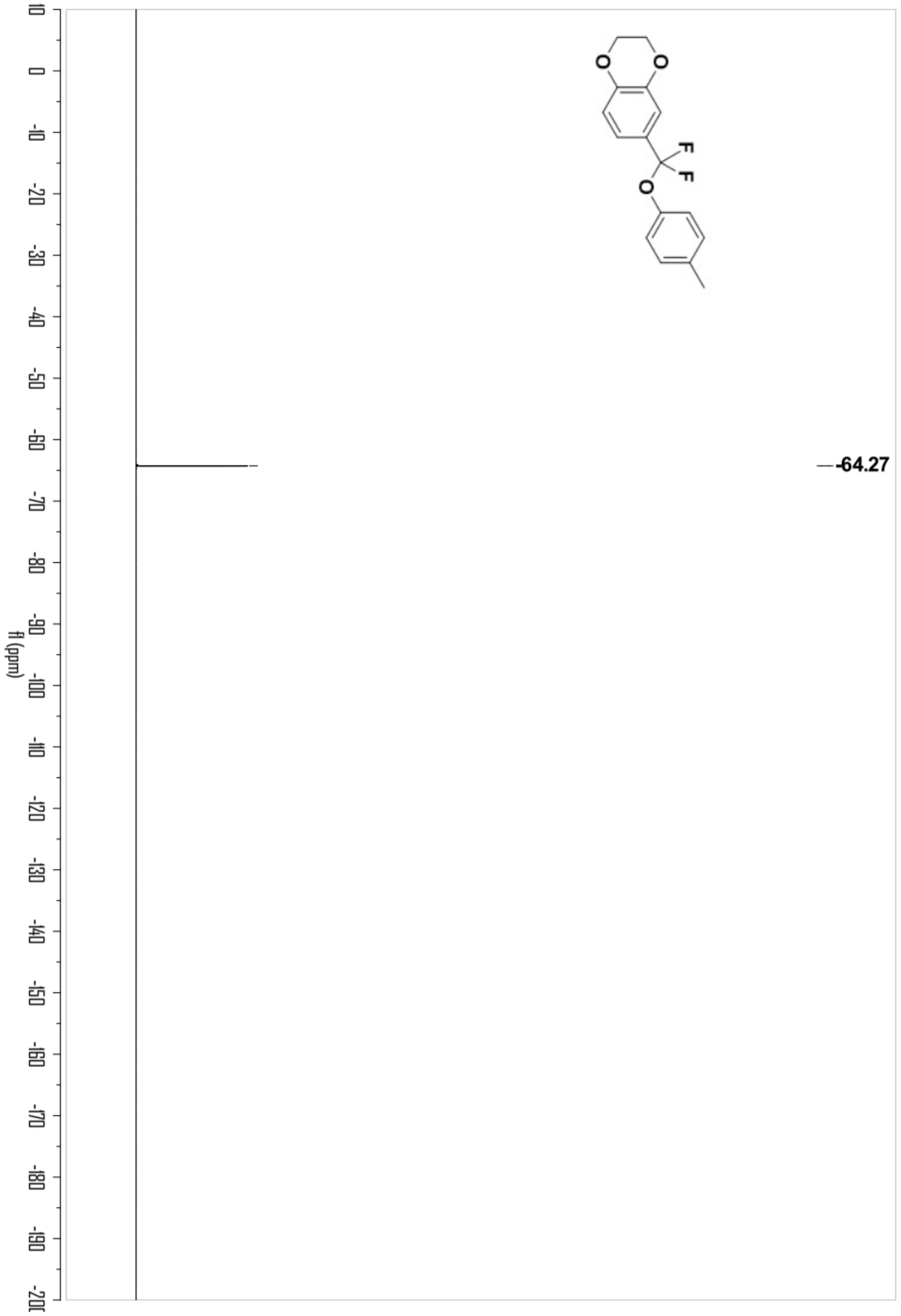
-64.27
f1 (ppm)
F
F
O
O
O

^{1}H, ^{13}C and ^{19}F NMR spectra of compound 3z

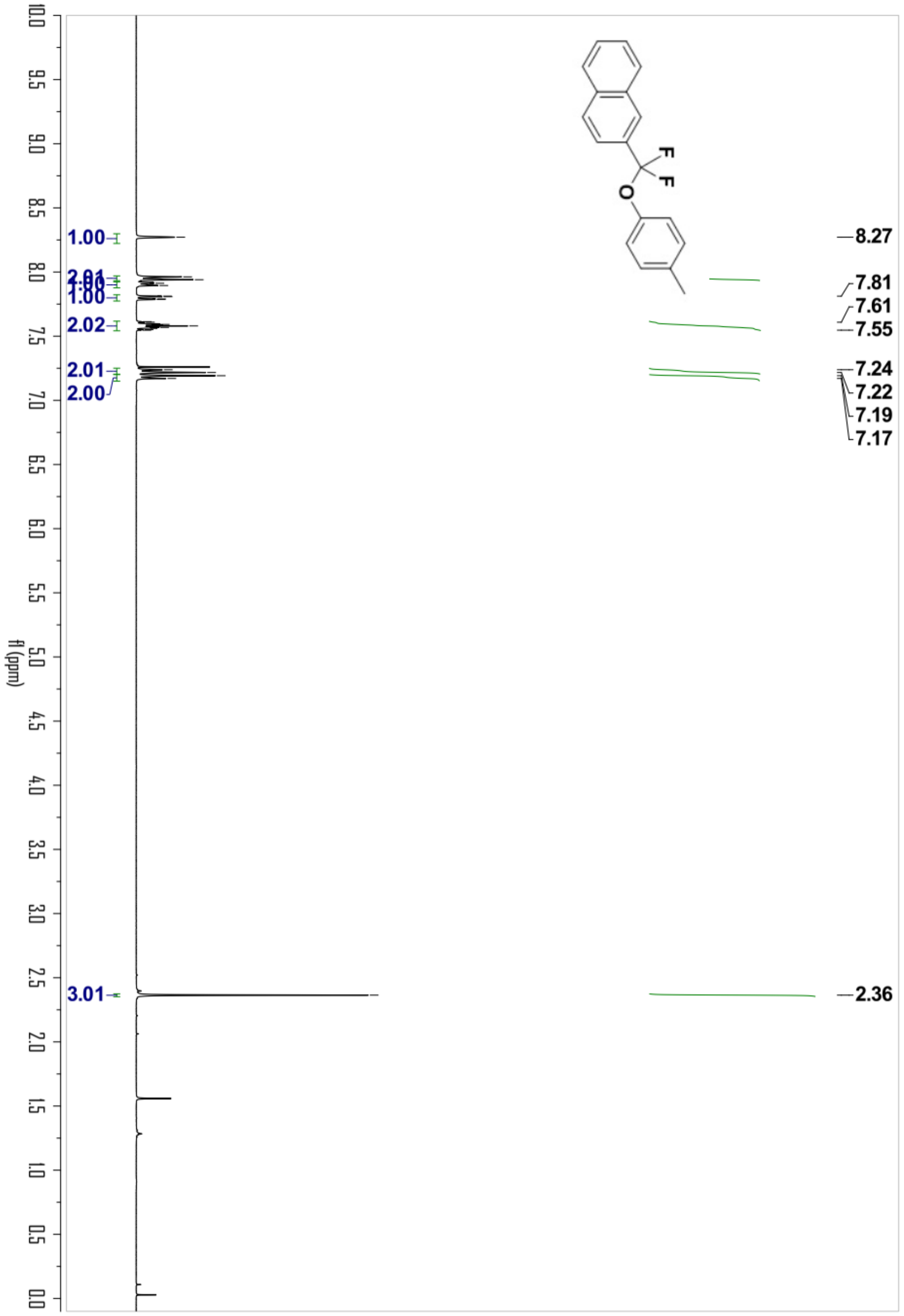

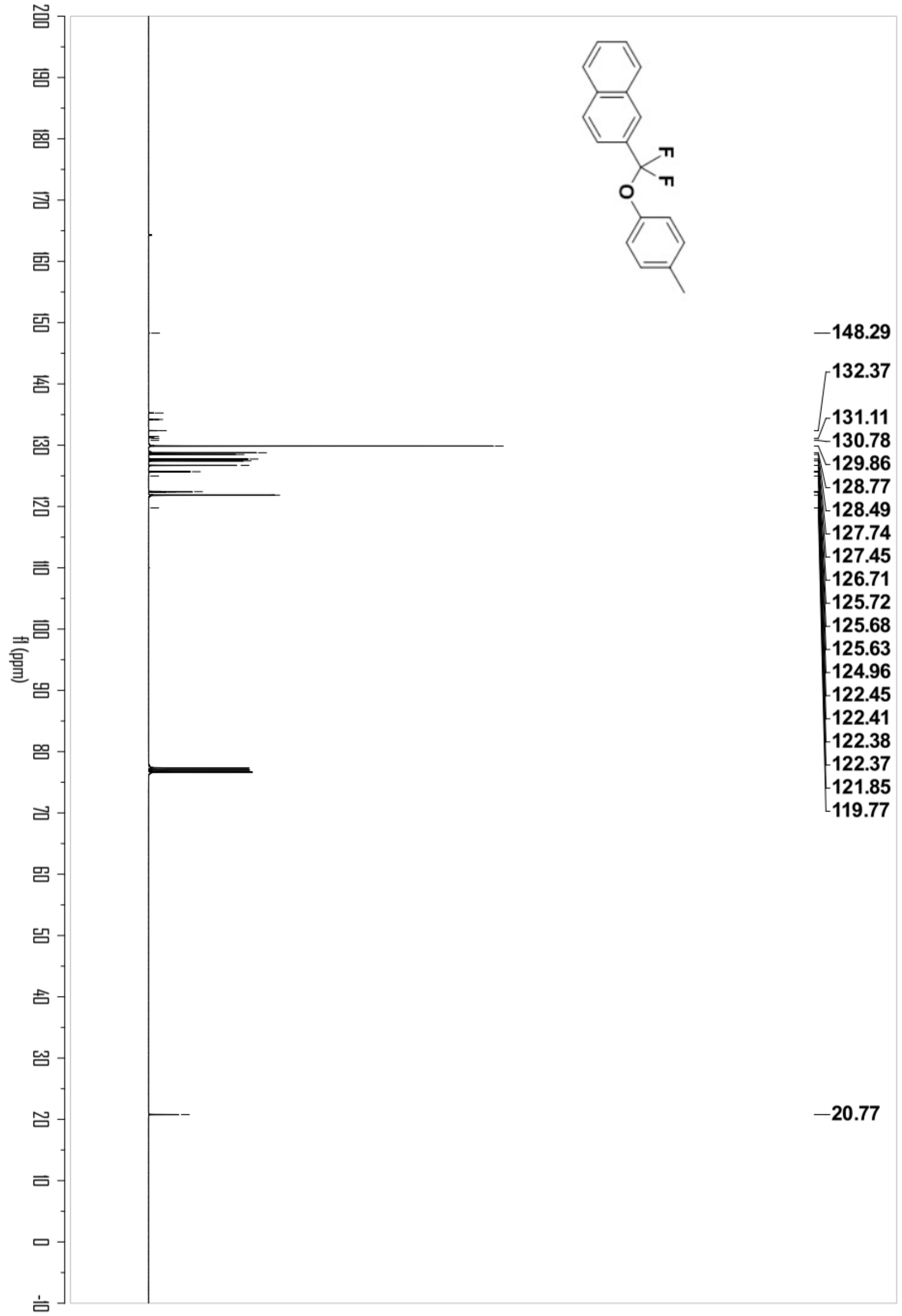
148.29
132.37
131.11
130.78
129.86
128.77
128.49
127.74
127.45
126.71
125.72
125.68
125.63
124.96
122.45
122.41
122.38
122.37
121.85
119.77
20.77
F
F
O

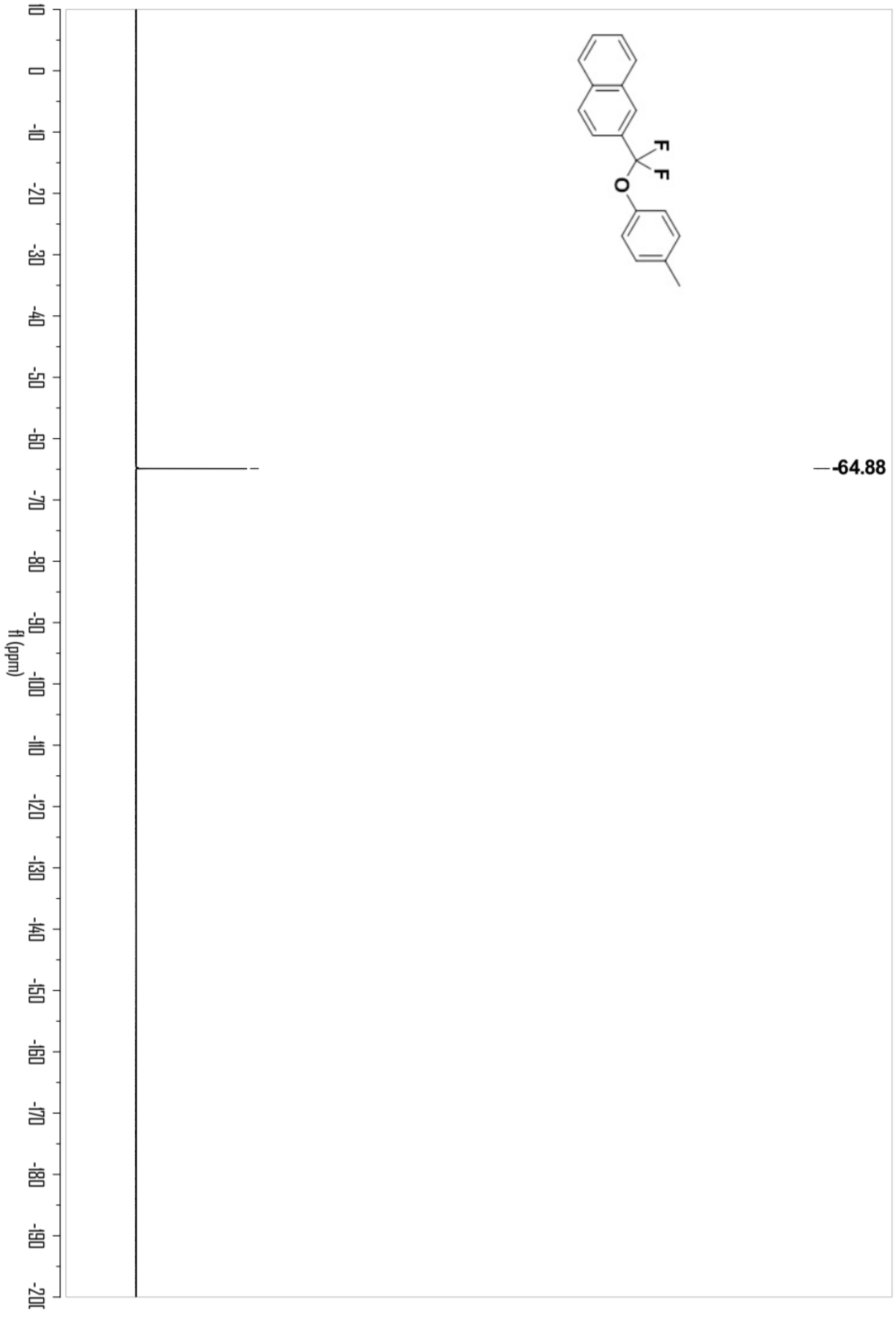
-64.88
f1 (ppm)
F
F
O

^{1}H, ^{13}C and ^{19}F NMR spectra of compound 3aa

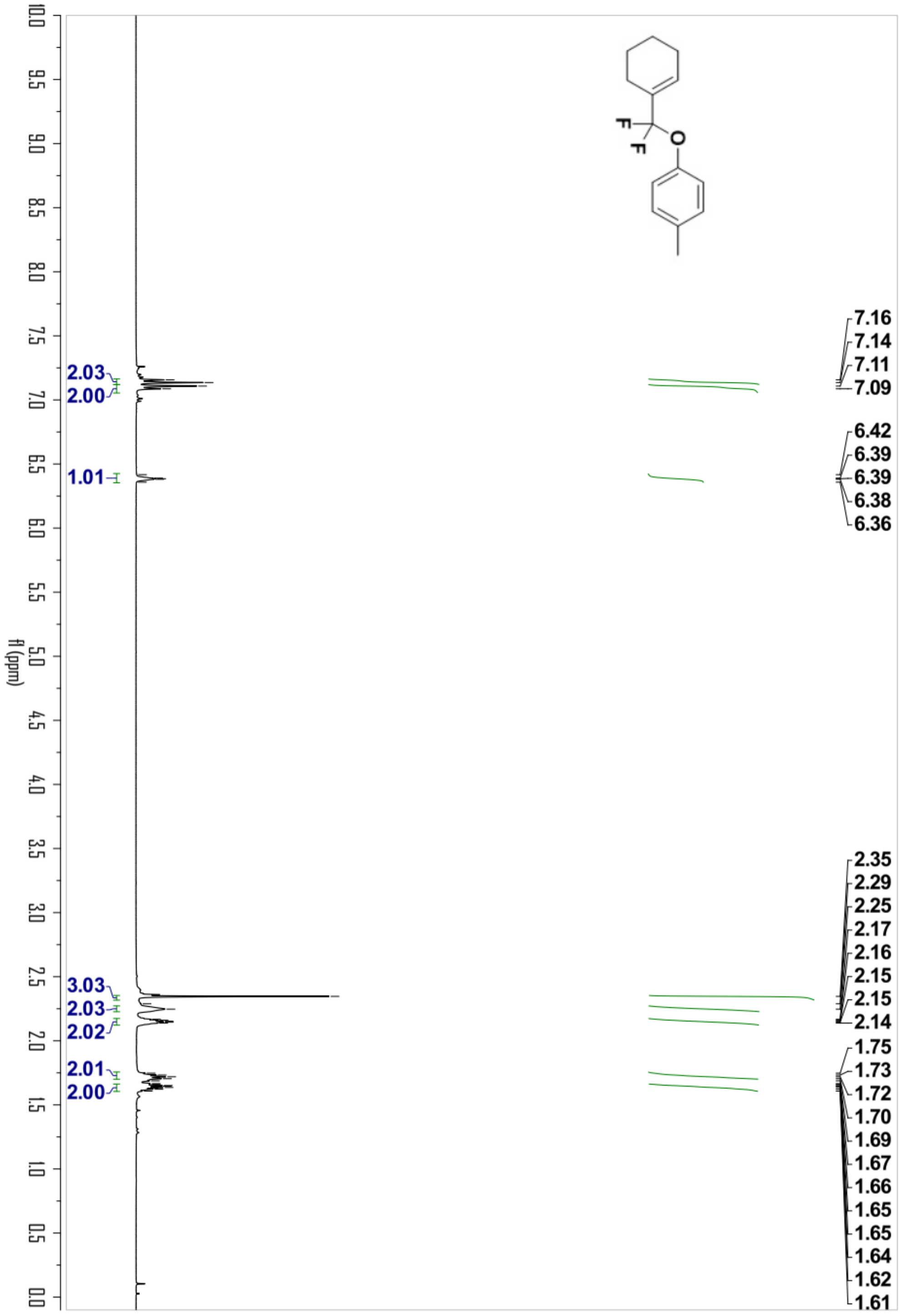

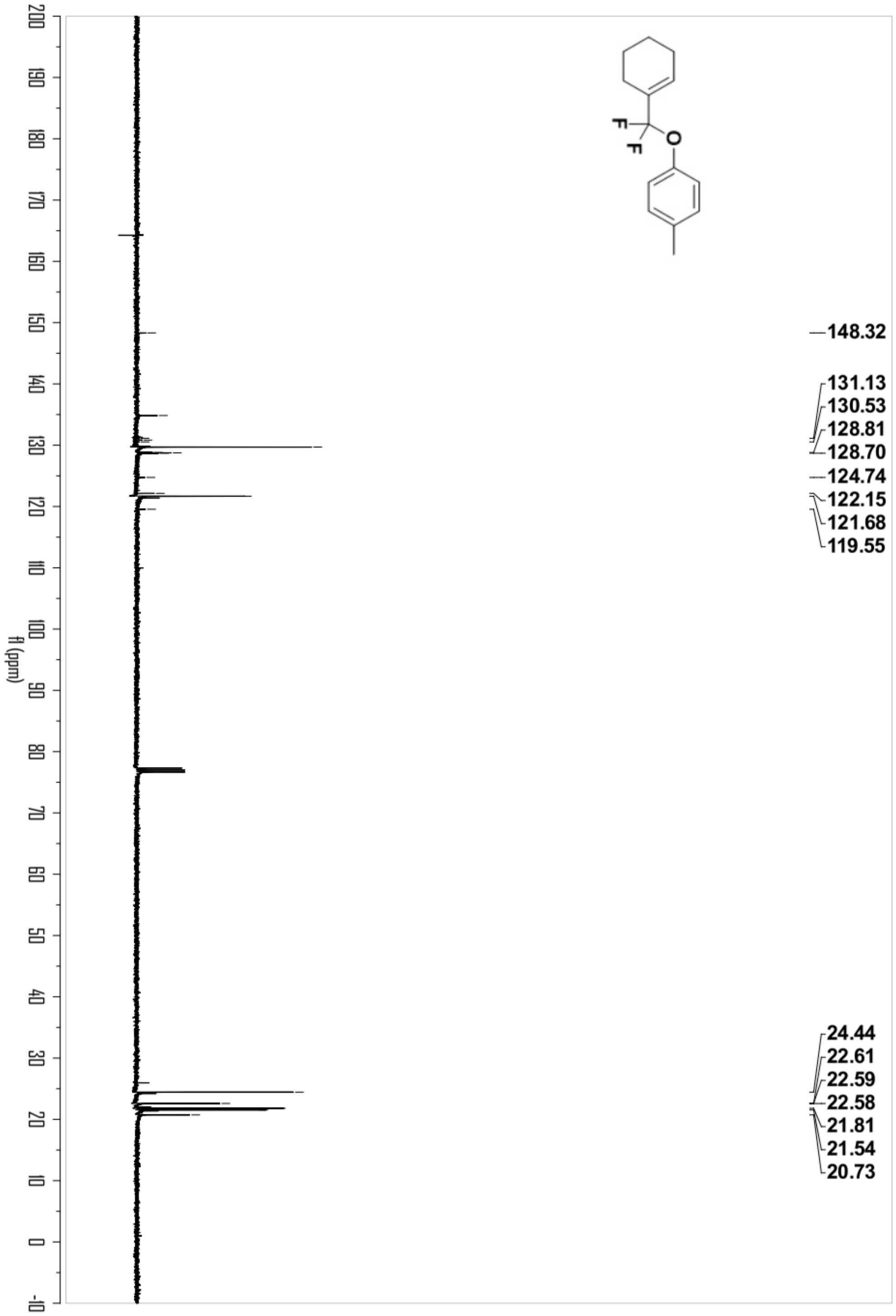
148.32
131.13
130.53
128.81
128.70
124.74
122.15
121.68
119.55
24.44
22.61
22.59
22.58
21.81
21.54
20.73
f1 (ppm)
F
F
O

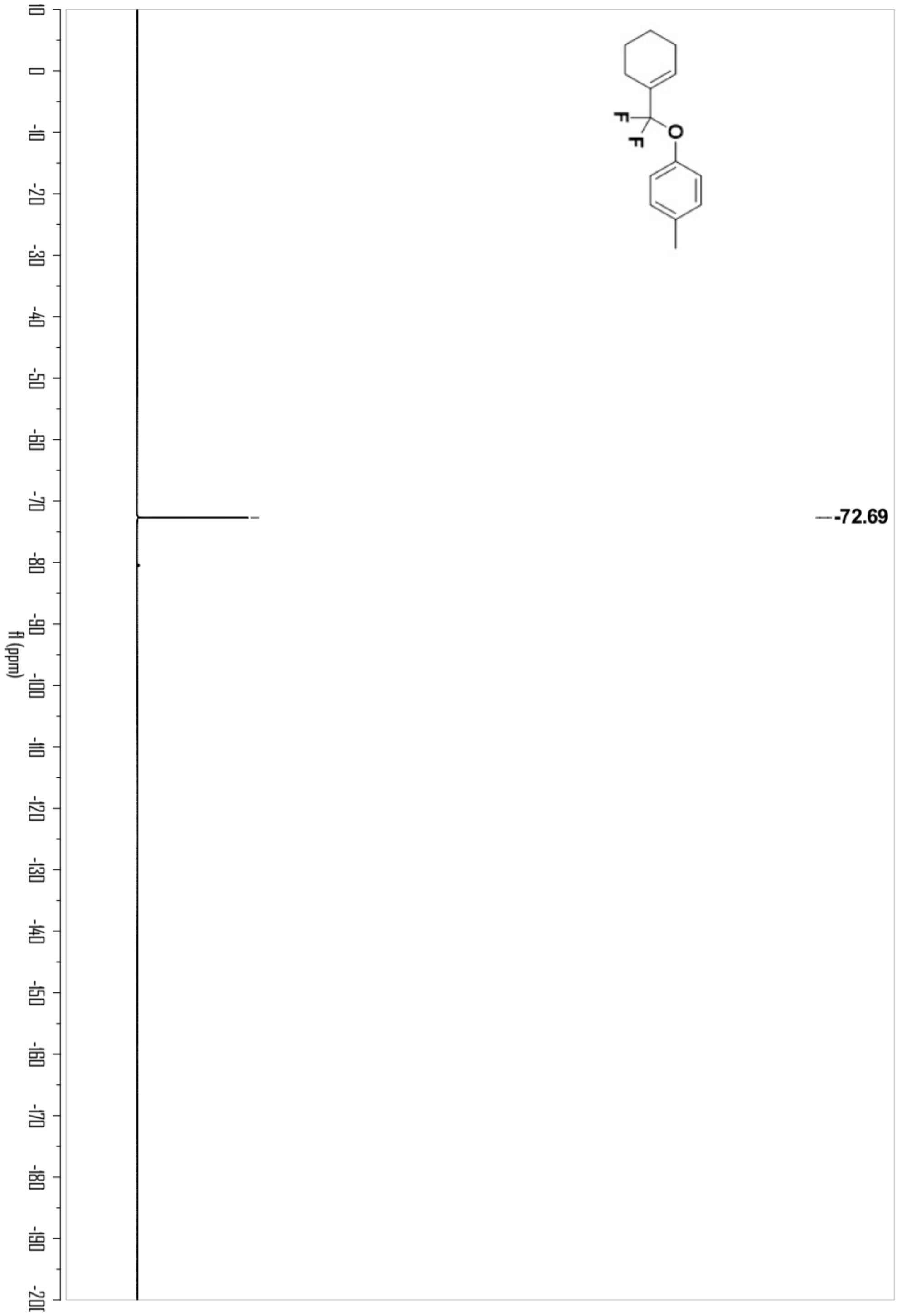
-72.69
f1 (ppm)

^{1}H, ^{13}C and ^{19}F NMR spectra of compound 3ab

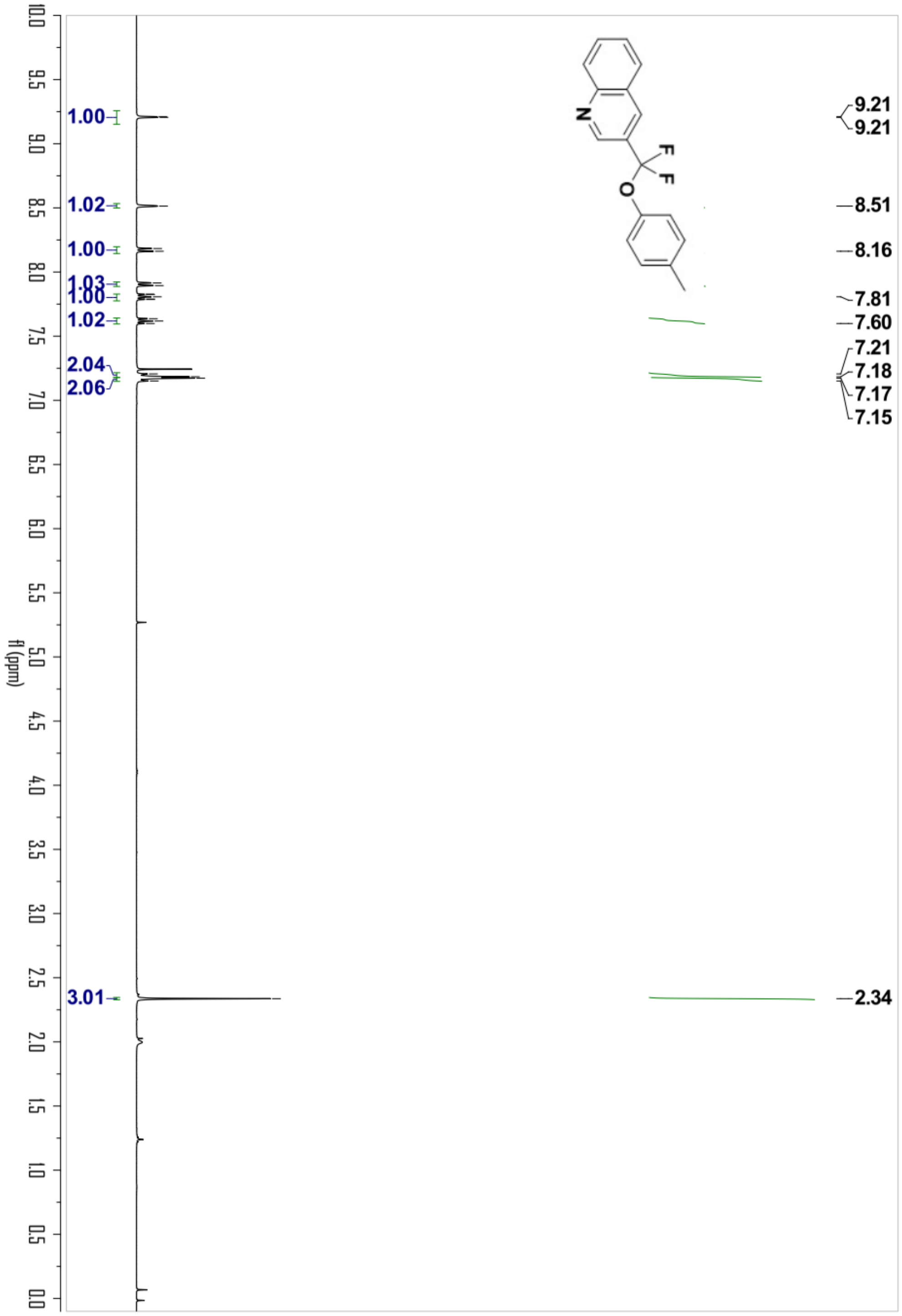

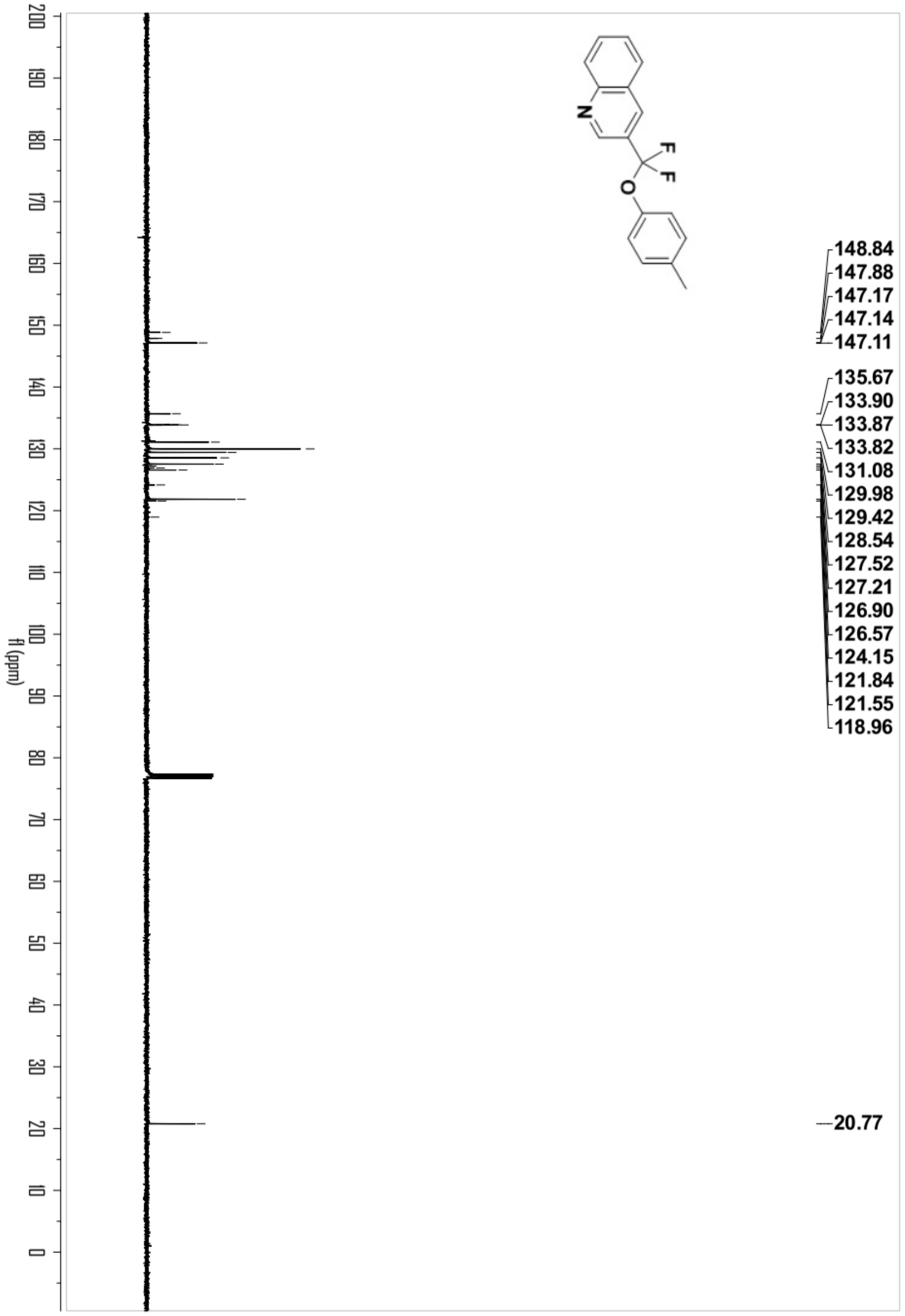

148.84
147.88
147.17
147.14
147.11
135.67
133.90
133.87
133.82
131.08
129.98
129.42
128.54
127.52
127.21
126.90
126.57
124.15
121.84
121.55
118.96
20.77
f1 (ppm)

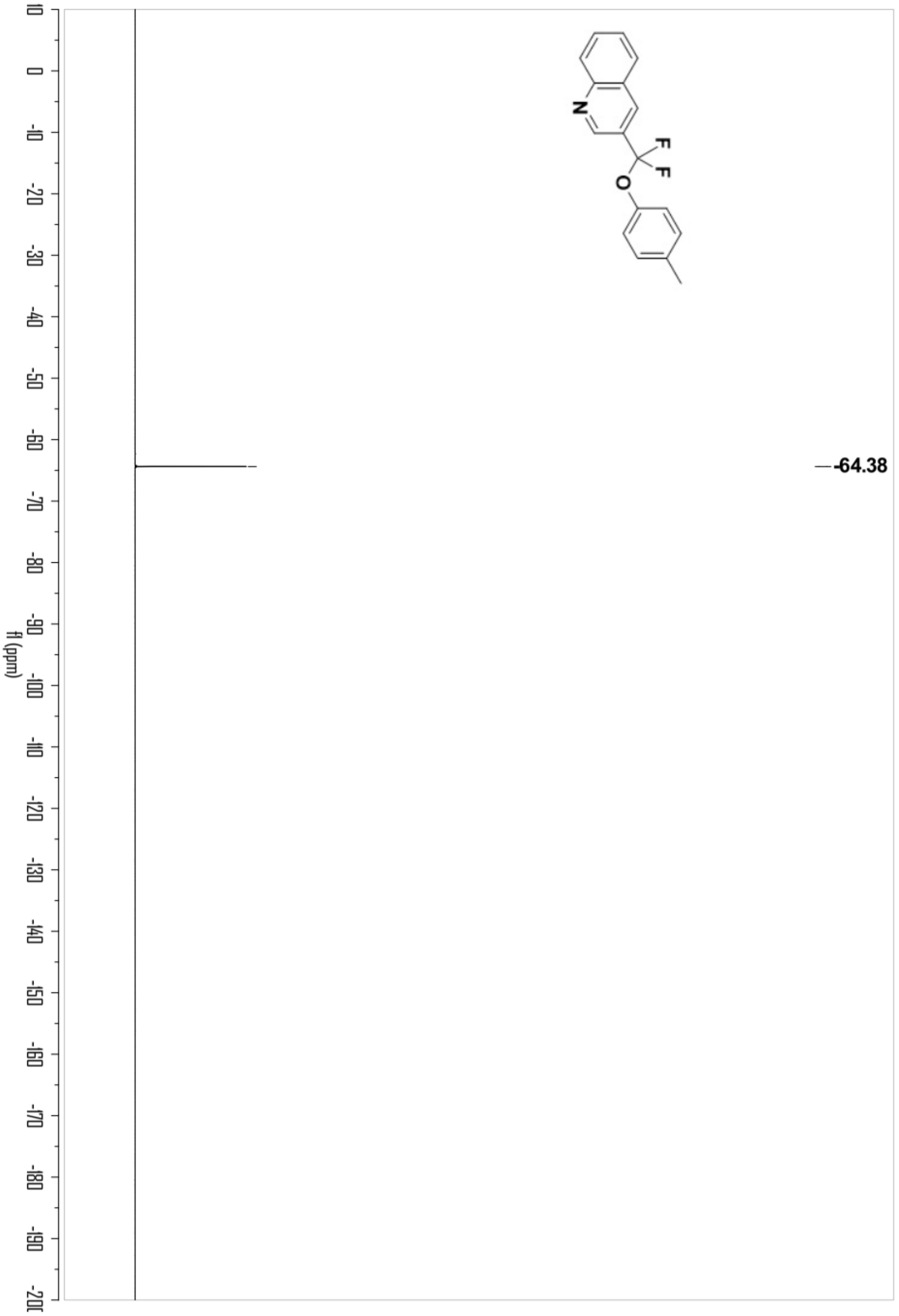

-64.38
f1 (ppm)

^{1}H, ^{13}C and ^{19}F NMR spectra of compound 3ac

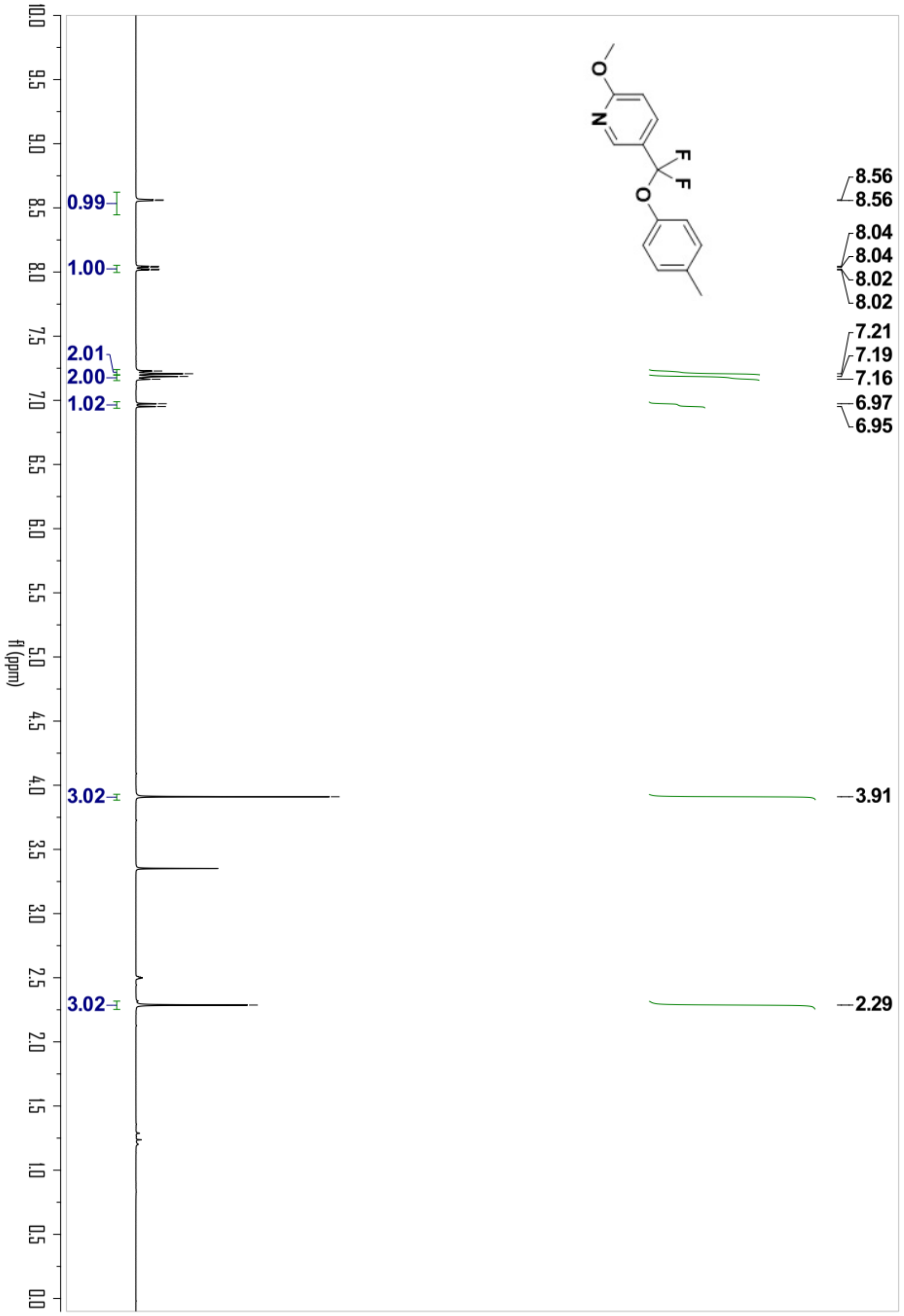

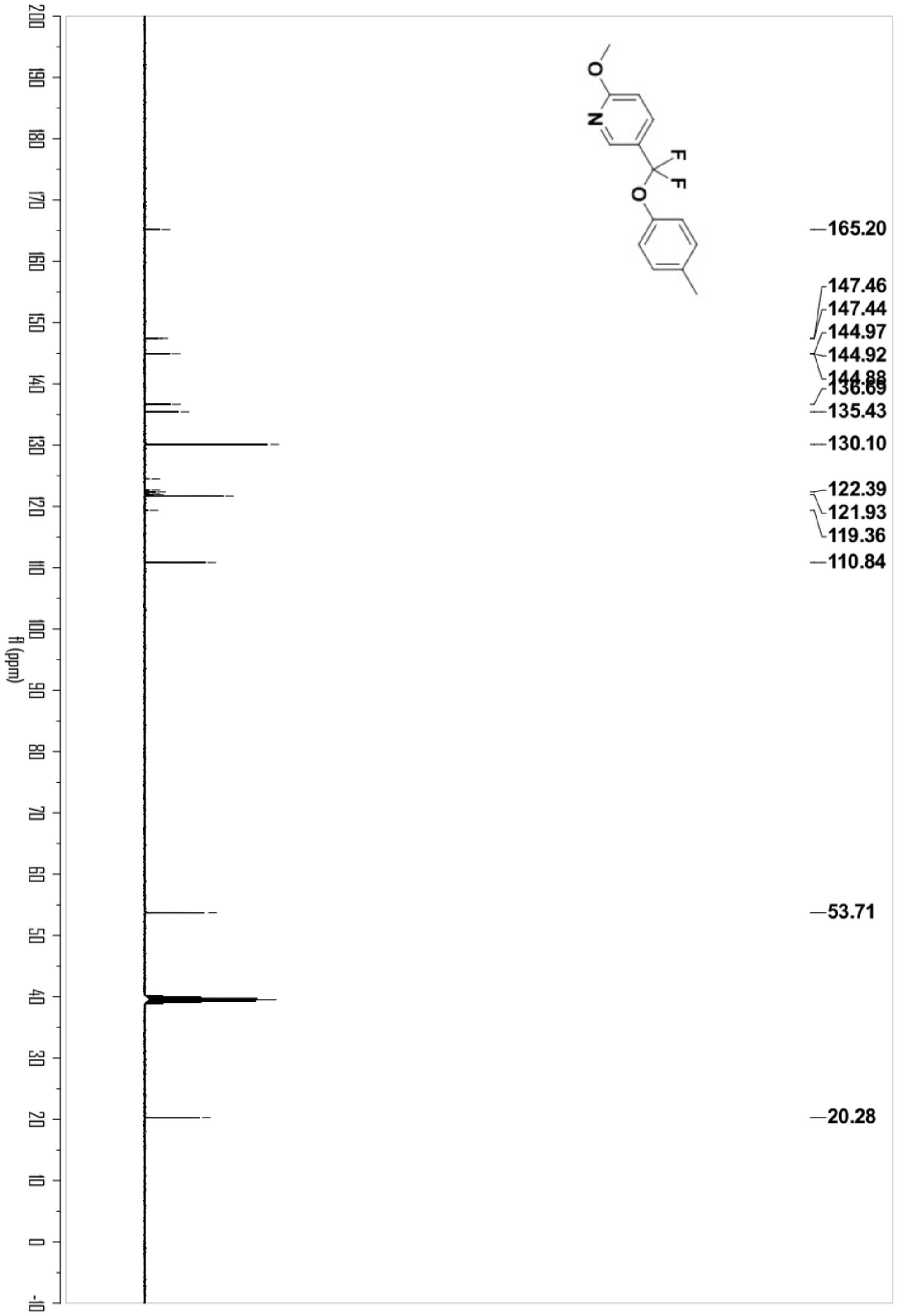
165.20
147.46
147.44
144.97
144.92
144.88
136.69
135.43
130.10
122.39
121.93
119.36
110.84
53.71
20.28
f1 (ppm)

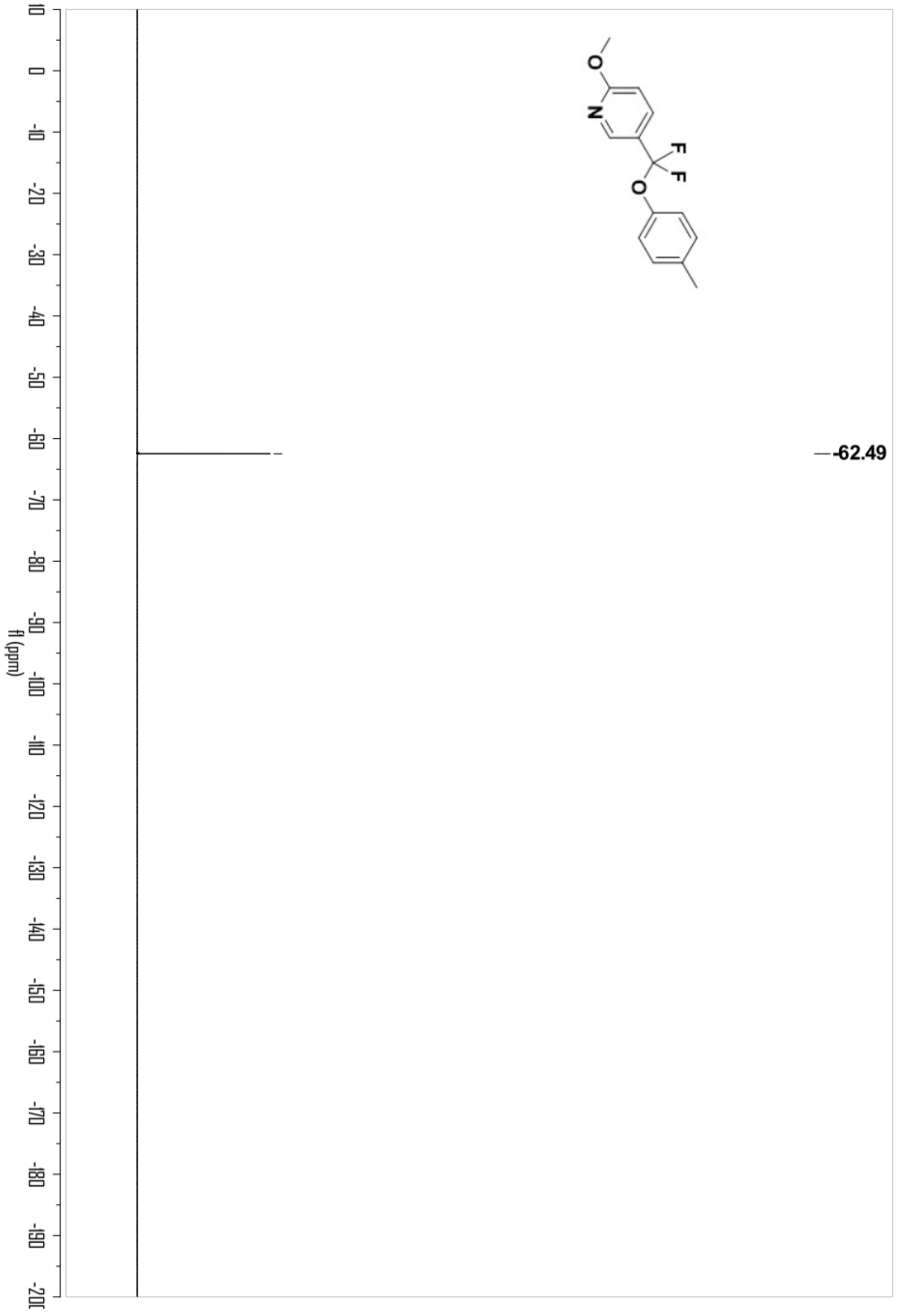
-62.49
f1 (ppm)
O
N
F
F
O

^{1}H, ^{13}C and ^{19}F NMR spectra of compound 3ad

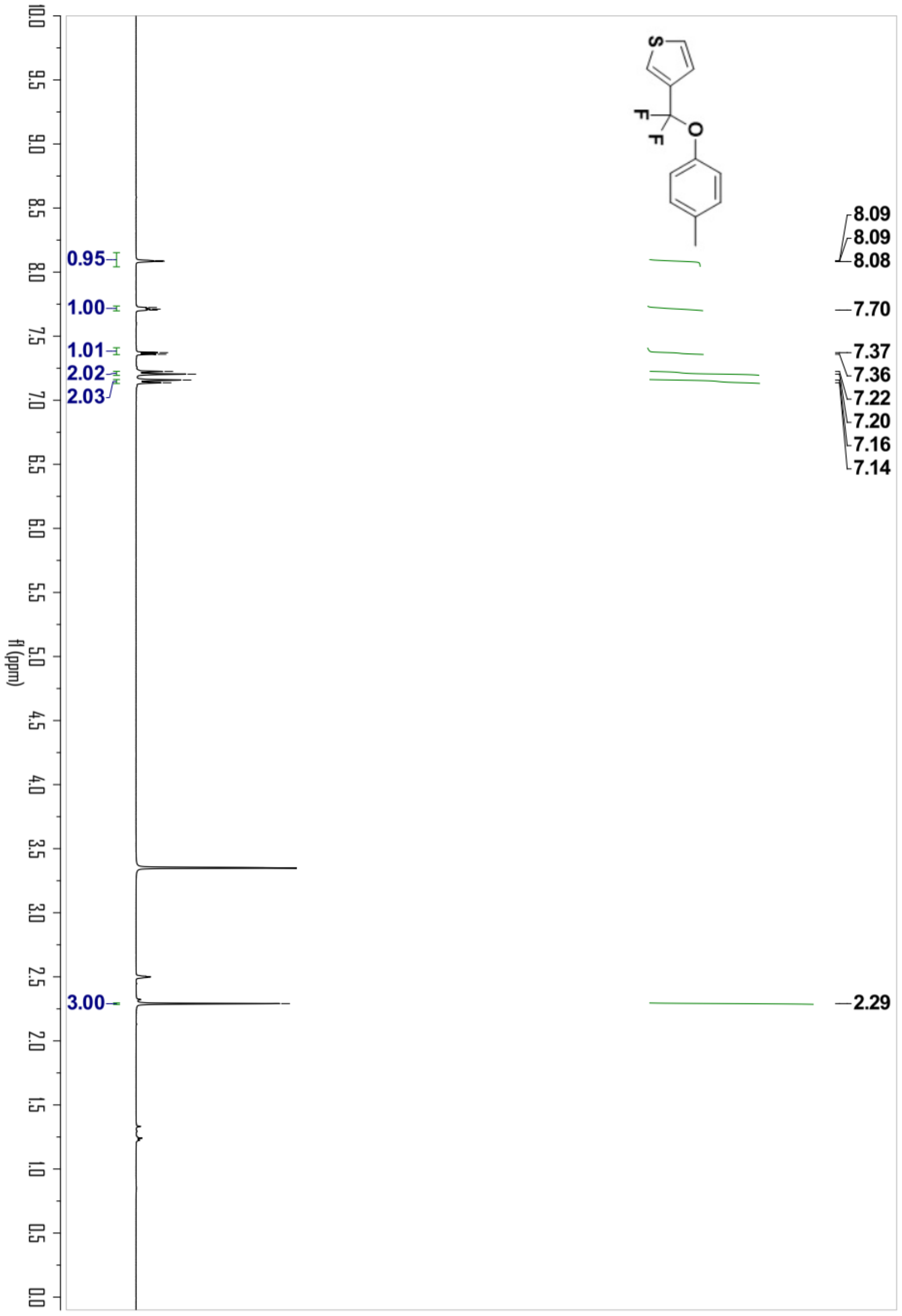

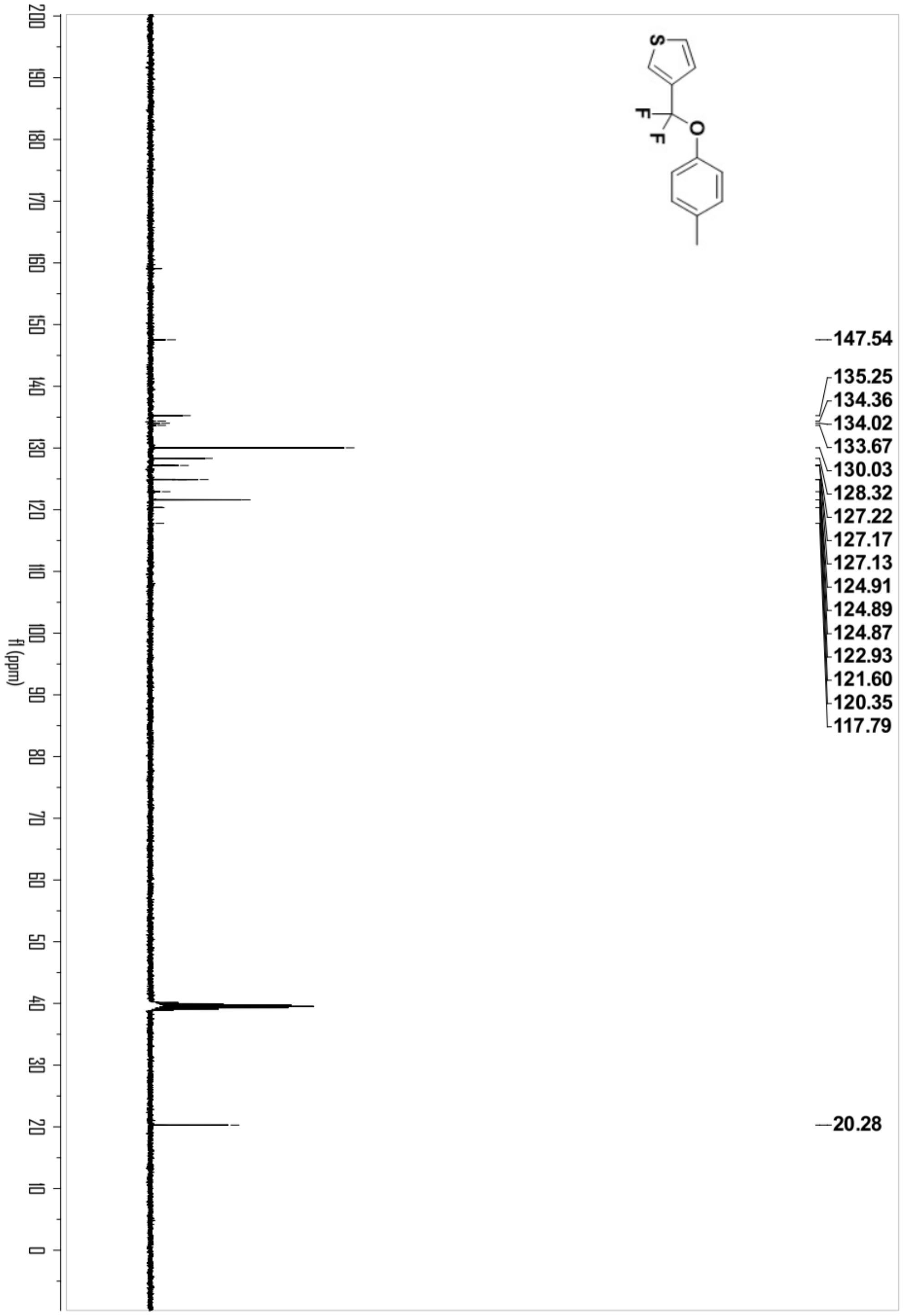
147.54
135.25
134.36
134.02
133.67
130.03
128.32
127.22
127.17
127.13
124.91
124.89
124.87
122.93
121.60
120.35
117.79
20.28
f1 (ppm)

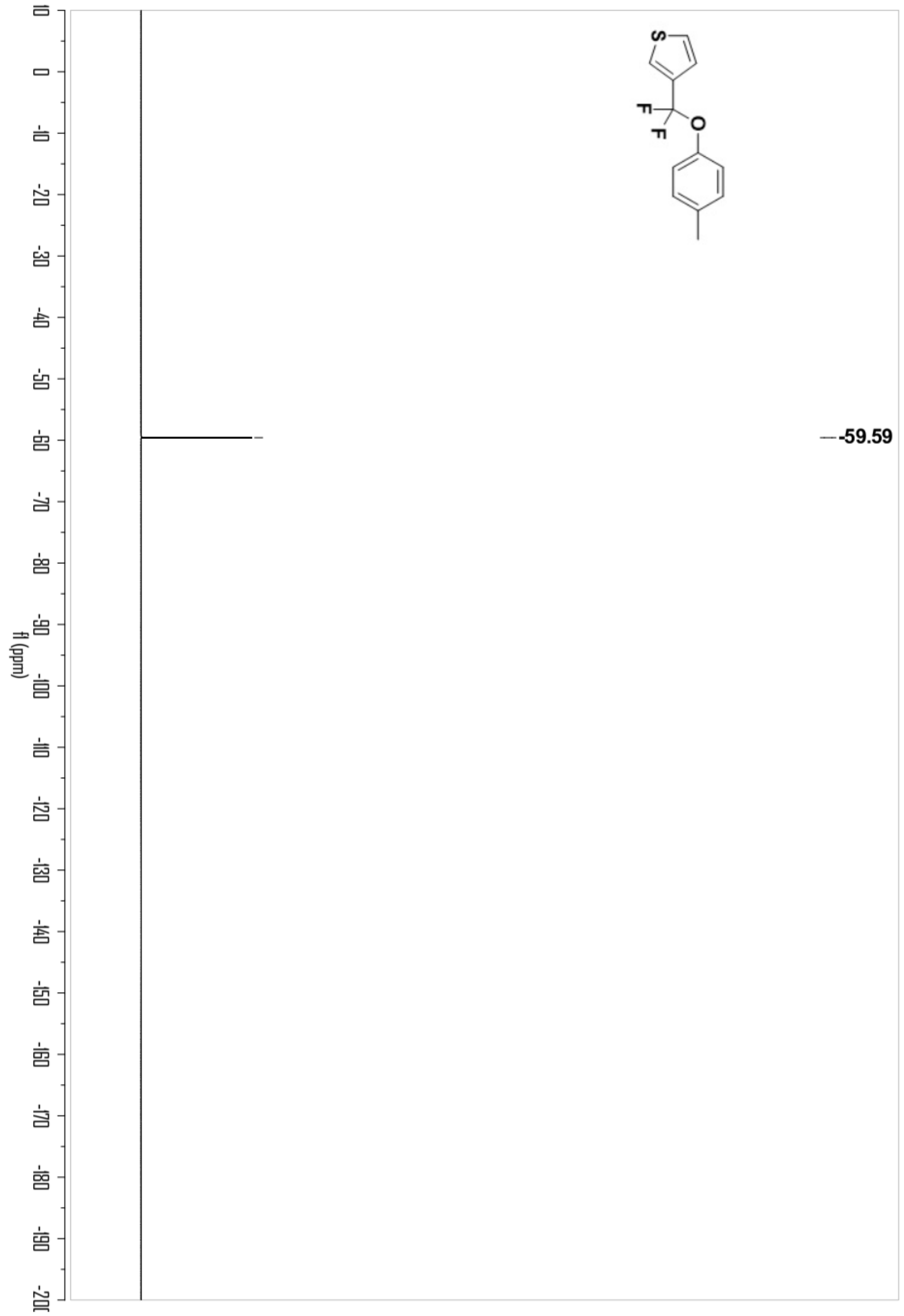
S
F
F
O
-59.59
f1 (ppm)

^{1}H, ^{13}C and ^{19}F NMR spectra of compound 3ae

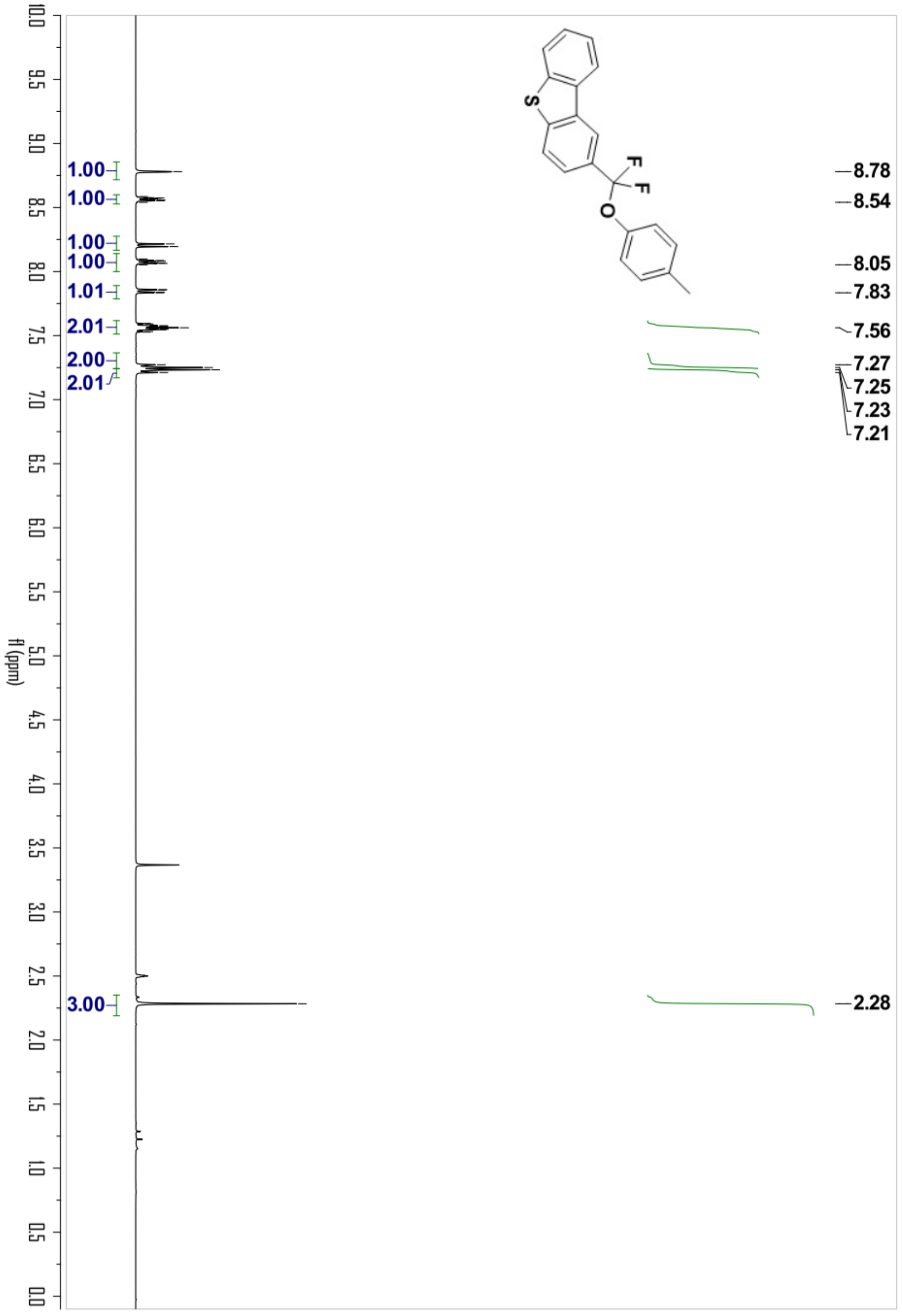

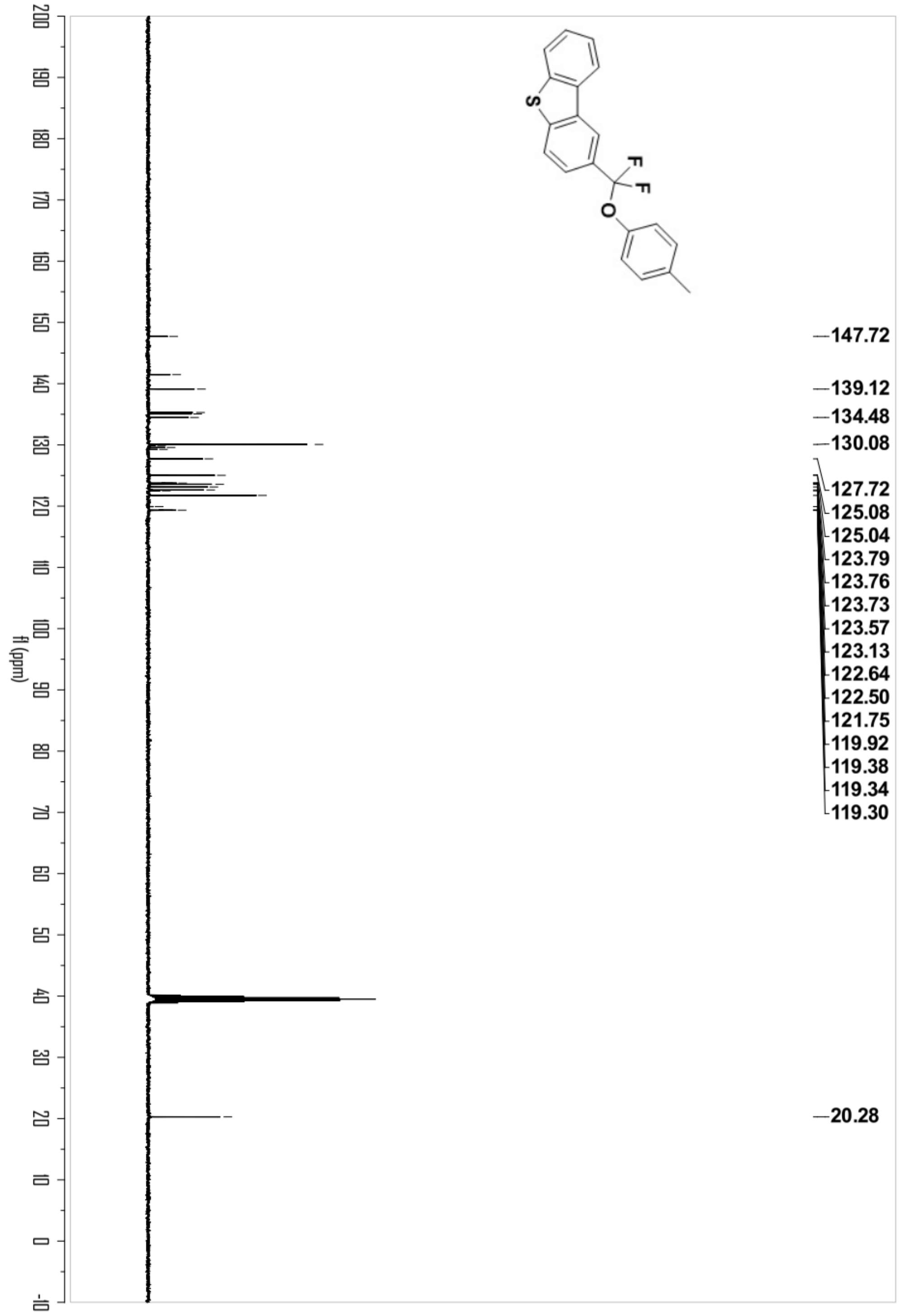
S
F
F
O
147.72
139.12
134.48
130.08
127.72
125.08
125.04
123.79
123.76
123.73
123.57
123.13
122.64
122.50
121.75
119.92
119.38
119.34
119.30
20.28
f1 (ppm)

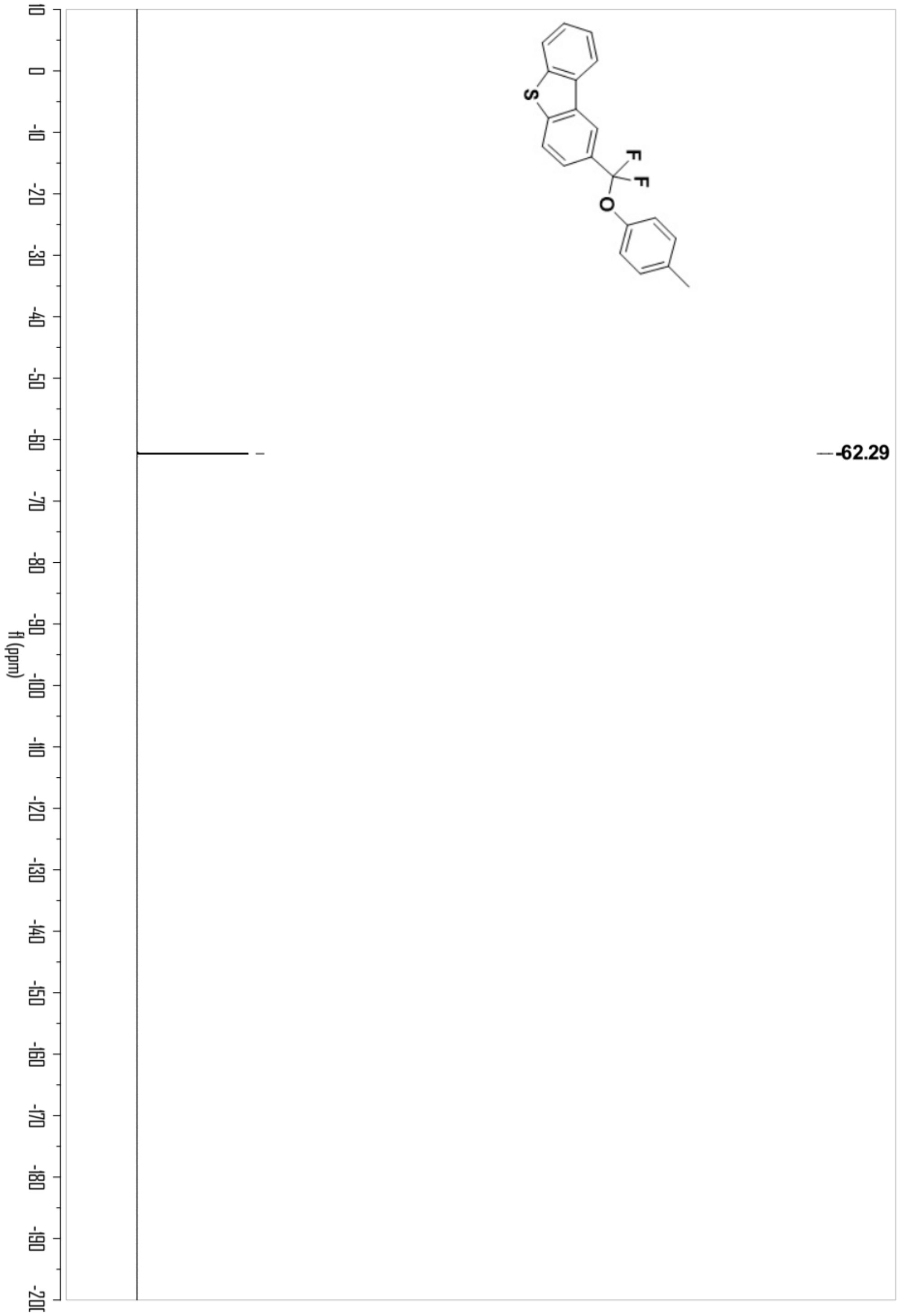
-62.29
f1 (ppm)

^{1}H, ^{13}C and ^{19}F NMR spectra of compound 3af

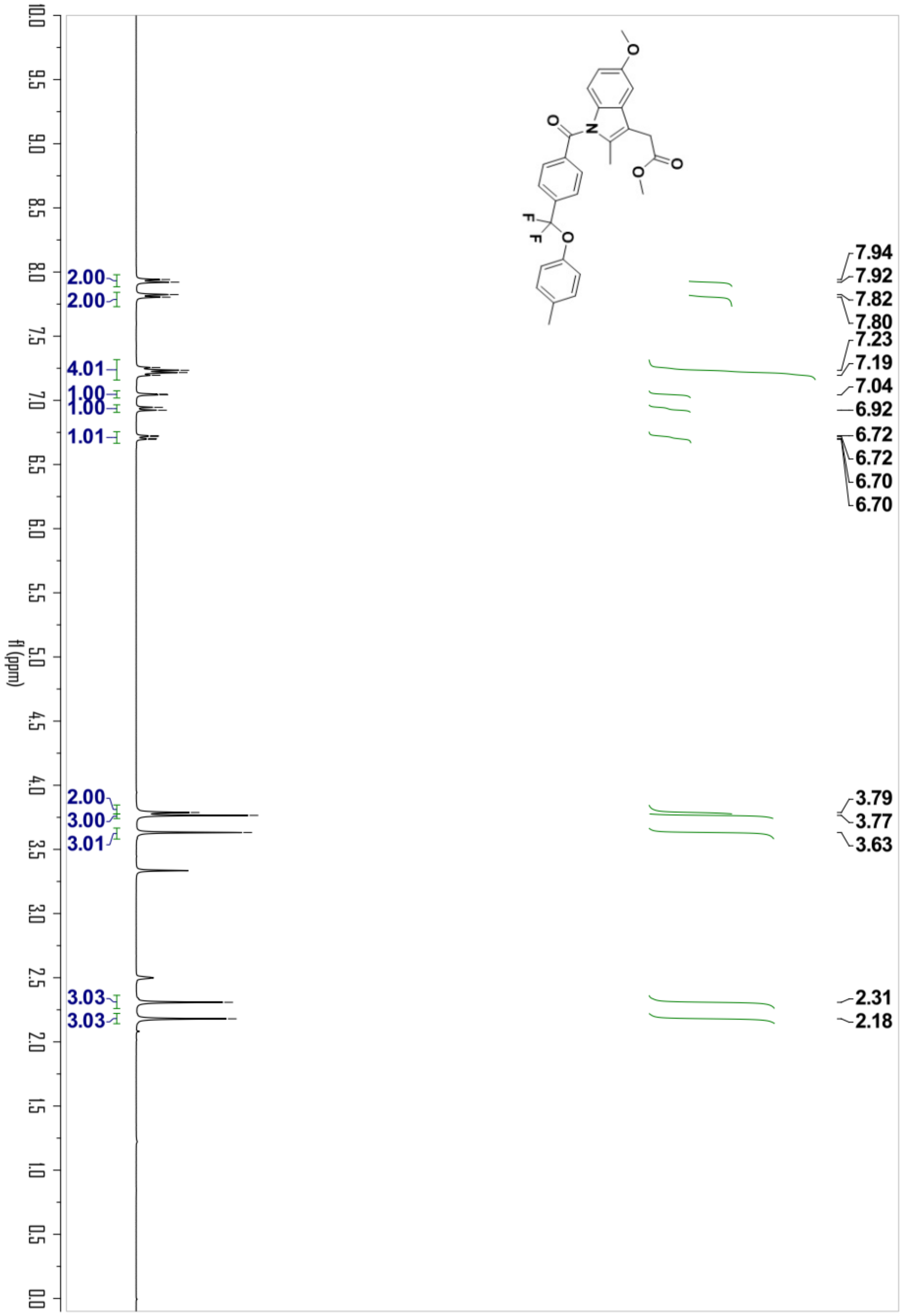

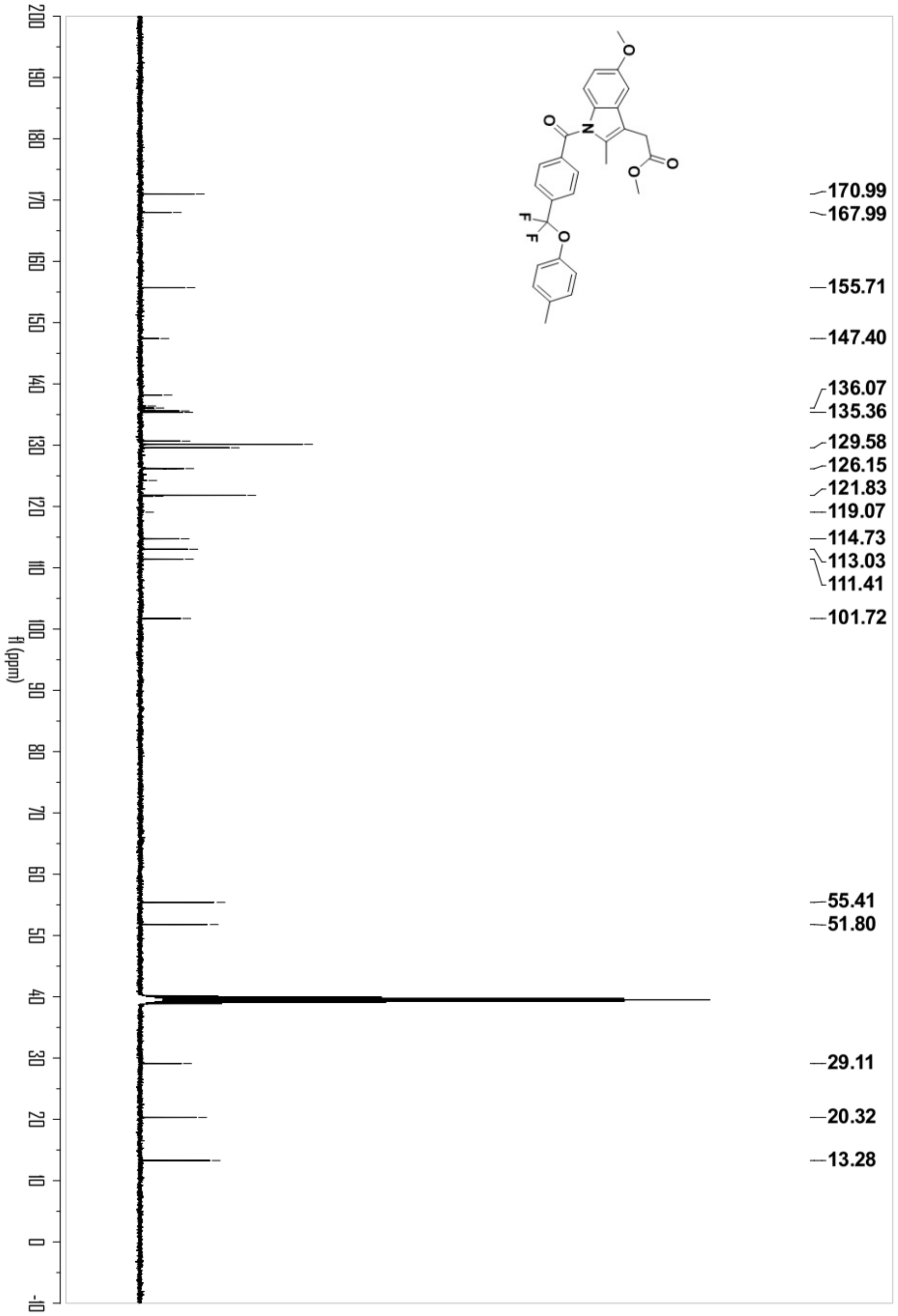
170.99
167.99
155.71
147.40
136.07
135.36
129.58
126.15
121.83
119.07
114.73
113.03
111.41
101.72
55.41
51.80
29.11
20.32
13.28
f1 (ppm)

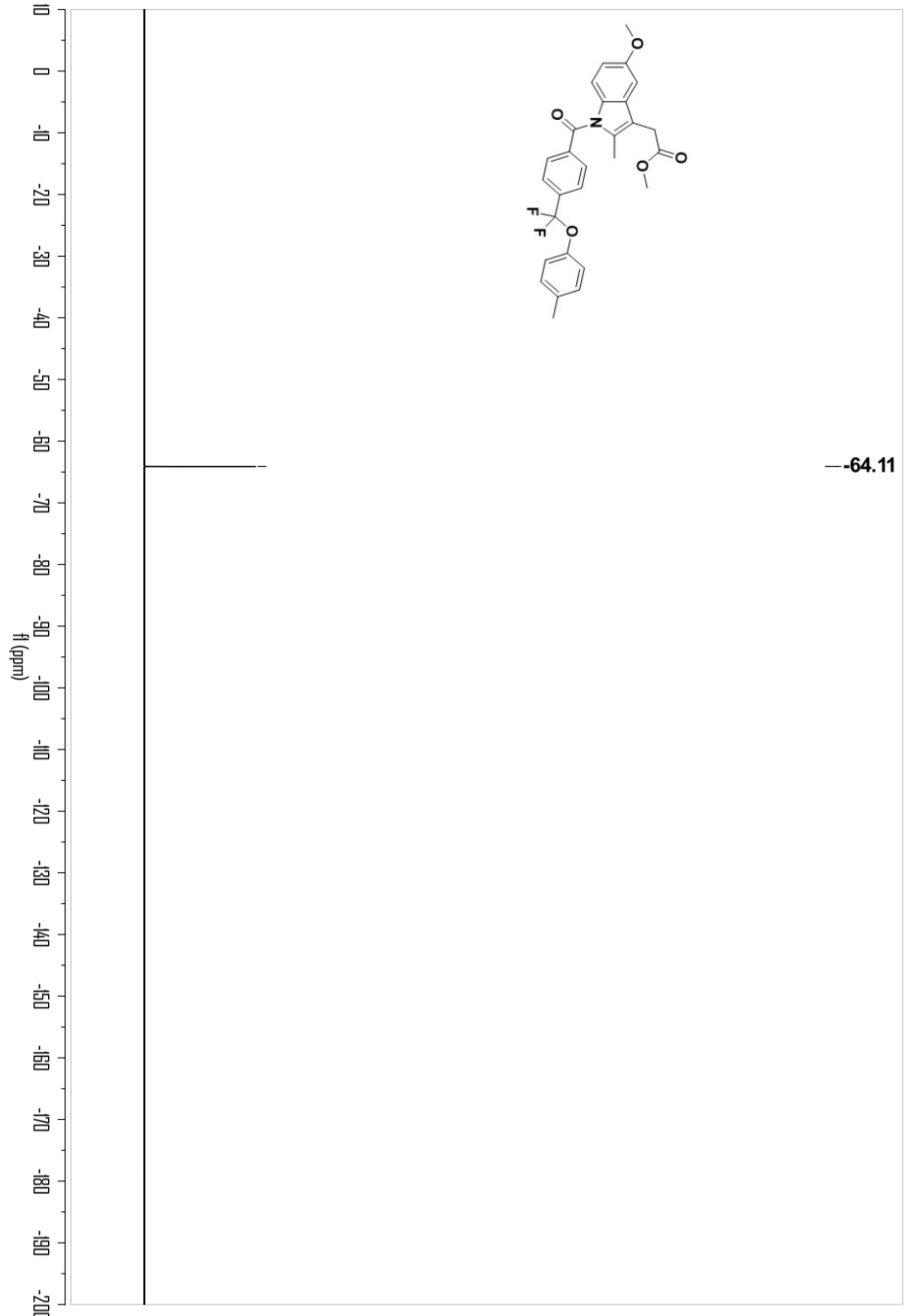
-64.11
f1 (ppm)

^{1}H, ^{13}C and ^{19}F NMR spectra of compound 3g

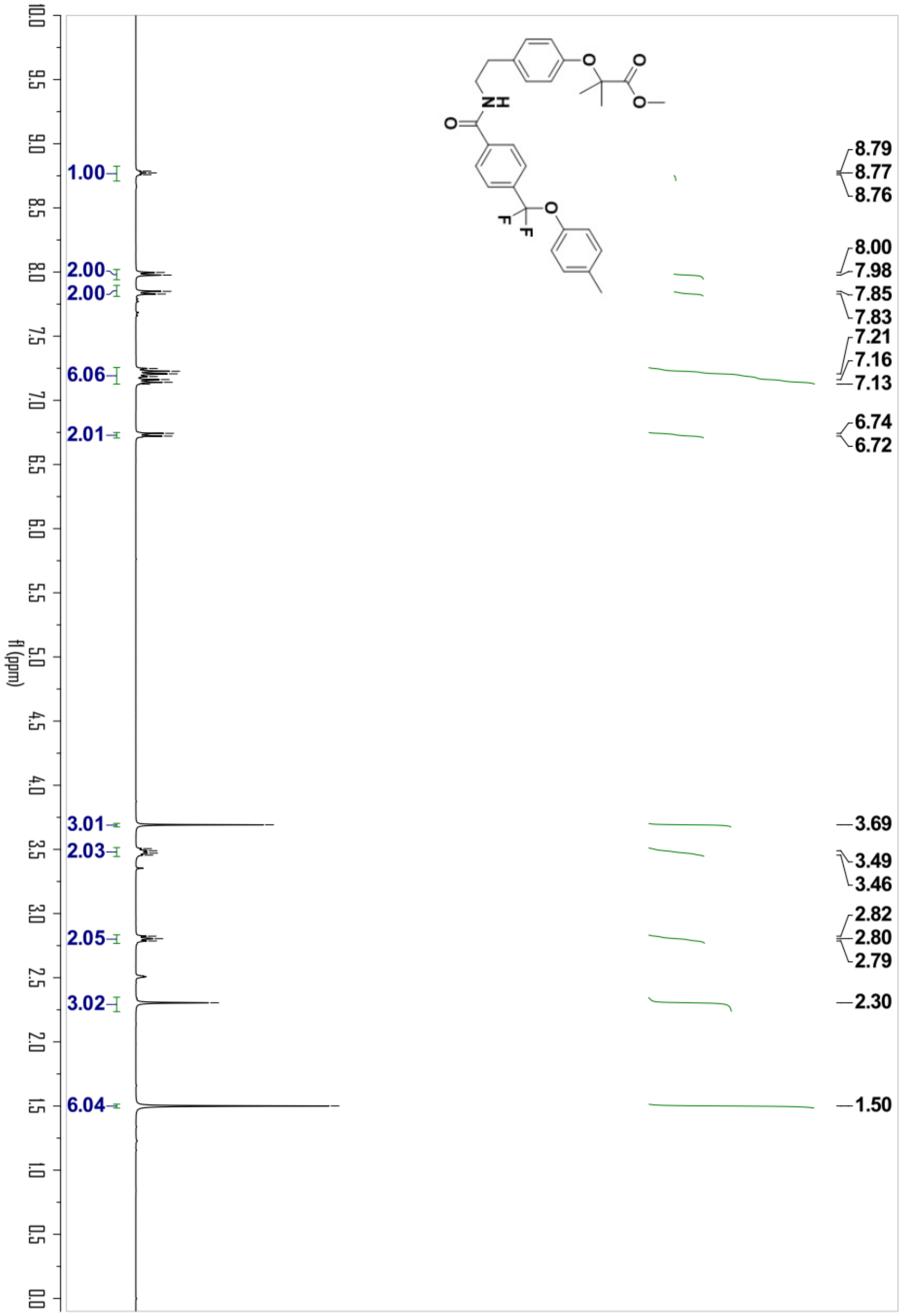

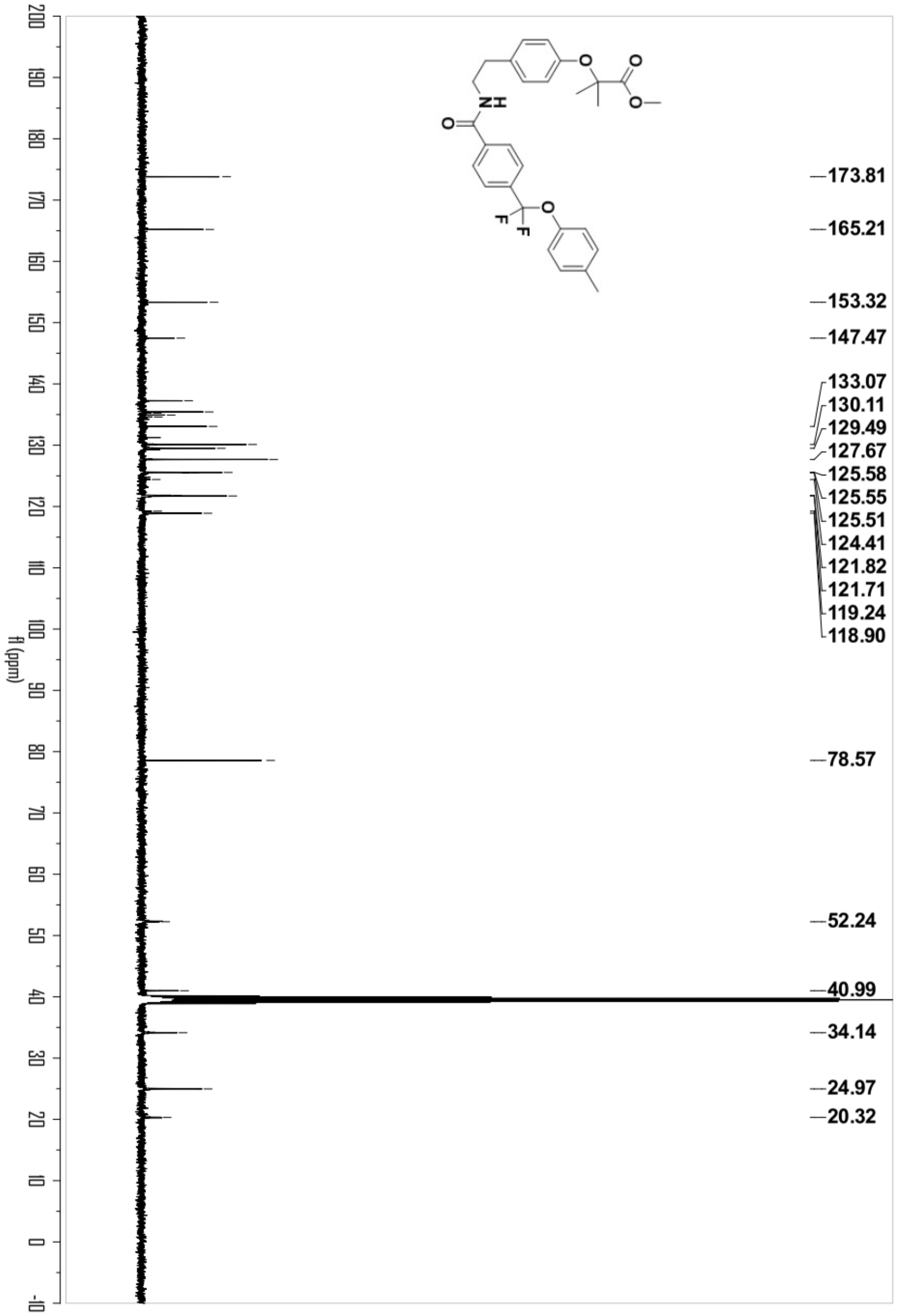
173.81
165.21
153.32
147.47
133.07
130.11
129.49
127.67
125.58
125.55
125.51
124.41
121.82
121.71
119.24
118.90
78.57
52.24
40.99
34.14
24.97
20.32
f1 (ppm)

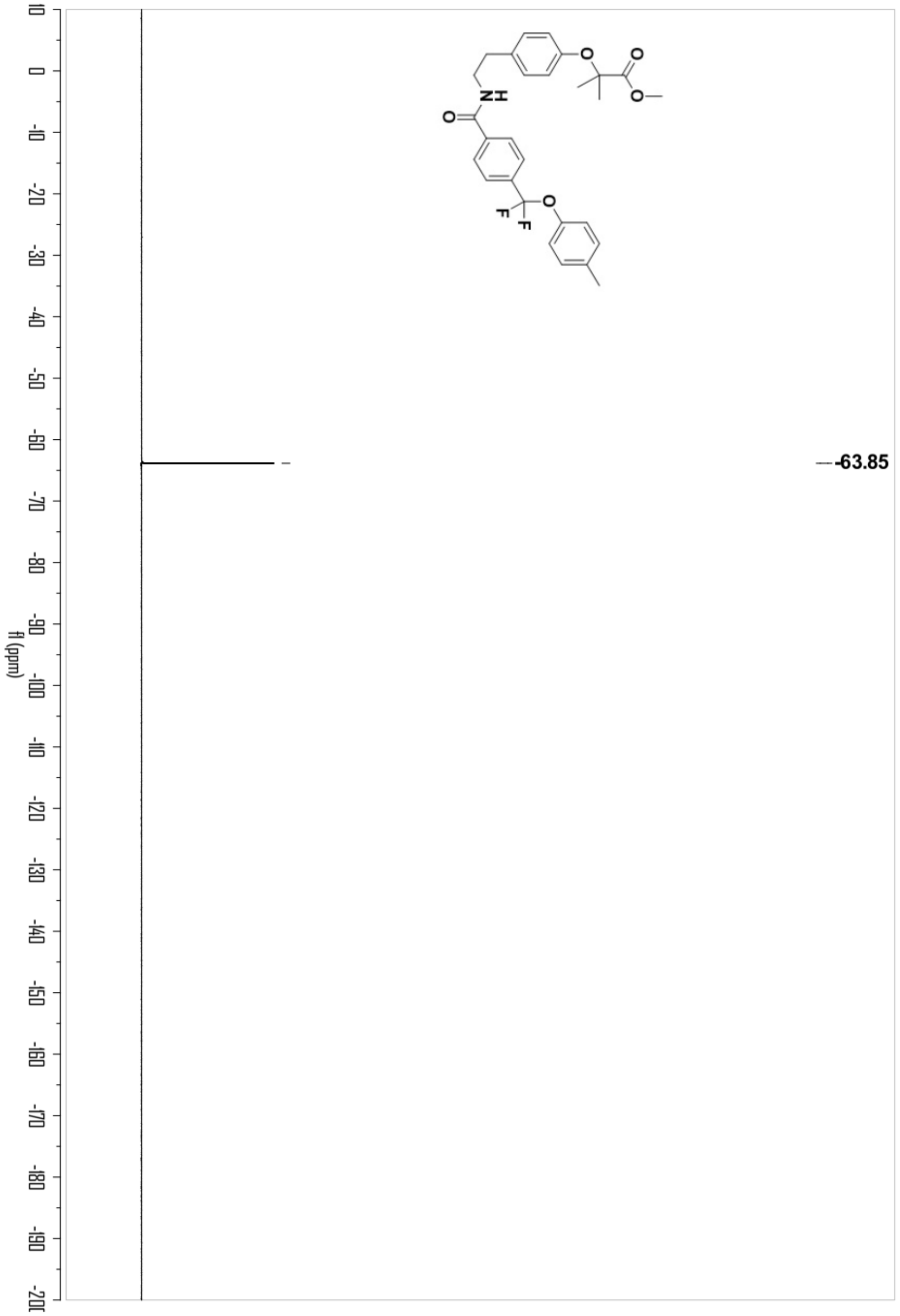
-63.85
f1 (ppm)
10
0
-10
-20
-30
-40
-50
-60
-70
-80
-90
-100
-110
-120
-130
-140
-150
-160
-170
-180
-190
-200

^{1}H, ^{13}C and ^{19}F NMR spectra of compound 3h

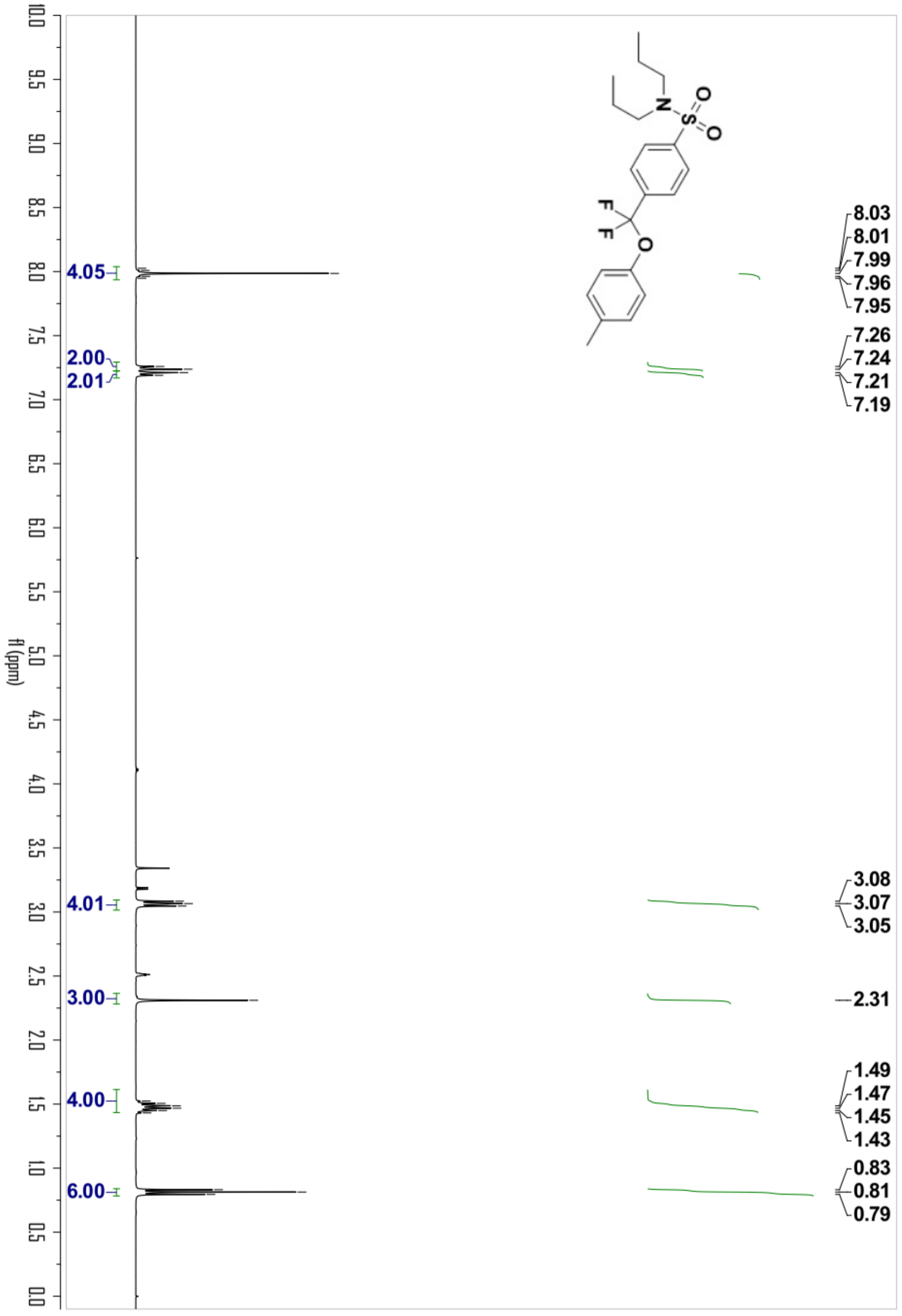

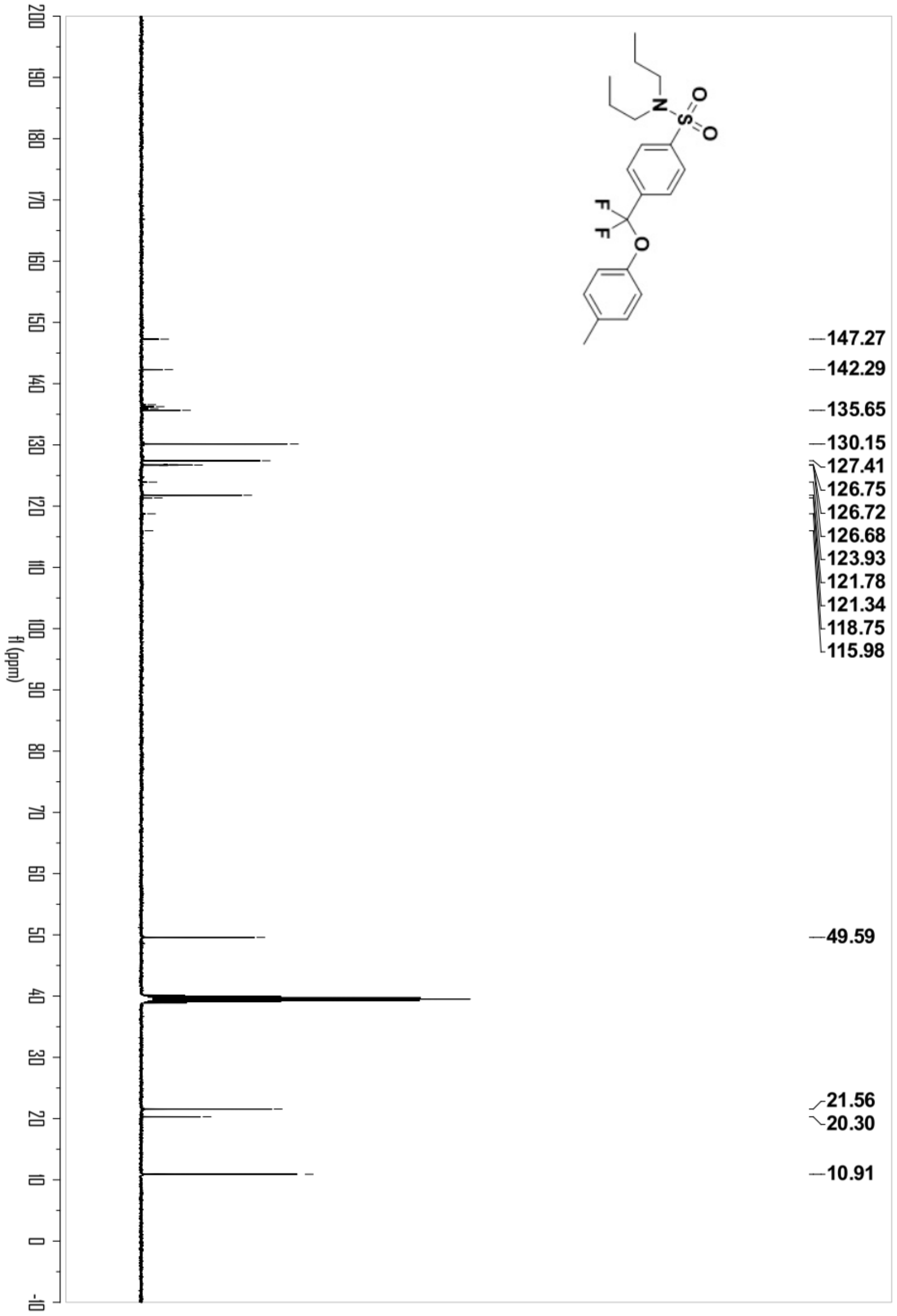
147.27
142.29
135.65
130.15
127.41
126.75
126.72
126.68
123.93
121.78
121.34
118.75
115.98
49.59
21.56
20.30
10.91
f1 (ppm)

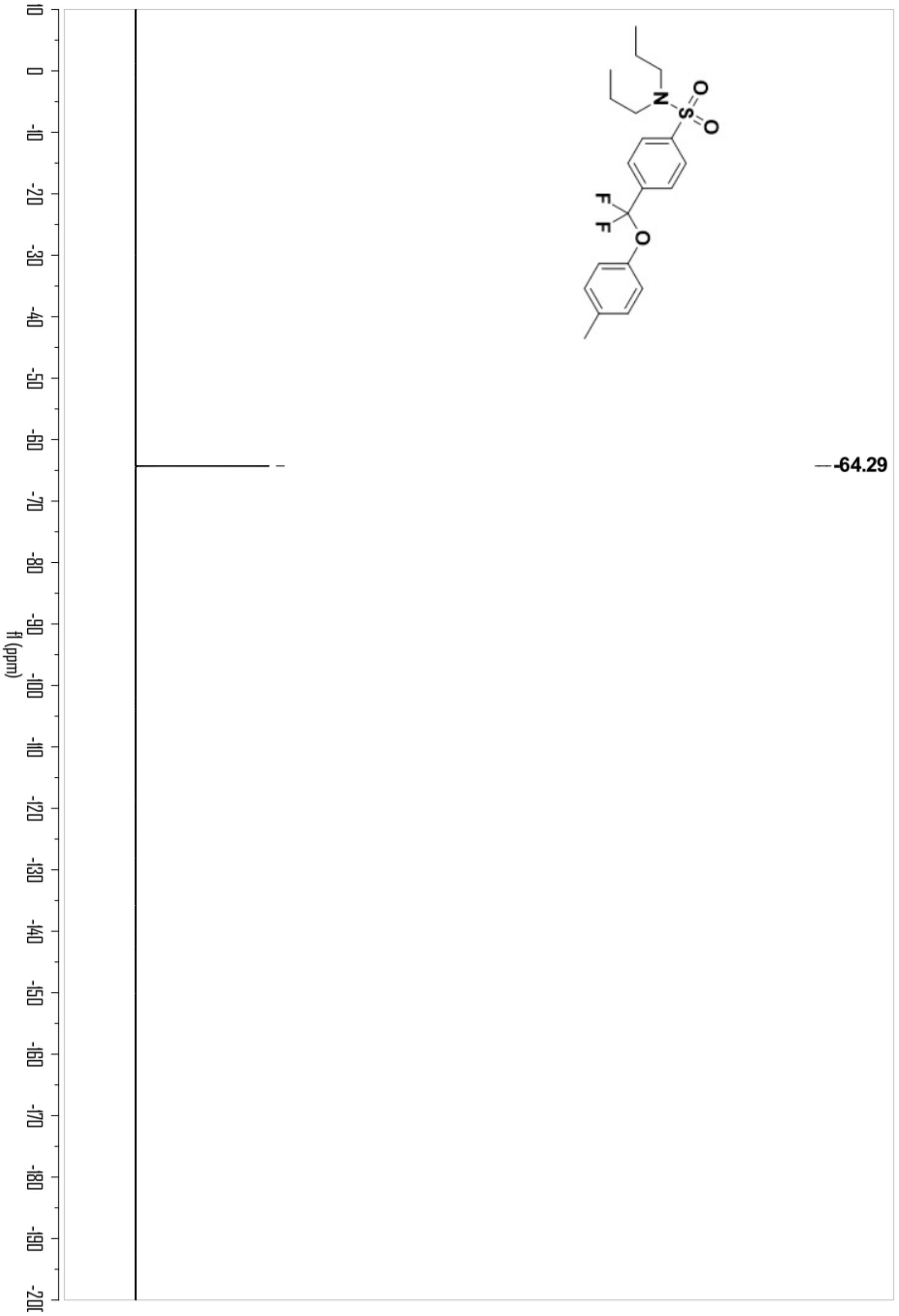
-64.29
f1 (ppm)

^{1}H, ^{13}C and ^{19}F NMR spectra of compound 3i

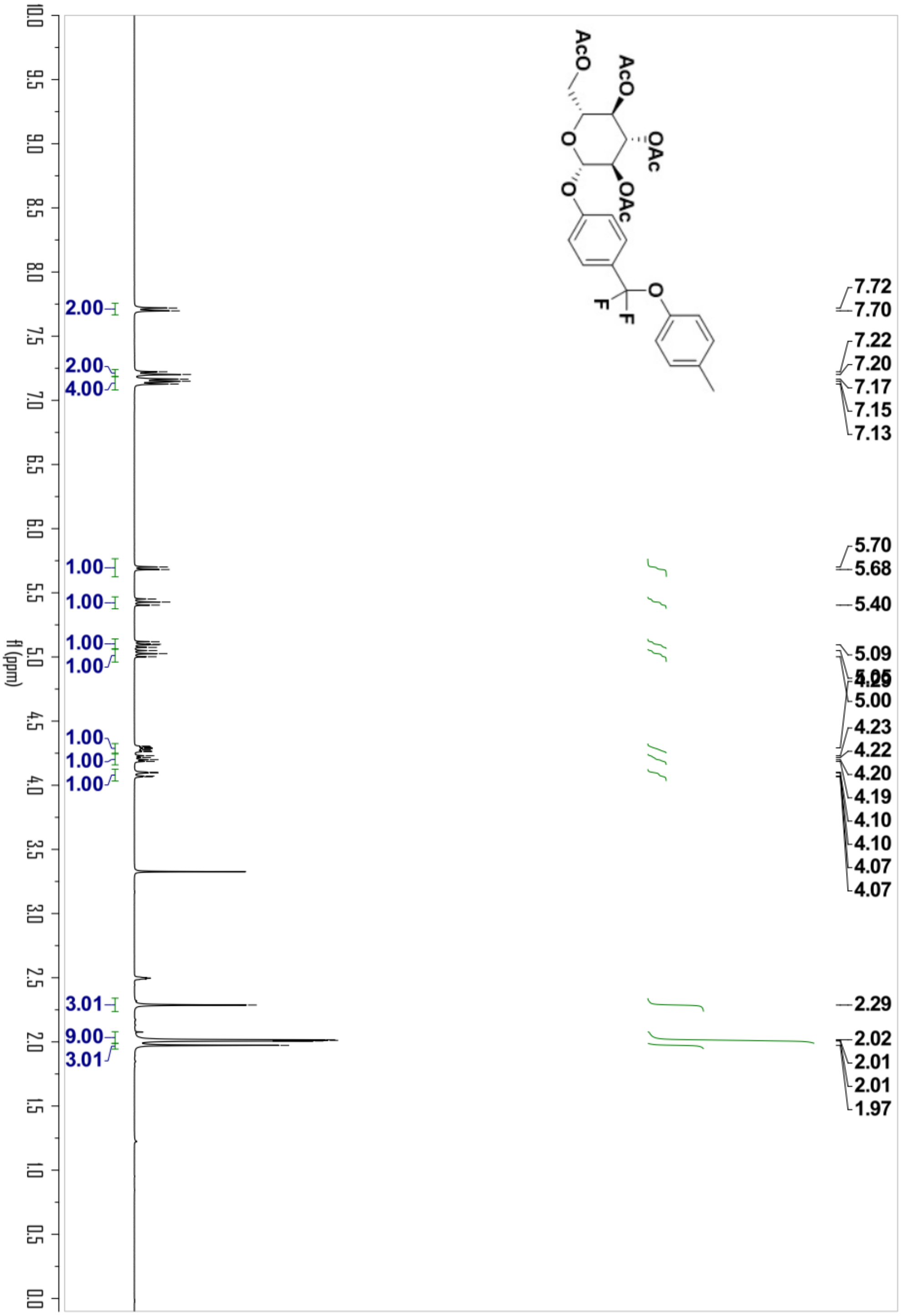

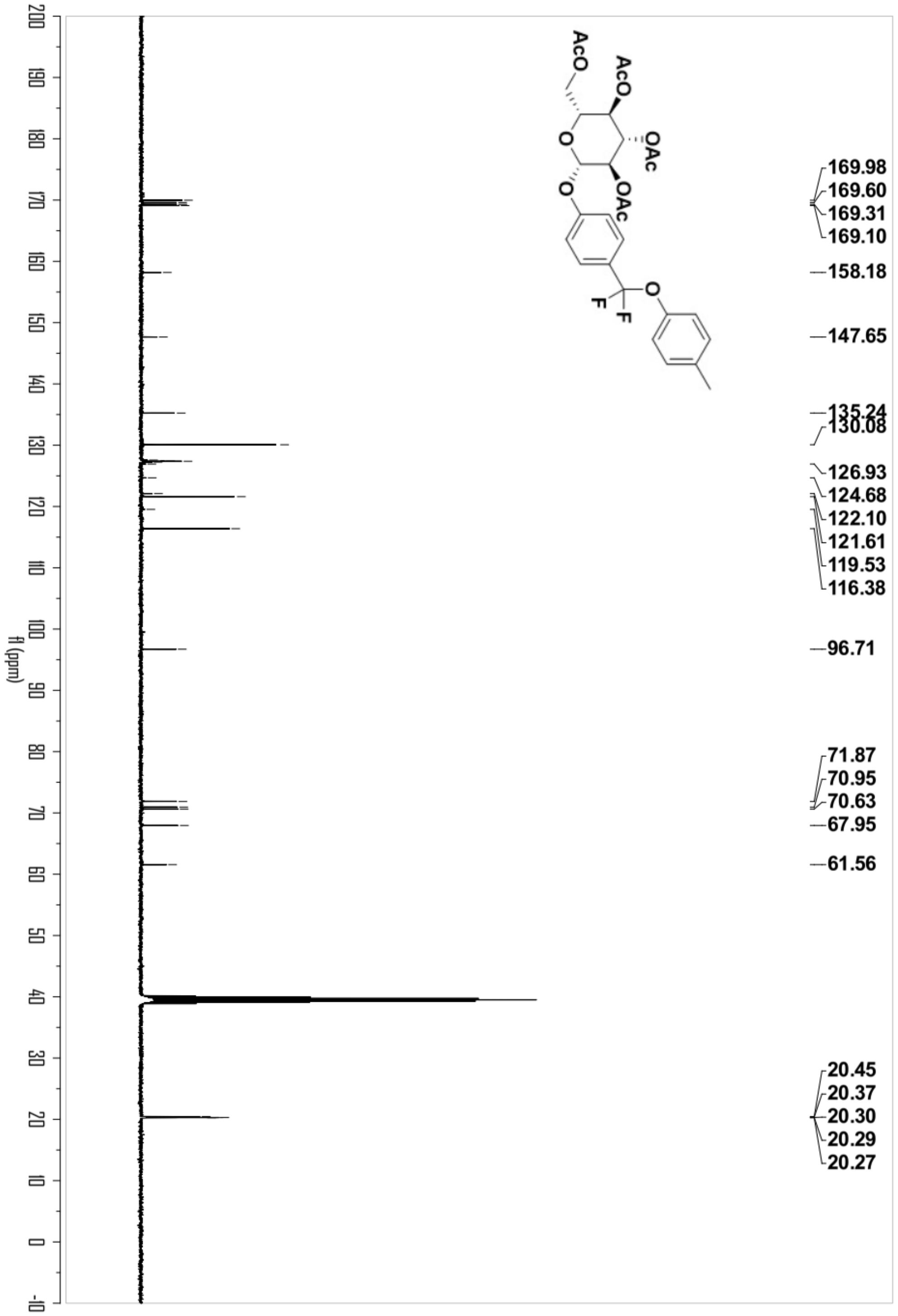
169.98
169.60
169.31
169.10
158.18
147.65
135.24
130.08
126.93
124.68
122.10
121.61
119.53
116.38
96.71
71.87
70.95
70.63
67.95
61.56
20.45
20.37
20.30
20.29
20.27
f1 (ppm)
AcO
AcO
OAc
OAc
O
O
O
F
F

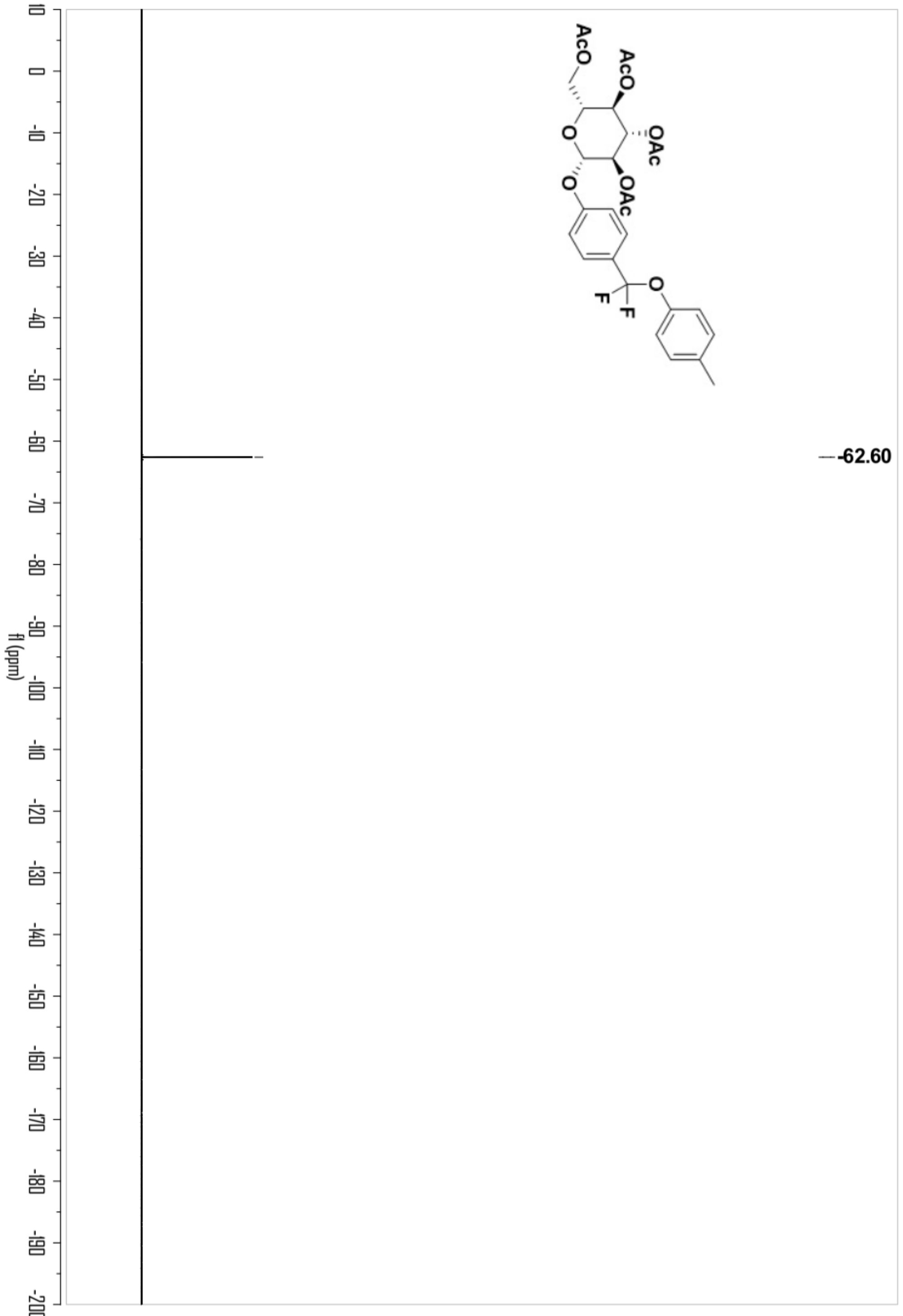
-62.60
f1 (ppm)
AcO
AcO
OAc
OAc
O
O
O
F
F

^{1}H, ^{13}C and ^{19}F NMR spectra of compound 3j

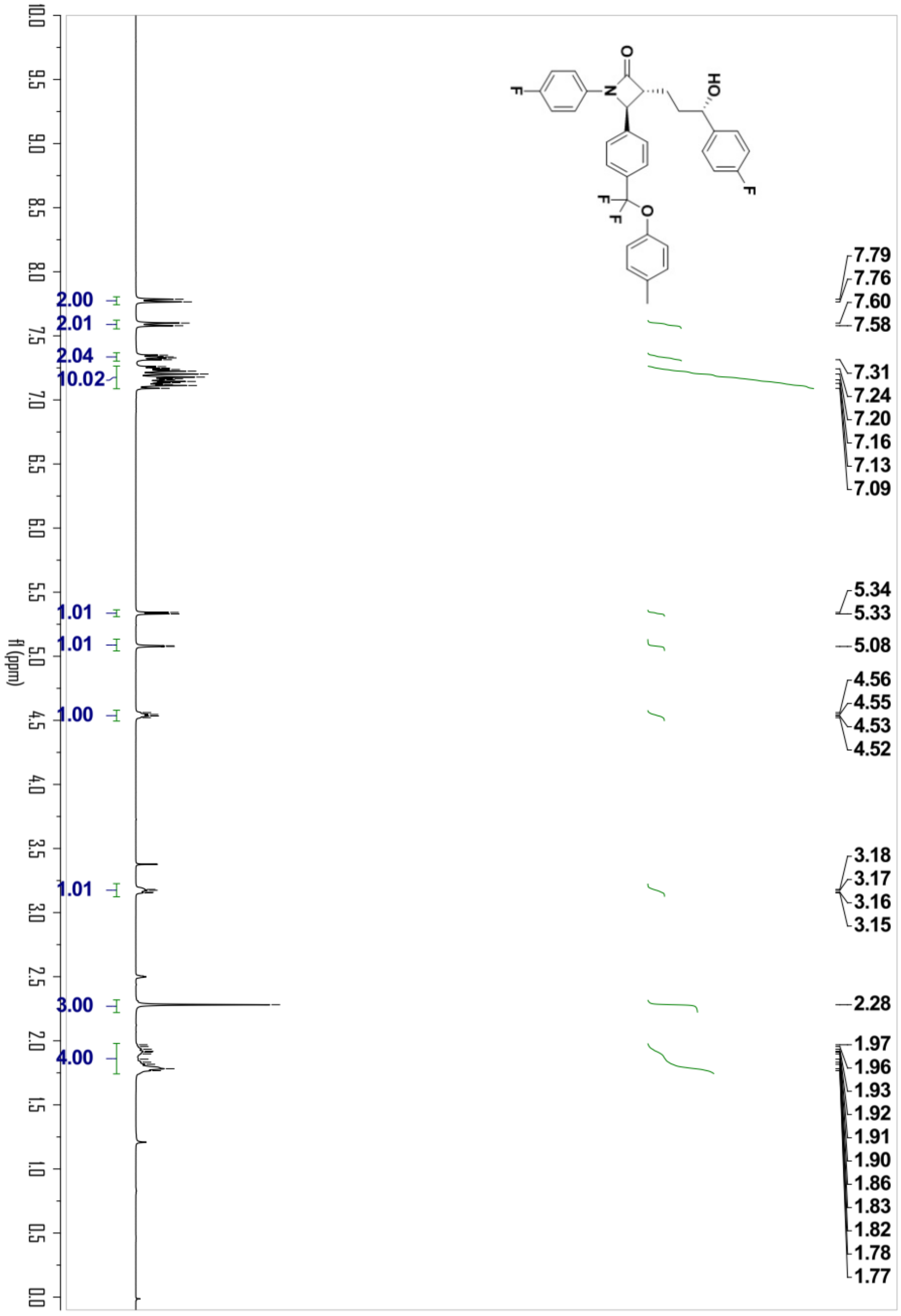

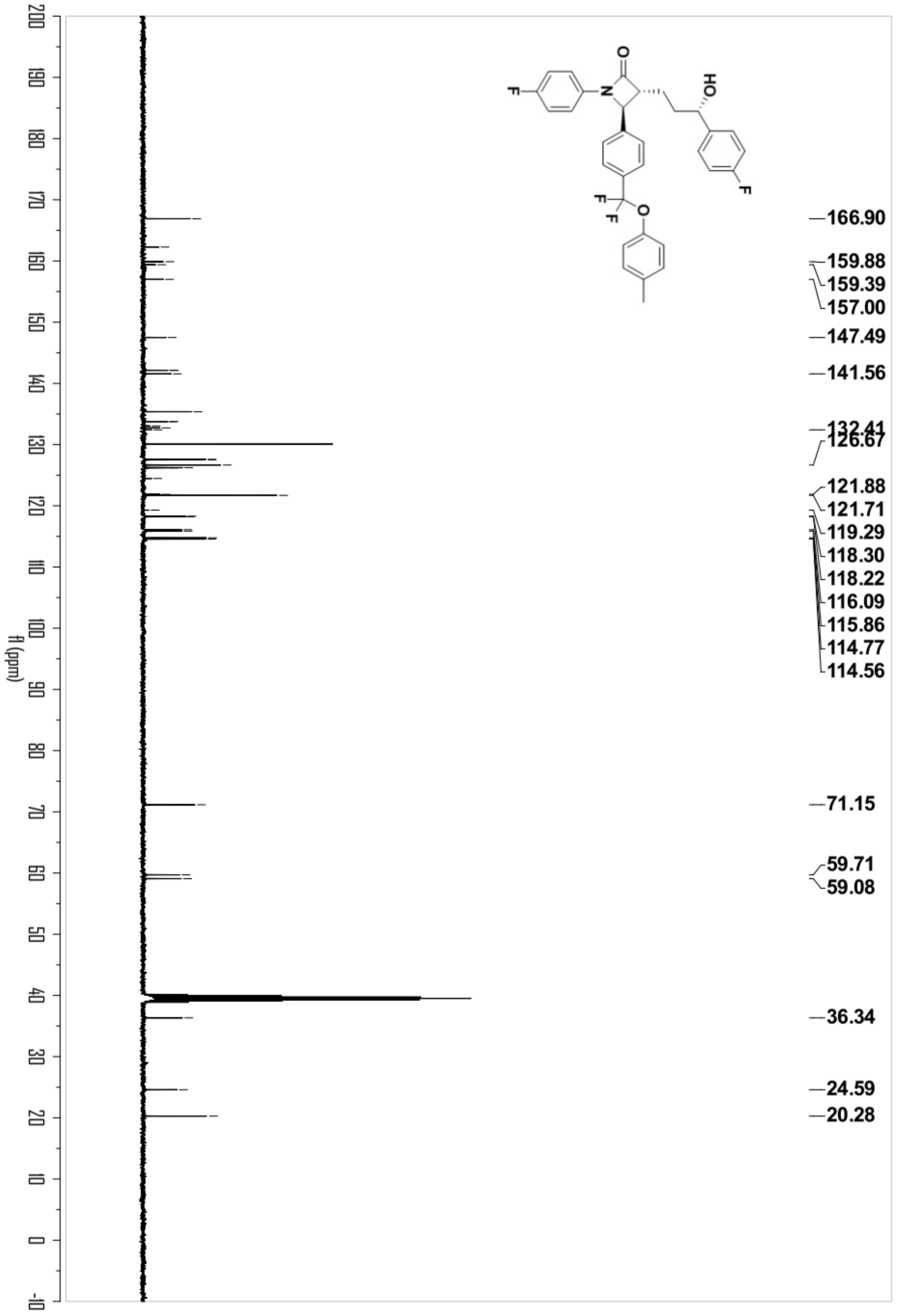
166.90
159.88
159.39
157.00
147.49
141.56
132.41
126.67
121.88
121.71
119.29
118.30
118.22
116.09
115.86
114.77
114.56
71.15
59.71
59.08
36.34
24.59
20.28
f1 (ppm)

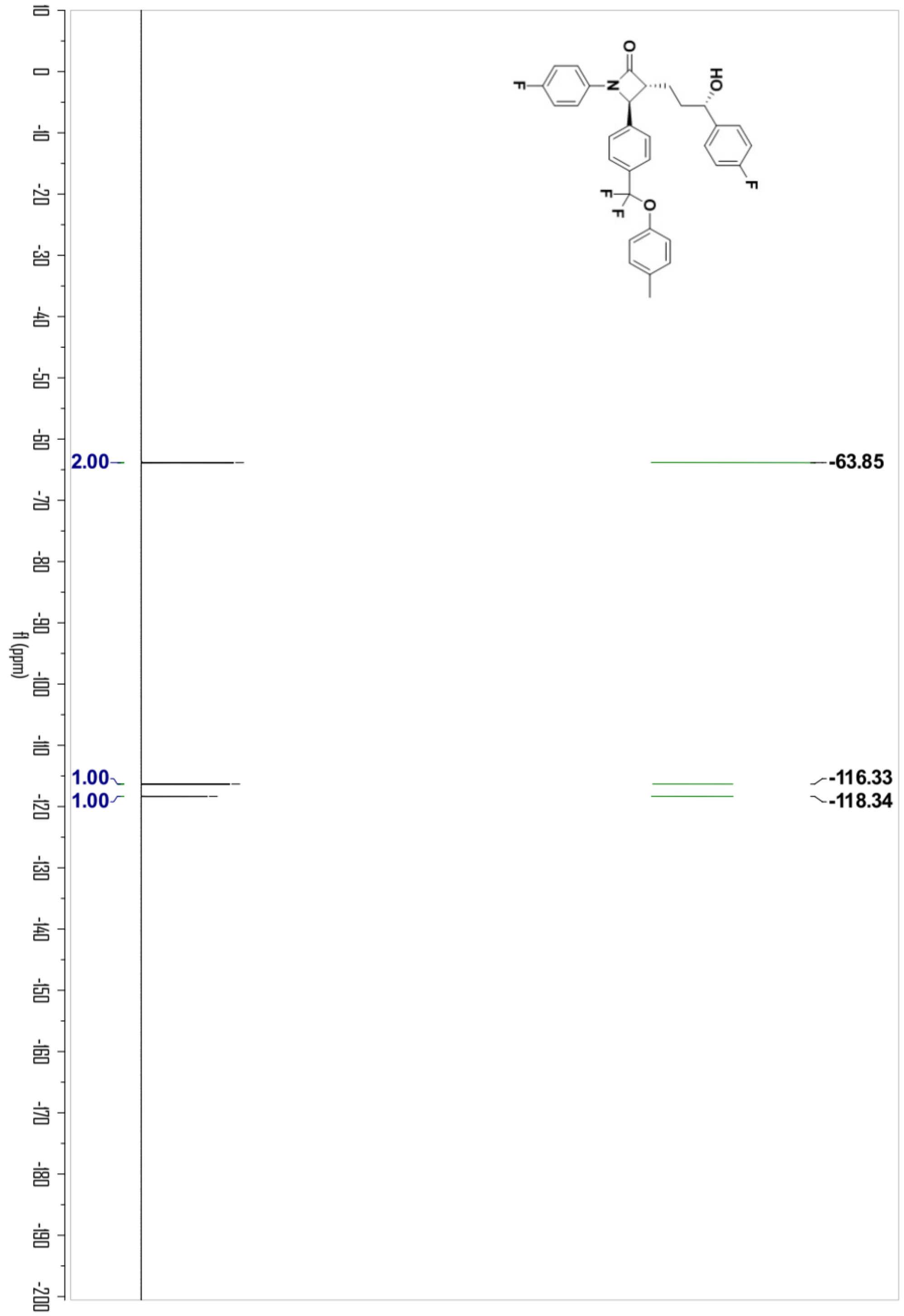
-63.85
-116.33
-118.34
2.00
1.00
1.00
f1 (ppm)

^{1}H, ^{13}C and ^{19}F NMR spectra of compound 7a

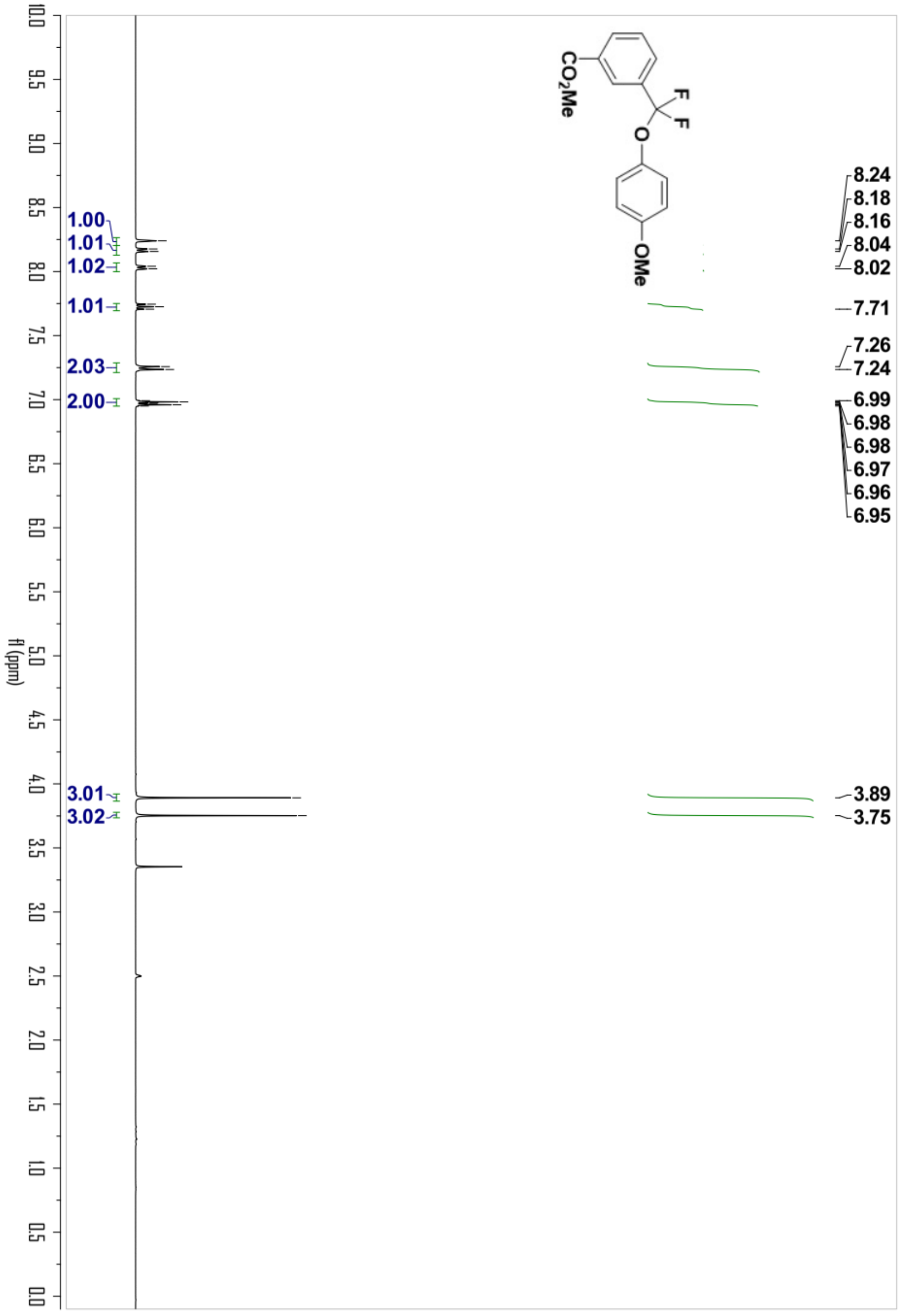

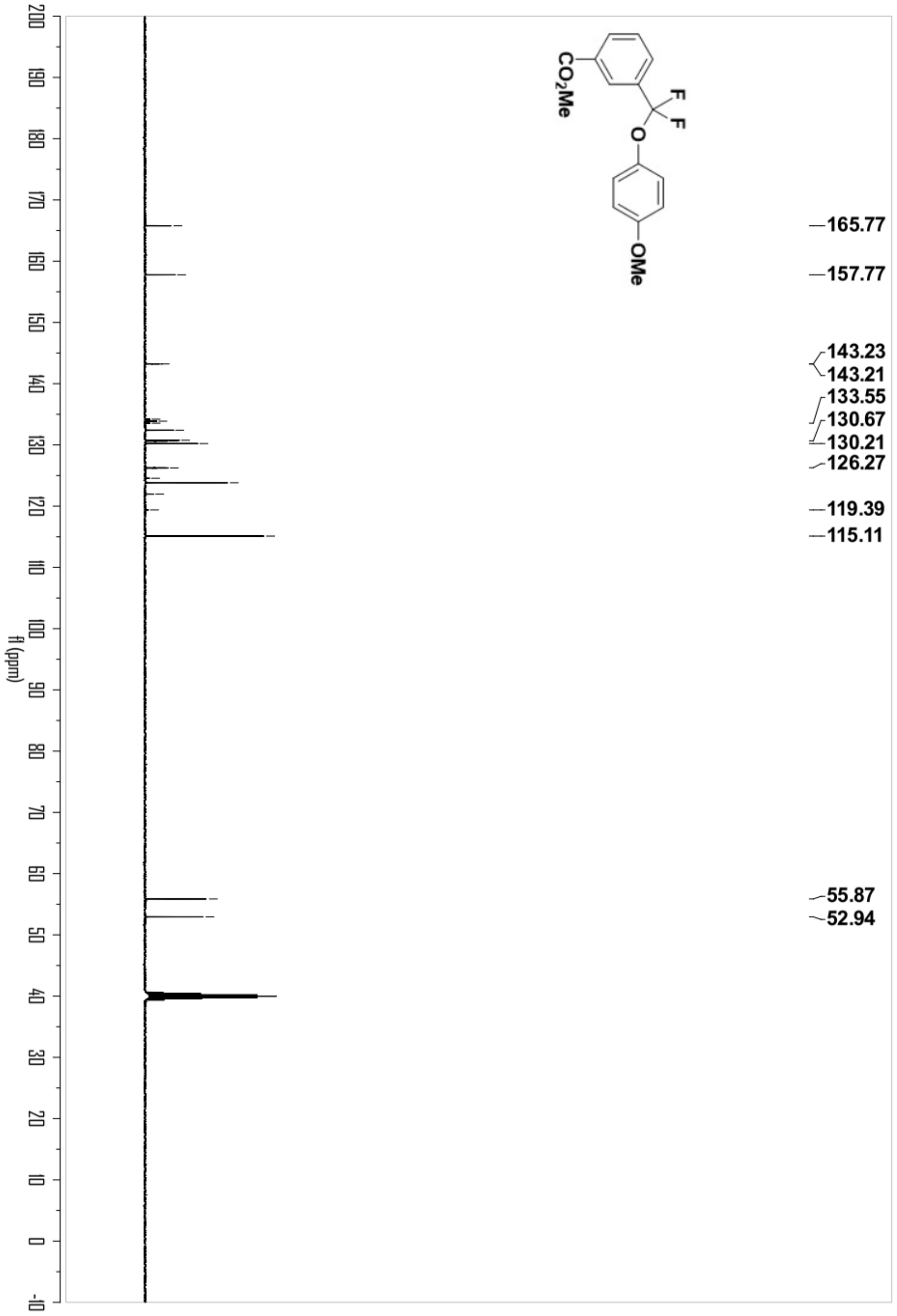
165.77
157.77
143.23
143.21
133.55
130.67
130.21
126.27
119.39
115.11
55.87
52.94
CO2Me
F F
O
OMe
f1 (ppm)

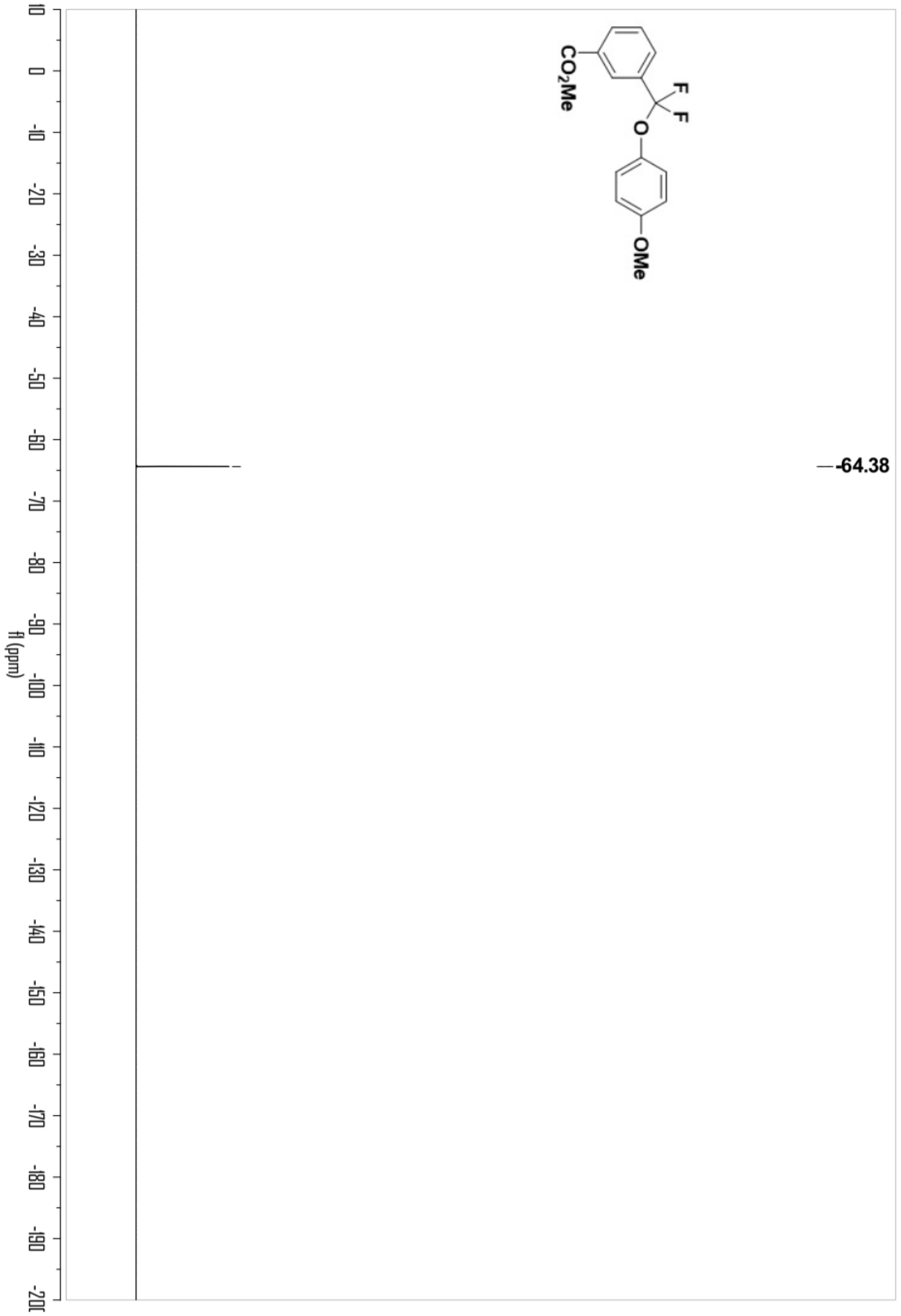
-64.38
f1 (ppm)
F
F
O
CO2Me
OMe

^{1}H, ^{13}C and ^{19}F NMR spectra of compound 7b

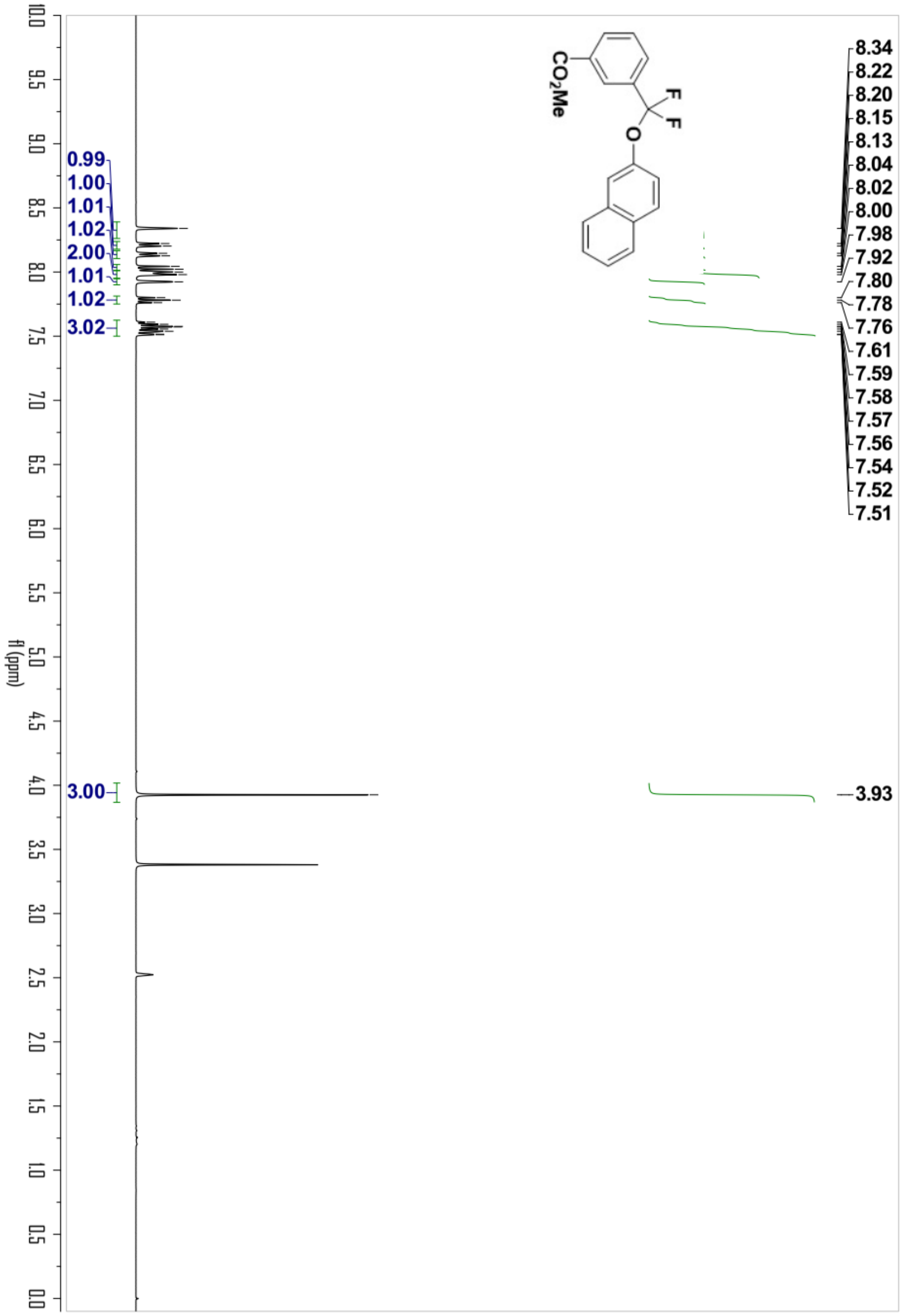

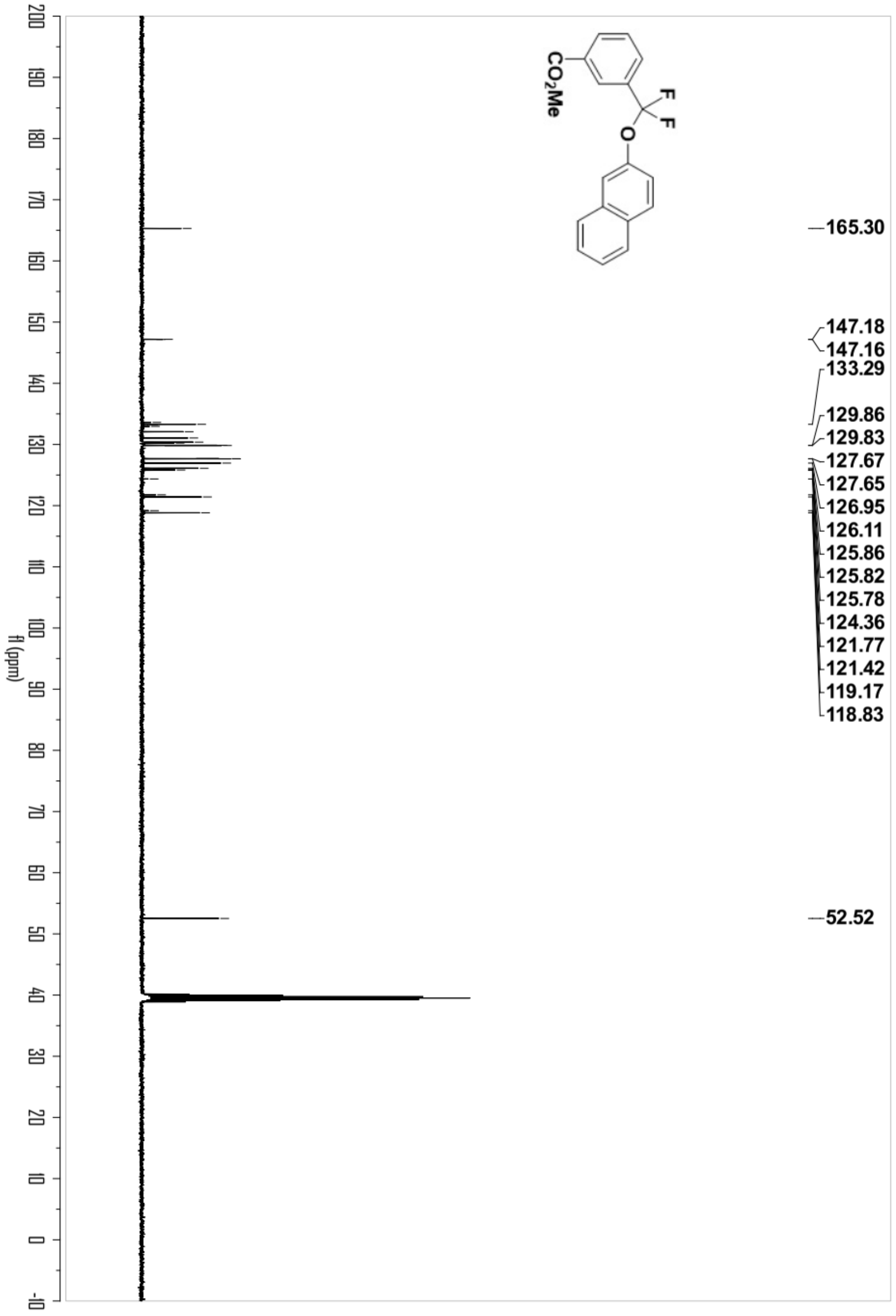

CO2Me
F F
O
165.30
147.18
147.16
133.29
129.86
129.83
127.67
127.65
126.95
126.11
125.86
125.82
125.78
124.36
121.77
121.42
119.17
118.83
52.52
f1 (ppm)

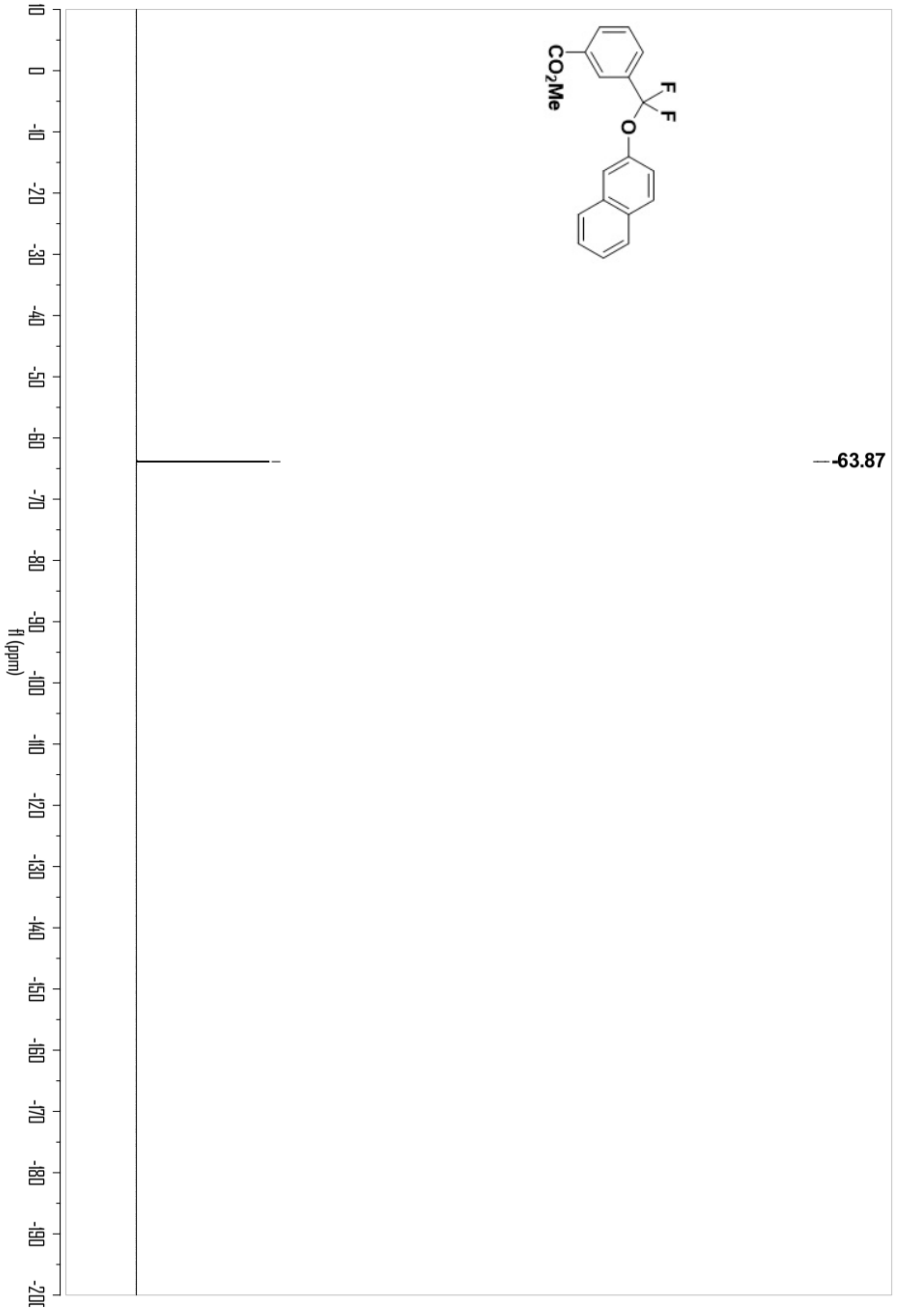
-63.87
f1 (ppm)
CO2Me
F
F
O

^{1}H, ^{13}C and ^{19}F NMR spectra of compound 7c

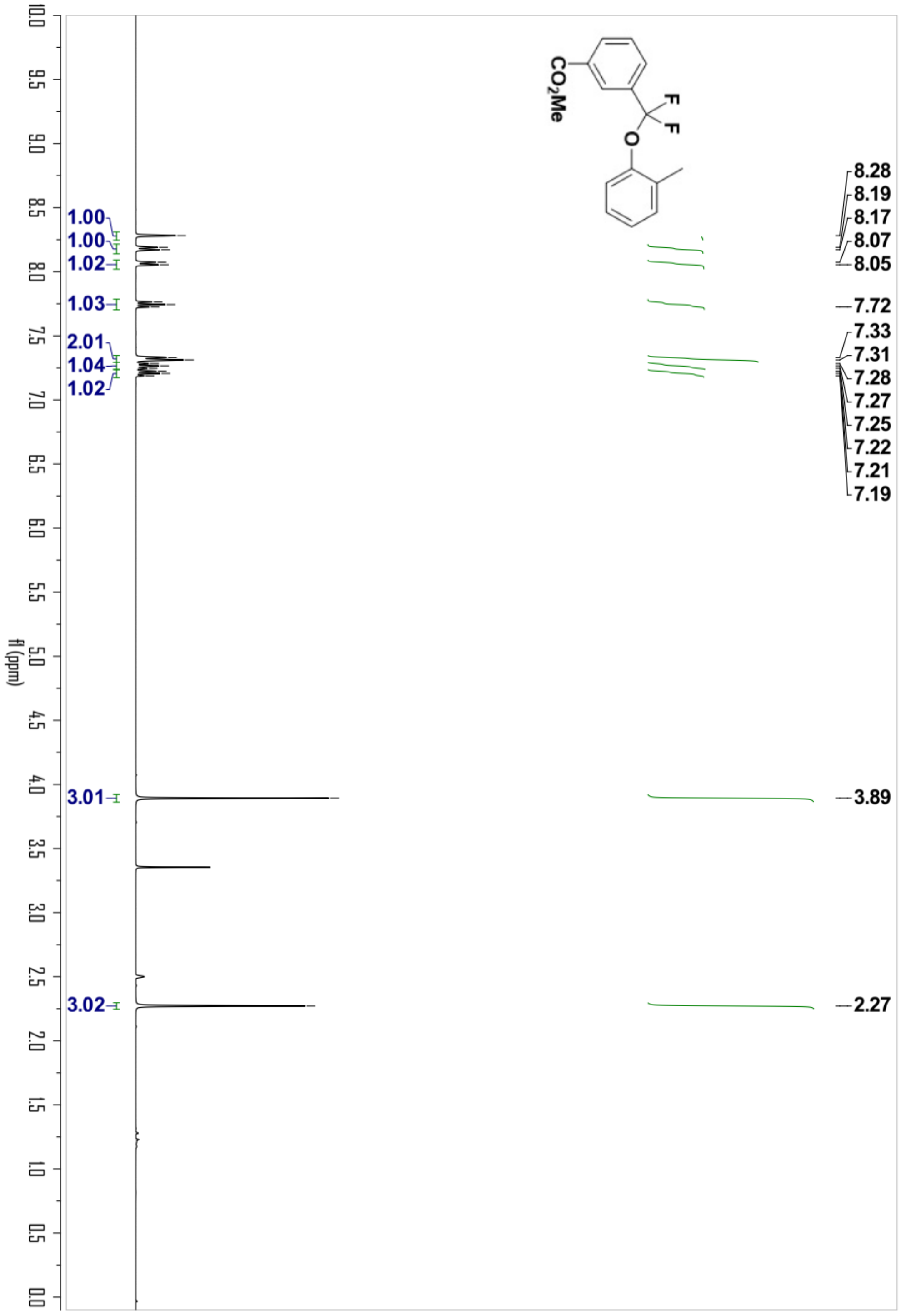

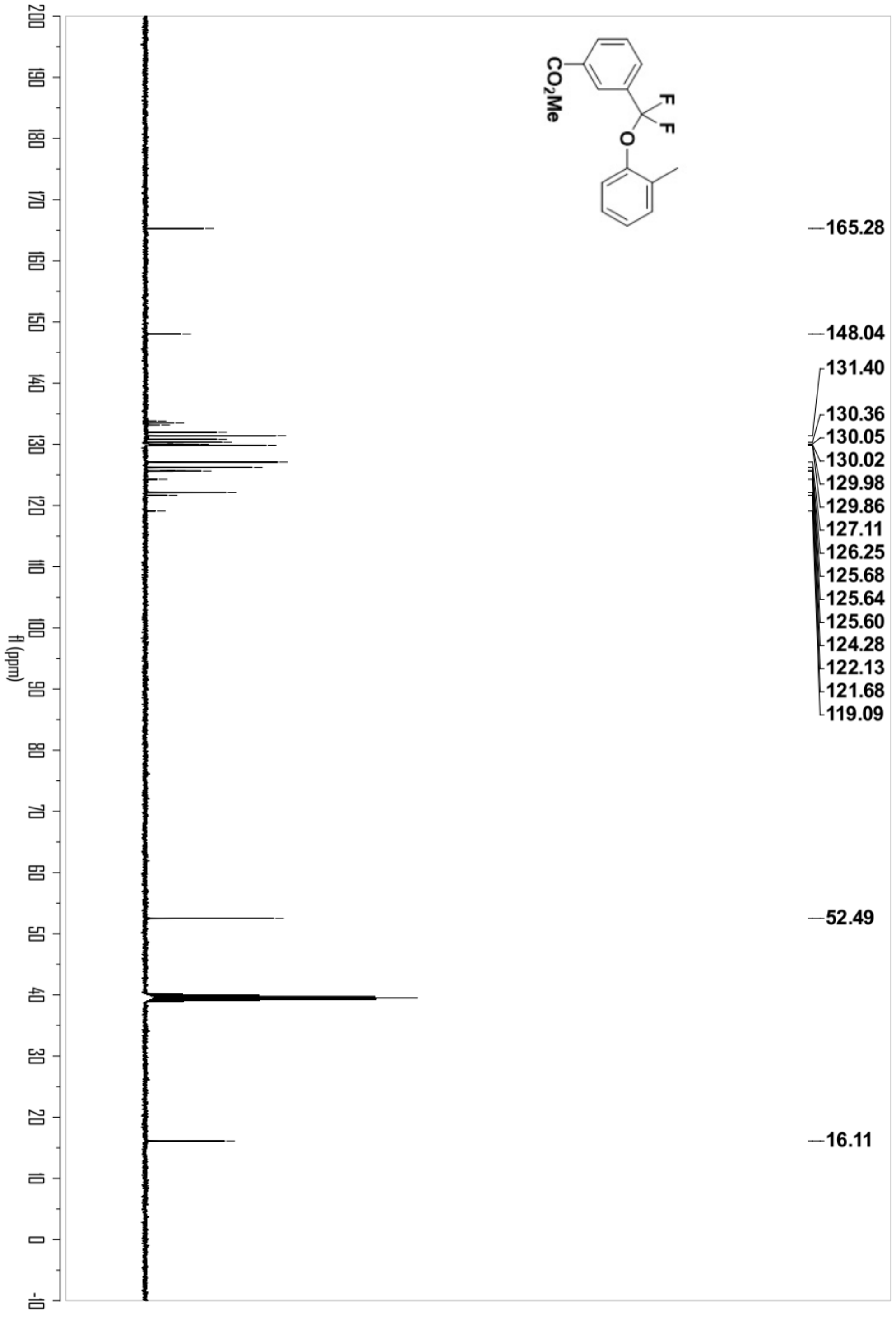
CO2Me
F F
O
165.28
148.04
131.40
130.36
130.05
130.02
129.98
129.86
127.11
126.25
125.68
125.64
125.60
124.28
122.13
121.68
119.09
52.49
16.11
f1 (ppm)
200
190
180
170
160
150
140
130
120
110
100
90
80
70
60
50
40
30
20
10
0
-10

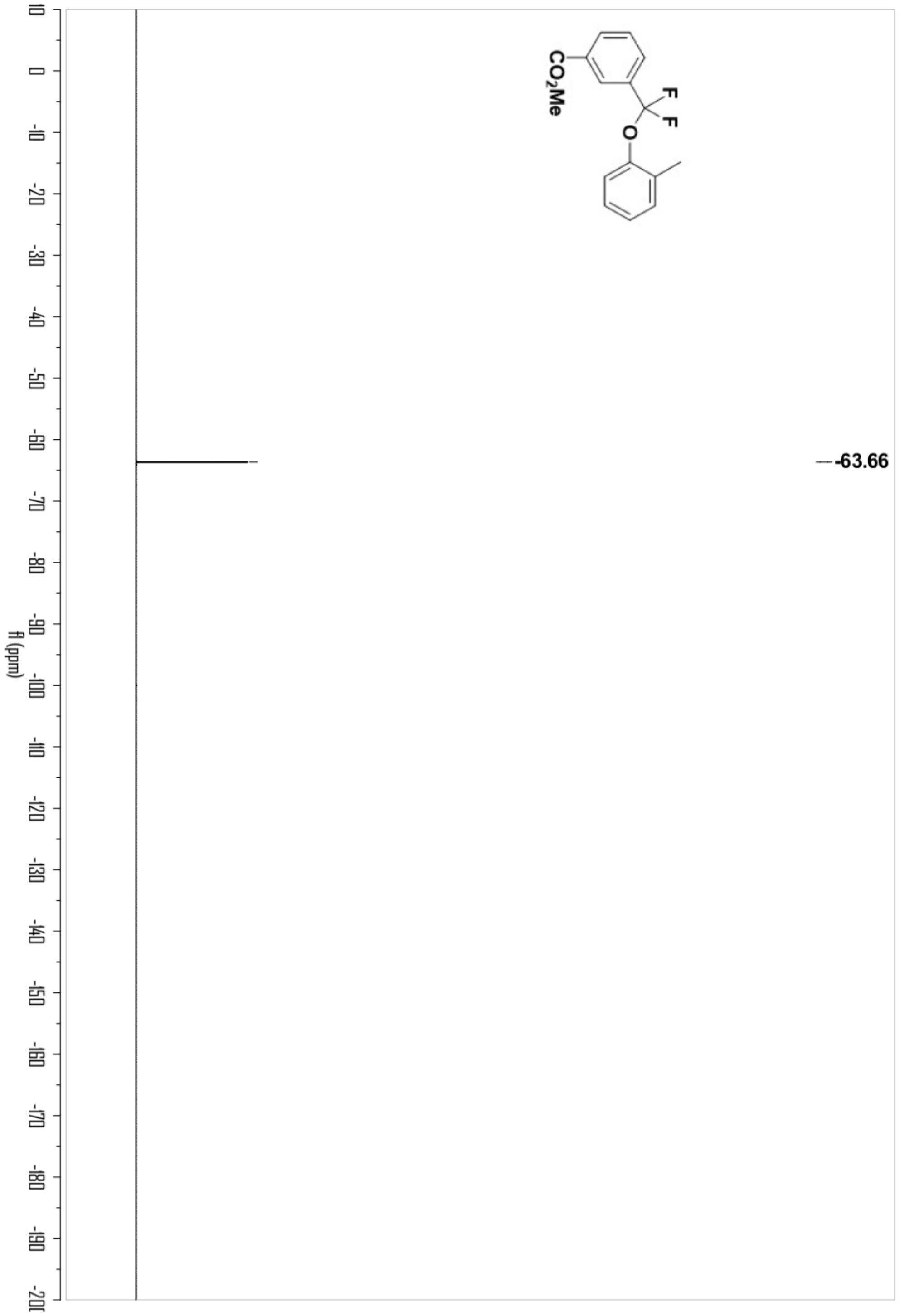
CO2Me
F
F
O
-63.66
f1 (ppm)

^{1}H, ^{13}C and ^{19}F NMR spectra of compound 7d

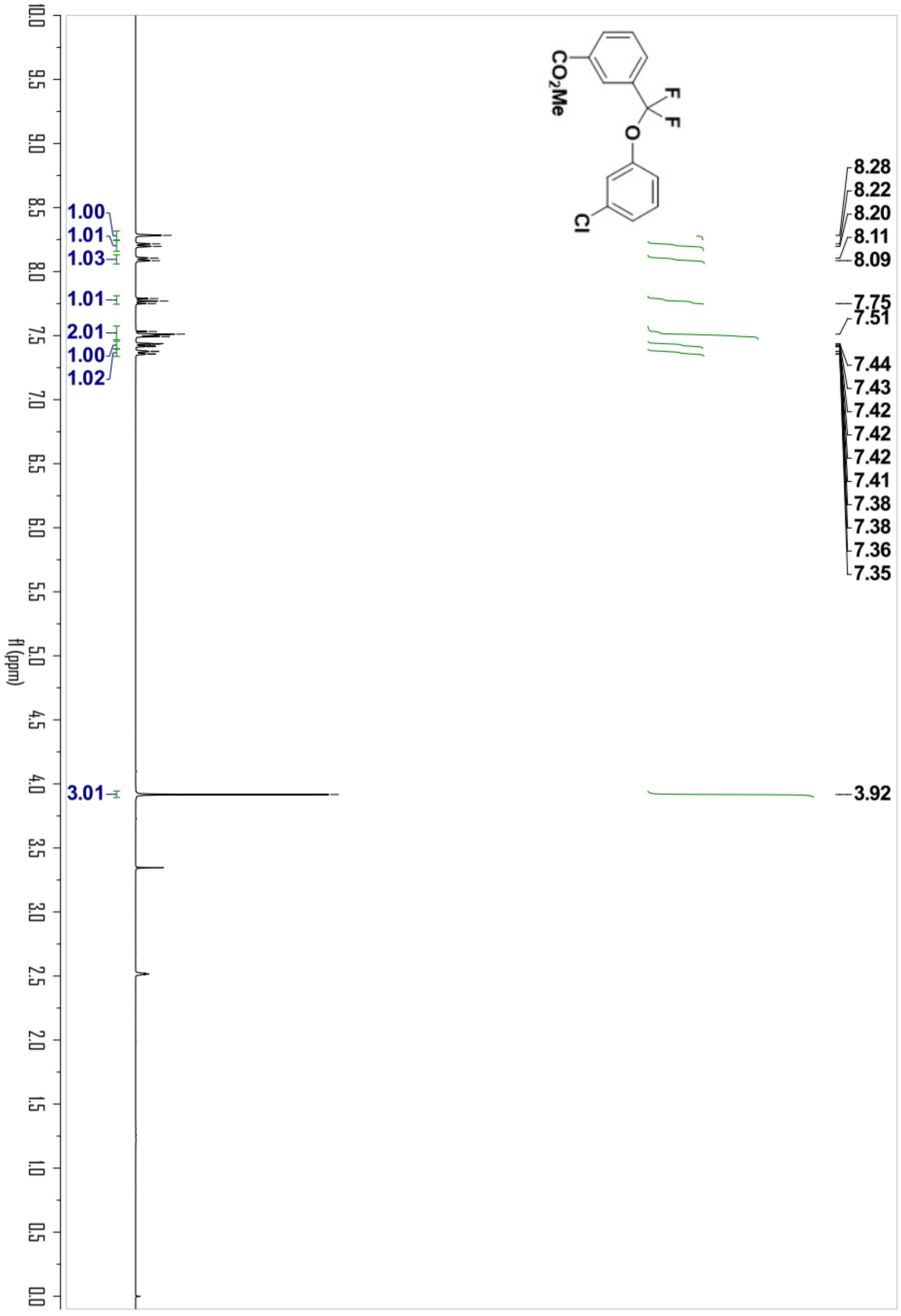

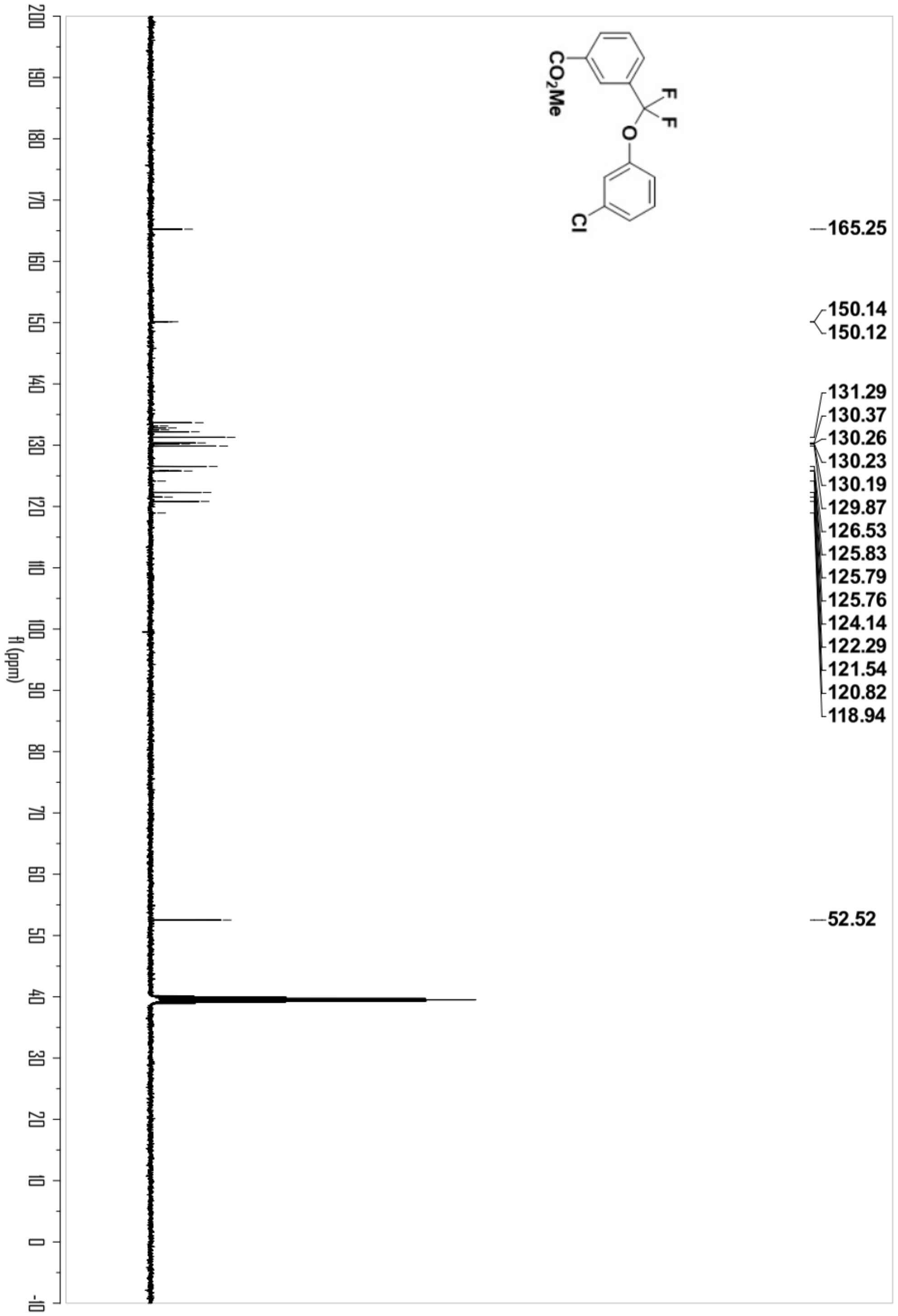
CO2Me
F
F
O
Cl
165.25
150.14
150.12
131.29
130.37
130.26
130.23
130.19
129.87
126.53
125.83
125.79
125.76
124.14
122.29
121.54
120.82
118.94
52.52
f1 (ppm)

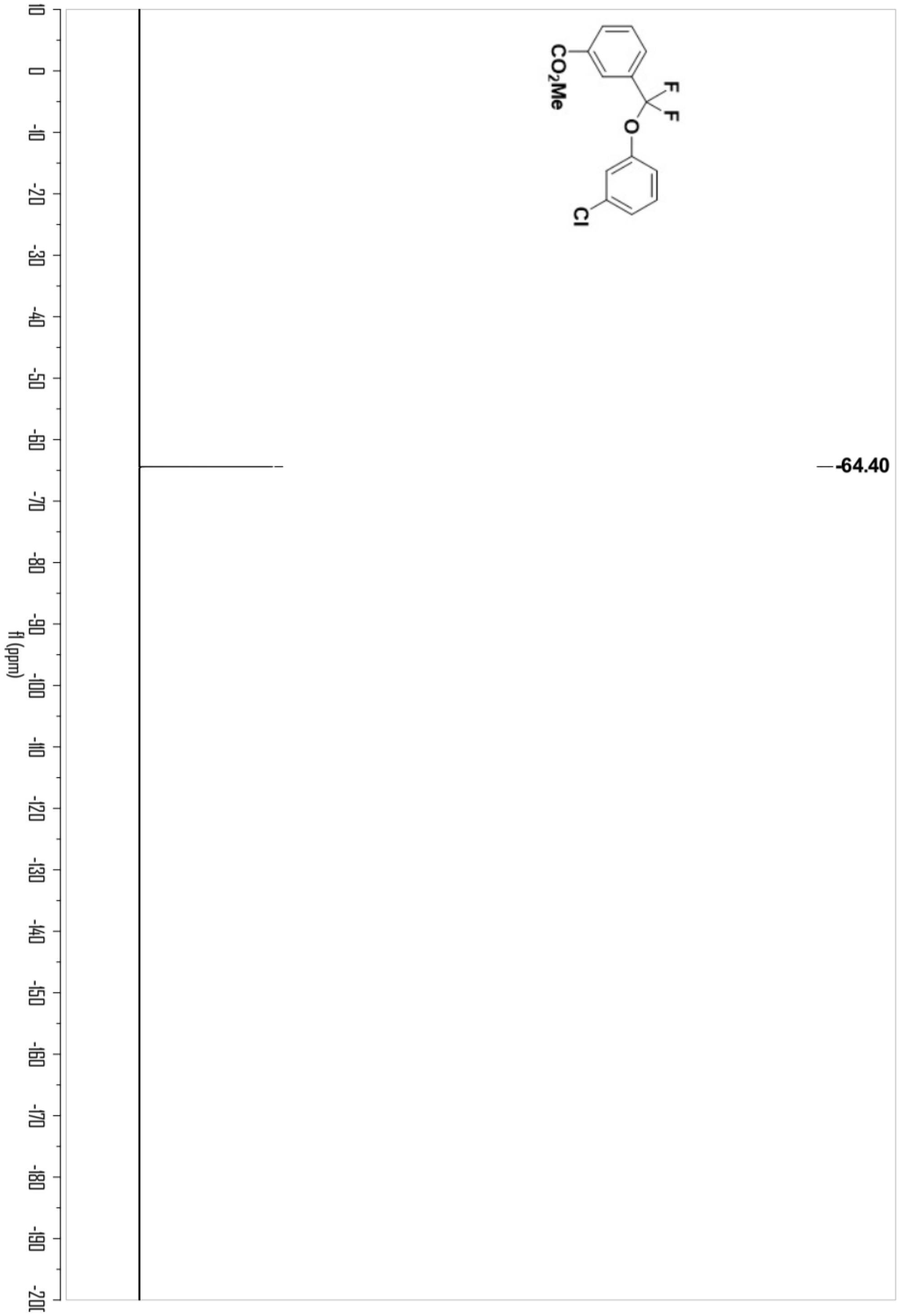

-64.40
CO2Me
F
F
O
Cl
f1 (ppm)
10
0
-10
-20
-30
-40
-50
-60
-70
-80
-90
-100
-110
-120
-130
-140
-150
-160
-170
-180
-190
-200

^{1}H, ^{13}C and ^{19}F NMR spectra of compound 7e

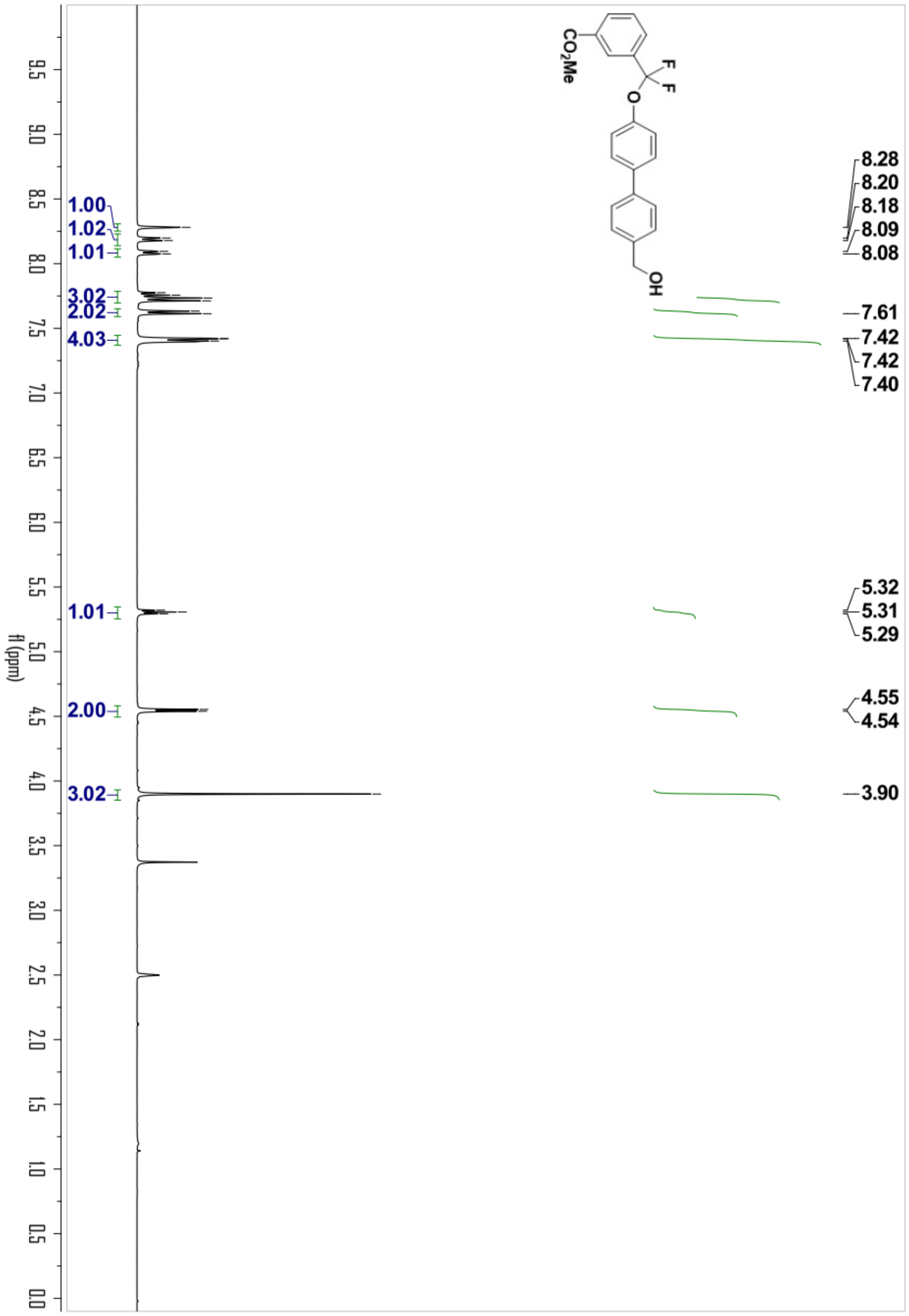

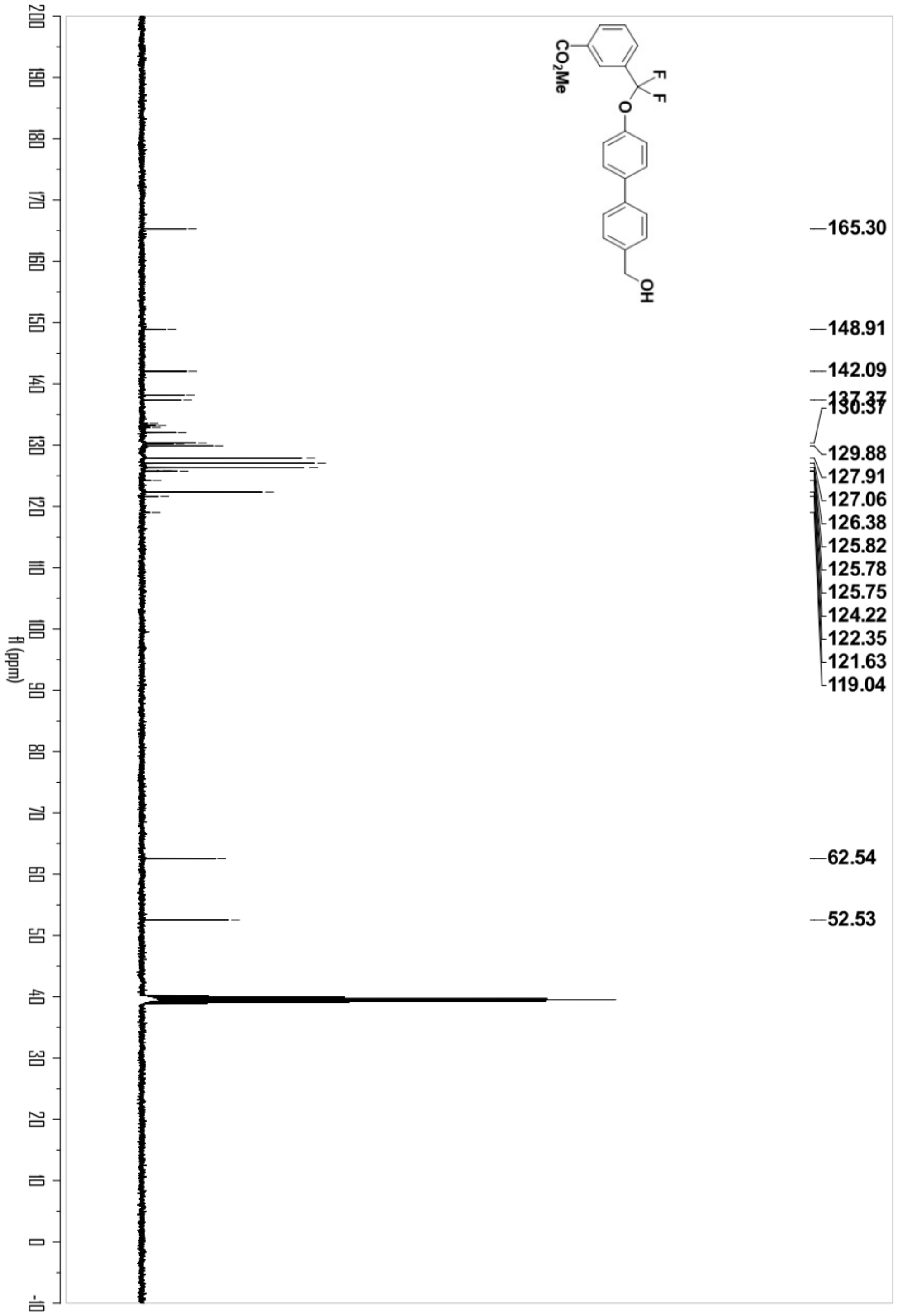
165.30
148.91
142.09
137.37
130.37
129.88
127.91
127.06
126.38
125.82
125.78
125.75
124.22
122.35
121.63
119.04
62.54
52.53
f1 (ppm)
CO2Me
F F
O
OH

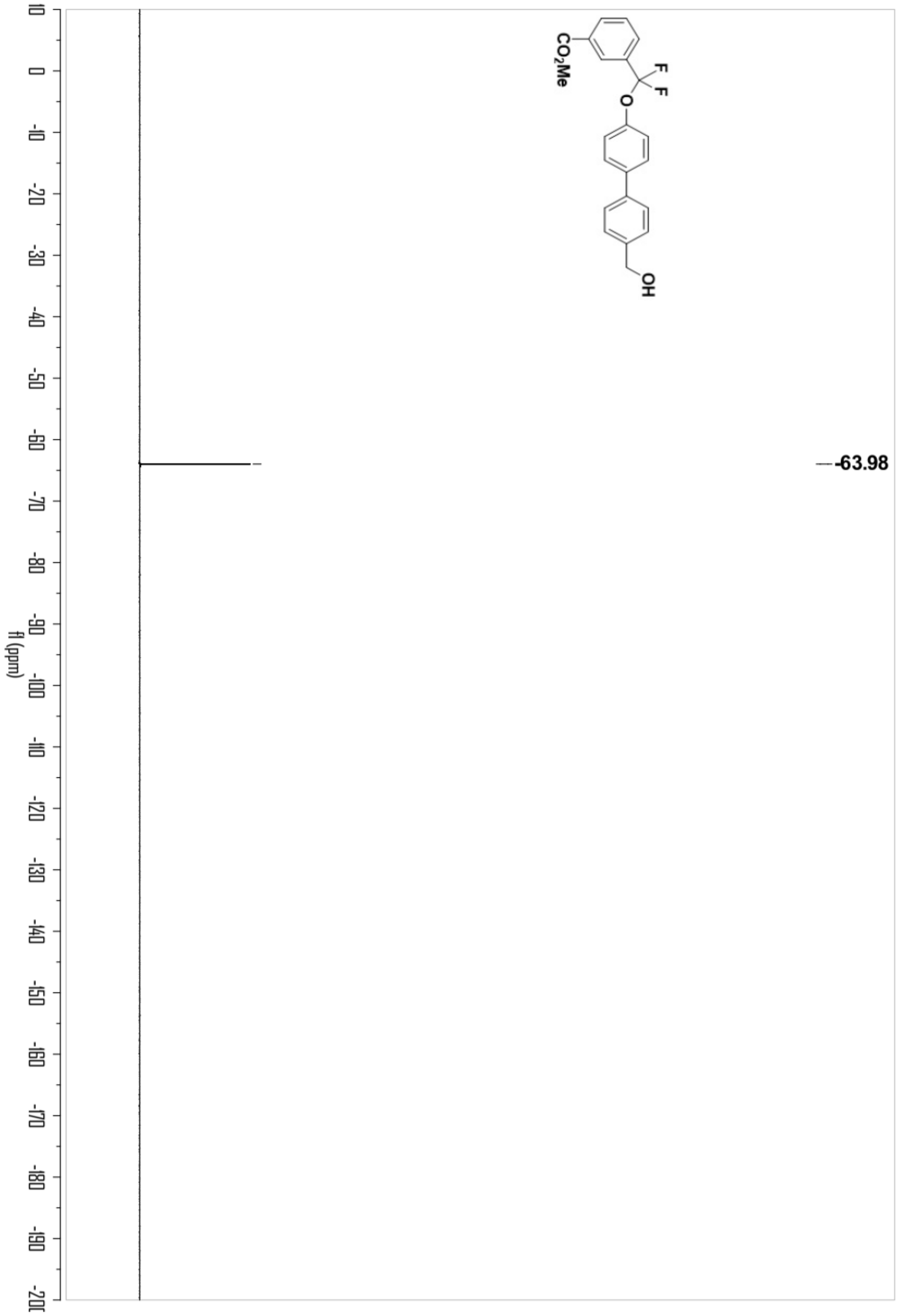
F F
O
CO2Me
OH
-63.98
f1 (ppm)

^1H, ^{13}C and ^{19}F NMR spectra of compound 7f

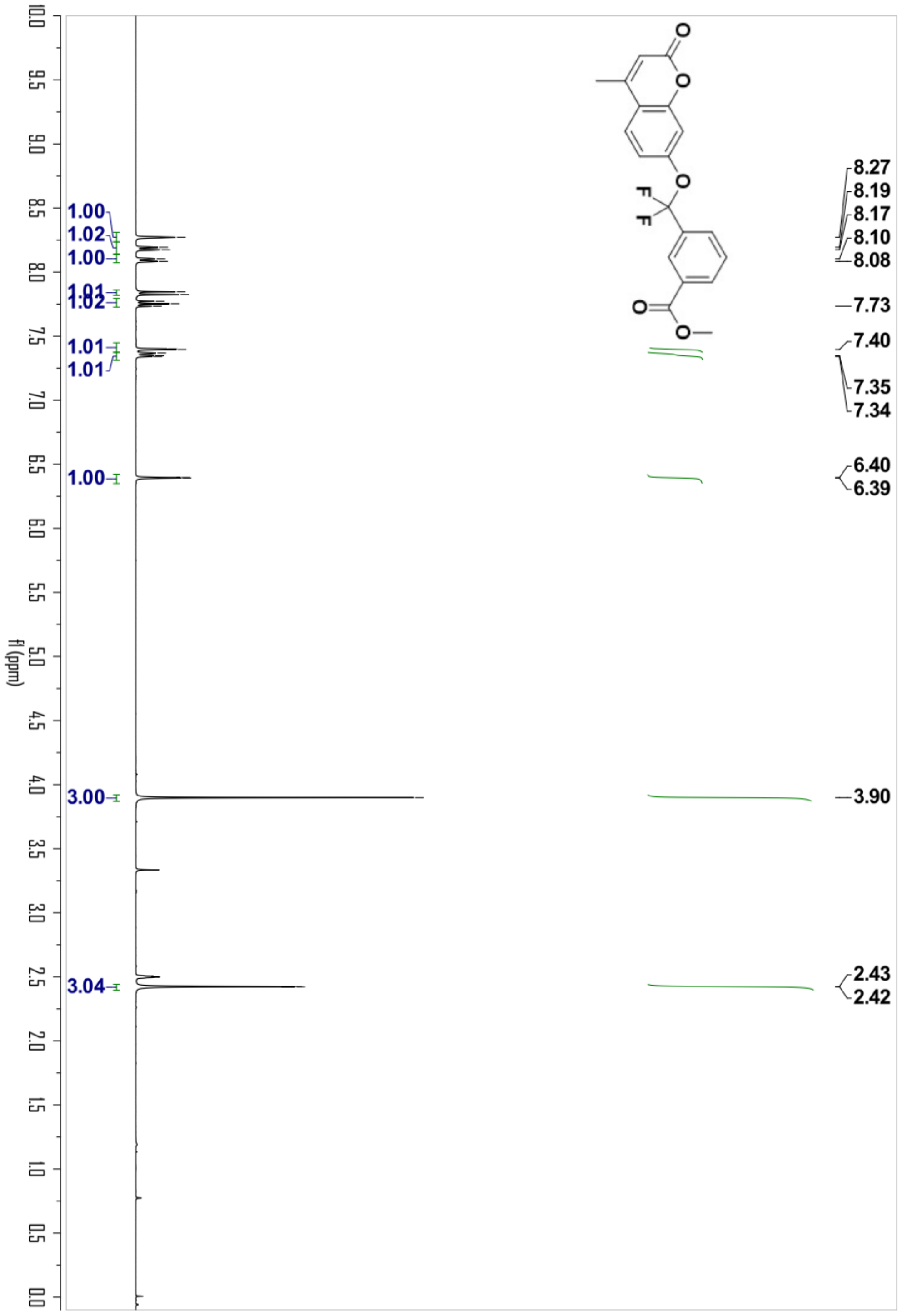

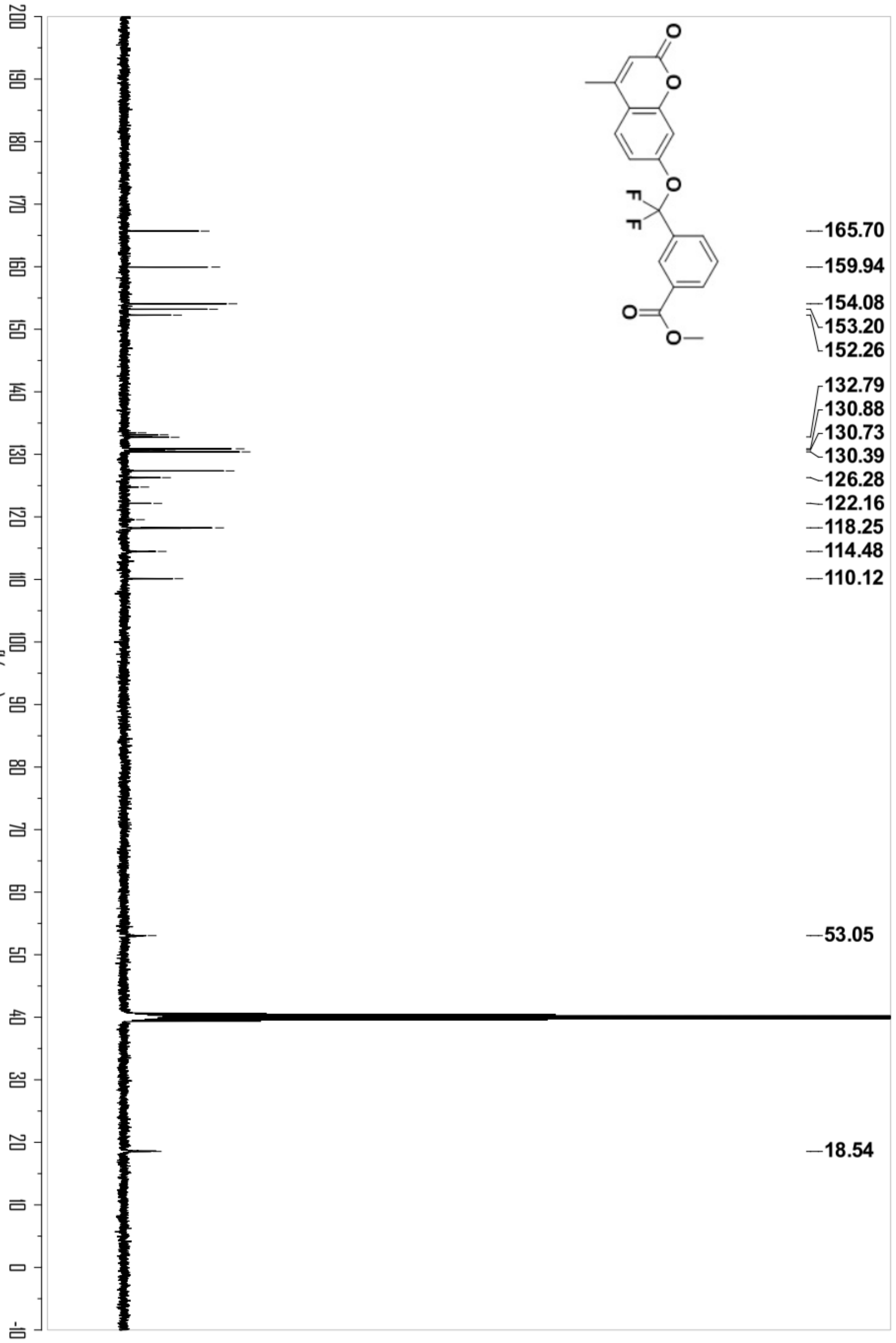

F
F
O
O
O
O
O
—165.70
—159.94
—154.08
153.20
152.26
132.79
130.88
130.73
130.39
126.28
122.16
118.25
114.48
110.12
—53.05
—18.54
200
190
180
170
160
150
140
130
120
110
100
90
80
70
60
50
40
30
20
10
0
-10
f1 (ppm)

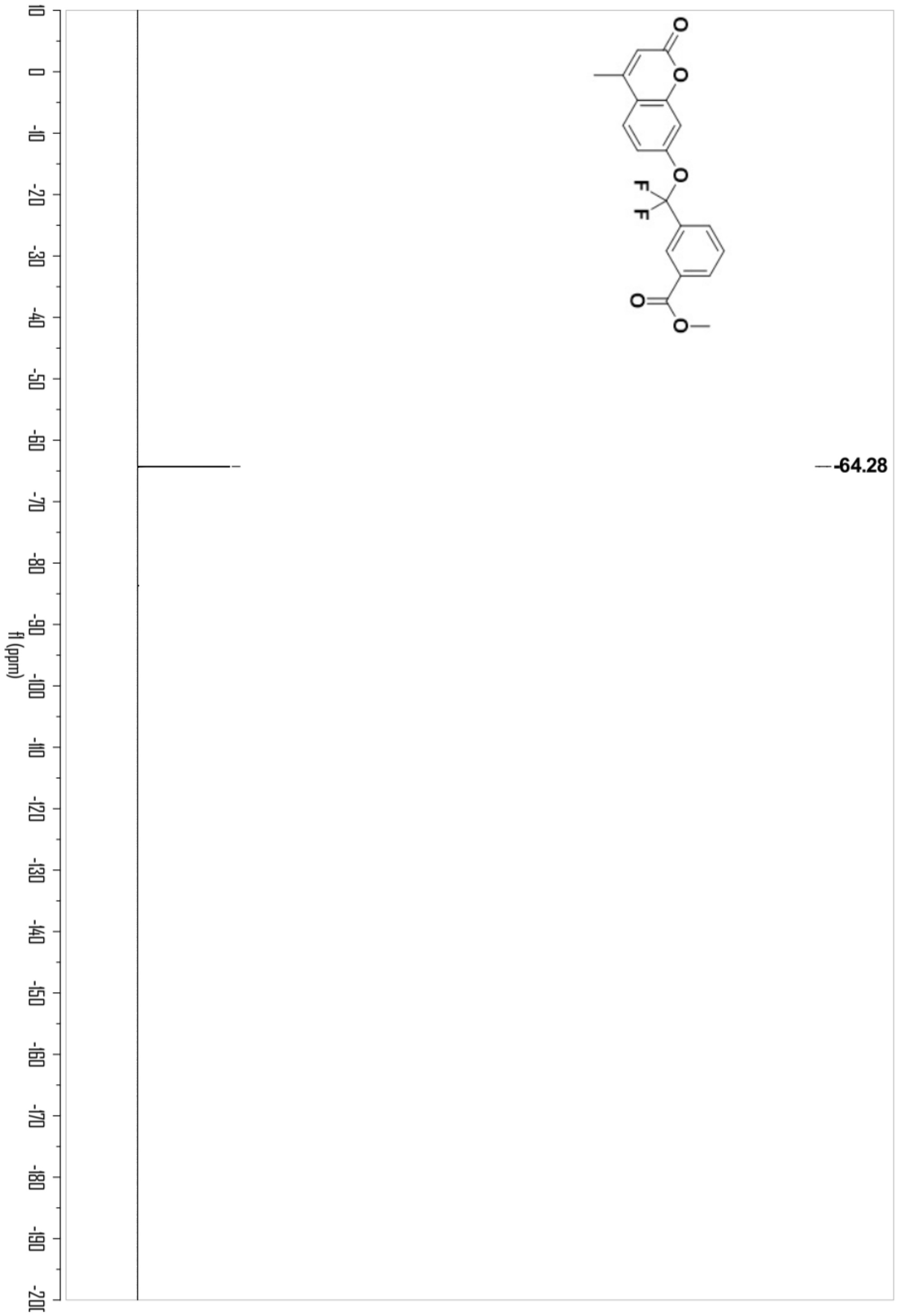
-64.28
f1 (ppm)

^{1}H, ^{13}C and ^{19}F NMR spectra of compound 7g

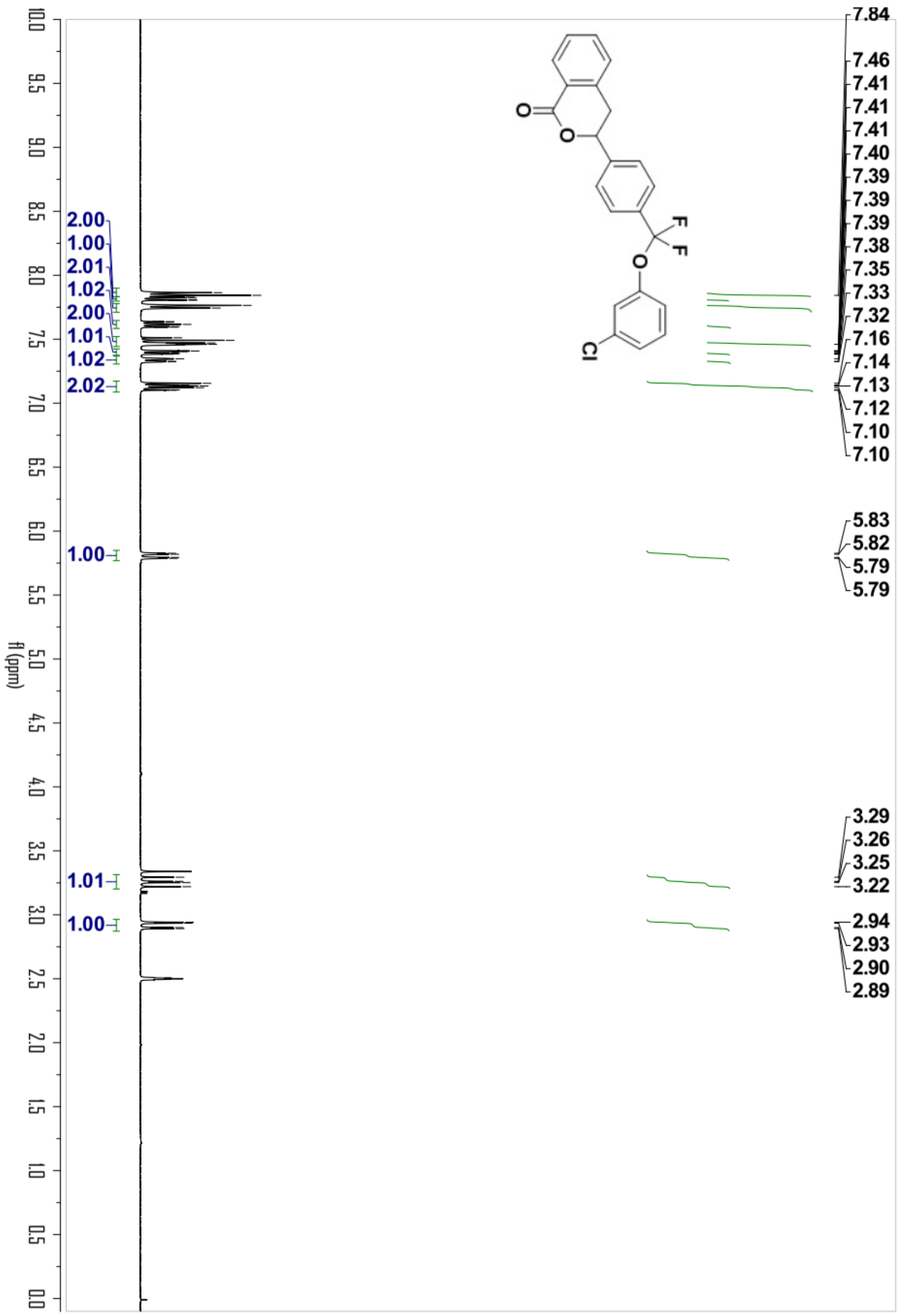

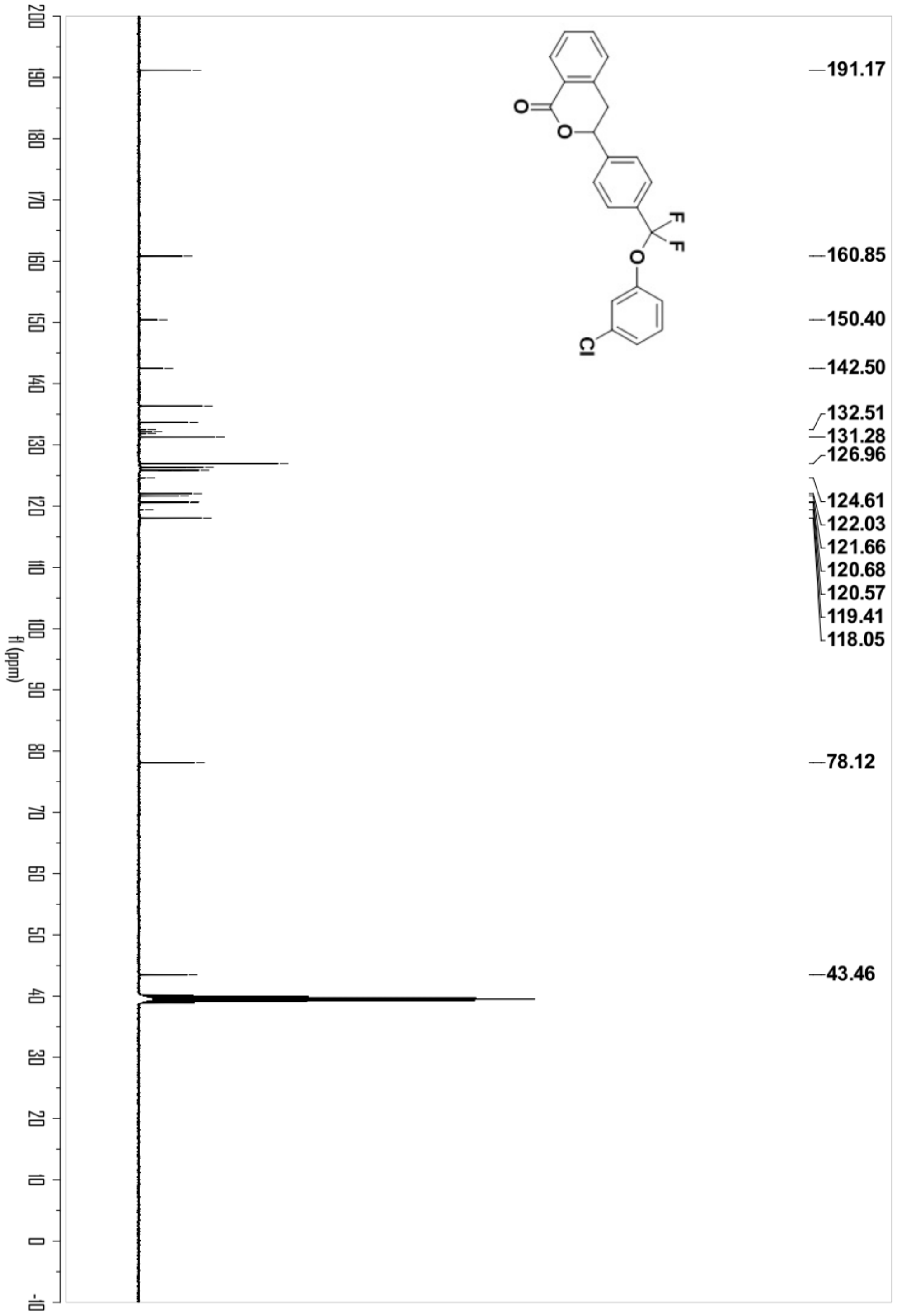
191.17
160.85
150.40
142.50
132.51
131.28
126.96
124.61
122.03
121.66
120.68
120.57
119.41
118.05
78.12
43.46
f1 (ppm)
O
O
F
F
O
Cl

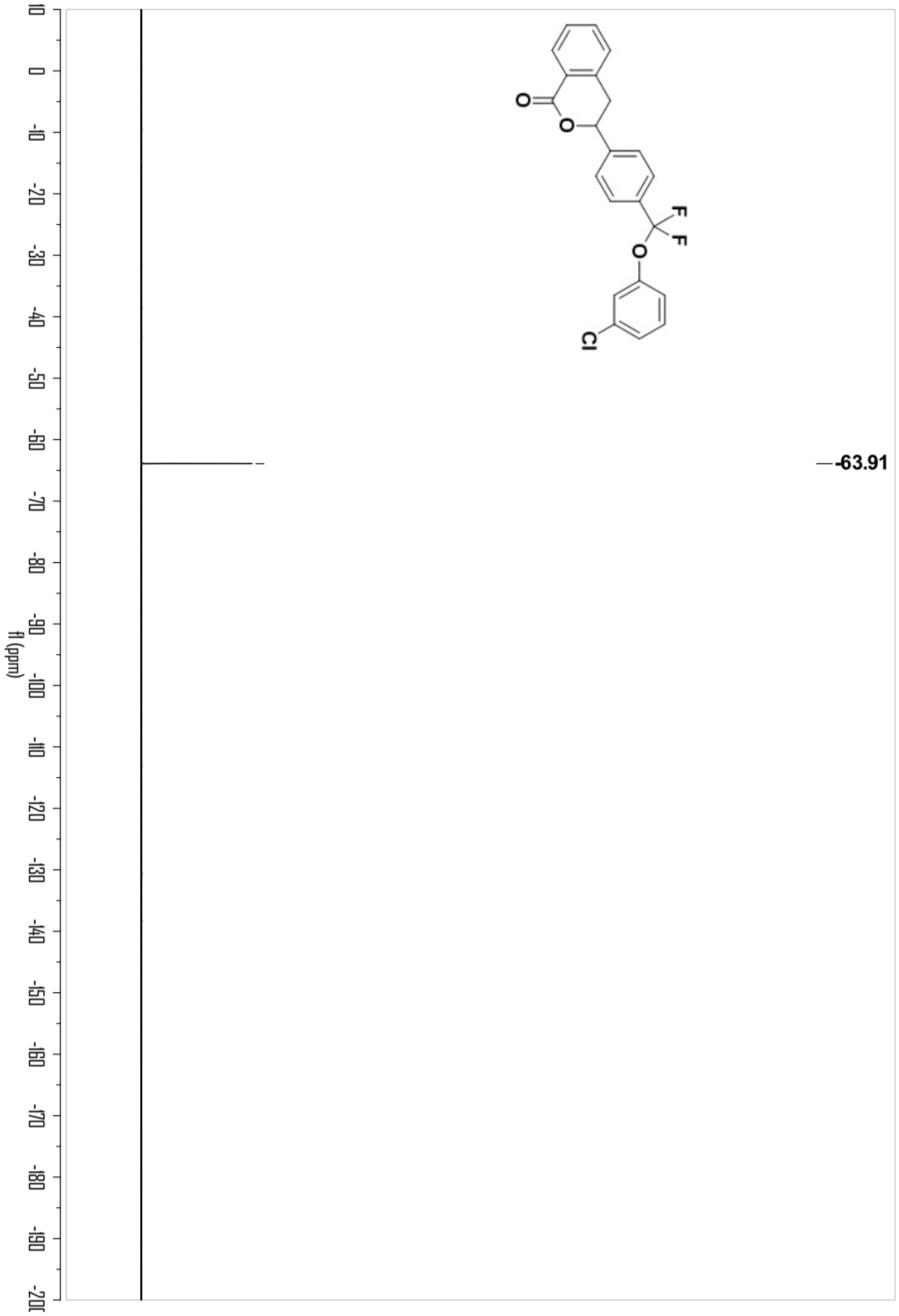
-63.91
f1 (ppm)
Cl

^{1}H, ^{13}C and ^{19}F NMR spectra of compound 7h

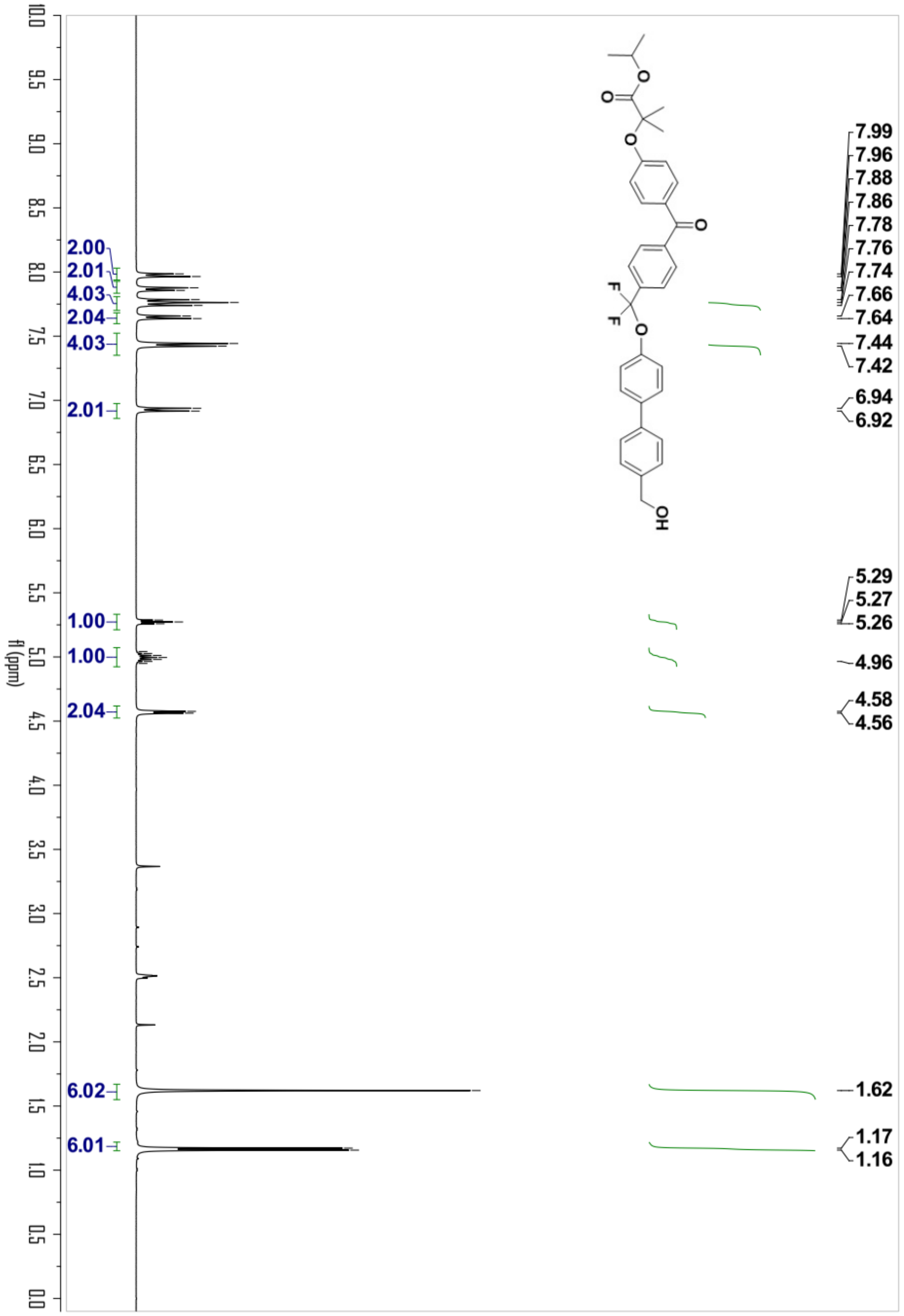

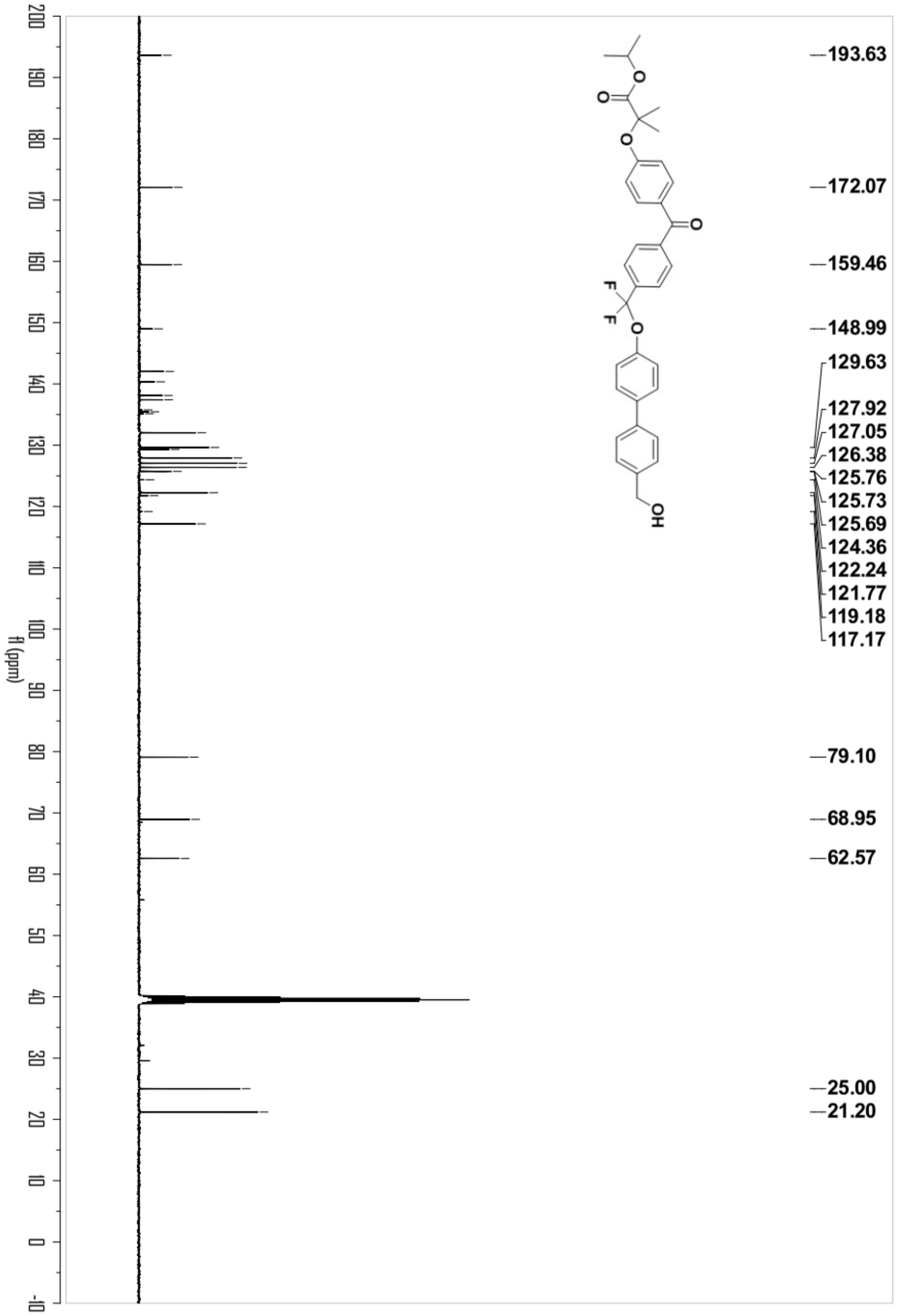

193.63
172.07
159.46
148.99
129.63
127.92
127.05
126.38
125.76
125.73
125.69
124.36
122.24
121.77
119.18
117.17
79.10
68.95
62.57
25.00
21.20
f1 (ppm)

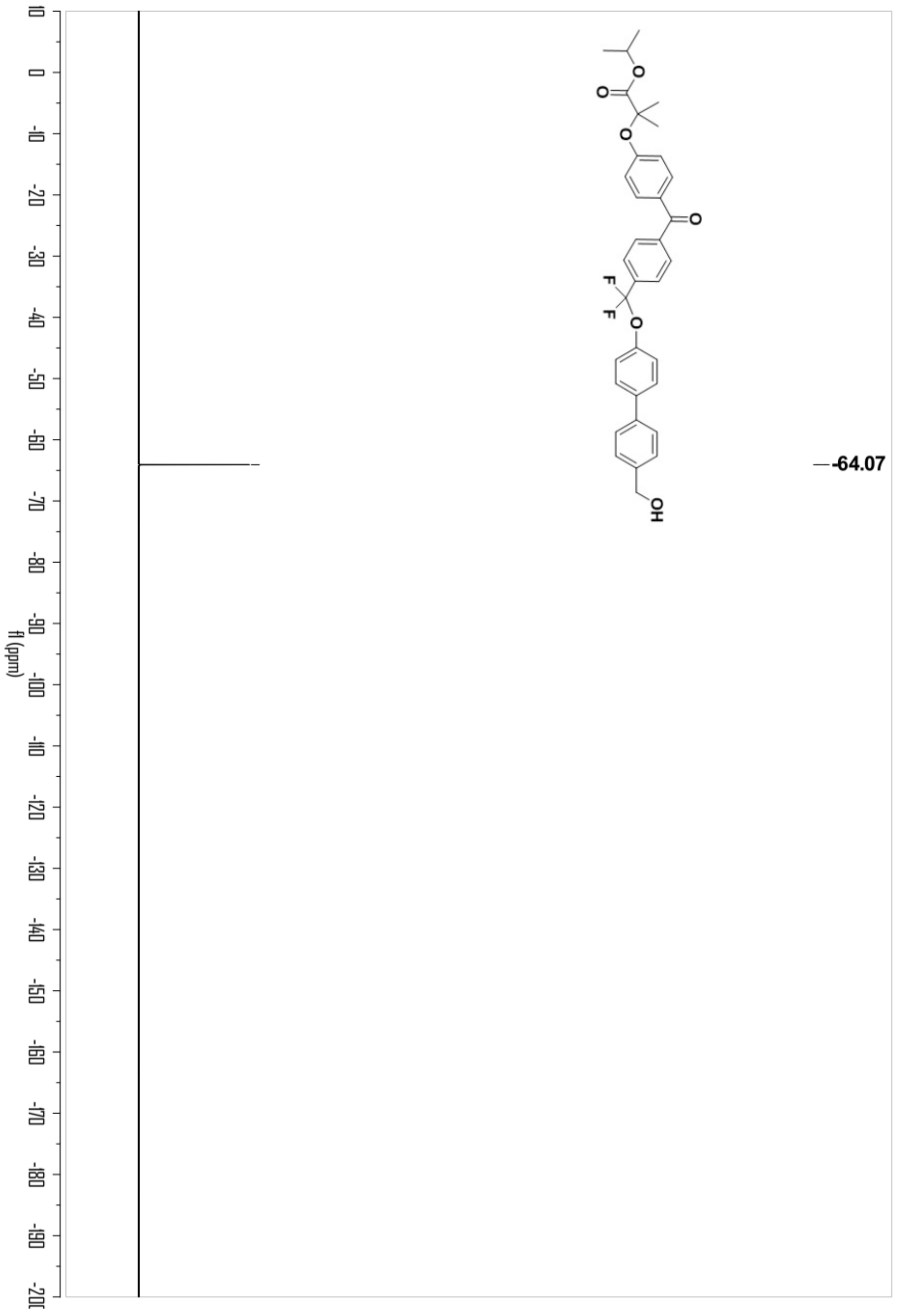
-64.07
f1 (ppm)
F
F
O
OH

^{1}H, ^{13}C and ^{19}F NMR spectra of compound 7i

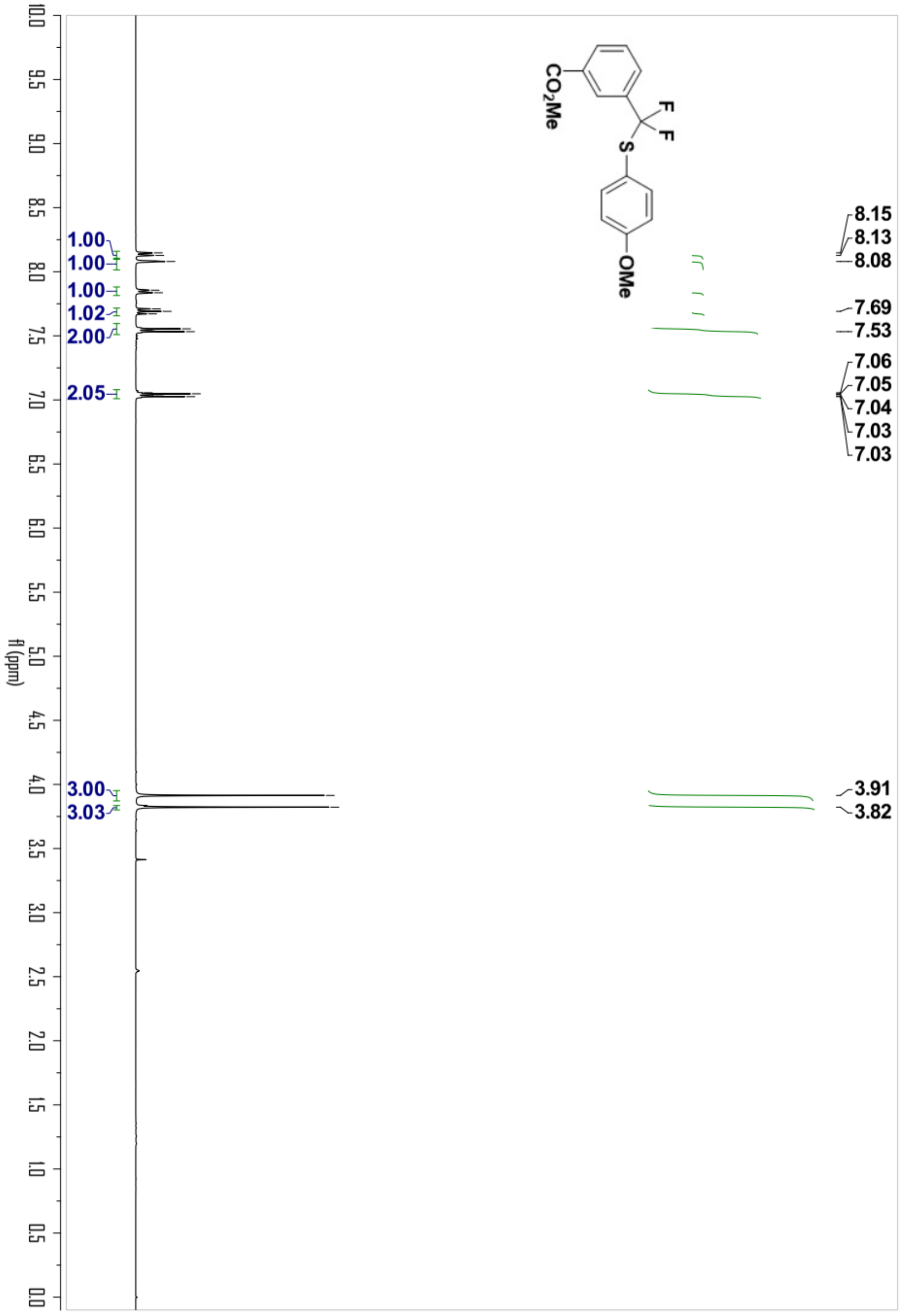

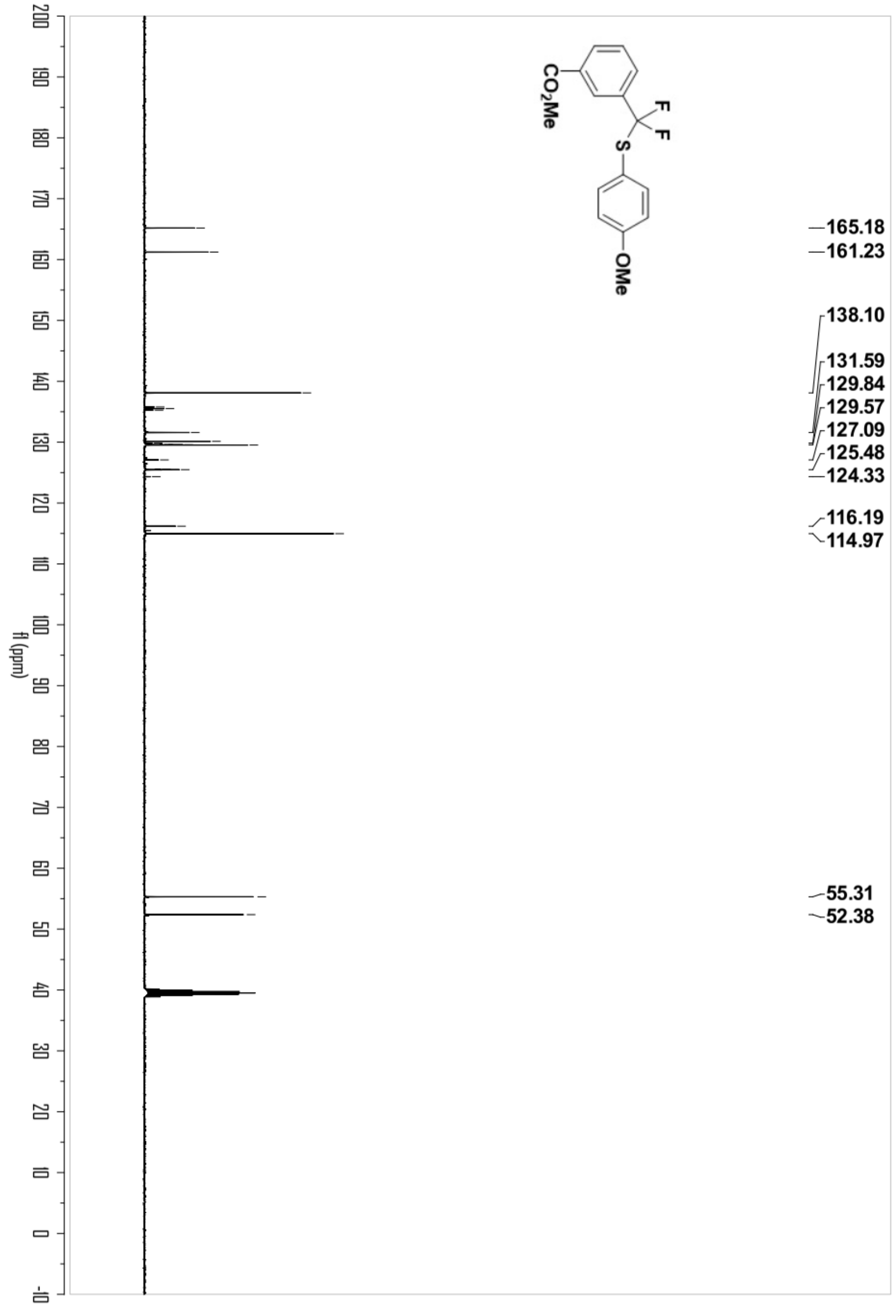
CO2Me
F F
S
OMe
165.18
161.23
138.10
131.59
129.84
129.57
127.09
125.48
124.33
116.19
114.97
55.31
52.38
f1 (ppm)

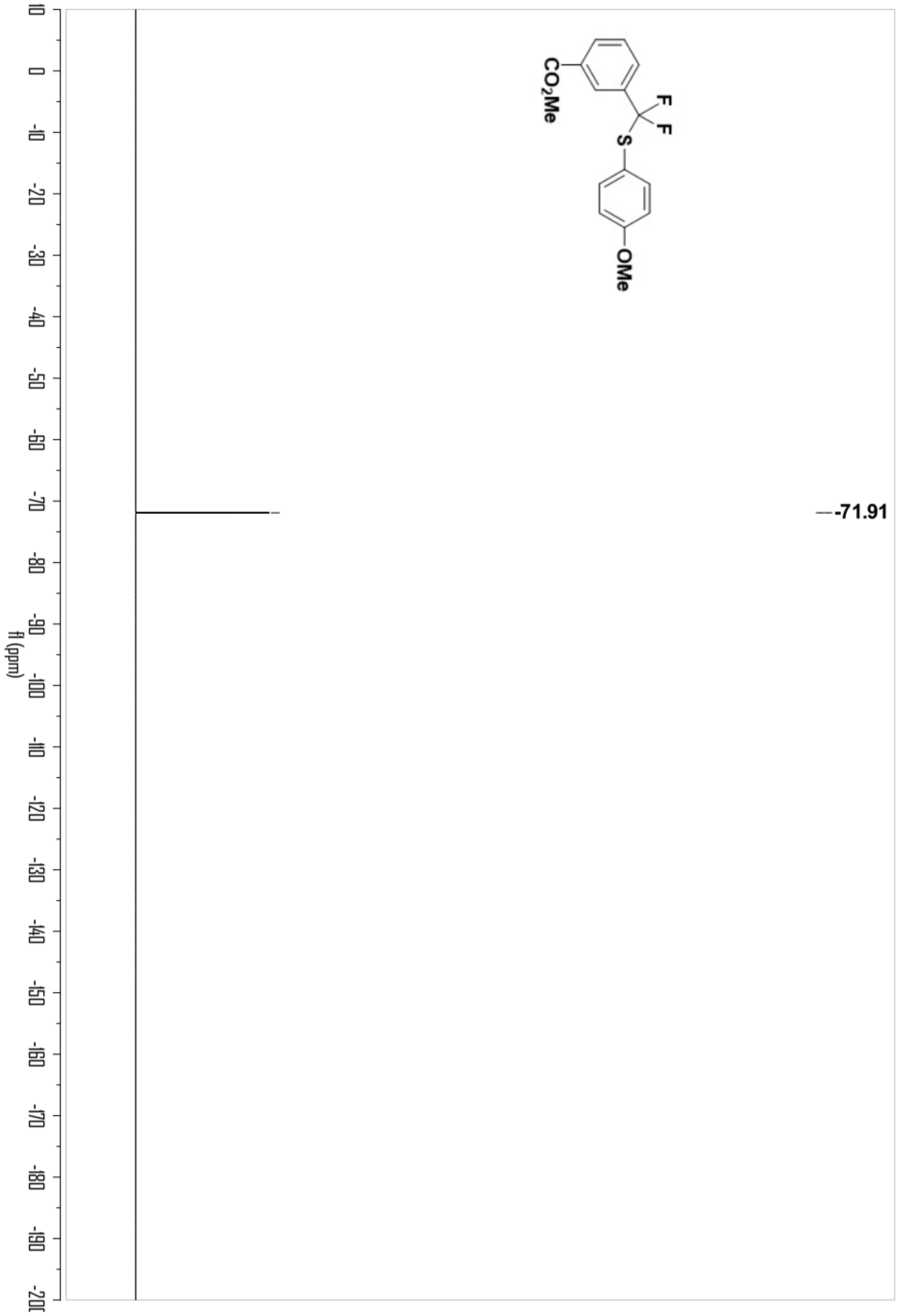

-71.91
f1 (ppm)
CO2Me
F
F
S
OMe

^{1}H, ^{13}C and ^{19}F NMR spectra of compound 7j

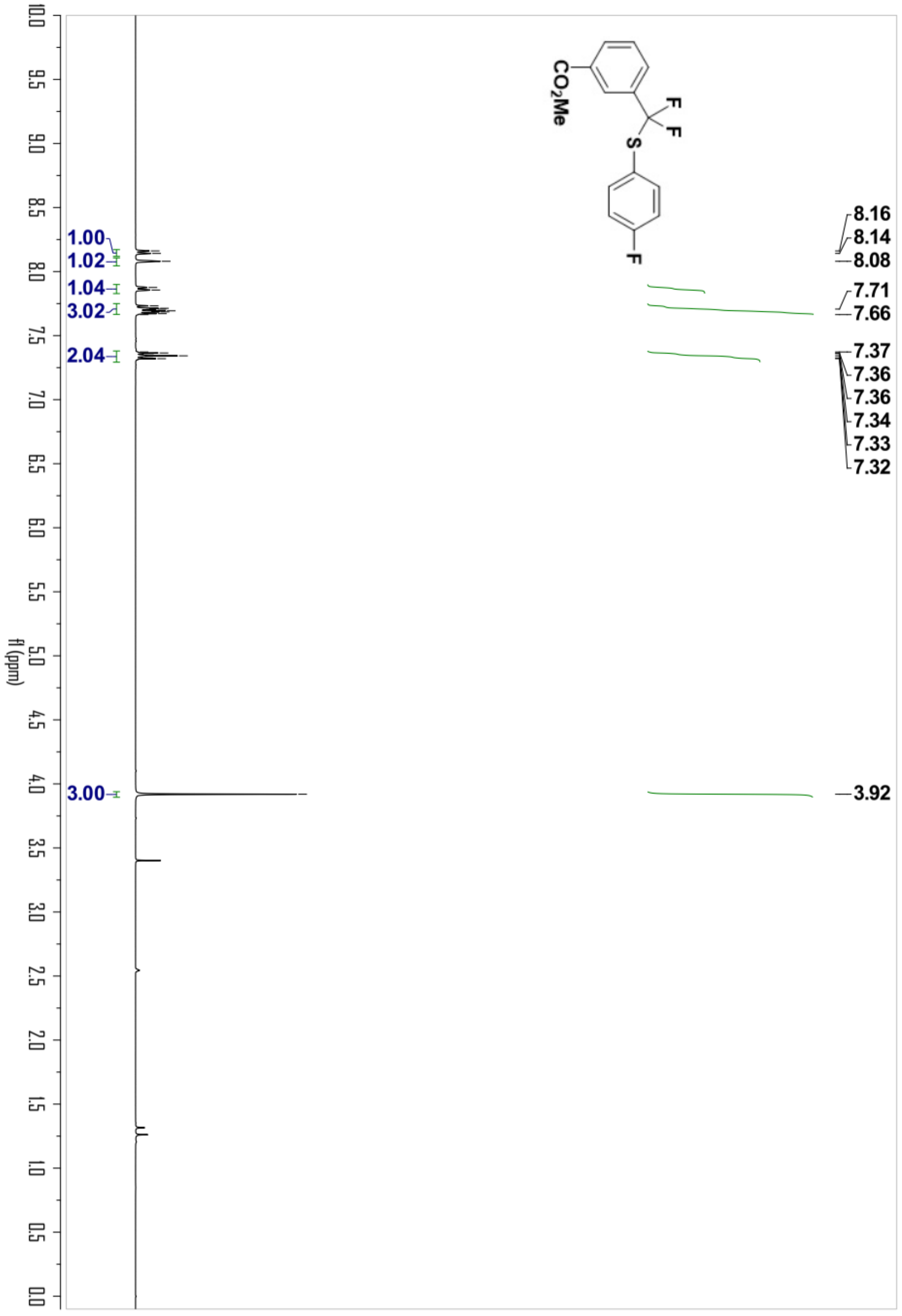

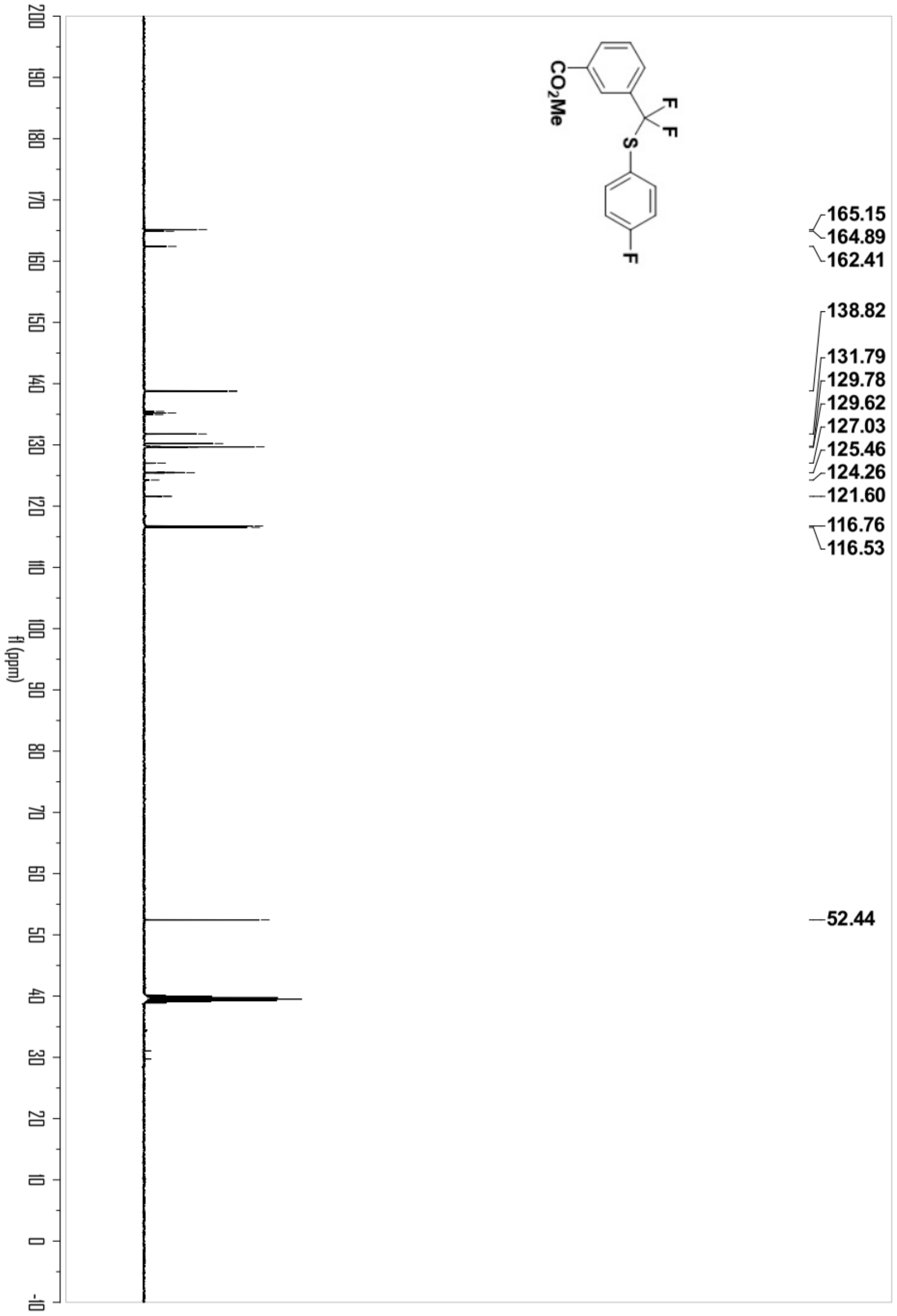
CO2Me
F
F
S
F
165.15
164.89
162.41
138.82
131.79
129.78
129.62
127.03
125.46
124.26
121.60
116.76
116.53
52.44
f1 (ppm)

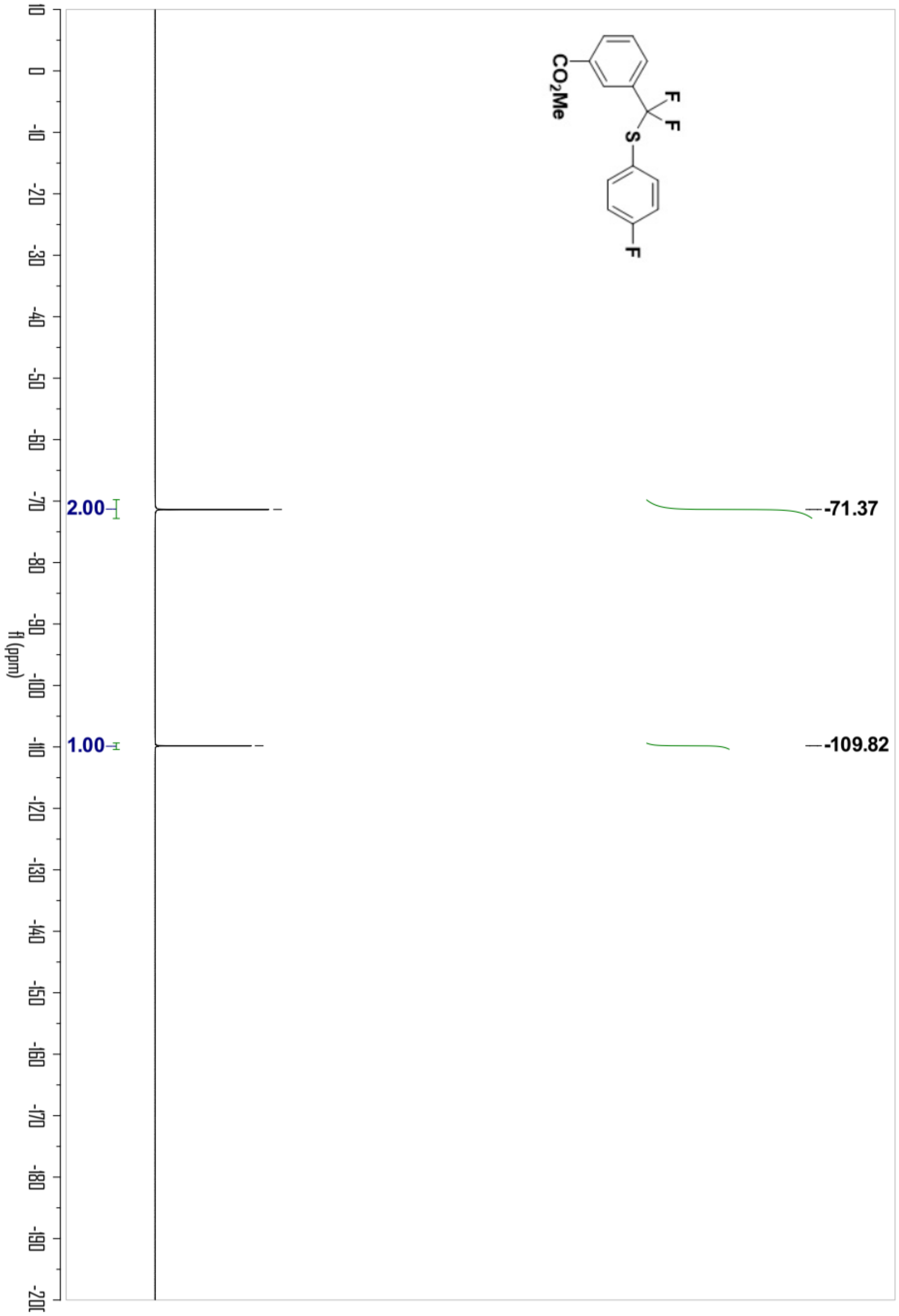

2.00
1.00
-71.37
-109.82
f1 (ppm)
CO₂Me
F
S

^{1}H, ^{13}C and ^{19}F NMR spectra of compound 10C

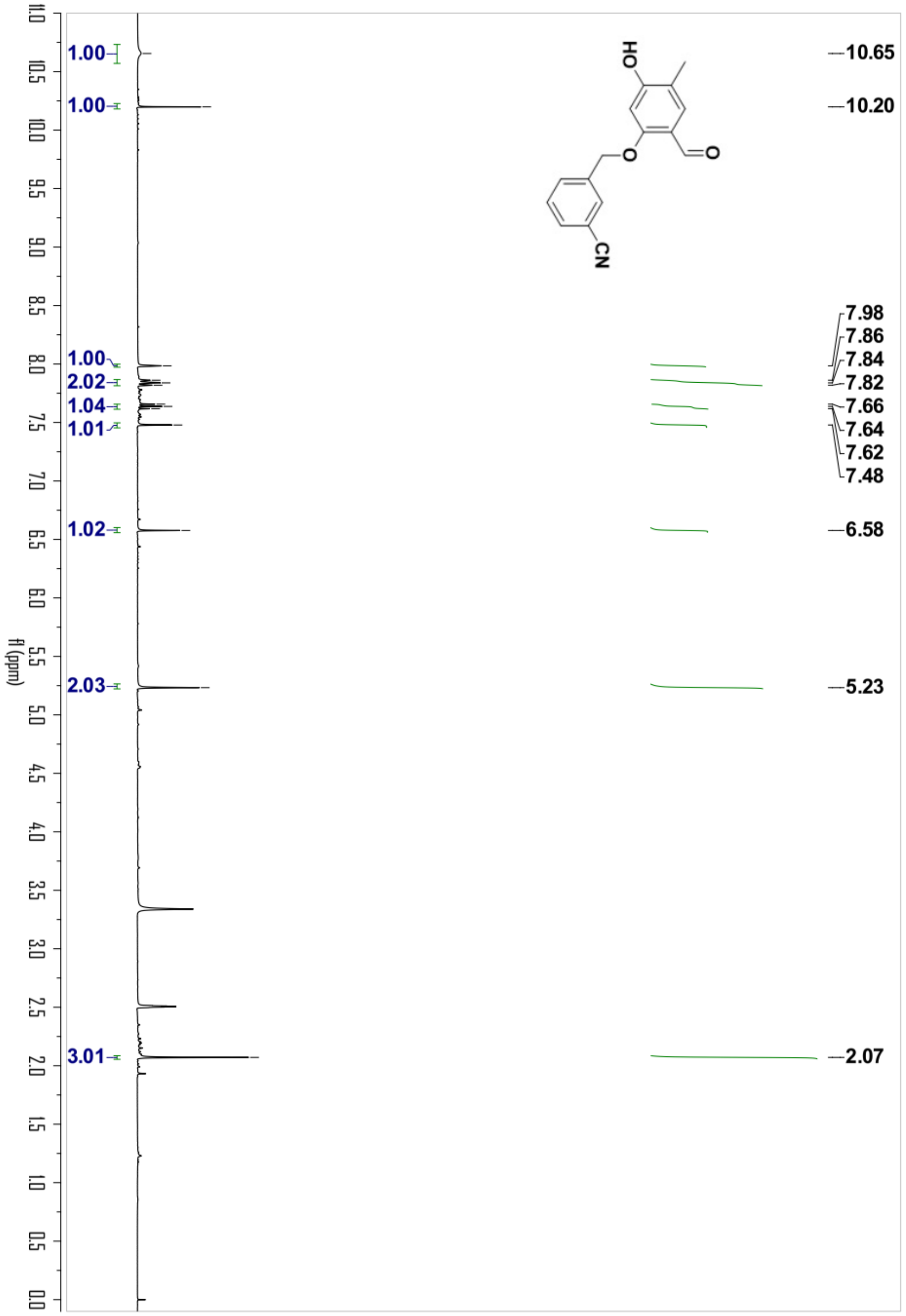

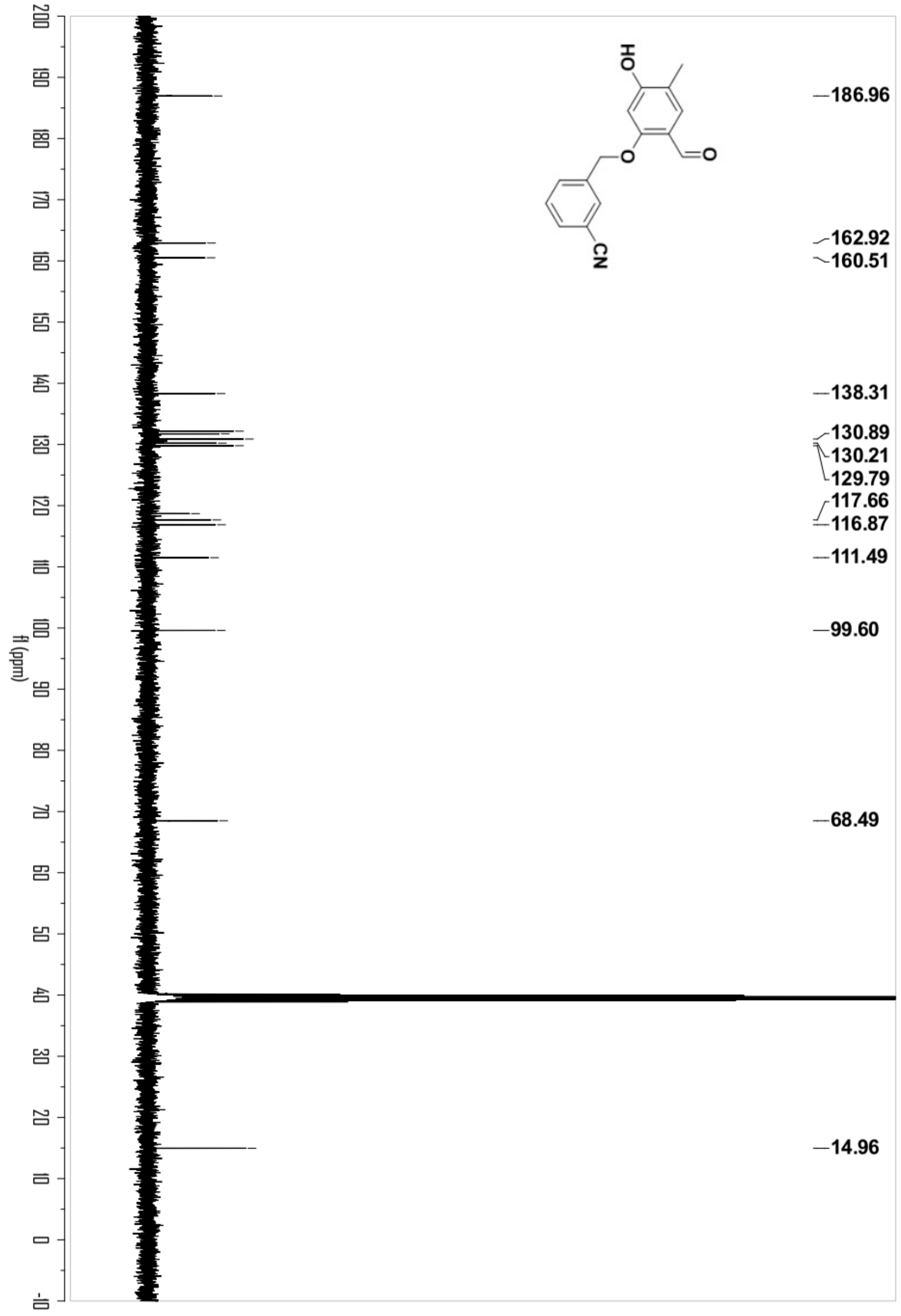
186.96
162.92
160.51
138.31
130.89
130.21
129.79
117.66
116.87
111.49
99.60
68.49
14.96
f1 (ppm)
HO
O
CN

^{1}H, ^{13}C and ^{19}F NMR spectra of compound 10D

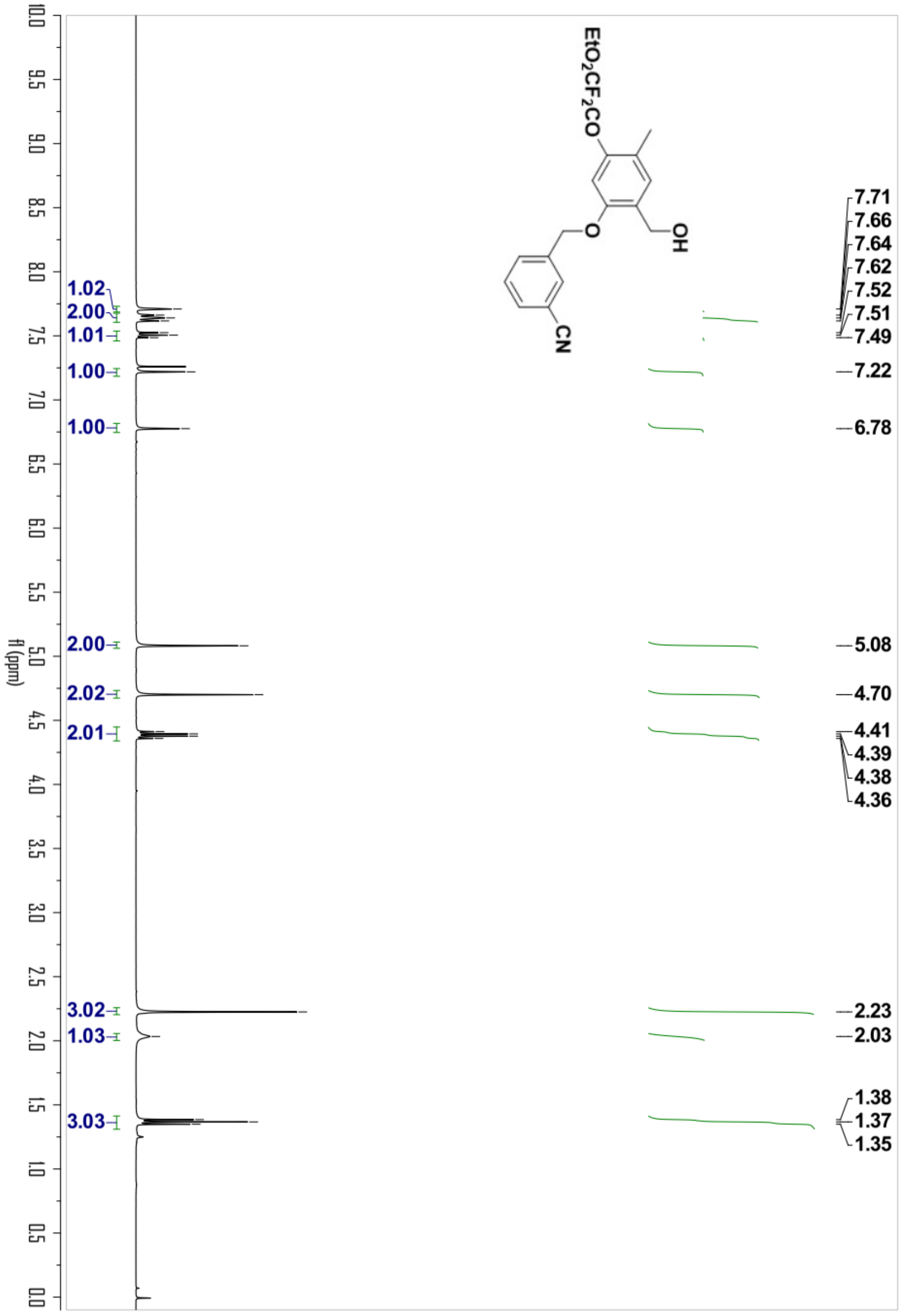

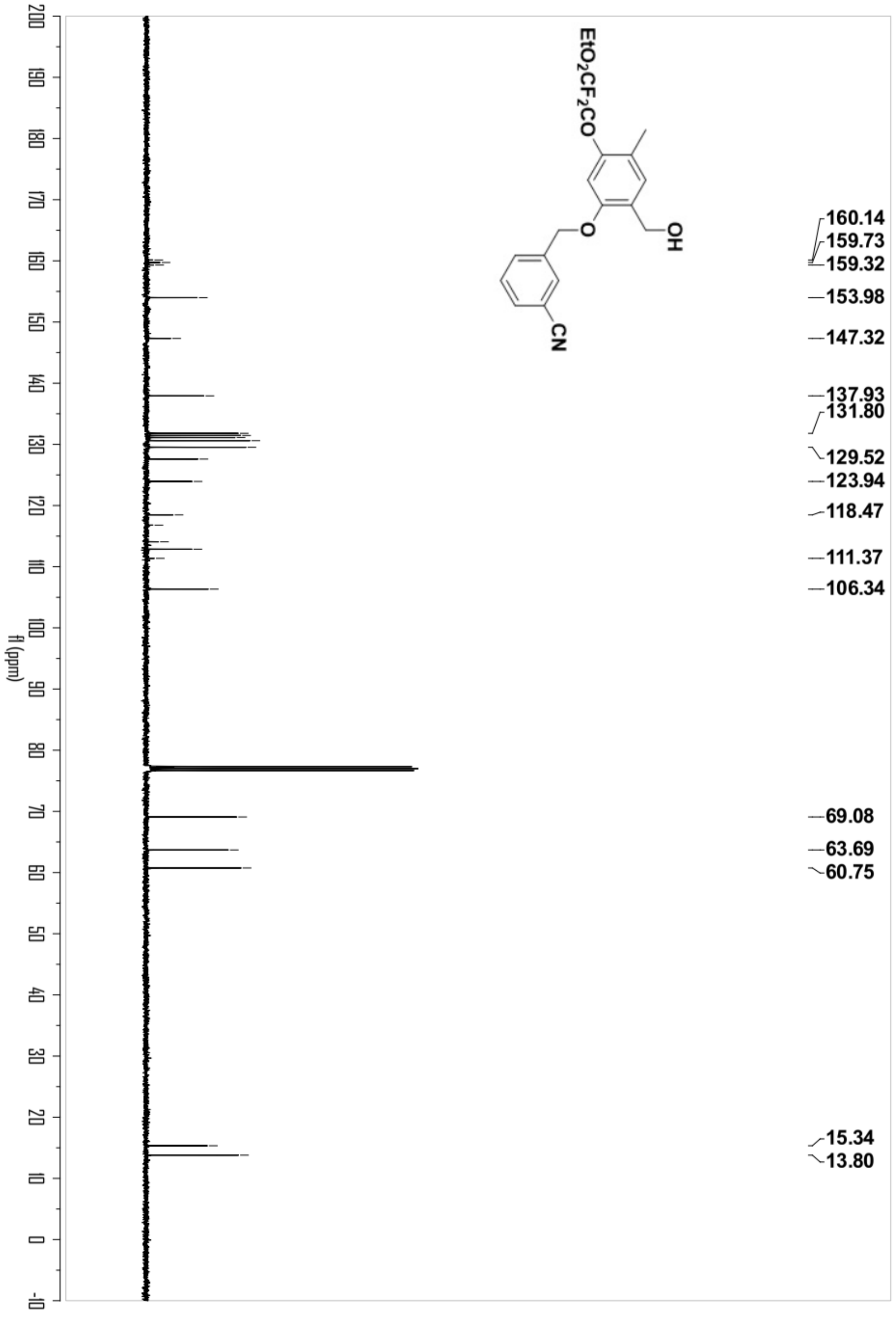
EtO2CF2CO
OH
O
CN
160.14
159.73
159.32
153.98
147.32
137.93
131.80
129.52
123.94
118.47
111.37
106.34
69.08
63.69
60.75
15.34
13.80
f1 (ppm)

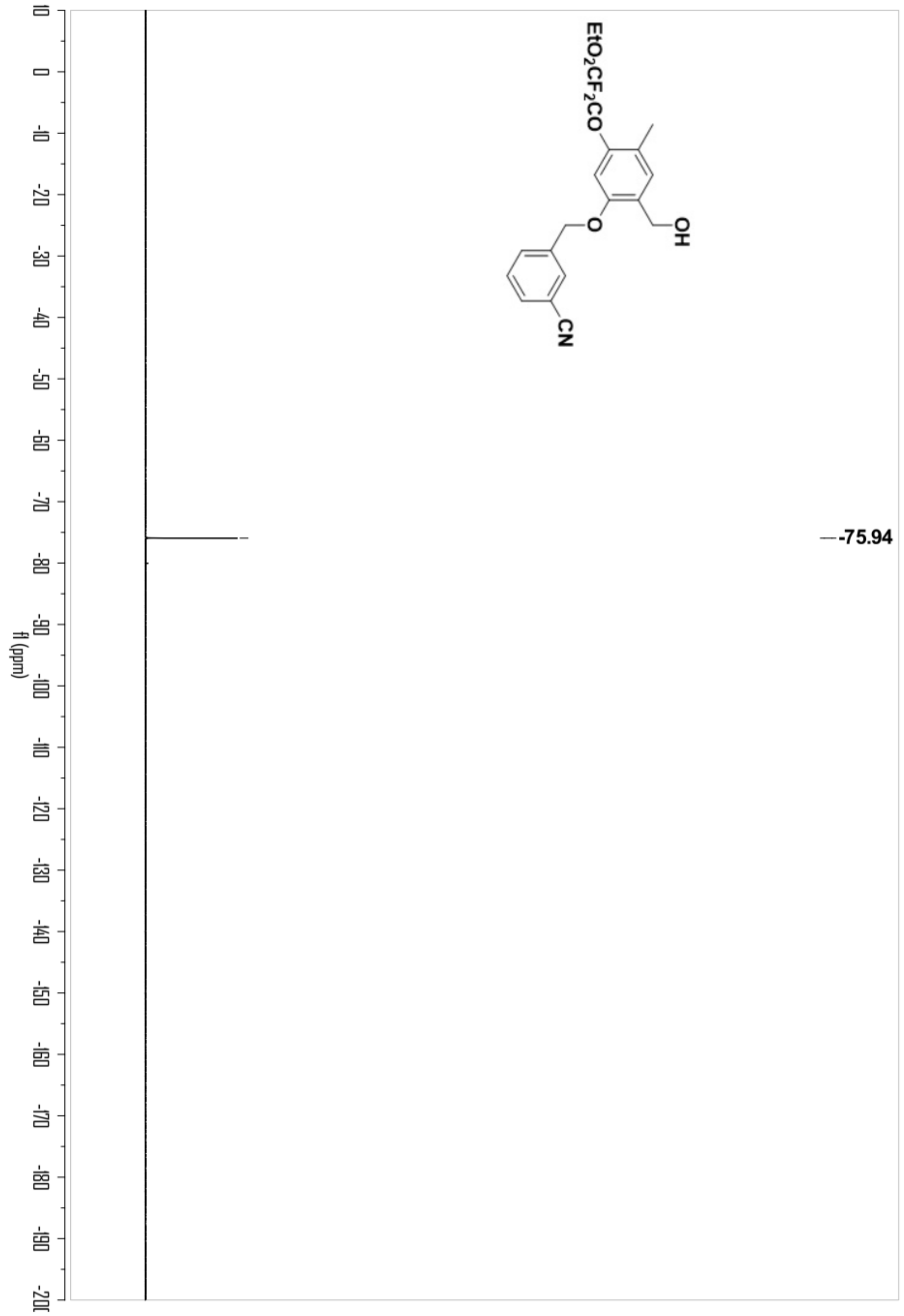
EtO2CF2CO
OH
CN
-75.94
f1 (ppm)

^{1}H, ^{13}C and ^{19}F NMR spectra of compound 10E

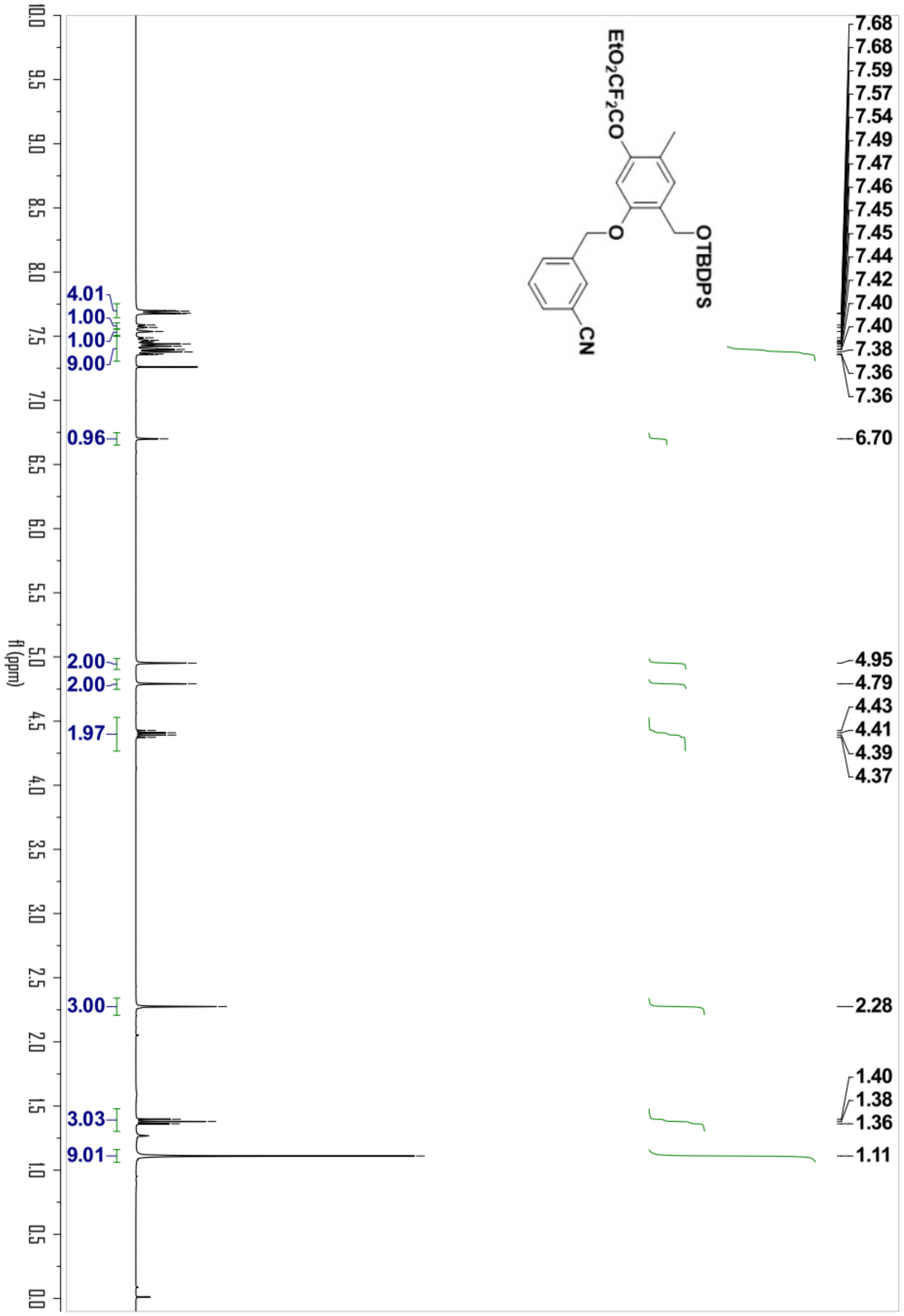

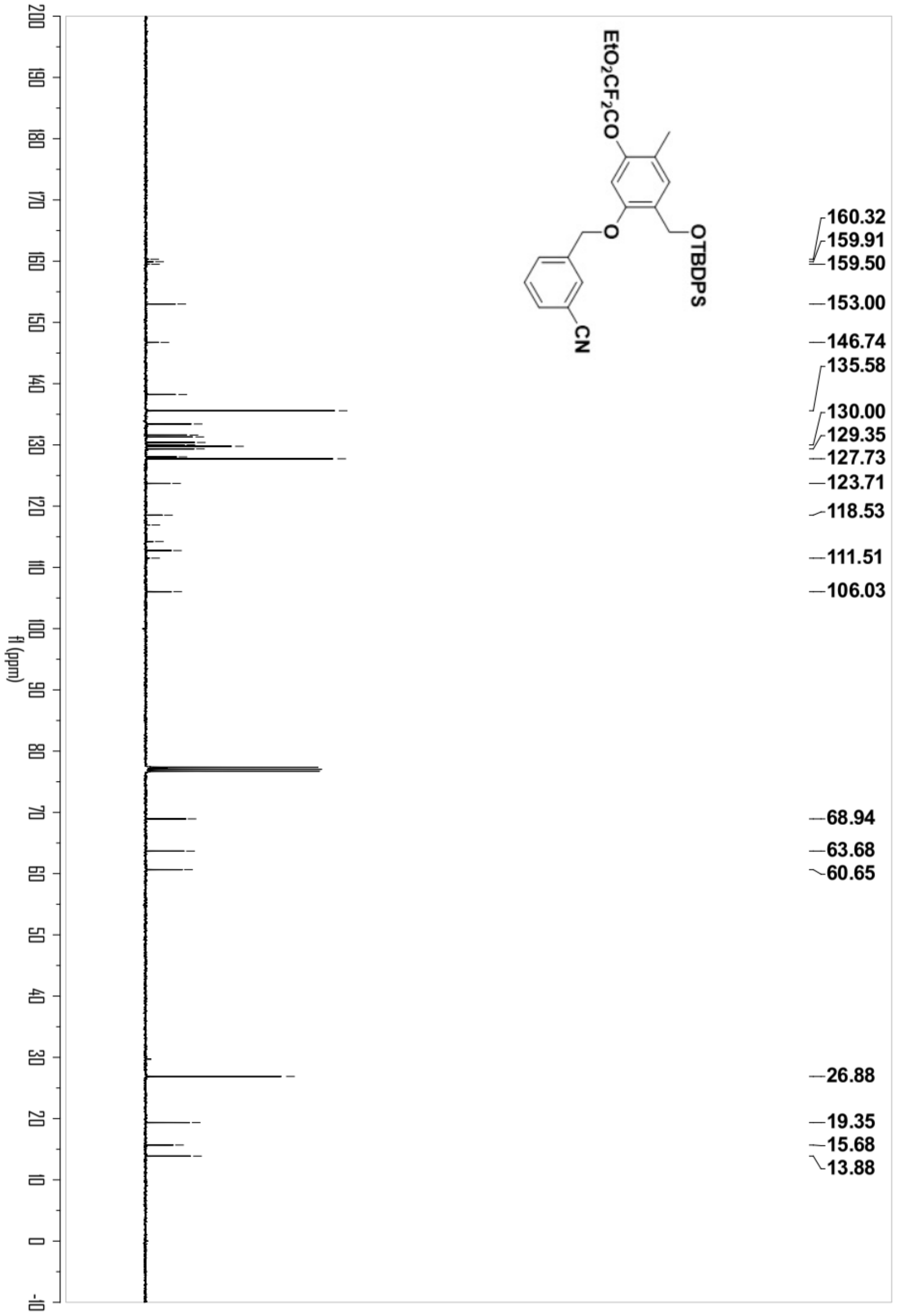
160.32
159.91
159.50
153.00
146.74
135.58
130.00
129.35
127.73
123.71
118.53
111.51
106.03
68.94
63.68
60.65
26.88
19.35
15.68
13.88
EtO_2CF_2CO
O
OTBDPS
CN
f1 (ppm)

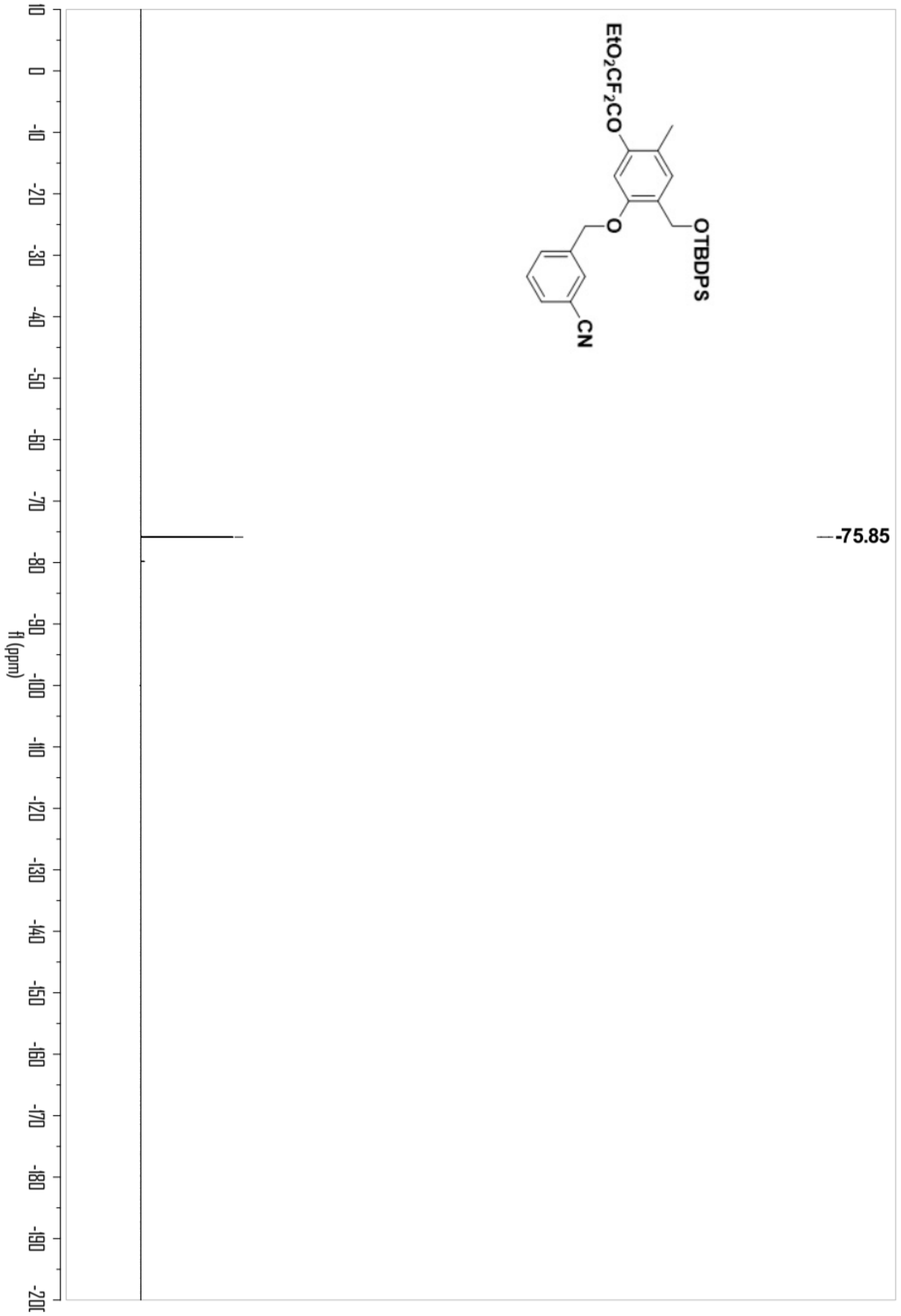
EtO2CF2CO
O
OTBDPS
CN
-75.85
f1 (ppm)

^{1}H, ^{13}C and ^{19}F NMR spectra of compound 10F

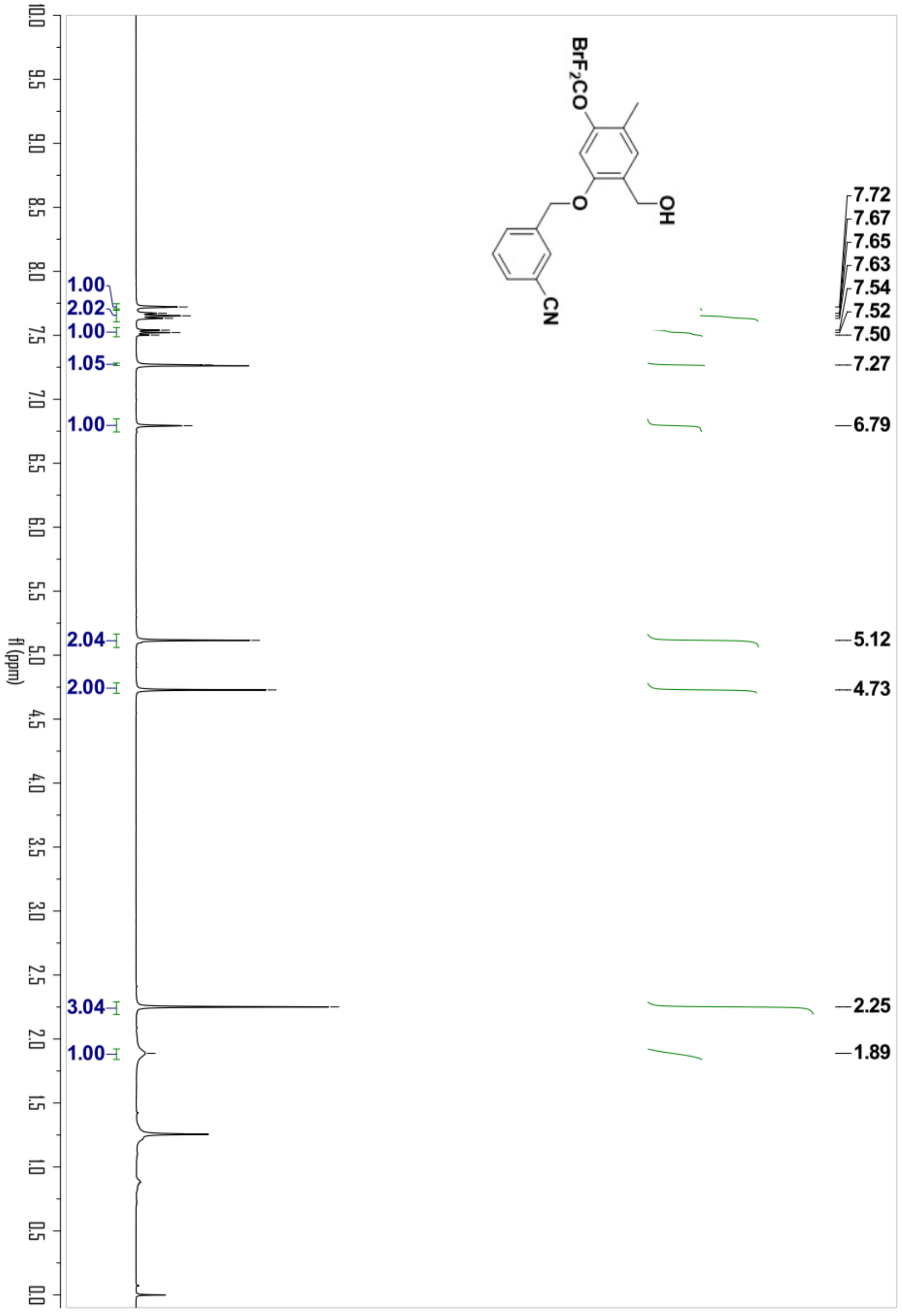

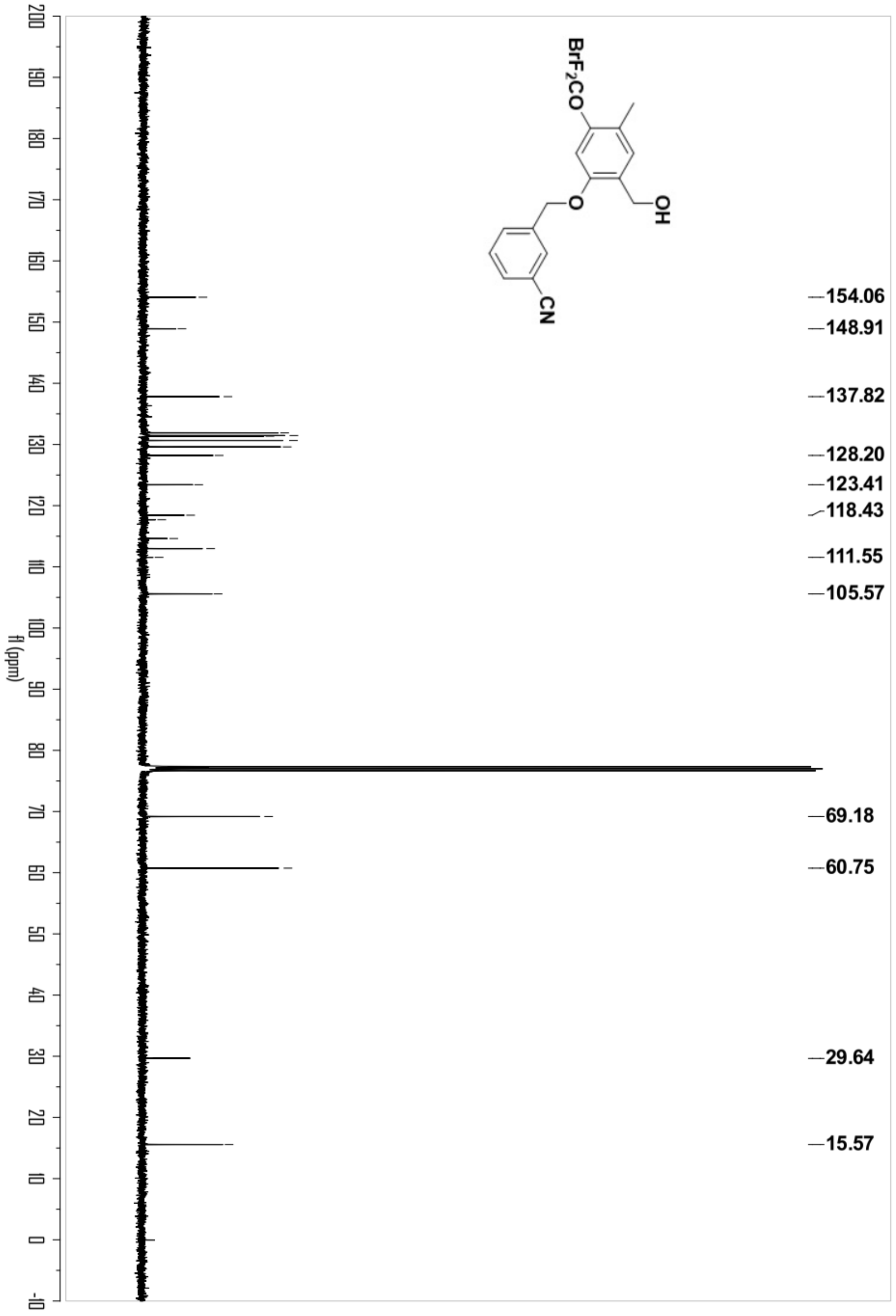

BrF2CO
OH
O
CN
154.06
148.91
137.82
128.20
123.41
118.43
111.55
105.57
69.18
60.75
29.64
15.57
f1 (ppm)

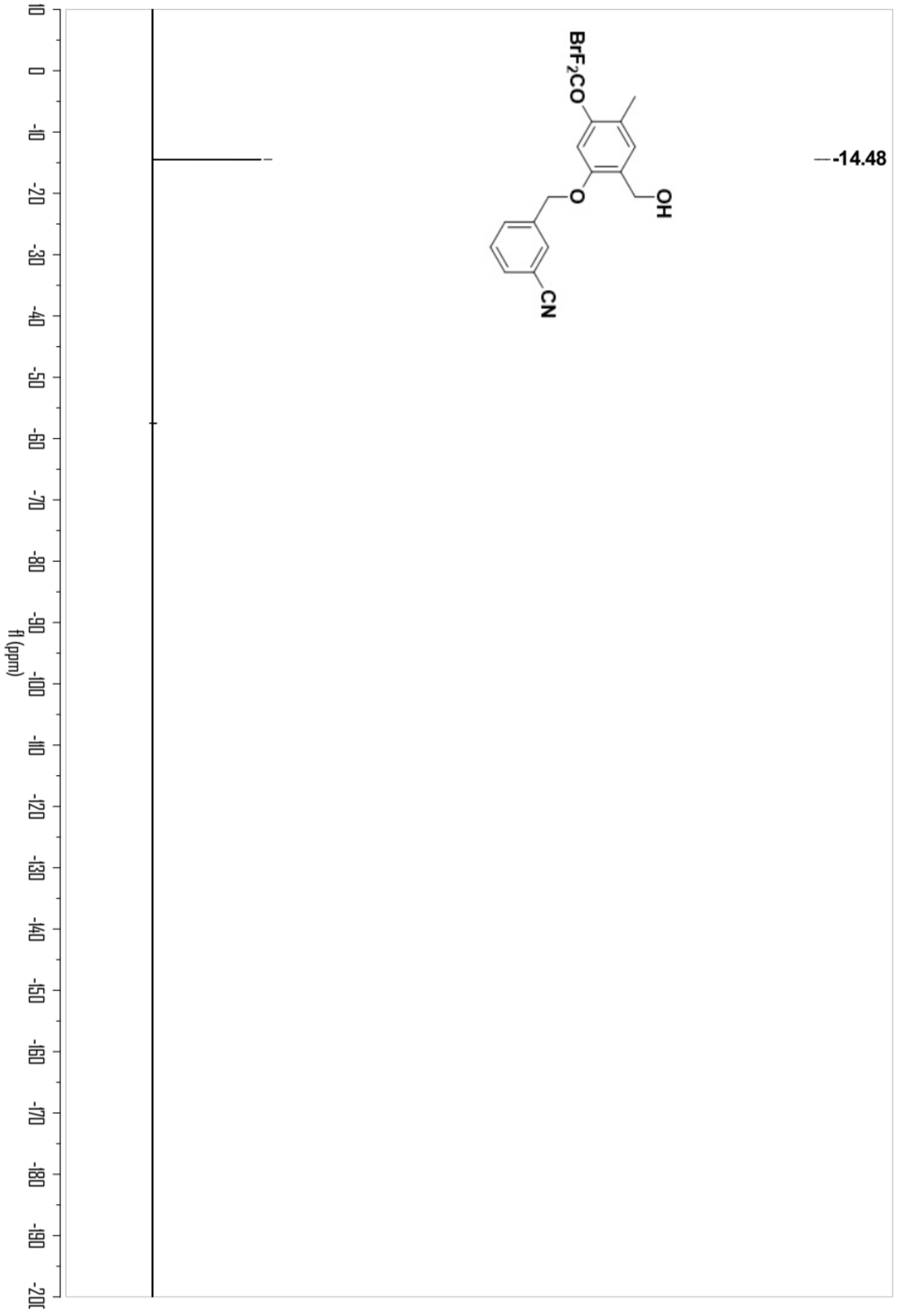

-14.48
BrF2CO
OH
CN
f1 (ppm)

^{1}H, ^{13}C and ^{19}F NMR spectra of compound 10G

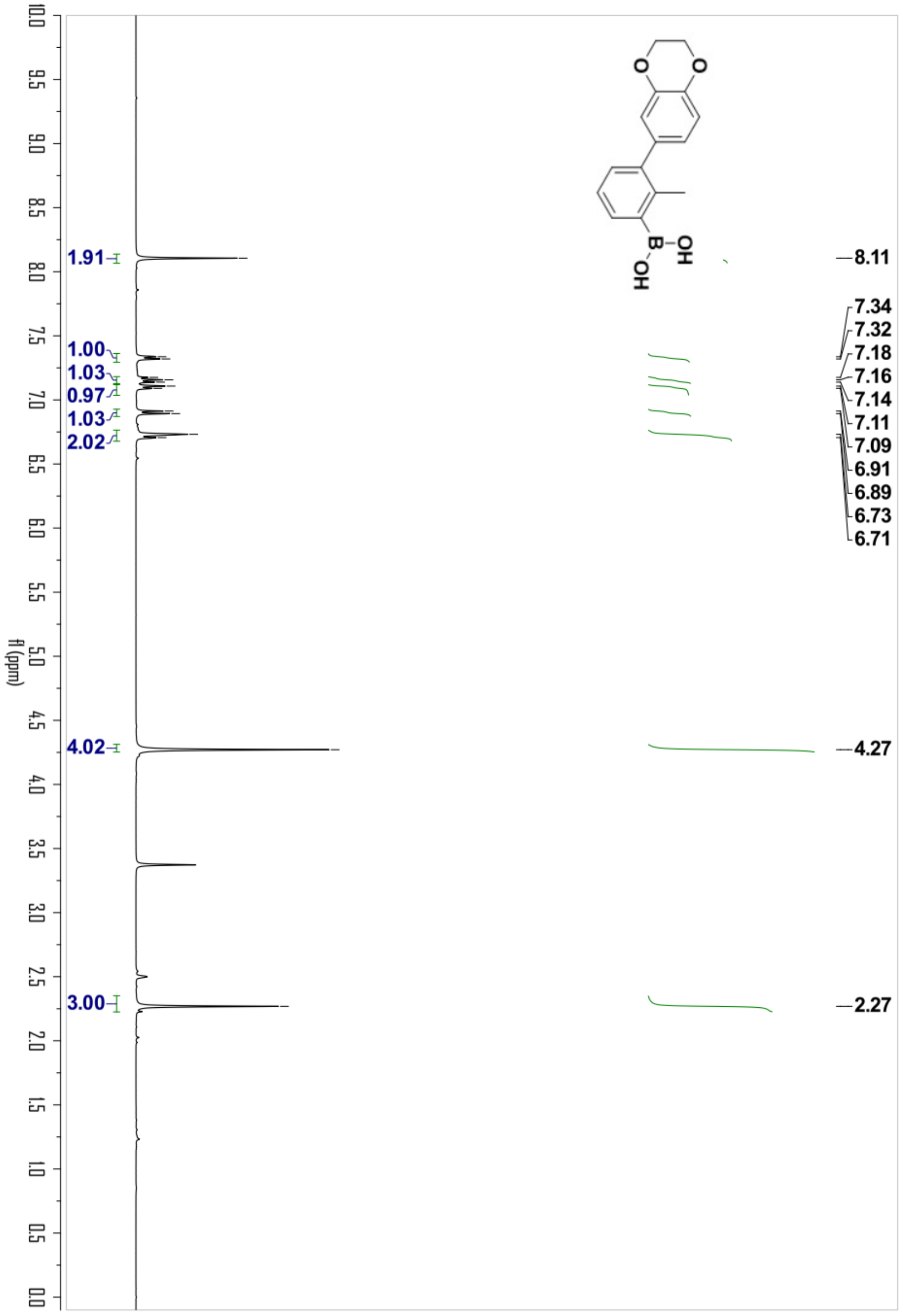

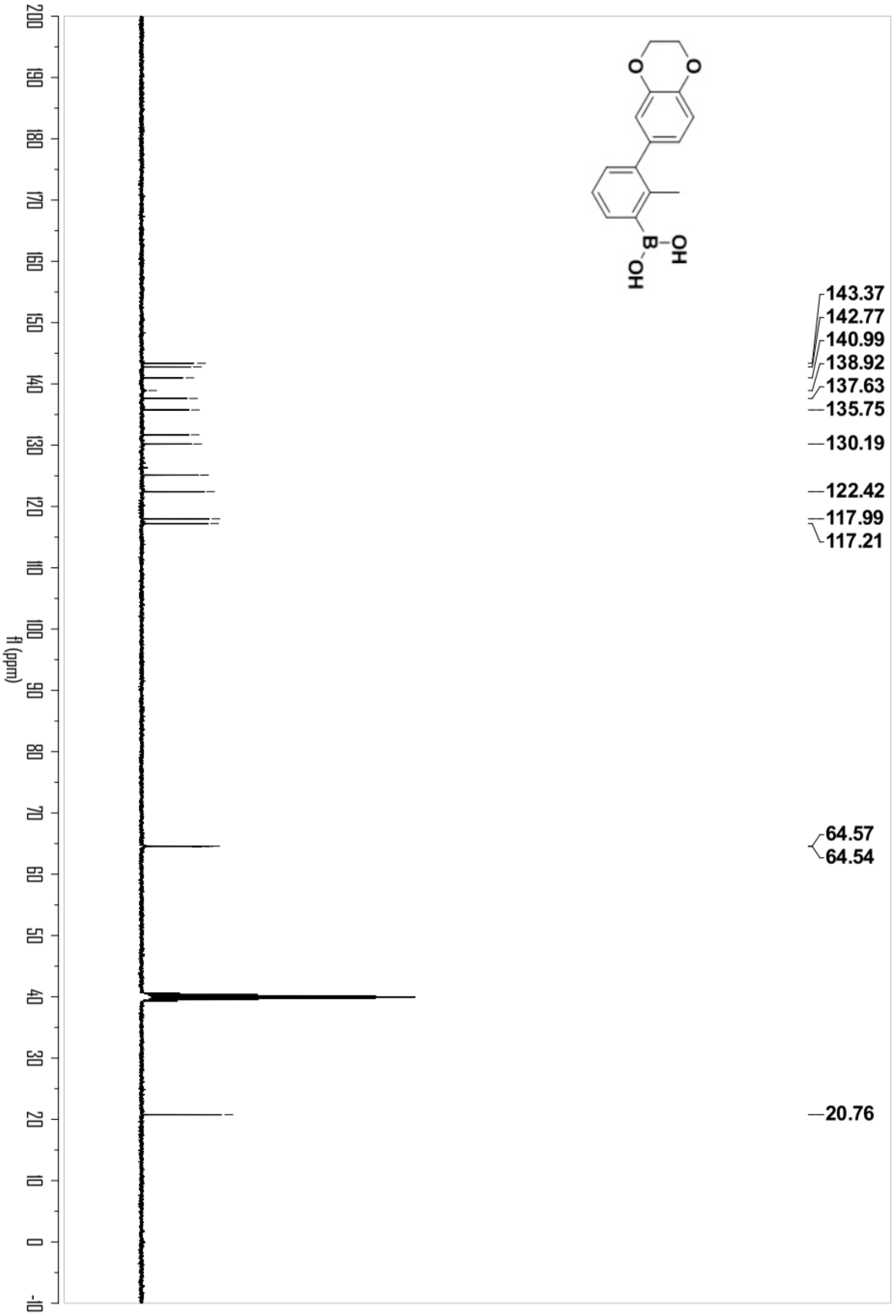
143.37
142.77
140.99
138.92
137.63
135.75
130.19
122.42
117.99
117.21
64.57
64.54
20.76
OH
B
OH
O
O
f1 (ppm)

^{1}H, ^{13}C and ^{19}F NMR spectra of compound 10H

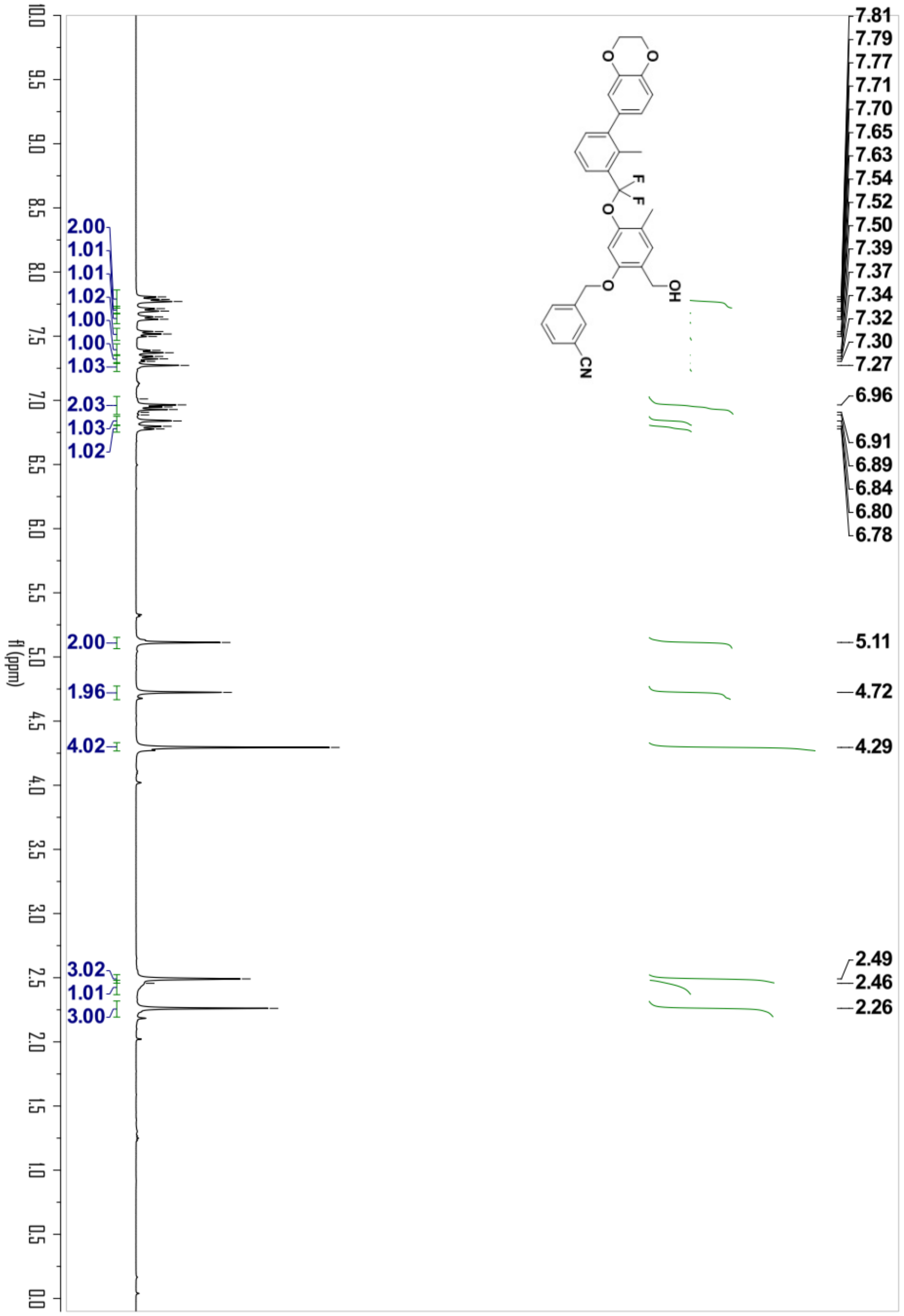

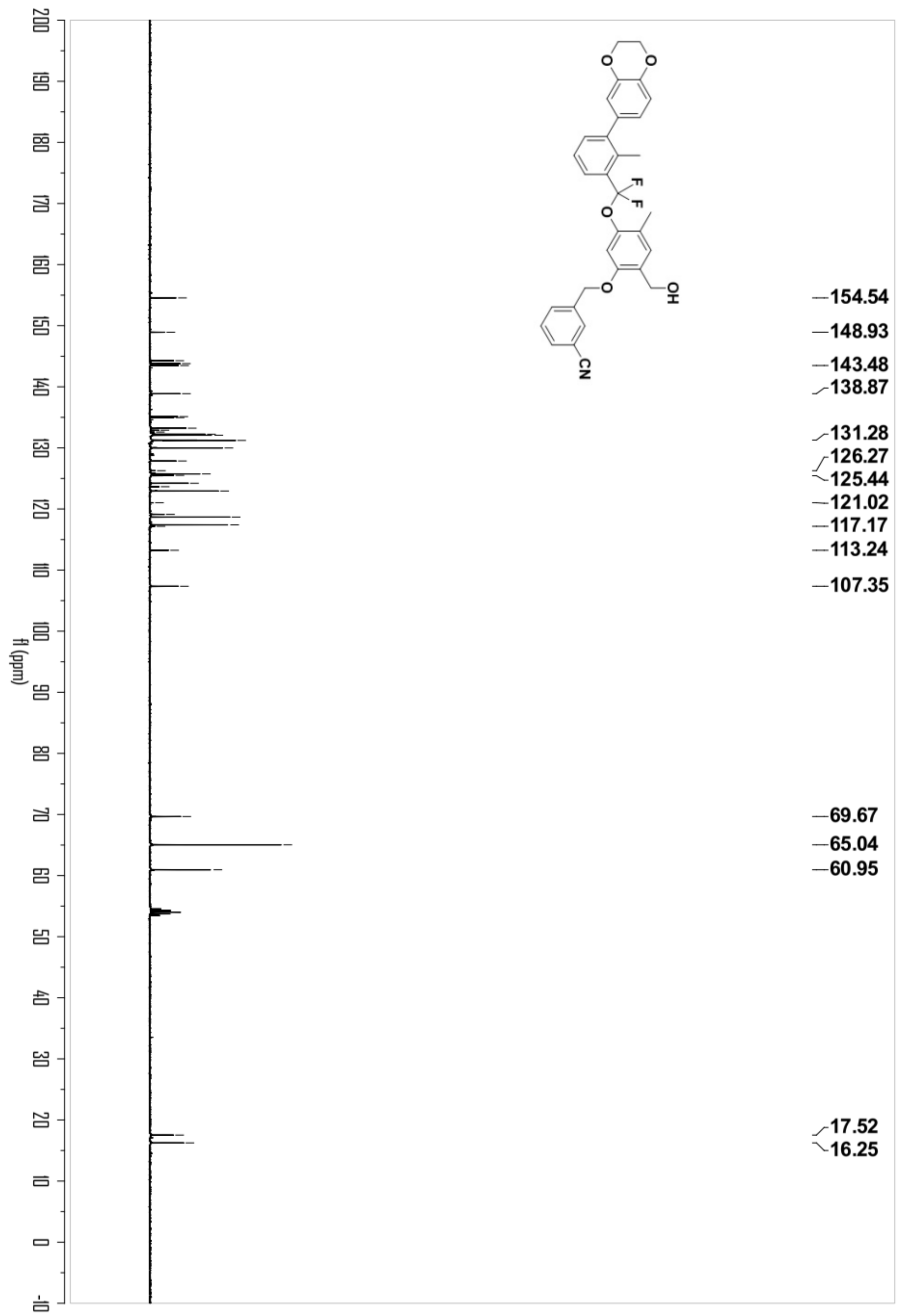
154.54
148.93
143.48
138.87
131.28
126.27
125.44
121.02
117.17
113.24
107.35
69.67
65.04
60.95
17.52
16.25
f1 (ppm)
OH
CN
F
F

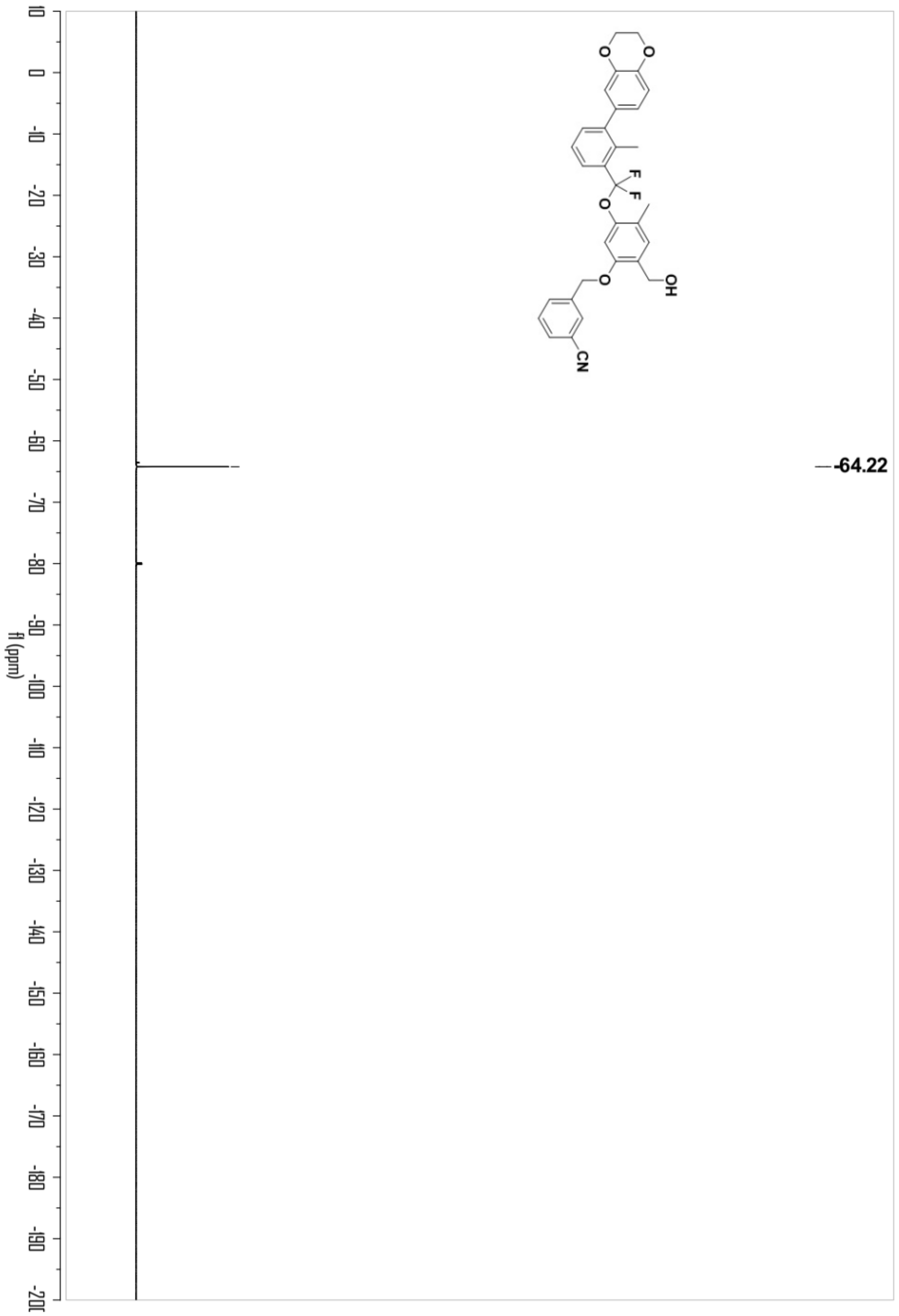
-64.22
f1 (ppm)
O
O
F
F
OH
CN

^{1}H, ^{13}C and ^{19}F NMR spectra of compound 10Ib

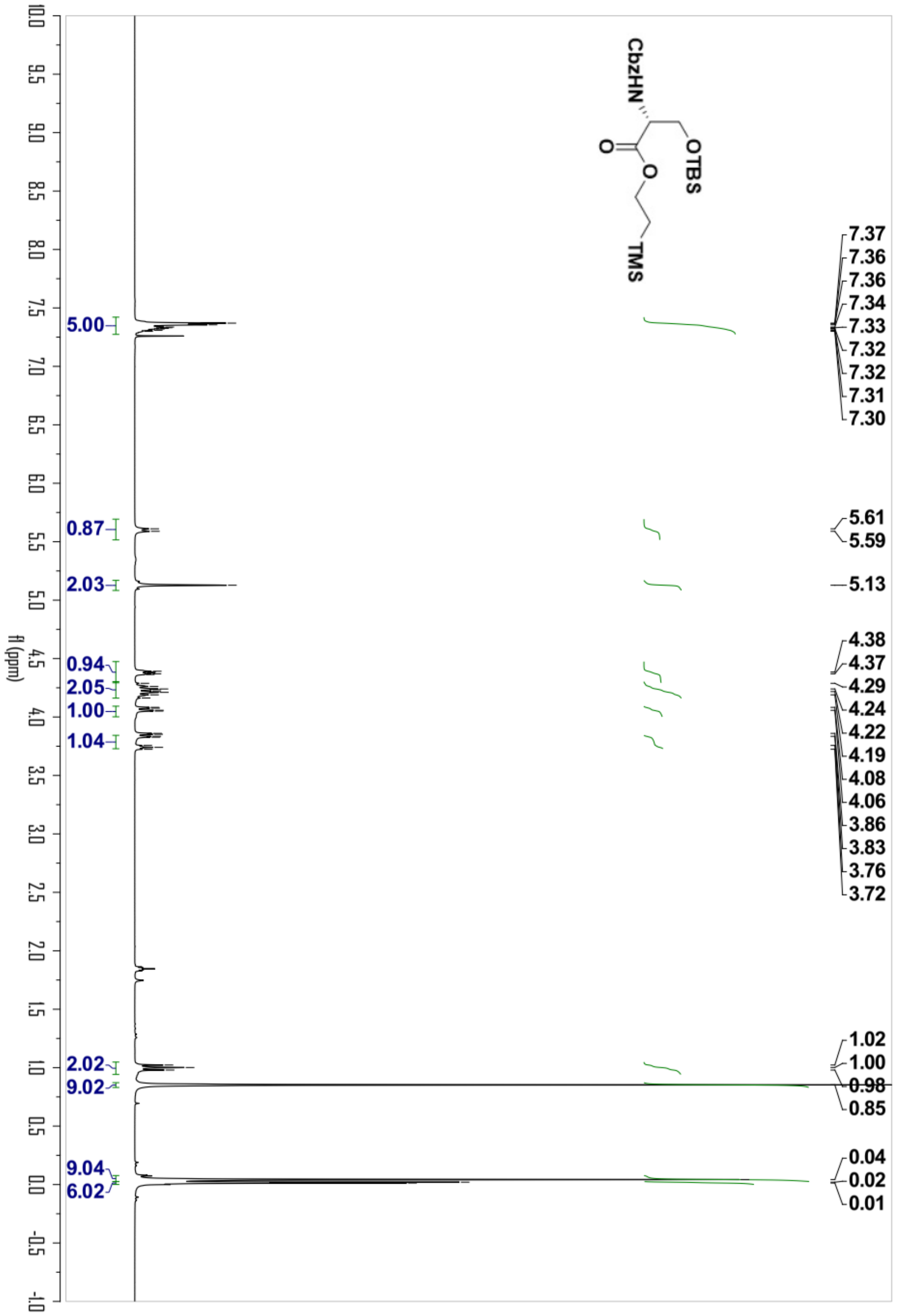

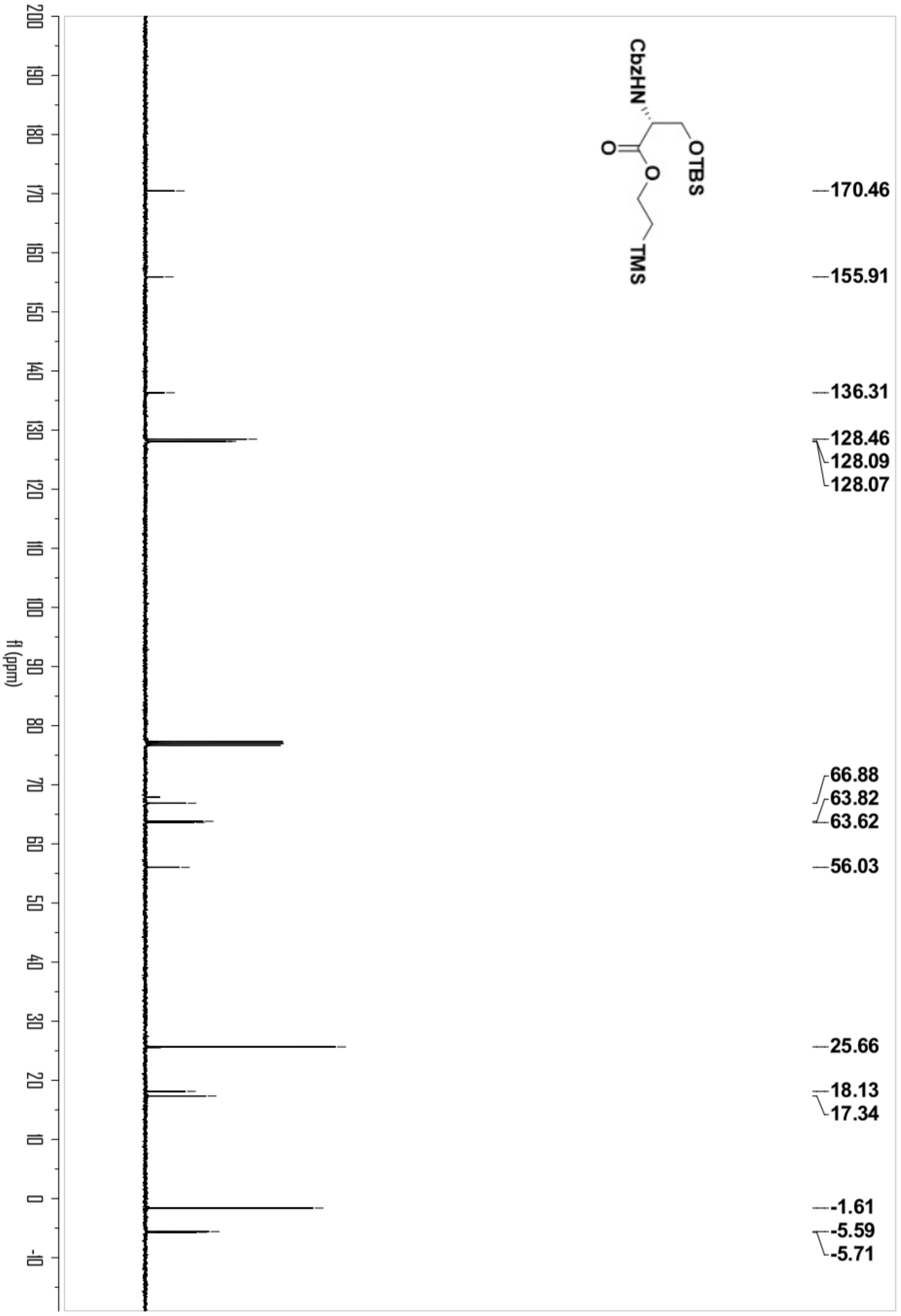
CbzHN
OTBS
O
O
TMS
170.46
155.91
136.31
128.46
128.09
128.07
66.88
63.82
63.62
56.03
25.66
18.13
17.34
-1.61
-5.59
-5.71
f1 (ppm)

^{1}H, ^{13}C and ^{19}F NMR spectra of compound 10I

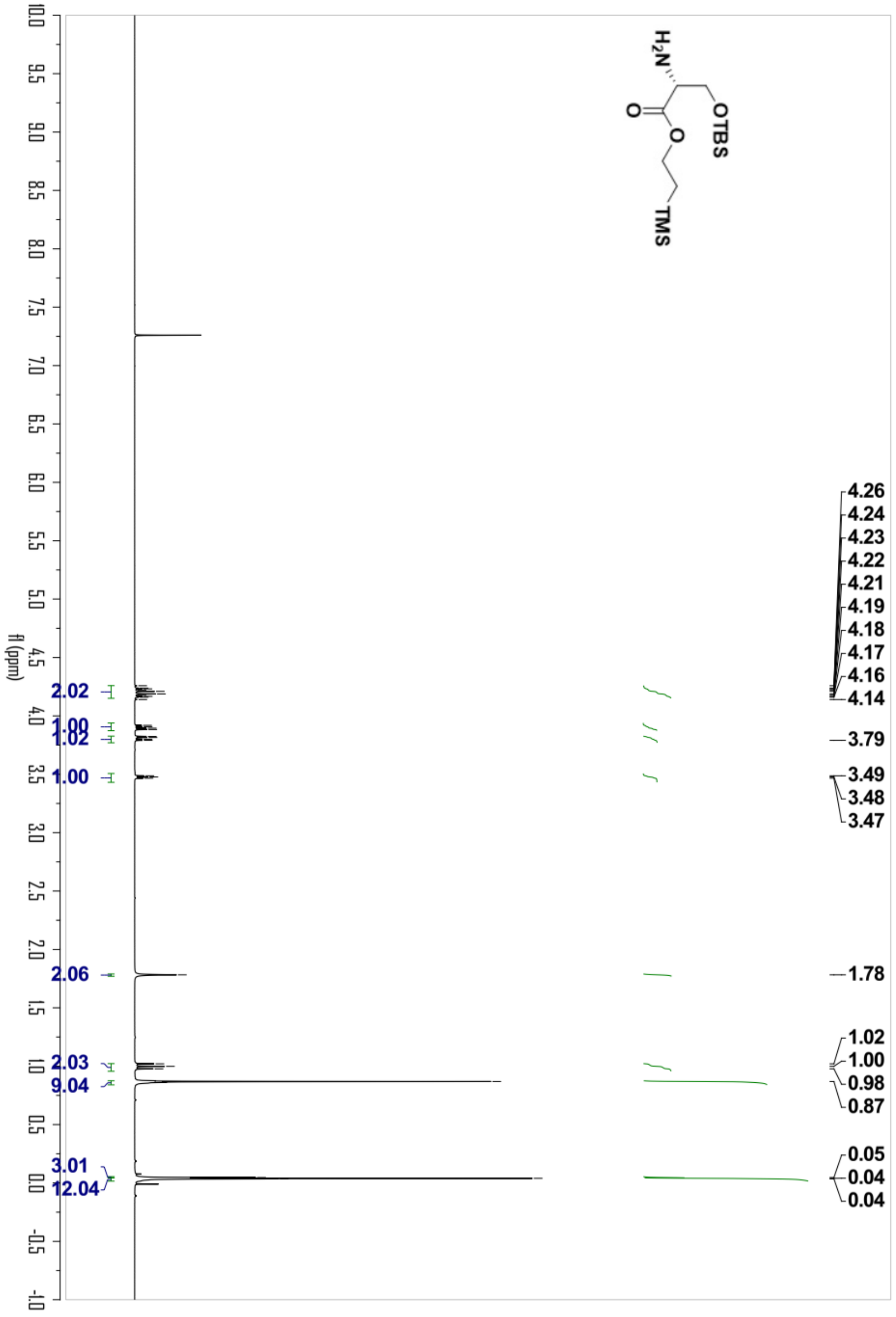

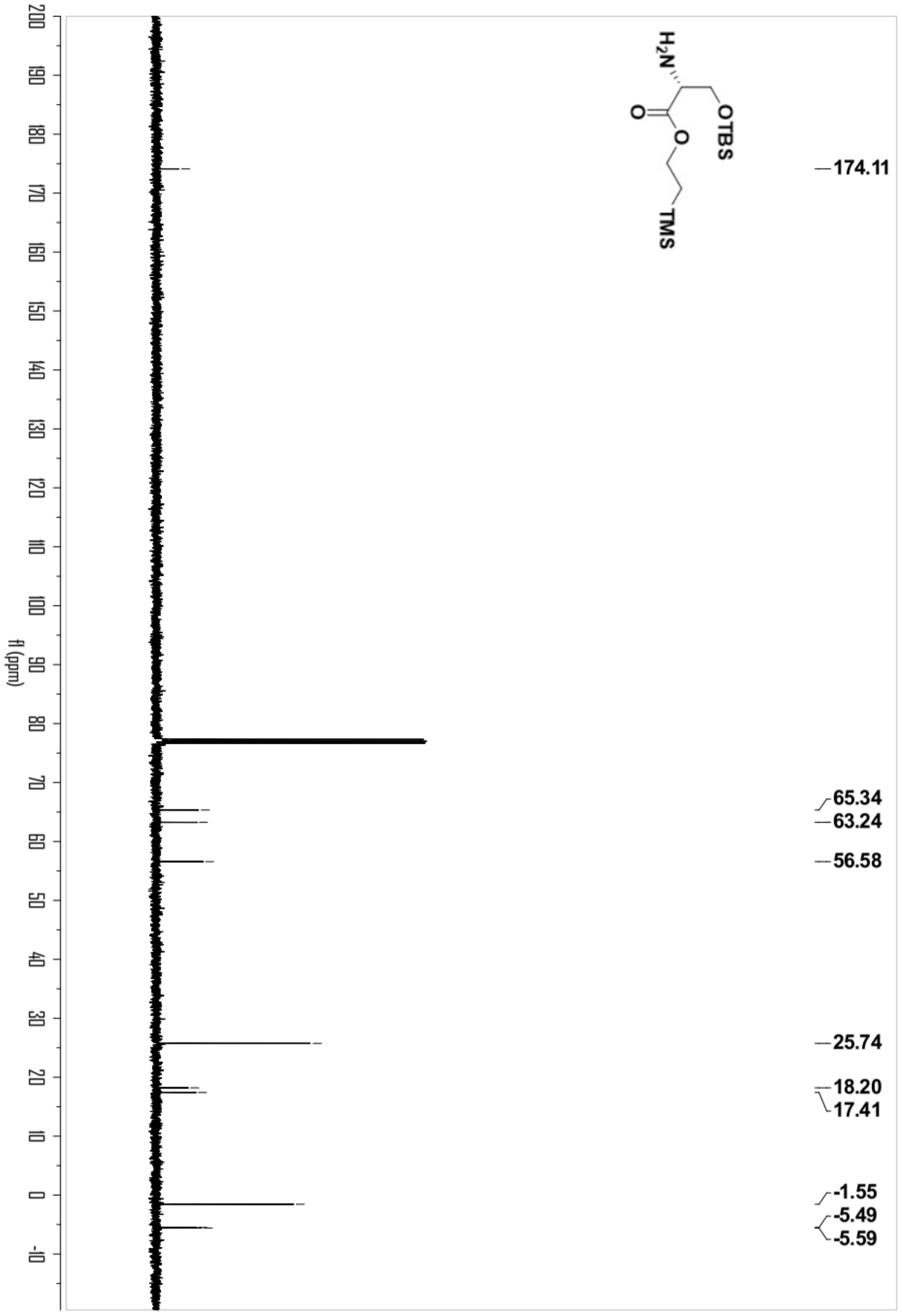

174.11
65.34
63.24
56.58
25.74
18.20
17.41
-1.55
-5.49
-5.59

^{1}H, ^{13}C and ^{19}F NMR spectra of compound 10J

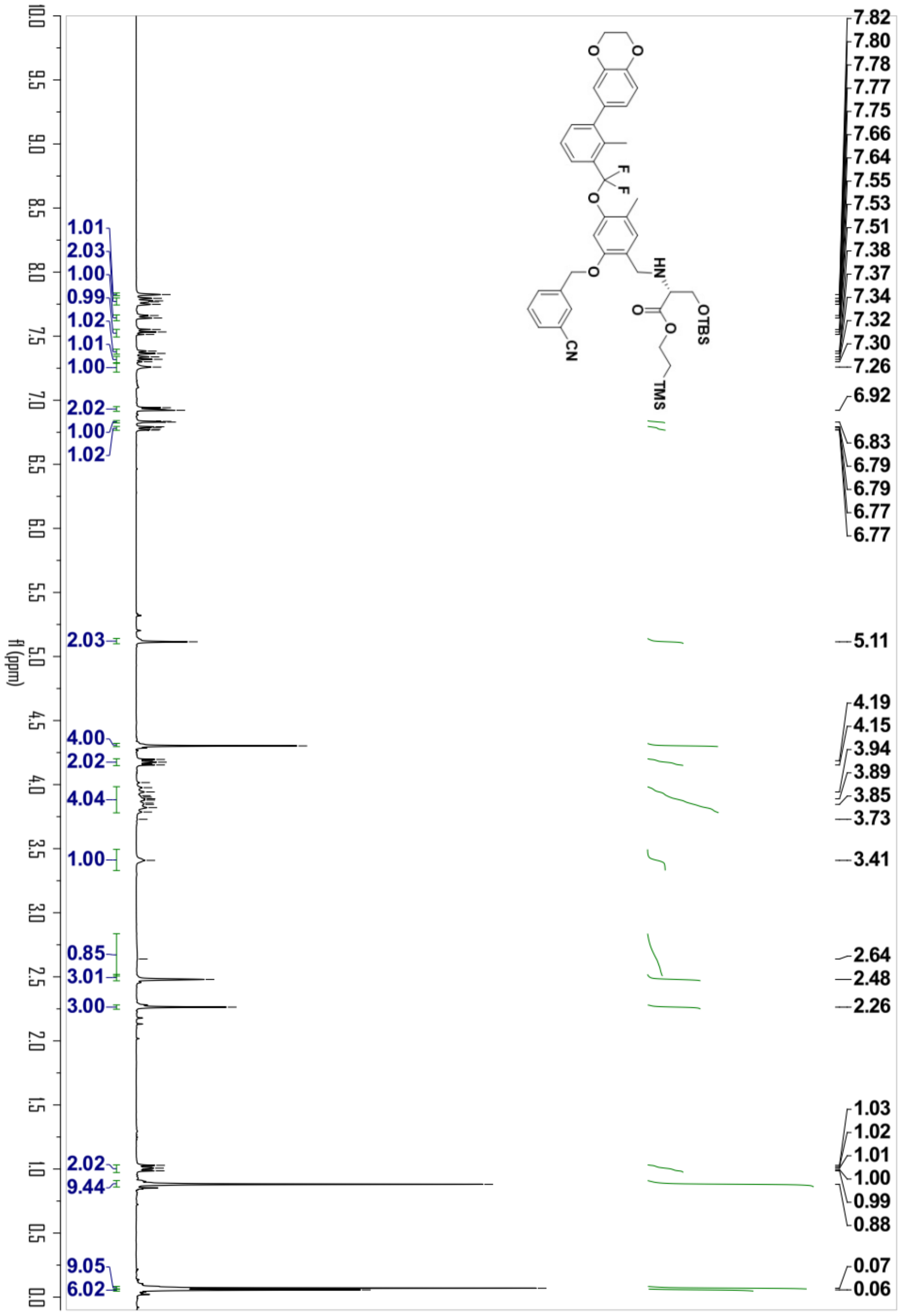

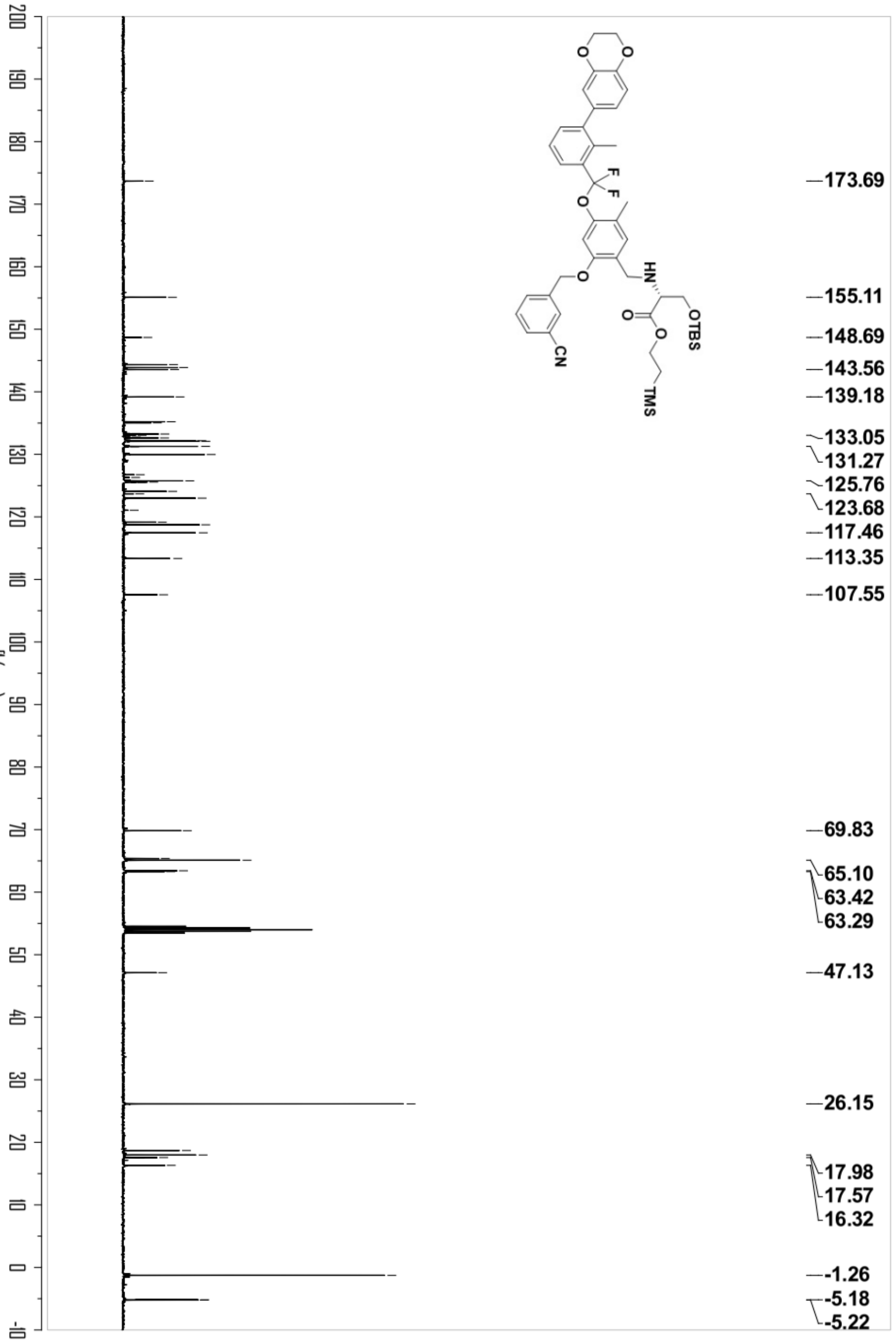

173.69
155.11
148.69
143.56
139.18
133.05
131.27
125.76
123.68
117.46
113.35
107.55
69.83
65.10
63.42
63.29
47.13
26.15
17.98
17.57
16.32
-1.26
-5.18
-5.22
f1 (ppm)
F
F
O
HN
OTBS
CN
TMS

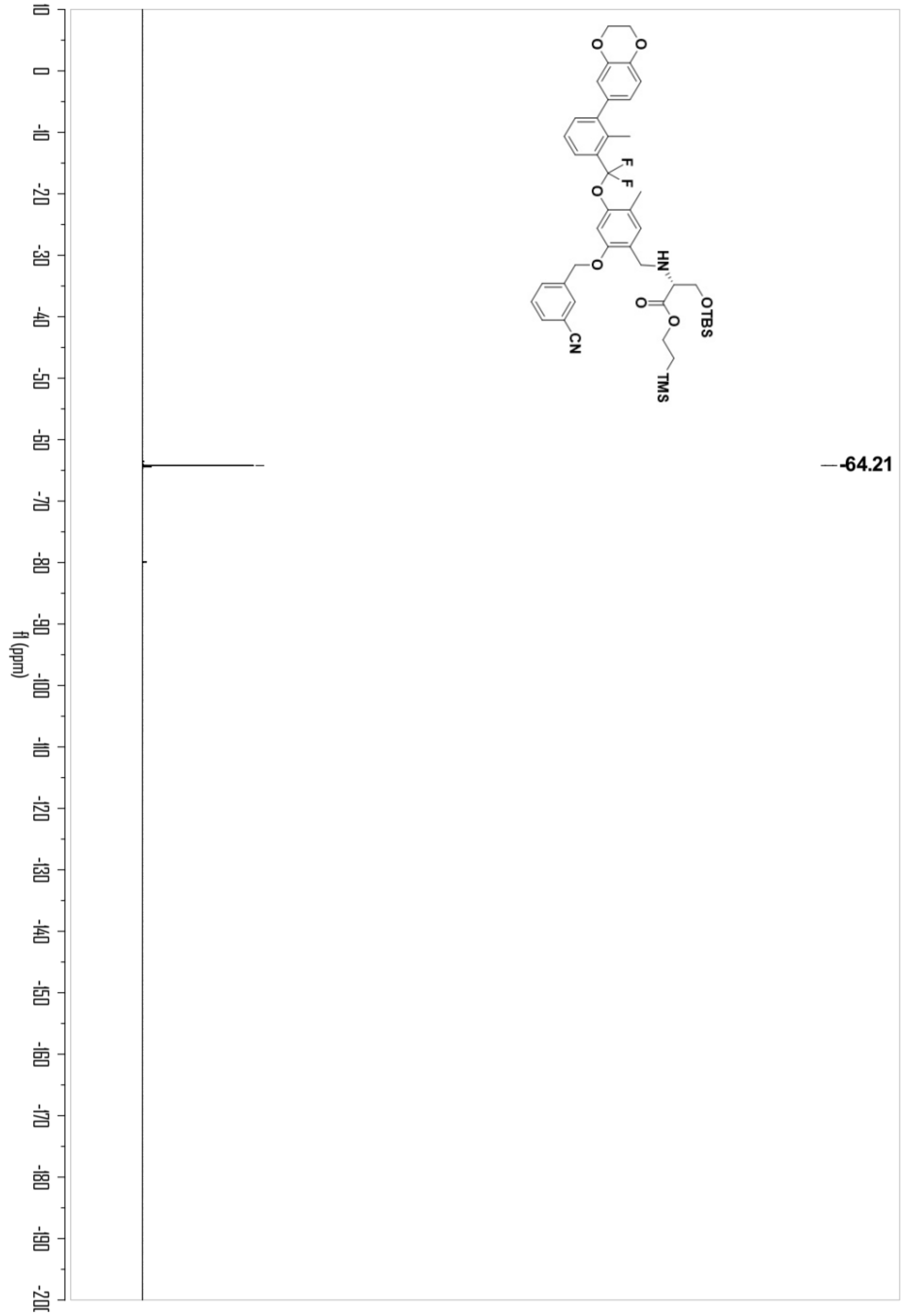
-64.21
f1 (ppm)
OTBS
TMS
CN
HN

^{1}H, ^{13}C and ^{19}F NMR spectra of compound 10

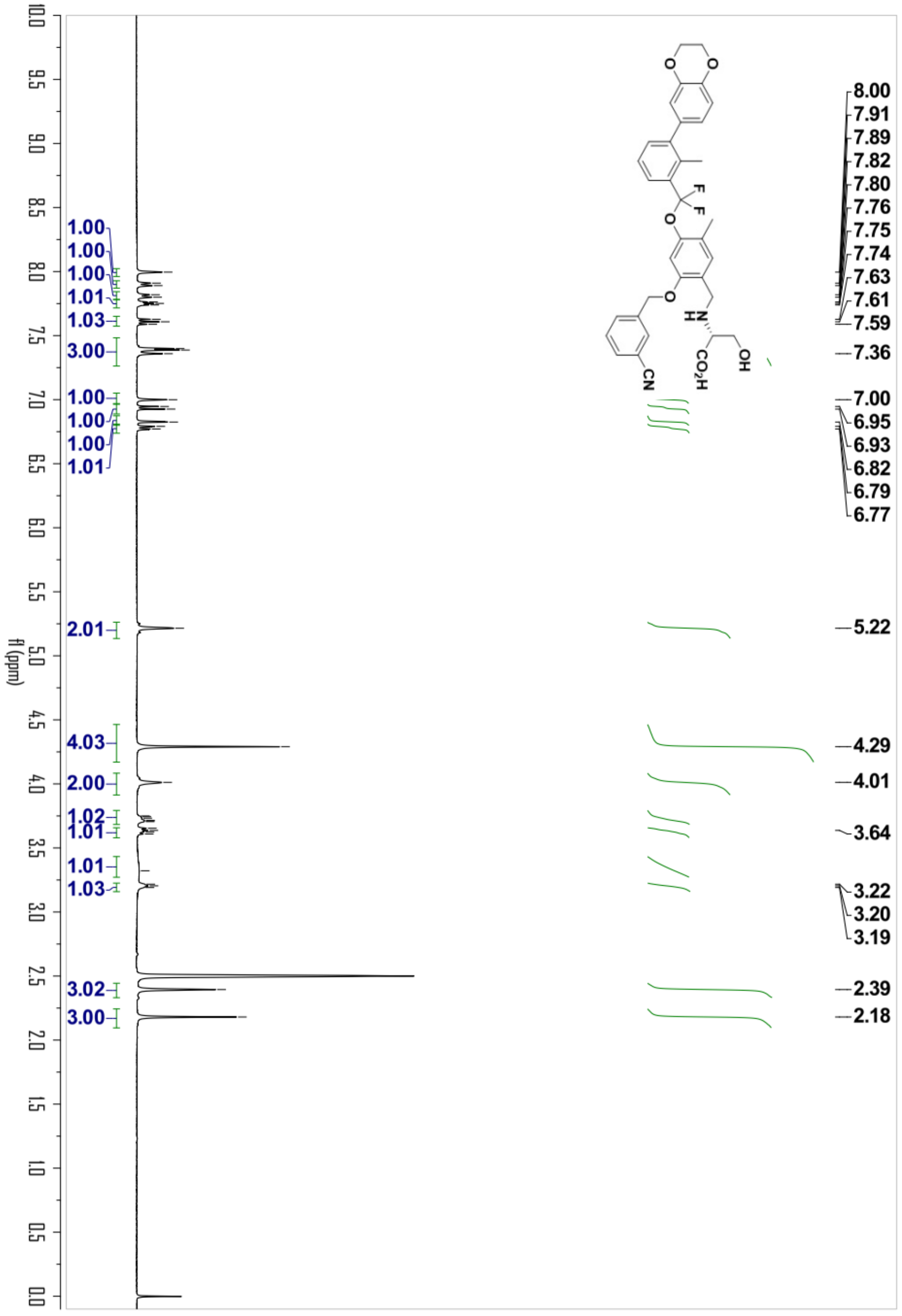

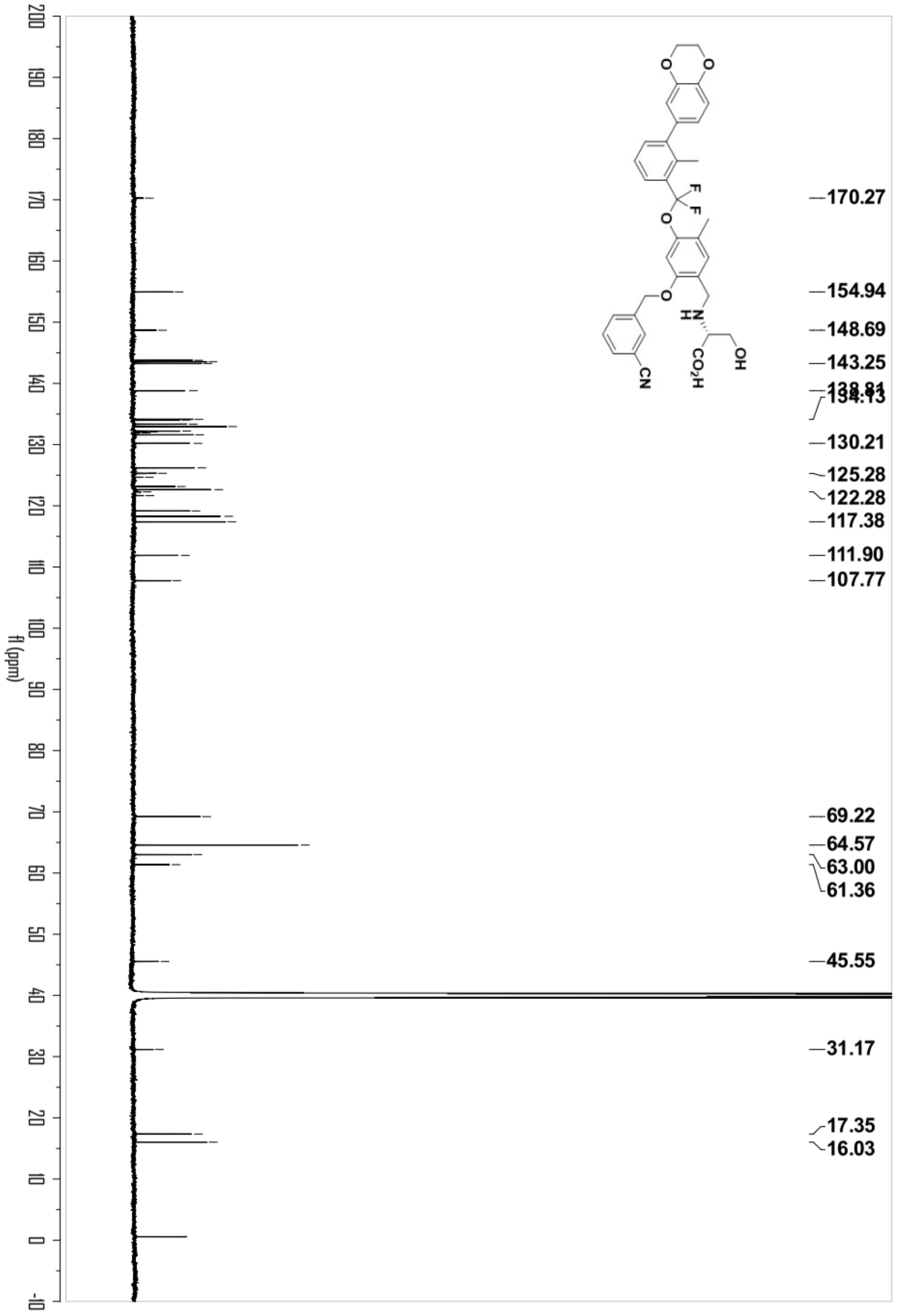
170.27
154.94
148.69
143.25
138.81
134.13
130.21
125.28
122.28
117.38
111.90
107.77
69.22
64.57
63.00
61.36
45.55
31.17
17.35
16.03
f1 (ppm)
CN
CO2H
OH

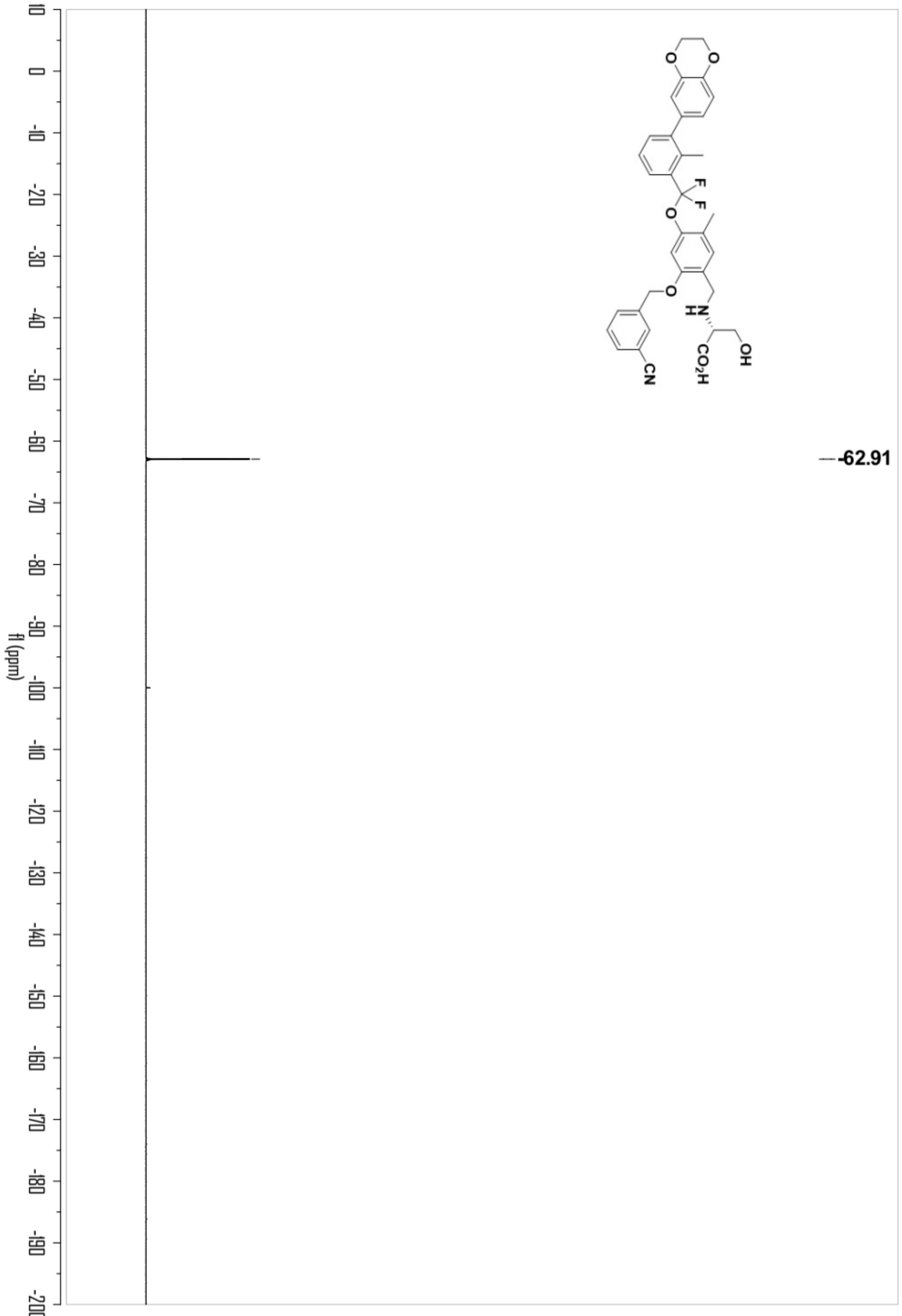
-62.91
f1 (ppm)

^{1}H, ^{13}C and ^{19}F NMR spectra of compound 9

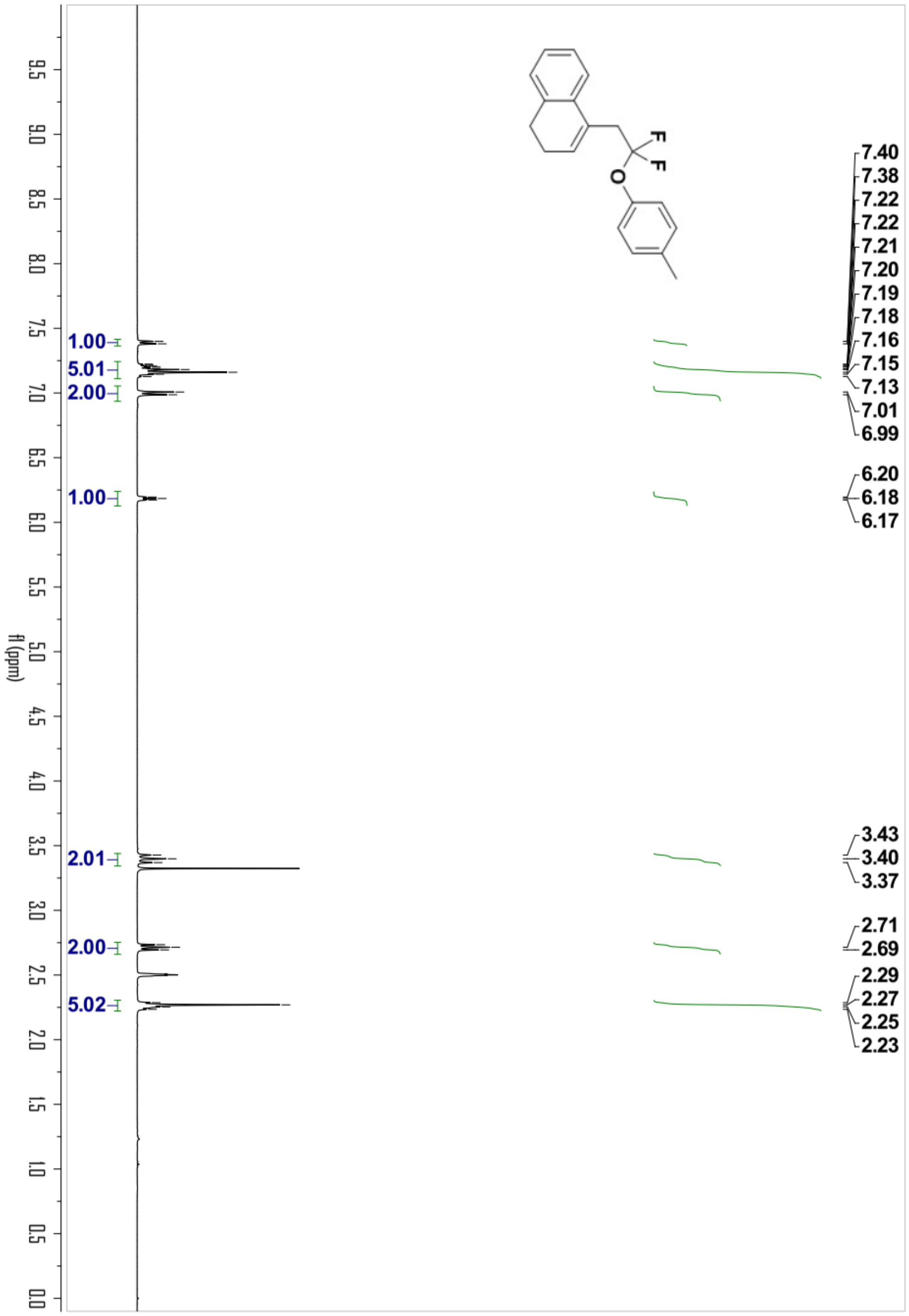

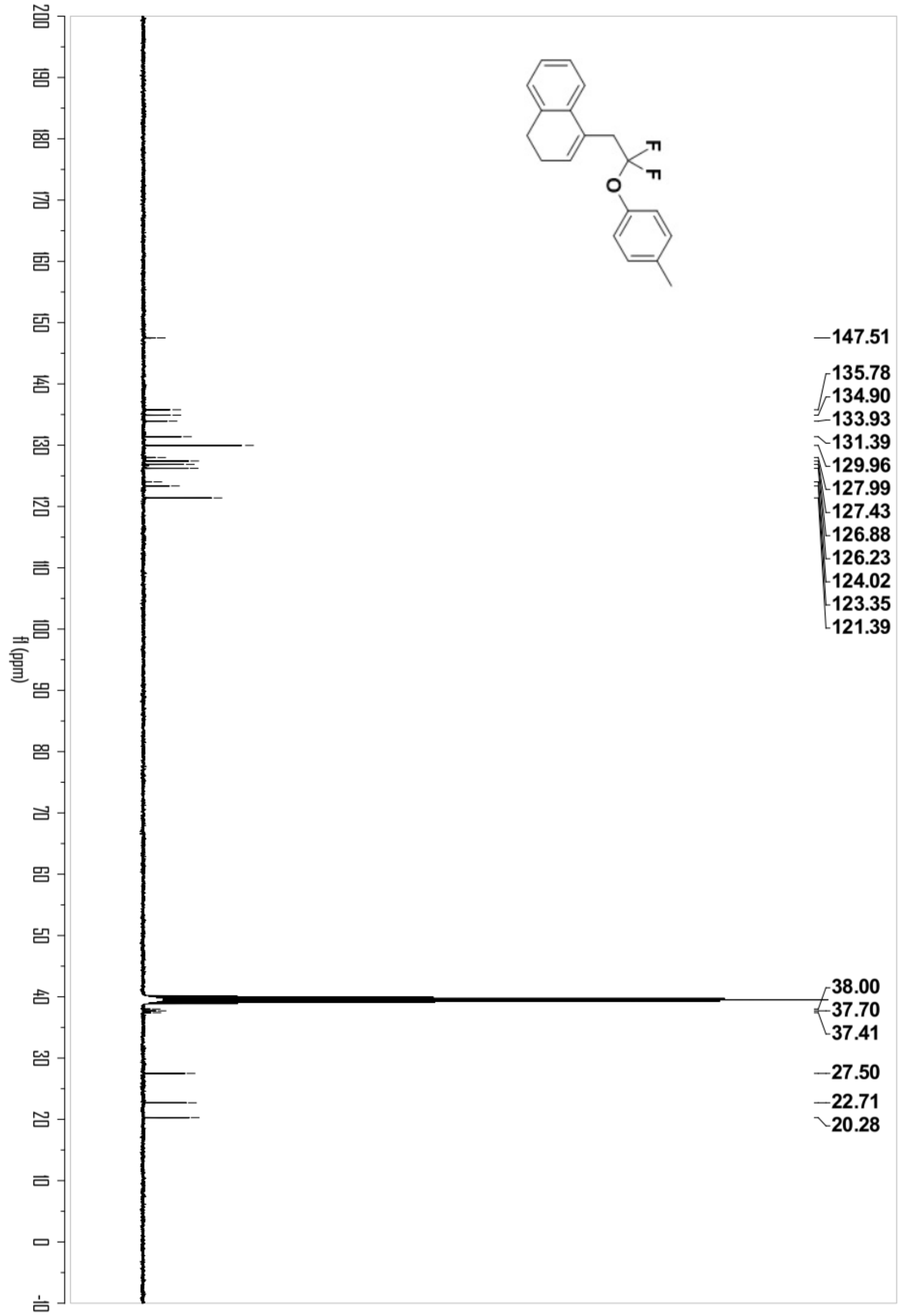
F
F
O
147.51
135.78
134.90
133.93
131.39
129.96
127.99
127.43
126.88
126.23
124.02
123.35
121.39
38.00
37.70
37.41
27.50
22.71
20.28
f1 (ppm)

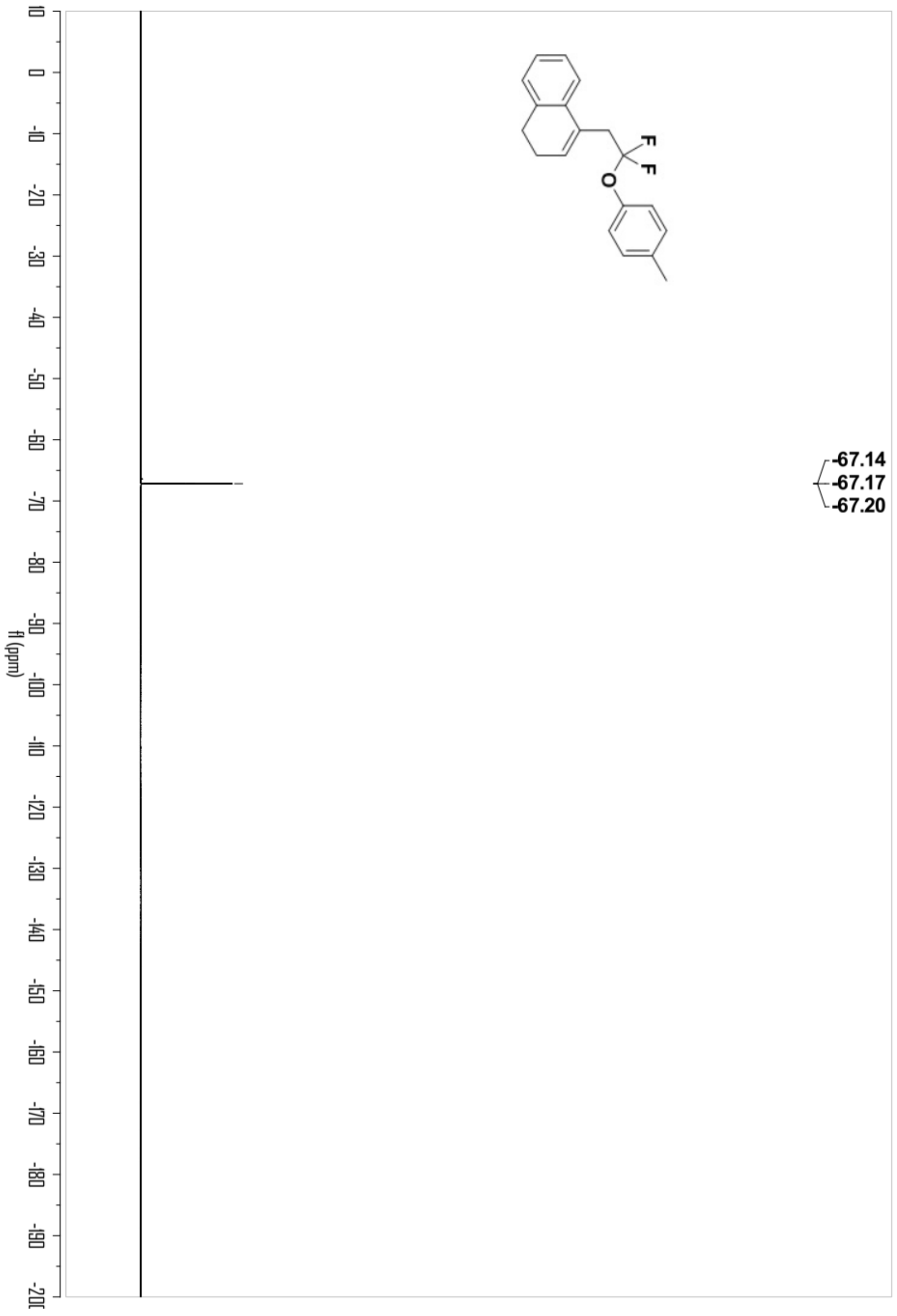
F
F
O
-67.14
-67.17
-67.20
f1 (ppm)
