# Supplementary material for: Synthesis of aryldifluoromethyl aryl ethers via nickel-catalyzed suzuki cross-coupling between aryloxydifluoromethyl bromides and boronic acids
Source: Commun Chem. 2022 Jul 4;5:78. doi: 10.1038/s42004-022-00694-4 (PMC9814959; doi:10.1038/s42004-022-00694-4)

checkCIF/PLATON report

Structure factors have been supplied for datablock(s) a_a

THIS REPORT IS FOR GUIDANCE ONLY. IF USED AS PART OF A REVIEW PROCEDURE FOR PUBLICATION, IT SHOULD NOT REPLACE THE EXPERTISE OF AN EXPERIENCED CRYSTALLOGRAPHIC REFEREE.

No syntax errors found. CIF dictionary Interpreting this report

Datablock: a_a

Bond precision: C-C = 0.0068 A Wavelength=1.54178

Cell:	a=13.9596(4)	b=14.9091(5)	c=15.0915(5)
	alpha=99.812(2)	beta=103.037(1)	gamma=103.820(2)
Temperature:	173 K		

	Calculated	Reported
Volume	2886.65(17)	2886.65(16)
Space group	P -1	P -1
Hall group	-P 1	-P 1
Moiety formula	C18 H40 N6 Ni O5, C12 H24 Br6 N4 Ni2 [+ solvent]	C12 H24 Br6 N4 Ni2, C18 H40 N6 Ni O5
Sum formula	C30 H64 Br6 N10 Ni3 O5 [+ solvent]	C30 H64 Br6 N10 Ni3 O5
Mr	1300.38	1300.50
Dx,g cm-3	1.496	1.496
Z	2	2
Mu (mm-1)	6.310	6.310
F000	1296.0	1296.0
F000’	1271.89	
h,k,lmax	16,17,18	16,17,18
Nref	10636	10599
Tmin,Tmax	0.242,0.532	0.483,0.753
Tmin’	0.155	

Correction method= # Reported T Limits: Tmin=0.483 Tmax=0.753
AbsCorr = MULTI-SCAN

Data completeness= 0.997 Theta(max)= 68.507

R(reflections)= 0.0516(8892) wR2(reflections)= 0.1428(10599)

S = 1.028 Npar= 609

The following ALERTS were generated. Each ALERT has the format
test-name_ALERT_alert-type_alert-level.
Click on the hyperlinks for more details of the test.

Alert level C

```
PLAT213_ALERT_2_C Atom C25            has ADP max/min Ratio .....        3.2 prolat
PLAT220_ALERT_2_C NonSolvent   Resd 1  C   Ueq(max)/Ueq(min) Range        3.7 Ratio
PLAT222_ALERT_3_C NonSolvent Resd 1  H   Uiso(max)/Uiso(min) Range        4.6 Ratio
PLAT230_ALERT_2_C Hirshfeld Test Diff for    O1       --C15      .        6.8 s.u.
PLAT241_ALERT_2_C High   'MainMol' Ueq as Compared to Neighbors of         O2 Check
PLAT241_ALERT_2_C High   'MainMol' Ueq as Compared to Neighbors of         O3 Check
PLAT241_ALERT_2_C High   'MainMol' Ueq as Compared to Neighbors of         O5 Check
PLAT241_ALERT_2_C High   'MainMol' Ueq as Compared to Neighbors of        C15 Check
PLAT242_ALERT_2_C Low    'MainMol' Ueq as Compared to Neighbors of        Ni3 Check
PLAT242_ALERT_2_C Low    'MainMol' Ueq as Compared to Neighbors of         N5 Check
PLAT242_ALERT_2_C Low    'MainMol' Ueq as Compared to Neighbors of         N7 Check
PLAT242_ALERT_2_C Low    'MainMol' Ueq as Compared to Neighbors of         N8 Check
PLAT242_ALERT_2_C Low    'MainMol' Ueq as Compared to Neighbors of         N9 Check
PLAT250_ALERT_2_C Large U3/U1 Ratio for Average U(i,j) Tensor ....        3.0 Note
PLAT341_ALERT_3_C Low Bond Precision on  C-C Bonds ...............    0.00683 Ang.
PLAT906_ALERT_3_C Large K Value in the Analysis of Variance ......      2.120 Check
PLAT911_ALERT_3_C Missing FCF Refl Between Thmin & STh/L=    0.600          4 Report
PLAT977_ALERT_2_C Check Negative Difference Density on H1BB             -0.38 eA-3
PLAT977_ALERT_2_C Check Negative Difference Density on H20A             -0.37 eA-3
```

Alert level G

```
PLAT002_ALERT_2_G Number of Distance or Angle Restraints on AtSite         27 Note
PLAT003_ALERT_2_G Number of Uiso or Uij Restrained non-H Atoms ...         42 Report
PLAT004_ALERT_5_G Polymeric Structure Found with Maximum Dimension          1 Info
PLAT042_ALERT_1_G Calc. and Reported MoietyFormula Strings  Differ     Please Check
PLAT083_ALERT_2_G SHELXL Second Parameter in WGHT  Unusually Large       9.72 Why ?
PLAT172_ALERT_4_G The CIF-Embedded .res File Contains DFIX Records          6 Report
PLAT173_ALERT_4_G The CIF-Embedded .res File Contains DANG Records          2 Report
PLAT176_ALERT_4_G The CIF-Embedded .res File Contains SADI Records         20 Report
PLAT177_ALERT_4_G The CIF-Embedded .res File Contains DELU Records          1 Report
PLAT178_ALERT_4_G The CIF-Embedded .res File Contains SIMU Records          2 Report
PLAT187_ALERT_4_G The CIF-Embedded .res File Contains RIGU Records          1 Report
PLAT232_ALERT_2_G Hirshfeld Test Diff (M-X)  Ni3      --O1       .        8.0 s.u.
PLAT300_ALERT_4_G Atom Site Occupancy of N11       Constrained at         0.5 Check
PLAT300_ALERT_4_G Atom Site Occupancy of N12       Constrained at         0.5 Check
PLAT300_ALERT_4_G Atom Site Occupancy of C1A       Constrained at         0.5 Check
PLAT300_ALERT_4_G Atom Site Occupancy of C1B       Constrained at         0.5 Check
PLAT300_ALERT_4_G Atom Site Occupancy of C1C       Constrained at         0.5 Check
PLAT300_ALERT_4_G Atom Site Occupancy of C1D       Constrained at         0.5 Check
PLAT300_ALERT_4_G Atom Site Occupancy of C1E       Constrained at         0.5 Check
PLAT300_ALERT_4_G Atom Site Occupancy of C1F       Constrained at         0.5 Check
PLAT300_ALERT_4_G Atom Site Occupancy of H1AA      Constrained at         0.5 Check
PLAT300_ALERT_4_G Atom Site Occupancy of H1AB      Constrained at         0.5 Check
PLAT300_ALERT_4_G Atom Site Occupancy of H1BA      Constrained at         0.5 Check
PLAT300_ALERT_4_G Atom Site Occupancy of H1BB      Constrained at         0.5 Check
PLAT300_ALERT_4_G Atom Site Occupancy of H1CB      Constrained at         0.5 Check
PLAT300_ALERT_4_G Atom Site Occupancy of H1CA      Constrained at         0.5 Check
PLAT300_ALERT_4_G Atom Site Occupancy of H1DA      Constrained at         0.5 Check
PLAT300_ALERT_4_G Atom Site Occupancy of H1DB      Constrained at         0.5 Check
PLAT300_ALERT_4_G Atom Site Occupancy of H1EB      Constrained at         0.5 Check
PLAT300_ALERT_4_G Atom Site Occupancy of H1EA      Constrained at         0.5 Check
PLAT300_ALERT_4_G Atom Site Occupancy of H1FA      Constrained at         0.5 Check
PLAT300_ALERT_4_G Atom Site Occupancy of H1FB      Constrained at         0.5 Check
PLAT301_ALERT_3_G Main Residue  Disorder .............(Resd  1  )         40% Note
```

```
PLAT380_ALERT_4_G Incorrectly? Oriented X(sp2)-Methyl Moiety .....        C13 Check
PLAT380_ALERT_4_G Incorrectly? Oriented X(sp2)-Methyl Moiety .....        C16 Check
PLAT606_ALERT_4_G Solvent Accessible VOID(S) in Structure ........          ! Info
PLAT720_ALERT_4_G Number of Unusual/Non-Standard Labels ..........         12 Note
PLAT789_ALERT_4_G Atoms with Negative _atom_site_disorder_group  #         20 Check
PLAT794_ALERT_5_G Tentative Bond Valency for Ni2       (II)      .       1.86 Info
PLAT860_ALERT_3_G Number of Least-Squares Restraints .............        530 Note
PLAT912_ALERT_4_G Missing # of FCF Reflections Above STh/L=  0.600         34 Note
PLAT913_ALERT_3_G Missing # of Very Strong Reflections in FCF ....          1 Note
PLAT978_ALERT_2_G Number C-C Bonds with Positive Residual Density.          0 Info
```

```
 0 ALERT level A = Most likely a serious problem - resolve or explain
 0 ALERT level B = A potentially serious problem, consider carefully
19 ALERT level C = Check. Ensure it is not caused by an omission or oversight
43 ALERT level G = General information/check it is not something unexpected

 1 ALERT type 1 CIF construction/syntax error, inconsistent or missing data
20 ALERT type 2 Indicator that the structure model may be wrong or deficient
 7 ALERT type 3 Indicator that the structure quality may be low
32 ALERT type 4 Improvement, methodology, query or suggestion
 2 ALERT type 5 Informative message, check
```

It is advisable to attempt to resolve as many as possible of the alerts in all categories. Often the minor alerts point to easily fixed oversights, errors and omissions in your CIF or refinement strategy, so attention to these fine details can be worthwhile. In order to resolve some of the more serious problems it may be necessary to carry out additional measurements or structure refinements. However, the purpose of your study may justify the reported deviations and the more serious of these should normally be commented upon in the discussion or experimental section of a paper or in the "special_details" fields of the CIF. checkCIF was carefully designed to identify outliers and unusual parameters, but every test has its limitations and alerts that are not important in a particular case may appear. Conversely, the absence of alerts does not guarantee there are no aspects of the results needing attention. It is up to the individual to critically assess their own results and, if necessary, seek expert advice.

Publication of your CIF in IUCr journals

A basic structural check has been run on your CIF. These basic checks will be run on all CIFs submitted for publication in IUCr journals (*Acta Crystallographica*, *Journal of Applied Crystallography*, *Journal of Synchrotron Radiation*); however, if you intend to submit to *Acta Crystallographica Section C* or *E* or *IUCrData*, you should make sure that full publication checks are run on the final version of your CIF prior to submission.

Publication of your CIF in other journals

Please refer to the *Notes for Authors* of the relevant journal for any special instructions relating to CIF submission.

PLATON version of 05/12/2020; check.def file version of 05/12/2020

Datablock a_a - ellipsoid plot

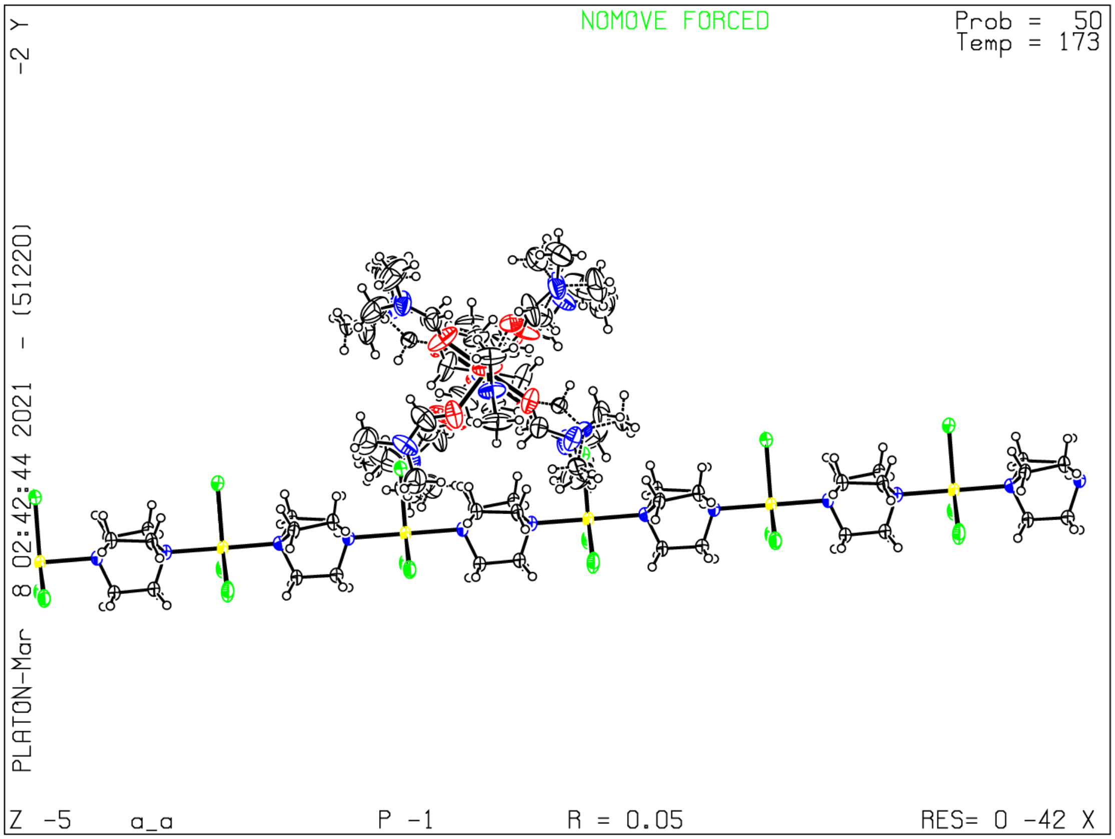

Supplement: Supplementary file 5 — Supplementary Data 3 [file 42004_2022_694_MOESM5_ESM.pdf]
