# Supplementary material for: Synthesis of aryldifluoromethyl aryl ethers via nickel-catalyzed suzuki cross-coupling between aryloxydifluoromethyl bromides and boronic acids
Source: Commun Chem. 2022 Jul 4;5:78. doi: 10.1038/s42004-022-00694-4 (PMC9814959; doi:10.1038/s42004-022-00694-4)
Supplement: Supplementary file 7 — Description of Additional Supplementary Files [file 42004_2022_694_MOESM7_ESM.docx]

Description of Additional Supplementary Files

**File name:** Supplementary Data 1

**Description:** all NMR spectra

**File name:** Supplementary Data 2

**Description:** Supplementary Data 2 file includes the single crystal structure of compound Ni-11

**File name:** Supplementary Data 3

**Description:** Supplementary Data 3 file includes check CIF/PLATON report of compound Ni-11**.**
